# Features of smoking cessation, alcohol reduction, and diet and physical activity interventions associated with changing behaviours during pregnancy: a systematic review of reviews

**DOI:** 10.1007/s10865-026-00702-1

**Published:** 2026-08-06

**Authors:** James J. Newham, Maria Raisa Jessica Aquino, Daniel Jones, Louise Kingston, Liane Azevedo, Anna Boath, Louise Hayes, Catherine McParlin, Shona Haston, Nicola Heslehurst

**Affiliations:** 1 School of Psychology, Faculty of Health and Wellbeing, Northumberland Building Room 146, Northumbria University, Newcastle upon Tyne, NE1 8ST UK; 2 Newcastle University, Newcastle upon Tyne, UK; 3 Teesside University, Middlesbrough, UK; 4 Sheffield Hallam University, Sheffield, UK; 5 University of Edinburgh, Edinburgh, UK

**Keywords:** Interventions, Behaviour change, Lifestyle, Pregnancy, Systematic review

## Abstract

Systematic reviews of smoking, alcohol, diet and physical activity (PA) behaviour change interventions in pregnancy have high heterogeneity in effectiveness estimates for maternal behaviours and pregnancy outcomes, and it is unclear which features of interventions are integral to promoting effectiveness. This systematic review of reviews aimed to compare findings to identify any shared behaviour change techniques (BCTs), content, or delivery of interventions that may be associated with effectiveness across diet, physical activity, smoking and alcohol behaviours, and to identify intervention features showing promise in targeting one behaviour but have yet to be tested in addressing other behaviours. The search strategy covered reviews published between 2013 and 2025 and identified 66 reviews (9 smoking, 6 alcohol, 50 diet/PA, 1 smoking, diet, and/or PA). Diet/PA interventions should begin in the first trimester, run for a longer duration, and entail frequent engagement via a range of modalities in a supervised format (ideally from a specialist provider). Smoking intervention effectiveness was consistently associated with incentives and counselling. Brief interventions were consistently reported as effective interventions for alcohol use but there was a lack of standardisation in delivery. BCTs from the "goals and planning" and "feedback and monitoring" domains were routinely associated with effectiveness. Further research is required to explore intervention features that have shown promise in one behaviour but have yet to be tested for other behaviours, such as incentives in alcohol interventions.

**Supplementary Information:**

The online version contains supplementary material available at https://doi.org/10.1007/s10865-026-00702-1.

## Introduction

Many health behaviours during pregnancy are known to increase the risk of adverse health outcomes in the perinatal period (e.g. gestational diabetes (GDM)) and potentially across the mother’s and infant’s lifespan (e.g. future risk of type 2 diabetes) (Bellamy et al., 2009; Morniroli et al., 2023). Behaviours with significant risk to the mother and fetus include smoking, alcohol use, poor diet, and physical inactivity. Smoking during pregnancy is an established risk factor for sudden infant death syndrome, asthma, oral clefts, stillbirth, and low birth weight (Avşar et al., 2021) with the prevalence of smoking in pregnancy as high as 20% in some countries (Lange et al., 2018). Alcohol use in pregnancy is associated with preterm birth, low birth weight, and fetal alcohol disorders, with prevalence around 10% globally but as high as 25% in Europe (Popova et al., 2023a, 2023b). Poor diet and low levels of physical activity (PA) increase the likelihood of obesity, GDM, and hypertensive disorders in the mother, and infants born large-for-gestational age; with a global prevalence of obesity at 20.9% and increasing annually (Kent et al., 2024). Given the high prevalence of these behaviours and associated risks, it is a public health priority to identify effective interventions for behaviour change during pregnancy.

Pregnancy is considered a ‘teachable moment’ where individuals may be motivated to adopt risk-reducing health behaviours (McBride et al., 2003). Underlying assumptions are that behaviour change is partly driven by women prioritising the health of their baby and responding to social norms on the acceptability of certain behaviours in pregnancy. These changes are supported by healthcare services offering women behaviour change advice on smoking cessation, alcohol consumption, PA and diet (Evenson et al., 2014; Thibaut et al., 2019; Tsakiridis et al., 2020). Yet despite potentially increased motivation from mothers and support from healthcare providers, many interventions often prove ineffective (Rockliffe et al., 2021). Potential explanations are that intervention designs are extrapolated from non-pregnant populations without consideration of the unique determinants of behaviour in pregnancy (Kelly & Barker, 2016; Rockliffe et al., 2021). In addition, many pregnant women engage in multiple negative health behaviours (1 in 8 pregnant women report clustered preventable risk factors) and yet guidance primarily adopts a single-behaviour approach (Desmet et al., 2023). The field of multiple-behaviour change (MBHC) is limited and complex, with few published interventions, a focus on addictive behaviour and disease prevention rather than promoting healthy behaviours, and difficulty identifying whether sequential or simultaneous MBHC is more effective (Maisano et al., 2020); and the evidence is sparser for pregnant populations. Previous comparison on interventions targeting MHBC versus a single behaviour are shown to include more behaviour change technique (BCT) content but increased content may be overburdensome and lead to ego depletion, and these self-regulatory resources may be more limited when pregnant and preparing for motherhood (Maisano et al., 2020; McSharry et al., 2015; Rockliffe et al., 2021). However, most women want support for these risk factors (Desmet et al., 2023) and healthcare providers report difficulty addressing multiple behaviours simultaneously and interventions may require variation in design to address this (Heslehurst et al., 2014; McSharry et al., 2015). The increasing need for multiple parallel referral systems, pathways, guidelines, and interventions presents a significant burden to women, healthcare professionals and services trying to manage complex pregnancies (Smith et al., 2009); and there is a need to streamline the content of interventions to identify the most salient BCTs that work across behaviours.

Multiple systematic reviews have been conducted relating to the effectiveness of smoking, alcohol, diet and PA behaviour change interventions on improving maternal behaviours (Heslehurst et al., 2020) and pregnancy outcomes (Hayes et al., 2021). However, reviews tend to be carried out in silos without an attempt to synthesise across behaviours, despite the overlap in target population, health professionals caring for this population, and comparable health outcomes. Therefore, it is essential to have a better understanding of whether there are any patterns in elements of intervention design, content, and delivery that are associated with change across behaviours. Umbrella reviews provide a comprehensive, high-level synthesis of a broad evidence base by integrating findings across multiple systematic reviews, thereby improving accessibility, highlighting gaps, and supporting informed decision-making. This approach not only summarises but identifies areas of agreement and discrepancy in the high-level overviews that systematic reviews individually provide. This is the third and final paper from a wider programme of systematic reviews of systematic reviews exploring diet, PA, smoking and alcohol behavioural interventions delivered during pregnancy. The first (Heslehurst et al., 2020) examined the effectiveness of interventions on changing maternal behaviours in pregnancy and concluded consistent evidence for interventions’ ability to improve healthy diet behaviours related to dietary quality, improve some PA outcomes (i.e., increase in energy expenditure and fitness levels), and reducing smoking. The second (Hayes et al., 2021) examined the effectiveness of interventions on improving health-related outcomes and reported consistent evidence from diet/PA interventions for reducing gestational weight gain (GWG), and larger reviews also reported a significant reduction in postnatal weight retention, caesarean delivery, preeclampsia, hypertension, GDM and preterm delivery. For smoking cessation interventions there was evidence of significantly increased birth weight. These two reviews treated interventions as homogeneous groups (e.g., “smoking cessation interventions”) without investigating the specific components or underlying mechanisms of individual interventions that may drive their effects on outcomes. In addition, it is unclear to what extent other systematic reviews have attempted to identify intervention components associated with effectiveness. The aim of this third and final review in the series is to compares findings of previous systematic reviews to identify any shared behaviour change techniques (BCTs), content, or delivery of interventions that may be associated with effectiveness across and within diet/PA, smoking and alcohol behaviours. The results of this umbrella review provide researchers and practitioners with evidence of intervention content (BCTs, therapeutic approaches) and context (setting, mode of delivery, intensity) that is associated with effectiveness across individual and multiple behaviours, and highlights intervention features that shows promise in targeting one behaviour but have yet to be tested in addressing other behaviours.

## Materials and methods

We used the Joanna Briggs Institute (JBI) methodology for umbrella reviews (Joanna Briggs Institute, 2015) and the PRISMA reporting guidelines and checklist (Moher et al., 2009). The protocol for this programme of systematic reviews has been published (Dinsdale et al., 2016) and was registered on the PROSPERO database (CRD42016046302). For this programme of reviews, a comprehensive search of fourteen bibliographic databases was originally conducted in May 2016, followed by a series of updates, the most recent in June 2025 to encompass reviews published since 2013 (Supplement 1). All full-text screening from the first two reviews in this series was repeated with the new inclusion criteria applied. The databases searches for this third review were updated to identify any published systematic reviews since the original searches were conducted for previous reviews. Following de-duplication, titles and abstracts were screened against the inclusion and exclusion criteria independently in duplicate. Full-text screening for the updated search and the previous reviews was conducted by one reviewer used a pre-defined template, with another reviewer validating screening decisions, and reasons for exclusion were recorded. Screening disagreements were discussed between reviewers and a third reviewer if required.

### Inclusion and exclusion criteria

The inclusion criteria are based on population, intervention, comparator, outcome and study design (PICOS). The population (P) was pregnant women. We excluded systematic reviews reporting interventions delivered exclusively during the preconception or postnatal periods, or as treatments for existing conditions or specific sub-groups (e.g., women diagnosed with GDM or having obesity). The interventions (I) needed to target maternal smoking, alcohol, diet, or PA behaviours. Pharmacological and dietary supplement interventions were excluded. Peer-reviewed systematic reviews (S) could include any type of comparator group (C). The outcomes (O) of interest were authors’ reporting on intervention features’ association with effectiveness in improving health/behavioural outcomes for the mother and infant. Using Davidson et al.’s (2003) reporting criteria, intervention features included, but were not limited to, intervention content (e.g. advice only vs. advice with exercise), type of provider (e.g. peer vs. clinician), mode of delivery (e.g. individual vs group-based, online vs in-person, supervised vs. unsupervised), and intensity/dose (features relating to frequency and duration of intervention contacts and/or magnitude of performance required e.g. low energy expenditure vs high energy expenditure exercise regimes). Systematic reviews focussing exclusively on one feature were eligible (e.g., only digital interventions). No restrictions were placed on the behaviours or outcomes that intervention features were associated with. Systematic reviews were excluded if they provided no synthesis of the studies’ findings or had no analysis on how effectiveness was impacted by different intervention features.

### Data extraction and quality assessment (QA)

All systematic reviews which met the inclusion criteria underwent data extraction using a standardised template, and the JBI critical appraisal instrument for umbrella reviews (Joanna Briggs Institute, 2014) was used to assess the methodological quality (Supplement 2). The data extraction and QA tools were piloted by all authors on a subset of studies and refined to improve consistency between reviewers. Studies were divided between pairs of authors to independently extract study characteristic data (Table 1) and extraction was compared for accuracy after completing all papers. Similarly, two experts in behaviour change coding (JN, MRJA) and QA (JN, LA) independently extracted behaviour change content and QA content across 10% of papers. Minor discrepancies identified during this validation process were documented and resolved through discussion between the two reviewers prior to continuing to extract data independently for all papers. Any inaccuracies were compared and subsequently resolved. To prevent bias from selective reporting across reviews, only conclusions from authors of the systematic reviews were extracted.

**Table 1:** Summary of included systematic reviews

	Systematic reviews reporting smoking interventions (n = 10)	Systematic reviews reporting alcohol interventions (n = 6)	Systematic reviews reporting diet and/or physical activity interventions (n = 51)
*Time period of included intervention studies in the reviews*			
Range in years:	1976 to 2021	1982 to 2020	1974 to 2024
Not reported *(n)*	0	1	2
*Search strategies*			
Databases only (*n*)	2	3	13
Supplementary searches (*n*)	8	3	38
*Included study designs*			
RCT only (*n*)	4	4	31
RCT + other design (*n*)	6	1	20
Not reported (*n*)	0	1	0
*Number included studies in the systematic reviews*			
Range (*n*)	7 to 88	6 to 25	5 to 113
Median (*n*)	27	10	19
*Pooled sample sizes included in the systematic reviews*			
Range (*n* women)	1009 to 69,082	972 to 8467	363 to 273,182
Median (*n* women)	6581	2198	5084
Not reported (*n*)	1	1	1
*Countries of intervention studies included in the systematic reviews (number of reviews with these countries represented)*			
Countries not reported*	3	1	13
HICs represented in the included interventions	13 countries: Australia (4), Canada (5), Germany (1), Greece (1), Japan (1), Netherlands (2), New Zealand (2), Norway (2), Poland (2), Spain (3), Sweden (2), UK (7), USA (7)	9 countries: Australia (1), Ireland (1), Israel (1), Netherlands (5), Norway (1), Spain (3), Sweden (1), UK (2), USA (5)	28 countries: Australia (26), Belgium (10), Canada (18), Chile (2), Croatia (7), Cyprus (2), Denmark (16), Finland (18), France (1), Germany (8), Greece (4), Ireland (8), Italy (12), Japan (7), Netherlands (12), New Zealand (11), Norway (23), Poland (2), Portugal (1), Slovenia (1), South Korea (2), Spain (23), Sweden (12), Switzerland (1), Taiwan (6), UK (14), USA (33)
Middle Income Countries represented in the included interventions	6 countries represented: Argentina (2), Brazil (1), Cuba (1), Mexico (1), South Africa (1), Turkey (1)	3 countries represented: Argentina (1), Brazil (1), South Africa (2)	17 countries represented: Argentina (1), Benin (2), Brazil (23), China (13), Colombia (8), Egypt (3), India (9), Indonesia (2), Iran (12), Kosovo (2), Mexico (1), Pakistan (2), Serbia (1), South Africa (3), Thailand (3), Turkey (9)
Low Income Countries represented in the included interventions	No countries represented	No countries represented	1 country represented: Gambia (1)

### Evidence synthesis

Supplementary tables 3–6 include the characteristics of the included systematic reviews, quality of the reviews, overlap of included studies within reviews, and author reported conflicts of interest. The analysis of results used a systematic narrative synthesis approach (Popay et al., 2006). Reviews were first grouped by target behaviour: diet/PA; smoking; alcohol. Due to the frequent reporting of diet and/or PA in the same reviews, these were combined in the synthesis. For each target behaviour group, thematic categories were developed to group and discuss the evidence based on the systematic reviews’ inclusion criteria as some were broad in inclusion criteria (e.g. included any intervention regardless of any specific feature) and others focus on a specific intervention feature (e.g., technology-supported mode of delivery). Data extracted from reviews with broad inclusion criteria focused on sub-group or sensitivity-analyses that compared specific intervention features and how they may be associated with effectiveness. Whereas data extracted from reviews that focused on specific intervention features often focused on the authors overall conclusions relating to effectiveness.

Results for each theme are presented in tabular format with narrative discussion. Supplementary tables 7–11 describe study findings using a Red-Amber-Green (RAG) rating. Green indicates effectiveness associated with intervention feature evidenced by a statistical test, meta-analysis or consensus across studies. Amber indicates possibility of an association between intervention features and effectiveness based on observed trends in the data but with no statistical evidence of significance (e.g. all the successful interventions were observed to have had a BCT in common but no robust analysis). Red indicates no clear association between intervention feature and effectiveness found or tested. The synthesis has prioritised green-rated findings and red- and amber-findings have been reported on when they challenge or contextualise green-rated findings.

## Results

**Fig. 1:**
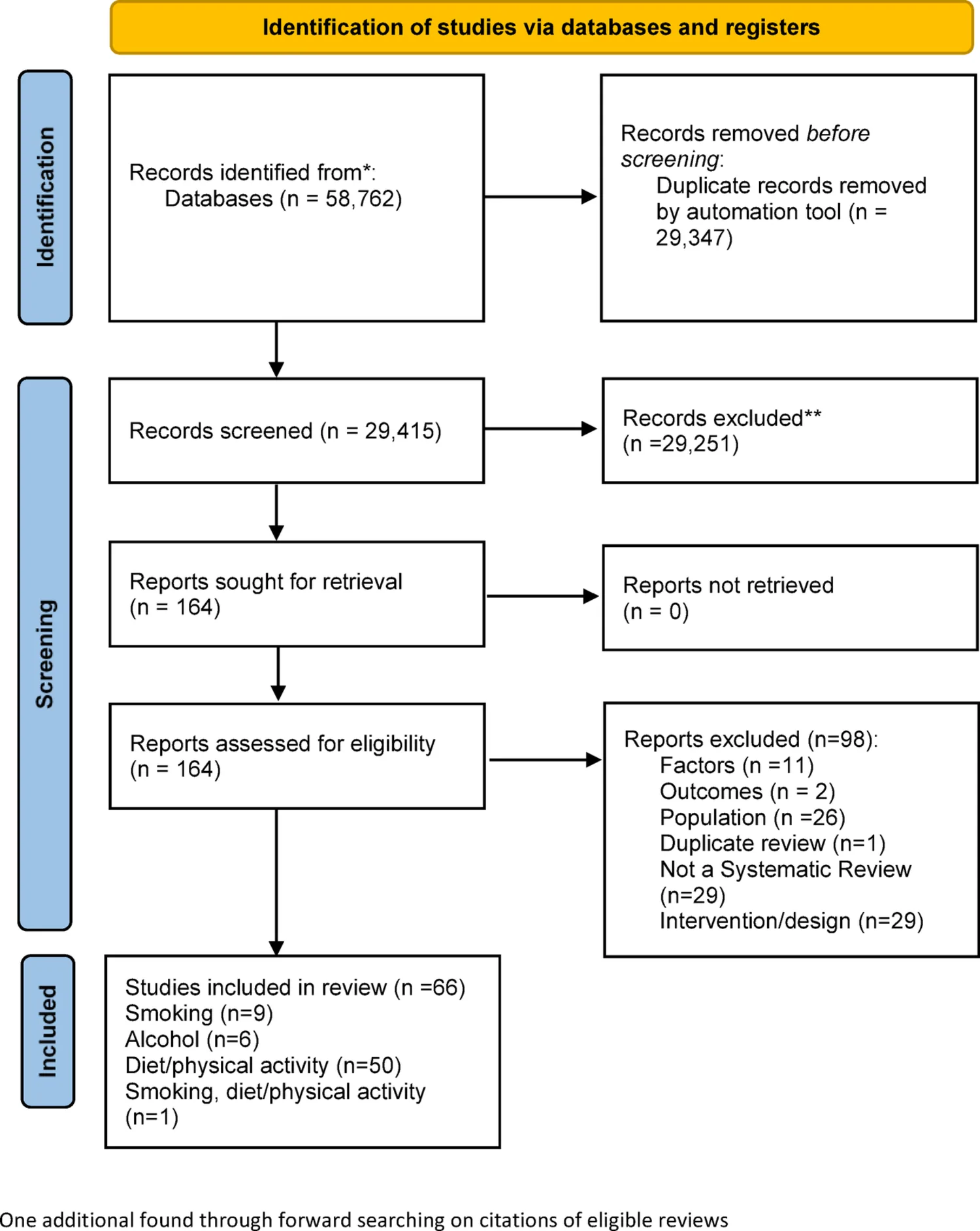
PRISMA flow diagram

The database searches and the searches from previous reviews identified 58,762 records, which were reduced to 29,415 after deduplication. 29,251 records were excluded at title and abstract screening and following full text screening 66 systematic reviews were included in this review (9 smoking, 6 alcohol, 50 diet/PA, 1 smoking, diet, and/or PA) (Fig. 1). Detailed overviews of the included reviews are provided in Supplement 3. Across all three behaviours, reviews’ included studies were published in similar time periods and median sample sizes ranged from 2198 to 6581 participants (Table 1). Reviews reporting alcohol and diet/PA were more likely to be restricted to randomised control trials, whereas smoking reviews were more likely to include other intervention study designs. There were differences observed in the number of included studies in reviews for different behaviours, with alcohol reviews having the lowest number of included studies (median 10) whereas diet/PA reviews had a median of 19, and smoking reviews had a median of 27. Across all behaviours, reviews were most likely to include studies from high-income countries, with some representation of middle-income countries across all behavioural domains. Only one diet/PA review, and no alcohol or smoking reviews, included studies from low-income countries.

### Quality of included reviews

The included reviews were predominantly high-quality (74%), with 24% moderate quality, and one deemed low quality (Table 2). A total of 14 out of the 66 reviews achieved a maximum score of 11. When comparing the QA of reviews across different behaviours (Table 2; Supplement 4), 80% of the smoking reviews, 75% of the diet/PA reviews, and 66% of alcohol reviews were considered high quality. Between 76 and 98% of reviews met each quality assessment criteria, except only 50% of the reviews assessed publication bias, 61% had two reviewers independently appraise studies, and 65% had methods to minimise errors in data extraction.

**Table 2:** Summary of the quality assessment of included systematic reviews

Intervention type	Quality assessment question											Total score	Quality category
	1	2	3	4	5	6	7	8	9	10	11		
Alcohol reviews	6/6 100%	5/6 83%	5/6 83%	5/6 83%	5/6 83%	3/6 50%	4/6 66%	4/6 66%	3/6 50%	6/6 100%	6/6 100%	Range 3–11	17% Low (1/6) 17% Low (1/6) 66% High (4/6)
Smoking reviews	10/10 100%	10/10 100%	10/10 100%	7/10 70%	7/10 70%	6/10 60%	8/10 80%	10/10 100%	5/10 50%	10/10 100%	10/10 100%	Range 6–11	20% Moderate (2/10) 80% High (8/10)
Diet and/or physical activity reviews	50/51 98%	46/51 90%	49/51 96%	39/51 76%	44/51 86%	24/51 46%	32/51 62%	46/51 90%	27/51 54%	43/51 84%	44/51 86%	Range 6–11	25% Moderate (13/51) 75% High (38/51)
Total for all behaviours	65/66 98%	60/66 91%	63/66 95%	50/66 76%	55/66 83%	40/66 61%	43/66 65%	59/66 89%	33/66 50%	58/66 88%	59/66 89%	Range 3–11	1% Low (1/66) 24% Moderate (16/66) 74% High (49/66)

Note1: (1) Is the review question clearly and explicitly stated?; (2) Were the inclusion criteria appropriate for the review question?; (3) Was the search strategy appropriate?; (4) Were the sources and resources used to search for studies adequate?; (5) Were the criteria for appraising studies appropriate?; (6) Was critical appraisal conducted by two or more reviewers independently?; (7) Were there methods to minimize errors in data extraction?; (8) Were the methods used to combine studies appropriate?; (9) Was the likelihood of publication bias assessed?; (10) Were recommendations for policy and/or practice supported by the reported data?; (11) Were the specific directives for new research appropriate?. For total score 1 is given if yes otherwise it is zero. For quality: Low quality is 0–3. Moderate quality is 4–7. High quality is 8–11

Note2: Please note that the study by Hussain et al., 2020 is counted twice in the smoking reviews and the diet and/or physical activity reviews, but it is counted only once in the total

### Overlap of included studies

Umbrella reviews inevitably include original studies reported by more than one systematic review and a detailed overview of the overlap in studies is provided in Supplement 5. Five of the 6 included alcohol reviews cited a total of 73 studies, and one alcohol review did not report details of the included studies. After removal of duplicate citations across more than one review, there were 44 unique studies included. The 10 smoking reviews yielded 498 citations; with 303 unique publications. The 51 diet/PA reviews had a total of 1567 citations including 719 unique publications.

### Conflict of interests

The conflicts of interest are reported in Supplement 6. From the 66 systematic reviews included, 5 did not provide a statement of conflict of interest (n = 2 smoking and n = 3 diet/PA reviews). Among the remaining 60 reviews that did provide conflict of interest statements, 56 reported having no conflict of interest. Four reported author conflicts of interest including receiving funding from organisations with a potential conflict of interest, receiving awards or being on the editorial board of the journal where the systematic review was published.

### Synthesis

The number of alcohol and smoking intervention reviews were small enough that findings could be compared without further thematic groupings (n = 6 and n = 10, respectively). However, to gain meaning from across the larger number of diet/PA reviews (n = 51), reviews were divided into the following categories based on their inclusion criteria: any diet/PA interventions (n = 11), PA-focused interventions (n = 29), diet-focused interventions (n = 6), MHealth interventions (n = 5). There were several rationales for these groupings and the observations that informed these groupings gave insight into how reviews in this field have been structured, how intervention features can be examined, and which outcomes have been prioritised. First, the 11 reviews with broad inclusion criteria examining any diet/PA intervention often focused on only one or two outcomes. Therefore, findings from across these reviews were grouped by outcome (Supplement 7). In contrast, the reviews with specific inclusion criteria tended to report on multiple outcomes and so subsequent tables are not grouped by outcome. Secondly, ‘PA-focused interventions’ and ‘diet-focused interventions’ were reviews where eligible interventions had to have a PA or diet component, respectively, but may have components that also target the other behaviour (Supplements 8–9). Beyond the increased number of reviews of PA-focused interventions, the two groups focused on different outcomes, with a greater proportion of reviews of diet-focused interventions reporting neonatal outcomes (e.g., birthweight) (4/6; 66.6%) than reviews of PA-focused interventions (12/29; 41.4%). Third, while attempts were made to group by specific intervention features to isolate their effect, this was complicated as many intervention features relating to intensity and dose were intertwined (e.g. frequency, duration). However, reviews of interventions delivered by phone call, text, smartphone app, or online (MHealth) were a group that were easily separated for insight into the impact of mode of delivery (Supplement 10).

#### Diet and/or PA interventions (n = 51 reviews)

##### Any diet and/or PA interventions (n = 11 reviews)

Of the 11 reviews that included any intervention, 9 reported on GWG (Agha et al., 2014; Cantor et al., 2021; Craemer et al., 2019; Harrison et al., 2023; Hill et al., 2013; Muktabhant et al., 2015; Skouteris et al., 2014; Soltani et al., 2016; Walker et al., 2018), and 3 reported on GDM (Cantor et al., 2021; Guo et al., 2019; Skouteris et al., 2014). Two reviews reported on caesarean delivery, and macrosomia/LGA (Cantor et al., 2019; Muktabhant et al., 2015). A single review reported on hypertension (Cantor et al., 2021), pre-eclampsia (Allen et al., 2014), and preterm birth (Cantor et al., 2021).

There was mixed evidence related to links between GWG and intervention content, mode of delivery, and dose. Regarding content, Agha et al. (2014) reported no difference between passive (advice-only) vs active interventions, whereas Cantor et al. (2021) reported that active interventions were associated with reduced likelihood of excessive GWG. Regarding the association of supervision and effectiveness, the findings were mixed: Harrison et al (2023) reported facilitator-led interventions were significantly more effective than with no facilitator; Muktabhant et al. (2015) reported no overall difference between supervised and unsupervised interventions, but found the largest reduction in GWG was observed for supervised interventions; and Craemer et al. (2019) reported no difference between supervised and unsupervised exercise programs. Given Muktabhant et al (2015) was the only of these reviews to have high quality evidence (Supplementary Table 4) and Harrison et al (2023) had a larger number of more recent studies (Supplementary Table 3), this suggests their evidence was more reliable than Craemer’s finding of no association. Regarding dose, a higher frequency of intervention sessions was associated with improve effectiveness in several studies (Cantor et al., 2021; Harrison et al., 2023; Skouteris et al., 2014), yet Walker et al (2018) reported neither frequency of sessions, intensity of intervention, trimester-initiated, nor duration influenced intervention effectiveness. However, Walker et al (2018) did report PA intervention effectiveness in changing GWG was significantly higher in studies that combined individual and group-delivery of interventions, compared with studies that were delivered to individuals only or groups only. Conversely, Harrison et al. (2023) reported that individual delivery was associated with increased effectiveness for dietary interventions, but delivery format was not a significant factor for PA-only or mixed interventions across interventions. Regarding setting, Agha et al. (2014) reported that only hospital-delivered interventions were effective in reducing GWG.

There was some evidence that specific BCT theory might be associated with effectiveness. Hill et al. (2013) reported that interventions incorporating four specific BCTs were more effective: “providing information on the consequences of behaviour to the individual”, “provide rewards contingent on successful behaviour”, “prompt self-monitoring of behaviour”, and “motivational interviewing”. Conversely, the following BCTs were not used in more effective interventions: outcome goal setting, action planning, prompt rewards contingent on effort of progress towards behaviour, prompt self-monitoring of behavioural outcome, use of follow-up prompts, and facilitate social comparison. Soltani et al (2016) reported that “feedback and monitoring,” “shaping knowledge,” and “goals and planning” appeared the most successful BCT categories, and noted that successful interventions always included BCTs from one or both of “goals and planning” or “feedback and monitoring”. Skouteris et al (2014) reported that “self-monitoring” and “goal setting” were used in studies that provided evidence of preventing excessive GWG. Agha et al (2014) reported that feedback on participants’ progress and weight monitoring during pregnancy were more commonly used in successful interventions; however, sub-analyses showed no difference to control groups for interventions with or without feedback and/or weight monitoring. Hill et al (2013) reported no difference in intervention effectiveness between interventions based on theory and those that were not.

Reviews provided minimal evidence of what was effective for GDM. Cantor et al (2021) reported that both active and counselling-only interventions were associated with decreased risk, and that frequency of contact sessions did not influence risk reduction. However, Guo et al (2019) and Skouteris et al (2014) both reported that a high frequency of contact sessions may lead to less risk. Guo et al (2019) also found that intervention effect size was significantly positively correlated with earlier initiation of the intervention. Skouteris et al (2014) reported no conclusive evidence for benefits of individual over group-based interventions or the importance of goal setting but suggest self-monitoring was an effective BCT.

For the remaining outcomes, Cantor et al. (2021) reported that interventions with a higher frequency of contacts were associated with reduced likelihood of hypertension and macrosomia, but no association was found between intervention frequency and likelihood of caesarean delivery nor LGA infants. However, whether interventions were active/passive made no difference to incidence of macrosomia, LGA, nor caesarean delivery (Cantor et al., 2021), but active interventions reduced the likelihood of hypertension. Muktabhant et al (2015) reported that supervised interventions may improve macrosomia but made no difference on incidence of caesarean delivery. Finally, sensitivity analyses showed no reduction in risk of pre-eclampsia when interventions commenced in the first trimester compared to any trimester (Allen et al., 2014). No clear associations were found between intervention features and preterm birth (Cantor et al., 2021).

##### PA-focused interventions (n = 29 reviews)

PA-focused reviews varied considerably in inclusion criteria. Whereas some were relatively broad, others focused on specific types of PA (e.g. yoga, aquanatal exercise) or mode of delivery (e.g. group setting). Reviews with broad inclusion criteria generally concluded that *all* PA interventions did not significantly influence GWG (McDonald et al., 2016; Wang et al., 2019), GDM (Yin et al. 2013), perinatal mortality, nor labour and delivery outcomes (Davenport et al., 2018a, 2018b, 2019a, 2019b). However sub-analyses often highlighted key intervention features associated with improved outcomes. Wang et al.’s (2019) sub-analyses highlighted significant improvements for women receiving a PA intervention from the 1st to the 3rd trimester and the 2nd to the 3rd trimester (but not 2nd to delivery), and that GWG decreased with physical exercise 3 times per week for over 30 min; yet, sub-group analyses by intensity of exercise showed no significant difference. Yin et al. (2014) concluded that beginning PA in early pregnancy may reduce the likelihood of GDM whereas Tombers et al. (2023) reported that the benefit of PA on pain intensity decreased as the duration of the intervention increased. Davenport et al.’s (2018a) meta-regression concluded that to achieve at least a 25% reduction in the odds of developing GDM, pre-eclampsia and hypertension, pregnant women need to accumulate at least 600 MET-min/week of moderate-intensity exercise and that the benefits would be attained when exercise is performed at a frequency of at least 3 days/week or at least 25 min per session. Davenport et al. (2018c) reported that larger volumes of exercise translated into greater reductions in blood glucose (without increasing the risk of hypoglycaemia), but there were no associations between exercise volume, intensity or frequency and the odds of fetal/newborn mortality (Davenport et al., 2019a), or any labour and delivery outcomes (Davenport et al., 2019b). Finally, McDonald et al. (2016) reported no discernible patterns of exercise dose (frequency, duration, intensity) and reductions in GWG.

Davenport et al. (2018a) and He et al. (2023) both reported PA interventions were associated with reduced antenatal depressive symptoms. Davenport et al. (2018a) reported the greatest reduction in supervised interventions, and meta-regression analyses identified the following thresholds to a achieve a moderate effect in reducing prenatal depression: an exercise intensity of 4.0 METs/min (e.g., light walking), exercise duration > 49.9 min per session; exercise frequency > 2.6 times per week; and volume of exercise per week > 644 MET-min/week (e.g., 150 min of moderate intensity exercise). In contrast, He et al. (2023) reported that even low intensity interventions delivered in the 2nd trimester had benefits pre-and postpartum when the duration was over 12 weeks. Liu et al. (2023) were firmer in their conclusions by recommending yoga for antenatal depression with group exercise 3–5 times a week for 30–60 min for 6–10 weeks; although aerobic exercises and single sessions were also effective, intervention effectiveness decreased gradually with extension of time.

Regarding improving PA behaviour, Currie et al. (2013) reported interventions incorporating goals and planning with feedback as effective, but James et al. (2020) concluded that interventions had low impact on PA promotion during pregnancy; with no single technique shown to be impactful. However, Currie et al. concluded that interventions delivered face-to-face were more likely to produce change in PA and James et al. similarly reported that all significant trials included an individual interview.

Seven reviews had inclusion criteria focused upon a specific intervention feature. Beetham et al.’s (2019) review of interventions with vigorous PA reported benefits in length of gestation and prematurity but no influence on infant birthweight. Despite the criterion on PA intensity, the authors concluded that the wide variety in volume, frequency, and duration of interventions limited firm conclusions. In contrast to vigorous PA, Sanabria-Martin et al. (2015) reported that low-to-moderate intensity interventions decreased the risk of GDM, particularly when PA was performed throughout pregnancy and with combined interventions (aerobic, strength, and flexibility exercises). In contrast, PA interventions decreased GWG both when delivered throughout pregnancy and from the 2nd trimester onwards and also with both aerobic-only and combined interventions. The authors found no differences in either outcome when comparing light and moderate intensity interventions. Earlier initiation was also recommended by DiMascio et al. (2016) who found women randomised to an aerobic regimen before 23 weeks had a significantly higher incidence of vaginal delivery, and a significantly lower incidence of caesarean delivery, GDM, and hypertensive disorders; however subgroup analyses of the type and length of exercise session showed no significant difference on health outcomes. Similarly, Bennett et al.’s (2023) review of supervised interventions reported decreased risk of GDM in interventions with increased training volume and starting in the first trimester. Earlier initiation of supervised PA interventions was also recommended by Makaruk et al. (2019) for GDM prevention. In contrast, Poyatos-Leon et al. (2015) sub-analyses reported benefits on delivery outcomes when supervised PA was performed in the second and third trimester. Finally, in group-delivered interventions, Jorge et al (2015) reported significant effects on an array of maternal outcomes but also concluded the lack of consistency across studies prevents conclusions regarding intensity or duration.

Nine reviews examined specific types of PA. Three reviews focused on Pilates and reported improved delivery outcomes (Baradwan et al., 2024) and pain scores (Zenitasari et al., 2025), with longer/more frequent sessions and supervision by certified instructors being associated with improved outcomes (Turan et al., 2025). Chan et al.’s (2019) review of land-based PA interventions concluded that while a compulsory exercise program consistently improved PA, the heterogeneity of studies prevented firm conclusions regarding intervention feature effectiveness. In contrast, Cancela-Carral et al.’s (2022) review of aquatic-based exercise reported positive effects in preventing excessive GWG and reducing mean birth weight differences; with greater benefits observed with a greater volume of exercise. However, there was no difference between aquatic and non-aquatic exercise in regulating newborn birth weight. Similarly, a dose–response was observed for yoga interventions with significant decreases in depression and anxiety observed, where decreases were optimized in studies longer than seven weeks (Sheffield et al., 2016). The evidence was more mixed for reviews focused on aerobic and/or resistance exercise interventions. Zhang et al (2023) reported significantly lower incidences of GDM and hypertension. Perales et al. (2016) reported a strong benefit of combined aerobic and resistance exercise on cardiorespiratory fitness and urinary incontinence, but weak or insufficient evidence for impact on other outcomes, nor aerobic or resistance exercise individually on a wide array of maternal and neonatal outcomes. Guillemette et al. (2018) also focused on aerobic and/or resistance exercise but with the additional criteria of a duration of over 4 weeks and reported that aerobic-only training reduced birth weight, but resistance training only, or combined regimens had non-significant effects. In addition, frequency of exercise did not impact birth weight and while light to moderate intensity interventions reduced birth weight, moderate to vigorous intensity interventions did not.

##### Diet-based interventions (n = 6 reviews)

Across the 6 diet-focused reviews, Gresham et al. (2016) reported that dietary counselling during pregnancy reduced maternal blood pressure, whereas dietary interventions focused on modifying macronutrient intakes during pregnancy reduced the incidence of preterm delivery. Their meta-analysis found no significant difference between the type of dietary intervention (counselling, specific diet vs. combination of counselling and specific diet) on hypertensive disorders, GDM, caesarean delivery, or gestational length. A further review by Gresham et al. (2014) reported that dietary interventions focused on modifying macronutrient intakes improved birth weight outcomes and infant length, with no effect on incidence of macrosomia or prolonged gestation; however, the effects of dietary counselling on these outcomes were not consistent. Hanifi et al. (2023) reported that 4 of 5 studies showed lower prevalence of low-birthweight infants in groups receiving registered dietitian involvement compared to no involvement. However, there was no significant difference in small-for-gestational-age prevalence. Vincze et al. (2019) was the only review to report on associations with GWG and concluded interventions were too heterogeneous to isolate the effective components of interventions, although successful interventions were more likely to include individual in-person sessions with a dietician. Tieu et al. (2017) analysed a wide range of outcomes and yet only found very low-quality evidence that suggests a possible reduction in GDM risk for women receiving dietary advice and low-quality evidence suggests no clear difference for women receiving low-versus moderate/high-GI dietary advice. O’Connor et al. (2025) was the only review to focus on dietary behaviours and reported that the BCTs category ‘Reward and threat’ and ‘Action planning’ were significantly associated with improved dietary quality. Interventions delivered by nutrition/health professionals and that included group sessions via multi-modalities were also found the most effective in improving dietary quality. Inversely interventions that did *not* include the BCTs ‘Discrepancy between current behaviour and goal’ and ‘Feedback on the behaviour’ were also more effective.

##### MHealth interventions (n = 5 reviews)

Across the five reviews focused on MHealth interventions there was evidence of them being effective at reducing GWG (Leonard et al., 2021), improving PA and eating behaviours (Leonard et al., 2021), but also no effect on GWG (Rhodes et al., 2020; Sherifali et al., 2017), and mixed findings on PA behaviour and no impact on GDM (Hussain et al., 2020). While the earliest review stated the quantity and quality of evidence was low and prevented any robust claims on what components were effective (O’Brien et al., 2014), more recent reviews have been firmer in their conclusions by identifying what intervention features have been associated with effectiveness. Interventions using technology with face-to-face sessions, tracking tools, and incorporating devices daily were associated with slightly larger effects in Leonard et al.’s review (particularly for PA and healthy eating behaviours) and the use of multiple components (including text messaging and use of social media) was also supported by Sherifali et al. (2017) and Hussain et al. (2020). Regarding BCT content, Rhodes et al (2020) reported effective interventions averaged over twice as many BCTs from the goals and planning, and feedback and monitoring domains as ineffective interventions; and information-only interventions were ineffective.

#### Smoking interventions (n = 10 reviews)

All smoking intervention reviews reported on smoking behaviour, and 5 reported on health outcomes (e.g. preterm birth, birth weight). Of the reviews that placed limited restrictions on intervention type, Chamberlain et al (2017) was the most comprehensive review (n = 88 trials included) and concluded that psychosocial interventions increased the proportion of women who stop smoking in late pregnancy, with counselling, feedback and incentives appearing to be effective components. The effect of health education, social support, and exercise was unclear. It was unclear whether incorporating the use of self-help manuals or telephone support increased the effectiveness of interventions. Similarly, Su et al. (2014) concluded incentivised interventions had the highest rates of cessation in pregnancy and maintained cessation into the short to medium-term postpartum period (however, once incentives were removed, smoking behaviours relapsed). Of the reviews focusing on intervention content effectiveness, Brown et al. (2019) report finding no clear pattern as to which modes of delivery and timings of intervention contact would be best. However, ‘problem solving’, ‘information about health consequences’, ‘information about social and environmental consequences’, ‘social support’, ‘reduce negative emotions’ and ‘instruction on how to perform a behaviour’ were considered ‘promising BCTs’. In contrast, Acar et al.’s (2022) review of interventions with a specified theoretical basis concluded there were no clear findings about the effectiveness of theory-based interventions, but that interventions based on stages of change may be supportive.

Tahan et al.’s review (2023) explored the effectiveness of specifically nurse or midwife-led counselling (NOMC), digital, and biofeedback interventions, and reported NOMC and digital interventions may assist abstinence rates in late pregnancy and postpartum. In contrast, biofeedback interventions showed little evidence of effectiveness. The authors concluded that NOMC interventions were more effective than nurse home visitation programmes or motivational interviewing, and that counselling effectiveness was increased with increased frequency and when added to other intervention components. In contrast, for digital interventions, short text message, computer and mobile application programmes that include contingency management, or gamified type behavioural interventions appeared to be more effective for promoting abstinence. A review focusing on contingency management interventions also found that contingency management was consistently more effective in increasing the likelihood of abstinence into the postpartum, while psychotherapy alone was not effective (Wilson et al., 2018).

Griffiths et al. (2018), Heminger et al. (2016), and Hussain et al. (2020) all focused on technology supported interventions. Hussain et al. (2020) and Heminger et al. (2016) both reported limited evidence of effectiveness, with studies undermined by low enrolment, engagement, and sample sizes. In contrast, Griffiths et al. (2018) had a wider remit by including digital interventions not necessarily delivered by mobile phone (e.g. website, DVD) and concluded that a higher number of BCTs in each digital intervention led to greater effects. Moderator analyses identified 7 BCTs associated with smoking cessation effectiveness: information about antecedents; action planning; problem solving; goal setting (behaviour); review behaviour goals; social support (unspecified); and pros and cons. Griffiths et al. (2018) concluded that computer-based and text-message interventions were the most effective platforms.

#### Alcohol interventions (n = 6 reviews)

Gomez et al.’s review (2021) placed no restrictions on intervention type. Sub-group analyses showed no difference in the BCTs applied in effective and non-effective interventions. The authors concluded that brief interventions (BIs) and motivational approaches show the most promise to change alcohol-related behaviour in pregnancy, particularly in those drinking more heavily but who do not meet the criteria for dependence. However, they state that while motivational interviewing may be most effective with people who are less motivated and more resistant to change, this may be less relevant for many pregnant women who may already be motivated to change unhealthy behaviours. Minozzi et al.’s (2024) review considered all eligible interventions to be BIs (ranging from a single session of 10–60 min to 5 sessions of 10 min each) and despite concluding that BIs were effective at increasing rates of abstinence, they could not determine the influence of intensity or frequency of psychosocial interventions. Regarding BIs, the number of included studies using this definition varied between single- to multiple-sessions, and there was no clear evidence on the optimal number of sessions. Froschl et al. (2013) and Popova et al., (2023a, 2023b) focused specifically on ‘short-term’ and BIs, respectively, but were flexible regarding defined number of sessions. Despite Froschl et al. (2013) being the only review graded ‘low-quality’, both reviews reached similar conclusions in that while interventions demonstrated effectiveness on alcohol behaviours and birth outcomes, there were comparable improvements observed in the control groups with a discussion of how alcohol screening being commonplace in standard care may have influenced the control arm findings. Both sets of authors stated there was no evidence for the key conditions or factors for the success of interventions, with studies giving little detail regarding the types of interventions and wide variation in duration and the follow-up of interventions. In contrast to these studies, Samawi et al (2021) noted interventions longer in duration with more frequent engagement appeared to be more effective.

Oh et al. (2022) had a more restrictive inclusion criteria of only including digital interventions. Meta-analysis demonstrated interventions decreased the risk of alcohol consumption. Sub-group analysis showed computer/internet-based interventions were an effective platform, but there were too few studies of text messaging to draw a conclusion. The authors concluded that the most effective interventions were those that incorporated both offline home counselling and internet or mobile-based feedback (i.e., “blended” care), suggesting these incurred less social pressure than face-to-face interactions. However, the authors were unable to control for intervention duration, quality, and intensity on the analysis throughout the studies.

## Discussion

This umbrella review aimed to identify what intervention features are associated with the effectiveness across interventions targeting diet/PA, smoking and alcohol behaviours, and identify which intervention features show promise in targeting one behaviour but have yet to be tested in addressing other behaviours. A summary of the findings, methodological observations, and recommendations for future exploration for each behaviour (and across behaviours) is provided in Table 3. The evidence predominantly suggests that diet/PA interventions should begin in the first trimester, run for a longer duration, and entail frequent engagement via a range of modalities in a supervised format (ideally from a specialist provider). Similarly, there was evidence of multi-modal delivery and more frequent engagement being associated with effectiveness for both smoking and alcohol interventions. Smoking intervention effectiveness was consistently associated with incentives and counselling. The least clear insights were for alcohol interventions as while BIs were consistently reported as effective, the frequency, length, and intensity of what defined BIs varied across studies/reviews. Across behaviours, BCTs from the goals and planning and feedback and monitoring domains were routinely associated with effectiveness; yet there was no evidence of theory-based interventions being more effective. ‘Problem solving’ and ‘social support’ were highlighted as effective in specifically smoking interventions.

**Table 3:** Synthesis of research findings

	Intervention features consistently associated with effectiveness	Methodological observations	Insights of potentially effective intervention features/future research
Smoking	Incentives Counselling Individual BCTs of ‘problem solving’ and ‘social support’	All reviews reported on smoking behaviour (e.g. abstinence rates) and not proxy health outcomes alone (e.g. preterm birth)	Explore impact of optimising (e.g. magnitude, frequency) of reward/incentives required Understanding persistence of effects after incentives removed
Alcohol	Brief interventions	Varying definition on what constitutes ‘brief intervention’ Studied substantially less than other behaviours	Individual BCTs of ‘problem solving’ and ‘social support’ show promise
Diet/PA	Commencing in the 1st trimester Run for longer duration (traverse a semester) Delivery by specialist provider	Reviews focusing exclusively on dietary interventions were more commonly focused on neonatal and delivery outcomes compared to PA interventions Reviews with broad intervention inclusion criteria focused on 1 or 2 outcomes; whereas reviews with more specific intervention inclusion criteria examined a wider array of outcomes (2 +)	Individual BCTs of ‘problem solving’ and ‘social support’ show promise Develop a pregnancy-specific taxonomy that helps interventions be classified according to intensity on several intervention features
Across behaviours	Multi-modal delivery (SMS, in-person) rather than single source Frequent engagement via range of modalities (SMS, in-person) BCTs from the ‘goals and planning’ and ‘feedback and monitoring’ domains	Smoking and alcohol interventions focused primarily on measuring behaviours whereas diet/PA interventions primarily focused on proxy health outcomes (e.g. GDM rather than dietary intake)	Controlling for the influence of social support may help understand the mixed findings of the importance of supervision and group settings, where heterogeneity may be due to an inability to differentiate informational and emotional support Paucity of evidence examining multiple behaviour change with only one eligible review focused on more than one behaviour

The underpinning mechanisms why these specific intervention features may be effective may be synergistic. Early pregnancy is a critical period when intentions and behaviours are still being established, and motivation for behaviour change is driven by heightened risk perception and increased receptivity (McBride et al., 2003). From a fetal developmental perspective, earlier intervention maximises the potential to influence exposures during sensitive periods, thereby increasing the likelihood of improving health outcomes (Murray, 2020). The BCTs identified as potentially effective (goal setting, feedback, self-monitoring etc.) are recognised components of Bandura’s social cognitive theory of self-regulation, and supervision in a safe environment (particularly by a specialist provider acting as another BCT of ‘credible source’) may bolster another component of the theory by increasing behaviour self-efficacy (Bandura, 1991). Earlier initiation and longer duration increase cumulative exposure to BCTs and allows greater opportunity for habit formation, changes to be observed, reinforcement, and maintenance before common pregnancy symptoms restrict engagement (e.g. backache, fatigue) (Rockliffe et al., 2021). The use of incentives provides further reinforcement as a form of operant conditioning by providing immediate tangible rewards as a form of feedback (Higgins 2007). Finally, the use of multiple delivery modalities (e.g., face-to-face, digital, telephone) may increase effectiveness by improving accessibility and engagement. Multimodal interventions may accommodate different preferences and contexts and thus increase likelihood to reinforce key messages as repeated exposure to content in varied formats may enhance comprehension and retention, while also supporting sustained engagement (Leonard et al., 2021). However, these explanations would contradict the association of BIs being effective for alcohol reduction where the intervention is inherently brief. A potential explanation is that authors themselves stated that definitions of ‘brief’ were not standardised, and it may be that the increased awareness raising in early pregnancy by a health professional such as a midwife is a sufficient driver of behaviour change, with ‘credible source’ cited as a BCT associated with BI effectiveness (Kaner et al., 2017). However, while the review provides understanding of what features are potentially effective, we cannot assume the underlying mechanisms of how they are effective. For example, while hospital-based and supervised interventions were shown to be more effective in several studies, this may be because women may feel safer knowing there is support nearby or may be because it gives the intervention more credence. Both factors may encourage greater adherence, and it may be these factors having the effect and not the setting nor delivery. Consequently, several intervention features are likely to have a synergistic effect.

Comparison across reviews highlighted differences in the intervention approaches that have been trialled for each behaviour. Testing incentive-based interventions for alcohol is a novel and potentially fruitful avenue given the success of these interventions for a comparable addictive behaviour of smoking and some PA/diet interventions. A potential barrier to extrapolating the effective approach is that smoking status over time can be objectively measured whereas alcohol use is more reliant on self-report, which may become biased if there is an incentive involved. Regarding trialling BCTs, with ‘problem solving’ and ‘social support’ identified as effective in exclusively smoking interventions, these BCTs could be trialled in alcohol and diet/PA interventions. Furthermore, controlling for the influence of social support may help understand the mixed findings of the importance of group settings, where heterogeneity may be due to an inability to differentiate informational and emotional support.

Across reviews there was wide variation in number of reviews for each target behaviour, the outcomes examined, and the variation in intervention features that had been focused upon. However, this heterogeneity provides useful insights into how these different behaviours have each been uniquely examined. With smoking intervention reviews, there was a small number of reviews of a larger number of studies, but it was easier to draw comparisons due to consistency in the outcome (smoking cessation). In contrast, while also targeting the prevention of an addictive behaviour, the evidence for alcohol interventions was far less clear. Partly due to limited studies/reviews, but the lack of standardisation of BIs may explain the contradictory findings of BIs being effective for alcohol but interventions of longer duration being more effective for diet/PA and smoking. In contrast, diet and PA interventions were routinely targeting multiple behaviours and yet those examining exclusively dietary interventions more commonly focused on neonatal and delivery outcomes. It may be that PA interventions may have benefits on these outcomes that were not measured. In addition, smoking and alcohol interventions focused primarily on measuring behaviours whereas diet/PA interventions primarily focused on proxy health outcomes (e.g. GDM rather than dietary intake), again masking potential intervention effects.

This review has several strengths relating to the rigorous methods applied following established methodological protocols and transparent reporting. While it is a standalone review reporting on factors which may have impacted on intervention effectiveness, it builds on the previous two reviews in this series that report on effectiveness of interventions on behavioural and health outcomes, providing a comprehensive overview of existing systematic review evidence base to date, including the patterns across different behaviours and the evidence gaps. The reviews included in the current review were mostly high quality but there are limitations inherent to this methodology. To synthesise findings from across reviews it was necessary to rely on the included review authors’ reporting and conclusions, and authors may have varied in their coding of BCTs and intervention content. One way we tried to address this and be conservative in our conclusions was to report primarily on significant findings rather than on ‘amber’ or ‘red’ findings where the associations are less definitive or a lack of association may be due to Type II error where sub-analyses may have been under-powered. Furthermore, individual systematic reviews may have included primary studies dating back several decades which may limit the applicability of some findings to contemporary contexts, particularly given changes in social norms towards these behaviours in pregnancy and the development of technologies for intervention delivery. However, this arguably strengthens the rationale to conduct umbrella reviews to identify consistent messaging across multiple systematic reviews where inclusion criteria differ. To make future research more focused, inclusion criteria should prioritise more recent interventions.

Overlap of individual studies is a recognised methodological challenge in umbrella reviews, as it can lead to disproportionate weighting of evidence from frequently included primary studies. While formally quantifying overlap (e.g., using corrected covered area metrics) is sometimes possible with a single highly specific topic, the wide range of outcomes, multiple behaviours, and high number of individual studies make this type of analysis beyond the scope of the present study. We agree that overlap may still have influenced the overall conclusions, but the high number of unique studies across reviews helps diminish this. In addition, we focused on synthesising findings narratively at the level of systematic reviews rather than aggregating effect sizes across reviews, thereby reducing the risk of double-counting individual primary studies in any quantitative analyses. However, the nature of this review had unique elements that mitigate these common criticisms of umbrella reviews. The review compared conclusions of reviews for separate behaviours, so the same studies do not appear across reviews. Only one review was included for more than one behaviour and this only included 9 studies (Hussain et al., 2020). Second, even when studies were included in multiple reviews, they were not necessarily extracting the same information, as the authors may have focused on different interventions features, or performed different BCT coding – so the same study may be used to present different information rather than simply being double-counted. These two reasons highlight further why an umbrella approach was needed to summarise both the disparity across individual reviews in reported associations in intervention effectiveness, and to identify what intervention features have yet to be trialled across multiple behaviours.

### Implications and future research

Despite many pregnant women engaging in multiple negative health behaviours (Desmet et al., 2023), only one review was found that examined interventions for multiple behaviours (Hussain et al., 2020). This review was focused on mobile phone-based health interventions in high-income countries for smoking, diet and/or PA and most reviews included studies primarily from high income countries. Thus, the current umbrella review further highlights that there is a paucity of evidence examining multiple behaviour change in pregnancy for the most vulnerable groups from socially deprived environments. Furthermore, given that the challenge of healthcare professionals globally is often addressing multiple behaviours in time-limited appointments, there is a need for process evaluations to gain evidence of which factors are most likely to prompt engagement/adherence; and whether these factors are consistent across behaviours.

Comparison of findings across reviews highlighted potential testing opportunities for interventions for each specific behaviour and whether these translate across behaviours. For smoking interventions, several authors recommended further exploration of the magnitude and frequency of reward/incentives required, and understanding the persistence of effects after incentives are removed. For diet/PA and alcohol interventions, the feature most ambiguous in its relation to effectiveness was intensity. Authors differed considerably in their definition of intensity with definitions ranging from increments within an intervention feature, incremental content, to algorithms that consider a combination of both with other features such as duration and frequency. An area for future research is to develop a pregnancy-specific taxonomy that helps interventions be classified according to intensity on several intervention features. Finally, the current findings provide insight into potentially effective intervention features but is beyond the scope of this review to advise on the practical implications on the extent these intervention features may be feasible to implement across different contexts. Effective interventions reliant on counselling and incentives are both expensive interventions to implement and may not be achievable at scale given the high prevalence of smoking in many low- and middle-income countries (Lange et al., 2018). Realist reviews on a specific intervention feature may be a method to identify the specify contexts when and how interventions can be successfully implemented for specific populations (Wong, 2018).

### Conclusion

The literature reviewed highlighted substantial variation across interventions in the target behaviour, outcomes, modes of delivery, and BCTs utilised. This heterogeneity precludes firm conclusions on how to optimise intervention design, but several features were identified as most likely to be beneficial when designing interventions. Diet/PA interventions should begin in the first trimester, run for a longer duration, and entail frequent engagement via a range of modalities in a supervised format (ideally from a specialist provider). Smoking intervention effectiveness was consistently associated with incentives and counselling. BIs were consistently reported as effective interventions for alcohol use but there was a lack of standardisation in delivery. BCTs from the goals and planning and feedback and monitoring domains were routinely associated with effectiveness. Further research is required to explore intervention features that have shown promise in one behaviour but have yet to be tested for other behaviours.

## Supplementary Information

Below is the link to the electronic supplementary material.

Supplementary Material 1

Supplementary Material 2

Supplementary Material 3

Supplementary Material 4

Supplementary Material 5

Supplementary Material 6

Supplementary Material 7

## Data Availability

All data are reported within the manuscript and supplementary materials.
