# Supplementary material for: Features of smoking cessation, alcohol reduction, and diet and physical activity interventions associated with changing behaviours during pregnancy: a systematic review of reviews
Source: J Behav Med. 2026 Aug 6;49(6):985–1001. doi: 10.1007/s10865-026-00702-1 (PMC13748204; doi:10.1007/s10865-026-00702-1)
Supplement: Supplementary file 1 [file 10865_2026_702_MOESM1_ESM.docx]

**Supplement 1: Search strategy**

**All searches conducted 16-6-2025**

| **Ovid Medline** | |
| --- | --- |
|  |  |
|  | **Search terms** |
| S1 | (MH "Pregnant Women") |
| S2 | (MH "Pregnancy+") |
| S3 | TI pregnan$ OR AB pregnan$ |
| S4 | TI gravid$ OR AB gravid$ |
| S5 | MH ("Gestational Age") |
| S6 | TI gestation$ OR AB gestation$ |
| S7 | (MH "Prenatal Care") |
| S8 | TI antenatal$ OR AB antenatal$ |
| S9 | TI perinatal$ OR AB perinatal$ |
| S10 | TI prenatal$ OR AB prenatal$ |
| S11 | TI maternal OR AB maternal |
| S12 | TI maternity OR AB maternity |
| S13 | (MH "Mothers") |
| S14 | TI mother$ OR AB mother$ |
| S15 | TI child bearing OR AB child bearing |
| S16 | TI antepartum OR AB antepartum |
| S17 | TI interpregnancy OR AB interpregnancy |
| S18 | TI intrapartum OR AB intrapartum |
| S19 | TI natal OR AB natal |
| S20 | TI parity OR AB parity |
| S21 | TI parous OR AB parous |
| S22 | TI nulligravid$ OR AB nulligravid$ |
| S23 | TI multigravid$ OR AB multigravid$ |
| S24 | TI primigravid$ OR AB primigravid$ |
| S25 | TI pregnant woman OR AB pregnant woman |
| S26 | TI pregnant women OR AB pregnant women |
| S27 | TI pregnant female$ OR AB pregnant female$ |
| S28 | TI pregnant human$ OR AB pregnant human$ |
| S29 | TI pregnant girl$ OR AB pregnant girl$ |
| S30 | TI reproductive woman OR AB reproductive woman |
| S31 | TI reproductive women OR AB reproductive women |
| S32 | TI reproductive female$ OR AB reproductive female$ |
| S33 | TI reproductive human$ OR AB reproductive human$ |
| S34 | TI reproductive girl$ OR AB reproductive girl$ |
| S35 | S1 OR S2 OR S3 OR S4 OR S5 OR S6 OR S7 OR S8 OR S9 OR S10 OR S11 OR S12 OR S13 OR S14 OR S15 OR S16 OR S17 OR S18 OR S19 OR S20 OR S21 OR S22 OR S23 OR S24 OR S25 OR S26 OR S27 OR S28 OR S29 OR S30 OR S31 OR S32 OR S33 OR S34 |
| S36 | (MH "Weight Gain") |
| S37 | TI weight OR AB weight |
| S38 | (MH "Overweight+") |
| S39 | TI overweight OR AB overweight |
| S40 | TI gestational weight gain OR AB gestational weight gain |
| S41 | TI GWG OR AB GWG |
| S42 | (MH "Obesity") |
| S43 | TI obes$ OR AB obes$ |
| S44 | (MH "Body Mass Index") |
| S45 | TI body mass index OR AB body mass index |
| S46 | TI BMI OR AB BMI |
| S47 | TI fatness OR AB fatness |
| S48 | TI body fat OR AB body fat |
| S49 | TI size OR AB size |
| S50 | (MH "Pregnancy Complications+") |
| S51 | (MH "Pregnancy Outcome+") |
| S52 | (MH "Smoking") |
| S53 | TI smoking OR AB smoking |
| S54 | TI smok$ OR AB smok$ |
| S55 | TI cigar$ OR AB cigar$ |
| S56 | TI pipe OR AB pipe |
| S57 | TI pipes OR AB pipes |
| S58 | TI nicotine OR AB nicotine |
| S59 | (MH "Alcohol Drinking+") |
| S60 | (MH "Alcoholic Intoxication") |
| S61 | (MH "Alcoholics") |
| S62 | (MH "Alcoholism") |
| S63 | (MH "Alcoholic Beverages+") |
| S64 | TI alcohol OR AB alcohol |
| S65 | TI binge$ OR AB binge$ |
| S66 | TI drunk$ OR AB drunk$ |
| S67 | TI liquor OR AB liquor |
| S68 | TI intoxication OR AB intoxication |
| S69 | S36 OR S37 OR S38 OR S39 OR S40 OR S41 OR S42 OR S43 OR S44 OR S45 OR S46 OR S47 OR S48 OR S49 OR S50 OR S51 OR S52 OR S53 OR S54 OR S55 OR S56 OR S57 OR S58 OR S59 OR S60 OR S61 OR S62 OR S63 OR S64 OR S65 OR S66 OR S67 OR S68 |
| S70 | (MH "Diet+") |
| S71 | (MH "Diet, Reducing") |
| S72 | TI diet$ OR AB diet$ |
| S73 | (MH "Weight Reduction Programs") |
| S74 | TI weight N5 intervention$ OR AB weight N5 intervention$ |
| S75 | (MH "Caloric Restriction") |
| S76 | TI caloric restriction$ OR AB caloric restriction$ |
| S77 | (MH "Feeding Behavior+") |
| S78 | TI feeding OR AB feeding |
| S79 | (MH "Food Habits+") |
| S80 | TI food$ OR AB food$ |
| S81 | TI eating OR AB eating |
| S82 | TI grazing OR AB grazing |
| S83 | TI snack$ OR AB snack$ |
| S84 | TI healthy eating OR AB healthy eating |
| S85 | (MH "Exercise+") |
| S86 | TI exercis$ OR AB exercis$ |
| S87 | TI physical activit$ OR AB physical activit$ |
| S88 | (MH "Sedentary Lifestyle") |
| S89 | (MH "Life Style+") |
| S90 | TI lifestyle$ OR AB lifestyle$ |
| S91 | (MH "Tobacco Use Cessation+") |
| S92 | (MH "Tobacco Use Cessation Products+") |
| S93 | TI nicotine replacement OR AB nicotine replacement |
| S94 | TI NRT OR AB NRT |
| S95 | TI habit$ OR AB habit$ |
| S96 | TI change$ OR AB change$ |
| S97 | TI modification OR AB modification |
| S98 | TI program$ OR AB program$ |
| S99 | TI assist$ OR AB assist$ |
| S100 | TI behavio?r OR AB behavio?r |
| S101 | TI avoid$ OR AB avoid$ |
| S102 | TI prevent$ OR AB prevent$ |
| S103 | TI maintenance OR AB maintenance |
| S104 | TI management OR AB management |
| S105 | TI gain OR AB gain |
| S106 | TI loss OR AB loss |
| S107 | TI lose OR AB lose |
| S108 | TI control OR AB control |
| S109 | TI reduction OR AB reduction |
| S110 | TI behavio?r OR AB behavio?r |
| S111 | TI control OR AB control |
| S112 | TI cessation OR AB cessation |
| S113 | TI therap$ OR AB therap$ |
| S114 | TI relapse$ OR AB relapse$ |
| S115 | TI abstain OR AB abstain |
| S116 | TI abstinence OR AB abstinence |
| S117 | TI intervention$ OR AB intervention$ |
| S118 | (MH "Alcohol Abstinence") |
| S119 | S70 OR S71 OR S72 OR S73 OR S74 OR S75 OR S76 OR S77 OR S78 OR S79 OR S80 OR S81 OR S82 OR S83 OR S84 OR S85 OR S86 OR S87 OR S88 OR S89 OR S90 OR S91 OR S92 OR S93 OR S94 OR S95 OR S96 OR S97 OR S98 OR S99 OR S100 OR S101 OR S102 OR S103 OR S104 OR S105 OR S106 OR S107 OR S108 OR S109 OR S110 OR S111 OR S112 OR S113 OR S114 OR S115 OR S116 OR S117 OR S118 |
| S120 | PT Review |
| S121 | TI systematic review$ OR AB systematic review$ |
| S122 | (MH "Review Literature as Topic+") |
| S123 | TI review$ OR AB review$ |
| S124 | (PT "Meta-Analysis") |
| S125 | (MH "Meta-Analysis as Topic") |
| S126 | TI meta analysis OR AB meta analysis |
| S127 | TI meta analyses OR AB meta analyses |
| S128 | TI metaanalysis OR AB metaanalysis |
| S129 | TI metaanalyses OR AB metaanalyses |
| S130 | TI overview$ OR AB overview$ |
| S131 | TI comprehensive$ OR AB comprehensive$ |
| S132 | TI systematic OR AB systematic |
| S133 | TI research synthesis OR AB research synthesis |
| S134 | TI research syntheses OR AB research syntheses |
| S135 | TI evidence summary OR AB evidence summary |
| S136 | S120 OR S121 OR S122 OR S123 OR S124 OR S125 OR S126 OR S127 OR S128 OR S129 OR S130 OR S131 OR S132 OR S133 OR S134 OR S135 |
| S137 | S35 AND S69 AND S119 AND S136 |
| S138 | S35 AND S69 AND S119 AND S136 (2019 to date) |
| S139 | S35 AND S69 AND S119 AND S136 (English only) |

| **EBSCO CINAHL** | | |
| --- | --- | --- |
|  |  |  |
|  | **Search terms** |  |
| S1 | (MH "Expectant Mothers") |  |
| S2 | (MH "Pregnancy+") |  |
| S3 | TI ( pregnancy or pregnant ) OR AB ( pregnancy or pregnant ) |  |
| S4 | TI gravid$ OR AB gravid$ |  |
| S5 | (MH "Gestational Age") |  |
| S6 | TI gestation$ OR AB gestation$ |  |
| S7 | (MH "Prenatal Care") |  |
| S8 | TI antenatal$ OR AB antenatal$ |  |
| S9 | TI perinatal$ OR AB perinatal$ |  |
| S10 | TI prenatal$ OR AB prenatal$ |  |
| S11 | TI maternal OR AB maternal |  |
| S12 | TI maternity OR AB maternity |  |
| S13 | (MH "Mothers+") | |
| S14 | TI mother$ OR AB mother$ | |
| S15 | TI child bearing OR AB child bearing | |
| S16 | TI antepartum OR AB antepartum | |
| S17 | TI interpregnancy OR AB interpregnancy | |
| S18 | TI ( TI intrapartum OR AB intrapartum ) OR AB ( TI intrapartum OR AB intrapartum ) | |
| S19 | TI natal OR AB natal | |
| S20 | TI parity OR AB parity | |
| S21 | TI parous OR AB parous | |
| S22 | TI nulligravid$ OR AB nulligravid$ | |
| S23 | TI multigravid$ OR AB multigravid$ | |
| S24 | TI primigravid$ OR AB primigravid$ | |
| S25 | TI pregnant woman OR AB pregnant woman | |
| S26 | TI pregnant women OR AB pregnant women | |
| S27 | TI pregnant female$ OR AB pregnant female$ | |
| S28 | TI pregnant human$ OR AB pregnant human$ | |
| S29 | TI pregnant human$ OR AB pregnant human$ | |
| S30 | TI reproductive woman OR AB reproductive woman | |
| S31 | TI reproductive women OR AB reproductive women | |
| S32 | TI reproductive female$ OR AB reproductive female$ | |
| S33 | TI reproductive human$ OR AB reproductive human$ | |
| S34 | TI reproductive girl$ OR AB reproductive girl$ | |
| S35 | S1 OR S2 OR S3 OR S4 OR S5 OR S6 OR S7 OR S8 OR S9 OR S10 OR S11 OR S12 OR S13 OR S14 OR S15 OR S16 OR S17 OR S18 OR S19 OR S20 OR S21 OR S22 OR S23 OR S24 OR S25 OR S26 OR S27 OR S28 OR S29 OR S30 OR S31 OR S32 OR S33 OR S34 | |
| S36 | (MH "Weight Gain+") | |
| S37 | TI weight OR AB weight | |
| S38 | TI overweight OR AB overweight | |
| S39 | TI gestational weight gain OR AB gestational weight gain | |
| S40 | TI GWG OR AB GWG | |
| S41 | (MH "Obesity+") | |
| S42 | TI obes$ OR AB obes$ | |
| S43 | (MH "Body Mass Index") | |
| S44 | TI body mass index OR AB body mass index | |
| S45 | TI BMI OR AB BMI | |
| S46 | TI fatness OR AB fatness | |
| S47 | TI body fat OR AB body fat | |
| S48 | TI size OR AB size | |
| S49 | (MH "Pregnancy Complications+") | |
| S50 | (MH "Pregnancy Outcomes") | |
| S51 | (MH "Smoking+") | |
| S52 | TI smoking OR AB smoking | |
| S53 | TI smoke$ OR AB smoke$ | |
| S54 | TI cigarette OR AB cigarettes | |
| S55 | TI cigar OR AB cigars | |
| S56 | TI pipe OR AB pipe | |
| S57 | TI pipes OR AB pipes | |
| S58 | TI nicotine OR AB nicotine | |
| S59 | (MH "Alcohol Drinking+") | |
| S60 | (MH "Alcoholism") | |
| S61 | (MH "Alcoholic Beverages+") | |
| S62 | (MH "Alcoholic Intoxication+") | |
| S63 | TI alcohol OR AB alcohol | |
| S64 | TI binge$ OR AB binge$ | |
| S65 | TI drunk$ OR AB drunk$ | |
| S66 | TI liquor OR AB liquor | |
| S67 | TI intoxication OR AB intoxication | |
| S68 | S36 OR S37 OR S38 OR S39 OR S40 OR S41 OR S42 OR S43 OR S44 OR S45 OR S46 OR S47 OR S48 OR S49 OR S50 OR S51 OR S52 OR S53 OR S54 OR S55 OR S56 OR S57 OR S58 OR S59 OR S60 OR S61 OR S62 OR S63 OR S64 OR S65 OR S66 OR S67 | |
| S69 | (MH "Diet+") | |
| S70 | (MH "Diet, Reducing") | |
| S71 | TI diet OR AB diet | |
| S72 | TI diets OR AB diets | |
| S73 | (MH "Weight Reduction Programs") | |
| S74 | TI weight N5 intervention$ OR AB weight N5 intervention$ | |
| S75 | TI caloric restriction$ OR AB caloric restriction$ | |
| S76 | (MH "Eating Behavior+") | |
| S77 | TI feeding OR AB feeding | |
| S78 | (MH "Food Habits") | |
| S79 | TI food$ OR AB food$ | |
| S80 | TI eating OR AB eating | |
| S81 | TI grazing OR AB grazing | |
| S82 | TI snack$ OR AB snack$ | |
| S83 | TI healthy eating OR AB healthy eating | |
| S84 | (MH "Exercise+") | |
| S85 | TI exercise OR AB exercise | |
| S86 | (MH "Physical Activity") | |
| S87 | TI physical activity OR AB physical activity | |
| S88 | (MH "Life Style, Sedentary") | |
| S89 | (MH "Life Style+") | |
| S90 | (MH "Smoking Cessation") | |
| S91 | (MH "Smoking Cessation Programs") | |
| S92 | (MH "Tobacco Use Cessation Products+") | |
| S93 | TI nicotine replacement OR AB nicotine replacement | |
| S94 | TI NRT OR AB NRT | |
| S95 | TI habit$ OR AB habit$ | |
| S96 | TI change$ OR AB change$ | |
| S97 | TI modification OR AB modification | |
| S98 | TI program$ OR AB program$ | |
| S99 | TI assist$ OR AB assist$ | |
| S100 | TI behavio?r OR AB behavio?r | |
| S101 | TI avoid$ OR AB avoid$ | |
| S102 | TI prevent$ OR AB prevent$ | |
| S103 | TI maintenance OR AB maintenance | |
| S104 | TI management OR AB management | |
| S105 | TI gain OR AB gain | |
| S106 | TI loss OR AB loss | |
| S107 | TI lose OR AB lose | |
| S108 | TI control OR AB control | |
| S109 | TI reduction OR AB reduction | |
| S110 | TI behavio?r OR AB behavio?r | |
| S111 | TI control OR AB control | |
| S112 | TI cessation OR AB cessation | |
| S113 | TI therapy OR AB therapy | |
| S114 | TI therapies OR AB therapies | |
| S115 | TI relapse$ OR AB relapse$ | |
| S116 | TI abstain OR AB abstain | |
| S117 | TI abstinence OR AB abstinence | |
| S118 | TI intervention$ OR AB intervention$ | |
| S119 | S69 OR S70 OR S71 OR S72 OR S73 OR S74 OR S75 OR S76 OR S77 OR S78 OR S79 OR S80 OR S81 OR S82 OR S83 OR S84 OR S85 OR S86 OR S87 OR S88 OR S89 OR S90 OR S91 OR S92 OR S93 OR S94 OR S95 OR S96 OR S97 OR S98 OR S99 OR S100 OR S101 OR S102 OR S103 OR S104 OR S105 OR S106 OR S107 OR S108 OR S109 OR S110 OR S111 OR S112 OR S113 OR S114 OR S115 OR S116 OR S117 OR S118 | |
| S120 | PT Review | |
| S121 | (MH "Systematic Review") | |
| S122 | TI systematic review$ OR AB systematic review$ | |
| S123 | (MH "Literature Review+") | |
| S124 | TI review$ OR AB review$ | |
| S125 | PT meta analysis | |
| S126 | (MH "Meta Analysis") | |
| S127 | TI meta analysis OR AB meta analysis | |
| S128 | TI meta analyses OR AB meta analyses | |
| S129 | TI metaanalysis OR AB metaanalysis | |
| S130 | TI metaanalyses OR AB metaanalyses | |
| S131 | TI overview$ OR AB overview$ | |
| S132 | TI comprehensive$ OR AB comprehensive$ | |
| S133 | TI systematic OR AB systematic | |
| S134 | TI research synthesis OR AB research synthesis | |
| S135 | TI research syntheses OR AB research syntheses | |
| S136 | TI evidence summary OR AB evidence summary | |
| S137 | S120 OR S121 OR S122 OR S123 OR S124 OR S125 OR S126 OR S127 OR S128 OR S129 OR S130 OR S131 OR S132 OR S133 OR S134 OR S135 OR S136 | |
| S138 | S35 AND S68 AND S119 AND S137 | |
| S139 | S35 AND S68 AND S119 AND S137 (2019 to date) | |
| S140 | S35 AND S68 AND S119 AND S137 (English only) | |

**Epistemonikos**

**Search terms**

(advanced_title_en:(pregnan* OR gravid* OR gestation* OR antenatal* OR perinatal* OR prenatal* OR maternal OR mother* OR child bearing OR antepartum OR interpregnancy OR intrapartum OR natal OR parity OR parous OR nulligravid* OR multigravid* OR primigravid* OR reproductive) OR advanced_abstract_en:(pregnan* OR gravid* OR gestation* OR antenatal* OR perinatal* OR prenatal* OR maternal OR mother* OR child bearing OR antepartum OR interpregnancy OR intrapartum OR natal OR parity OR parous OR nulligravid* OR multigravid* OR primigravid* OR reproductive)) AND (advanced_title_en:(weight OR overweight OR gestational weight gain OR GWG OR obes* OR body mass index OR BMI OR fatness OR body fat OR size OR complication* OR outcome* OR smoking OR smok* OR cigar* OR pipe OR pipes OR nicotine OR alcohol* OR binge* OR drunk* OR liquor OR intoxication) OR advanced_abstract_en:(weight OR overweight OR gestational weight gain OR GWG OR obes* OR body mass index OR BMI OR fatness OR body fat OR size OR complication* OR outcome* OR smoking OR smok* OR cigar* OR pipe OR pipes OR nicotine OR alcohol* OR binge* OR drunk* OR liquor OR intoxication)) AND (advanced_title_en:(diet OR weight reduction OR weight intervention* OR caloric restriction* OR feeding OR food OR eating OR grazing OR snack* OR healthy eating OR exercise* OR physical activit* OR lifestyle OR cessation OR nicotine replacement OR NRT OR habit* OR change* OR modification OR program* OR assist* OR behavio* OR avoid* OR prevent* OR maintenance OR management OR gain OR loss OR lose OR control OR reduction OR therap* OR relapse* OR abstain OR abstinence OR intervention*) OR advanced_abstract_en:(diet OR weight reduction OR weight intervention* OR caloric restriction* OR feeding OR food OR eating OR grazing OR snack* OR healthy eating OR exercise* OR physical activit* OR lifestyle OR cessation OR nicotine replacement OR NRT OR habit* OR change* OR modification OR program* OR assist* OR behavio* OR avoid* OR prevent* OR maintenance OR management OR gain OR loss OR lose OR control OR reduction OR therap* OR relapse* OR abstain OR abstinence OR intervention*)) AND (advanced_title_en:(review* OR meta* OR systematic* OR overview* OR comprehensive* OR synthes* OR summar*) OR advanced_abstract_en:(review* OR meta* OR systematic* OR overview* OR comprehensive* OR synthes* OR summar*)) [Filters: protocol=no, min_year=2019, max_year=2023]

**ProQuest ASSIA**

**Search terms**

(((abstract(lose) OR abstract(control) OR abstract(reduction) OR abstract(therap*) OR abstract(relapse*) OR abstract(abstain) OR abstract(abstinence) AND pd(>20181231)) OR (abstract(modification) OR abstract(program*) OR abstract(assist*) OR abstract(behavio*) OR abstract(avoid*) OR abstract(prevent*) OR abstract(maintenance) OR abstract(management) OR abstract(gain) OR abstract(loss) AND pd(>20181231)) OR (abstract(snack*) OR abstract(healthy eating) OR abstract(exercise) OR abstract(physical activit*) OR abstract(lifestyle) OR abstract(cessation) OR abstract(nicotine replacement) OR abstract(NRT) OR abstract(habit*) OR abstract(change*) AND pd(>20181231)) OR (abstract(diet) OR abstract(weight reduction) OR abstract(weight intervention*) OR abstract(caloric restriction*) OR abstract(feeding behavio*) OR abstract(feeding) OR abstract(food habit*) OR abstract(food*) OR abstract(eating) OR abstract(grazing) AND pd(>20181231))) OR ((title(lose) OR title(control) OR title(reduction) OR title(therap*) OR title(relapse*) OR title(abstain) OR title(abstinence) AND pd(>20181231)) OR (title(modification) OR title(program*) OR title(assist*) OR title(behavio*) OR title(avoid*) OR title(prevent*) OR title(maintenance) OR title(management) OR title(gain) OR title(loss) AND pd(>20181231)) OR (title(snack*) OR title(healthy eating) OR title(exercise) OR title(physical activit*) OR title(lifestyle) OR title(cessation) OR title(nicotine replacement) OR title(NRT) OR title(habit*) OR title(change*) AND pd(>20181231)) OR (title(diet) OR title(weight reduction) OR title(weight intervention*) OR title(caloric restriction*) OR title(feeding behavio*) OR title(feeding) OR title(food habit*) OR title(food*) OR title(eating) OR title(grazing) AND pd(>20181231))) OR ((SU.EXACT("Nicotine gum") OR SU.EXACT("Nicotine patches") OR SU.EXACT("Abstinence") AND pd(>20181231)) OR (SU.EXACT("Diet") OR SU.EXACT("High fat diet") OR SU.EXACT("Low fat diet") OR SU.EXACT("Omnivorous") OR SU.EXACT("Veganism") OR SU.EXACT("Vegetarianism") OR SU.EXACT("Caloric intake") OR SU.EXACT("Feeding patterns") OR SU.EXACT("Food habits") OR SU.EXACT("Caloric intake") AND pd(>20181231)) OR (SU.EXACT("Structured exercise") OR SU.EXACT("Tai Chi") OR SU.EXACT("Water exercise") OR SU.EXACT("Weight training") OR SU.EXACT("Weightlifting") OR SU.EXACT("Yoga") OR SU.EXACT("Health Promoting Lifestyle Profile") OR SU.EXACT("Lifestyle") OR SU.EXACT("Cessation") OR SU.EXACT("Nicotine replacement therapy") AND pd(>20181231)) OR (SU.EXACT("Feeding patterns") OR SU.EXACT("Food habits") OR SU.EXACT("Eating behaviour") OR SU.EXACT("Aerobic exercise") OR SU.EXACT("Dance exercise") OR SU.EXACT("Exercise") OR SU.EXACT("Fitness training") OR SU.EXACT("Hatha yoga") OR SU.EXACT("Pelvic floor exercise") OR SU.EXACT("Sahaja Yoga") AND pd(>20181231)))) AND (((abstract(drunk*) OR abstract(liquor) OR abstract(intoxication) AND pd(>20181231)) OR (abstract(pregnancy complication*) OR abstract(pregnancy outcome*) OR abstract(smoking) OR abstract(smok*) OR abstract(cigar*) OR abstract(pipe) OR abstract(pipes) OR abstract(nicotine) OR abstract(alcohol) OR abstract(binge*) AND pd(>20181231)) OR (abstract(weight) OR abstract(overweight) OR abstract(gestational weight gain) OR abstract(GWG) OR abstract(obes*) OR abstract(body mass index) OR abstract(BMI) OR abstract(fatness) OR abstract(body fat) OR abstract(size) AND pd(>20181231))) OR ((title(weight) OR title(overweight) OR title(gestational weight gain) OR title(GWG) OR title(obes*) OR title(body mass index) OR title(BMI) OR title(fatness) OR title(body fat) OR title(size) AND pd(>20181231)) OR (title(pregnancy complication*) OR title(pregnancy outcome*) OR title(smoking) OR title(smok*) OR title(cigar*) OR title(pipe) OR title(pipes) OR title(nicotine) OR title(alcohol) OR title(binge*) AND pd(>20181231)) OR (title(drunk*) OR title(liquor) OR title(intoxication) AND pd(>20181231))) OR ((SU.EXACT("Nicotine") OR SU.EXACT("Alcohol dependence") OR SU.EXACT("Alcohol intoxication") OR SU.EXACT("Alcoholics") AND pd(>20181231)) OR (SU.EXACT("Body Mass Index") OR SU.EXACT("Body Mass Index") OR SU.EXACT("Weight gain") OR SU.EXACT("Obesity") OR SU.EXACT("Heavy smoking") OR SU.EXACT("Moderate smoking") OR SU.EXACT("Occasional smoking") OR SU.EXACT("Passive smoking") OR SU.EXACT("Smoking") OR SU.EXACT("Tobacco smoke") AND pd(>20181231)))) AND ((SU.EXACT("Homeless pregnant women") OR SU.EXACT("Low income pregnant women") OR SU.EXACT("Older pregnant women") OR SU.EXACT("Poor pregnant women") OR SU.EXACT("Pregnant women") OR SU.EXACT("Single pregnant women") OR SU.EXACT("mothers") OR SU.EXACT("pregnant women") OR SU.EXACT("gestational age") OR SU.EXACT("prenatal care") AND pd(>20181231)) OR ((title(antepartum) OR title(interpregnancy) OR title(intrapartum) OR title(natal) OR title(parity) OR title(parous) OR title(nulligravid*) OR title(multigravid*) OR title(primigravid*) OR title(reproductive) AND pd(>20181231)) OR (title(pregnan*) OR title(gravid*) OR title(gestation*) OR title(antenatal*) OR title(perinatal*) OR title(prenatal*) OR title(maternal) OR title(maternity) OR title(mother*) OR title(child bearing) AND pd(>20181231))) OR ((abstract(antepartum) OR abstract(interpregnancy) OR abstract(intrapartum) OR abstract(natal) OR abstract(parity) OR abstract(parous) OR abstract(nulligravid*) OR abstract(multigravid*) OR abstract(primigravid*) OR abstract(reproductive) AND pd(>20181231)) OR (abstract(pregnan*) OR abstract(gravid*) OR abstract(gestation*) OR abstract(antenatal*) OR abstract(perinatal*) OR abstract(prenatal*) OR abstract(maternal) OR abstract(maternity) OR abstract(mother*) OR abstract(child bearing) AND pd(>20181231)))) AND (SU.EXACT("Systematic reviews") OR title(review*) OR title(meta*) OR abstract(review*) OR abstract(meta*) AND pd(>20181231)) AND pd(>20181231)

**LILACS**

**Search terms**

(pregnan$ OR gravid$ OR gestation$ OR antenatal$ OR perinatal$ OR prenatal$ OR maternal OR mother$ OR child bearing OR antepartum OR interpregnancy OR intrapartum OR natal OR parity OR parous OR nulligravid$ OR multigravid$ OR primigravid$ OR reproductive) [Title words] AND (weight OR overweight OR gestational weight gain OR GWG OR obes$ OR body mass index OR BMI OR fatness OR body fat OR size OR complication$ OR outcome$ OR smoking OR smok$ OR cigar$ OR pipe OR pipes OR nicotine OR alcohol$ OR binge$ OR drunk$ OR liquor OR intoxication) [Title words] AND (diet$ OR weight reduction OR weight intervention$ OR caloric restriction$ OR feeding OR food OR eating OR grazing OR snack$ OR healthy eating OR exercise$ OR physical activit$ OR lifestyle OR cessation OR nicotine replacement OR NRT OR habit$ OR change$ OR modification OR program$ OR assist$ OR behavio$ OR avoid$ OR prevent$ OR maintenance OR management OR gain OR loss OR lose OR control OR reduction OR therap$ OR relapse$ OR abstain OR abstinence OR intervention$) [Title words]

**Social Care Online**

**Search terms**

pregnancy obesity OR pregnancy smoking OR pregnancy alcohol OR pregnancy alcoholic OR pregnancy alcoholism OR cessation abstinence

| **Ovid HMIC** | |
| --- | --- |
|  |  |
|  |  |
| **Search string** | **Search terms** |
| 1 | exp Pregnant women/ |
| 2 | exp Pregnancy/ |
| 3 | exp Maternal health/ |
| 4 | pregnan*.tw. |
| 5 | gravid*.tw. |
| 6 | exp Gestational age/ |
| 7 | gestation*.tw. |
| 8 | exp Antenatal care/ |
| 9 | antenatal.tw. |
| 10 | prenatal.tw. |
| 11 | exp Perinatal care/ |
| 12 | perinatal.tw. |
| 13 | maternal.tw. |
| 14 | exp Maternity care/ |
| 15 | maternity.tw. |
| 16 | exp Mothers/ |
| 17 | mother*.tw. |
| 18 | child bearing.tw. |
| 19 | antepartum*.tw. |
| 20 | interpregnancy.tw. |
| 21 | intrapartum*.tw. |
| 22 | natal.tw. |
| 23 | parity.tw. |
| 24 | parous.tw. |
| 25 | nulligravid*.tw. |
| 26 | multigravid*.tw. |
| 27 | primigravid*.tw. |
| 28 | reproductive.tw. |
| 29 | 1 or 2 or 3 or 4 or 5 or 6 or 7 or 8 or 9 or 10 or 11 or 12 or 13 or 14 or 15 or 16 or 17 or 18 or 19 or 20 or 21 or 22 or 23 or 24 or 25 or 26 or 27 or 28 |
| 30 | exp Weight gain/ |
| 31 | exp Weight/ |
| 32 | weight.tw. |
| 33 | overweight.tw. |
| 34 | gestational weight gain.tw. |
| 35 | GWG.tw. |
| 36 | exp Obesity/ |
| 37 | obes*.tw. |
| 38 | exp body mass index/ |
| 39 | body mass index.tw. |
| 40 | BMI.tw. |
| 41 | fatness.tw. |
| 42 | body fat.tw. |
| 43 | size.tw. |
| 44 | exp Pregnancy complications/ |
| 45 | exp Pregnancy outcome/ |
| 46 | exp Smoking/ |
| 47 | smoking.tw. |
| 48 | smok*.tw. |
| 49 | cigar*.tw. |
| 50 | pipe.tw. |
| 51 | pipes.tw. |
| 52 | exp Nicotine/ |
| 53 | nicotine.tw. |
| 54 | exp Alcohol/ |
| 55 | exp Alcohol consumption/ |
| 56 | alcohol drinking.tw. |
| 57 | exp Drunkenness/ |
| 58 | intoxication.tw. |
| 59 | exp Alcoholism/ |
| 60 | alcohol*.tw. |
| 61 | binge*.tw. |
| 62 | drunk*.tw. |
| 63 | liquor.tw. |
| 64 | 30 or 31 or 32 or 33 or 34 or 35 or 36 or 37 or 38 or 39 or 40 or 41 or 42 or 43 or 44 or 45 or 46 or 47 or 48 or 49 or 50 or 51 or 52 or 53 or 54 or 55 or 56 or 57 or 58 or 59 or 60 or 61 or 62 or 63 |
| 65 | exp Diet/ |
| 66 | exp Diet planning/ |
| 67 | diet*.tw. |
| 68 | weight reduction program*.tw. |
| 69 | weight intervention*.tw. |
| 70 | caloric restriction*.tw. |
| 71 | exp Feeding behaviour/ |
| 72 | feeding.tw. |
| 73 | food habit*.tw. |
| 74 | food*.tw. |
| 75 | exp Healthy eating/ |
| 76 | healthy eating.tw. |
| 77 | exp exercise/ |
| 78 | exercis*.tw. |
| 79 | exp physical activity/ |
| 80 | physical activit*.tw. |
| 81 | exp lifestyle/ |
| 82 | lifestyle*.tw. |
| 83 | exp Smoking cessation/ |
| 84 | cessation.tw. |
| 85 | exp Nicotine replacement therapy/ |
| 86 | NRT.tw. |
| 87 | exp Nicotine chewing gum/ |
| 88 | exp Nicotine patches/ |
| 89 | habit*.tw. |
| 90 | change*.tw. |
| 91 | modification*.tw. |
| 92 | program*.tw. |
| 93 | assist*.tw. |
| 94 | behavio?r.tw. |
| 95 | avoid*.tw. |
| 96 | prevent*.tw. |
| 97 | maintenance.tw. |
| 98 | management.tw. |
| 99 | gain.tw. |
| 100 | loss.tw. |
| 101 | lose.tw. |
| 102 | control.tw. |
| 103 | reduction.tw. |
| 104 | therap*.tw. |
| 105 | relapse*.tw. |
| 106 | exp Alcoholism treatment/ |
| 107 | exp Alcoholism control/ |
| 108 | abstain.tw. |
| 109 | abstinence.tw. |
| 110 | intervention*.tw. |
| 111 | 65 or 66 or 67 or 68 or 69 or 70 or 71 or 72 or 73 or 74 or 75 or 76 or 77 or 78 or 79 or 80 or 81 or 82 or 83 or 84 or 85 or 86 or 87 or 88 or 89 or 90 or 91 or 92 or 93 or 94 or 95 or 96 or 97 or 98 or 99 or 100 or 101 or 102 or 103 or 104 or 105 or 106 or 107 or 108 or 109 or 110 |
| 112 | exp Systematic reviews/ |
| 113 | systematic review*.tw. |
| 114 | review*.tw. |
| 115 | exp Literature reviews/ |
| 116 | exp Meta analysis/ |
| 117 | meta analysis.tw. |
| 118 | meta analyses.tw. |
| 119 | metaanalysis.tw. |
| 120 | metaanalyses.tw. |
| 121 | overview*.tw. |
| 122 | comprehensive*.tw. |
| 123 | systematic.tw. |
| 124 | research synthesis.tw. |
| 125 | research syntheses.tw. |
| 126 | evidence summary.tw. |
| 127 | 112 or 113 or 114 or 115 or 116 or 117 or 118 or 119 or 120 or 121 or 122 or 123 or 124 or 125 or 126 |
| 128 | 29 and 64 and 111 and 127 |
| 129 | limit 128 to yr="2019 -Current" |

**Ovid Embase**

| **Search string** | **Search terms** |
| --- | --- |
| 1 | exp pregnant woman/ |
| 2 | exp pregnancy/ |
| 3 | exp maternal welfare/ |
| 4 | pregnan*.tw. |
| 5 | gravid*.tw. |
| 6 | exp gestational age/ |
| 7 | gestation*.tw. |
| 8 | exp prenatal care/ |
| 9 | prenatal.tw. |
| 10 | antenatal.tw. |
| 11 | exp perinatal care/ |
| 12 | perinatal.tw. |
| 13 | exp maternal care/ |
| 14 | maternal.tw. |
| 15 | maternity.tw. |
| 16 | exp mother/ |
| 17 | mother*.tw. |
| 18 | child bearing.tw. |
| 19 | antepartum*.tw. |
| 20 | interpregnancy.tw. |
| 21 | exp intrapartum care/ |
| 22 | intrapartum*.tw. |
| 23 | natal.tw. |
| 24 | exp parity/ |
| 25 | parity.tw. |
| 26 | parous.tw. |
| 27 | exp nulligravida/ |
| 28 | nulligravid*.tw. |
| 29 | exp multigravida/ |
| 30 | multigravid*.tw. |
| 31 | exp primigravida/ |
| 32 | exp reproductive health/ |
| 33 | 1 or 2 or 3 or 4 or 5 or 6 or 7 or 8 or 9 or 10 or 11 or 12 or 13 or 14 or 15 or 16 or 17 or 18 or 19 or 20 or 21 or 22 or 23 or 24 or 25 or 26 or 27 or 28 or 29 or 30 or 31 or 32 |
| 34 | exp weight gain/ |
| 35 | weight.tw. |
| 36 | overweight.tw. |
| 37 | gestational weight gain.tw. |
| 38 | GWG.tw. |
| 39 | exp obesity/ |
| 40 | exp maternal obesity/ |
| 41 | obes*.tw. |
| 42 | body mass index.tw. |
| 43 | BMI.tw. |
| 44 | fatness.tw. |
| 45 | exp body fat/ |
| 46 | exp body size/ |
| 47 | size.tw. |
| 48 | exp pregnancy complication/ |
| 49 | exp pregnancy outcome/ |
| 50 | exp smoking/ |
| 51 | smoking.tw. |
| 52 | smok*.tw. |
| 53 | cigar*.tw. |
| 54 | pipe.tw. |
| 55 | pipes.tw. |
| 56 | exp nicotine/ |
| 57 | nicotine.tw. |
| 58 | exp alcohol consumption/ |
| 59 | exp alcohol intoxication/ |
| 60 | alcohol drinking.tw. |
| 61 | exp alcoholism/ |
| 62 | exp alcohol/ |
| 63 | alcohol.tw. |
| 64 | binge*.tw. |
| 65 | drunk*.tw. |
| 66 | liquor.tw. |
| 67 | intoxication.tw. |
| 68 | 34 or 35 or 36 or 37 or 38 or 39 or 40 or 41 or 42 or 43 or 44 or 45 or 46 or 47 or 48 or 49 or 50 or 51 or 52 or 53 or 54 or 55 or 56 or 57 or 58 or 59 or 60 or 61 or 62 or 63 or 64 or 65 or 66 or 67 |
| 69 | exp diet/ |
| 70 | exp low calory diet/ |
| 71 | diet*.tw. |
| 72 | exp weight reduction/ |
| 73 | exp weight loss program/ |
| 74 | weight reduction program*.tw. |
| 75 | weight intervention*.tw. |
| 76 | exp caloric restriction/ |
| 77 | caloric restriction*.tw. |
| 78 | exp feeding behavior/ |
| 79 | food habit*.tw. |
| 80 | food*.tw. |
| 81 | exp eating/ |
| 82 | grazing.tw. |
| 83 | snack*.tw. |
| 84 | healthy eating.tw. |
| 85 | exp exercise/ |
| 86 | exercis*.tw. |
| 87 | physical activit*.tw. |
| 88 | exp lifestyle/ |
| 89 | exp lifestyle modification/ |
| 90 | lifestyle*.tw. |
| 91 | exp smoking cessation/ |
| 92 | cessation.tw. |
| 93 | exp nicotine replacement therapy/ |
| 94 | nicotine replacement.tw. |
| 95 | NRT.tw. |
| 96 | exp nicotine gum/ |
| 97 | exp nicotine lozenge/ |
| 98 | exp nicotine vaccine/ |
| 99 | exp behavior modification/ |
| 100 | habit*.tw. |
| 101 | change*.tw. |
| 102 | modification*.tw. |
| 103 | program*.tw. |
| 104 | assist*.tw. |
| 105 | behavio?r.tw. |
| 106 | avoid*.tw. |
| 107 | prevent*.tw. |
| 108 | maintenance.tw. |
| 109 | management.tw. |
| 110 | gain.tw. |
| 111 | lose.tw. |
| 112 | control.tw. |
| 113 | reduction.tw. |
| 114 | therap*.tw. |
| 115 | relapse*.tw. |
| 116 | exp alcohol abstinence/ |
| 117 | abstain.tw. |
| 118 | abstinence.tw. |
| 119 | intervention.tw. |
| 120 | 69 or 70 or 71 or 72 or 73 or 74 or 75 or 76 or 77 or 78 or 79 or 80 or 81 or 82 or 83 or 84 or 85 or 86 or 87 or 88 or 89 or 90 or 91 or 92 or 93 or 94 or 95 or 96 or 97 or 98 or 99 or 100 or 101 or 102 or 103 or 104 or 105 or 106 or 107 or 108 or 109 or 110 or 111 or 112 or 113 or 114 or 115 or 116 or 117 or 118 or 119 |
| 121 | "review"/ |
| 122 | exp "systematic review"/ |
| 123 | systematic review*.tw. |
| 124 | literature review*.tw. |
| 125 | exp "meta analysis (topic)"/ |
| 126 | meta analysis/ |
| 127 | meta analysis.tw. |
| 128 | meta analyses.tw. |
| 129 | metaanalysis.tw. |
| 130 | metaanalyses.tw. |
| 131 | overview*.tw. |
| 132 | comprehensive*.tw. |
| 133 | research synthesis.tw. |
| 134 | research syntheses.tw. |
| 135 | evidence summary.tw. |
| 136 | 121 or 122 or 123 or 124 or 125 or 126 or 127 or 128 or 129 or 130 or 131 or 132 or 133 or 134 or 135 |
| 137 | 33 and 68 and 120 and 136 |
| 138 | limit 137 to yr="2005 -Current" |
| 139 | limit 138 to english language |
| 140 | 138 not 139 |
| 141 | limit 138 to (conference abstract or conference paper or conference proceeding or "conference review") |
| 142 | 139 not 141 |
|  | limit 137 to yr="2019 -Current" |

**Ovid PsycInfo**

| **Search strings** | **Search terms** |
| --- | --- |
| **#** | **Query** |
| S1 | DE "Pregnancy" |
| S2 | TI pregnan$ OR AB pregnan$ |
| S3 | TI gravid$ OR AB gravid$ |
| S4 | TI gestation$ OR AB gestation$ |
| S5 | DE "Prenatal Care" |
| S6 | TI antenatal$ OR AB antenatal$ |
| S7 | TI perinatal$ OR AB perinatal$ |
| S8 | TI prenatal$ OR AB prenatal$ |
| S9 | TI maternal OR AB maternal |
| S10 | TI maternity OR AB maternity |
| S11 | DE "Mothers" |
| S12 | TI mother$ OR AB mother$ |
| S13 | TI child bearing OR AB child bearing |
| S14 | TI antepartum OR AB antepartum |
| S15 | TI interpregnancy OR AB interpregnancy |
| S16 | TI intrapartum OR AB intrapartum |
| S17 | TI natal OR AB natal |
| S18 | TI parity OR AB parity |
| S19 | TI parous OR AB parous |
| S20 | TI nulligravid$ OR AB nulligravid$ |
| S21 | TI multigravid$ OR AB multigravid$ |
| S22 | TI primigravid$ OR AB primigravid$ |
| S23 | TI pregnant woman OR AB pregnant woman |
| S24 | TI pregnant women OR AB pregnant women |
| S25 | TI pregnant female$ OR AB pregnant female$ |
| S26 | TI pregnant human$ OR AB pregnant human$ |
| S27 | TI pregnant girl$ OR AB pregnant girl$ |
| S28 | TI reproductive woman OR AB reproductive woman |
| S29 | TI reproductive women OR AB reproductive women |
| S30 | TI reproductive female$ OR AB reproductive female$ |
| S31 | TI reproductive human$ OR AB reproductive human$ |
| S32 | TI reproductive girl$ OR AB reproductive girl$ |
| S33 | S1 OR S2 OR S3 OR S4 OR S5 OR S6 OR S7 OR S8 OR S9 OR S10 OR S11 OR S12 OR S13 OR S14 OR S15 OR S16 OR S17 OR S18 OR S19 OR S20 OR S21 OR S22 OR S23 OR S24 OR S25 OR S26 OR S27 OR S28 OR S29 OR S30 OR S31 OR S32 |
| S34 | DE "Weight Gain" |
| S35 | TI weight OR AB weight |
| S36 | DE "Overweight" |
| S37 | TI overweight OR AB overweight |
| S38 | TI gestational weight gain OR AB gestational weight gain |
| S39 | TI GWG OR AB GWG |
| S40 | DE "Obesity" |
| S41 | TI obes$ OR AB obes$ |
| S42 | DE "Body Mass Index" |
| S43 | TI body mass index OR AB body mass index |
| S44 | TI BMI OR AB BMI |
| S45 | TI fatness OR AB fatness |
| S46 | TI body fat OR AB body fat |
| S47 | TI size OR AB size |
| S48 | TI pregnancy complication$ OR AB pregnancy complication$ |
| S49 | DE "Pregnancy Outcomes" |
| S50 | DE "Tobacco Smoking" |
| S51 | TI smoking OR AB smoking |
| S52 | TI smok$ OR AB smok$ |
| S53 | TI cigar$ OR AB cigar$ |
| S54 | TI pipe OR AB pipe |
| S55 | TI pipes OR AB pipes |
| S56 | TI nicotine OR AB nicotine |
| S57 | DE "Alcohol Drinking Patterns" |
| S58 | DE "Alcohol Intoxication" |
| S59 | DE "Alcoholism" |
| S60 | DE "Alcoholic Beverages" |
| S61 | DE "Beer" |
| S62 | DE "Liquor" |
| S63 | DE "Wine" |
| S64 | TI alcohol OR AB alcohol |
| S65 | TI binge$ OR AB binge$ |
| S66 | TI drunk$ OR AB drunk$ |
| S67 | TI liquor OR AB liquor |
| S68 | TI intoxication OR AB intoxication |
| S69 | S34 OR S35 OR S36 OR S37 OR S38 OR S39 OR S40 OR S41 OR S42 OR S43 OR S44 OR S45 OR S46 OR S47 OR S48 OR S49 OR S50 OR S51 OR S52 OR S53 OR S54 OR S55 OR S56 OR S57 OR S58 OR S59 OR S60 OR S61 OR S62 OR S63 OR S64 OR S65 OR S66 OR S67 OR S68 |
| S70 | DE "Diets" |
| S71 | TI diet$ OR AB diet$ |
| S72 | DE "Weight Loss" |
| S73 | DE "Weight Control" |
| S74 | TI weight reduction OR AB weight reduction |
| S75 | TI weight intervention$ OR AB weight intervention$ |
| S76 | TI caloric restriction$ OR AB caloric restriction$ |
| S77 | DE "Eating Behavior" |
| S78 | TI feeding OR AB feeding |
| S79 | TI food$ OR AB food$ |
| S80 | TI eating OR AB eating |
| S81 | TI grazing OR AB grazing |
| S82 | TI snack$ OR AB snack$ |
| S83 | TI healthy eating OR AB healthy eating |
| S84 | DE "Exercise" |
| S85 | TI exercis$ OR AB exercis$ |
| S86 | TI physical activit$ OR AB physical activit$ |
| S87 | DE "Lifestyle Changes" |
| S88 | TI lifestyle$ OR AB lifestyle$ |
| S89 | DE "Smoking Cessation" |
| S90 | TI cessation OR AB cessation |
| S91 | TI nicotine replacement OR AB nicotine replacement |
| S92 | TI NRT OR AB NRT |
| S93 | TI habit$ OR AB habit$ |
| S94 | TI change$ OR AB change$ |
| S95 | DE "Behavior Modification" |
| S96 | TI modification OR AB modification |
| S97 | TI program$ OR AB program$ |
| S98 | TI assist$ OR AB assist$ |
| S99 | TI behavio?r OR AB behavio?r |
| S100 | TI avoid$ OR AB avoid$ |
| S101 | TI prevent$ OR AB prevent$ |
| S102 | TI maintenance OR AB maintenance |
| S103 | TI management OR AB management |
| S104 | TI gain OR AB gain |
| S105 | TI loss OR AB loss |
| S106 | TI lose OR AB lose |
| S107 | TI control OR AB control |
| S108 | TI reduction OR AB reduction |
| S109 | TI therap$ OR AB therap$ |
| S110 | TI relapse$ OR AB relapse$ |
| S111 | TI abstain OR AB abstain |
| S112 | TI abstinence OR AB abstinence |
| S113 | TI intervention$ OR AB intervention$ |
| S114 | S70 OR S71 OR S72 OR S73 OR S74 OR S75 OR S76 OR S77 OR S78 OR S79 OR S80 OR S81 OR S82 OR S83 OR S84 OR S85 OR S86 OR S87 OR S88 OR S89 OR S90 OR S91 OR S92 OR S93 OR S94 OR S95 OR S96 OR S97 OR S98 OR S99 OR S100 OR S101 OR S102 OR S103 OR S104 OR S105 OR S106 OR S107 OR S108 OR S109 OR S110 OR S111 OR S112 OR S113 |
| S115 | S33 AND S69 AND S114 |
| S116 | DE "Literature Review" |
| S117 | TI review$ OR AB review$ |
| S118 | TI systematic review$ OR AB systematic review$ |
| S119 | DE "Meta Analysis" |
| S120 | TI meta analysis OR AB meta analysis |
| S121 | TI meta analyses OR AB meta analyses |
| S122 | TI metaanalysis OR AB metaanalysis |
| S123 | TI metaanalyses OR AB metaanalyses |
| S124 | TI overview$ OR AB overview$ |
| S125 | TI comprehensive$ OR AB comprehensive$ |
| S126 | TI systematic OR AB systematic |
| S127 | TI research synthesis OR AB research synthesis |
| S128 | TI research syntheses OR AB research syntheses |
| S129 | TI evidence summary OR AB evidence summary |
| S130 | S116 OR S117 OR S118 OR S119 OR S120 OR S121 OR S122 OR S123 OR S124 OR S125 OR S126 OR S127 OR S128 OR S129 |
| S131 | S33 AND S69 AND S114 AND S130 |
| S132 | S33 AND S69 AND S114 AND S130 |
|  | limit 129 to (english language and yr="2019 -Current") |

**Cochrane Library**

| **Search strings** | **Search terms** |
| --- | --- |
| #1 | MeSH descriptor: [Pregnant Women] explode all trees |
| #2 | MeSH descriptor: [Pregnancy] explode all trees |
| #3 | pregnan*:ti,ab,kw (Word variations have been searched) |
| #4 | gravid*:ti,ab,kw (Word variations have been searched) |
| #5 | MeSH descriptor: [Gestational Age] explode all trees |
| #6 | gestation*:ti,ab,kw (Word variations have been searched) |
| #7 | MeSH descriptor: [Prenatal Care] explode all trees |
| #8 | antenatal*:ti,ab,kw (Word variations have been searched) |
| #9 | perinatal*:ti,ab,kw (Word variations have been searched) |
| #10 | prenatal*:ti,ab,kw (Word variations have been searched) |
| #11 | maternal:ti,ab,kw (Word variations have been searched) |
| #12 | MeSH descriptor: [Mothers] explode all trees |
| #13 | mother*:ti,ab,kw (Word variations have been searched) |
| #14 | child bearing:ti,ab,kw (Word variations have been searched) |
| #15 | antepartum:ti,ab,kw (Word variations have been searched) |
| #16 | interpregnancy:ti,ab,kw (Word variations have been searched) |
| #17 | intrapartum:ti,ab,kw (Word variations have been searched) |
| #18 | natal:ti,ab,kw (Word variations have been searched) |
| #19 | parity:ti,ab,kw (Word variations have been searched) |
| #20 | parous:ti,ab,kw (Word variations have been searched) |
| #21 | nulligravid*:ti,ab,kw (Word variations have been searched) |
| #22 | multigravid*:ti,ab,kw (Word variations have been searched) |
| #23 | primigravid*:ti,ab,kw (Word variations have been searched) |
| #24 | reproductive:ti,ab,kw (Word variations have been searched) |
| #25 | {or #1-#24} |
| #26 | MeSH descriptor: [Weight Gain] explode all trees |
| #27 | weight:ti,ab,kw (Word variations have been searched) |
| #28 | MeSH descriptor: [Overweight] explode all trees |
| #29 | overweight:ti,ab,kw (Word variations have been searched) |
| #30 | gestational weight gain:ti,ab,kw (Word variations have been searched) |
| #31 | GWG:ti,ab,kw (Word variations have been searched) |
| #32 | MeSH descriptor: [Obesity] explode all trees |
| #33 | obes*:ti,ab,kw (Word variations have been searched) |
| #34 | MeSH descriptor: [Body Mass Index] explode all trees |
| #35 | body mass index:ti,ab,kw (Word variations have been searched) |
| #36 | BMI:ti,ab,kw (Word variations have been searched) |
| #37 | fatness:ti,ab,kw (Word variations have been searched) |
| #38 | body fat:ti,ab,kw (Word variations have been searched) |
| #39 | size:ti,ab,kw (Word variations have been searched) |
| #40 | MeSH descriptor: [Pregnancy Complications] explode all trees |
| #41 | MeSH descriptor: [Pregnancy Outcome] explode all trees |
| #42 | MeSH descriptor: [Smoking] explode all trees |
| #43 | smoking:ti,ab,kw (Word variations have been searched) |
| #44 | smok*:ti,ab,kw (Word variations have been searched) |
| #45 | cigar*:ti,ab,kw (Word variations have been searched) |
| #46 | pipe:ti,ab,kw (Word variations have been searched) |
| #47 | pipes:ti,ab,kw (Word variations have been searched) |
| #48 | nicotine:ti,ab,kw (Word variations have been searched) |
| #49 | MeSH descriptor: [Nicotine] explode all trees |
| #50 | MeSH descriptor: [Alcohol Drinking] explode all trees |
| #51 | MeSH descriptor: [Alcoholism] explode all trees |
| #52 | intoxication:ti,ab,kw (Word variations have been searched) |
| #53 | alcohol*:ti,ab,kw (Word variations have been searched) |
| #54 | MeSH descriptor: [Alcoholic Beverages] explode all trees |
| #55 | binge*:ti,ab,kw (Word variations have been searched) |
| #56 | drunk*:ti,ab,kw (Word variations have been searched) |
| #57 | liquor:ti,ab,kw (Word variations have been searched) |
| #58 | {or #26-#57} |
| #59 | MeSH descriptor: [Diet] explode all trees |
| #60 | MeSH descriptor: [Diet, Reducing] explode all trees |
| #61 | diet*:ti,ab,kw (Word variations have been searched) |
| #62 | MeSH descriptor: [Weight Reduction Programs] explode all trees |
| #63 | weight intervention*:ti,ab,kw (Word variations have been searched) |
| #64 | MeSH descriptor: [Caloric Restriction] explode all trees |
| #65 | caloric restriction*:ti,ab,kw (Word variations have been searched) |
| #66 | MeSH descriptor: [Feeding Behavior] explode all trees |
| #67 | feeding:ti,ab,kw (Word variations have been searched) |
| #68 | MeSH descriptor: [Food Habits] explode all trees |
| #69 | food*:ti,ab,kw (Word variations have been searched) |
| #70 | eating:ti,ab,kw (Word variations have been searched) |
| #71 | grazing:ti,ab,kw (Word variations have been searched) |
| #72 | snack*:ti,ab,kw (Word variations have been searched) |
| #73 | healthy eating:ti,ab,kw (Word variations have been searched) |
| #74 | MeSH descriptor: [Exercise] explode all trees |
| #75 | exercise*:ti,ab,kw (Word variations have been searched) |
| #76 | physical activit*:ti,ab,kw (Word variations have been searched) |
| #77 | MeSH descriptor: [Life Style] explode all trees |
| #78 | lifestyle*:ti,ab,kw (Word variations have been searched) |
| #79 | MeSH descriptor: [Tobacco Use Cessation] explode all trees |
| #80 | MeSH descriptor: [Tobacco Use Cessation Products] explode all trees |
| #81 | nicotine replacement:ti,ab,kw (Word variations have been searched) |
| #82 | NRT:ti,ab,kw (Word variations have been searched) |
| #83 | habit*:ti,ab,kw (Word variations have been searched) |
| #84 | change*:ti,ab,kw (Word variations have been searched) |
| #85 | modification:ti,ab,kw (Word variations have been searched) |
| #86 | program*:ti,ab,kw (Word variations have been searched) |
| #87 | assist*:ti,ab,kw (Word variations have been searched) |
| #88 | behavio*:ti,ab,kw (Word variations have been searched) |
| #89 | avoid*:ti,ab,kw (Word variations have been searched) |
| #90 | prevent*:ti,ab,kw (Word variations have been searched) |
| #91 | maintenance:ti,ab,kw (Word variations have been searched) |
| #92 | management:ti,ab,kw (Word variations have been searched) |
| #93 | gain:ti,ab,kw (Word variations have been searched) |
| #94 | loss:ti,ab,kw (Word variations have been searched) |
| #95 | lose:ti,ab,kw (Word variations have been searched) |
| #96 | control:ti,ab,kw (Word variations have been searched) |
| #97 | reduction:ti,ab,kw (Word variations have been searched) |
| #98 | cessation:ti,ab,kw (Word variations have been searched) |
| #99 | therap*:ti,ab,kw (Word variations have been searched) |
| #100 | relapse*:ti,ab,kw (Word variations have been searched) |
| #101 | abstain:ti,ab,kw (Word variations have been searched) |
| #102 | abstinence:ti,ab,kw (Word variations have been searched) |
| #103 | intervention*:ti,ab,kw (Word variations have been searched) |
| #104 | MeSH descriptor: [Alcohol Abstinence] explode all trees |
| #105 | {or #59-#104} |
| #106 | #25 and #58 and #105 |
| #107 | #106 with Cochrane Library publication date from Jan 2019 to present |
