# Supplementary material for: Features of smoking cessation, alcohol reduction, and diet and physical activity interventions associated with changing behaviours during pregnancy: a systematic review of reviews
Source: J Behav Med. 2026 Aug 6;49(6):985–1001. doi: 10.1007/s10865-026-00702-1 (PMC13748204; doi:10.1007/s10865-026-00702-1)
Supplement: Supplementary file 3 [file 10865_2026_702_MOESM3_ESM.docx]

**Supplement 3: Tables of included SRs**

**Table 3a: Systematic reviews reporting effectiveness of smoking cessation interventions (n=10)**

| **Review** | **Aim of the review** | **Search Strategy** | **Inclusion criteria** | **Exclusion criteria** | - **Included studies** | **Study locations** | **Outcomes reported** |
| --- | --- | --- | --- | --- | --- | --- | --- |
| Acar *et al.* 2022 | To review descriptive studies which handle smoking cessation during pregnancy within the framework of “health behavioral theories” and theory-based intervention studies | Databases: PubMed, Google Scholar, and TÜBİTAK ULAKBİM  Supplementary Searches: None reported. | - Descriptive & intervention studies - Outcome smoking cessation or reduction - Pregnant women | - Not published between 2000 and 2020 - Theoretical basis for intervention not given | - 9 studies - Published: 2000 to 2020 - Study design: RCT n=8; before and after n=1 - n=3301 women | Not reported | Smoking abstinence |
| Brown *et al.* 2019 | To use Behaviour change technique taxonomy to identify Behaviour change techniques components which could be considered ‘promising’ to maintain smoking cessation post-partum | Databases: Medline, EMBASE  Supplementary Searches: None reported. | - RCT - Maintain smoking cessation vs. less intensive intervention or standard care - Healthy pregnant or postpartum women - Reporting smoking status in the post partum period | - Not published in English | - 32 studies - Study design: RCT n=32 - Published 1995-2016 - N=women not reported | USA n= 25  Canada n=2  UK n=2  Germany n=1  Spain n=1  Poland n=1 | Smoking status |
| Chamberlain *et al.* 2013 | To assess the effects of smoking cessation interventions during pregnancy on smoking behaviour and perinatal health outcomes. | Databases: Cochrane Pregnancy and Childbirth Group’s Trials Register  Supplementary searches: reference lists, contacted authors. | - RCTs, cluster-RCTs, and randomised cross-over trials - Quasi-randomised studies if very low risk of interference with sequence generation - Psychosocial interventions for smoking cessation in pregnancy. - Women currently smoking or recently quit smoking, in any care setting. - Implementation strategies of psychosocial interventions to support pregnant women to stop smoking - Pharmacological therapies as part of a tailored intervention where higher levels of psychosocial support provided - Smokeless tobacco use, environmental tobacco smoke exposure or partner smoking in conjunction with interventions aim of supporting pregnant women to stop smoking. - Dissemination studies | - Studies with infant outcome measures such as birthweight, preterm birth, breastfeeding and perinatal mortality - Trials where the sole aim was to reduce: smokeless tobacco use; environmental tobacco smoke exposure - The primary population was not pregnant women - The intervention was not primarily aimed at cessation during pregnancy | - 86 studies - Published: 1976 – 2012 - Study design: RCTs n=86 - n= >29,000 women | USA n=57  Canada n=1  UK n=13  Norway n=3  Sweden n=1  Holland n=1  Spain n=1  Australia n=5  New Zealand n=2 | Smoking cessation (including duration), smoking reduction, infant birthweight (low/very low birthweight), preterm birth, stillbirth, neonatal death, all perinatal death, NICU admission, mode of birth, breastfeeding, psychological effects (anxiety/depression) |
| Chamberlain *et al.* 2017 | To assess the effects of smoking cessation interventions during pregnancy on smoking behaviour and perinatal health outcomes. | Databases: Cochrane Pregnancy and Childbirth’s Trials Register    Supplementary Searches: reference lists, contacted authors. | - RCTs, cluster-RCTs, and randomised cross-over trials - Psychosocial interventions for smoking cessation in pregnancy. - Quasi-randomised studies if very low risk of interference with sequence generation | - None reported | - 88 studies - Published: 1976-2015 - Study design: RCT n=88 - n>26,000 women | USA n=68  UK n=18  Europe n=11 (Norway, Holland, Netherlands, Sweden, Spain, Poland)  Australia/ New Zealand n=9  South America n=1  Canada n=2  Greece n=1  Multiple countries n=1 (Argentina,  Brazil, Cuba and Mexico) | Birth weight, pre-term delivery, mortality outcomes (still birth, neonatal mortality, neonatal intensive care unit (NICU) admission |
| Griffiths *et al.* 2018 | 1.Are digital interventions more effective in increasing smoking cessation rates in pregnancy than  usual care/other control groups?  2. Is the platform of delivery of digital interventions associated with smoking cessation in pregnancy?  3. Which Behaviour change techniques/combinations of Behaviour change techniques, when included in digital interventions, are associated with?  4. Are the number of Behaviour change techniques used in digital interventions associated with smoking cessation in pregnancy?  smoking cessation in pregnancy? | Databases: Academic Search Complete; ASSIA, CINAHL; Cochrane library; EMBASE; Medline: PsycINFO; Scopus and Web of Science    Supplementary searches: Research registers of NIHR UK Clinical Trials Gateway; ClinicalTrials.gov; ISRCTN. Contacted authors. Reference lists. | - RCTs and quasi-RCTs - English language. - No restrictions on publication date - Women at any stage of pregnancy, reporting to be current cigarette smokers. - Digital interventions delivered by computer, video or DVD, mobile telephone or portable handheld device. This included email, video, DVDs, websites or web-based games, mobile or tablet applications and SMS text messages or MMS multimedia messages. - Reporting smoking abstinence. | - Studies with only ex-smokers or post-natal participants. | - 12 studies - Study design: RCT and quasi-RCT (numbers not reported) - Published: 1991 to 2017 - n=2306 women | USA n=8  UK n=4 | Smoking abstinence reported |
| Heminger *et al.* 2016 | To summarize mHealth programs (text messaging and app-based interventions) currently available for pregnant smokers and, where available, to summarize study results and outcomes. | Databases: PubMed, PsychINFO and Medline    Supplementary searches: iTunes and GooglePlay app stores, gray literature searches using Google | - Include a mHealth element (text messaging or mobile app) - Smoking cessation among women during and/or after pregnancy - Report smoking cessation outcome. - Programs aimed at a larger target audience (ie, “adults”) with an explicit pregnancy component | - General text messaging programs and applications that made no mention of pregnancy. | - 7 studies - Published: 2012-2015 - Study design: RCT n=3, other design n=4 - n=1,009 women when reported, + 2 studies reporting study sample as “10,000-50,000 downloads” and “100-500 downloads” | USA n=2  UK n=2  Australia n=1  Canada n=2 | Smoking cessation during pregnancy or additional elements related to general pregnancy health (not specified) |
| Hussain *et al.* 2020^a^ | To systematically evaluate  mHealth interventions used in pregnancy in high-income countries and their effects on maternal health behaviors and maternal-fetal health outcomes | Databases: MEDLINE via PubMed, EMBASE, Web of Science, Cochrane Database of Controlled Trials, CINAHL, and PsycINFO  Supplementary Searches: Hand searches of bibliographies | - English-language - pregnant women who utilized pregnancy-related mobile phone interventions - developed or high-income countries - stand-alone mobile phone–based interventions | - Technology interventions aimed solely at communication between patients and clinicians without educational, motivational, or interactive component - Interventions not primarily intended for mobile phone use, eg, websites. - Focused solely on neonatal health (e.g., feeding support or growth tracking). - Published abstracts or conference proceedings - Feasibility or desirability of hypothetical interventions - Outcomes with fewer than 2 weeks of intervention use. | - 9 studies - Published: 2012 to 2018 - Study design: RCT n=7; observational n=2 - n=1,908 women | USA n=5  Japan n=1  UK n=3 | smoking cessation |
| Su and Buttenheim 2014 | To investigate the prevention of postpartum smoking relapse for nonspontaneous quitters. | Databases: PubMed, Cochrane Library, CINAHL.  Supplementary Searches: Hand searches of bibliographies | - Smoking cessation interventions - During pregnancy and continued for at least 1 month postpartum. | - Qualitative or descriptive studies - Still in trial - No intervention - Began postpartum - Did not follow participants past 1 month postpartum | - 32 studies - Published: 1990 to 2015 - Study design: RCT n=27, non-randomised CT n=5 - n= 24,095 women | Not reported | Smoking cessation. Identifying differences in quit and relapse rates between spontaneous and nonspontaneous quitter. Postpartum smoking relapse. |
| Tahan *et al* 20 | To assess the effect of digital health, biomarker feedback and nurse or midwife-led counselling interventions on abstinence in pregnant smokers during pregnancy and postpartum. | Databases:  Embase, Medline, Medline IP, Web of Science, Google Scholar, PsychINFO, CINAHL, PubMed  Supplementary Searches: Hand searches of references from search results and contacted authors of studies where full text were not available | - Counselling, advice, coaching, home visitation or other psychological interventions that were delivered by nurses or midwives - Quantitative efficacy studies (ie, randomised, quasi-experimental evaluation, real-life and prospective intervention trials) | - Pregnant adolescent only studies, studies that did not quantify effect of intervention on pregnant smokers, post hoc analysis of original studies, feasibility, acceptability and development of interventions only, study protocols and studies with no comparator, no full text or non-English were excluded | - 57 studies - Published 1982 to 2021 - Study design: RCT = 41, quasi-experiment n=9, prospective intervention studies n=9, controlled trials/projects n=3 - N= 69,082 participants | USA n=25  UK n=11  Rest of Europe n=12  Austrailia n=2  Canada n=2  Argentina n=1  South Africa n=1  Turkey n=1  New Zealand n=1 | At least one abstinence outcome |
| Wilson *et al.* 2018 | Among RCTs for women smoking tobacco during preg- nancy, what are the effect sizes of contingency management for prenatal smoking cessation compared with traditional psycho- therapeutic interventions with no financial incentives? | Databases: PubMed, PsycINFO, Web of Science, the Cochrane Library, and EMBASE  Supplementary Searches: bibliographies of included trials and systematic reviews. | - RCTs - Pregnant women - At least 3 months follow up - Active treatment compared to standard care | - Animal studies - Non-incentive or non-psychotherapeutic intervention techniques | - 22 studies - Published: 1994-2017 - Study design RCT n=22 - N= 6581 women | Not reported | Smoking cessation, abstinence at three dedicated time points. |

a. Review by Hussain et al 2020 also reported data for diet/physical activity interventions.

**Table 3b: Systematic reviews reporting effectiveness of alcohol interventions (n=6)**

| **Review** | **Aim of the review** | **Search Strategy** | **Inclusion criteria** | **Exclusion criteria** | **Included studies** | **Study locations** | **Outcomes reported** |
| --- | --- | --- | --- | --- | --- | --- | --- |
| Froschl *et al.* 2013 | Which preventive/ interventional strategies for women are effective and efficient to prevent fetal alcohol syndrome | Databases: 30 including Medline and Embase – full list not provided.  Supplementary searches: None reported | - Not reported | - Not reported | - 10 studies (8 primary, 2 secondary) - Published: not reported - Study design: not described - n=not reported | not reported | FAS, alcohol intake/ abstinence, fetal mortality, birth weight, gestational age, preterm, NICU |
| Gomez *et al.* 2020 | (i) what type of interventions have been used to reduce drinking during pregnancy and motherhood?; (ii) are these interventions effective?; and (iii) what BCTs are used in effective interventions? | Databases: PsycINFO, PubMed and Scopus  Supplementary Searches: none reported. | - RCTS - Alcohol interventions vs control - Pregnant women with dependents <18 years | - Not reported | - 34 studies (20 studies in pregnancy) - Published: 1982-2019 - Study Design; RCT n=16, C-RCT n=4 - N=8467 women | Argentina n=1  USA n=13  UK n=2  Spain n=1  Netherlands n=1  Australia n=1  South Africa n=1 | Abstinence of alcohol in pregnancy and reduction of drinking in motherhood |
| Minozzi *et al.* 2024 | To assess the efficacy of psychosocial interventions and medications to reduce or stop alcohol consumption during pregnancy | Databases: Cochrane Drugs and Alcohol Group Specialised Register (via CRSLive), Cochrane Central Register of Controlled Trials (CENTRAL), MEDLINE, Embase, CINAHL,Web of Science, PsycINFO  Supplementary searches: Ongoing and unpublished studies via ClinicalTrials.gov and the World Health Organization (WHO) International Clinical Trials Registry Platform (ICTRP). Hand searched references of topic-related systematic reviews and included studies. | - RCTs that compared medications or psychosocial interventions, or both, to placebo, no intervention, usual care, or other medications or psychosocial interventions used to reduce or stop alcohol use during pregnancy | - Pregnant individuals who did not drink alcohol at baseline. - Individuals with a diagnosis of a substance use disorder involving substances other than alcohol | - 8 studies - Published 1995 to 2014 - Study design: RCT n=8 - N=1369 participants | UA n=6  Spain n=1  Netherlands n=1 | Abstinence from alcohol, reduction in alcohol consumption, retention in treatment, and women with any adverse event. |
| Oh *et al.* 2022 | To assess whether digital interventions are effective in preventing alcohol consumption during pregnancy or pregnancy-planning period, and the differential effectiveness of alternative digital intervention platforms | Databases: PubMed, Embase, CINAHL, and Web of Science  Supplementary searches: reference lists of identified studies | - Targeted pregnant women or women planning to become pregnant - Digital intervention aiming to prevent alcohol consumption - Reported alcohol abstinence. | - Studies where interventions included (social media where subjects communicated with one another were excluded. | - 6 studies - Published: 2012 to 2018 - Study design: RCT n=6 - n= 972 women | USA n=5  Netherland n=1 | Reduction in alcohol consumption |
| Popova *et al.* 2023 | To investigate the effectiveness of alcohol brief interventions (BIs) in eliminating or reducing 1) alcohol consumption during pregnancy and 2) PAE- related adverse neonatal outcomes | Databases: MEDLINE, CINAHL, PsycINFO, EMBASE, CENTRAL, Web of Science, Google Scholar, ICH-GCP Clinical Trial Registry, European Monitoring Centre for Drugs and Drug Addiction, Canadian Centre on Substance Use and Addiction.  Supplementary Searches: none reported. | - Experimental or quasi- experimental design. - Control group - Intervention was a BI - Pregnant women - Alcohol reported separately from other substance use | - BI combined with pharmacological interventions - BI conducted outside of the pregnancy period. | - 25 studies - Published: 1986-2020 - Design: RCTs n=21, C-RCTs n=3, pre-post study n=1 - n>7862 | USA n=16  South Africa n=2  Brazil n=1  Ireland n=1  Israel n=1  Netherlands n=1 Norway n=1  Spain n=1  Sweden n=1  UK n=1 | Change in alcohol use (quantity and frequency).  Preterm delivery, NICU admission, birthweight, body length, head circumference, APGAR score |
| Samawi *et al.* 2021 | To synthesise the available evidence on psychological interventions to reduce alcohol consumption among pregnant and postpartum women. | Databases: Medline, Embase, Global Health, PsychInfo, Cinahl  Plus and Web of Science  Supplementary searches: handsearching reference lists of included articles and past systematic reviews | - RCTs and quasi-experimental studies - Pregnant and postpartum women who drink or are at-risk of drinking. - Non-pharmacological intervention aimed at the reduction of alcohol consumption - Change in alcohol use - Published in peer-reviewed journals - English language. | - Qualitative, cross-sectional, before- after, observational. - No control group - Not the population of interest. - Enrolled >6 months post-delivery. - Pharmacological interventions - Aimed at changing attitude, knowledge, intentions re alcohol consumption, or at general prenatal or postnatal health promotion. - Abstinence or reduction outcomes not reported. - Neonatal outcomes and/or adverse obstetric outcomes only - Editorials, commentaries, case reports, clinical guidelines, book chapters, conference abstracts or posters, and dissertations. Grey literature. - Non-English language. | - 11 studies - Published: 1999- 2016 - Study design: RCTs n=9 and cluster RCT n=2 n= 2198 women | USA n= 10  Netherlands n=1 | Alcohol reduction |

**Table 3c: Systematic reviews reporting effectiveness of diet and/or physical activity interventions (n=51)**

| **Review** | **Aim of the review** | **Search Strategy** | **Inclusion criteria** | **Exclusion criteria** | **Included studies** | **Study locations** | **Outcomes reported** |
| --- | --- | --- | --- | --- | --- | --- | --- |
| Agha *et al.* 2014 | To assess the efficacy of behavioural interventions for weight management during pregnancy. | Databases: Pub Med, Scopus, Cochrane Library, CINAHL, PsycINFO  Supplementary searches: reference list; NICE guidance on obesity; US NIH clinical Trials Registry; Google Scholar; ONS and National Obesity Observatory contacted; NICE personal communication | - English language - Parallel group study - Behavioural interventions on weight management with standard maternity care/no intervention/ placebo. - Women planning to get pregnant or those who are already pregnant. - Underweight, normal weight, overweight, obese or morbidly obese. | - Studies of women with conditions such as diabetes and PCOS. - Interventions based on weight reducing pharmaceutical or surgical interventions. - Interventions aimed at other conditions/diseases in pregnancy or postpartum weight management. | - 14 studies - Published: 2002 to 2011 - Study design: RCT n=12, CCT n=1, CBA n=1 - n=2,734 women | USA n=5  Australia n=2  Finland n=2  Sweden n=1  Norway n=1  Belgium n=1 Denmark n=1  Canada n=1 | GWG |
| Allen *et al.* 2014 | To evaluate the effect of dietary and lifestyle interventions with the potential to modify metabolic risk factors on the risk of PE | Databases: MEDLINE, EMBASE and Cochrane  Supplementary searches: Reference lists of all primary and review articles. | - RCTs - pregnant women - dietary and lifestyle interventions with potential to modify metabolic risks | - Non-randomized - Animal studies | - 18 studies - Published: 1992 to 2012 - Study design: RCT n= 18 - n=8,712 women | Not Reported | Preeclampsia |
| Baradwan *et al*. 2024 | To investigate the effectiveness of the Pilates exercise program during pregnancy on different delivery outcomes. | Databases: PubMed, Scopus, Cochrane Library, ISI Web of Science  Supplementary searches: None reported. | - Healthy pregnant women attending antenatal classes - Pilates exercise intervention - Control: routine antenatal care - Outcomes: and (5) Study - Randomized controlled trials (RCTs), non-RCTs, and cohort or case-control studies | - Review articles, opinion articles, letters, abstracts only, editorial articles - Studies without numerical data for analysis - Studies without a control group | - 7 included studies - Published 2017-2022 - Study design: RCT n=4, non RCT n=1, cohort study n=1, case control study n=1 - N=1003 patients | Turkey n=3  Spain n=1  Austrailia n=1  Iran n=1  Cyprus n=1 | Obstetric, maternal, and neonatal outcomes |
| Beetham *et al.* 2019 | To investigate the effects of vigorous intensity exercise during pregnancy on birth weight. | Databases: PubMed, Medline, EMBASE, Cochrane Library, Web of Science and CINAHL  Supplementary searches: none reported. | - RCTs, quasi-experimental studies, cohort studies and case-control studies. - Studies were required to include an intervention or report of pregnant women performing vigorous exercise in any trimester compared to lower intensity exercise or standard care. - Outcomes: birth weight, SGA, LBW, gestational age at delivery, preterm birth, or gestational weight gain. | - Studies where the sample was a population of women specifically with gestational diabetes mellitus (However, studies reporting gestational diabetes as an outcome were included in the study) | - 15 studies (13 in statistical analysis) - Published; 1987 to 2017 - Study design: 5 RCTs and 10 cohort studies (5 RCT’s and 8 cohort studies in statistical analysis) - n=32,748 women (7,848 in analysis) | Canada n=2  Brazil n=1  New Zealand n=1  China n=1  Australia n=1  USA n=5  Denmark n=1  UK n=2  International n=1 | Birth weight, incidence of small for gestational age, low birth weight, risk of prematurity, gestational age at delivery and maternal weight gain |
| Bennett *et al.* 2023 | To examine the interaction between both timing and volume of supervise exercise on GDM risk | Databases: Cochrane library, CINAHL, EMbase, Medline  Supplementary searches: OpenGrey, British Library database, reference list searches | - RCT - Pregnant women without contraindication to exercise, previous GDM, diabetes - Compared to standard antenatal care - Reporting GDM - Any type of exercise, any frequency/ intensity or duration | - Combined intervention (diet and exercise) | - 20 studies - Published 2012- 2019 - Study design: RCT - n=6812 | Not reported | GDM incidence |
| Cancela-Carral *et al.* 2022 | Identify the effects generated by different aquatic physical activity programs for pregnant women on their physical, anthropometric and cognitive components | Databases: MEDLINE-PubMed, Scopus, Wed of Science, PEDro, SPORT-Discus.  Supplementary searches: none | - Pregnant women - RCT - Aquatic physical activity intervention - Reporting data on fitness, anthropometry and/or cognition | - Not reported | - 17 studies - Published: not reported - Study design: RCT - n=2439 | Not reported | GWG, birth weight, fitness, anthropomorphic and cognitive factors. |
| Cantor *et al.* 2021 | Synthesise evidence on interventions to inform guidelines on healthy weight gain in pregnancy. | Databases: MEDLINE, Cochran central library, Cochrane database of systematic reviews  Supplementary searches: reference lists | - Women pregnancy or planning pregnancy - BMI > 18.5 - Intervention vs control (usual care, attention control, minimal intervention) | - Women with underweight | - 68 studies - Published: 2002-2019 - Study design: RCT n=64, non-RCT n=4 - n=25,789 | Not reported | GWG, GDM, hypertension, caesarean delivery, emergency caesarean delivery, macrosomia, LGA, preterm birth |
| Chan *et al.* 2019 | To provide an in-depth review on the major components of physical activity interventions involving land-based exercises that are dedicated for pregnant women and to examine the effectiveness of the interventions on improving exercise self-efficacy, physical activity levels, and pregnancy-related outcomes including depression, pregnancy-related pain, and GWG. | Databases: PubMed, OVID MEDLINE, EMBASE, PsycINFO, and CINAHL    Supplementary searches: None reported | - RCTs or controlled clinical trials (CCT) - Physical activity interventions or lifestyle interventions that contain a physical activity component - Outcomes: exercise self-efficacy, physical activity levels, depression, lumbopelvic pain, and/or GWG - Healthy pregnant women, aged 18 or above, singleton pregnancy, free from medical or obstetrical contraindication against physical activity in pregnancy. - Published before September 2018 | - Pregnant teenage women - Not published in English - Study protocols, case reports, or qualitative studies | - 29 studies - Published: 2000-2018 - Study design: RCT n=28, CCT n=1 - n= 8,257 women | Brazil n=2  Canada n=2  Columbia n=2  Croatia n=1  Finland n=1  Iran n=2  Norway n=9  South Africa n=1  Spain n=2  Sweden n=1  Taiwan n=2  Thailand n=1  Turkey n=2  USA n=1 | GWG, pain and depression, self-efficacy, physical activity level, quality of life |
| Craemer *et al.* 2019 | To evaluate nutrition-only, exercise- only, and nutrition-plus-exercise interventions for optimizing GWG based on the 2009 IOM guidelines. | Databases: PubMed, Google Scholar  Supplementary searches: 2015 Cochrane Review (Muktabhant *et al.* 2015). | - RCTs - At least 20 singleton pregnant women - 18 years or older - Control group standard obstetric care - Report means of GWG based on baseline BMI or pre-pregnancy BMI - Use 2009 IOM guidelines for GWG. | - Studies published before 2009 - Mean GWG not reported - Study in trial state | - 31 studies - Published: 2009 to 2016 - Study design: RCT n=31 - n=8,558 women | Netherlands n=1  USA n=4 Turkey n=1 Belgium n=1 Australia and New Zealand n=3  Italy n=2 Norway n=2 Ireland n=3 Taiwan n=1 Canada n=3 Finland n=3 China n=1 Brazil n=1  UK n=1 Germany n=1 Denmark n=2 Spain n=1 | GWG |
| Currie *et al.* 2013 | To evaluate the content of PA interventions to reduce the decline of PA in pregnant women with a specific emphasis on the behaviour change techniques that were employed  to elicit this change | Databases: EMBASE,  Medline, AMED, PsycInfo, SportDiscus, , PEDro, Cochrane CENTRAL library.   Supplementary searches: Index to Thesis, DART Europe, NIHR, ClinicalTrials.gov.  Hand searches of relevant journals. Citation searches using Web of Knowledge. | - RCTs - Pregnant women - No known medical or obstetric condition - BMI in normal, overweight or obese category - Interventions designed to maintain or increase PA during pregnancy as an outcome measure | - Only abstract available - Inclusion of women with diabetes - Intervention designed for underweight pregnant women | - 14 studies - Published: 2002-2012 - Study design: RCT n=14 - n=2,553 women | Not reported | Change in physical activity during pregnancy |
| Davenport *et al.* 2018a | To examine the influence of prenatal exercise on depression and anxiety during pregnancy and the postpartum period. | Databases: MEDLINE, EMBASE, PsycINFO, Cochrane Database of Systematic Reviews, Scopus and Web of Science Core Collection, CINAHL Plus with Full-text, Child Development & Adolescent Studies, ERIC, Sport Discus, the Trip Database  Supplementary searches: Cochrane Central Register of Controlled Trials, ClinicalTrials.gov | - Pregnant women without contraindication to exercise. - Objective or subjective measures of frequency, intensity, duration, volume or type of exercise. - Acute or habitual prenatal exercise and interventions including exercise alone or in combination with other interventions | - Women with absolute or relative contraindications to exercise. - Exercise began after the initiation of labour. - Case studies and not original research (narrative or systematic reviews and meta-analyses). - Studies not published in either English, Spanish or French. | - 52 studies - Published: 2008 to 2016 - Study design: RCTs n=26, non-RCT n=7, cohort n=10, cross-sectional n=6, case-control n=3 - n=131,406 women | USA n=18  Canada n=3 Sweden n=1 India n=2 Multi- countries n=1  Australia n=3  Egypt n=1  Norway n=4  Poland n=2  Iran n=2 Finland n=1 Brazil n=1  UK n=3 Spain n=3 Serbia n=1 Colombia n=1  South Korea n=2  Denmark n=1  Japan n=1  Portugal n=1 | Prenatal and postnatal anxiety and depression |
| Davenport *et al.* 2018b | To perform a systematic review and meta-analysis of the relationships between prenatal exercise and GDM, GH and PE. | Databases: MEDLINE, EMBASE, PsycINFO, Cochrane Database of Systematic Reviews, Scopus and Web of Science  Core Collection, CINAHL Plus with Full-text, Child Development & Adolescent Studies, ERIC, Sport Discus, the Trip Database  Supplementary searches: Cochrane Central Register of Controlled Trials, ClinicalTrials.gov | - Pregnant women without contraindication to exercise. - Objective or subjective measures of frequency, intensity, duration, volume or type of exercise (reports outcomes for PA and combined diet/PA separately). - Relevant outcomes were GDM, GH and PE. | - Women with absolute or relative contraindications to exercise. - Exercise began after the initiation of labour. - Case studies and not original research (narrative or systematic reviews and meta-analyses). - Studies not published in either English, Spanish or French. | - 106 studies - Published: 1974 to 2017 - Study design: RCTs n=65, non-RCTs n=9, cohort n=13, cross-sectional n=11, case-control n=8 - n=273,182 women | Australia n=5  USA n=33  Thailand n=1  Spain n=11  Sweden n=3  Canada n=11  India n=1  Brazil n=3  Belgium n=1  New Zealand n=2  Denmark n=3  Finland n=4  Norway n=6  China n=6  UK n=3  Kosovo n=1  Germany n=1  Croatia n=1  Netherlands n=2  Iran n=3  Italy n=1  Pakistan n=1  Greece n=1  Multiple countries n=2  Japan n=1 | GDM, preeclampsia, Gestational hypertensive |
| Davenport *et al.* 2018c | To perform a systematic review and meta-analysis to explore the relationship between prenatal exercise and glycaemic control. | Databases: MEDLINE, EMBASE, PsycINFO, Cochrane Database of Systematic Reviews, Scopus and Web of Science Core Collection, CINAHL Plus with Full-text, Child Development & Adolescent Studies, ERIC, Sport Discus, the Trip Database  Supplementary searches: Cochrane Central Register of Controlled Trials, ClinicalTrials.gov | - Pregnant women without contraindication to exercise. - Objective or subjective measures of frequency, intensity, duration, volume or type of exercise. - Original studies of any design (RCT and observational) - Outcomes were indicators of glucose tolerance | - Women with absolute or relative contraindications to exercise. - Exercise began after the initiation of labour. - Case studies and not original research (narrative or systematic reviews and meta-analyses). - Studies not published in either English, Spanish or French | - 58 studies - Published: 1981 to 2017 - Study design: RCTs n=31, non-RCTs n=7, cohort n=8, cross-sectional n=12 - n=8,699 women | Not reported | Glycaemic control: Maternal blood glucose before, during and after exercise, fasting blood glucose |
| Davenport *et al.* 2019a | The aim of the study is to perform a systematic review of the relationship between prenatal exercise and fetal or new-born death. | Databases: MEDLINE, EMBASE, PsycINFO, Cochrane Database of Systematic Reviews, Cochrane Central Register of Controlled Trials, Scopus and Web of Science Core Collection, CINAHL Plus, Child Development and Adolescent Studies, ERIC, Sport Discus, ClinicalTrials.gov, Trip Database.  Supplementary searches: None reported. | - Pregnant women without contraindication to exercise - Objective or subjective measures of frequency, intensity, duration, volume or type of exercise. - Interventions including exercise alone or in combination with other interventions. - Eligible comparators no exercise or different frequencies, intensities, durations, volumes and types of exercise or exercise in different trimesters. | - Women with absolute or relative contraindications to exercise. - Exercise began after the initiation of labour. - Case studies and not original research (narrative or systematic reviews and meta-analyses). - Studies not published in either English, Spanish or French. | - 46 studies - Published: 1976 to 2016 Study design: RCT n=30, n- RCT n=8 (4 cohort, 1 cross-sectional, 3 case- control studies) - n=266,778 women | Finland n=4 UK n= 3 USA n=10 Denmark n=3 India n=2 Canada n=2 Japan n=2 Germany n=1  China n=3 Sweden n=5 Australia n=2 Belgium n=1 South Africa n=1  Norway n=3  Benin n=1  New Zealand n=1  Brazil n=1  Greece n=1 | Miscarriage, perinatal mortality |
| Davenport *et al.* 2019b | To assess the associations between prenatal PA (in terms of frequency, intensity, type and volume of PA), with maternal harms, labour and delivery outcomes. | Databases: MEDLINE, EMBASE, PsycINFO, Cochrane Database of Systematic Reviews, Scopus and Web of Science Core Collection, CINAHL Plus with Full-text, Child Development & Adolescent Studies, ERIC, Sport Discus, the Trip Database  Supplementary searches: Cochrane Central Register of Controlled Trials, ClinicalTrials.gov. | - Pregnant women without contraindication to exercise. - Objective or subjective measures of frequency, intensity, duration, volume or type of exercise (reports outcomes for PA and combined diet/PA separately). | - Women with absolute or relative contraindications to exercise. - Exercise began after the initiation of labour. - Case studies and not original research (narrative or systematic reviews and meta-analyses). - Studies not published in either English, Spanish or French | - 113 studies Published: 1974 to 2017 Study design: RCTs n=79, non-RCTs n=12, observational n=22 - n=52,858 women | USA n=32 Spain n=12 Australia n=7  Brazil n=7 Norway n=7 UK n=6 Canada n=5 Thailand n=4 Sweden n=4 Netherlands n=4  Denmark n=3  China n=3  India n=1 Poland n=2 Japan n=2  Italy n=2 Benin n=1 Belgium n=1 Switzerland n=1 Colombia n=1  Iran n=1 Germany n=1  Croatia n=1 Finland n=1 Mexico n=1  Turkey n=1  France n=1  New Zealand n=1 | preterm/prelabour rupture of membranes, caesarean section, instrumental delivery, induction of labour, length of labour, vaginal tears, fatigue, injury, musculoskeletal trauma, maternal harms (author defined) and diastasis recti) |
| Di Mascio *et al.* 2016 | To evaluate the effects of exercise during pregnancy on the risk of PTB. | Databases: MEDLINE, EMBASE, Web of Science, Scopus, OVID, and Cochrane Library  Supplementary Searches: ClinicalTrial.gov | - RCTs reporting PTB as an outcome. - Women with uncomplicated, singleton pregnancies without any obstetric contraindication to PA. | - Only underweight or only overweight or obese women - Diet counselling, weight monitoring, exercise reduction - Quasi-RCTs | - 9 studies - Published: 1990 to 2016 - Study design: RCT n=9 - n=2,059 women | Spain n=5  Brazil n=2  USA n=1  Norway n=1 | Pre-term birth, gestational age, spontaneous and operative vaginal delivery, caesarean, GDM, hypertensive disorders, birthweight and low birthweight <2500g. |
| Gresham *et al.* 2014 | To synthesize the best of the available evidence by conducting a systematic review and meta-analysis to determine whether dietary interventions before or during pregnancy have any effect on neonatal or infant outcomes. | Databases: EMBASE; Pre-Medline; MEDLINE; Proquest; Web of Science; CINAHL; Scopus; The Cochrane Library; Mosby Index; and Maternity and Infant Care.    Supplementary searches: hand searching | - Preconception or pregnant women, any age, weight or BMI, without date limits. - At least three RCTs per outcome - RCTs or pseudo-RCTs on dietary interventions with any intensity, frequency or timing. - Positive or neutral methodological quality | - Health conditions that may influence dietary intake. - Non-English language. - Studies in animals - Case study, editorial, conference proceeding - Studies in GWG or nutrient supplementation - Duplicate populations. - Multiple birth - Negative methodological quality | - 29 studies (31 publications) - Published: 1978 to 2011 - Study design: RCT n=29 - n=10,026 women | USA n=10 Netherlands n=3  Denmark n=3 Finland n=2 Australia n=1 Greece n=1 Italy n=1  Norway n=1  Chile n=1  Gambia n=1 Taiwan n=1 Columbia n=1  East Java n=1  Iran n=1 South Africa n=1 | Neonatal outcomes (e.g. birth weight, length, head circumference, Apgar score), perinatal mortality |
| Gresham *et al.* 2016 | To investigate if dietary interventions have any effect on pregnancy outcome. | Databases: EMBASE; Pre-Medline; MEDLINE; Proquest; Web of Science; CINAHL; Scopus; The Cochrane Library; Mosby Index; and Maternity and Infant Care.  Supplementary searches: reference lists of included papers. | - Studies that reported any pregnancy, neonatal or infant outcomes - Any age, weight or BMI - No date limits. - Any healthy, human population. - Randomised or pseudo-RCTs on dietary interventions. - Any intensity, frequency or timing of intervention. - Positive or neutral quality. | - Health conditions that may influence dietary intake. - Non-English language. - Studies in animals - Case study, editorial, conference proceeding - Studies in GWG or nutrient supplementation - Duplicate populations. - Multiple birth - Negative methodological quality | - 14 studies - Published: 1989 to 2011 - Study design: RCT n=14 - n=9,756 women | USA n=5  Australia n=1 Denmark n=2 Greece n=1 Italy n=1 Finland n=1 Netherlands n=2  Norway n=1 | Pregnancy outcomes GDM, pregnancy hypertensive disorders, Maternal blood pressure, caesarean section, length of gestation |
| Guillemette *et al.* 2018 | To characterize and quantify the specific impact of prenatal exercise on children’s cardiometabolic health markers, at birth and in childhood. | Databases: Medline, EMBASE, Scopus, CINAHL, SPORTDiscus  Supplementary searches: CENTRAL, bibliographies of relevant narrative and systematic reviews and included studies. | - RCT or prospective cohort study - Pregnant women Aerobic and/or resistance (strength) training exercises ≥4weeks in duration - Birth weight (primary outcome), body composition (fat and/or lean mass), LGA; follow up (any time after birth) weight, body composition (fat and/or lean mass), blood pressure, blood glucose, blood lipids). | - Non-human - Intervention >60% non-aerobic/ resistance training (e.g. yoga) - Acute exercise, or training <4 weeks in duration - Exposures not distinguishing PA from aerobic/ resistance training - Not linking prenatal exercise to offspring outcomes - Unequal controls | - 54 studies - Published: 1993 to - 2017 Study design: RCT n=39, Cohort n=15 - n=6,870 neonates | Finland n=1 USA n=12 Sweden n=2 Iran n=2 Brazil n=7 Spain n=7 Australia n=2 New Zealand n=2  Norway n=3 Netherlands n=1  Colombia n=1  Kosovo n=1 UK n=3 Ireland n=1 Canada n=2 Croatia n=1 Denmark n=5  China n=1 | Offspring cardiometabolic risk factors including weight, adiposity, and blood pressure at birth and in childhood, the effects of maternal body mass index (BMI) and training variables. (Birth weight, large-for-gestational age status, fat and lean mass, dyslipidemia, dysglycemia, and blood pressure) |
| Guo *et al.* 2019 | To investigate whether lifestyle interventions during pregnancy prevent GDM; and explore which characteristics related to participants, interventions, and study design contributed to an effective intervention. | Databases: Pubmed, Scopus, Cochrane  Supplementary searches: reference lists of included studies and relevant reviews | - RCTs - evaluating any lifestyle interventions during pregnancy with the potential to prevent GDM. | - Studies with overlapping subject groups. - Studies with no GDM case in the intervention group or the control group. - Studies with severely unbalanced risk factors or dropout rates that were suspected to exaggerate the effect of interventions | - 47 studies - Published: 2002 to 2017 - Study design: RCT n=47 - n=15,745 participants | Spain n=4  Canada n=3  Belgium n=1  Italy n=2  Australia n=8  Norway n=3  USA n=9  China n=4  Finland n=4  Netherlands n=1  UK n=1  India n=1  Germany n=1  Denmark n=3  New Zealand n=1  Croatia n=1  Ireland n=1 | GDM |
| Hanifi *et al.* 2023 | To determine if RD involvement during pregnancy is associated with a lower prevalence of adverse birth outcomes in the United States and Canada | Databases: PubMed, CINAHL, Embase, Web of Science  Supplementary searches: reference lists | - Conducted in USA or Canada - Include reporting of low birth weight, SGA, LGA, macrosomia, infant mortality, pre-term birth - Direct involvement with RD | - Reviews, editorials, grey literature | - 14 studies - Published 1989-2018 - Study designs: n=2 cross-sectional, n=2 case-control, n=3 retrospective cohort, n=5 RCTs, n=1 pilot, n=1 implementation trial - n=6,566 women | USA n=13  Canada n=1 | low birth weight, SGA, LGA, macrosomia, infant mortality |
| Harrison *et al. 2023* | To evaluate intervention components using the Template for Intervention Description and Replication (TIDieR) framework to inform implementation of antenatal lifestyle interventions in routine antenatal care. | Databases: Cochrane Database of Systematic Reviews, Database of Abstracts of Reviews of Effects, Cochrane Central Register of Controlled Trials, Health Technology Assessment Database, MEDLINE, Embase  Supplementary searches: reference lists of eligible articles | RCTs of antenatal diet, physical activity, or mixed interventions in pregnant individuals that reported mean GWG per group. | Individuals with multiple pregnancies  Preexisting conditions (eg, gestational diabetes)  Non–lifestyle interventions (ie, GWG monitoring only)  Published prior to 1990. | 99 studies  Published 1999-2020  Study design: RCT n=99  N=34565 pregnant individuals | USA n=25  Spain n=16  Austrailia n= 9  UK n=5  Brazil n=5  Iran n=4  Norway n=4  China n=4  Canada n=3  Ireland n=3  Italy n=3  New Zealand n=3  Sweden n=2  Belguim n=2  Finland n=2  Netherlands n=2  Turkey n=2  Denmark n=1  Taiwan n=1  Argentina n=1  Chile n=1  Germany n=1 | Mean GWG |
| He *et al.* 2023 | To assess the impact of physical activity intensity, dose, period, and duration on perinatal depression, comprehensively evaluating the impact of physical activity on perinatal women | Databases: PubMed, Embase, Cochrane Library, Web of Science  Supplementary searches: none | - Perinatal women - Physical activity intervention compared to usual care/other therapies that do not involve physical activity - Reporting depression and depression severity - Controlled trials - English | - Animal/, in-vitro - Incomplete or valid data - Conference reports, case reports, meta-analyses, reviews, editorial materials, letters, and protocols. | - 35 studies - Published 2003-2021 - Study design CT n=35 - n=5,084 women | Spain n=6  China n=5  Australia n=4  USA n=8  Turkey n=1  Brazil n=1  UK n=3  Egypt n=1  Iran n=1  India n=1  Japan n=1  Norway n=1  Colombia n=1 | Edinburgh perinatal depression score/ CES-D I perinatal and post-partum period.  HADS score |
| Hill *et al.* 2013 | To evaluate the overall effectiveness of GWG interventions derived from theories of behaviour change using a generalized health psychology perspective, and assessing the behaviour change techniques reported in the interventions | Databases: CINAHL, Global Health, Health Source (nursing/academic), Medline, Psychology and Behavioral Sciences Collection, and PsycINFO  Supplementary searches: Eligible papers and similar reviews reference lists | - Papers that reported studies in which excessive GWG was the primary or secondary focus of the intervention, and they reported total GWG or adherence to GWG recommendations. - No limits on pre-pregnancy BMI, socioeconomic status, ethnicity, parity, or gestational duration were set | - Studies including adolescent pregnancies - Interventions designed to prevent gestational diabetes were excluded - Interventions focused on smoking cessation and exercise/physical activity for any other purpose than to limit GWG | - 21 studies - Published: 2002-2012 - Study design: RCT n=14; Case control n=1; Controlled trial n=2; Prospective intervention n=2; Cohort n=2 - n=3,823 women | USA n=6 Nordic n=6 Canada n=3 Brazil n=2 Belgium n=2 Australia n=1 Taiwan n=1 | GWG or adherence to GWG guidelines |
| Hussain *et al.* 2020^a^ | To systematically evaluate  mHealth interventions used in pregnancy in high-income countries and their effects on maternal health behaviors and maternal-fetal health outcomes | Databases: MEDLINE via PubMed, EMBASE, Web of Science, Cochrane Database of Controlled Trials, CINAHL, and PsycINFO  Supplementary Searches: Hand searches of bibliographies | - English-language - pregnant women who utilized pregnancy-related mobile phone interventions - developed or high-income countries - stand-alone mobile phone–based interventions | - Technology interventions aimed solely at communication between patients and clinicians without educational, motivational, or interactive component - Interventions not primarily intended for mobile phone use, eg, websites. - Focused solely on neonatal health (e.g., feeding support or growth tracking). - Published abstracts or conference proceedings - Feasibility or desirability of hypothetical interventions - Outcomes with fewer than 2 weeks of intervention use. | - 9 studies - Published: 2012 to 2018 - Study design: RCT n=7; observational n=2 - n=1,908 women | USA n=5  Japan n=1  UK n=3 | GWG, birth weight, small or large for gestational age, GDM |
| James *et al.* 2020 | To conduct a systematic review of literature on the efficacy of interventions to improve PA among pregnant women | Databases: Pubmed/Medline  Supplementary searches: Manual searches of the reference lists | - RCTs - Intervention designed to promote PA during pregnancy. - The impact of the intervention on PA was either a primary or secondary endpoint. | - Non-RCTs - post-partum period - evaluated level of physical performance to be attained - No control group. | - 15 studies - Published: 2008 and 2019 - Study design: RCT n=15 - n=5633 participants | USA = 5  Australia =4  Finland =3  Canada =1  China =1  Ireland =1 | Behavioural outcomes only.  (change in physical activity levels) |
| Jorge *et al.* 2015 | To evaluate the available evidence on the effectiveness of group exercise programs in improving women’s health outcomes during pregnancy, as well as newborn’s health outcomes and to assess the content of the programs. | Databases: PubMed, Scopus and Scielo.  Supplementary searches: reference lists | - adult pregnant women - no medical or obstetric - BMI in range of normal; - interventions minimum of 12 weeks, group exercise designed to maintain or increase physical activity during pregnancy - targeting maternal and fetal health outcomes; - comparison with no intervention - outcome related to health, PA, quality of life - RCTs and CCTs - articles published in the English, Portuguese and Spanish languages; - published since 2009 | - pregnant women with diagnosed type 1 or type 2 diabetes at - recruitment or other pregnancy-related medical conditions; - interventions specifically designed for underweight, overweight - or obese pregnant women or other pregnancy-related medical - conditions; inclusion of teenagers; unpublished data, theses, - books and congress abstracts | - 17 studies Published: 2009 to 2014 - Study design: RCTs n=17 - n=2,569 women | Brazil n=1  USA n=1  Spain n= 8  Norway n=7 | maternal perception of state of health, cardiorespiratory fitness, GWG, GDM, hypertension, preeclampsia, anaemia, changes in haematological variables, disability, lumbopelvic & pelvic girdle pain, urinary incontinence, flatus, anal incontinence, preterm delivery, gestational age, type of delivery, lacerations, duration of labor, uteroplacental blood flow, placenta weight, route of delivery, birth weight and length, fetal growth, macrosomia, head circumference, umbilical cord pH |
| Leonard *et al.* 2021 | To examine the effect of technology-supported interventions on prenatal GWG, physical activity and diet. | Database: PubMed (MEDLINE), Web of Science, and CINAHL  (EBSCOhost).  Supplementary searches: hand searches from references lists of included articles | - RCTs - technology-supported interventions - Standalone or additional to face to face - Control consisted of standard care or other delivery not including technology - GWG, physical activity or eating behaviours as an outcome post-intervention | - Only reported GWG, physical activity, or healthy eating as secondary outcomes - Focus on clinicians as participants rather than pregnant women - RCT protocol studies - Only qualitative data - Only reported long-term follow-up (e.g., post partum) | - 21 studies - Published: 2006 to 2019 - Study design: 48% pilot studies, 95% two-arm RCT - n=6,265 women | Not reported | GWG; physical activity and healthy eating. |
| Liu *et al.* 2023 | Examine the effect of exercise intervention for antenatal depression using meta- analysis and to propose the best exercise intervention program. | Databases: Chinese National Knowledge Infrastructure (CNKI), Wanfang Data, VIP Database, foreign language search included Cochrane Library, SCI, PubMed, Medline, and Embase    Supplementary searches: none reported | - RCTs - Singleton pregnancy, met criteria for depression - Exercise intervention | - Depressiong due to alcoholism /psychoactive substance addiction/ohysical illness - No outcome information - Not published in Chinese or English - Unpublished papers - Animal studies | - 17 studies - Published: 2012-2021 - Study design: n=17 RCTs - n=1,463 women | Not reported | Antenatal maternal depression |
| Makaruk *et al.* 2019 | To compare and analyse regular prenatal exercise programs and examine  their effectiveness in the prevention of GDM. | Databases: EBSCOhost–Academic Search  Complete, Health Source–Consumer Edition, Health  Source-Nursing/Academic Edition, Master File Premier,  MEDLINE, and SportDiscus  Supplementary searches: none reported. | - RCTs or control trials - regular exercise programme led by a pregnancy exercise specialist; - defined the type, intensity, duration, and frequency of exercises. - Healthy pregnant women, without contraindications to exercise/GDM diagnosis. - Report neonatal and maternal outcomes - The exercise program is the only intervention | - Not reported | - 10 studies - Published: 2012 to 2017 - Study design: not reported (only RCTs and controlled trials included) - n=3,770 women | Norway n=2  Spain n=5  Brazil n=1  Netherlands n=1  China n=1 | GDM |
| McDonald *et al.* 2016 | To systematically review the current literature and examine the exercise dose  prescribed in interventions during pregnancy and its influence on  GWG | Databases: Pubmed, Web of Science, CINAHL, Academic Search Premiere  Supplementary searches: references of obtained articles | - Inclusion of any control condition - GWG measured and reported - Inclusion of any intervention with exercise as the primary focus - Complete description of the exercise dose - multiple intervention arms - a sufficient number of experimental/control conditions must have been utilized in order to examine the independent effect of exercise on GWG. | - Not reported | - 21 studies - Published: 1983 to 2014 - Study design: RCT n=18; Quasi n=3 - n=2,942 women | USA n=6 Canada n=2  Brazil n=3  Norway n=2  Spain n=3 Netherlands n=1  Iran n=2 Australia n=1  New Zealand n=1 | GWG |
| Muktabhant *et al.* 2015 | To determine whether diet or exercise measures, or both, could prevent excessive gestational weight gain, and if they were safe | Databases: Cochrane Pregnancy and Childbirth Group’s Trials Register  (includes CENTRAL, MEDLINE, Embase)    Supplementary searches: Hand searched 30 journals and conference proceedings; weekly alerts for 44 journals; BioMed Central alerts; contacted investigators.  Reference lists of retrieved studies. | - Pregnant women of any BMI - RCTs | - Not reported | - 65 studies - Published: 1990-2014 - Study design: RCT n=65 - n=>13, 523 women (2 studies did not report number of participants) - 49 RCTs (n=11,444 women) included in meta-analysis | Australia n=10  Belgium n=2 Canada n=5 Denmark n=3 Finland n=3 Germany n=1 Ireland n=1  Italy n=3  Norway n=1 Sweden n=2 Netherlands n=2  Spain n=3  UK n=1  USA n=20  Brazil n=4  Columbia n=1  Taiwan n=1 | **Mother:** GWG; Low weight gain; Preterm birth; PPROM; Pre-eclampsia/ eclampsIa; Hypertension. Induction; Caesarean; Postpartum complication (haemorrhage, wound infection, endometritis, antibiotics, perineal trauma, thromboembolic disease, maternal death); diet, physical activity. **Newborns:** Birthweight; macrosomia; <2500 g or <10th centile; hypoglycaemia, hyperbilirubinaemia, infant birth trauma, respiratory distress syndrome. **Long-term health outcomes:** PPWR; childhood weight |
| O’Brien *et al.* 2014 | To systematically review the literature examining technology-supported lifestyle interventions among healthy pregnant women, and to determine the potential impact that such interventions could have on modern antenatal care | Databases: PubMed, MEDLINE, CINAHL, EMBASE, Cochrane Database, controlled-trials.com clinicaltrials.gov    Supplementary searches: Journals. References of included manuscripts. | - English language articles - Healthy pregnant women - RCTs, cross-sectional observational studies, feasibility studies and ongoing trials were included | - Diagnosed with GDM prior to intervention. | - 7 studies - Published: 2009 - 2013 - Study design: RCT n=5, observational n=2 - n=4,500 women (RCTs) n=15,328 women (observational) | USA n=4  Netherlands n=2  Australia n=1 | Fasting maternal glucose; GWG; incidence of GDM; dietary or physical activity modifications |
| O’Connor *et al.* 2025 | To summarise the effectiveness of dietary  interventions for pregnant woman, identify which BCTs and intervention features were most frequently used in these interventions and determine which were most effective at improving dietary intake. | Databases: MEDLINE, CINAHL, EMBASE, Psychinfo, CENTRAL, Cochrane Library, Web of Science  Supplementary searches:  None reported | - Pregnant women over 16 years of age - Interventions including one or more BCTs aimed at improving dietary intake - Usual care or a less intensive intervention control - Published, peer reviewed RCTs, cluster RCTs and pilot RCTs | - Pregnant women in specialist care for a medical diagnosis including physical and mental health conditions and women experiencing pregnancy complications - Interventions claiming to change dietary intake without describing specific BCTs or with insufficient detail provided for BCT coding - No reported control | - 37 studies - Published: 2010-2022 - Study design: n=5 pilot RCTs, n=28 RCTs, n=4 cluster RCTs - N=17,300 | Belgium n=1  USA n=8  Canada n=1  Australia n=6  Finland n=3  UK n=5  China n=2  Turkey n=1  Norway n=1  Spain n=3  Italy n=1  Europe n=1  Iran n=1  Germany n=1  France n=1  Sweden n=1 | Between group change in the following dietary measures: fruits, vegetables, grains and cereals, meat and alternatives, dairy, discretionary foods and dietary quality measure scores. GWG (according to pre-pregnancy body mass index (BMI)), GDM diagnosis and maintenance of dietary change post pregnancy |
| Perales *et al.* 2016 | To identify the benefits of each of aerobic, resistance or combined (aerobic + resistance) exercise modalities, as well as exercise counselling, on maternal health and on perinatal outcomes. | Databases: MEDLINE    Supplementary searches: None reported | - RCT (excluding protocol/methodological papers) - Evaluating the effect of exercise performed only during pregnancy on maternal health and/or perinatal outcomes - Conducted with healthy pregnant women - Written in English. | - Not reported | - 61 studies - Published: not reported - Study design: RCT n=61 - n women: not reported | Not reported | **Maternal health:** GWG; Cardiorespiratory fitness; Muscle strength; Urinary incontinence;  GDM; Caesarean;  Duration of labour; Lumbopelvic pain; Blood pressure. **Perinatal outcomes**  Birth weight;  Macrosomia |
| Poyatos-Leon *et al.* 2015 | To determine the influence of physical exercise interventions on the mode of delivery | Databases: Cochrane Library Plus, Science Direct, EMBASE, PubMed, Web of Science and ClinicalTrials.gov  Supplementary searches: None reported | - RCTs in which the control group received no physical exercise - Aim to assess the influence of PA on pregnant women - Population: healthy women or women with low to moderate levels of PA and with uncomplicated and singleton pregnancies - Intervention was supervised exercise program - Delivery outcomes assessed | - Not published in English or Spanish - Women at high risk of preterm delivery - Participating in other exercise programs during pregnancy - Contraindication for exercise - Not planning to give birth in the same hospital obstetrics department in which the antenatal care during the pregnancy was provided | - 10 studies - Published: 1990 to 2013 - Study design: RCT n=10 - n=3,160 women | Spain n=7  Norway n=1  Brazil n=1  USA n=1 | Mode of delivery (i.e. normal, operative and caesarean) |
| Rhodes *et al.* 2020 | To  determine the effectiveness of exclusively digital diet and  physical activity interventions to improve lifestyle behaviors  or avoid excessive weight gain during pregnancy. | Databases: MEDLINE, PsycINFO, EMBASE,  CINAHL, Web of Science, and ProQuest Dissertations  and Theses  Supplementary searches: Cochrane Library and reference lists of  eligible studies. | - Pregnant women >18 years, of all BMIs. - Digital interventions targeting dietary or physical activity in pregnancy - Exclusively digital and used text messages, apps or websites. - Comparators were usual antenatal care, minimal interventions, or non-diet or physical activity interventions. - Studies measuring GWG, dietary, physical activity. - RCTs and pilot RCTs. | - Pregnant women with physical or mental health issues that would preclude them from participating in a diet- or physical activity–based intervention - Interventions aimed at increasing GWG - Interventions using interpersonal coaching. | - 11 studies included in the systematic review and 6 studies included in the meta-analysis. - Published: 2012 to 2018 - Study design: randomised pilot or feasibility studies n=7, RCTs n=4 - n=3,280 women | USA n= 9  Australia n= 2 | GWG |
| Sanabria-Martin *et al.* 2015 | To conduct a meta-analysis of randomised controlled trials for assessing the effectiveness of physical exercise interventions during pregnancy to prevent gestational diabetes mellitus and excessive maternal weight gain | Databases: Cochrane Library Plus, Science Direct, EMBASE, PubMed, Web of Science, ClinicalTrials.gov    Supplementary searches: Manual search of the references of published studies | - RCTs that aimed to assess the influence of PA on pregnant women - Control group received no exercise - Sedentary healthy women or with low levels of PA - Uncomplicated and singleton pregnancies - Physical exercise programmes included low to moderate intensity exercises - Pregnancy outcomes: GDM and/or GWG - Published 1990 to 2014 - Spanish or English | - Women at high risk of preterm delivery - Women involved in any other trial - Women with any contraindication for exercise - Women who were not planning to give birth at the same obstetrics hospital department in which the medical control of the pregnancy was performed | - 14 studies - Published: 1990 to 2013 - Study design: RCT n=14 - n=2,873 women | Spain n=7  Norway n=1  Brazil n=1  USA n=2  Croatia n=2  New Zealand n=1 | Risk of GDM, GWG |
| Sheffield & Woods-Giscombe 2016 | To examine existing empirical literature on yoga and its effects on women’s health and well-being during the perinatal period. | Databases: CINAHL, PubMed, PsycINFO, and EMBASE.  Supplementary searches: Hand searches of the reference lists. | - Perinatal women, all ages - English language - full-text intervention studies - all years up to 2013. | - Exclusion criteria included any form of CAM that did not include yoga as part of the intervention - Dissertations, textbooks, editorials, literature reviews - Studies that focused specifically on labour or perinatal outcomes | - 13 studies - Published: 2009-2013 - Study design: RCT n=7, one group pre/post-test n=1, prospective two group pre/post-test n=2, qualitative study n=1, quasi experimental one group pre/post-test n=1, correlation one group pre/post assessment survey design n=1. - n=667 women | Not reported | Depression; Anxiety; Stress; Pain; maternal fetal attachment; Sleep;  Relationships; Biomarkers (Cortisol, Progesterone, Estriol);  Anger; Fatigue; Heart rate variability; Birth outcomes; Mindfulness |
| Sherifali *et al.* 2017 | The primary objective of this systematic review was to assess the effectiveness of eHealth technologies for managing weight (loss, gain, or maintenance) during pregnancy and the postpartum period. The secondary objectives were to assess the effectiveness of eHealth technologies on other clinical outcomes, including (1) glycemic parameters and (2) health behaviors (ie, nutrition and physical activity). | Databases:  MEDLINE, EMBASE, Cochrane database of systematic reviews (CDSR), Cochrane central, register of controlled trials (CENTRAL), CINAHL (Cumulative Index to Nursing and Allied Health Literature), and PsycINFO.  Supplementary searches: Reference lists and associated paper citations | - Study design: All relevant primary studies that involved RCTs, non-RCTs such as clinical CCTs, pre-post studies, historically controlled studies, and pilots. - Adult women ≥18 years during pregnancy or the postpartum period. - Intervention included nutrition or physical activity in the eHealth technology, with a specific goal of targeting either GWG during pregnancy or weight loss during the postpartum period. - eHealth technologies included: mobile phone, Web-based, email, personal digital assistant, handheld computer, home computer, or tablet app. - minimum intervention duration of 3 months. - comparators: (1) in-person interventions, (2) other health technology interventions, and (3) no intervention | - Protocols without preliminary results for data extraction. - All other study designs not part of the inclusion criteria | - 6 studies - Published: 2010-2016 - Study design: RCTs n=5, CCTs n=1 - n=363 women (only including interventions during pregnancy) | UK n= 1  Spain n=2  USA n= 3  (only including interventions during pregnancy) | GWG, weight loss postpartum; changes in glycemic status, nutritional measures (eg, total energy intake), and physical activity (eg, minutes of physical activity). |
| Skouteris *et al.* 2014 | To systematically review published controlled trials evaluating behaviour modification interventions to prevent the development of GDM. | Databases: Academic Search Complete, CINAHL, Global Health, PsycInfo, and MEDLINE  Supplementary searches: review of reference lists of sentinel papers. | - the incidence, prevalence, or treatment of GDM was the primary or secondary outcome, irrespective of study design. - No limits on the method in which the intervention was administered (eg, via telephone, internet, in person) | - preliminary studies such as feasibility studies, pilot studies - grey literature (nonpeer reviewed or without scientific credibility). | - 9 studies - Published: 2002 to 2012 - Study design: n=8 RCT, n=1 prospective matched controlled design - n=2,139 women | Finland n=1  Norway n=1  Australia n=2  USA n=4  Canada n=1 | GDM, GWG |
| Soltani *et al.* 2016 | To evaluate the behaviour change techniques included in diet, physical activity, or mixed interventions with a potential to impact on maternal or fetal outcomes related to weight and to identify the categories of behaviour change technique of those interventions which were effective | Databases: Medline, Embase, BIOSIS, LILACS, Science Citation Index, CDSR, CENTRAL, DARE, HTA, PsychInfo  Supplementary searches: Inside Conferences, SIGLE, Dissertation Abstracts, and Clinical Trials.gov. Internet searches with specialist search gateways (such as OMNI: www.omni.ac.uk), general search engines (such as Google), and meta-search engines (such as Copernic: www. copernic.com) | - RCTs that evaluated any dietary or lifestyle interventions with potential to influence maternal and fetal outcomes related to weight. | - Studies on pregnant women who were underweight (BMI <18.5) | - 44 studies - Published: 1976 to 2012 - Study design: 44 randomised control trials - n=7,627 women | Not reported | GWG |
| Tieu *et al.* 2017 | To assess the effects of dietary advice interventions for preventing GDM and associated adverse health outcomes for women and their babies. | Databases: MEDLINE, Embase and CINAHL  Supplementary searches: Cochrane Pregnancy and Childbirth’s Trials Register, CENTRAL, hand searched journals, conference proceedings, journals reviewed via the current awareness service, reference lists of retrieved articles | - RCTs and quasi-RCTs involving pregnant women - Interventions that assessed any type of dietary advice before testing for GDM - Involving pregnant women regardless of age, gestation, parity or plurality. | - Cross-over trials - Trials presented only as abstracts - Studies involving women with pre- existing type 1 or type 2 diabetes Interventions assessing combined dietary advice and exercise interventions | - 11 studies - Published: 1983 to 2016 - Study design: RCTs n=11 - n=2,786 women | USA n=2 UK n=1  Finland n=1 Australia n=4 Brazil n=1  Ireland n=1 Denmark n=1 | **Mother:** GDM, LGA, fasting glucose levels; Caesarean, Operative vaginal birth; Induction; Perineal trauma; Placental abruption; PPH; Postpartum infection; Breastfeeding; GWG; Behaviour change; biomarker changes; well-being and quality of life; Postnatal depression; GDM in a subsequent pregnancy; T1DM; T2DM; IGT; PPWR; BMI; Cardiovascular health; **Child:** Stillbirth; Neonatal mortality; Preterm; Apgar score; Macrosomia; SGA; Shoulder dystocia; Nerve palsy; Bone fracture; Respiratory distress syndrome; Hypoglycaemia; Hyperbilirubinemia; Gestational age; Birthweight; Head circumference; Length; Ponderal index; Adiposity; Weight; Height; Adiposity; Cardiovascular health; Employment, education and social status/achievement; T1DM, T2DM; IGT; Neurosensory disability. |
| Tombers *et al.* 2023 | To deter­mine the effect of exercise on reducing lumbopelvic pain intensity | Databases:  Medline Complete, CINAHL, ScienceDirect, SportDiscus, ProQuest, Google Scholar, and Cochrane Library  Supplementary searches:  Reference lists of relevant review articles and the included studies | - Exercise types could include aerobic, aquatic, resistance training, mobility exercise, core stabilization, or relaxation exercises (supervised or unsupervised) - Pregnant women at least 18 years of age with any number of past pregnancies and carrying any number of fetuses - A single exercise type was used as the intervention | - History of chronic low back or pel­vic pain - Complicated pregnancy - Exercise was combined with other interventions in the treatment group (such as manual therapies or modali­ties) - No control group was used, or the control group had an intervention other than usual daily activity or usual prenatal care. | - 21 studies - Published 1999 to 2021 - Study design: RCT n=19, Pre/Post design n=2 - 2379 participants | Not reported | Pain intensity prior to delivery |
| Turan *et al.* 2025 | To review the effects of Pilates during pregnancy, focusing on type, duration, frequency, practitioners, and parameters it influences | Databases: PubMed, Scopus, Web of Science, PEDro, Cochrane Library, and CINAHL  Supplementary searches: None reported | - Randomized or non-randomized controlled studies - Pilates methods in healthy pregnant women in the 2nd or 3rd trimester of pregnancy - Published in the last 10 years with full text available in English | - Studies involv­ing non-pregnant individuals - Case reports, case series, systematic reviews, meta-analyses, and non-English studies for which full text was not available - Studies without a con­trol group | - 21 studies - Published 2004 to 2024 - Study design: RCT/RT n=14, Pre/post design n=1, quasi-experimental/experimental n=5,Case control study n=1 - 1245 individuals | Turkey n=6  South Korea n=3  Brazil n=2  Cyprus n=2  Iran n=1  Italy n=1  Pakistan n=1  Spain n=1  Slovenia n=1  Indonesia n=1  India n=1  Austrailia n=1 | All outcomes eligible but subgroups of pain, sleep, depression, and quality of life |
| Vincze *et al.* 2019 | To evaluate the effectiveness of weight management interventions which included a diet component aimed at limiting gestational weight gain. | Databases: MEDLINE, Embase, CINAHL, Scopus, PsycINFO  Supplementary searches: Cochrane Central, reference lists | - Pregnant women aged 18 years or over - All pre-pregnancy BMI categories. - Participants had GDM or diabetes - Intervention with dietary component aimed at managing GWG, compared to usual care or ‘‘other’’ - Weight related primary outcome measured during pregnancy - RCTs published between 1980 and January 21, 2016. | - Any other medical conditions - Unpublished results | - 20 studies - Published: 2002 to 2016 - Study design: RCT n=20 - n=5,895 women | USA n=6  Australia n=2 Canada n=2 Denmark n=3 Finland n=1  Italy n=2  Sweden n=1 Turkey n=1 Belgium n=1 Germany n=1 | GWG |
| Walker *et al.* 2018 | To address compare the efficacy of all approaches that have been used to prevent excessive GWG (e.g., diet, PA, lifestyle, sleep, eHealth, and medical) | Databases: MEDLINE, EMBASE, Cochrane Library, PsycInFO, CINAHL, Scopus, LILACS  Supplementary searches: reference lists of included trials and systematic reviews | - RCTs of interventions designed to prevent excessive GWG. - Comparators were standard care, an alternate intervention, or placebo. - Participants were women of all ages, ethnicities, and pre-pregnancy weight status with singleton pregnancies. | - Studies for women with pre-existing diabetes (type 1 or type 2) or GDM | - 89 studies - Published: 2000 to 2018 - Study design: RCT n=89 - n=25,345 women | USA n=22 Australia n=11  Spain n=10 Italy n=5 Finland n=3 Egypt n=1 Turkey n=2 Sweden n=2 Brazil n=4 Ireland n=2 Denmark n=3 | GWG |
| Wang *et al.* 2019 | To collect and reassess all the evidence regarding whether physical exercise and its characteristics can have an effect on maternal GWG. | Databases: Pubmed, Embase and Cochrane Library  Supplementary searches: Hand search included trials bibliographies | - RCTs in pregnant women - PA such as aerobic exercises, strength training, walking, cycling, weight training - compared with conventional medical care | - Non RCT studies | - 23 studies - Published: 1999 to 2017 - Study design: RCT n=23 - n=4,462 women | China n=1 Brazil n=2 Spain n=9 Japan n=1 USA n=4 Norway n=1 New Zealand n=1  Columbia n=1  Iran n=2  Sweden n=1 | GWG |
| Yin *et al.* 2013 | To collect all the evidence available from RCTs regarding the association between physical exercise during pregnancy and the incidence of GDM. | Databases: PUBMED, ISI Web of Knowledge, CBM, CNKI, VIP    Supplementary searches: Cochrane CENTRAL Library, reference lists | - RCT Pregnant women without previous - GDM and other complications. Increased physical activity was the only - intervention. Incidence of GDM was documented - separately for the control and intervention groups, with diagnosis criteria as defined in individual trials. | - Not reported | - 6 studies - Published: 2009 to 2012 - Study design: RCT n=6 - n=1,278 women | USA n=1  Norway n=1  Australia n=1  Spain n=2  India n=1 | GDM |
| Zenitasari et al. 2025 | To evaluate the effects of Pilates training on labor pain and the duration of labor. | Databases: PubMed, Science Direct, Scopus  Supplementary searches: Reference lists of included studies | - Pregnant Women - Pilates training intervention - Control group of women who did not do Pilates during pregnancy - RCT and Cohort published in Indonesian and English - constituted the exclusion criteria of the study. | Quasi-experimental studies, reviews, case reports, qualitative studies, unpublished theses, congress papers, and descriptive studies | - 5 studies - Published 2021-2023 - Study design: RCT n =3, Cohort study n=2 - N=409 participants | Not reported | Labor pain and duration of labor |
| Zhang *et al.*  2023 | To evaluate the effects of aerobic exercise performed during pregnancy on GDM, GH, and pregnancy outcomes. | Databases:  PubMed, EMbase, Web of Science, China Knowledge Network, Wanfang, Weipu  Supplementary searches: None reported | - RCTs with blinding not restricted - Pregnant women without cardiovascular, liver, or kidney disease, as well as reproductive system or other basic diseases - Intervention was aerobic exercise with no restriction on type of exercise - Control group was conventional care - Baseline is comparable | - Non-randomized controlled trials - Subjects with a history of psychiatric disorders, diabetes, hypertension, and other underlying diseases - Incomplete primary data - Full text not available, abstract only, duplicate publications, conference papers | - 11 studies - Published 2014 to 2022 - Study design: RCT n=11 - N=3165 women | China n=4  Italy n=1  Croatia n=1  India n=2  Spain n=2  Brazil n=1 | GDM, GH, and pregnancy outcomes |

a. Review by Hussain et al 2020 also reported data for diet/physical activity interventions.
