# Supplementary material for: Features of smoking cessation, alcohol reduction, and diet and physical activity interventions associated with changing behaviours during pregnancy: a systematic review of reviews
Source: J Behav Med. 2026 Aug 6;49(6):985–1001. doi: 10.1007/s10865-026-00702-1 (PMC13748204; doi:10.1007/s10865-026-00702-1)
Supplement: Supplementary file 4 [file 10865_2026_702_MOESM4_ESM.docx]

**Supplement 4 Critical appraisal results for systematic reviews reporting outcomes for each behavioural domain, and overall quality for all included systematic reviews**

**Supplement 4a: Systematic reviews reporting the effectiveness of alcohol interventions**

| Author, year | Quality assessment question ^1^ | | | | | | | | | | | Total score ^2^ | Quality ^3^ |
| --- | --- | --- | --- | --- | --- | --- | --- | --- | --- | --- | --- | --- | --- |
|  | 1 | 2 | 3 | 4 | 5 | 6 | 7 | 8 | 9 | 10 | 11 |  |  |
| Froschl *et al.* 2013 | Yes | No | Unclear | Unclear | Unclear | Unclear | Unclear | Unclear | Unclear | Yes | Yes | 3 | Low |
| Gomez *et al.* 2020 | Yes | Yes | Yes | No | Yes | Yes | Yes | Yes | No | Yes | Yes | 9 | High |
| Minozzi et al. 2024 | Yes | Yes | Yes | Yes | Yes | Yes | Yes | Yes | Yes | Yes | Yes | 11 | High |
| Popova *et al.* 2023 | Yes | Yes | Yes | Yes | Yes | Yes | Yes | Yes | Yes | Yes | Yes | 11 | High |
| Oh *et al.* 2022 | Yes | Yes | Yes | Yes | Yes | Unclear | Yes | Yes | Yes | Yes | Yes | 10 | High |
| Samawi *et al.* 2021 | Yes | Yes | Yes | Yes | Yes | Unclear | No | No | No | Yes | Yes | 7 | Moderate |
| **Subtotal**  **(yes/total number of studies)** | 6/6  100% | 5/6 83% | 5/6  83% | 5/6  83% | 5/6  83% | 3/6  50% | 4/6  66% | 4/6  66% | 3/6  50% | 6/6  100% | 6/6  100% | Range 3-11 | 17% Low (1/6)  17% Low (1/6)  66% High (4/6) |

**Supplement 4b: Systematic reviews reporting the effectiveness of smoking cessation interventions**

| **Author, year** | **Quality assessment question ^1^** | | | | | | | | | | | **Total Score^2^** | **Quality^3^** | |  |
| --- | --- | --- | --- | --- | --- | --- | --- | --- | --- | --- | --- | --- | --- | --- | --- |
|  | 1 | 2 | 3 | 4 | 5 | 6 | 7 | 8 | 9 | 10 | 11 |  | |  | |
| Acar *et al.* 2022 | Yes | Yes | Yes | No | Unclear | Unclear | Unclear | Yes | No | Yes | Yes | 6 | | Moderate | |
| Brown *et al.* 2019 | Yes | Yes | Yes | No | Yes | Yes | Yes | Yes | Yes | Yes | Yes | 10 | | High | |
| Chamberlain *et al.* 2013 | Yes | Yes | Yes | Yes | Yes | Unclear | Yes | Yes | Yes | Yes | Yes | 10 | | High | |
| Chamberlain *et al.* 2017 | Yes | Yes | Yes | Yes | Unclear | Yes | Yes | Yes | Yes | Yes | Yes | 10 | | High | |
| Griffiths *et al.* 2018 | Yes | Yes | Yes | Yes | Yes | Yes | Yes | Yes | Yes | Yes | Yes | 11 | | High | |
| Heminger *et al.* 2016 | Yes | Yes | Yes | Yes | Unclear | Unclear | No | Yes | No | Yes | Yes | 7 | | Moderate | |
| Hussain *et al*. 2020 | Yes | Yes | Yes | Yes | Yes | Yes | Yes | Yes | No | Yes | Yes | 10 | | High | |
| Su & Buttenheim 2014 | Yes | Yes | Yes | Yes | Yes | Yes | Yes | Yes | No | Yes | Yes | 10 | | High | |
| Tahan et al 2023 | Yes | Yes | Yes | Yes | Yes | No | Yes | Yes | Yes | Yes | Yes | 10 | | High | |
| Wilson *et al.* 2018 | Yes | Yes | Yes | No | Yes | Yes | Yes | Yes | No | Yes | Yes | 9 | | High | |
| **Subtotal**  **(yes/total number of studies)** | 10/10  100% | 10/10  100% | 10/10  100% | 7/10  70% | 7/10  70% | 6/10  60% | 8/10  80% | 10/10  100% | 5/10  50% | 10/10  100% | 10/10  100% | Range 6-11 | | 20% Moderate (2/10)  80% High (8/10) | |

**Supplement 4c: Systematic reviews reporting the effectiveness of diet and/or physical activity interventions**

| **Author, year** | **Quality assessment question ^1^** | | | | | | | | | | | **Total Score^2^** | **Quality^3^** |
| --- | --- | --- | --- | --- | --- | --- | --- | --- | --- | --- | --- | --- | --- |
|  | **1** | **2** | **3** | **4** | **5** | **6** | **7** | **8** | **9** | **10** | **11** |  |  |
| Agha *et al.* 2014 | Yes | Yes | Yes | Yes | Yes | Yes | Yes | Yes | Yes | Yes | Yes | 11 | High |
| Allen *et al.* 2014 | Yes | Yes | Yes | No | Yes | Yes | No | Yes | No | Yes | Yes | 7 | Moderate |
| Baradwan et al., 2024 | Yes | Yes | Yes | Yes | Yes | Yes | Yes | Yes | Yes | Yes | Yes | 11 | High |
| Beetham *et al.* 2019 | Yes | Yes | Yes | Unclear | No | Unclear | Yes | Yes | Yes | No | Yes | 7 | Moderate |
| Bennett *et al.* 2023 | Yes | Yes | Yes | Yes | Yes | Unclear | Yes | Yes | Yes | Yes | Yes | 10 | High |
| Cancela-Carral *et al.* 2022 | Yes | Yes | Yes | Yes | Yes | Yes | Yes | Yes | No | Yes | No | 9 | High |
| Cantor *et al.* 2021 | Yes | Yes | Yes | Yes | Yes | Yes | Yes | Yes | No | Yes | Yes | 10 | High |
| Chan *et al.* 2019 | Yes | Yes | Yes | No | Yes | No | Yes | Yes | No | Yes | Yes | 8 | High |
| Craemer *et al.* 2019 | Yes | No | Yes | No | No | No | No | Yes | Yes | Yes | Yes | 6 | Moderate |
| Currie *et al.* 2013 | Yes | Yes | Yes | Yes | Yes | Unclear | Unclear | No | No | Yes | Yes | 7 | Moderate |
| Davenport *et al.* 2018a | Yes | Yes | Yes | Yes | Yes | No | Yes | Yes | Yes | Yes | No | 9 | High |
| Davenport *et al.* 2018b | Yes | Yes | Yes | No | Yes | No | Yes | Yes | Yes | Yes | Yes | 9 | High |
| Davenport *et al.* 2018c | Yes | No | No | Yes | Yes | Yes | Yes | Yes | Unclear | Yes | Yes | 8 | High |
| Davenport *et al.* 2019a | Yes | Yes | Yes | Yes | Yes | No | Yes | Yes | No | No | Yes | 8 | High |
| Davenport *et al.* 2019b | Yes | Yes | Yes | No | Yes | Unclear | Yes | Yes | No | No | Yes | 8 | High |
| Di Mascio *et al.* 2016 | Yes | Yes | Yes | No | Yes | Unclear | Unclear | Yes | Yes | Yes | No | 7 | Moderate |
| Gresham *et al.* 2014 | Yes | Yes | Yes | Yes | Yes | Yes | Yes | Yes | Yes | Yes | Yes | 11 | High |
| Gresham *et al.* 2016 | Yes | Yes | Yes | Yes | Yes | Yes | Yes | Yes | Yes | Yes | Yes | 11 | High |
| Guillemette *et al.* 2018 | Yes | No | Yes | Yes | No | Yes | Yes | Yes | Yes | Unclear | No | 7 | Moderate |
| Guo *et al.* 2019 | Yes | Yes | Yes | No | Yes | Yes | Yes | Yes | Yes | Yes | No | 9 | High |
| Hanifi *et al.* 2023 | Yes | Yes | Yes | Yes | Yes | Yes | Yes | Yes | Yes | Yes | Yes | 11 | High |
| Harrison et al. 2023 | Yes | Yes | Yes | Yes | No | No | Unclear | Yes | Unclear | No | Yes | 6 | Moderate |
| He *et al.* 2023 | Yes | Yes | Yes | No | Yes | Unclear | Unclear | Yes | Yes | Yes | Yes | 8 | High |
| Hill *et al.* 2013 | Yes | Yes | Yes | Yes | Yes | Yes | Unclear | Yes | No | Yes | Yes | 9 | High |
| Hussain *et al.* 2020 | Yes | Yes | Yes | Yes | Yes | Yes | Yes | Yes | No | Yes | Yes | 9 | High |
| James *et al.* 2020 | Yes | Yes | Yes | No | No | No | Unclear | Yes | Unclear | Yes | Unclear | 6 | Moderate |
| Jorge *et al.* 2015 | Yes | Yes | Yes | Yes | Yes | Yes | Yes | Unclear | No | Yes | Yes | 9 | High |
| Leonard *et al.* 2021 | Yes | Yes | Yes | Yes | Yes | Unclear | Yes | Yes | Yes | Yes | Yes | 10 | High |
| Liu *et al.* 2023 | Yes | Yes | Yes | Yes | Yes | No | Yes | Yes | Yes | Yes | No | 8 | High |
| Makaruk *et al.* 2019 | No | Yes | Yes | Yes | No | No | No | No | No | Yes | Yes | 7 | Moderate |
| McDonald *et al.* 2016 | Yes | Yes | Yes | Yes | Yes | Unclear | Unclear | Unclear | No | Yes | Yes | 7 | Moderate |
| Muktabhant *et al.* 2015 | Yes | Yes | Yes | Yes | Yes | Yes | Yes | Yes | Yes | Yes | Yes | 11 | High |
| O’Brien *et al.* 2014 | Yes | No | Yes | Yes | Unclear | Unclear | Unclear | Yes | No | Yes | Yes | 6 | Moderate |
| O’Connor *et al* 2025 | Yes | Yes | Yes | Yes | Yes | Yes | Yes | Yes | No | Yes | Yes | 10 | High |
| Perales *et al.* 2016 | Yes | Yes | Yes | No | Yes | Unclear | Unclear | Yes | Unclear | Yes | Yes | 7 | Moderate |
| Poyatos-Leon *et al.* 2015 | Yes | Yes | Yes | Unclear | Yes | Yes | No | Yes | Yes | Yes | Yes | 9 | High |
| Rhodes *et al.* 2020 | Yes | Yes | Yes | Yes | Yes | Yes | Yes | Yes | No | No | Yes | 9 | High |
| Sanabria-Martin *et al.* 2015 | Yes | Yes | Yes | Yes | Yes | Yes | No | Yes | Yes | Yes | Yes | 10 | High |
| Sheffield & Woods-Giscombe 2016 | Yes | Yes | Yes | Yes | Yes | Unclear | Unclear | Yes | Yes | Yes | Yes | 9 | High |
| Sherifali *et al.* 2017 | Yes | Yes | Yes | Yes | Yes | Unclear | Yes | Yes | No | Yes | Yes | 9 | High |
| Skouteris *et al.* 2014 | Yes | Yes | Yes | Yes | Yes | Unclear | Unclear | Yes | Yes | Yes | Yes | 9 | High |
| Soltani *et al.* 2016 | Yes | Yes | Yes | Yes | Yes | Unclear | Yes | Yes | Yes | Yes | Yes | 10 | High |
| Tieu *et al.* 2017 | Yes | Yes | Yes | Yes | Yes | Yes | Yes | Yes | Yes | Yes | Yes | 11 | High |
| Tombers et al. 2023 | Yes | Yes | Yes | Yes | Yes | Yes | Unclear | Yes | Yes | Yes | Yes | 10 | High |
| Turan et al. 2025 | Yes | Yes | Yes | Yes | Yes | No | Yes | Unclear | No | No | Yes | 7 | Moderate |
| Vincze *et al.* 2019 | Yes | Yes | Yes | Yes | Yes | Yes | Yes | Yes | No | Yes | Yes | 10 | High |
| Walker *et al.* 2018 | Yes | Yes | Yes | Yes | Yes | Yes | Unclear | Yes | Yes | Yes | Yes | 10 | High |
| Wang *et al.* 2019 | Yes | Yes | Yes | Yes | Yes | Yes | Yes | Yes | Yes | No | Yes | 10 | High |
| Yin *et al.* 2013 | Yes | Unclear | Yes | Yes | Yes | Unclear | Yes | Yes | Yes | Yes | Yes | 9 | High |
| Zenitasari et al. 2025 | Yes | Yes | Yes | Yes | Yes | No | Yes | Yes | No | Yes | Yes | 9 | High |
| Zhang et al. 2023 | Yes | Yes | Unclear | Yes | Yes | No | Yes | Yes | No | Yes | Yes | 8 | High |
| **Subtotal**  **(yes/total number of studies)** | 50/5198% | 46/51  90% | 49/5196% | 39/51  76% | 44/51  86% | 24/51  46% | 32/51  62% | 46/51  90% | 27/51  54% | 43/51  84% | 44/51  86% | Range 6-11 | 25% Moderate 13/51  75% High 38/51 |

Notes: 1) Is the review question clearly and explicitly stated?; 2) Were the inclusion criteria appropriate for the review question?; 3) Was the search strategy appropriate?; 4) Were the sources and resources used to search for studies adequate?; 5) Were the criteria for appraising studies appropriate?; 6) Was critical appraisal conducted by two or more reviewers independently?; 7) Were there methods to minimize errors in data extraction?; 8) Were the methods used to combine studies appropriate?; 9) Was the likelihood of publication bias assessed?; 10) Were recommendations for policy and/or practice supported by the reported data?; 11) Were the specific directives for new research appropriate?. For total score 1 is given if yes otherwise it is zero. For quality: Low quality is 0-3. Moderate quality is 4-7. High quality is 8-11.
