# Supplementary material for: Features of smoking cessation, alcohol reduction, and diet and physical activity interventions associated with changing behaviours during pregnancy: a systematic review of reviews
Source: J Behav Med. 2026 Aug 6;49(6):985–1001. doi: 10.1007/s10865-026-00702-1 (PMC13748204; doi:10.1007/s10865-026-00702-1)
Supplement: Supplementary file 5 [file 10865_2026_702_MOESM5_ESM.docx]

**Supplement 5. Overlap of included studies in the systematic reviews**

**Included studies in alcohol reviews**

| **Review** | **References of included alcohol interventions delivered in pregnancy** |
| --- | --- |
| Gomez *et al.* 2020 | 1. Belizán J., Barros F., Langer A., Farnot U., Victora C., Villar J. Impact of health education during pregnancy on behavior and utilization of health resources. Am J Obstet Gynecol 1995; 173: 894–49. 2. Chang G., McNamara T. K., Orav E. J., Koby D., Lavigne A., Ludman B., et al. Brief intervention for prenatal alcohol use: a randomized trial. Obstet Gynecol 2005; 105: 991–8. 3. Chang G., Wilkins-Haug L., Berman S., Goetz M. A. Brief intervention for alcohol use in pregnancy: a randomized trial. Addiction 1999; 94: 1499–508. 4. Crawford-Williams F., Fielder A., Mikocka-Walus A., Esterman A., Steen M. A public health intervention to change knowledge, attitudes and behaviour regarding alcohol consumption in pregnancy. Evid Based Midwif 2016; 14: 4–10. 5. Fleming M. F., Lund M. R., Wilton G., Landry M., Scheets D. The healthy moms study: the efficacy of brief alcohol intervention in postpartum women. Alcohol Clin Exp Res 2008; 32: 1600–6. 6. Gwadz M. V., Leonard N. R., Cleland C. M., Riedel M., Arredondo G. N., Wolfe H., et al. Behavioral interventions for HIV infected and uninfected mothers with problem drinking. Addi Res Theory 2008; 16: 47–65. 7. Handmaker N. S., Miller W. R., Manicke M. Findings of a pilot study of motivational interviewing with pregnant drinkers. J Stud Alcohol 1999; 60: 285–7. 8. Joya X., Mazarico E., Ramis J., Pacifici R., Salat-Batlle J., Mortali C.,et al. Segmental hair analysis to assess effectiveness of single-session motivational intervention to stop ethanol use during pregnancy. Drug Alcohol Depend 2016; 158: 45–51. 9. Marais S., Jordaan E., Viljoen D., Olivier L., de Waal J., Poole C. The effect of brief interventions on the drinking behaviour of pregnant women in a high-risk rural south African community: a cluster randomised trial. Early Child Dev Care 2011; 181: 463–74. 10. O’Connor M. J., Whaley S. E. Brief intervention for alcohol use by pregnant women. Am J Public Health 2007; 97: 252–8. 11. Ondersma S. J., Beatty J. R., Svikis D. S., Strickler R. C., Tzilos G. K., Chang G., et al. Computer-delivered screening and brief intervention for alcohol use in pregnancy: a pilot randomized trial. Alcohol Clin Exp Res 2015; 12. Ondersma S. J., Svikis D. S., Thacker L. R., Beatty J. R., Lockhart N. A randomised trial of a computer-delivered screening and brief intervention for postpartum alcohol use. Drug Alcohol Rev 2016; 35: 710–8. 13. Osterman R. L., Carle A. C., Ammerman R. T., Gates D. Singlesession motivational intervention to decrease alcohol use during pregnancy. J Subst Abuse Treat 2014; 47: 10–9. 14. Osterman R. L., Dyehouse J. Effects of a motivational interviewing intervention to decrease prenatal alcohol use. West J Nurs Res 2012; 34: 434–54. 15. Osterman R., Lewis D., Winhusen T. Efficacy of motivational enhancement therapy to decrease alcohol and illicit-drug use in pregnant substance users reporting baseline alcohol use. J Subst Abuse Treat 2017; 77: 150–5. 16. Reading A. E., Campbell S., Cox D. N., Sledmere C. M. Health beliefs and health-care behavior in pregnancy. Psychol Med 1982; 12: 379–83. 17. Reynolds K. D., Coombs D. W., Lowe J. B., Peterson P. L., Gayoso E. Evaluation of a self-help program to reduce alcohol consumption among pregnant women. Int J Addict 1995; 30: 427–43. 18. Rotheram-Borus M. J., Arfer K. B., Christodoulou J., Comulada W. S., Tubert J. E., Stewart J.,et al. The association of maternal alcohol use and paraprofessional home visiting with children’s health: a randomized controlled trial. J Consult Clin Psychol 2019; 87: 551–62. 19. Rubio D. M., Day N. L., Conigliaro J., Hanusa B. H., Larkby C., McNeil M.,et al. Brief motivational enhancement intervention to prevent or reduce postpartum alcohol use: a single-blinded, randomized controlled effectiveness trial. J Subst Abuse Treat 2014; 46: 382–9. 20. Slesnick N., Erdem G. Efficacy of ecologically-based treatment with substance-abusing homeless mothers: substance use and housing outcomes. J Subst Abuse Treat 2013; 45: 416–25. 21. Tzilos G. K., Sokol R. J., Ondersma S. J. A randomized phase I trial of a brief computer-delivered intervention for alcohol use during pregnancy. J Womens Health (Larchmt) 2011; 20: 1517–24. 22. van der Wulp N. Y., Hoving C., Eijmael K., Candel M. J., van Dalen W., de Vries H. Reducing alcohol use during pregnancy via health counseling by midwives and internet-based computer-tailored feedback: a cluster randomized trial. J Med Internet Res 2014; 16: e274. 23. Waterson E. J., Murray-Lyon I. M. Preventing fetal alcohol effects; a trial of three methods of giving information in the antenatal clinic. Health Educ Res 1990; 5: 53–61. 24. Yonkers K. A., Forray A., Howell H. B., Gotman N., Kershaw T., Rounsaville B. J., et al. Motivational enhancement therapy coupled with cognitive behavioral therapy versus brief advice: a randomized trial for treatment of hazardous substance use in pregnancy and after delivery. Gen Hosp Psychiatry 2012; 34: 439–49. |
| Minozzi *et al.* 2024 | 1. Chang G, Goetz MA, Wilkins-Haug L, Berman S. A brief intervention for prenatal alcohol use: an in-depth look. Journal of Substance Abuse Treatment 2000;18(4):365-9. [DOI: 10.1016/ s0740-5472(99)00105-1] [PMID: 10812310] 2. Chang G, McNamara TK, Orav EJ, Koby D, Lavigne A, Ludman B, Vincitorio NA, Wilkins-Haug L. A brief intervention for prenatal alcohol use: results from a randomized trial of 304 couples. In: 15th Annual Meeting of American Academy of Addiction Research. Vol. 26. 2004. 3. Chang G, Wilkins-Haug L, Berman S, Goetz MA. Brief intervention for alcohol use in pregnancy: a randomized trial. Addiction 1999;94(10):1499-508. [DOI: 10.1046/ j.1360-0443.1999.941014996.x.] [PMID: 10790902] 4. Handmaker NS, Miller WR, Manicke M. Findings of a pilot study of motivational interviewing with pregnant drinkers. Journal of Studies on Alcohol 1999;60(2):285-7. [PMID: 10091968] 5. Joya X, Mazarico E, Ramis J, Pacifici R, Salat-Batlle J, Mortali C, et al. Segmental hair analysis to assess eGectiveness of singlesession motivational intervention to stop ethanol use during pregnancy. Drug and Alcohol Dependence 2016;158:45-51. [DOI: 10.1016/j.drugalcdep.2015.10.028] [PMID: 26589976] 6. O'Connor MJ, Whaley SE. Brief intervention for alcohol use by pregnant women. American Journal of Public Health 2007;97(2):252-8. [DOI: 10.2105/AJPH.2005.077222] [PMID: 17194863 7. Reynolds KD, Coombs DW, Lowe JB, Peterson PL, Gayoso E. Evaluation of a self-help program to reduce alcohol consumption among pregnant women. International Journal of the Addictions 1995;30(4):427-43. [DOI: 10.3109/10826089509048735] [PMID: 7607777] 8. Rubio DM, Day NL, Conigliaro J, Hanusa BH, Larkby C, McNeil M, et al. Brief motivational enhancement intervention to prevent or reduce postpartum alcohol use: a singleblinded, randomized controlled eGectiveness trial. Journal of Substance Abuse Treatment 2014;46(3):382-9. [DOI: 10.1016/ j.jsat.2013.10.009] [PMID: 24315218] 9. Tzilos GK, Ondersma SJ. Assessing feasibility of a brief computer-based intervention for alchol use during pregnancy. In: Alcoholism: Clinical and Experimental Research. Vol. 33. 2009:111A. 10. Tzilos GK, Ondersma SJ. Cognitive functioning and participant ratings of acceptability following computer-delivered brief intervention for alcohol use during pregnancy. In: Alcoholism: Clinical and Experimental Research. Vol. 35. 2011:145A. 11. Tzilos GK, Sokol RJ, Ondersma SJ. A randomized phase I trial of a brief computer-delivered intervention for alcohol use during pregnancy. Journal of Women's Health 2011;20(10):1517-24. [DOI: 10.1089/jwh.2011.2732] [PMID: 21823917] 12. Tzilos GK. A brief computer-based intervention for alcohol use during pregnancy. Graduate School of Wayne State University, Detroit, Michigan 2010. 13. Van Der Wulp NY, Hoving C, Van Dalen W, De Vries H. Preventing prenatal alcohol use via health counselling by midwives and Internet-based computer tailored feedback: a randomized controlled trial. In: Journal of Population Therapeutics and Clinical Pharmacology. Vol. 19(3). 2012:e419-20. 14. van der Wulp NY, Hoving C, Eijmael K, Candel MJ, van Dalen W, De Vries H. Reducing alcohol use during pregnancy via health counseling by midwives and internet-based computer-tailored feedback: a cluster randomized trial. Journal of Medical Internet Research 2014;16(12):e274. [DOI: 10.2196/jmir.3493] [PMID: 25486675] |
| Oh *et al.* 2022 | 1. Evans WD, Wallace Bihm J, Szekely D, Nielsen P, Murray E, Abroms L, et al. Initial outcomes from a 4-week follow-up study of the Text4baby program in the military women's population: randomized controlled trial. J Med Internet Res 2014 May 20;16(5):e131 2. Evans WD, Wallace JL, Snider J. Pilot evaluation of the text4baby mobile health program. BMC Public Health 2012 Nov 26;12:1031 3. Ondersma SJ, Beatty JR, Svikis DS, Strickler RC, Tzilos GK, Chang G, et al. Computer-Delivered Screening and Brief Intervention for Alcohol Use in Pregnancy: A Pilot Randomized Trial. Alcohol Clin Exp Res 2015 Jul;39(7):1219-1226 4. van der Wulp NY, Hoving C, Eijmael K, Candel MJJ, van Dalen W, De Vries H. Reducing alcohol use during pregnancy via health counseling by midwives and internet-based computer-tailored feedback: a cluster randomized trial. J Med Internet Res 2014 Dec 05;16(12):e274 5. Wernette GT, Plegue M, Kahler CW, Sen A, Zlotnick C. A Pilot Randomized Controlled Trial of a Computer-Delivered Brief Intervention for Substance Use and Risky Sex During Pregnancy. J Womens Health (Larchmt) 2018 Jan;27(1):83-92 |
| Popova *et al.* 2023 | 1. Chang G. Personal communication. Email communication received 2008 August. 2. Chang G, Goetz MA, Wilkins-Haug L, Berman S. A brief intervention for prenatal alcohol use: an in-depth look. Journal of Substance Abuse Treatment 2000;18:365–9. 3. Chang G, Wilkins-Haug L, Berman S, Goetz MA. Brief intervention for alcohol use in pregnancy: randomized trial. Addiction 1999;94(10):1499–508. 4. Handmaker N, Miller W, Manicke M. Findings of a pilot study of motivational interviewing with pregnant drinkers. *Journal of Studies on Alcohol* 1999;**60**: 285–7. 5. O’Connor M, Whaley S. Brief intervention for alcohol use by pregnant women. *American Journal of Public Health* 200; **97**(2):252–8. 6. O’Connor MJ. Personal communication. Email communication received 2008 July. 7. Reynolds KD, Coombs DW, Lowe JB, Peterson PL, Gayoso E. Evaluation of a self-help program to reduce alcohol consumption among pregnant women. *International* *Journal of the Addictions* 1995;**30**(4):427–43. |
| Samawi *et al*. 2021 | 1. de Vries MM, Joubert B, Cloete M, Roux S, Baca BA, Hasken JM, et al. Indicated prevention of fetal alcohol Spectrum disorders in South Africa: effectiveness of case management. Int J Environ Res Public Health. 2015;13(1):ijerph13010076.  2. Handmaker NS, Miller WR, Manicke M. Findings of a pilot study of motivational interviewing with pregnant drinkers. J Stud Alcohol. 1999;60(2):285–7.  3. Joya X, Mazarico E, Ramis J, Pacifici R, Salat-Batlle J, Mortali C, et al. Seg- mental hair analysis to assess effectiveness of single-session motivational intervention to stop ethanol use during pregnancy. Drug Alcohol Depend. 2016;158:45–51.  4. Marais S, Jordaan E, Viljoen D, Olivier L, de Waal J, Poole C. The effect of brief interventions on the drinking behaviour of pregnant women in a high-risk rural south African community: a cluster randomised trial. Early Child Dev Care. 2011;181(4):463–74.  5. Meberg A, Halvorsen B, Holter B, Ek IJ, Askeland A, Gaaserud W, et al. Moder- ate alcohol consumption—need for intervention programs in pregnancy? Acta Obstet Gynecol Scand. 1986;65(8):861–4.  6. Moura, AAMD. Efeito do monitoramento por telefone de Intervenções Breves para uso de álcool e tabaco entre gestantes: ensaio clínico randomi- zado: Universidade Federal de São Carlos Centro de Ciências Biológicas e da Saúde; 2019.  7. Nilsen P, Holmqvist M, Bendtsen P, Hultgren E, Cedergren M. Is question- naire-based alcohol counseling more effective for pregnant women than standard maternity care? J Women’s Health (Larchmt). 2010;19(1):161–7.  8. O’Connor MJW, Shannon E. Brief intervention for alcohol use by pregnant women. Am J Public Health. 2007;97(2):252–8.  9. Ondersma SJ, Beatty JR, Svikis DS, Strickler RC, Tzilos GK, Chang G, et al. Com- puter-delivered screening and brief intervention for alcohol use in pregnancy: a pilot randomized trial. Alcohol Clin Exp Res. 2015;39(7):1219–26.  10. Osterman RL, Carle AC, Ammerman RT, Gates D. Single-session motivational intervention to decrease alcohol use during pregnancy. J Subst Abus Treat. 2014;47(1):10–9.  11. Osterman RL, Dyehouse J. Effects of a motivational interviewing interven- tion to decrease prenatal alcohol use. West J Nurs Res. 2012;34(4):434–54.  12. Peles E, Sason A, Bloch M, Maslovitz S, Dollberg S, Many A, et al. The prevalence of alcohol, substance and cigarettes exposure among pregnant women within a general hospital and the compliance to brief intervention for exposure reduction. Isr J Psychiatry Relat Sci. 2014;51(4):248–56.  13. Reynolds KD, Coombs DW, Lowe JB, Peterson PL, Gayoso E. Evaluation of a self-help program to reduce alcohol consumption among pregnant women. Int J Addict. 1995;30(4):427–43.  14. Rubio DM, Day NL, Conigliaro J, Hanusa BH, Larkby C, McNeil M, et al. Brief motivational enhancement intervention to prevent or reduce postpartum alcohol use: a single-blinded, randomized controlled effectiveness trial. J Subst Abus Treat. 2014;46(3):382–9.  15. Sarvela PD, Ford TD. An evaluation of a substance abuse education program for Mississippi delta pregnant adolescents. J Sch Health. 1993;63(3):147–52.  16. Sheehan J, Gill A, Kelly BD. The effectiveness of a brief intervention to reduce alcohol consumption in pregnancy: a controlled trial. Ir J Psychol Med. 2014;31(3):175–89.  17. Tzilos GK, Sokol RJ, Ondersma SJ. A randomized phase I trial of a brief computer-delivered intervention for alcohol use during pregnancy. J Women’s Health (Larchmt). 2011;20(10):1517–24.  18. Tzilos Wernette G, Plegue M, Kahler CW, Sen A, Zlotnick C. A pilot randomized controlled trial of a computer-delivered brief intervention for substance use and risky sex during pregnancy. J Women’s Health (Larchmt). 2018;27(1):83–92.  19. van der Wulp NY, Hoving C, Eijmael K, Candel MJ, van Dalen W, De Vries H. Reducing alcohol use during pregnancy via health counseling by midwives and internet-based computer-tailored feedback: a cluster randomized trial. J Med Internet Res. 2014;16(12):e274.  20. Waterson EJ, Murray-Lyon I. Preventing fetal alcohol effects; a trial of three methods of giving information in the antenatal clinic. Health Educ Res. 1990;5:53–61.  21. Winhusen T, Kropp F, Babcock D, Hague D, Erickson SJ, Renz C, et al. Motivational enhancement therapy to improve treatment utilization and outcome in pregnant substance users. J Subst Abus Treat. 2008;35(2):161–73.  22. Yonkers KA, Dailey JI, Gilstad-Hayden K, Ondersma SJ, Forray A, Olmstead TA, et al. Abstinence outcomes among women in reproductive health centers administered clinician or electronic brief interventions. J Subst Abus Treat. 2020;113:107995.  23. Yonkers KA, Forray A, Howell HB, Gotman N, Kershaw T, Rounsaville BJ, et al. Motivational enhancement therapy coupled with cognitive behavioral therapy versus brief advice: a randomized trial for treatment of hazardous substance use in pregnancy and after delivery. Gen Hosp Psychiatry. 2012;34(5):439–49. |
| Froschl *et al.* 2013 | Included studies not provided in the publication for this review |

**Unique publications included in alcohol reviews**

**Alcohol unique publications**

1. Armstrong MA, Kaskutas LA, Witbrodt J, Taillac CJ, Hung Y-Y, Osejo VM, et al. Using drink size to talk about drinking during pregnancy: a randomized clinical clinical trial of early start plus. Soc Work Health Care. 2009;48(1):90–103.
2. Belizán J., Barros F., Langer A., Farnot U., Victora C., Villar J. Impact of health education during pregnancy on behavior and utilization of health resources. Am J Obstet Gynecol 1995; 173: 894–49.
3. Chang G, McNamara TK, Orav EJ, Koby D, Lavigne A, Ludman B, et al. Brief intervention for prenatal alcohol use: a randomized trial. Obstet Gynecol. 2005;105(5 Pt 1):991–8.
4. Chang G, Wilkins‐Haug L, Berman S, Goetz MA. Brief intervention for alcohol use in pregnancy: a randomized trial. Addiction. 1999;94(10):1499–508.
5. Chang G, McNamara TK, Orav EJ, Koby D, Lavigne A, Ludman B, Vincitorio NA, Wilkins-Haug L. A brief intervention for prenatal alcohol use: results from a randomized trial of 304 couples. In: 15th Annual Meeting of American Academy of Addiction Research. Vol. 26. 2004.
6. Chang G, Goetz MA, Wilkins-Haug L, Berman S. A brief intervention for prenatal alcohol use: an in-depth look. Journal of Substance Abuse Treatment 2000;18(4):365-9. [DOI: 10.1016/ s0740-5472(99)00105-1] [PMID: 10812310]
7. Crawford-Williams F., Fielder A., Mikocka-Walus A., Esterman A., Steen M. A public health intervention to change knowledge, attitudes and behaviour regarding alcohol consumption in pregnancy. Evid Based Midwif 2016; 14: 4–10.
8. de Vries MM, Joubert B, Cloete M, Roux S, Baca BA, Hasken JM, et al. Indicated prevention of fetal alcohol Spectrum disorders in South Africa: effectiveness of case management. Int J Environ Res Public Health. 2015;13(1):ijerph13010076.
9. Evans WD, Wallace Bihm J, Szekely D, Nielsen P, Murray E, Abroms L, et al. Initial outcomes from a 4-week follow-up study of the Text4baby program in the military women's population: randomized controlled trial. J Med Internet Res 2014 May 20;16(5):e131
10. Evans WD, Wallace JL, Snider J. Pilot evaluation of the text4baby mobile health program. BMC Public Health 2012 Nov 26;12:1031
11. Fleming M. F., Lund M. R., Wilton G., Landry M., Scheets D. The healthy moms study: the efficacy of brief alcohol intervention in postpartum women. Alcohol Clin Exp Res 2008; 32: 1600–6.
12. Gwadz M. V., Leonard N. R., Cleland C. M., Riedel M., Arredondo G. N., Wolfe H., et al. Behavioral interventions for HIV infected and uninfected mothers with problem drinking. Addi Res Theory 2008; 16: 47–65.
13. Handmaker N. S., Miller W. R., Manicke M. Findings of a pilot study of motivational interviewing with pregnant drinkers. J Stud Alcohol 1999; 60: 285–7.
14. Joya X., Mazarico E., Ramis J., Pacifici R., Salat-Batlle J., Mortali C.,et al. Segmental hair analysis to assess effectiveness of single-session motivational intervention to stop ethanol use during pregnancy. Drug Alcohol Depend 2016; 158: 45–51.
15. Marais S, Jordaan E, Viljoen D, Olivier L, de Waal J, Poole C. The effect of brief interventions on the drinking behaviour of pregnant women in a high-risk rural south African community: a cluster randomised trial. Early Child Dev Care. 2011;181(4):463–74.
16. Meberg A, Halvorsen B, Holter B, Ek IJ, Askeland A, Gaaserud W, et al. Moder- ate alcohol consumption—need for intervention programs in pregnancy? Acta Obstet Gynecol Scand. 1986;65(8):861–4.
17. Moura, AAMD. Efeito do monitoramento por telefone de Intervenções Breves para uso de álcool e tabaco entre gestantes: ensaio clínico randomi- zado: Universidade Federal de São Carlos Centro de Ciências Biológicas e da Saúde; 2019.
18. Nilsen P, Holmqvist M, Bendtsen P, Hultgren E, Cedergren M. Is question- naire-based alcohol counseling more effective for pregnant women than standard maternity care? J Women’s Health (Larchmt). 2010;19(1):161–7.
19. O’Connor M. J., Whaley S. E. Brief intervention for alcohol use by pregnant women. Am J Public Health 2007; 97: 252–8.
20. Ondersma S. J., Beatty J. R., Svikis D. S., Strickler R. C., Tzilos G. K., Chang G., et al. Computer-delivered screening and brief intervention for alcohol use in pregnancy: a pilot randomized trial. Alcohol Clin Exp Res 2015;
21. Ondersma S. J., Svikis D. S., Thacker L. R., Beatty J. R., Lockhart N. A randomised trial of a computer-delivered screening and brief intervention for postpartum alcohol use. Drug Alcohol Rev 2016; 35: 710–8.
22. Osterman R. L., Carle A. C., Ammerman R. T., Gates D. Single session motivational intervention to decrease alcohol use during pregnancy. J Subst Abuse Treat 2014; 47: 10–9.
23. Osterman R. L., Dyehouse J. Effects of a motivational interviewing intervention to decrease prenatal alcohol use. West J Nurs Res 2012; 34: 434–54.
24. Osterman R., Lewis D., Winhusen T. Efficacy of motivational enhancement therapy to decrease alcohol and illicit-drug use in pregnant substance users reporting baseline alcohol use. J Subst Abuse Treat 2017; 77: 150–5.
25. Peles E, Sason A, Bloch M, Maslovitz S, Dollberg S, Many A, et al. The prevalence of alcohol, substance and cigarettes exposure among pregnant women within a general hospital and the compliance to brief intervention for exposure reduction. Isr J Psychiatry Relat Sci. 2014;51(4):248–56.
26. Reading A. E., Campbell S., Cox D. N., Sledmere C. M. Health beliefs and health-care behavior in pregnancy. Psychol Med 1982; 12: 379–83.
27. Reynolds K. D., Coombs D. W., Lowe J. B., Peterson P. L., Gayoso E. Evaluation of a self-help program to reduce alcohol consumption among pregnant women. Int J Addict 1995; 30: 427–43.
28. Rotheram-Borus M. J., Arfer K. B., Christodoulou J., Comulada W. S., Tubert J. E., Stewart J.,et al. The association of maternal alcohol use and paraprofessional home visiting with children’s health: a randomized controlled trial. J Consult Clin Psychol 2019; 87: 551–62.
29. Rubio D. M., Day N. L., Conigliaro J., Hanusa B. H., Larkby C., McNeil M.,et al. Brief motivational enhancement intervention to prevent or reduce postpartum alcohol use: a single-blinded, randomized controlled effectiveness trial. J Subst Abuse Treat 2014; 46: 382–9.
30. Sarvela PD, Ford TD. An evaluation of a substance abuse education program for Mississippi delta pregnant adolescents. J Sch Health. 1993;63(3):147–52.
31. Sheehan J, Gill A, Kelly BD. The effectiveness of a brief intervention to reduce alcohol consumption in pregnancy: a controlled trial. Ir J Psychol Med. 2014;31(3):175–89.
32. Slesnick N., Erdem G. Efficacy of ecologically-based treatment with substance-abusing homeless mothers: substance use and housing outcomes. J Subst Abuse Treat 2013; 45: 416–25.
33. Tzilos G. K., Sokol R. J., Ondersma S. J. A randomized phase I trial of a brief computer-delivered intervention for alcohol use during pregnancy. J Womens Health (Larchmt) 2011; 20: 1517–24.
34. Tzilos Wernette G, Plegue M, Kahler CW, Sen A, Zlotnick C. A pilot randomized controlled trial of a computer-delivered brief intervention for substance use and risky sex during pregnancy. J Women’s Health (Larchmt). 2018;27(1):83–92.
35. Tzilos GK, Ondersma SJ. Assessing feasibility of a brief computer-based intervention for alchol use during pregnancy. In: Alcoholism: Clinical and Experimental Research. Vol. 33. 2009:111A.
36. Tzilos GK, Ondersma SJ. Cognitive functioning and participant ratings of acceptability following computer-delivered brief intervention for alcohol use during pregnancy. In: Alcoholism: Clinical and Experimental Research. Vol. 35. 2011:145A.
37. Tzilos GK. A brief computer-based intervention for alcohol use during pregnancy. Graduate School of Wayne State University, Detroit, Michigan 2010.
38. van der Wulp N. Y., Hoving C., Eijmael K., Candel M. J., van Dalen W., de Vries H. Reducing alcohol use during pregnancy via health counseling by midwives and internet-based computer-tailored feedback: a cluster randomized trial. J Med Internet Res 2014; 16: e274.
39. Van Der Wulp NY, Hoving C, Van Dalen W, De Vries H. Preventing prenatal alcohol use via health counselling by midwives and Internet-based computer tailored feedback: a randomized controlled trial. In: Journal of Population Therapeutics and Clinical Pharmacology. Vol. 19(3). 2012:e419-20.
40. Waterson E. J., Murray-Lyon I. M. Preventing fetal alcohol effects; a trial of three methods of giving information in the antenatal clinic. Health Educ Res 1990; 5: 53–61.
41. Wernette GT, Plegue M, Kahler CW, Sen A, Zlotnick C. A Pilot Randomized Controlled Trial of a Computer-Delivered Brief Intervention for Substance Use and Risky Sex During Pregnancy. J Womens Health (Larchmt) 2018 Jan;27(1):83-92
42. Winhusen T, Kropp F, Babcock D, Hague D, Erickson SJ, Renz C, et al. Motivational enhancement therapy to improve treatment utilization and outcome in pregnant substance users. J Subst Abus Treat. 2008;35(2):161–73.
43. Yonkers K. A., Forray A., Howell H. B., Gotman N., Kershaw T., Rounsaville B. J., et al. Motivational enhancement therapy coupled with cognitive behavioral therapy versus brief advice: a randomized trial for treatment of hazardous substance use in pregnancy and after delivery. Gen Hosp Psychiatry 2012; 34: 439–49.
44. Yonkers KA, Dailey JI, Gilstad-Hayden K, Ondersma SJ, Forray A, Olmstead TA, et al. Abstinence outcomes among women in reproductive health centers administered clinician or electronic brief interventions. J Subst Abus Treat. 2020;113:107995.

**Included studies in smoking reviews**

| **Review** | **References of included smoking interventions delivered in pregnancy** |
| --- | --- |
| Acar *et al.* 2022 | 1. Aveyard P, Lawrence T, Cheng KK, Grifﬁn C, Croghan E, Johnson C (2006) A randomized controlled trial of smoking cessation for pregnant women to test the effect of a transtheoretical model-based intervention on movement in stage and interaction with baseline stage. Br J Health Psychol, 11:263–278. 2. Karatay G, Kublay G, Emiroğlu ON (2010) Effect of motivational interviewing on smoking cessation in pregnant women. J Adv Nurs 66:1328–1337 3. Lawrence T, Aveyard P, Cheng KK (2003) A cluster randomised controlled trial of smoking cessation in pregnant women comparing interventions based on the transtheoretical (stages of change) model to standard care. Tob Control, 12:168-177. 4. Patten CA, Koller KR, Flanagan CA, Hiratsuka VY, Hughes CA, Wolfe AW et al. (2019) Biomarker feedback intervention for smoking cessation among Alaska Native pregnant women: Randomized pilot study. Patient Educ Couns, 102:528-535. 5. Patten CA, Windsor RA, Renner CC, Enoch C, Hochreiter A, Nevak C et al. (2010) Feasibility of a tobacco cessation intervention for pregnant Alaska Native women. Nicotine Tob Res, 12:79-87. 6. Pletsch PK (2002) Reduction of primary and secondary smoke exposure for low-income black pregnant women. Nurs Clin, 37:315-329. 7. Rigotti NA, Park ER, Regan S, Chang Y, Perry K, Loudin B et al. (2006) Efficacy of telephone counseling for pregnant smokers. Obstet Gynecol, 108:83–92 8. Stotts AL, DeLaune KA, Schmitz JM, Grabowski J (2004) Impact of a motivational intervention on mechanisms of change in low-income pregnant smokers. Addict Behav, 29:1649–1657. 9. Tappin D, Bauld L, Purves D, Boyd K, Sinclair L, MacAskill S et al (2015) Financial incentives for smoking cessation in pregnancy: Randomised controlled trial. BMJ, 350:h134 |
| Brown *et al.* 2019 | 1. Allen S.S., Allen A.M., Lunos S., Tosun N. Progesterone and postpartum smoking relapse: A pilot double-blind placebo-controlled randomized trial. Nicotine & Tobacco Research. 2016;18(11):2145–2153. 2. Brandon T.H., Simmons V.N., Meade C.D. Self-help booklets for preventing postpartum smoking relapse: A randomized trial. American Journal of Public Health. 2012;102(11):2109–2115 3. Cummins S.E., Tedeschi G.J., Anderson C.M., Zhu S.H. Telephone intervention for pregnant smokers: A randomized controlled trial. American Journal of Preventive Medicine. 2016;51(3):318–326 4. Edwards N.C., Sims-Jones N. A randomized controlled trial of alternative approaches to community follow-up for postpartum women. Canadian Journal of Public Health. 1997;88(2):123–128. 5. El-Mohandes A.A., El-Khorazaty M.N., Kiely M., Gantz M.G. Smoking cessation and relapse among pregnant African-American smokers in Washington, DC. Maternal and Child Health Journal. 2011;15(Suppl. 1):S96–105 6. Ershoff D.H., Quinn V.P., Mullen P.D. Relapse prevention among women who stop smoking early in pregnancy: A randomized clinical trial of a self-help intervention. American Journal of Preventive Medicine. 1995;11(3):178–184. 7. Forray A., Gilstad-Hayden K., Sofuoglu M., Yonkers K. Progesterone for postpartum smoking relapse prevention. Neuropsychopharmacology. 2016;41:S606. 8. Forray A., Waters A. Ecological momentary assessments and attentional bias modification for postpartum smoking. Neuropsychopharmacology. 2015;40:S590–S591 9. Hajek P., West R., Lee A. Randomized controlled trial of a midwife-delivered brief smoking cessation intervention in pregnancy. Addiction. 2001;96(3):485–494 10. Hannover W., Thyrian J.R., Roske K. Smoking cessation and relapse prevention for postpartum women: Results from a randomized controlled trial at 6, 12, 18 and 24 months. Addictive Behaviors. 2009;34(1):1–8. 11. Jimenez-Muro A., Nerin I., Samper P. A proactive smoking cessation intervention in postpartum women. Midwifery. 2013;29(3):240–245 12. Johnson J.L., Ratner P.A., Bottorff J.L., Hall W., Dahinten S. Preventing smoking relapse in postpartum women. Nursing Research. 2000;49(1):44–52. 13. Kendrick J.S., Zahniser S.C., Miller N. Integrating smoking cessation into routine public prenatal care: The Smoking Cessation in Pregnancy project. American Journal of Public Health. 1995;85(2):217–222. 14. Kientz E., Kupperschmidt B. KICCS: A successful strategy to promote smoking cessation in women during and post pregnancy. The Oklahoma Nurse. 2005;50(4):27–30. 15. Levine M.D., Cheng Y., Marcus M.D., Kalarchian M.A., Emery R.L. Preventing postpartum smoking relapse: A randomized clinical trial. JAMA Internal Medicine. 2016;176(4):443–452 16. Lillington L., Royce J., Novak D., Ruvalcaba M., Chlebowski R. Evaluation of a smoking cessation program for pregnant minority women. Cancer Practice. 1995;3(3):157–163. 17. McBride C.M., Baucom D.H., Peterson B.L. Prenatal and postpartum smoking abstinence a partner-assisted approach. American Journal of Preventive Medicine. 2004;27(3):232–238. 18. McBride C.M., Curry S.J., Lando H.A., Pirie P.L., Grothaus L.C., Nelson J.C. Prevention of relapse in women who quit smoking during pregnancy. American Journal of Public Health. 1999;89(5):706–711. 19. Morasco B.J., Dornelas E.A., Fischer E.H., Oncken C., Lando H.A. Spontaneous smoking cessation during pregnancy among ethnic minority women: A preliminary investigation. Addictive Behaviors. 2006;31(2):203–210. 20. Mullen P.D., DiClemente C.C., Bartholomew L.K. Theory and context in project PANDA: a program to help postpartum women stay off cigarettes. In: Bartholomew L.K., Parcel G.S., Kok G., Gottlieb N.H., editors. Intervention Mapping: A Process for Designing Theory- and Evidence-based Health Promotion Programs. McGraw-Hill Publishing Co.; Palo Alto, CA: Mayfield: 2001 21. Pbert L., Ockene J.K., Zapka J. A community health center smoking-cessation intervention for pregnant and postpartum women. American Journal of Preventive Medicine. 2004;26(5):377–385. 22. Petersen L., Handel J., Kotch J., Podedworny T., Rosen A. Smoking reduction during pregnancy by a program of self-help and clinical support. Obstetrics and Gynecology. 1992;79:924–930 23. Polanska K., Hanke W., Sobala W., Lowe J.B. Efficacy and effectiveness of the smoking cessation program for pregnant women. International Journal of Occupational Medicine and Environmental Health. 2004;17(3):369–377 24. Pollak K.I., Fish L.J., Lyna P. Efficacy of a nurse-delivered intervention to prevent and delay postpartum return to smoking: The quit for two trial. Nicotine & Tobacco Research. 2016;18(10):1960–1966 25. Reitzel L.R., Vidrine J.I., Businelle M.S. Preventing postpartum smoking relapse among diverse low-income women: A randomized clinical trial. Nicotine & Tobacco Research. 2010;12(4):326–335 26. Ruger J.P., Weinstein M.C., Hammond S.K., Kearney M.H., Emmons K.M. Cost-effectiveness of motivational interviewing for smoking cessation and relapse prevention among low-income pregnant women: A randomized controlled trial. Value in Health. 2008;11(2):191–198. 27. Secker-Walker R.H., Solomon L.J., Flynn B.S. Smoking relapse prevention counseling during prenatal and early postnatal care. American Journal of Preventive Medicine. 1995;11(2):86–93. 28. Secker-Walker R.H., Solomon L.J., Flynn B.S., Skelly J.M., Mead P.B. Smoking relapse prevention during pregnancy. A trial of coordinated advice from physicians and individual counseling. American Journal of Preventive Medicine. 1998;15(1):25–31. 29. Strecher V.J., Bishop K.R., Bernhardt J., Thorp J.M., Cheuvront B., Potts P. Quit for keeps: tailored smoking cessation guides for pregnancy and beyond. Tobacco Control. 2000;9(Suppl. 3) (Iii78-79) 30. Suplee P.D. The importance of providing smoking relapse counseling during the postpartum hospitalization. Journal of Obstetric, Gynecologic, and Neonatal Nursing. 2005;34(6):703–712. 31. Thornton L. Department of Public Health, Eastern Health Board; Ireland: 1997. The rotunda stop smoking programme: smoking and pregnancy: Feasability and effectiveness of a smoking intervention programme among pregnant women 32. Winickoff J.P., Healey E.A., Regan S. Using the postpartum hospital stay to address mothers' and fathers' smoking: The NEWS Study. Pediatrics. 2010;125(3):518. |
| Chamberlain *et al.* 2013 | 1. Albrecht S, Cassidy B, Salamie D, Reynolds M. What’s happening. A smoking cessation intervention for pregnant adolescents: implications for nurse practitioners. Journal of American Academy of Nurse Practitioners. 1999; 11(4):155–9. 2. Albrecht S, Cornelius M, Braxter B, Reynolds M, Stone C, Cassidy B. An assessment of nicotine dependence among pregnant adolescents. Journal of Substance Abuse Treatment. 1999; 16(4): 337–44. 3. Albrecht S, Stone CA, Payne L, Reynolds MD. A preliminary study of the use of peer support in smoking cessation programs for pregnant adolescents. Journal of the American Academy of Nurse Practitioners. 1998; 10:119– 25. 4. Albrecht SA, Caruthers D, Patrick T, Reynolds M, Salamie D, Higgins LW, et al. A randomised controlled trial of a smoking cessation intervention for pregnant adolescents. Nursing Research. 2006; 55(6):402–10. 5. Albrecht SA, Caruthers D. Characteristics of inner-city pregnant Smoking teenagers. Journal of Obstetric Gynecologic and Neonatal Nursing. 2002; 31:462–9. 6. Albrecht SA, Higgins LW, Lebow H. Knowledge about the deletrious effects of smoking and its relationship to smoking cessation among pregnant adolescents. Adolescence. 2000; 35(140):709– 16. 7. Albrecht SA, Higgins LW, Stone C. Factors relating to pregnant adolescents’ decisions to complete a smoking cessation intervention. Journal of Pediatric Nursing. 1999; 14(5):322–8. 8. Albrecht, SA.; Patrick, T.; Kim, Y.; Caruthers, D. A randomised controlled trial of a smoking cessation intervention for pregnant adolescents. Society for Research on Nicotine and Tobacco 9th Annual Meeting; New Orleans, Louisiana. 2003 February 19-23; 2003. p. 91 9. Aranda Regules JM, Mateos Vilchez P, Gonzalez Villalba A, Sanchez F, de Dios Luna del Castillo J. Validity of smoking measurements during pregnancy: specificity, sensitivity and cut-off points. Revista Espanola de Salud Publica. 2008; 82(5):535–45. 10. Aveyard P, Lawrence T, Cheng KK, Griffin C, Croghan E, Johnson C. A randomized controlled trial of smoking cessation for pregnant women to test the effect of a transtheoretical model-based intervention on movement in stage and interaction with baseline stage. British Journal of Health Psychology. 2006; 11:263–78. 11. Aveyard P, Lawrence T, Croghan E, Evans O, Cheng KK. Is advice to stop smoking from a midwife stressful for pregnant women who smoke? Data from a randomized controlled trial. Preventive Medicine. 2004; 40:575–82. 12. Aveyard P, Lawrence T, Evans O, Cheng KK. The influence of in-pregnancy smoking cessation programmes on partner quitting and women’s social support mobilization: a randomized controlled trial. BMC Public Health. 2005; 5:80. 13. Aveyard P, West R. Managing smoking cessation. BMJ. 2007; 335:37–41. 14. Bailey, JW.; Loeb, BK.; Waage, G. A randomized trial of smoking intervention during pregnancy. Proceedings of the American Public Health Association 111th Annual Meeting; Dallas, Texas, USA. 1983 Nov 15; 1983. p. 58 15. Baric L, MacArthur C, Sherwood M. A study of health education aspects of smoking in pregnancy. International Journal of Health Education. 1976; 19(2 Suppl):1–17. 16. Baric L, MacArthur C. Health norms in pregnancy. British Journal of Preventive and Social Medicine. 1977; 31:30–8. 17. Bauman KE, Koch GG, Dent CW, Bryan ES. The influence of observing carbon monoxide level on cigarette smoking by public prenatal patients. American Journal of Public Health. 1983; 73:1089–91. 18. Belizan JM, Villar J, Victora C, Farnot U, Langer A, Barros F. Impact of health education during pregnancy on behavior and utilization of health resources. American Journal of Obstetrics and Gynecology. 1995; 173:894–9 19. Berg CJ, Park ER, Chang Y, Rigotti NA. Is concern about post-cessation weight gain a barrier to smoking cessation among pregnant women? Nicotine & Tobacco Research. 2008; 10(7):1159–63. 20. Bernstein IM, Mongeon JA, Badger GJ, Solomon L, Heil SH, Higgins ST. Maternal smoking and its association with birthweight. Obstetrics & Gynecology. 2005; 106(5 Pt 1):986–91. [PubMed: 16260516] Heil, SH.; Higgins, ST. Characterizing nicotine withdrawal and craving in pregnant cigarette smokers. 66th Annual Scientific Meeting of the College on Problems of Drug Dependence; San Juan, Puerto Rico. 2004 June 12-17; 2004. * 21. Blake SM, Murray KD, ElKhorazaty MN, Gantz MG, Kiely M, Best D, et al. Environmental tobacco smoke avoidance among pregnant African-American nonsmokers. American Journal of Preventive Medicine. 2009; 36(3):225–34. 22. Blake, S.; El-Mohandes, A.; Schwartz, D.; ElKhorazaty, N.; Gantz, M.; Joseph, J., et al. Promoting smoking cessation during pregnancy and preventing postpartum relapse [abstract]. Pediatric Academic Societies Annual Meeting; Washington DC, USA. 2005 May 14-17; 2005. Abstract no: 3074 23. Blake, S.; Joseph, J.; Schwartz, D.; El-Khorazaty, N.; Gantz, M.; El-Mohandes, A., et al. Preventing prenatal and postpartum environmental tobacco smoke (ETS) exposure [abstract]. Pediatric Academic Societies Annual Meeting; Washington DC, USA. 2005 May 14-17; Abstract no: 2353 24. Blalock JA, Fouladi RT, Wetter DW, Cinciripini PM. Depression in pregnant women seeking smoking cessation treatment. Addictive Behaviours. 2005; 30(6):1195–208. 25. Bonollo DP, Zapka JG, Stoddard AM, Ma Y, Pbert L, Ockene JK. Treating nicotine dependence during pregnancy and postpartum: understanding clinician knowledge and performance. Patient Education and Counselling. 2002; 48:265–74. 26. Bullock L, Everett KD, Mullen PD, Geden E, Longo DR, Madsen R. Baby BEEP: A randomized controlled trial of nurses’ individualized social support for poor rural pregnant smokers. Maternal and Child Health Journal. 2009; 13(3):395–406. 27. Bullock LF, Everett KD, Mullen PD. Baby beep: a randomized clinical trial of smoking cessation for low-income rural pregnant women using nurse-delivered social support. Annals of Behavioral Medicine. 2008; 35:S99. 28. Bullock LF, Hornblow AR, Duff GB, Wells JE. Telephone support for pregnant women: outcome in late pregnancy. New Zealand Medical Journal. 1995; 108:476–8. 29. Burling TA, Bigelow GE, Robinson JC, Mead AM. Smoking during pregnancy: reduction via objective assessment and directive advice. Behavior Therapy. 1991; 22:31–40. 30. Byrd JC, Meade CD. Smoking cessation among pregnant women in an urban setting. Wisconsin Medical Journal. 1993; 92:609–12. 31. Calderón SH, Gilbert P, Jackson R, Kohn MA, Gerbert B. Cueing prenatal providers: effects on discussions of intimate partner violence. American Journal of Preventive Medicine. 2008; 34(2):134–7. 32. Campbell E, Walsh RA, Sanson-Fisher, Burrows S, Stojanovski E. A group randomised trial of two methods for disseminating a smoking cessation programme to public antenatal clinics: effects on patient outcomes. Tobacco Control. 2006; 15(2):97–102 33. Cinciripini PM, Blalock JA, Minnix JA, Robinson JD, Brown VL, Lam C, et al. Effects of an intensive depression-focused intervention for smoking cessation in pregnancy. Journal of Consulting & Clinical Psychology. 2010; 78(1):44–54. 34. Cinciripini PM, McClure JB, Wetter DW, Perry J, Blalock JA, Cinciripini LG, et al. An evaluation of videotaped vignettes for smoking cessation and relapse prevention during pregnancy: The Very Important Pregnant Smokers (VIPS) Program. Tobacco Control. 2000; 9(3):iii61–iii63. 35. Cook C, Ward S, Myers S, Spinnato J. A prospective, randomized evaluation of intensified therapy for smoking reduction in pregnancy. American Journal of Obstetrics and Gynecology. 1995; 172:290. 36. Cooke M, Mattick R, Campbell E. The influence of individual and organisational factors on the reported smoking intervention practices of staff in 20 antenatal clinics. Drug and Alcohol Review. 1998; 17(2): 175–85. 37. Cooke M, Mattick RP, Campbell E. A description of the adoption of the ‘Fresh Start’ smoking cessation program by antenatal clinic managers. Australian Journal of Advanced Nursing. 2000; 18(1):13–21 38. Cooke M, Mattick RP, Campbell E. The dissemination of a smoking cessation program to 23 antenatal clinics: the predictors of initial program adoption by managers. Australian and New Zealand Journal of Public Health. 1999; 23(1):99–103. 39. Cooke M, Mattick RP, Walsh RA. Differential uptake of a smoking cessation programme disseminated to doctors and midwives in antenatal in antenatal clinics. Addiction. 2001; 96(3):495–505. 40. Cooke M, Mattick RP, Walsh RA. Implementation of the ‘Fresh Start’ smoking cessation programme to 23 antenatal clinics: a randomized controlled trial investigating two methods of dissemination. Drug and Alcohol Review. 2001; 20:19–28. 41. Cope G, Nayyar P, Holder R, Gibbons J, Brunce R. A simple near patient test for nicotine and its metabolites in urine to assess smoking habit. Clinical Chimica Acta. 1996; 256:135–49.Cope GF. Smoking status and pregnancy: point of care cotinine test. BMJ. 2009; 339:b5652 42. Cope GF, Nayyar P, Holder R. Feedback from a point-of-care test for nicotine intake to reduce smoking during pregnancy. Annals of Clinical Biochemistry. 2003; 40(Pt 6):674–9. 43. Cope GF, Nayyar P, Holder R. Measurement of nicotine intake in pregnant women - associations to changes in blood cell count. Nicotine & Tobacco Research. 2001; 3(2):119–22 44. Crittenden KS, Manfredi C, Cho YI, Dolecek TA. Smoking cessation processes in lowSES women: the impact of timevarying pregnancy status, healthcare messages, stress and health concerns. Addictive Behaviors. 2007; 32:1347–66. 45. Curry SJ, McBride C, Grothus L, Lando H, Pirie P. Motivation for smoking cessation among pregnant women. Psychology of Addictive Behaviors. 2001; 15(2):126–32. 46. Donatelle RJ, Hudson D, Dobie S, Goodall A, Hunsberger M, Oswald K. Incentives in smoking cessation: status of the field and implications for research and practice with pregnant smokers. Nicotine and Tobacco Research. 2004; 6(S2):S163–S179. 47. Donatelle RJ, Prows SL, Champeau D, Hudson D. Randomised controlled trial using social support and financial incentives for high risk pregnant smokers: Significant Other Supporter (SOS) program. Tobacco Control. 2000; 9(Suppl 3):iii67–iii69 48. Donatelle, RJ.; Hudson, D. Using 5 A’s and incentives to promote prenatal smoking cessation. National Conference of Tobacco or Health; San Francisco, California, USA. 2002 November 19-21; 2002. 49. Donovan J. Randomised controlled trial of anti-smoking advice in pregnancy. British Journal of Preventive and Social Medicine. 1977; 31(1):6–12. 50. Donovan JW, Burgess PL, Hossack CM, Yudkin GD. Routine advice against smoking in pregnancy. Journal of the Royal College of General Practitioners. 1975; 25(153):264–8. 51. Donovan JW. Randomised controlled trial of anti-smoking advice in pregnancy. Journal of Epidemiology and Community Health. 1996; 50(3):232–6. 52. Dornelas EA, Magnavita J, Beazoglou T, Fischer EH, Oncken C, Lando H, et al. Efficacy and cost-effectiveness of a clinicbased counseling intervention tested in an ethnically diverse sample of pregnant smokers. Patient Education and Counseling. 2006; 64(1-3):342–9. 53. Dunkley J. Training midwives to help pregnant women stop smoking. Nursing Times. 1997; 93(5):64–6. 54. Eades SJ, Sanson-Fisher RW, Wenitong M, Panaretto K, D’Este C, Gilligan C, et al. An intensive smoking intervention for pregnant Aboriginal and Torres Strait Islander women: a randomised controlled trial. Medical Journal of Australia. 2012; 197(1):42–6. 55. El-Khorazaty MN, Johnson AA, Kiely M, ElMohandes AA, Subramanian S, Laryea HA, et al. Recruitment and retention of low-income minority women in a behavioral intervention to reduce smoking, depression, and intimate partner violence during pregnancy. BMC Public Health. 2007; 7:233. 56. El-Mohandes AA, El-Khorazaty MN, Kiely M, Gantz MG. Smoking cessation and relapse among pregnant African-American smokers in Washington, DC. Maternal & Child Health Journal. 2011; 15(Suppl 1):S96–S105. 57. El-Mohandes AA, Kiely M, Blake SM, Gantz MG, El-Khorazaty MN. An intervention to reduce environmental tobacco smoke exposure improves pregnancy outcomes. Pediatrics. 2010; 125(4):721–8. 58. El-Mohandes AA, Kiely M, Joseph JG, Subramanian S, Johnson AA, Blake SM, et al. An intervention to improve postpartum outcomes in African-American mothers: a randomized controlled trial. Obstetrics & Gynecology. 2008; 112(3):611–20. 59. El-Mohandes AEE, Kiely M, Gantz MG, El-Khorazaty N. Very preterm birth is reduced in women receiving an integrated behavioural intervention: A randomized controlled trial. Maternal Child Health Journal. 2011; 15:19–28. 60. El-Mohandes, AAE. A psycho-behavioral intervention on African American pregnant women with a history of intimate partner violence (IPV) improves birth weight distribution of their newborns [abstract]. Pediatric Academic Societies Annual Meeting; San Francisco, CA, USA. 2006 April 29-May 2 61. El-Mohandes, AAE. An integrated behavioral intervention reduces rates of moderate and extreme prematurity in African American (AA) mothers with a history of smoking during pregnancy [abstract]. Pediatric Academic Societies Annual Meeting; San Francisco, CA, USA. 2006 April 29-May 2 62. El-Mohandes, AAE.; for the NIH-DC initiative to reduce infant mortality. An integrated psycho-behavioral intervention during pregnancy has significant effects in reducing risks during the post-partum period in African-American women. Pediatric Academic Societies Annual Meeting; Washington DC, USA. 2005 May 14-17; Abstract no: 39 63. El-Mohandes, AAE.; Kiely, M.; Gantz, MG.; ElKhorazaty, N. A multiple risk factor behavioral intervention reduces environmental tobacco smoke exposure. Pediatric Academic Societies Annual Meeting; Toronto, Canada. 2007 May 5-8 64. England LJ, Kendrick JS, Wilson HG, Merritt RK, Gargiullo PM, Zahniser SC. Effects of smoking reduction during pregnancy on the birth weight of term infants. American Journal of Epidemiology. 2001; 154:694–701. 65. Ershoff DH, Lairson DR, Mullen PD, Quinn VP. Pregnancy and medical cost outcomes of a self-help prenatal smoking cessation program in an HMO. Public Health Reports. 1990; 105(4):340–7. 66. Ershoff DH, Quinn VP, Boyd NR, Stern J, Gregory M, Wirtschafter D. The Kaiser Permanente prenatal smoking cessation trial. When more isn’t better, what is enough?. American Journal of Preventive Medicine. 1999; 17(3):161–8. 67. Ershoff DH, Quinn VP, Boyd NR, Stern J, Gregory M, Wirtschafter D. The Kaiser Permanente prenatal smoking cessation trial: when more isn’t better, what is enough? Tobacco Control. 2000; 9(Suppl 3):iii60. 68. Ershoff DH, Quinn VP, Mullen PD. A randomized trial of a serialized self-help smoking cessation program for pregnant women in an HMO. American Journal of Public Health. 1989; 79(2):182– 7. 69. Ershoff DH, Quinn VP, Mullen PD. Relapse prevention among women who stop smoking early in pregnancy: a randomized clinical trial of a self-help intervention. American Journal of Preventive Medicine. 1995; 11(3):178–84. 70. Ershoff DH, Solomon LJ, Dolan-Mullen P. Predictors of intentions to stop smoking early in prenatal care. Tobacco Control. 2000; 9(3):41. 71. Ewigman B, Crane J, Frigoletto F, LeFevre M, Bain R, McNellis D. Effect of prenatal ultrasound screening on perinatal outcome. New England Journal of Medicine. 1993; 329:821–9. 72. Fox NL, Sexton M, Hebel JR, Thompson B. The reliability of self-reports of smoking and alcohol consumption by pregnant women. Addictive Behaviors. 1989; 14(2):187–95. [ 73. Fox NL, Sexton MJ, Hebel JR. Alcohol consumption among pregnant smokers: effects of a smoking cessation intervention program. American Journal of Public Health. 1987; 77:211–3. 74. Gielen AC, Windsor R, Faden RR, O’Campo P, Repke J, Davis M. Evaluation of a smoking cessation intervention for pregnant women in an urban prenatal clinic. Health Education Research. 1997; 12(2):247–54. 75. Gilligan C, Sanson-Fisher R, Eades S, Wenitong M, Panaretto K, D’Este C. Assessing the accuracy of self-reported smoking status and impact of passive smoke exposure among pregnant Aboriginal and Torres Strait Islander women using cotinine biochemical validation. Drug and Alcohol Review. 2010; 29:35–40. 76. Gilligan C, Sanson-Fisher RW, D-Este C, Eades S, Wenitong M. Knowledge and attitudes regarding smoking during pregnancy among Aboriginal and Torres Strait Islander women. Medical Journal of Australia. 2009; 190(10):557–61. 77. Gilligan, C. A pilot randomised controlled trial to test the effectiveness of an intervention to help Aboriginal and Torres Strait Islander women to quit smoking during pregnancy: study design and preliminary results [thesis]. University of Newcastle; Newcastle, Australia: 2008. 78. Graham AV, Reeb KG, Kitson GC, Zyzanski SJ, Frank SH. A clinical trial to reduce the rate of low birth weight in an inner-city black population. Family Medicine. 1992; 24:439–46. 79. Groff, J.; Stotts, A.; Velasquez, M.; Benjamin-Garner, R.; Green, C.; Mastrobattista, J. Ultrasound and motivational enhancement for prenatal smoking cessation. Annual Meeting of the Society for Behavioural Medicine; Boston, MA. 2005 April 13-16; 2005. 80. Haddow JE, Polomak JE, Sepulveda D. Smoking cessation during routine public prenatal care. American Journal of Public Health. 1995; 85(10):1451–2. 81. Haddow JE, Wald NJ, Palomaki GE, Kloza EM, Knight GJ. Cotinine-assisted intervention in pregnancy to reduce smoking and low birthweight delivery. British Journal of Obstetrics and Gynaecology. 1991; 98(9):859–65. 82. Hajek P, West R, Lee A, Foulds J, Owen L, Eiser JR, et al. Randomized controlled trial of a midwife-delivered brief smoking cessation intervention in pregnancy. Addiction. 2001; 96(3):485–94 83. Hamilton BH. Estimating treatment effects in randomized clinical trials with non-compliance: the impact of maternal smoking on birthweight. Health Economics. 2001; 10(5):399–410. 84. Hartmann K, Thorp J, Pahel-Short L, Koch M. A randomized controlled trial of smoking cessation intervention in pregnancy. American Journal of Obstetrics and Gynecology. 1995; 172:287 85. Hartmann KE, Koch MA, Pahel-Short L, Thorp JM. A randomized controlled trial of smoking cessation intervention in pregnancy in an academic clinic. Obstetrics & Gynecology. 1996; 87:621–6 86. Haug K, Fugelli P, Aaro LE, Foss OP. Is smoking intervention in general practice more successful among pregnant than non-pregnant women? Family Practice. 1994; 11:111–6. 87. Haug K, Fugelli P, Aaro LE. Recruitment and participation of General Practitioners in a multipractice study of smoking cessation. Scandinavian Journal of Primary Health Care. 1992; 10(3):206–10. 88. Haug NA, DiClemente C, Svikis DS. Motivational enhancement therapy for nicotine dependence in methadone-maintained pregnant women. Psychology of Addictive Behaviours. 2004; 18(3):289–92. 89. Hebel JR, Sexton M, Nowicki P. The effect of antismoking intervention during pregnancy: an assessment of interactions with maternal characteristics. American Journal of Epidemiology. 1985; 122:135–48. 90. Hegaard H, Hjaergaard H, Moller L, Wachmann H, Ottesen B. Multimodel intervention raises smoking cessation rate during pregnancy. Acta Obstetricia et Gynecologica Scandinavica. 2003; 82:813–9. 91. Hegaard HK, Kjaergaard H, Moller LF, Wachmann H, Ottesen B. Determination of a saliva cotinine cut-off to distinguish pregnant smokers from pregnanct non-smokers. Acta Obstetricia et Gynecologica. 2007; 86:401–6. 92. Hegaard HK, Kjaergaard H, Moller LF, Wachmann H, Ottesen B. Long-term nicotine replacement therapy. British Journal of Midwifery. 2004; 12(4):214–20. 93. Heil SH, Higgins ST, Bernstein IM, Solomon LJ, Rogers RE, Thomas CS, et al. Effects of voucher-based incentives on abstinence from cigarette smoking and fetal growth among pregnant women. Addiction. 2008; 103(6):1009–18. 94. Heil SH, Tidey JW, Holmes HW, Badger GJ, Higgins ST. A contingent payment model of smoking cessation: effects on abstinence and withdrawal. Nicotine and Tobacco Research. 2003; 5(2):205–13. 95. Heil SH.; Higgins, ST.; Solomon, LJ.; Lynch, ME.; McHale, L.; Dumeer, A., et al. Voucher-based incentives for abstinence from cigarette smoking in pregnant and postpartum women. Society for Research on Nicotine and Tobacco 13th Annual Meeting; Austin, Texas. 2007 Feb 21-24; 2007. p. 25Abstract no: PA6-1 96. Hennrikus D, Pirie P, Hellerstedt W, Lando HA, Steele J, Dunn C. Increasing support for smoking cessation during pregnancy and postpartum: results of a randomized controlled pilot study. Preventive Medicine. 2010; 50(3):134–7. 97. Hiett A, Brazus S, Hedberg J, Brown H. Smoking cessation program effectiveness during pregnancy. American Journal of Obstetrics and Gynecology. 2000; 182(1 Pt 2):S150. 98. Higgins ST, Bernstein IM, Washio Y, Heil SH, Badger GJ, Skelly JM, et al. Effects of smoking cessation with voucher-based contingency management on birth outcomes. Addiction. 2010; 105:2023–30. 99. Higgins ST, Heil SH, Badger GJ, Mongeon JA, Solomon LJ, McHale L, et al. Biochemical verification of smoking status in pregnant and recently postpartum women. Experimental and Clinical Psychopharmacology. 2007; 15(1):58–66. [ 100. Higgins ST, Heil SH, Dumeer AM, Thomas CS, Solomon LJ, Bernstein IM. Smoking status in the initial weeks of quitting as a predictor of smoking-cessation outcomes in pregnant women. Drug and Alcohol Dependence. 2006; 85:138–41. 101. Higgins ST, Heil SH, Solomon LJ, Bernstein IM, Lussier JP, Abel RL, et al. A pilot study on voucher-based incentives to promote abstinence from cigarette smoking during pregnancy and postpartum. Nicotine and Tobacco Research. 2004; 6(6):1015–20. [ 102. Higgins TM, Higgins ST, Heil SH, Badger GJ, Skelly JM, Bernstein IM, et al. Effects of cigarette smoking cessation on breastfeeding duration. Nicotine & Tobacco Research. 2010; 12(5):483–8. 103. Hjalmarson AIM, Svanberg B, Hahn L. Stopping smoking in pregnancy: effect of a self-help manual in a controlled trial. British Journal of Obstetrics and Gynaecology. 1991; 98:260–4. 104. Hughes E, Lamont D, Beecroft M, Wilson D. Randomized trial of a “stage-of-change” orientated smoking cessation intervention in infertile and pregnant women. Fertility and Sterility. 2000; 74(3):498–503. 105. Hughes EG, Beecroft ML, Lamont D, Rice S, Wilson D, Freebury M, et al. A randomised controlled trial of a “State-of Change” smoking cessation intervention for subfertile and pregnant patients. Fertility and Sterility. 1999; 72(3 Suppl 1):S61–S62. 106. Joseph JG, El-Mohandes AA, Kiely M, El-Khorazaty MN, Gantz MG, Johnson AA, et al. Reducing psychosocial and behavioral pregnancy risk factors: results of a randomized clinical trial among high-risk pregnant African American women. American Journal of Public Health. 2009; 99(6):1053–61. 107. Joseph, J.; for NIH-DC initiative to reduce infant mortality. Randomized trial to reduce 4 behaviors linked to adverse pregnancy outcomes among 1048 inner-city African American women [abstract]. Pediatric Academic Societies Annual Meeting; Washington DC, USA. 2005 May 14-17; Abstract no: 1701 108. Katz KS, Blake SM, Milligan RA, Sharps PW, White DB, Rodan MF, et al. The design, implementation and acceptability of an integrated intervention to address multiple behavioural and psychosocial risk factors among pregnant African American women. BMC Pregnancy and Childbirth. 2008; 8(22) 109. Kendrick JS, Metzger RW, Sexton M, Spierto FW, Floyd RL, Gargiullo PM, et al. Integrating smoking cessation into routine public prenatal care: the Smoking Cessation in Pregnancy project. American Journal of Public Health. 1995; 85:217–22. 110. Kiely, M.; ElKhorazaty, MN.; El-Mohandes, AAE. Depression and smoking during pregnancy impact the efficacy of an integral behavioral intervention to resolve risks. Pediatric Academic Societies Annual Meeting; Toronto, Canada. 2007 May 5-8 111. Lando HA, Valanis BG, Lichtenstein E, Curry SJ, McBride CM, Pirie PL, et al. Promoting smoking abstinence in pregnant and postpartum patients: a comparison of 2 approaches. American Journal of Managed Care. 2001; 7:685–93. [ 112. Lawrence T, Aveyard P, Cheng KK, Griffin C, Johnson C, Croghan E. Does stage-based smoking cessation advice in pregnancy result in long term quitters? 18-month postpartum follow-up of a randomized controlled trial. Addiction. 2005; 100:107–16. 113. Lawrence T, Aveyard P, Croghan E. What happens to women’s self-reported cigarette consumption and urinary cotinine levels in pregnancy? Addiction. 2003; 98:1315–20. [ 114. Lawrence T, Aveyard P, Evans O, Cheng KK. A cluster randomised controlled trial of smoking cessation in pregnant women comparing interventions based on the transtheoretical (stages of change) model to standard care. Tobacco Control. 2003; 12:168–77. 115. Lawrence, T.; Aveyard, P.; Cheng, KK.; Griffin, C.; Johnson, C.; Croghan, E. Does stage-based smoking advice in pregnancy result in long-term quitters? 18- month post-partum follow up of a randomised controlled trial. Society for Research on Nicotine and Tobacco 11th Annual Meeting; Prague. 2005 March 20-23; Czech Republic; 2005. 116. LeFevre ML, Ewigman B, Evans JK. Is smoking an indication for prenatal ultrasonography? RADIUS Study Group. Archives of Family Medicine. 1995; 4:120–3. 117. Lilley J, Forster DP. A randomised controlled trial of individual counselling of smokers in pregnancy. Public Health. 1986; 100:309–15. 118. Lillington L, Chlebowski R, Ruvalcaba M, Novak D, Royce J. Evaluation of a smoking cessation program for pregnant minority smokers. Cancer Practice. 1995; 3(3):157–63. [ 119. Linares Scott TJ, Heil SH, Higgins ST, Badger GJ, Bernstein IM. Depressive symptoms predict smoking status among pregnant women. Addictive Behaviours. 2009; 34:705–8. 120. Loeb, BK.; Waage, G.; Bailey, J. Smoking intervention in pregnancy. Proceedings of the Fifth World Conference on Smoking and Health; Winnipeg, Canada. Ottawa. 1983 July; Canadian Council on Smoking and Health; 1983. p. 389-95. 121. Lowe JB, Balanda KP, Stanton WR, Del Mar C, O’Connor V. Dissemination of an efficacious antenatal smoking cessation program in public hospitals in Australia: a randomised controlled trial. Health Education & Behavior. 2002; 29(5):608–19. 122. Lowe JB, Windsor R, Balanda K, Woodby L. Smoking relapse prevention methods for pregnant women: a formative evaluation. American Journal of Health Promotion. 1997; 11:244–6. [ 123. Lussier JP, Heil SH, Mongeon JA, Badger GJ, Higgins ST. A meta-analysis of voucher-based reinforcement therapy for substance use disorders. Addiction. 2006; 101:192–203. 124. Ma Y, Goins KV, Pbert L, Ockene JK. Predictors of smoking cessation in pregnancy and maintenance postpartum in low-income women. Maternal and Child Health Journal. 2005; 9(4):393–402. 125. Malchodi CS, Oncken C, Dornelas EA, Caramanica L, Gregonis E. The effects of peer counselling on smoking cessation and reduction. Obstetrics & Gynecology. 2003101(3):504–10. 126. Manfredi C, Cho YI, Warnecke R, Saunders S, Sullivan M. Dissemination strategies to improve implementation of the PHS smoking cessation guideline in MCH public health clinics: experimental evaluation results and contextual factors. Health Education Research. 2011; 26(2):348–60. 127. Manfredi C, Crittenden KS, Warnecke R, Engler J, Cho YI, Shaligram C. Evaluation of a motivational smoking cessation intervention for women in public health clinics. Preventive Medicine. 1999; 28:51–60. 128. Mayer JP, Todd R, Hawkins B. A randomised evaluation of smoking cessation interventions for pregnant women at a WIC clinic. American Journal of Public Health. 1990; 80:76–7. 129. McBride CM, Baucom DH, Peterson BL, Pollak KI, Palmer C, Westman E, et al. Prenatal and postpartum smoking abstinence: a partner assisted approach. American Journal of Preventive Medicine. 2004; 27(3):232–8. 130. McBride CM, Curry SJ, Grothaus LC, Nelson JC. Partner smoking status and pregnant smokers’ perception of support for and likelihood of smoking cessation. Health Psychology. 1998; 17:63–9. 131. McBride CM, Curry SJ, Lando HA, Pirie PL, Grothaus LC, Nelson JC. Prevention of relapse in women who quit smoking during pregnancy. American Journal of Public Health. 1999; 89:706–11. 132. McLeod D, Benn C, Pullon S, Viccars A, White S, Cookson T, et al. The midwife’s role in facilitating smoking behaviour change during pregnancy. Midwifery. 2003; 19(4):285–97. 133. McLeod D, Pullon S, Benn C, Cookson T, Dowell A, Viccars A, et al. Can support and education for smoking cessation and reduction be provided effectively by midwives within primary maternity care? Midwifery. 2004; 20:37–50. 134. Messimer SR, Henry RC, Hickner JM. A comparison of two antismoking interventions among pregnant women in eleven primary care practices. Journal of Family Practice. 1989; 28(3):283–8. 135. Moore LO, Campbell R, Whelan A, Mills N, Lupton P, Misselbrook E, et al. Self help smoking cessation in pregnancy: cluster randomised controlled trial. BMJ. 2002; 325:1383–6. 136. Moore ML, Meis PJ, Ernest JM, Wells HB, Zaccaro DJ, Terrell T. A randomized trial of nurse intervention to reduce preterm and low birth weight births. Obstetrics & Gynecology. 1998; 91:656–61. 137. Moore ML, Zaccaro DJ. Cigarette smoking, low birth weight, and preterm births in low-income AfricanAmerican women. Journal of Perinatology. 2000; 3:176–80. 138. Morasco BJ, Dornelas EA, Fischer EH, Oncken C, Lando HA. Spontaneous smoking cessation during pregnancy among ethnic minority women: a preliminary investigation. Addictive Behaviors. 2006; 31(2):203–10. 139. Mullen PD, Carbonari JP, Tabak ER, Glenday MC. Improving disclosure of smoking by pregnant women. American Journal of Obstetrics and Gynecology. 1991; 165:409–13. 140. Naughton F, Prevost AT, Gilbert H, Sutton S. Randomized controlled trial evaluation of a tailored leaflet and SMS text message self-help intervention for pregnant smokers (MiQuit). Nicotine and Tobacco Research. 2012; 14(5):569–77. 141. Nowicki P, Gintzig L, Hebel JR, Lathem R, Miller V, Sexton M. Effective smoking intervention during pregnancy. Birth. 1984; 11:217–24. 142. Olds D, Robinson J, O’Brien R, Luckey D, Pettit L, Henderson C, et al. Home visiting by paraprofessionals and by nurses: a randomized, controlled trial. Pediatrics. 2002; 110(3):486–96. 143. Olds DL, Henderson CR, Chamberlin R, Tatelbaum R. Preventing child abuse and neglect: a randomized trial of nurse home visitation. Pediatrics. 1986; 78:65–78. 144. Olds DL, Henderson CR, Tatelbaum R, Chamberlin R. Improving the delivery of prenatal care and outcomes of pregnancy: a randomized trial of nurse home visitation. Pediatrics. 1986; 77:16–28. 145. Olds DL, Henderson CR, Tatelbaum R. Prevention of intellectual impairment in children of women who smoke cigarettes during pregnancy. Pediatrics. 1994; 93:228–33. 146. Ondersma SJ, Chase SK, Svikis DS, Schuster CR. Computer-based brief motivational intervention for perinatal drug use. Journal of Substance Abuse Treatment. 2005; 28:305–12. 147. Ondersma SJ, Svikis DS, Lam PK, Connors-Burge VS, Ledgerwood DM, Hopper JA. A randomized trial of computerdelivered brief intervention and low-intensity contingency management for smoking during pregnancy. Nicotine & Tobacco Research. 2012; Vol. 14(issue 3):351–60. 148. Ondersma, S.; Svikis, DS.; Beatty, JR.; Lockhart, N. A randomized clinical trial of a computer delivered brief intervention for post-partum drug, alcohol, and tobacco use: three-month outcomes. Proceedings of the 73rd Annual Scientific Meeting of the College on Problems of Drug Dependence; Hollywood, Florida. 2011 June 18-23; 2011. 149. Panaretto KS, Mitchell MR, Anderson L, Gilligan C, Buettner P, Larkins SL, et al. Tobacco use and measuring nicotine dependence among urban Indigenous pregnant women. Medical Journal of Australia. 2009; 191(10):554–7. 150. Panjari M, Bell R, Astbury J, Bishop S, Dalais F, Rice G. Women who spontaneously quit smoking in early pregnancy. Australian New Zealand Journal of Obstetrics and Gynaecology. 1997; 37(3):271. 151. Panjari M, Bell R, Bishop S, Astbury J, Rice G, Doery J. A randomized controlled trial of a smoking cessation intervention during pregnancy. Australian and New Zealand Journal of Obstetrics and Gynaecology. 1999; 39(3):312–7. 152. Park ER, Quinn VP, Chang Y, Regan S, Loudin B, Cummins S, et al. Recruiting pregnant smokers into a clinical trial: using a network-model managed care organization versus community-based practices. Preventive Medicine. 2007; 44:223–9. 153. Parker DR, Windsor RA, Roberts MB, Hecht J, Hardy NV, Strolla LO, et al. Feasibility, cost, and cost-effectiveness of a telephone-based motivational intervention for underserved pregnant smokers. Nicotine and Tobacco Research. 2007; 9(10):1043–51. 154. Parker, DR.; Roberts, MB.; Windsor, RA.; Lasater, TM. Telephone-based smoking cessation interventions effective in high-risk, underserved pregnant women. Joint Conference of SRNT and SRNT-Europe; Dublin, Ireland. 2009 April 27-30; 2099. 155. Patten CA, Windsor RA, Renner CC, Enoch C, Hochreiter A, Nevak C, et al. Feasibility of a tobacco cessation intervention for pregnant Alaska Native women. Nicotine & Tobacco Research. 2009; 12(2):79–87. 156. Patten CA. Tobacco cessation intervention during pregnancy among Alaska Native women. Journal of Cancer Education. 2012; 27(Supp 1):S86–90. 157. Pbert L, Ockene JK, Zapka J, Ma Y, Goins KV, Oncken C, et al. A community health center smoking cessation intervention for pregnant and postpartum women. American Journal of Preventive Medicine. 2004; 26(5):377–85. 158. Peterson L, Rosen A, Podedworny T, Kotch J, Handel J. Smoking reduction during pregnancy by a program of self-help and clinical support. Obstetrics & Gynecology. 1992; 79:924–30. 159. Polanska K, Hanke W, Sobala W, Lowe JB. Efficacy and effectiveness of the smoking cessation program for pregnant women. International Journal of Occupational Medicine and Environmental Health. 2004; 17(3):369–77. 160. Polanska K, Hanke W, Sobala W. Smoking relapse one year after delivery among women who quit smoking during pregnancy. International Journal of Occupational Medicine and Environmental Health. 2005; 18(2):159–65. 161. Polanska K, Hanke W, Sobala. Characteristic of the smoking habit among pregnant women on the base of the test “Why am I a smoker?” [Charakterystyka nagogu palenia papierosow wsrod kobiet ciezarnych na podstawie testu Dlaczego pale?]. Przeglad Lekarski. 2005; 62(10):1095–8. 162. Price JH, Snyder FF, Roberts SM, Losh DP, Desmond SM, Krol RA. Comparison of three antismoking interventions among pregnant women in an urban setting: a randomized trial. Psychological Reports. 1991; 68:595–604. 163. Pullon S, McCleod D, Benn C, Viccars A, White S, Cookson T, et al. Smoking cessation in New Zealand: education and resources for use by midwives for women who smoke during pregnancy. Health Promotion International. 2003; 18(4):315–24. 164. Reading AE, Cox DN. The effects of ultrasound on maternal anxiety. Journal of Behavioral Medicine. 1982; 5(2):237–47. 165. Reading AE, Sledmere CM, Cox DNB, Campbell S. Health beliefs and health care behaviour in pregnancy. Psychological Medicine. 1982; 12:379–83. 166. Rigotti N, Park ER, Regan S, Chang Y, Perry K, Loudin B, et al. Efficacy of telephone counseling for pregnant smokers. Obstetrics & Gynecology. 2006; 108(1):83–92. 167. Rigotti NA, Park ER, Chang Y, Regan S. Smoking cessation medication use among pregnant and postpartum smokers. Obstetrics & Gynecology. 2008; 111(2 Pt 1):348–55. 168. Rigotti, N.; Park, E.; Regan, S.; Chang, Y.; Perry, K.; Loudin, B., et al. Efficacy of proactive telephone counseling for pregnant smokers: a randomized trial. Society for Research on Nicotine and Tobacco 12th Annual Meeting; Orlando, Florida, USA. 2006 February 15-18; 2006. p. 22 169. Rigotti, N.; Park, E.; Regan, S.; Chang, Y.; Perry, K.; Loudin, B., et al. The efficacy of telephone couseling for pregnant smokers: a randomized controlled trial [abstract]. 13th World Conference on Tobacco or Health; Washington DC, USA. 2006 July 12-15; 2006 170. Secker-Walker RH, Mead PB, Goodwin GD, Lepage SS, Skelly JM, Flynn BS, et al. Individualised smoking cessation counseling during prenatal and early postnatal care. American Journal of Obstetrics and Gynecology. 1994; 71:1347–55. 171. Secker-Walker RH, Solomon LJ, Flynn BS, LePage SS, Crammond JE, Worden JK, et al. Training obstetric and family practice residents to give smoking cessation advice during prenatal care. American Journal of Obstetrics and Gynecology. 1992; 166:1356–63. 172. Secker-Walker RH, Solomon LJ, Flynn BS, Skelly JM, Lepage SS, Goodwin GD, et al. Smoking relapse prevention counseling during prenatal and early postnatal care. American Journal of Preventive Medicine. 1995; 11(2):86–93. 173. Secker-Walker RH, Solomon LJ, Flynn BS, Skelly JM, Mead PB. Reducing smoking during pregnancy and postpartum: physician’s advice supported by individual counseling. Preventive Medicine. 1998; 27:422–30. 174. Secker-Walker RH, Solomon LJ, Flynn BS, Skelly JM, Mead PB. Smoking relapse prevention during pregnancy. A trial of coordinated advice from physicians and individual counseling. American Journal of Preventive Medicine. 1998; 15:25–31. 175. Secker-Walker RH, Solomon LJ, Geller BM, Flynn BS, Worden JK, Skelly JM, et al. Modeling smoking cessation: exploring the use of a videotape to help pregnant women quit smoking. Women & Health. 1997; 25:23–35. 176. Secker-Walker RH, Vacek PM, Flynn BS, Mead PB. Estimated gains in birth weight associated with reductions in smoking during pregnancy. Journal of Reproductive Medicine. 1998; 43(11):967–74. 177. Secker-Walker RH, Vacek PM, Flynn BS, Mead PB. Smoking in pregnancy, exhaled carbon monoxide, and birth weight. Obstetrics and Gynecology. 1997; 89:648–53. 178. Sexton M, Hebel JR. A clinical trial of change in maternal smoking and its effect on birth weight. JAMA. 1984; 251:911–5. 179. Sexton M, Nowicki P, Hebel JR. Verification of smoking status by thiocyanate in unrefrigerated, mailed saliva samples. Preventive Medicine. 1986; 15(1):28–34. 180. Sierto FW, Hannon WH, Kendrick JS, Bernert JT, Pirkle J, Gargiullo PM. Urinary cotinine levels in women enrolled in a smoking cessation study during and after pregnancy. Journal of Smoking-Related Disease.   1994; 5:65–76.   181. Solomon LJ, Higgins ST, Heil SH, Badger GJ, Thomas CS, Bernstein IM. Predictors of postpartum relapse to smoking. Drug and Alcohol Dependence. 2007; 90:224–7. [ 182. Solomon LJ, Secker-Walker RH, Flynn BS, Skelly JM, Capeless EL. Proactive telephone peer support to help pregnant women stop smoking. Tobacco Control. 2000; 9(Suppl 3):iii72–iii74. 183. Solomon LJ, Secker-Walker RH, Skelly JM, Flynn BS. Stages of change in smoking during pregnancy in low risk women. Journal of Behavioral Medicine. 1996; 19:333–4. 184. Stanton WR, Lowe JB, Moffatt J, DelMar CB. Randomised control trial of a smoking cessation intervention directed at men whose partners are pregnant. Preventive Medicine. 2004; 38:6–9. 185. Stotts AL, DeLaune KA, Schmitz JM, Grabowski J. Impact of a motivational intervention on mechanisms of change in low-income pregnant women. Addictive Behaviors. 2004; 29(8):1649–57. 186. Stotts AL, Groff JY, Velasquez MM, Benjamin-Garner R, Green C, Carbonari JP, et al. Ultrasound feedback and motivational interviewing targeting smoking cessation in the second and third trimesters of pregnancy. Nicotine & Tobacco Research. 2009; 11(8):961–8. 187. Stotts S, DiClemente CC, Dolan-Mullen P. One-to-One: a motivational intervention for resistant pregnant smokers. Addictive Behaviors. 2002; 27:275–92. 188. Stotts, AL.; Schmitz, JM.; Shipley, SL.; Delaune, KA.; Grabowski, J. Impact of a motivational interviewing intervention on mechanisms of change in low-income pregnant smokers (POS4-32) [abstract]. Society for Research on Nicotine and Tobacco 9th Annual Meeting; New Orleans, Louisiana. 2003 February 19-22; 2003. p. 89 189. Strecher VJ, Bishop KR, Bernhardt J, Thorp JM, Cheuvrout B, Potts P. Quits for keeps: tailored smoking cessation guides for pregnancy and beyond. Tobacco Control. 2000; 9(Suppl 3):iii78–iii79. 190. Subramanian S, Katz KS, Rodam M, Gantz MG, El-Khorazaty NM, Johnson A, et al. An integrated randomized intervention to reduce behavioural and psychosocial risks: Pregnancy and neonatal outcomes. Maternal Child Health Journal. 2012; 16:545–54. 191. Svanberg B. Smoking during pregnancy: possibilities of prevention in antenatal care. International Journal of Technology Assessment in Health Care. 1992; 8(Suppl 1):96–100. 192. Tan S, Courtney LP, El-Mohandes AAE, Gantz MG, Blake SM, Thornberry J, et al. Relationships between self-reported smoking, household environmental tobacco smoke exposure and depressive symptoms in a pregnant minority population. Maternal Child Health Journal. 2011; 15:S65–S74. 193. Tappin DM, Lumsden MA, Gilmour WH, Crawford F, McIntyre D, Stone DH, et al. Randomised controlled trial of home based motivational interviewing by midwives to help pregnant smokers quit or cut down. BMJ. 2005; 331:373–7. 194. Tappin DM, Lumsden MA, McIntyre D, McKay C, Gilmour WH, Webber R, et al. A pilot study to establish a randomized trial methodology to test the efficacy of a behavioural intervention. Health Education Research. 2000; 15(4):491–502. 195. Tappin DM, Lumsden MA, McKay C, McIntyre D, Gilmour H, Webber R, et al. The effect of home-based motivational interviewing on the smoking behaviour of pregnant women: a pilot randomised controlled efficacy study. Ambulatory Child Health. 2000; 6(Suppl 1):34–5. 196. Thornton L, Gogan C, McKenna P. The rotunda stop smoking programme [abstract]. Irish Journal of Medical Science. 1998; 167(Suppl 9):28 197. Tsoh JY, Kohn MA, Gerbert B. Promoting smoking cessation in pregnancy with Video Doctor plus provider cueing: a randomized trial. Acta Obstetricia et Gynecologica Scandinavica. 2010; 89(4):515–23. 198. Tuten M, Fitzsimons H, Chisolm MS, Nuzzo PA, Jones HE. Contingent incentives reduce cigarette smoking among pregnant, methadone-maintained women: results of an initial feasibility and efficacy randomized clinical trial. Addiction. 2012; 107(10): 1868–77. 199. Valbo A, Eide T. Smoking cessation in pregnancy: the effect of hypnosis in a randomized study. Addictive Behaviors. 1996; 21:29–35. 200. Valbo A, Nylander G. Smoking cessation in pregnancy: intervention among heavy smokers. Acta Obstetricia et Gynecologica Scandinavica. 1994; 73:215–9. 201. Victora CG, Langer A, Barros F, Belizan J, Farnot U, Villar J, et al. The Latin American Multicenter Trial on psychosocial support during pregnancy: methodology and baseline comparability. Controlled Clinical Trials. 1994; 15:379–94 202. Vilches, P. Doctoral Thesis. Univesidad di Malaga; 2009. Consumption of tobacco in pregnant women: Proposal of a psychological intervention model in the public health system of Andalucia. [Consumo de tabaco en mujeres gestantes: propuesta de un modelo de intervención psicológica en el sistema sanitario público de Andalucía]. 203. Villar J, Farnot U, Barros F, Victora C, Langer A, Belizan JM. A randomized trial of psychosocial support during high-risk pregnancies. New England Journal of Medicine. 1992; 327:1266–71. 204. Walsh RA, Melmeth A, Byrne JM, Brinsmead MW, Redman S. A smoking cessation program at a public antenatal clinic. American Journal of Public Health. 1997; 87:1201–4. 205. Walsh RA, Redman S, Byrne JM, Melmeth A, Brinsmead MW. Process measures in an antenatal smoking cessation trial: another part of the picture. Health Education Research. 2000; 15(4):469–83. 206. Washio Y, Higgins ST, Heil SH, Badger GJ, Skelly J, Bernstein IM, et al. Examining maternal weight gain during contingency-management treatment for smoking cessation among pregnant women. Drug and Alcohol Dependence. 2011; 114(1):73–6. 207. Windsor R, Woodby L, Miller T, Hardin M. Effectiveness of Smoking Cessation and Reduction in Pregnancy Treatment (SCRIPT) methods in Medicaidsupported prenatal care: Trial III. Health Education & Behavior. 2011; 38(4):412–22. 208. Windsor RA, Contreras L, Artz L, Lowe JB. Smoking cessation and pregnancy intervention trial: preliminary mid-trial results. Progress in Clinical and Biological Research. 1990; 339:107–17. 209. Windsor RA, Li CQ, Boyd NR, Hartmann KE. The use of significant reduction rates to evaluate health education methods for pregnant smokers: a new harm reduction behavioural indicator. Health Education and Behavior. 1999; 26:648–61. 210. Windsor RA, Lowe JB, Perkins LL, Smith-Yoder D, Artz L, Crawford M, et al. Health education for pregnant smokers: its behavioral impact and cost benefit. American Journal of Public Health. 1993; 83:201–6. 211. Windsor RA, Spanos D, Samuelsson C, Bartlett EE, Manzella B, Reese Y, et al. The effectiveness of smoking cessation methods for smokers in public health maternity clinics: a randomized trial. American Journal of Public Health. 1985; 75:1389–92. 212. Windsor RA, Warner KE, Cutter GR. A cost-effectiveness analysis of self-help smoking cessation methods for pregnant women. Public Health Reports. 1988; 103(1):83–8. 213. Windsor RA. The efficacy and cost-effectiveness of smoking cessation methods for pregnant women. Southern Medical Journal. 1986; 79:34.* 214. Yoon JH, Higgins ST, Heil SH, Sugarbaker RJ, Thomas CS, Badger GJ. Delay discounting predicts postpartum relapse to cigarette smoking among pregnant women. Experimental and Clinical Psychopharmacology. 2007; 15(2):176–86. 215. Zapka J, Goins KV, Pbert L, Ockene JK. Translating efficacy research into effectiveness studies in practice: lessons from research to promote smoking cessation in community health centers. Health Promotion Practice. 2004; 5(3):245–55. 216. Zapka JG, Pbert L, Stoddard AM, Ockene JK, Goins KV, Bonollo D. Smoking cessation counseling with pregnant and postpartum women: a survey of community health center providers. American Journal of Public Health. 2000; 90:78–84. |
| Chamberlain *et al.* 2017 | 1. Albrecht S, Cassidy B, Salamie D, Reynolds M. What’s happening A smoking cessation intervention for pregnant adolescents: implications for nurse practitioners. Journal of American Academy of Nurse Practitioners 199911(4):155–9 2. Albrecht S, Stone CA, Payne L, Reynolds MD. A preliminary study of the use of peer support in smoking cessation programs for pregnant adolescents. Journal of the American Academy of Nurse Practitioners 199810:119–25 3. Albrecht SA, Caruthers D, Patrick T, Reynolds M, Salamie D, Higgins LW, et al. A randomised controlled trial of a smoking cessation intervention for pregnant adolescents. Nursing Research 200655(6):402–10 4. Baric L, MacArthur C, Sherwood M. A study of health education aspects of smoking in pregnancy. International Journal of Health Education 197619(2 Suppl):1–17 5. Bauman 1983  Bauman KE, Koch GG, Dent CW, Bryan ES. The influence of observing carbon monoxide level on cigarette smoking by public prenatal patients. American Journal of Public Health 198373:1089–91 6. Belizan JM, Villar J, Victora C, Farnot U, Langer A, Barros F. Impact of health education during pregnancy on behavior and utilization of health resources. American Journal of Obstetrics and Gynecology 1995173:894–9 7. Bullock L, Everett KD, Mullen PD, Geden E, Longo DR, Madsen R. Baby BEEP: A randomized controlled trial of nurses’ individualized social support for poor rural pregnant smokers. Maternal and Child Health Journal 200913(3): 395–406 8. Bullock LF, Everett KD, Mullen PD. Baby beep: a randomized clinical trial of smoking cessation for lowincome rural pregnant women using nurse-delivered social support. Annals of Behavioral Medicine 200835:S99 9. Bullock LF, Hornblow AR, Duff GB, Wells JE. Telephone support for pregnant women: outcome in late pregnancy. New Zealand Medical Journal 1995108:476–8 10. Burling TA, Bigelow GE, Robinson JC, Mead AM. Smoking during pregnancy: reduction via objective assessment and directive advice. Behavior Therapy 199122:31–40 11. Byrd JC, Meade CD. Smoking cessation among pregnant women in an urban setting. Wisconsin Medical Journal 199392:609–12 12. Campbell E, Walsh RA, Sanson-Fisher R, Burrows S, Stojanovski E. A group randomised trial of two methods for disseminating a smoking cessation programme to public antenatal clinics: effects on patient outcomes. Tobacco Control 200615(2):97–102 13. Cinciripini 2000  Blalock JA, Fouladi RT, Wetter DW, Cinciripini PM. Depression in pregnant women seeking smoking cessation treatment. Addictive Behaviours 200530(6):1195–208 14. Cinciripini PM, Blalock JA, Minnix JA, Robinson JD, Brown VL, Lam C, et al. Effects of an intensive depression focused intervention for smoking cessation in pregnancy. Journal of Consulting & Clinical Psychology 201078(1): 44–54 15. Cook C, Ward S, Myers S, Spinnato J. A prospective, randomized evaluation of intensified therapy for smoking reduction in pregnancy.  American Journal of Obstetrics and Gynecology 1995172:290 16. Cope GF, Nayyar P, Holder R. Feedback from a point of-care test for nicotine intake to reduce smoking during pregnancy. Annals of Clinical Biochemistry 2003.40(Pt 6): 674–9 17. Donatelle RJ, Prows SL, Champeau D, Hudson D. Randomised controlled trial using social support and financial incentives for high risk pregnant smokers: Significant Other Supporter (SOS) program. Tobacco Control 2000.9 Suppl 3:iii67–iii69 18. Donovan J. Randomised controlled trial of anti-smoking advice in pregnancy. British Journal of Preventive and Social Medicine 1977.31(1):6–12 19. Dornelas EA, Magnavita J, Beazoglou T, Fischer EH, Oncken C, Lando H, et al. Efficacy and cost-effectiveness of a clinic-based counseling intervention tested in an ethnically diverse sample of pregnant smokers. Patient Education and Counseling 2006.64(1-3):342–9 20. Dunkley J. Training midwives to help pregnant women stop smoking  Nursing Times 1997.93(5):64–6 21. Eades SJ, Sanson-Fisher RW, Wenitong M, Panaretto K, D’Este C, Gilligan C, et al. An intensive smoking intervention for pregnant Aboriginal and Torres Strait Islander women: a randomised controlled trial. Medical Journal of Australia 2012.197(1):42–6 22. El-Mohandes AA, El-Khorazaty MN, Kiely M, Gantz MG. Smoking cessation and relapse among pregnant African-American smokers in Washington, DC. Maternal & Child Health Journal 2011.15 Suppl 1:S96–S105 23. Ershoff DH, Quinn VP, Boyd NR, Stern J, Gregory M, Wirtschafter D. The Kaiser Permanente prenatal smoking cessation trial. When more isn’t better, what is enough? American Journal of Preventive Medicine 1999.17(3):161–8 24. Ershoff DH, Quinn VP, Mullen PD. A randomized trial of a serialized self-help smoking cessation program for pregnant women in an HMO. American Journal of Public Health 1989.79(2):182–7 25. Gielen AC, Windsor R, Faden RR, O’Campo P, Repke J, Davis M.  Evaluation of a smoking cessation intervention for pregnant women in an urban prenatal clinic. Health Education Research 1997.12(2):247–54 26. Glover M, Kira A, Walker N, Bauld L. Using incentives to encourage smoking abstinence among pregnant indigenous women? A feasibility study. Maternal and Child Health Journal 2014.19(6):1393–9 27. Graham AV, Reeb KG, Kitson GC, Zyzanski SJ, Frank SH. A clinical trial to reduce the rate of low birth weight in an inner-city black population. Family Medicine 1992.24: 439–46 28. Haddow JE, Wald NJ, Palomaki GE, Kloza EM, Knight GJ. Cotinine-assisted intervention in pregnancy to reduce smoking and low birthweight delivery.  British Journal of Obstetrics and Gynaecology 1991.98(9):859–65 29. Hajek P, West R, Lee A, Foulds J, Owen L, Eiser JR, et al. Randomized controlled trial of a midwife-delivered brief smoking cessation intervention in pregnancy. Addiction 2001.96(3):485–94 30. Harris M, Reynolds B. A pilot study of home-based smoking cessation programs for rural, Appalachian, pregnant smokers. Journal of Obstetric, Gynecologic, and Neonatal Nursing 2015 [EPub ahead of print] 31. Hartmann KE, Koch MA, Pahel-Short L, Thorp JM. A randomized controlled trial of smoking cessation intervention in pregnancy in an academic clinic. Obstetrics & Gynecology 1996.87:621–6 32. Haug K, Fugelli P, Aaro LE, Foss OP. Is smoking intervention in general practice more successful among pregnant than non-pregnant women? Family Practice 1994 11:111–6 33. Haug NA, DiClemente C, Svikis DS. Motivational enhancement therapy for nicotine dependence in methadone-maintained pregnant women. Psychology of Addictive Behaviours 2004.18(3):289–92 34. Hegaard H, Hjaergaard H, Moller L, Wachmann H, Ottesen B. Multimodel intervention raises smoking cessation rate during pregnancy. Acta Obstetricia et Gynecologica Scandinavica 2003.82:813–9 35. Heil SH, Higgins ST, Bernstein IM, Solomon LJ, Rogers RE, Thomas CS, et al. Effects of voucher-based incentives on abstinence from cigarette smoking and fetal growth among pregnant women. Addiction. 2008.103(6):1009–18 36. Hennrikus D, Pirie P, Hellerstedt W, Lando HA, Steele J, Dunn C. Increasing support for smoking cessation during pregnancy and postpartum: results of a randomized controlled pilot study. Preventive Medicine 2010.50(3): 134–7 37. Herbec A, Brown J, Tombor I, Michie S, West R. Pilot randomized controlled trial of an internet-based smoking cessation intervention for pregnant smokers (’MumsQuit’). Drug & Alcohol Dependence 2014.140:130–6 38. Hiett A, Brazus S, Hedberg J, Brown H. Smoking cessation program effectiveness during pregnancy. American Journal of Obstetrics and Gynecology 2000.182(1 Pt 2):S150 39. Higgins ST, Washio Y, Lopez AA, Heil SH, Solomon LJ, Lynch ME, et al. Examining two different schedules of financial incentives for smoking cessation among pregnant women. Preventive Medicine 2014 Vol 68:51–7 40. Hjalmarson AIM, Svanberg B, Hahn L. Stopping smoking in pregnancy: effect of a self-help manual in a controlled trial. British Journal of Obstetrics and Gynaecology 1991.98: 260–4 41. Hughes E, Lamont D, Beecroft M, Wilson D. Randomized trial of a “stage-of-change” orientated smoking cessation intervention in infertile and pregnant women. Fertility and Sterility 2000.74(3):498–503 42. Kemp L, Harris E, McMahon C, Matthey S, Vimpani G, Anderson T, Schmied V, Aslam H, Zapart S. Child and family outcomes of a long-term nurse home visitation programme: a randomised controlled trial. Archives of Disease in Childhood 2011.96(6):533–540 43. Kendrick JS, Metzger RW, Sexton M, Spierto FW, Floyd RL, Gargiullo PM, et al. Integrating smoking cessation into routine public prenatal care: the Smoking Cessation in Pregnancy project. American Journal of Public Health 1995 85:217–22 44. Lawrence T, Aveyard P, Croghan E. What happens to women’s self-reported cigarette consumption and urinary cotinine levels in pregnancy? Addiction 2003.98:1315–20 45. Lee M, Miller SM, Wen KY, Hui SA, Roussi P, Hernandez E. Cognitive-behavioral intervention to promote smoking cessation for pregnant and postpartum inner city women. Journal of Behavioral Medicine 2015.38(6):923–43 46. LeFevre ML, Ewigman B, Evans JK. Is smoking an indication for prenatal ultrasonography? RADIUS Study Group. Archives of Family Medicine 1995.4:120–3 47. Lilley J, Forster DP. A randomised controlled trial of individual counselling of smokers in pregnancy. Public Health 1986.100:309–15 48. Lillington L, Chlebowski R, Ruvalcaba M, Novak D, Royce J. Evaluation of a smoking cessation program for pregnant minority smokers. Cancer Practice 1995.3(3):157–63 49. Loeb BK, Waage G, Bailey J. Smoking intervention in pregnancy. Proceedings of the Fifth World Conference on Smoking and Health 1983 July Winnipeg, Canada. 1983: 389–95 50. Loukopoulou A, Vardavas C, Tzatzarakis M, Farmakides G, Rossolymos C, Chrelias C, et al. Urinary nicotine and cotinine as a method for assessing the effectiveness of an intervention to aid smoking cessation during pregnancy. Chest 2012.142(4 Suppl 1):1081A 51. Lowe JB, Balanda KP, Stanton WR, Del Mar C, O’Connor V. Dissemination of an efficacious antenatal smoking cessation program in public hospitals in Australia: a randomised controlled trial. Health Education & Behavior 2002.29(5):608–19 52. Lowe JB, Windsor R, Balanda K, Woodby L. Smoking relapse prevention methods for pregnant women: a formative evaluation. American Journal of Health Promotion 1997.11:244–6 53. Malchodi CS, Oncken C, Dornelas EA, Caramanica L, Gregonis E. The effects of peer counselling on smoking cessation and reduction. Obstetrics & Gynecology 2003.101 (3):504–10 54. Manfredi C, Crittenden KS, Warnecke R, Engler J, Cho YI, Shaligram C. Evaluation of a motivational smoking cessation intervention for women in public health clinics. Preventive Medicine 1999.28:51–60 55. Mauriello L, Dyment S, Prochaska J, Gagliardi A, Weingrad-Smith J. Acceptability and feasibility of a multiple-behaviour, computer-tailored intervention for underserved pregnant women. Journal of Midwifery and Women’s Health 2011.56:75–80 56. Mayer JP, Todd R, Hawkins B. A randomised evaluation of smoking cessation interventions for pregnant women at a WIC clinic. American Journal of Public Health 1990.80: 76–7 57. McBride CM, Baucom DH, Peterson BL, Pollak KI, Palmer C, Westman E, et al. Prenatal and postpartum smoking abstinence: a partner assisted approach. American Journal of Preventive Medicine 2004.27(3):232–8 58. McBride CM, Curry SJ, Lando HA, Pirie PL, Grothaus LC, Nelson JC. Prevention of relapse in women who quit smoking during pregnancy. American Journal of Public Health 1999.89:706–11 59. McLeod D, Pullon S, Benn C, Cookson T, Dowell A, Viccars A, et al. Can support and education for smoking cessation and reduction be provided effectively by midwives within primary maternity care? Midwifery 2004.20:37–50 60. Mejdoubi J, van den Heijkant SCCM, van Leerdam FJM, Crone M, Crijnen A, HiraSing RA. Effects of nurse home visitation on cigarette smoking pregnancy outcomes: a randomized controlled trial. Midwifery 2014.30:688–95 61. Messimer SR, Henry RC, Hickner JM. A comparison of two antismoking interventions among pregnant women in eleven primary care practices. Journal of Family Practice 1989.28(3):283–8 62. Moore LO, Campbell R, Whelan A, Mills N, Lupton P, Misselbrook E, et al. Self help smoking cessation in pregnancy: cluster randomised controlled trial. BMJ 2002 325:1383–6 63. Moore ML, Meis PJ, Ernest JM, Wells HB, Zaccaro DJ, Terrell T. A randomized trial of nurse intervention to reduce preterm and low birth weight births. Obstetrics & Gynecology 1998.91:656–61 64. Naughton F, Prevost AT, Gilbert H, Sutton S. Randomized controlled trial evaluation of a tailored leaflet and SMS text message self-help intervention for pregnant smokers (MiQuit). Nicotine and Tobacco Research 2012.14(5): 569–77 65. Olds D, Robinson J, O’Brien R, Luckey D, Pettit L, Henderson C, et al. Home visiting by paraprofessionals and by nurses: a randomized, controlled trial. Pediatrics 2002 110(3):486–96 66. Olds DL, Henderson CR, Chamberlin R, Tatelbaum R. Preventing child abuse and neglect: a randomized trial of nurse home visitation. Pediatrics 1986.78:65–78 67. Ondersma SJ, Svikis DS, Lam PK, Connors-Burge VS, Ledgerwood DM, Hopper JA. A randomized trial of computer-delivered brief intervention and low-intensity contingency management for smoking during pregnancy. Nicotine & Tobacco Research 2012 Vol 14, issue 3: 351–60 68. Oude Wesselink SF, Lingsma HF, Reulings PG, Wentzel HR, Erasmus V, Robben PB, et al. Does government supervision improve stop-smoking counseling in midwifery practices? Nicotine & Tobacco Research 2015 Vol 17, issue 5:572–9 69. Panjari M, Bell R, Bishop S, Astbury J, Rice G, Doery J. A randomized controlled trial of a smoking cessation intervention during pregnancy. Australian and New Zealand Journal of Obstetrics and Gynaecology. 1999.39(3):312–7 70. Parker DR, Windsor RA, Roberts MB, Hecht J, Hardy NV, Strolla LO, et al. Feasibility, cost, and costeffectiveness of a telephone-based motivational intervention for underserved pregnant smokers. Nicotine and Tobacco Research 2007.9(10):1043–51 71. Patten CA, Windsor RA, Renner CC, Enoch C, Hochreiter A, Nevak C, et al. Feasibility of a tobacco cessation intervention for pregnant Alaska Native women. Nicotine & Tobacco Research 2009.12(2):79–87 72. Pbert L, Ockene JK, Zapka J, Ma Y, Goins KV, Oncken C, et al. A community health center smoking cessation intervention for pregnant and postpartum women. American Journal of Preventive Medicine 2004.26(5): 377–85 73. Peterson L, Rosen A, Podedworny T, Kotch J, Handel J. Smoking reduction during pregnancy by a program of selfhelp and clinical support. Obstetrics & Gynecology 1992.79: 924–30 74. Polanska K, Hanke W, Sobala W, Lowe JB. Efficacy and effectiveness of the smoking cessation program for pregnant women. International Journal of Occupational Medicine and Environmental Health 2004.17(3):369–77 75. Pollak KI, Lyna P, Bilheimer A, Farrell D, Gao X, Swamy GK, et al. A pilot study testing SMS text delivered scheduled gradual reduction to pregnant smokers. Nicotine & Tobacco Research 2013.15(10):1773–6 76. Prapavessis H, De Jesus S, Harper T, Cramp A, Fitzgeorge L, Mottola MF, et al. The effects of acute exercise on tobacco cravings and withdrawal symptoms in temporary abstinent pregnant smokers. Addictive Behaviors 2014.39(3):703–8 77. Price JH, Snyder FF, Roberts SM, Losh DP, Desmond SM, Krol RA. Comparison of three antismoking interventions among pregnant women in an urban setting: a randomized trial. Psychological Reports 1991.68:595–604 78. Reading AE, Cox DN. The effects of ultrasound on maternal anxiety. Journal of Behavioral Medicine 1982.5(2): 237–47 79. Rigotti N, Park ER, Regan S, Chang Y, Perry K, Loudin B, et al. Efficacy of telephone counseling for pregnant smokers. Obstetrics & Gynecology 2006.108(1):83–92 80. Robling M, Bekkers MJ, Bell K, Butler CC, CanningsJohn R, Channon S, et al. Effectiveness of a nurse-led intensive home-visitation programme for first-time teenage mothers (Building Blocks): a pragmatic randomised controlled trial. Lancet 2016.387(10014):146–55 81. Secker-Walker RH, Mead PB, Goodwin GD, Lepage SS, Skelly JM, Flynn BS, et al. Individualised smoking cessation counseling during prenatal and early postnatal care. American Journal of Obstetrics and Gynecology 1994 71:1347–55 82. Secker-Walker RH, Solomon LJ, Flynn BS, Skelly JM, Mead PB. Reducing smoking during pregnancy and postpartum: physician’s advice supported by individual counselling. Preventive Medicine 1998.27:422–30 83. Secker-Walker RH, Solomon LJ, Geller BM, Flynn BS, Worden JK, Skelly JM, et al. Modeling smoking cessation: exploring the use of a videotape to help pregnant women quit smoking. Women & Health 1997.25:23–35 84. Sexton M, Hebel JR. A clinical trial of change in maternal smoking and its effect on birth weight. JAMA 1984.251: 911–5 85. Solomon LJ, Secker-Walker RH, Flynn BS, Skelly JM, Capeless EL. Proactive telephone peer support to help pregnant women stop smoking. Tobacco Control 2000.9 Suppl 3:iii72–iii74 86. Stotts AL, DeLaune KA, Schmitz JM, Grabowski J. Impact of a motivational intervention on mechanisms of change in low-income pregnant women. Addictive Behaviors 2004.29 (8):1649–57 87. Stotts AL, Groff JY, Velasquez MM, Benjamin-Garner R, Green C, Carbonari JP, et al. Ultrasound feedback and motivational interviewing targeting smoking cessation in the second and third trimesters of pregnancy. Nicotine & Tobacco Research 2009.11(8):961–8 88. Stotts S, DiClemente CC, Dolan-Mullen P. One-to-One: a motivational intervention for resistant pregnant smokers. Addictive Behaviors 2002.27:275–92 89. Strecher VJ, Bishop KR, Bernhardt J, Thorp JM, Cheuvrout B, Potts P. Quits for keeps: tailored smoking cessation guides for pregnancy and beyond. Tobacco Control 2000.9 (Suppl 3):iii78–iii79 90. Tappin D, Bauld L, Purves D, Boyd K, Sinclair L, MacAskill S, et al. Financial incentives for smoking cessation in pregnancy: randomised controlled trial. BMJ (Clinical Research Ed.) 2015.350:h134 91. Tappin DM, Lumsden MA, Gilmour WH, Crawford F, McIntyre D, Stone DH, et al. Randomised controlled trial of home based motivational interviewing by midwives to help pregnant smokers quit or cut down. BMJ 2005.331: 373–7 92. Tappin DM, Lumsden MA, McIntyre D, McKay C, Gilmour WH, Webber R, et al. A pilot study to establish a randomized trial methodology to test the efficacy of a behavioural intervention. Health Education Research 2000 15(4):491–502 93. Thornton L. Smoking and Pregnancy: Feasibility and Effectiveness of a Smoking Intervention Programme Among Pregnant Women [thesis]. Dublin: Dept of Public Health, 1997 94. Tsoh JY, Kohn MA, Gerbert B. Promoting smoking cessation in pregnancy with Video Doctor plus provider cueing: a randomized trial. Acta Obstetricia et Gynecologica Scandinavica 2010.89(4):515–23 95. Tuten M, Fitzsimons H, Chisolm MS, Nuzzo PA, Jones HE. Contingent incentives reduce cigarette smoking among pregnant, methadone-maintained women: results of an initial feasibility and efficacy randomized clinical trial. Addiction 2012.107(10):1868–77 96. Ussher M, Lewis S, Aveyard P, Manyonda I, West R, Lewis B, et al. Physical activity for smoking cessation in pregnancy: randomised controlled trial. BMJ (Clinical Research Ed.) 2015.350:h2145 97. Valbo A, Eide T. Smoking cessation in pregnancy: the effect of hypnosis in a randomized study. Addictive Behaviors 1996.21:29–35 98. Valbo A, Nylander G. Smoking cessation in pregnancy: intervention among heavy smokers. Acta Obstetricia et Gynecologica Scandinavica 1994.73:215–9 99. Vilches P. Consumption of Tobacco in Pregnant Women: Proposal of a Psychological Intervention Model in the Public Health System of Andalucia [thesis] Malaga: Univesidad di Malaga, 2009 100. Walsh RA, Melmeth A, Byrne JM, Brinsmead MW, Redman S. A smoking cessation program at a public antenatal clinic. American Journal of Public Health 1997.87: 1201–4 101. Wilkinson SA, McIntyre HD. Evaluation of the ’healthy start to pregnancy’ early antenatal health promotion workshop: a randomized controlled trial. BMC Pregnancy and Childbirth 2012.0.590972222222222 102. Windsor R, Woodby L, Miller T, Hardin M. Effectiveness of Smoking Cessation and Reduction in Pregnancy Treatment (SCRIPT) methods in Medicaid-supported prenatal care: Trial III. Health Education & Behavior 2011.38(4):412–22 103. Windsor RA, Lowe JB, Perkins LL, Smith-Yoder D, Artz L, Crawford M, et al. Health education for pregnant smokers: its behavioral impact and cost benefit. American Journal of Public Health 1993.83:201–6 104. Windsor RA, Spanos D, Samuelsson C, Bartlett EE, Manzella B, Reese Y, et al. The effectiveness of smoking cessation methods for smokers in public health maternity clinics: a randomized trial. American Journal of Public Health 1985.75:1389–92 |
| Griffiths *et al.* 2018 | 1. Abroms, L. C., Johnson, P. R., Leavitt, L. E., Cleary, S. D., Bushar, J., Brandon, T. H., & Chiang, S. C. (2017). A randomized trial of text messaging for smoking cessation in pregnant women. American Journal of Preventive Medicine, 53(6), 781–790. doi:10.1016/j.amepre.2017.08.002 2. Cinciripini, P. M., McClure, J. B., Wetter, D. W., Perry, J., Blalock, J. A., Cinciripini, L. G., … Skaar, K. (2000). An evaluation of videotaped vignettes for smoking cessation and relapse prevention during pregnancy: The Very Important Pregnant Smokers (VIPS) program. Tobacco Control, 9(suppl 3), iii61–iii63. doi:10.1136/tc.9.suppl_3.iii61 3. Ershoff, D. H., Quinn, V. P., Boyd, N. R., Stern, J., Gregory, M., & Wirtschafter, D. (1999). The Kaiser Permanente prenatal smoking-cessation trial: When more isn’t better, what is enough? American Journal of Preventive Medicine, 17(3), 161– 168. doi:10.1016/S0749-3797(99)00071-9 4. Harris, M., & Reynolds, B. (2015). A pilot study of home-based smoking cessation programs for rural, Appalachian, pregnant smokers. Journal of Obstetric, Gynecologic, & Neonatal Nursing, 44(2), 236–245. doi:10.1111/1552-6909.12547 5. Herbec, A., Brown, J., Tombor, I., Michie, S., & West, R. (2014). Pilot randomized controlled trial of an internet-based smoking cessation intervention for pregnant smokers (‘MumsQuit’). Drug and Alcohol Dependence, 140, 130–136. doi:10.1016/j.drugalcdep.2014.04.010 6. Lawrence, T., Aveyard, P., Evans, O., & Cheng, K. (2003). A cluster randomised controlled trial of smoking cessation in pregnant women comparing interventions based on the transtheoretical (stages of change) model to standard care. Tobacco Control, 12(2), 168–177. doi:10.1136/tc.12.2.168 7. Naughton, F., Cooper, S., Foster, K., Emery, J., Leonardi-Bee, J., Sutton, S., … Leighton, M. (2017). Large multi-centre pilot randomized controlled trial testing a low-cost, tailored, self-help smoking cessation text message intervention for pregnant smokers (MiQuit). Addiction, 112(7), 1238–1249. doi:10.1093/ntr/ntr254 8. Naughton, F., Prevost, A. T., Gilbert, H., & Sutton, S. (2012). Randomized controlled trial evaluation of a tailored leaflet and SMS text message self-help intervention for pregnant smokers (MiQuit). Nicotine & Tobacco Research, 14(5), 569–577. doi:10.1093/ntr/ntr254 9. Ondersma, S. J., Svikis, D. S., Lam, P. K., Connors-Burge, V. S., Ledgerwood, D. M., & Hopper, J. A. (2012). A randomized trial of computer-delivered brief intervention and low-intensity contingency management for smoking during pregnancy. Nicotine & Tobacco Research, 14(3), 351–360. doi:10.1093/ntr/ntr221 10. Pollak, K. I., Lyna, P., Bilheimer, A., Farrell, D., Gao, X., Swamy, G. K., & Fish, L. J. (2013). A pilot study testing SMS text delivered scheduled gradual reduction to pregnant smokers. Nicotine & Tobacco Research, 15(10), 1773–1776. doi:10.1093/ ntr/ntt045 11. Price, J. H., Desmond, S. M., Roberts, S. M., Krol, R. A., Losh, D. P., & Snyder, F. F. (1991). Comparison of three antismoking interventions among pregnant women in an urban setting: A randomized trial. Psychological Reports, 68(2), 595–604. doi:10.2466/PR0.68.2.595-604 12. Secker-Walker, R. H., Solomon, L. J., Geller, B. M., Flynn, B. S., Worden, J. K., Skelly, J. M., & Mead, P. B. (1997). Modeling smoking cessation: Exploring the use of a videotape to help pregnant women quit smoking. Women & Health, 25(1), 23–35. doi:10.1300/J013v25n01_02 |
| Heminger *et al.* 2016 | 1. Abroms LC, Johnson PR, Heminger CL, et al. Quit4baby: results from a pilot test of a mobile smoking cessation program for pregnant women. JMIR Mhealth Uhealth. 2015;3(1):e10 2. Cooper S, Foster K, Naughton F, et al. Pilot study to evaluate a tailored text message intervention for pregnant smokers (MiQuit): study protocol for a randomised controlled trial. Trials. 2015;16:29. 29 3. Naughton F, Jamison J, Sutton S. Attitudes towards SMS text message smoking cessation support: a qualitative study of pregnant smokers. Health Educ Res. 2013;28(5):911–922. doi: 10.1093/her/cyt057 4. Naughton F, Prevost AT, Gilbert H, Sutton S. Randomized controlled trial evaluation of a tailored leaflet and SMS text message self-help intervention for pregnant smokers (MiQuit). Nicotine Tob Res. 2012; 14(5):569–577. 27 5. Pollack KI, Lyna P, Bilheimer A, et al. A pilot study testing SMS text delivered scheduled gradual reduction to pregnant smokers. Nicotine Tob Res. 2013;15(10):1773–1776 6. van Mierlo T, Fournier R, Jean-Charles A, Hovington J, Ethier I, Selby P. I’ll txt u if i have a problem: how the Société? Canadienne du cancer in Quebec applied behavior-change theory, data mining and agile software development to help young adults quit smoking. PLoS One. 2014;9(3):e91832. 30 |
| Hussain *et al.* 2020 | 1. Abroms LC, Chiang S, Macherelli L, Leavitt L, Montgomery M. Assessing the national cancer institute's SmokefreeMOM text-messaging program for pregnant smokers: pilot randomized trial. J Med Internet Res 2017 Oct 3;19(10):e333 [FREE Full text] [doi: 10.2196/jmir.8411] [Medline: 28974483] 2. Abroms LC, Johnson PR, Heminger CL, van Alstyne JM, Leavitt LE, Schindler-Ruwisch JM, et al. Quit4baby: results from a pilot test of a mobile smoking cessation program for pregnant women. JMIR Mhealth Uhealth 2015 Jan 23;3(1):e10 [FREE Full text] [doi: 10.2196/mhealth.3846] [Medline: 25650765] 3. Abroms LC, Johnson PR, Leavitt LE, Cleary SD, Bushar J, Brandon TH, et al. A randomized trial of text messaging for smoking cessation in pregnant women. Am J Prev Med 2017 Dec;53(6):781-790 [FREE Full text] [doi:10.1016/j.amepre.2017.08.002] [Medline: 28982527] 4. Forinash AB, Yancey A, Chamness D, Koerner J, Inteso C, Miller C, et al. Smoking cessation following text message intervention in pregnant women. Ann Pharmacother 2018 Nov;52(11):1109-1116. [doi: 10.1177/1060028018780448] [Medline: 29857773] 5. Fujioka N, Kobayashi T, Turale S. Short-term behavioral changes in pregnant women after a quit-smoking program via e-learning: a descriptive study from Japan. Nurs Health Sci 2012 Sep;14(3):304-311. [doi: 10.1111/j.1442-2018.2012.00702.x] [Medline: 22950611] 6. Naughton F, Cooper S, Foster K, Emery J, Leonardi-Bee J, Sutton S, et al. Large multi-centre pilot randomized controlled trial testing a low-cost, tailored, self-help smoking cessation text message intervention for pregnant smokers (MiQuit). Addiction 2017 Jul;112(7):1238-1249 [FREE Full text] [doi: 10.1111/add.13802] [Medline: 28239919] 7. Naughton F, Prevost A, Gilbert H, Sutton S. Randomized controlled trial evaluation of a tailored leaflet and SMS text message self-help intervention for pregnant smokers (MiQuit). Nicotine Tob Res 2012 May;14(5):569-577. [doi:10.1093/ntr/ntr254] [Medline: 22311960] 8. Pollak KI, Lyna P, Bilheimer A, Farrell D, Gao X, Swamy GK, et al. A pilot study testing SMS text delivered scheduled gradual reduction to pregnant smokers. Nicotine Tob Res 2013 Oct;15(10):1773-1776 [FREE Full text] [doi:10.1093/ntr/ntt045] [Medline: 23569007] 9. Tombor I, Beard E, Brown J, Shahab L, Michie S, West R. Randomized factorial experiment of components of the SmokeFree Baby smartphone application to aid smoking cessation in pregnancy. Transl Behav Med 2019 Jul 16;9(4):583-593 [FREE Full text] [doi: 10.1093/tbm/iby073] [Medline: 30011020] |
| Su *et al*. 2014 | 1. Albrecht, S. A., Caruthers, D., Patrick, T., et al. (2006). A randomized controlled trial of a smoking cessation intervention for pregnant adolescents. Nursing Research, 55(6), 402–410. 2. Brandon, T. H., Simmons, V. N., Meade, C. D., et al. (2012). Self-help booklets for preventing postpartum smoking relapse: A randomized trial. American Journal of Public Health, 102(11), 2109–2115. 3. Bullock, L., Everett, K. D., Mullen, P. D., Geden, E., Longo, D. R., Madsen, R., et al. (2009). A randomized controlled trial of nurses’ individualized social support for poor rural pregnant smokers. Maternal and Child Health Journal, 13(3), 395–406. 4. Cinciripini, P. M., Blalock, J. A., Minnix, J. A., et al. (2010). Effects of an intensive depression-focused intervention for smoking cessation in pregnancy. Journal of Consulting and Clinical Psychology, 78(1), 44–54. 5. de Vries, H., Bakker, M., Mullen, P. D., & van Breukelen, G. (2006). The effects of smoking cessation counseling by midwives on Dutch pregnant women and their partners. Patient Education and Counseling, 63(1–2), 177–187. 6. Donatelle, R. J., Prows, S. L., Champeau, D., & Hudson, D. (2000). Randomised controlled trial using social support and financial incentives for high risk pregnant smokers: Significant other supporter (SOS) program. Tobacco Control, 9(Suppl 3), III67–III69. 7. El-Mohandes, A. A., El-Khorazaty, M. N., Kiely, M., & Gantz, M. G. (2011). Smoking cessation and relapse among pregnant African-American smokers in Washington, DC. Maternal and Child Health Journal, 15(Suppl 1), S96–S105. 8. Gadomski, A., Adams, L., Tallman, N., Krupa, N., & Jenkins, P. (2011). Effectiveness of a combined prenatal and postpartum smoking cessation program. Maternal and Child Health Journal, 15(2), 188–197. 9. Heil, S. H., Higgins, S. T., Bernstein, I. M., et al. (2008). Effects of voucher-based incentives on abstinence from cigarette smoking and fetal growth among pregnant women. Addiction, 103(6), 1009–1018. 10. Hennrikus, D., Pirie, P., Hellerstedt, W., Lando, H. A., Steele, J., & Dunn, C. (2010). Increasing support for smoking cessation during pregnancy and postpartum: Results of a randomized controlled pilot study. Preventive Medicine, 50(3), 134–137. 11. Higgins, S. T., Heil, S. H., Solomon, L. J., et al. (2004). A pilot study on voucher-based incentives to promote abstinence from cigarette smoking during pregnancy and postpartum. Nicotine & Tobacco Research, 6(6), 1015–1020. 12. Lawrence, T., Aveyard, P., Cheng, K. K., Griffin, C., Johnson, C., & Croghan, E. (2005). Does stage-based smoking cessation advice in pregnancy result in long-term quitters? 18-month postpartum follow-up of a randomized controlled trial. Addiction, 100(1), 107–116. 13. Lillington, L., Royce, J., Novak, D., Ruvalcaba, M., & Chlebowski, R. (1995). Evaluation of a smoking cessation program for pregnant minority women. Cancer Practice, 3(3), 157–163. 14. McBride, C. M., Baucom, D. H., Peterson, B. L., et al. (2004). Prenatal and postpartum smoking abstinence a partner-assisted approach. American Journal of Preventive Medicine, 27(3), 232–238. 15. McBride, C. M., Curry, S. J., Lando, H. A., Pirie, P. L., Grothaus, L. C., & Nelson, J. C. (1999). Prevention of relapse in women who quit smoking during pregnancy. American Journal of Public Health, 89(5), 706–711. 16. Morasco, B. J., Dornelas, E. A., Fischer, E. H., Oncken, C., & Lando, H. A. (2006). Spontaneous smoking cessation during pregnancy among ethnic minority women: A preliminary investigation. Addictive Behaviors, 31(2), 203–210. 17. Mullen, P. D., Quinn, V. P., & Ershoff, D. H. (1990). Maintenance of nonsmoking postpartum by women who stopped smoking during pregnancy. American Journal of Public Health, 80(8), 992–994. 18. O’Connor, A. M., Davies, B. L., Dulberg, C. S., et al. (1992). Effectiveness of a pregnancy smoking cessation program. Journal of Obstetric, Gynecologic, and Neonatal Nursing, 21(5), 385–392. 19. Pbert, L., Ockene, J. K., Zapka, J., et al. (2004). A community health center smoking-cessation intervention for pregnant and postpartum women. American Journal of Preventive Medicine, 26(5), 377–385. 20. Peden, A. R., Rayens, M. K., Hall, L. A., et al. (2008). Nicotine addiction in pregnancy: Preliminary efficacy of a mental health intervention. Addictive Disorders and Their Treatment, 7(4), 179–189. 21. Petersen, L., Handel, J., Kotch, J., Podedworny, T., & Rosen, A. (1992). Smoking reduction during pregnancy by a program of self-help and clinical support. Obstetrics and Gynecology, 79(6), 924–930. 22. Polan´ska, K., Hanke, W., & Sobala, W. (2005). Smoking relapse one year after delivery among women who quit smoking during pregnancy. International Journal of Occupational Medicine and Environmental Health, 18(2), 159–165. 23. Pollak, K. I., Oncken, C. A., Lipkus, I. M., et al. (2007). Nicotine replacement and behavioral therapy for smoking cessation in pregnancy. American Journal of Preventive Medicine, 33(4), 297–305. 24. Reitzel, L. R., Vidrine, J. I., Businelle, M. S., et al. (2010). Preventing postpartum smoking relapse among diverse lowincome women: A randomized clinical trial. Nicotine & Tobacco Research, 12(4), 326–335. 25. Rigotti, N. A., Park, E. R., Regan, S., et al. (2006). Efficacy of telephone counseling for pregnant smokers: A randomized controlled trial. Obstetrics and Gynecology, 108(1), 83–92. 26. Secker-Walker, R. H., Solomon, L. J., Flynn, B. S., et al. (1994). Individualized smoking cessation counseling during prenatal and early postnatal care. American Journal of Obstetrics and Gynecology, 171(5), 1347–1355. 27. Secker-Walker, R. H., Solomon, L. J., Flynn, B. S., Skelly, J. M., & Mead, P. B. (1998). Reducing smoking during pregnancy and postpartum: Physician’s advice supported by individual counseling. Preventive Medicine, 27(3), 422–430. 28. Stotts, A. L., Diclemente, C. C., & Dolan-Mullen, P. (2002). Oneto-one: A motivational intervention for resistant pregnant smokers. Addictive Behaviors, 27(2), 275–292. 29. Valanis, B., Lichtenstein, E., Mullooly, J. P., et al. (2001). Maternal smoking cessation and relapse prevention during health care visits. American Journal of Preventive Medicine, 20(1), 1–8. 30. Wall, M. A., Severson, H. H., Andrews, J. A., Lichtenstein, E., & Zoref, L. (1995). Pediatric office-based smoking intervention: Impact on maternal smoking and relapse. Pediatrics, 96(4 Pt 1), 622–628. 31. Walsh, R. A., Redman, S., Brinsmead, M. W., Byrne, J. M., & Melmeth, A. (1997). A smoking cessation program at a public antenatal clinic. American Journal of Public Health, 87(7), 1201–1204. 32. Wisborg, K., Henriksen, T. B., Jespersen, L. B., & Secher, N. J. (2000). Nicotine patches for pregnant smokers: A randomized controlled study. Obstetrics and Gynecology, 96(6), 967–971 |
| Tahan *et al.* 2023 | 1. Hajek P, West R, Lee A, et al. Randomized controlled trial of a midwife-delivered brief smoking cessation intervention in pregnancy. Addiction 2001;96:485–94. 2. Lawrence T, Aveyard P, Evans O, et al. A cluster randomised controlled trial of smoking cessation in pregnant women comparing interventions based on the transtheoretical (stages of change) model to standard care. Tob Control 2003;12:168–77. 3. Windsor R, Woodby L, Miller T, et al. Effectiveness of smoking cessation and reduction in pregnancy treatment (script) methods in medicaid-supported prenatal care: trial III. Health Educ Behav 2011;38:412–22. 4. Tombor I, Beard E, Brown J, et al. Randomized factorial experiment of components of the smokefree baby smartphone application to aid smoking cessation in pregnancy. Transl Behav Med 2019;9:583–93. 5. Marin-Gomez FX, Garcia-Moreno Marchán R, Mayos-Fernandez A, et al. Exploring efficacy of a serious game (tobbstop) for smoking cessation during pregnancy: randomized controlled trial. JMIR Serious Games 2019;7:e12835. 6. Kurti AN, Tang K, Bolivar HA, et al. Smartphone-Based financial incentives to promote smoking cessation during pregnancy: a pilot study. Prev Med 2020;140:S0091-7435(20)30225-5:106201. 7. Joyce CM, Saulsgiver K, Mohanty S, et al. Remote patient monitoring and incentives to support smoking cessation among pregnant and postpartum Medicaid members: three randomized controlled pilot studies. JMIR Form Res 2021;5:e27801. 8. Pollak KI, Lyna P, Gao X, et al. Efficacy of a Texting program to promote cessation among pregnant smokers: a randomized control trial. Ann Behav Med 2020;22:1187–94. 9. Pollak KI, Lyna P, Bilheimer A, et al. A pilot study testing SMS text delivered scheduled gradual reduction to pregnant smokers. Nicotine Tob Res 2013;15:1773–6. 10. Balmumcu A, Ünsal Atan Ş. Smoking cessation programs for pregnant women: utilizing whatsapp text messaging. J Addict Nurs 2021;32:188–96. 11. Forinash AB, Yancey A, Chamness D, et al. Smoking cessation following text message intervention in pregnant women. Ann Pharmacother 2018;52:1109–16. 12. Coleman T, Clark M, Welch C, et al. Effectiveness of offering tailored text message, self-help smoking cessation support to pregnant women who want information on stopping smoking: miquit3 randomised controlled trial and meta-analysis. Addiction 2022;117:1079–94. 13. Abroms L, Leavitt L, Johnson P, et al. A randomized trial of a textmessaging program to promote smoking cessation in pregnant smokers. Ann Behav Med 2017;51:S1590–1. 14. Naughton F. Delivering “ just-in-time” smoking cessation support via mobile phones: current knowledge and future directions. Nicotine Tob Res 2017;19:379–83. 15. Naughton F, Prevost AT, Gilbert H, et al. Randomized controlled trial evaluation of a tailored leaflet and SMS text message selfhelp intervention for pregnant smokers (miquit). Nicotine Tob Res 2012;14:569–77. 16. Harris M, Reynolds B. A pilot study of home-based smoking cessation programs for rural, appalachian, pregnant smokers. J Obstet Gynecol Neonatal Nurs 2015;44:236–45. 17. Herbec A, Brown J, Tombor I, et al. Pilot randomized controlled trial of an internet-based smoking cessation intervention for pregnant smokers ('mumsquit’). Drug Alcohol Depend 2014;140:130–6. 18. Ondersma SJ, Svikis DS, Lam PK, et al. A randomized trial of computer-delivered brief intervention and low-intensity contingency management for smoking during pregnancy. Nicotine Tob Res 2012;14:351–60. 19. Ershoff DH, Quinn VP, Boyd NR, et al. The Kaiser permanente prenatal smoking-cessation trial: when more is ’'t better, what is enough? Am J Prev Med 1999;17:161–8. 20. Patten CA, Koller KR, Flanagan CA, et al. Biomarker feedback intervention for smoking cessation among Alaska native pregnant women: randomized pilot study. Patient Education and Counseling 2019;102:528–35. 21. Burling TA, Bigelow GE, Robinson JC, et al. Smoking during pregnancy: reduction via objective assessment and directive advice. Behavior Therapy 1991;22:31–40. 22. Bauman KE, Bryan ES, Dent CW, et al. The influence of observing carbon monoxide level on cigarette smoking by public prenatal patients. Am J Public Health 1983;73:1089–91. 23. Thornton L. The rotunda stop smoking programme: smoking and pregnancy: feasability and effectiveness of a smoking intervention programme among pregnant women. 1997. 24. Wesselink SFO, Lingsma HF, Robben PBM, et al. Provision and effect of quit-smoking counselling by primary care midwives. Midwifery 2015;31:986–92. 25. O’Connor AM, Davies BL, Dulberg CS, et al. Effectiveness of a pregnancy smoking cessation program. J Obstet Gynecol Neonatal Nurs 1992;21:385–92. 26. Zhang X, Devasia R, Czarnecki G, et al. Effects of incentive-based smoking cessation program for pregnant women on birth outcomes. Matern Child Health J 2017;21:745–51. 27. Lancaster T, Dobbie W, Vos K, et al. Randomized trial of nurseassisted strategies for smoking cessation in primary care. Br J Gen Pract 1999;49:191–4. 28. Petersen L, Handel J, Kotch J, et al. Smoking reduction during pregnancy by a program of self-help and clinical support. Obstet Gynecol 1992;79:924–30. 29. Everett-Murphy K, Steyn K, Mathews C, et al. The effectiveness of adapted, best practice guidelines for smoking cessation counseling with disadvantaged, pregnant smokers attending public sector antenatal clinics in Cape town, South Africa. Acta Obstet Gynecol Scand 2010;89:478–89. 30. de Vries H, Bakker M, Mullen PD, et al. The effects of smoking cessation counseling by midwives on Dutch pregnant women and their partners. Patient Educ Couns 2006;63:177–87. 31. Øien T, Storrø O, Jenssen JA, et al. The impact of a minimal smoking cessation intervention for pregnant women and their partners on perinatal smoking behaviour in primary health care: a real-life controlled study. BMC Public Health 2008;8:325. 32. Ferreira-Borges C. Effectiveness of a brief counseling and behavioral intervention for smoking cessation in pregnant women. Prev Med 2005;41:295–302. 33. McLeod D, Pullon S, Benn C, et al. Can support and education for smoking cessation and reduction be provided effectively by midwives within primary maternity care? Midwifery 2004;20:37–50. 34. Wisborg K, Henriksen TB, Secher NJ. A prospective intervention study of stopping smoking in pregnancy in a routine antenatal care setting. Br J Obstet Gynaecol 1998;105:1171–6. 35. Kendrick JS, Zahniser SC, Miller N, et al. Integrating smoking cessation into routine public prenatal care: the smoking cessation in pregnancy project. Am J Public Health 1995;85:217–22. 36. Ruger JP, Weinstein MC, Hammond SK, et al. Cost-Effectiveness of motivational interviewing for smoking cessation and relapse prevention among low-income pregnant women: a randomized controlled trial. Value Health 2008;11:191–8. 37. Robling M, Bekkers M-J, Bell K, et al. Effectiveness of a nurse-led intensive home-visitation programme for first-time teenage mothers (building blocks): a pragmatic randomised controlled trial. Lancet 2016;387:146–55. 38. Mejdoubi J, van den Heijkant S, van Leerdam FJM, et al. Effects of nurse home visitation on cigarette smoking, pregnancy outcomes and breastfeeding: a randomized controlled trial. Midwifery 2014;30:688–95 39. Tappin DM, Lumsden MA, Gilmour WH, et al. Randomised controlled trial of home based motivational interviewing by midwives to help pregnant smokers quit or cut down. BMJ 2005;331:373–7. 40. Belizán JM, Barros F, Langer A, et al. Impact of health education during pregnancy on behavior and utilization of health resources. American Journal of Obstetrics and Gynecology 1995;173:894–9. 41. Loukopoulou AN, Vardavas CI, Farmakides G, et al. Counselling for smoking cessation during pregnancy reduces tobacco-specific nitrosamine (NNAL) concentrations: a randomized controlled trial. Eur J Midwifery 2018;2:14. 42. Alemán A, Morello P, Colomar M, et al. Brief counseling on secondhand smoke exposure in pregnant women in Argentina and Uruguay. Int J Environ Res Public Health 2016;14:28. 43. Edwards MJ, Geiser T, Chafin C, et al. S.m.a.r.t. mothers are resisting tobacco: prenatal smoking cessation in WIC mothers. J Allied Health 2009;38:170–6. 44. Gebauer C, Kwo CY, Haynes EF, et al. A nurse-managed smoking cessation intervention during pregnancy. J Obstet Gynecol Neonatal Nurs 1998;27:47–53. 45. Avidano Britton GR, Brinthaupt J, Stehle JM, et al. The effectiveness of a nurse-managed perinatal smoking cessation program implemented in a rural County. Nicotine Tob Res 2006;8:13–28. 46. Valanis B, Lichtenstein E, Mullooly JP, et al. Maternal smoking cessation and relapse prevention during health care visits. Am J Prev Med 2001;20:1–8. 47. Lawrence T, Aveyard P, Cheng KK, et al. Does stage-based smoking cessation advice in pregnancy result in long-term quitters? 18-month postpartum follow-up of a randomized controlled trial. Addiction 2005;100:107–16. 48. Joseph JG, El-Mohandes AAE, Kiely M, et al. Reducing psychosocial and behavioral pregnancy risk factors: results of a randomized clinical trial among high-risk pregnant African American women. Am J Public Health 2009;99:1053–61. 49. Albrecht SA, Caruthers D, Patrick T, et al. A randomized controlled trial of a smoking cessation intervention for pregnant adolescents. Nurs Res 2006;55:402–10. 50. Hegaard HK, Kjaergaard H, Møller LF, et al. Multimodal intervention raises smoking cessation rate during pregnancy. Acta Obstet Gynecol Scand 2003;82:813–9. 51. Hayes CB, Collins C, O’Carroll H, et al. Effectiveness of motivational interviewing in influencing smoking cessation in pregnant and postpartum disadvantaged women. Nicotine Tob Res 2013;15:969–77. 52. Abroms LC, Johnson PR, Leavitt LE, et al. A randomized trial of text messaging for smoking cessation in pregnant women. Am J Prev Med 2017;53:781–90. 53. Naughton F, Cooper S, Foster K, et al. Large multi-centre pilot randomized controlled trial testing a low-cost, tailored, self-help smoking cessation text message intervention for pregnant smokers (miquit). Addiction 2017;112:1238–49. 54. Walsh RA, Redman S, Brinsmead MW, et al. A smoking cessation program at a public antenatal clinic. Am J Public Health 1997;87:1201–4. 55. Bullock L, Everett KD, Mullen PD, et al. Baby BEEP: a randomized controlled trial of nurses’ individualized social support for poor rural pregnant smokers. Matern Child Health J 2009;13:395–406. 56. Eades SJ, Sanson-Fisher RW, Wenitong M, et al. An intensive smoking intervention for pregnant Aboriginal and Torres Strait Islander women: a randomised controlled trial. Med J Aust 2012;197:42–6 |
| Wilson *et al.* 2018 | 1. Bradizza, C. M., Stasiewicz, P. R., Zhuo, Y., Ruszczyk, M., Maisto, S. A., Lucke, J. F., Giarratano, P. (2017). Smoking cessation for pregnant smokers: Development and and pilot test of an emotion regulation treatment supplement to standard smoking cessation for negative affect smokers. Nicotine & Tobacco Research, 19(5), 578–584 2. Bullock, L., Everett, K. D., Mullen, P. D., Geden, E., Longo, D. R., & Madsen, R. (2009). Baby BEEP: A randomized controlled trial of nurses’ individualized social support for poor rural pregnant smokers. Maternal and Child Health Journal, 13(3), 395–406 3. Cinciripini, P. M., Blalock, J. A., Minnix, J. A., Robinson, J. D., Brown, V. L., Lam, C., . Karam-Hage, M. (2010). Effects of an intensive depression-focused intervention for smoking cessation in pregnancy. Journal of Consulting and Clinical Psychology, 78(1), 44–54. 4. Donatelle, R. J., Prows, S. L., Champeau, D., & Hudson, D. (2000). Randomised controlled trial using social support and financial incentives for high risk pregnant smokers: significant other supporter (SOS) program. Tobacco Control, 9(Suppl 3), iii67–iii69 5. Dornelas, E. A., Magnavita, J., Beazoglou, T., Fischer, E. H., Oncken, C., Lando, H., . Gregonis, E. (2006). Efficacy and cost-effectiveness of a clinic-based counseling intervention tested in an ethnically diverse sample of pregnant smokers. Patient Education and Counseling, 64(1-3), 342– 349 6. Ershoff, D. H., Quinn, V. P., Boyd, N. R., Stern, J., Gregory, M., & Wirtschafter, D. (1999). The Kaiser Permanente prenatal smoking-cessation trial: when more isn’t better, what is enough? American Journal of Preventive Medicine, 17(3), 161–168. 7. Heil, S. H., Higgins, S. T., Bernstein, I. M., Solomon, L. J., Rogers, R. E., Thomas, C. S., . Lynch, M. E. (2008). Effects of voucher-based incentives on abstinence from cigarette smoking and fetal growth among pregnant women. Addiction, 103(6), 1009–1018 8. Higgins, S. T., Washio, Y., Lopez, A. A., Heil, S. H., Solomon, L. J., Lynch, M. E., . Bernstein, I. M. (2014). Examining two different schedules of financial incentives for smoking cessation among pregnant women. Preventive Medicine, 68, 51–57. 9. Lee, M., Miller, S. M., Wen, K. Y., Hui, S. K., Roussi, P., & Hernandez, E. (2015). Cognitive-behavioral intervention to promote smoking cessation for pregnant and postpartum inner city women. Journal of Behavioral Medicine, 38, 932–943 10. McBride, C. M., Baucom, D. H., Peterson, B. L., Pollak, K. I., Palmer, C., Westman, E., & Lyna, P. (2004). Prenatal and postpartum smoking abstinence a partnerassisted approach. American Journal of Preventive Medicine, 27(3), 232–238. 11. Ondersma, S. J., Svikis, D. S., Lam, P. K., Connors-Burge, V. S., Ledgerwood, D. M., & Hopper, J. A. (2012). A randomized trial of computer-delivered brief intervention and low-intensity contingency management for smoking during pregnancy. Nicotine & Tobacco Research, 14(3), 351–360. 12. Panjari, M., Bell, R., Bishop, S., Astbury, J., Rice, G., & Doery, J. (1999). A randomized controlled trial of a smoking cessation intervention during pregnancy. Australian & New Zealand Journal of Obstetrics & Gynaecology, 39(3), 312–317. 13. Rigotti, N. A., Park, E. R., Regan, S., Chang, Y., Perry, K., Loudin, B., & Quinn, V. (2006). Efficacy of telephone counseling for pregnant smokers: A randomized controlled trial. Obstetrics and Gynecology, 108(1), 83–92. 14. Ruger, J. P., Weinstein, M. C., Hammond, S. K., Kearney, M. H., & Emmons, K. M. (2008). Cost-effectiveness of motivational interviewing for smoking cessation and relapse prevention among low-income pregnant women: a randomized controlled trial. Value in Health, 11(2), 191–198. 15. Secker-Walker, R. H., Solomon, L. J., Flynn, B. S., Skelly, J. M., & Mead, P. B. (1998). Reducing smoking during pregnancy and postpartum: Physician’s advice supported by individual counseling. Preventive Medicine, 27(3), 422–430. 16. Secker-Walker, R. H., Solomon, L. J., Flynn, B. S., Skelly, J. M., Lepage, S. S., Goodwin, G. D., & Mead, P. B. (1994). Individualized smoking cessation counseling during prenatal and early postnatal care. American Journal of Obstetrics and Gynecology, 171(5), 1347–1355 17. Stotts, A. L., DiClemente, C. C., & Dolan-Mullen, P. (2002). One-to-one: A motivational intervention for resistant pregnant smokers. Addictive Behaviors, 27(2), 275–292 18. Stotts, A. L., Groff, J. Y., Velasquez, M. M., Benjamin-Garner, R., Green, C., Carbonari, J. P., & DiClemente, C. C. (2009). Ultrasound feedback and motivational interviewing targeting smoking cessation in the second and third trimesters of pregnancy. Nicotine & Tobacco Research, 11(8):961-968 19. Tappin, D. M., Bauld, L., Purves, D., Boyd, K., Sinclair, L., MacAskill, S., . Coleman, T. (2015). Financial incentives for smoking cessation in pregnancy: Randomised controlled trial. BMJ, 350, h134. 20. Tappin, D. M., Lumsden, M. A., Gilmour, W. H., Crawford, F., McIntyre, D., Stone, D. H., . Mohammed, E. (2005). Randomised controlled trial of home based motivational interviewing by midwives to help pregnant smokers quit or cut down. BMJ, 331, 373–377 21. Tappin, D. M., Lumsden, M. A., McIntyre, D., McKay, C., Gilmour, W. H., Webber, R., . Currie, F. (2000). A pilot study to establish a randomized trial methodology to test the efficacy of a behavioural intervention. Health Education Research, 15(4), 491–502 22. Tuten, M., Fitzsimons, H., Chisolm, M. S., Nuzzo, P. A., & Jones, H. E. (2012). Contingent incentives reduce cigarette smoking among pregnant, methadone-maintained women: Results of an initial feasibility and efficacy randomized clinical trial. Addiction, 107(10), 1868–1877. |

**Unique publications included in smoking reviews**

**Smoking unique publications**

1. Abroms LC, JohnsAbroms LC, Chiang S, Macherelli L, Leavitt L, Montgomery M. Assessing the national cancer institute's SmokefreeMOM text-messaging program for pregnant smokers: pilot randomized trial. J Med Internet Res 2017 Oct 3;19(10):e333 [FREE Full text] [doi: 10.2196/jmir.8411] [Medline: 28974483]
2. Abroms LC, Johnson PR, Heminger CL, et al. Quit4baby: results from a pilot test of a mobile smoking cessation program for pregnant women. JMIR Mhealth Uhealth. 2015;3(1):e10
3. Abroms, L. C., Johnson, P. R., Leavitt, L. E., Cleary, S. D., Bushar, J., Brandon, T. H., & Chiang, S. C. (2017). A randomized trial of text messaging for smoking cessation in pregnant women. American Journal of Preventive Medicine, 53(6), 781–790. doi:10.1016/j.amepre.2017.08.002
4. Albrecht S, Cassidy B, Salamie D, Reynolds M. What’s happening A smoking cessation intervention for pregnant adolescents: implications for nurse practitioners. Journal of American Academy of Nurse Practitioners 199911(4):155–9
5. Albrecht S, Cornelius M, Braxter B, Reynolds M, Stone C, Cassidy B. An assessment of nicotine dependence among pregnant adolescents. Journal of Substance Abuse Treatment. 1999; 16(4): 337–44.
6. Albrecht S, Stone CA, Payne L, Reynolds MD. A preliminary study of the use of peer support in smoking cessation programs for pregnant adolescents. Journal of the American Academy of Nurse Practitioners 199810:119–25
7. Albrecht SA, Caruthers D, Patrick T, Reynolds M, Salamie D, Higgins LW, et al. A randomised controlled trial of a smoking cessation intervention for pregnant adolescents. Nursing Research 200655(6):402–10
8. Albrecht SA, Caruthers D. Characteristics of inner-city pregnant Smoking teenagers. Journal of Obstetric Gynecologic and Neonatal Nursing. 2002; 31:462–9.
9. Albrecht SA, Higgins LW, Lebow H. Knowledge about the deletrious effects of smoking and its relationship to smoking cessation among pregnant adolescents. Adolescence. 2000; 35(140):709– 16.
10. Albrecht SA, Higgins LW, Stone C. Factors relating to pregnant adolescents’ decisions to complete a smoking cessation intervention. Journal of Pediatric Nursing. 1999; 14(5):322–8.
11. Albrecht, SA.; Patrick, T.; Kim, Y.; Caruthers, D. A randomised controlled trial of a smoking cessation intervention for pregnant adolescents. Society for Research on Nicotine and Tobacco 9th Annual Meeting; New Orleans, Louisiana. 2003 February 19-23; 2003. p. 91
12. Alemán A, Morello P, Colomar M, et al. Brief counseling on secondhand smoke exposure in pregnant women in Argentina and Uruguay. Int J Environ Res Public Health 2016;14:28.
13. Allen S.S., Allen A.M., Lunos S., Tosun N. Progesterone and postpartum smoking relapse: A pilot double-blind placebo-controlled randomized trial. Nicotine & Tobacco Research. 2016;18(11):2145–2153.
14. Aranda Regules JM, Mateos Vilchez P, Gonzalez Villalba A, Sanchez F, de Dios Luna del Castillo J. Validity of smoking measurements during pregnancy: specificity, sensitivity and cut-off points. Revista Espanola de Salud Publica. 2008; 82(5):535–45.
15. Aveyard P, Lawrence T, Cheng KK, Griffin C, Croghan E, Johnson C. A randomized controlled trial of smoking cessation for pregnant women to test the effect of a transtheoretical model-based intervention on movement in stage and interaction with baseline stage. British Journal of Health Psychology. 2006; 11:263–78.
16. Aveyard P, Lawrence T, Croghan E, Evans O, Cheng KK. Is advice to stop smoking from a midwife stressful for pregnant women who smoke? Data from a randomized controlled trial. Preventive Medicine. 2004; 40:575–82.
17. Aveyard P, Lawrence T, Evans O, Cheng KK. The influence of in-pregnancy smoking cessation programmes on partner quitting and women’s social support mobilization: a randomized controlled trial. BMC Public Health. 2005; 5:80.
18. Aveyard P, West R. Managing smoking cessation. BMJ. 2007; 335:37–41
19. Avidano Britton GR, Brinthaupt J, Stehle JM, et al. The effectiveness of a nurse-managed perinatal smoking cessation program implemented in a rural County. Nicotine Tob Res 2006;8:13–28.
20. Bailey, JW.; Loeb, BK.; Waage, G. A randomized trial of smoking intervention during pregnancy. Proceedings of the American Public Health Association 111th Annual Meeting; Dallas, Texas, USA. 1983 Nov 15; 1983. p. 58
21. Balmumcu A, Ünsal Atan Ş. Smoking cessation programs for pregnant women: utilizing whatsapp text messaging. J Addict Nurs 2021;32:188–96.
22. Baric L, MacArthur C, Sherwood M. A study of health education aspects of smoking in pregnancy. International Journal of Health Education 197619(2 Suppl):1–17
23. Baric L, MacArthur C. Health norms in pregnancy. British Journal of Preventive and Social Medicine. 1977; 31:30–8.
24. Bauman KE, Koch GG, Dent CW, Bryan ES. The influence of observing carbon monoxide level on cigarette smoking by public prenatal patients. American Journal of Public Health. 1983; 73:1089–91.
25. Belizan JM, Villar J, Victora C, Farnot U, Langer A, Barros F. Impact of health education during pregnancy on behavior and utilization of health resources. American Journal of Obstetrics and Gynecology 1995173:894–9
26. Berg CJ, Park ER, Chang Y, Rigotti NA. Is concern about post-cessation weight gain a barrier to smoking cessation among pregnant women? Nicotine & Tobacco Research. 2008; 10(7):1159–63.
27. Bernstein IM, Mongeon JA, Badger GJ, Solomon L, Heil SH, Higgins ST. Maternal smoking and its association with birthweight. Obstetrics & Gynecology. 2005; 106(5 Pt 1):986–91. [PubMed: 16260516] Heil, SH.; Higgins, ST. Characterizing nicotine withdrawal and craving in pregnant cigarette smokers. 66th Annual Scientific Meeting of the College on Problems of Drug Dependence; San Juan, Puerto Rico. 2004 June 12-17; 2004. *
28. Blake SM, Murray KD, ElKhorazaty MN, Gantz MG, Kiely M, Best D, et al. Environmental tobacco smoke avoidance among pregnant African-American nonsmokers. American Journal of Preventive Medicine. 2009; 36(3):225–34.
29. Blake, S.; El-Mohandes, A.; Schwartz, D.; ElKhorazaty, N.; Gantz, M.; Joseph, J., et al. Promoting smoking cessation during pregnancy and preventing postpartum relapse [abstract]. Pediatric Academic Societies Annual Meeting; Washington DC, USA. 2005 May 14-17; 2005. Abstract no: 3074
30. Blake, S.; Joseph, J.; Schwartz, D.; El-Khorazaty, N.; Gantz, M.; El-Mohandes, A., et al. Preventing prenatal and postpartum environmental tobacco smoke (ETS) exposure [abstract]. Pediatric Academic Societies Annual Meeting; Washington DC, USA. 2005 May 14-17; Abstract no: 2353
31. Blalock JA, Fouladi RT, Wetter DW, Cinciripini PM. Depression in pregnant women seeking smoking cessation treatment. Addictive Behaviours. 2005; 30(6):1195–208.
32. Bonollo DP, Zapka JG, Stoddard AM, Ma Y, Pbert L, Ockene JK. Treating nicotine dependence during pregnancy and postpartum: understanding clinician knowledge and performance. Patient Education and Counselling. 2002; 48:265–74.
33. Bradizza, C. M., Stasiewicz, P. R., Zhuo, Y., Ruszczyk, M., Maisto, S. A., Lucke, J. F., Giarratano, P. (2017). Smoking cessation for pregnant smokers: Development and and pilot test of an emotion regulation treatment supplement to standard smoking cessation for negative affect smokers. Nicotine & Tobacco Research, 19(5), 578–584
34. Brandon, T. H., Simmons, V. N., Meade, C. D., et al. (2012). Self-help booklets for preventing postpartum smoking relapse: A randomized trial. American Journal of Public Health, 102(11), 2109–2115.
35. Bullock L, Everett KD, Mullen PD, Geden E, Longo DR, Madsen R. Baby BEEP: A randomized controlled trial of nurses’ individualized social support for poor rural pregnant smokers. Maternal and Child Health Journal 200913(3): 395–406
36. Bullock LF, Everett KD, Mullen PD. Baby beep: a randomized clinical trial of smoking cessation for low income rural pregnant women using nurse-delivered social support. Annals of Behavioral Medicine 200835:S99
37. Bullock LF, Hornblow AR, Duff GB, Wells JE. Telephone support for pregnant women: outcome in late pregnancy. New Zealand Medical Journal. 1995; 108:476–8.
38. Bullock, L., Everett, K. D., Mullen, P. D., Geden, E., Longo, D. R., & Madsen, R. (2009). Baby BEEP: A randomized controlled trial of nurses’ individualized social support for poor rural pregnant smokers. Maternal and Child Health Journal, 13(3), 395–406
39. Burling TA, Bigelow GE, Robinson JC, Mead AM. Smoking during pregnancy: reduction via objective assessment and directive advice. Behavior Therapy 1991:22:31–40
40. Byrd JC, Meade CD. Smoking cessation among pregnant women in an urban setting. Wisconsin Medical Journal 199392:609–12
41. Calderón SH, Gilbert P, Jackson R, Kohn MA, Gerbert B. Cueing prenatal providers: effects on discussions of intimate partner violence. American Journal of Preventive Medicine. 2008; 34(2):134–7.
42. Campbell, E., Walsh, R. A., Sanson-Fisher, R., Burrows, S., & Stojanovski, E. (2006). A group randomised trial of two methods for disseminating a smoking cessation pro-gramme to public antenatal clinics: Eﬀects on patient outcomes. Tobacco Control, 15(2), 97–102.
43. Cinciripini 2000  Blalock JA, Fouladi RT, Wetter DW, Cinciripini PM. Depression in pregnant women seeking smoking cessation treatment. Addictive Behaviours 200530(6):1195–208
44. Cinciripini PM, Blalock JA, Minnix JA, Robinson JD, Brown VL, Lam C, et al. Effects of an intensive depression focused intervention for smoking cessation in pregnancy. Journal of Consulting & Clinical Psychology 201078(1): 44–54
45. Cinciripini PM, McClure JB, Wetter DW, Perry J, Blalock JA, Cinciripini LG, et al. An evaluation of videotaped vignettes for smoking cessation and relapse prevention during pregnancy: The Very Important Pregnant Smokers (VIPS) Program. Tobacco Control. 2000; 9(3):iii61–iii63.
46. Coleman T, Clark M, Welch C, et al. Effectiveness of offering tailored text message, self-help smoking cessation support to pregnant women who want information on stopping smoking: miquit3 randomised controlled trial and meta-analysis. Addiction 2022;117:1079–94.
47. Cook C, Ward S, Myers S, Spinnato J. A prospective, randomized evaluation of intensified therapy for smoking reduction in pregnancy. American Journal of Obstetrics and Gynecology. 1995; 172:290.
48. Cooke M, Mattick R, Campbell E. The influence of individual and organisational factors on the reported smoking intervention practices of staff in 20 antenatal clinics. Drug and Alcohol Review. 1998; 17(2): 175–85.
49. Cooke M, Mattick RP, Campbell E. A description of the adoption of the ‘Fresh Start’ smoking cessation program by antenatal clinic managers. Australian Journal of Advanced Nursing. 2000; 18(1):13–21
50. Cooke M, Mattick RP, Campbell E. The dissemination of a smoking cessation program to 23 antenatal clinics: the predictors of initial program adoption by managers. Australian and New Zealand Journal of Public Health. 1999; 23(1):99–103.
51. Cooper S, Foster K, Naughton F, et al. Pilot study to evaluate a tailored text message intervention for pregnant smokers (MiQuit): study protocol for a randomised controlled trial. Trials. 2015;16:29. 29
52. Cope G, Nayyar P, Holder R, Gibbons J, Brunce R. A simple near patient test for nicotine and its metabolites in urine to assess smoking habit. Clinical Chimica Acta. 1996; 256:135–49.Cope GF. Smoking status and pregnancy: point of care cotinine test. BMJ. 2009; 339:b5652
53. Cope GF, Nayyar P, Holder R. Feedback from a point of-care test for nicotine intake to reduce smoking during pregnancy. Annals of Clinical Biochemistry 2003.40(Pt 6): 674–9
54. Cope GF, Nayyar P, Holder R. Measurement of nicotine intake in pregnant women - associations to changes in blood cell count. Nicotine & Tobacco Research. 2001; 3(2):119–22
55. Crittenden KS, Manfredi C, Cho YI, Dolecek TA. Smoking cessation processes in lowSES women: the impact of timevarying pregnancy status, healthcare messages, stress and health concerns. Addictive Behaviors. 2007; 32:1347–66.
56. Cummins S.E., Tedeschi G.J., Anderson C.M., Zhu S.H. Telephone intervention for pregnant smokers: A randomized controlled trial. American Journal of Preventive Medicine. 2016;51(3):318–326
57. Curry SJ, McBride C, Grothus L, Lando H, Pirie P. Motivation for smoking cessation among pregnant women. Psychology of Addictive Behaviors. 2001; 15(2):126–32.
58. de Vries H, Bakker M, Dolan Mullen P, van Breukelen G. The effects of smoking cessation counseling by midwives on Dutch pregnant women and their partners. Patient Educ Couns. 2006;63:177–87
59. Donatelle RJ, Hudson D, Dobie S, Goodall A, Hunsberger M, Oswald K. Incentives in smoking cessation: status of the field and implications for research and practice with pregnant smokers. Nicotine and Tobacco Research. 2004; 6(S2):S163–S179.
60. Donatelle RJ, Prows SL, Champeau D, Hudson D. Randomised controlled trial using social support and financial incentives for high risk pregnant smokers: Significant Other Supporter (SOS) program. Tobacco Control 2000.9 Suppl 3:iii67–iii69
61. Donatelle, RJ.; Hudson, D. Using 5 A’s and incentives to promote prenatal smoking cessation. National Conference of Tobacco or Health; San Francisco, California, USA. 2002 November 19-21; 2002.
62. Donovan J. Randomised controlled trial of anti-smoking advice in pregnancy. British Journal of Preventive and Social Medicine 1977.31(1):6–12
63. Donovan JW, Burgess PL, Hossack CM, Yudkin GD. Routine advice against smoking in pregnancy. Journal of the Royal College of General Practitioners. 1975; 25(153):264–8.
64. Donovan JW. Randomised controlled trial of anti-smoking advice in pregnancy. Journal of Epidemiology and Community Health. 1996; 50(3):232–6.
65. Dornelas EA, Magnavita J, Beazoglou T, Fischer EH, Oncken C, Lando H, et al. Efficacy and cost-effectiveness of a clinic-based counseling intervention tested in an ethnically diverse sample of pregnant smokers. Patient Education and Counseling 2006.64(1-3):342–9
66. Dunkley J. Training midwives to help pregnant women stop smoking  Nursing Times 1997.93(5):64–6
67. Eades SJ, Sanson-Fisher RW, Wenitong M, Panaretto K, D’Este C, Gilligan C, et al. An intensive smoking intervention for pregnant Aboriginal and Torres Strait Islander women: a randomised controlled trial. Medical Journal of Australia 2012.197(1):42–6
68. Edwards N.C., Sims-Jones N. A randomized controlled trial of alternative approaches to community follow-up for postpartum women. Canadian Journal of Public Health. 1997;88(2):123–128.
69. Edwards MJ, Geiser T, Chafin C, et al. S.m.a.r.t. mothers are resisting tobacco: prenatal smoking cessation in WIC mothers. J Allied Health 2009;38:170–6.
70. El-Khorazaty MN, Johnson AA, Kiely M, ElMohandes AA, Subramanian S, Laryea HA, et al. Recruitment and retention of low-income minority women in a behavioral intervention to reduce smoking, depression, and intimate partner violence during pregnancy. BMC Public Health. 2007; 7:233.
71. El-Mohandes AA, El-Khorazaty MN, Kiely M, Gantz MG. Smoking cessation and relapse among pregnant African-American smokers in Washington, DC. Maternal & Child Health Journal 2011.15 Suppl 1:S96–S105
72. El-Mohandes AA, Kiely M, Blake SM, Gantz MG, El-Khorazaty MN. An intervention to reduce environmental tobacco smoke exposure improves pregnancy outcomes. Pediatrics. 2010; 125(4):721–8.
73. El-Mohandes AA, Kiely M, Joseph JG, Subramanian S, Johnson AA, Blake SM, et al. An intervention to improve postpartum outcomes in African-American mothers: a randomized controlled trial. Obstetrics & Gynecology. 2008; 112(3):611–20.
74. El-Mohandes AEE, Kiely M, Gantz MG, El-Khorazaty N. Very preterm birth is reduced in women receiving an integrated behavioural intervention: A randomized controlled trial. Maternal Child Health Journal. 2011; 15:19–28.
75. El-Mohandes, AAE. A psycho-behavioral intervention on African American pregnant women with a history of intimate partner violence (IPV) improves birth weight distribution of their newborns [abstract]. Pediatric Academic Societies Annual Meeting; San Francisco, CA, USA. 2006 April 29-May 2
76. El-Mohandes, AAE. An integrated behavioral intervention reduces rates of moderate and extreme prematurity in African American (AA) mothers with a history of smoking during pregnancy [abstract]. Pediatric Academic Societies Annual Meeting; San Francisco, CA, USA. 2006 April 29-May 2
77. El-Mohandes, AAEE. An integrated psycho-behavioral intervention during pregnancy has significant effects in reducing risks during the post-partum period in African-American women. Pediatric Academic Societies Annual Meeting; Washington DC, USA. 2005 May 14-17; Abstract no: 39
78. El-Mohandes, AAE.; Kiely, M.; Gantz, MG.; ElKhorazaty, N. A multiple risk factor behavioral intervention reduces environmental tobacco smoke exposure. Pediatric Academic Societies Annual Meeting; Toronto, Canada. 2007 May 5-8
79. England LJ, Kendrick JS, Wilson HG, Merritt RK, Gargiullo PM, Zahniser SC. Effects of smoking reduction during pregnancy on the birth weight of term infants. American Journal of Epidemiology. 2001; 154:694–701.
80. Ershoff D.H., Quinn V.P., Mullen P.D. Relapse prevention among women who stop smoking early in pregnancy: A randomized clinical trial of a self-help intervention. American Journal of Preventive Medicine. 1995;11(3):178–184.
81. Ershoff DH, Lairson DR, Mullen PD, Quinn VP. Pregnancy and medical cost outcomes of a self-help prenatal smoking cessation program in an HMO. Public Health Reports. 1990; 105(4):340–7.
82. Ershoff DH, Quinn VP, Boyd NR, Stern J, Gregory M, Wirtschafter D. The Kaiser Permanente prenatal smoking cessation trial. When more isn’t better, what is enough? American Journal of Preventive Medicine 1999.17(3):161–8
83. Ershoff DH, Quinn VP, Boyd NR, Stern J, Gregory M, Wirtschafter D. The Kaiser Permanente prenatal smoking cessation trial: when more isn’t better, what is enough? Tobacco Control. 2000; 9(Suppl 3):iii60.
84. Ershoff DH, Quinn VP, Mullen PD. A randomized trial of a serialized self-help smoking cessation program for pregnant women in an HMO. American Journal of Public Health 1989.79(2):182–7
85. Ershoff DH, Quinn VP, Mullen PD. Relapse prevention among women who stop smoking early in pregnancy: a randomized clinical trial of a self-help intervention. American Journal of Preventive Medicine. 1995; 11(3):178–84.
86. Ershoff DH, Solomon LJ, Dolan-Mullen P. Predictors of intentions to stop smoking early in prenatal care. Tobacco Control. 2000; 9(3):41.
87. Ershoff, D. H., Quinn, V. P., Boyd, N. R., Stern, J., Gregory, M., & Wirtschafter, D. (1999). The Kaiser Permanente prenatal smoking-cessation trial: When more isn’t better, what is enough? American Journal of Preventive Medicine, 17(3), 161– 168. doi:10.1016/S0749-3797(99)00071-9
88. Everett-Murphy K, Steyn K, Mathews C, et al. The effectiveness of adapted, best practice guidelines for smoking cessation counseling with disadvantaged, pregnant smokers attending public sector antenatal clinics in Cape town, South Africa. Acta Obstet Gynecol Scand 2010;89:478–89.
89. Ewigman B, Crane J, Frigoletto F, LeFevre M, Bain R, McNellis D. Effect of prenatal ultrasound screening on perinatal outcome. New England Journal of Medicine. 1993; 329:821–9.
90. Ferreira-Borges C. Effectiveness of a brief counseling and behavioral intervention for smoking cessation in pregnant women. Prev Med 2005;41:295–302.
91. Forinash AB, Yancey A, Chamness D, Koerner J, Inteso C, Miller C, et al. Smoking cessation following text message intervention in pregnant women. Ann Pharmacother 2018 Nov;52(11):1109-1116. [doi: 10.1177/1060028018780448] [Medline: 29857773]
92. Forray A., Gilstad-Hayden K., Sofuoglu M., Yonkers K. Progesterone for postpartum smoking relapse prevention. Neuropsychopharmacology. 2016;41:S606.
93. Forray A., Waters A. Ecological momentary assessments and attentional bias modification for postpartum smoking. Neuropsychopharmacology. 2015;40:S590–S591
94. Fox NL, Sexton M, Hebel JR, Thompson B. The reliability of self-reports of smoking and alcohol consumption by pregnant women. Addictive Behaviors. 1989; 14(2):187–95. [
95. Fox NL, Sexton MJ, Hebel JR. Alcohol consumption among pregnant smokers: effects of a smoking cessation intervention program. American Journal of Public Health. 1987; 77:211–3.
96. Fujioka N, Kobayashi T, Turale S. Short-term behavioral changes in pregnant women after a quit-smoking program via e-learning: a descriptive study from Japan. Nurs Health Sci 2012 Sep;14(3):304-311. [doi: 10.1111/j.1442-2018.2012.00702.x] [Medline: 22950611]
97. Gadomski, A., Adams, L., Tallman, N., Krupa, N., & Jenkins, P. (2011). Effectiveness of a combined prenatal and postpartum smoking cessation program. Maternal and Child Health Journal, 15(2), 188-197. doi: 10.1007/s10995-010-0568-9
98. Gebauer C, Kwo CY, Haynes EF, et al. A nurse-managed smoking cessation intervention during pregnancy. J Obstet Gynecol Neonatal Nurs 1998;27:47–53.
99. Gielen AC, Windsor R, Faden RR, O’Campo P, Repke J, Davis M.  Evaluation of a smoking cessation intervention for pregnant women in an urban prenatal clinic. Health Education Research 1997.12(2):247–54
100. Gilligan C, Sanson-Fisher R, Eades S, Wenitong M, Panaretto K, D’Este C. Assessing the accuracy of self-reported smoking status and impact of passive smoke exposure among pregnant Aboriginal and Torres Strait Islander women using cotinine biochemical validation. Drug and Alcohol Review. 2010; 29:35–40.
101. Gilligan C, Sanson-Fisher RW, D-Este C, Eades S, Wenitong M. Knowledge and attitudes regarding smoking during pregnancy among Aboriginal and Torres Strait Islander women. Medical Journal of Australia. 2009; 190(10):557–61.
102. Gilligan, C. A pilot randomised controlled trial to test the effectiveness of an intervention to help Aboriginal and Torres Strait Islander women to quit smoking during pregnancy: study design and preliminary results [thesis]. University of Newcastle; Newcastle, Australia: 2008.
103. Glover M, Kira A, Walker N, Bauld L. Using incentives to encourage smoking abstinence among pregnant indigenous women? A feasibility study. Maternal and Child Health Journal 2014.19(6):1393–9
104. Graham AV, Reeb KG, Kitson GC, Zyzanski SJ, Frank SH. A clinical trial to reduce the rate of low birth weight in an inner-city black population. Family Medicine 1992.24: 439–46
105. Groff, J.; Stotts, A.; Velasquez, M.; Benjamin-Garner, R.; Green, C.; Mastrobattista, J. Ultrasound and motivational enhancement for prenatal smoking cessation. Annual Meeting of the Society for Behavioural Medicine; Boston, MA. 2005 April 13-16; 2005.
106. Haddow JE, Polomak JE, Sepulveda D. Smoking cessation during routine public prenatal care. American Journal of Public Health. 1995; 85(10):1451–2.
107. Haddow JE, Wald NJ, Palomaki GE, Kloza EM, Knight GJ. Cotinine-assisted intervention in pregnancy to reduce smoking and low birthweight delivery.  British Journal of Obstetrics and Gynaecology 1991.98(9):859–65
108. Hajek P, West R, Lee A, Foulds J, Owen L, Eiser JR, et al. Randomized controlled trial of a midwife-delivered brief smoking cessation intervention in pregnancy. Addiction 2001.96(3):485–94
109. Hajek, P., West, R., Lee, A., et al. (2001). Randomized controlled trial of a midwife-delivered brief smoking cessation intervention in pregnancy. Addiction, 96(3), 485–494.
110. Hamilton BH. Estimating treatment effects in randomized clinical trials with non-compliance: the impact of maternal smoking on birthweight. Health Economics. 2001; 10(5):399–410.
111. Hannover W., Thyrian J.R., Roske K. Smoking cessation and relapse prevention for postpartum women: Results from a randomized controlled trial at 6, 12, 18 and 24 months. Addictive Behaviors. 2009;34(1):1–8.
112. Harris M, Reynolds B. A pilot study of home-based smoking cessation programs for rural, Appalachian, pregnant smokers. Journal of Obstetric, Gynecologic, and Neonatal Nursing 2015 [EPub ahead of print]
113. Harris, M., & Reynolds, B. (2015). A pilot study of homebased smoking cessation programs for rural, Appalachian, pregnant smokers. Journal of Obstetric, Gynecologic, & Neonatal Nursing, 44(2), 236-245
114. Hartmann K, Thorp J, Pahel-Short L, Koch M. A randomized controlled trial of smoking cessation intervention in pregnancy. American Journal of Obstetrics and Gynecology. 1995; 172:287
115. Hartmann KE, Koch MA, Pahel-Short L, Thorp JM. A randomized controlled trial of smoking cessation intervention in pregnancy in an academic clinic. Obstetrics & Gynecology 1996.87:621–6
116. Haug K, Fugelli P, Aaro LE, Foss OP. Is smoking intervention in general practice more successful among pregnant than non-pregnant women? Family Practice 1994 11:111–6
117. Haug K, Fugelli P, Aaro LE. Recruitment and participation of General Practitioners in a multipractice study of smoking cessation. Scandinavian Journal of Primary Health Care. 1992; 10(3):206–10.
118. Haug NA, DiClemente C, Svikis DS. Motivational enhancement therapy for nicotine dependence in methadone-maintained pregnant women. Psychology of Addictive Behaviours 2004.18(3):289–92
119. Hayes CB, Collins C, O’Carroll H, et al. Effectiveness of motivational interviewing in influencing smoking cessation in pregnant and postpartum disadvantaged women. Nicotine Tob Res 2013;15:969–77.
120. Hebel JR, Sexton M, Nowicki P. The effect of antismoking intervention during pregnancy: an assessment of interactions with maternal characteristics. American Journal of Epidemiology. 1985; 122:135–48.
121. Hegaard H, Hjaergaard H, Moller L, Wachmann H, Ottesen B. Multimodel intervention raises smoking cessation rate during pregnancy. Acta Obstetricia et Gynecologica Scandinavica 2003.82:813–9
122. Hegaard HK, Kjaergaard H, Moller LF, Wachmann H, Ottesen B. Determination of a saliva cotinine cut-off to distinguish pregnant smokers from pregnanct non-smokers. Acta Obstetricia et Gynecologica. 2007; 86:401–6.
123. Hegaard HK, Kjaergaard H, Moller LF, Wachmann H, Ottesen B. Long-term nicotine replacement therapy. British Journal of Midwifery. 2004; 12(4):214–20.
124. Heil SH, Higgins ST, Bernstein IM, Solomon LJ, Rogers RE, Thomas CS, et al. Effects of voucher-based incentives on abstinence from cigarette smoking and fetal growth among pregnant women. Addiction. 2008.103(6):1009–18
125. Heil SH, Tidey JW, Holmes HW, Badger GJ, Higgins ST. A contingent payment model of smoking cessation: effects on abstinence and withdrawal. Nicotine and Tobacco Research. 2003; 5(2):205–13.
126. Heil SH.; Higgins, ST.; Solomon, LJ.; Lynch, ME.; McHale, L.; Dumeer, A., et al. Voucher-based incentives for abstinence from cigarette smoking in pregnant and postpartum women. Society for Research on Nicotine and Tobacco 13th Annual Meeting; Austin, Texas. 2007 Feb 21-24; 2007. p. 25Abstract no: PA6-1
127. Hennrikus D, Pirie P, Hellerstedt W, Lando HA, Steele J, Dunn C. Increasing support for smoking cessation during pregnancy and postpartum: results of a randomized controlled pilot study. Preventive Medicine 2010.50(3): 134–7
128. Herbec A, Brown J, Tombor I, Michie S, West R. Pilot randomized controlled trial of an internet-based smoking cessation intervention for pregnant smokers (’MumsQuit’). Drug & Alcohol Dependence 2014.140:130–6
129. Hiett A, Brazus S, Hedberg J, Brown H. Smoking cessation program effectiveness during pregnancy. American Journal of Obstetrics and Gynecology 2000.182(1 Pt 2):S150
130. Higgins ST, Bernstein IM, Washio Y, Heil SH, Badger GJ, Skelly JM, et al. Effects of smoking cessation with voucher-based contingency management on birth outcomes. Addiction. 2010; 105:2023–30.
131. Higgins ST, Heil SH, Badger GJ, Mongeon JA, Solomon LJ, McHale L, et al. Biochemical verification of smoking status in pregnant and recently postpartum women. Experimental and Clinical Psychopharmacology. 2007; 15(1):58–66.
132. Higgins ST, Heil SH, Dumeer AM, Thomas CS, Solomon LJ, Bernstein IM. Smoking status in the initial weeks of quitting as a predictor of smoking-cessation outcomes in pregnant women. Drug and Alcohol Dependence. 2006; 85:138–41.
133. Higgins ST, Heil SH, Solomon LJ, Bernstein IM, Lussier JP, Abel RL, et al. A pilot study on voucher-based incentives to promote abstinence from cigarette smoking during pregnancy and postpartum. Nicotine and Tobacco Research. 2004; 6(6):1015–20. [
134. Higgins TM, Higgins ST, Heil SH, Badger GJ, Skelly JM, Bernstein IM, et al. Effects of cigarette smoking cessation on breastfeeding duration. Nicotine & Tobacco Research. 2010; 12(5):483–8.
135. Higgins, S. T., Washio, Y., Lopez, A. A., Heil, S. H., Solomon, L. J., Lynch, M. E., . Bernstein, I. M. (2014). Examining two different schedules of financial incentives for smoking cessation among pregnant women. Preventive Medicine, 68, 51–57.
136. Hjalmarson AIM, Svanberg B, Hahn L. Stopping smoking in pregnancy: effect of a self-help manual in a controlled trial. British Journal of Obstetrics and Gynaecology 1991.98: 260–4
137. Hughes E, Lamont D, Beecroft M, Wilson D. Randomized trial of a “stage-of-change” orientated smoking cessation intervention in infertile and pregnant women. Fertility and Sterility 2000.74(3):498–503
138. Hughes EG, Beecroft ML, Lamont D, Rice S, Wilson D, Freebury M, et al. A randomised controlled trial of a “State-of Change” smoking cessation intervention for subfertile and pregnant patients. Fertility and Sterility. 1999; 72(3 Suppl 1):S61–S62.
139. Jimenez-Muro A., Nerin I., Samper P. A proactive smoking cessation intervention in postpartum women. Midwifery. 2013;29(3):240–245
140. Johnson J.L., Ratner P.A., Bottorff J.L., Hall W., Dahinten S. Preventing smoking relapse in postpartum women. Nursing Research. 2000;49(1):44–52.
141. Joseph JG, El-Mohandes AA, Kiely M, El-Khorazaty MN, Gantz MG, Johnson AA, et al. Reducing psychosocial and behavioral pregnancy risk factors: results of a randomized clinical trial among high-risk pregnant African American women. American Journal of Public Health. 2009; 99(6):1053–61.
142. Joseph, J.; for NIH-DC initiative to reduce infant mortality. Randomized trial to reduce 4 behaviors linked to adverse pregnancy outcomes among 1048 inner-city African American women [abstract]. Pediatric Academic Societies Annual Meeting; Washington DC, USA. 2005 May 14-17; Abstract no: 1701
143. Joyce CM, Saulsgiver K, Mohanty S, et al. Remote patient monitoring and incentives to support smoking cessation among pregnant and postpartum Medicaid members: three randomized controlled pilot studies. JMIR Form Res 2021;5:e27801.
144. Karatay G, Kublay G, Emiroğlu ON (2010) Effect of motivational interviewing on smoking cessation in pregnant women. J Adv Nurs 66:1328–1337
145. Katz KS, Blake SM, Milligan RA, Sharps PW, White DB, Rodan MF, et al. The design, implementation and acceptability of an integrated intervention to address multiple behavioural and psychosocial risk factors among pregnant African American women. BMC Pregnancy and Childbirth. 2008; 8(22)
146. Kemp L, Harris E, McMahon C, Matthey S, Vimpani G, Anderson T, Schmied V, Aslam H, Zapart S. Child and family outcomes of a long-term nurse home visitation programme: a randomised controlled trial. Archives of Disease in Childhood 2011.96(6):533–540
147. Kendrick JS, Metzger RW, Sexton M, Spierto FW, Floyd RL, Gargiullo PM, et al. Integrating smoking cessation into routine public prenatal care: the Smoking Cessation in Pregnancy project. American Journal of Public Health 1995 85:217–22
148. Kiely, M.; ElKhorazaty, MN.; El-Mohandes, AAE. Depression and smoking during pregnancy impact the efficacy of an integral behavioral intervention to resolve risks. Pediatric Academic Societies Annual Meeting; Toronto, Canada. 2007 May 5-8
149. Kientz E., Kupperschmidt B. KICCS: A successful strategy to promote smoking cessation in women during and post pregnancy. The Oklahoma Nurse. 2005;50(4):27–30.
150. Kurti AN, Tang K, Bolivar HA, et al. Smartphone-Based financial incentives to promote smoking cessation during pregnancy: a pilot study. Prev Med 2020;140:S0091-7435(20)30225-5:106201.
151. Lancaster T, Dobbie W, Vos K, et al. Randomized trial of nurseassisted strategies for smoking cessation in primary care. Br J Gen Pract 1999;49:191–4.
152. Lando HA, Valanis BG, Lichtenstein E, Curry SJ, McBride CM, Pirie PL, et al. Promoting smoking abstinence in pregnant and postpartum patients: a comparison of 2 approaches. American Journal of Managed Care. 2001; 7:685–93.
153. Lawrence T, Aveyard P, Cheng KK, Griffin C, Johnson C, Croghan E. Does stage-based smoking cessation advice in pregnancy result in long term quitters? 18-month postpartum follow-up of a randomized controlled trial. Addiction. 2005; 100:107–16.
154. Lawrence T, Aveyard P, Croghan E. What happens to women’s self-reported cigarette consumption and urinary cotinine levels in pregnancy? Addiction. 2003; 98:1315–20.
155. Lawrence T, Aveyard P, Evans O, Cheng KK. A cluster randomised controlled trial of smoking cessation in pregnant women comparing interventions based on the transtheoretical (stages of change) model to standard care. Tobacco Control. 2003; 12:168–77.
156. Lawrence, T., Aveyard, P., Evans, O., & Cheng, K. (2003). A cluster randomised controlled trial of smoking cessation in pregnant women comparing interventions based on the transtheoretical (stages of change) model to standard care. Tobacco Control, 12(2), 168–177. doi:10.1136/tc.12.2.168
157. Lee M, Miller SM, Wen KY, Hui SA, Roussi P, Hernandez E. Cognitive-behavioral intervention to promote smoking cessation for pregnant and postpartum inner city women. Journal of Behavioral Medicine 2015.38(6):923–43
158. LeFevre ML, Ewigman B, Evans JK. Is smoking an indication for prenatal ultrasonography? RADIUS Study Group. Archives of Family Medicine 1995.4:120–3
159. Levine M.D., Cheng Y., Marcus M.D., Kalarchian M.A., Emery R.L. Preventing postpartum smoking relapse: A randomized clinical trial. JAMA Internal Medicine. 2016;176(4):443–452
160. Lilley J, Forster DP. A randomised controlled trial of individual counselling of smokers in pregnancy. Public Health 1986.100:309–15
161. Lillington L, Chlebowski R, Ruvalcaba M, Novak D, Royce J. Evaluation of a smoking cessation program for pregnant minority smokers. Cancer Practice 1995.3(3):157–63
162. Linares Scott TJ, Heil SH, Higgins ST, Badger GJ, Bernstein IM. Depressive symptoms predict smoking status among pregnant women. Addictive Behaviours. 2009; 34:705–8.
163. Loeb, BK.; Waage, G.; Bailey, J. Smoking intervention in pregnancy. Proceedings of the Fifth World Conference on Smoking and Health; Winnipeg, Canada. Ottawa. 1983 July; Canadian Council on Smoking and Health; 1983. p. 389-95.
164. Loukopoulou A, Vardavas C, Tzatzarakis M, Farmakides G, Rossolymos C, Chrelias C, et al. Urinary nicotine and cotinine as a method for assessing the effectiveness of an intervention to aid smoking cessation during pregnancy. Chest 2012.142(4 Suppl 1):1081A
165. Loukopoulou AN, Vardavas CI, Farmakides G, et al. Counselling for smoking cessation during pregnancy reduces tobacco-specific nitrosamine (NNAL) concentrations: a randomized controlled trial. Eur J Midwifery 2018;2:14.
166. Lowe JB, Balanda KP, Stanton WR, Del Mar C, O’Connor V. Dissemination of an efficacious antenatal smoking cessation program in public hospitals in Australia: a randomised controlled trial. Health Education & Behavior 2002.29(5):608–19
167. Lowe JB, Windsor R, Balanda K, Woodby L. Smoking relapse prevention methods for pregnant women: a formative evaluation. American Journal of Health Promotion 1997.11:244–6
168. Lussier JP, Heil SH, Mongeon JA, Badger GJ, Higgins ST. A meta-analysis of voucher-based reinforcement therapy for substance use disorders. Addiction. 2006; 101:192–203.
169. Ma Y, Goins KV, Pbert L, Ockene JK. Predictors of smoking cessation in pregnancy and maintenance postpartum in low-income women. Maternal and Child Health Journal. 2005; 9(4):393–402.
170. Malchodi CS, Oncken C, Dornelas EA, Caramanica L, Gregonis E. The effects of peer counselling on smoking cessation and reduction. Obstetrics & Gynecology 2003.101 (3):504–10
171. Manfredi C, Cho YI, Warnecke R, Saunders S, Sullivan M. Dissemination strategies to improve implementation of the PHS smoking cessation guideline in MCH public health clinics: experimental evaluation results and contextual factors. Health Education Research. 2011; 26(2):348–60.
172. Manfredi C, Crittenden KS, Warnecke R, Engler J, Cho YI, Shaligram C. Evaluation of a motivational smoking cessation intervention for women in public health clinics. Preventive Medicine 1999.28:51–60
173. Marin-Gomez FX, Garcia-Moreno Marchán R, Mayos-Fernandez A, et al. Exploring efficacy of a serious game (tobbstop) for smoking cessation during pregnancy: randomized controlled trial. JMIR Serious Games 2019;7:e12835
174. Mauriello L, Dyment S, Prochaska J, Gagliardi A, Weingrad-Smith J. Acceptability and feasibility of a multiple-behaviour, computer-tailored intervention for underserved pregnant women. Journal of Midwifery and Women’s Health 2011.56:75–80
175. Mayer JP, Todd R, Hawkins B. A randomised evaluation of smoking cessation interventions for pregnant women at a WIC clinic. American Journal of Public Health 1990.80: 76–7
176. McBride C.M., Curry S.J., Lando H.A., Pirie P.L., Grothaus L.C., Nelson J.C. Prevention of relapse in women who quit smoking during pregnancy. American Journal of Public Health. 1999;89(5):706–711.
177. McBride CM, Baucom DH, Peterson BL, Pollak KI, Palmer C, Westman E, et al. Prenatal and postpartum smoking abstinence: a partner assisted approach. American Journal of Preventive Medicine 2004.27(3):232–8
178. McBride CM, Curry SJ, Grothaus LC, Nelson JC. Partner smoking status and pregnant smokers’ perception of support for and likelihood of smoking cessation. Health Psychology. 1998; 17:63–9.
179. McLeod D, Benn C, Pullon S, Viccars A, White S, Cookson T, et al. The midwife’s role in facilitating smoking behaviour change during pregnancy. Midwifery. 2003; 19(4):285–97.
180. McLeod D, Pullon S, Benn C, Cookson T, Dowell A, Viccars A, et al. Can support and education for smoking cessation and reduction be provided effectively by midwives within primary maternity care? Midwifery 2004.20:37–50
181. Mejdoubi J, van den Heijkant SCCM, van Leerdam FJM, Crone M, Crijnen A, HiraSing RA. Effects of nurse home visitation on cigarette smoking pregnancy outcomes: a randomized controlled trial. Midwifery 2014.30:688–95
182. Messimer SR, Henry RC, Hickner JM. A comparison of two antismoking interventions among pregnant women in eleven primary care practices. Journal of Family Practice 1989.28(3):283–8
183. Moore LO, Campbell R, Whelan A, Mills N, Lupton P, Misselbrook E, et al. Self help smoking cessation in pregnancy: cluster randomised controlled trial. BMJ 2002 325:1383–6
184. Moore ML, Meis PJ, Ernest JM, Wells HB, Zaccaro DJ, Terrell T. A randomized trial of nurse intervention to reduce preterm and low birth weight births. Obstetrics & Gynecology 1998.91:656–61
185. Moore ML, Zaccaro DJ. Cigarette smoking, low birth weight, and preterm births in low-income AfricanAmerican women. Journal of Perinatology. 2000; 3:176–80.
186. Morasco BJ, Dornelas EA, Fischer EH, Oncken C, Lando HA. Spontaneous smoking cessation during pregnancy among ethnic minority women: a preliminary investigation. Addictive Behaviors. 2006; 31(2):203–10.
187. Moss, D. R., Cluss, P. A., Watt-Morse, M., & Pike, F. (2009). Targeting pregnant and parental smokers: Long-term outcomes of a practice-based intervention. Nicotine & Tobacco Research, 11(3), 278–285
188. Mullen P.D., DiClemente C.C., Bartholomew L.K. Theory and context in project PANDA: a program to help postpartum women stay off cigarettes. In: Bartholomew L.K., Parcel G.S., Kok G., Gottlieb N.H., editors. Intervention Mapping: A Process for Designing Theory- and Evidence-based Health Promotion Programs. McGraw-Hill Publishing Co.; Palo Alto, CA: Mayfield: 2001
189. Mullen PD, Carbonari JP, Tabak ER, Glenday MC. Improving disclosure of smoking by pregnant women. American Journal of Obstetrics and Gynecology. 1991; 165:409–13.
190. Mullen, P. D., Quinn, V. P., & Ershoff, D. H. (1990). Maintenance of nonsmoking postpartum by women who stopped smoking during pregnancy. American Journal of Public Health, 80(8), 992–994.
191. Naughton F, Cooper S, Foster K, Emery J, Leonardi-Bee J, Sutton S, et al. Large multi-centre pilot randomized controlled trial testing a low-cost, tailored, self-help smoking cessation text message intervention for pregnant smokers (MiQuit). Addiction 2017 Jul;112(7):1238-1249 [FREE Full text] [doi: 10.1111/add.13802] [Medline: 28239919]
192. Naughton F, Jamison J, Sutton S. Attitudes towards SMS text message smoking cessation support: a qualitative study of pregnant smokers. Health Educ Res. 2013;28(5):911–922. doi: 10.1093/her/cyt057
193. Naughton F, Prevost AT, Gilbert H, Sutton S. Randomized controlled trial evaluation of a tailored leaflet and SMS text message self-help intervention for pregnant smokers (MiQuit). Nicotine and Tobacco Research. 2012; 14(5):569–77.
194. Naughton F. Delivering “ just-in-time” smoking cessation support via mobile phones: current knowledge and future directions. Nicotine Tob Res 2017;19:379–83.
195. Nowicki P, Gintzig L, Hebel JR, Lathem R, Miller V, Sexton M. Effective smoking intervention during pregnancy. Birth. 1984; 11:217–24.
196. O’Connor, A. M., Davies, B. L., Dulberg, C. S., et al. (1992). Effectiveness of a pregnancy smoking cessation program. Journal of Obstetric, Gynecologic, and Neonatal Nursing, 21(5), 385–392.
197. Øien T, Storrø O, Jenssen JA, Johnsen R. The impact of a minimal smoking cessation intervention for pregnant women and their partners on perinatal smoking behavior in primary health care: a real-life controlled study. BMC Pub Health. 2008;8:325
198. Olds D, Robinson J, O’Brien R, Luckey D, Pettit L, Henderson C, et al. Home visiting by paraprofessionals and by nurses: a randomized, controlled trial. Pediatrics 2002 110(3):486–96
199. Olds DL, Henderson CR, Chamberlin R, Tatelbaum R. Preventing child abuse and neglect: a randomized trial of nurse home visitation. Pediatrics 1986.78:65–78
200. Olds DL, Henderson CR, Tatelbaum R, Chamberlin R. Improving the delivery of prenatal care and outcomes of pregnancy: a randomized trial of nurse home visitation. Pediatrics. 1986; 77:16–28.
201. Olds DL, Henderson CR, Tatelbaum R. Prevention of intellectual impairment in children of women who smoke cigarettes during pregnancy. Pediatrics. 1994; 93:228–33.
202. Ondersma SJ, Chase SK, Svikis DS, Schuster CR. Computer-based brief motivational intervention for perinatal drug use. Journal of Substance Abuse Treatment. 2005; 28:305–12.
203. Ondersma SJ, Svikis DS, Lam PK, Connors-Burge VS, Ledgerwood DM, Hopper JA. A randomized trial of computer-delivered brief intervention and low-intensity contingency management for smoking during pregnancy. Nicotine & Tobacco Research 2012 Vol 14, issue 3: 351–60
204. Ondersma, S.; Svikis, DS.; Beatty, JR.; Lockhart, N. A randomized clinical trial of a computer delivered brief intervention for post-partum drug, alcohol, and tobacco use: three-month outcomes. Proceedings of the 73rd Annual Scientific Meeting of the College on Problems of Drug Dependence; Hollywood, Florida. 2011 June 18-23; 2011.
205. Oude Wesselink SF, Lingsma HF, Reulings PG, Wentzel HR, Erasmus V, Robben PB, et al. Does government supervision improve stop-smoking counseling in midwifery practices? Nicotine & Tobacco Research 2015 Vol 17, issue 5:572–9
206. Panaretto KS, Mitchell MR, Anderson L, Gilligan C, Buettner P, Larkins SL, et al. Tobacco use and measuring nicotine dependence among urban Indigenous pregnant women. Medical Journal of Australia. 2009; 191(10):554–7.
207. Panjari M, Bell R, Astbury J, Bishop S, Dalais F, Rice G. Women who spontaneously quit smoking in early pregnancy. Australian New Zealand Journal of Obstetrics and Gynaecology. 1997; 37(3):271.
208. Panjari M, Bell R, Bishop S, Astbury J, Rice G, Doery J. A randomized controlled trial of a smoking cessation intervention during pregnancy. Australian and New Zealand Journal of Obstetrics and Gynaecology. 1999.39(3):312–7
209. Park ER, Quinn VP, Chang Y, Regan S, Loudin B, Cummins S, et al. Recruiting pregnant smokers into a clinical trial: using a network-model managed care organization versus community-based practices. Preventive Medicine. 2007; 44:223–9.
210. Parker DR, Windsor RA, Roberts MB, Hecht J, Hardy NV, Strolla LO, et al. Feasibility, cost, and costeffectiveness of a telephone-based motivational intervention for underserved pregnant smokers. Nicotine and Tobacco Research 2007.9(10):1043–51
211. Parker, DR.; Roberts, MB.; Windsor, RA.; Lasater, TM. Telephone-based smoking cessation interventions effective in high-risk, underserved pregnant women. Joint Conference of SRNT and SRNT-Europe; Dublin, Ireland. 2009 April 27-30; 2099.
212. Patten CA, Koller KR, Flanagan CA, Hiratsuka VY, Hughes CA, Wolfe AW et al. (2019) Biomarker feedback intervention for smoking cessation among Alaska Native pregnant women: Randomized pilot study. Patient Educ Couns, 102:528-535.
213. Patten CA, Windsor RA, Renner CC, Enoch C, Hochreiter A, Nevak C, et al. Feasibility of a tobacco cessation intervention for pregnant Alaska Native women. Nicotine & Tobacco Research 2009.12(2):79–87
214. Patten CA. Tobacco cessation intervention during pregnancy among Alaska Native women. Journal of Cancer Education. 2012; 27(Supp 1):S86–90.
215. Pbert L, Ockene JK, Zapka J, Ma Y, Goins KV, Oncken C, et al. A community health center smoking cessation intervention for pregnant and postpartum women. American Journal of Preventive Medicine 2004.26(5): 377–85
216. Peden, A. R., Rayens, M. K., Hall, L. A., et al. (2008). Nicotine addiction in pregnancy: Preliminary efficacy of a mental health intervention. Addictive Disorders and Their Treatment, 7(4), 179–189.
217. Petersen L., Handel J., Kotch J., Podedworny T., Rosen A. Smoking reduction during pregnancy by a program of self-help and clinical support. Obstetrics and Gynecology. 1992;79:924–930
218. Pletsch PK (2002) Reduction of primary and secondary smoke exposure for low-income black pregnant women. Nurs Clin, 37:315-329.
219. Polan´ska, K., Hanke, W., & Sobala, W. (2005). Smoking relapse one year after delivery among women who quit smoking during pregnancy. International Journal of Occupational Medicine and Environmental Health, 18(2), 159–165.
220. Polanska K, Hanke W, Sobala W, Lowe JB. Efficacy and effectiveness of the smoking cessation program for pregnant women. International Journal of Occupational Medicine and Environmental Health 2004.17(3):369–77
221. Polanska K, Hanke W, Sobala. Characteristic of the smoking habit among pregnant women on the base of the test “Why am I a smoker?” [Charakterystyka nagogu palenia papierosow wsrod kobiet ciezarnych na podstawie testu Dlaczego pale?]. Przeglad Lekarski. 2005; 62(10):1095–8.
222. Pollack KI, Lyna P, Bilheimer A, et al. A pilot study testing SMS text delivered scheduled gradual reduction to pregnant smokers. Nicotine Tob Res. 2013;15(10):1773–1776
223. Pollak K.I., Fish L.J., Lyna P. Efficacy of a nurse-delivered intervention to prevent and delay postpartum return to smoking: The quit for two trial. Nicotine & Tobacco Research. 2016;18(10):1960–1966
224. Pollak KI, Lyna P, Bilheimer A, Farrell D, Gao X, Swamy GK, et al. A pilot study testing SMS text delivered scheduled gradual reduction to pregnant smokers. Nicotine & Tobacco Research 2013.15(10):1773–6
225. Pollak KI, Lyna P, Gao X, et al. Efficacy of a Texting program to promote cessation among pregnant smokers: a randomized control trial. Ann Behav Med 2020;22:1187–94.
226. Pollak, K. I., Oncken, C. A., Lipkus, I. M., et al. (2007). Nicotine replacement and behavioral therapy for smoking cessation in pregnancy. American Journal of Preventive Medicine, 33(4), 297–305.
227. Prapavessis H, De Jesus S, Harper T, Cramp A, Fitzgeorge L, Mottola MF, et al. The effects of acute exercise on tobacco cravings and withdrawal symptoms in temporary abstinent pregnant smokers. Addictive Behaviors 2014.39(3):703–8
228. Price JH, Snyder FF, Roberts SM, Losh DP, Desmond SM, Krol RA. Comparison of three antismoking interventions among pregnant women in an urban setting: a randomized trial. Psychological Reports 1991.68:595–604
229. Pullon S, McCleod D, Benn C, Viccars A, White S, Cookson T, et al. Smoking cessation in New Zealand: education and resources for use by midwives for women who smoke during pregnancy. Health Promotion International. 2003; 18(4):315–24.
230. Reading AE, Cox DN. The effects of ultrasound on maternal anxiety. Journal of Behavioral Medicine 1982.5(2): 237–47
231. Reading AE, Sledmere CM, Cox DNB, Campbell S. Health beliefs and health care behaviour in pregnancy. Psychological Medicine. 1982; 12:379–83.
232. Reitzel, L. R., Vidrine, J. I., Businelle, M. S., et al. (2010). Preventing postpartum smoking relapse among diverse lowincome women: A randomized clinical trial. Nicotine & Tobacco Research, 12(4), 326–335.
233. Rigotti NA, Park ER, Chang Y, Regan S. Smoking cessation medication use among pregnant and postpartum smokers. Obstetrics & Gynecology. 2008; 111(2 Pt 1):348–55.
234. Rigotti, N. A., Park, E. R., Regan, S., Chang, Y., Perry, K., Loudin, B., & Quinn, V. (2006). Efficacy of telephone counseling for pregnant smokers: A randomized controlled trial. Obstetrics and Gynecology, 108(1), 83–92.
235. Rigotti, N.; Park, E.; Regan, S.; Chang, Y.; Perry, K.; Loudin, B., et al. Efficacy of proactive telephone counseling for pregnant smokers: a randomized trial. Society for Research on Nicotine and Tobacco 12th Annual Meeting; Orlando, Florida, USA. 2006 February 15-18; 2006. p. 22
236. Rigotti, N.; Park, E.; Regan, S.; Chang, Y.; Perry, K.; Loudin, B., et al. The efficacy of telephone couseling for pregnant smokers: a randomized controlled trial [abstract]. 13th World Conference on Tobacco or Health; Washington DC, USA. 2006 July 12-15; 2006
237. Robling M, Bekkers MJ, Bell K, Butler CC, CanningsJohn R, Channon S, et al. Effectiveness of a nurse-led intensive home-visitation programme for first-time teenage mothers (Building Blocks): a pragmatic randomised controlled trial. Lancet 2016.387(10014):146–55
238. Ruger, J. P., Weinstein, M. C., Hammond, S. K., Kearney, M. H., & Emmons, K. M. (2008). Cost-effectiveness of motivational interviewing for smoking cessation and relapse prevention among low-income pregnant women: a randomized controlled trial. Value in Health, 11(2), 191–198.
239. Secker-Walker RH, Mead PB, Goodwin GD, Lepage SS, Skelly JM, Flynn BS, et al. Individualised smoking cessation counseling during prenatal and early postnatal care. American Journal of Obstetrics and Gynecology 1994 71:1347–55
240. Secker-Walker RH, Solomon LJ, Flynn BS, LePage SS, Crammond JE, Worden JK, et al. Training obstetric and family practice residents to give smoking cessation advice during prenatal care. American Journal of Obstetrics and Gynecology. 1992; 166:1356–63.
241. Secker-Walker RH, Solomon LJ, Flynn BS, Skelly JM, Lepage SS, Goodwin GD, et al. Smoking relapse prevention counseling during prenatal and early postnatal care. American Journal of Preventive Medicine. 1995; 11(2):86–93.
242. Secker-Walker RH, Solomon LJ, Flynn BS, Skelly JM, Mead PB. Reducing smoking during pregnancy and postpartum: physician’s advice supported by individual counselling. Preventive Medicine 1998.27:422–30
243. Secker-Walker RH, Solomon LJ, Geller BM, Flynn BS, Worden JK, Skelly JM, et al. Modeling smoking cessation: exploring the use of a videotape to help pregnant women quit smoking. Women & Health 1997.25:23–35
244. Secker-Walker RH, Vacek PM, Flynn BS, Mead PB. Estimated gains in birth weight associated with reductions in smoking during pregnancy. Journal of Reproductive Medicine. 1998; 43(11):967–74.
245. Secker-Walker RH, Vacek PM, Flynn BS, Mead PB. Smoking in pregnancy, exhaled carbon monoxide, and birth weight. Obstetrics and Gynecology. 1997; 89:648–53.
246. Secker-Walker, R. H., Solomon, L. J., Flynn, B. S., Skelly, J. M., & Mead, P. B. (1998). Reducing smoking during pregnancy and postpartum: Physician’s advice supported by individual counseling. Preventive Medicine, 27(3), 422–430.
247. Secker-Walker, R. H., Solomon, L. J., Flynn, B. S., Skelly, J. M., Lepage, S. S., Goodwin, G. D., & Mead, P. B. (1994). Individualized smoking cessation counseling during prenatal and early postnatal care. American Journal of Obstetrics and Gynecology, 171(5), 1347–1355
248. Sexton M, Hebel JR. A clinical trial of change in maternal smoking and its effect on birth weight. JAMA 1984.251: 911–5
249. Sexton M, Nowicki P, Hebel JR. Verification of smoking status by thiocyanate in unrefrigerated, mailed saliva samples. Preventive Medicine. 1986; 15(1):28–34.
250. Sierto FW, Hannon WH, Kendrick JS, Bernert JT, Pirkle J, Gargiullo PM. Urinary cotinine levels in women enrolled in a smoking cessation study during and after pregnancy. Journal of Smoking-Related Disease.
251. 1994; 5:65–76."
252. Solomon LJ, Higgins ST, Heil SH, Badger GJ, Thomas CS, Bernstein IM. Predictors of postpartum relapse to smoking. Drug and Alcohol Dependence. 2007; 90:224–7. [
253. Solomon LJ, Secker-Walker RH, Flynn BS, Skelly JM, Capeless EL. Proactive telephone peer support to help pregnant women stop smoking. Tobacco Control 2000.9 Suppl 3:iii72–iii74
254. Solomon LJ, Secker-Walker RH, Skelly JM, Flynn BS. Stages of change in smoking during pregnancy in low risk women. Journal of Behavioral Medicine. 1996; 19:333–4.
255. Stanton WR, Lowe JB, Moffatt J, DelMar CB. Randomised control trial of a smoking cessation intervention directed at men whose partners are pregnant. Preventive Medicine. 2004; 38:6–9.
256. Stotts AL, DeLaune KA, Schmitz JM, Grabowski J. Impact of a motivational intervention on mechanisms of change in low-income pregnant women. Addictive Behaviors. 2004; 29(8):1649–57.
257. Stotts AL, Groff JY, Velasquez MM, Benjamin-Garner R, Green C, Carbonari JP, et al. Ultrasound feedback and motivational interviewing targeting smoking cessation in the second and third trimesters of pregnancy. Nicotine & Tobacco Research 2009.11(8):961–8
258. Stotts, A. L., DiClemente, C. C., & Dolan-Mullen, P. (2002). One-to-one: A motivational intervention for resistant pregnant smokers. Addictive Behaviors, 27(2), 275–292
259. Stotts, A. L., Groff, J. Y., Velasquez, M. M., Benjamin-Garner, R., Green, C., Carbonari, J. P., & DiClemente, C. C. (2009). Ultrasound feedback and motivational interviewing targeting smoking cessation in the second and third trimesters of pregnancy. Nicotine & Tobacco Research, 11(8):961-968
260. Stotts, AL.; Schmitz, JM.; Shipley, SL.; Delaune, KA.; Grabowski, J. Impact of a motivational interviewing intervention on mechanisms of change in low-income pregnant smokers (POS4-32) [abstract]. Society for Research on Nicotine and Tobacco 9th Annual Meeting; New Orleans, Louisiana. 2003 February 19-22; 2003. p. 89
261. Strecher VJ, Bishop KR, Bernhardt J, Thorp JM, Cheuvrout B, Potts P. Quits for keeps: tailored smoking cessation guides for pregnancy and beyond. Tobacco Control 2000.9 (Suppl 3):iii78–iii79
262. Subramanian S, Katz KS, Rodam M, Gantz MG, El-Khorazaty NM, Johnson A, et al. An integrated randomized intervention to reduce behavioural and psychosocial risks: Pregnancy and neonatal outcomes. Maternal Child Health Journal. 2012; 16:545–54.
263. Suplee P.D. The importance of providing smoking relapse counseling during the postpartum hospitalization. Journal of Obstetric, Gynecologic, and Neonatal Nursing. 2005;34(6):703–712.
264. Svanberg B. Smoking during pregnancy: possibilities of prevention in antenatal care. International Journal of Technology Assessment in Health Care. 1992; 8(Suppl 1):96–100.
265. Tan S, Courtney LP, El-Mohandes AAE, Gantz MG, Blake SM, Thornberry J, et al. Relationships between self-reported smoking, household environmental tobacco smoke exposure and depressive symptoms in a pregnant minority population. Maternal Child Health Journal. 2011; 15:S65–S74.
266. Tappin D, Bauld L, Purves D, Boyd K, Sinclair L, MacAskill S et al (2015) Financial incentives for smoking cessation in pregnancy: Randomised controlled trial. BMJ, 350:h134
267. Tappin DM, Lumsden MA, Gilmour WH, Crawford F, McIntyre D, Stone DH, et al. Randomised controlled trial of home based motivational interviewing by midwives to help pregnant smokers quit or cut down. BMJ 2005.331: 373–7
268. Tappin DM, Lumsden MA, McIntyre D, McKay C, Gilmour WH, Webber R, et al. A pilot study to establish a randomized trial methodology to test the efficacy of a behavioural intervention. Health Education Research 2000 15(4):491–502
269. Tappin DM, Lumsden MA, McKay C, McIntyre D, Gilmour H, Webber R, et al. The effect of home-based motivational interviewing on the smoking behaviour of pregnant women: a pilot randomised controlled efficacy study. Ambulatory Child Health. 2000; 6(Suppl 1):34–5.
270. Thornton L. Smoking and Pregnancy: Feasibility and Effectiveness of a Smoking Intervention Programme Among Pregnant Women [thesis]. Dublin: Dept of Public Health, 1997
271. Tombor I, Beard E, Brown J, Shahab L, Michie S, West R. Randomized factorial experiment of components of the SmokeFree Baby smartphone application to aid smoking cessation in pregnancy. Transl Behav Med 2019 Jul 16;9(4):583-593 [FREE Full text] [doi: 10.1093/tbm/iby073] [Medline: 30011020]
272. Tsoh JY, Kohn MA, Gerbert B. Promoting smoking cessation in pregnancy with Video Doctor plus provider cueing: a randomized trial. Acta Obstetricia et Gynecologica Scandinavica 2010.89(4):515–23
273. Tuten M, Fitzsimons H, Chisolm MS, Nuzzo PA, Jones HE. Contingent incentives reduce cigarette smoking among pregnant, methadone-maintained women: results of an initial feasibility and efficacy randomized clinical trial. Addiction 2012.107(10):1868–77
274. Ussher M, Lewis S, Aveyard P, Manyonda I, West R, Lewis B, et al. Physical activity for smoking cessation in pregnancy: randomised controlled trial. BMJ (Clinical Research Ed.) 2015.350:h2145
275. Valanis, B., Lichtenstein, E., Mullooly, J. P., et al. (2001). Maternal smoking cessation and relapse prevention during health care visits. American Journal of Preventive Medicine, 20(1), 1–8.
276. Valbo A, Eide T. Smoking cessation in pregnancy: the effect of hypnosis in a randomized study. Addictive Behaviors 1996.21:29–35
277. Valbo A, Nylander G. Smoking cessation in pregnancy: intervention among heavy smokers. Acta Obstetricia et Gynecologica Scandinavica 1994.73:215–9
278. van Mierlo T, Fournier R, Jean-Charles A, Hovington J, Ethier I, Selby P. I’ll txt u if i have a problem: how the Société? Canadienne du cancer in Quebec applied behavior-change theory, data mining and agile software development to help young adults quit smoking. PLoS One. 2014;9(3):e91832. 30
279. Victora CG, Langer A, Barros F, Belizan J, Farnot U, Villar J, et al. The Latin American Multicenter Trial on psychosocial support during pregnancy: methodology and baseline comparability. Controlled Clinical Trials. 1994; 15:379–94
280. Vilches P. Consumption of Tobacco in Pregnant Women: Proposal of a Psychological Intervention Model in the Public Health System of Andalucia [thesis] Malaga: Univesidad di Malaga, 2009
281. Vilches, P. Doctoral Thesis. Univesidad di Malaga; 2009. Consumption of tobacco in pregnant women: Proposal of a psychological intervention model in the public health system of Andalucia. [Consumo de tabaco en mujeres gestantes: propuesta de un modelo de intervención psicológica en el sistema sanitario público de Andalucía].
282. Villar J, Farnot U, Barros F, Victora C, Langer A, Belizan JM. A randomized trial of psychosocial support during high-risk pregnancies. New England Journal of Medicine. 1992; 327:1266–71.
283. Wall, M. A., Severson, H. H., Andrews, J. A., Lichtenstein, E., & Zoref, L. (1995). Pediatric office-based smoking intervention: Impact on maternal smoking and relapse. Pediatrics, 96(4 Pt 1), 622–628.
284. Walsh RA, Melmeth A, Byrne JM, Brinsmead MW, Redman S. A smoking cessation program at a public antenatal clinic. American Journal of Public Health 1997.87: 1201–4
285. Walsh RA, Redman S, Byrne JM, Melmeth A, Brinsmead MW. Process measures in an antenatal smoking cessation trial: another part of the picture. Health Education Research. 2000; 15(4):469–83.
286. Walsh, R. A., Redman, S., Brinsmead, M. W., Byrne, J. M., & Melmeth, A. (1997). A smoking cessation program at a public antenatal clinic. American Journal of Public Health, 87(7), 1201–1204.
287. Washio Y, Higgins ST, Heil SH, Badger GJ, Skelly J, Bernstein IM, et al. Examining maternal weight gain during contingency-management treatment for smoking cessation among pregnant women. Drug and Alcohol Dependence. 2011; 114(1):73–6
288. Wesselink SFO, Lingsma HF, Robben PBM, et al. Provision and effect of quit-smoking counselling by primary care midwives. Midwifery 2015;31:986–92.
289. Wilkinson SA, McIntyre HD. Evaluation of the ’healthy start to pregnancy’ early antenatal health promotion workshop: a randomized controlled trial. BMC Pregnancy and Childbirth 2012.0.590972222222222
290. Windsor R, Woodby L, Miller T, Hardin M. Effectiveness of Smoking Cessation and Reduction in Pregnancy Treatment (SCRIPT) methods in Medicaid-supported prenatal care: Trial III. Health Education & Behavior 2011.38(4):412–22
291. Windsor RA, Contreras L, Artz L, Lowe JB. Smoking cessation and pregnancy intervention trial: preliminary mid-trial results. Progress in Clinical and Biological Research. 1990; 339:107–17.
292. Windsor RA, Li CQ, Boyd NR, Hartmann KE. The use of significant reduction rates to evaluate health education methods for pregnant smokers: a new harm reduction behavioural indicator. Health Education and Behavior. 1999; 26:648–61.
293. Windsor RA, Lowe JB, Perkins LL, Smith-Yoder D, Artz L, Crawford M, et al. Health education for pregnant smokers: its behavioral impact and cost benefit. American Journal of Public Health. 1993; 83:201–6.
294. Windsor RA, Spanos D, Samuelsson C, Bartlett EE, Manzella B, Reese Y, et al. The effectiveness of smoking cessation methods for smokers in public health maternity clinics: a randomized trial. American Journal of Public Health 1985.75:1389–92
295. Windsor RA, Warner KE, Cutter GR. A cost-effectiveness analysis of self-help smoking cessation methods for pregnant women. Public Health Reports. 1988; 103(1):83–8.
296. Windsor RA. The efficacy and cost-effectiveness of smoking cessation methods for pregnant women. Southern Medical Journal. 1986; 79:34.*
297. Winickoff J.P., Healey E.A., Regan S. Using the postpartum hospital stay to address mothers' and fathers' smoking: The NEWS Study. Pediatrics. 2010;125(3):518.
298. Wisborg, K., Henriksen, T. B., Jespersen, L. B., & Secher, N. J. (2000). Nicotine patches for pregnant smokers: A randomized controlled study. Obstetrics and Gynecology, 96(6), 967–971
299. Wisborg K, Henriksen TB, Secher NJ. A prospective intervention study of stopping smoking in pregnancy in a routine antenatal care setting. Br J Obstet Gynaecol 1998;105:1171–6.
300. Yoon JH, Higgins ST, Heil SH, Sugarbaker RJ, Thomas CS, Badger GJ. Delay discounting predicts postpartum relapse to cigarette smoking among pregnant women. Experimental and Clinical Psychopharmacology. 2007; 15(2):176–86.
301. Zapka J, Goins KV, Pbert L, Ockene JK. Translating efficacy research into effectiveness studies in practice: lessons from research to promote smoking cessation in community health centers. Health Promotion Practice. 2004; 5(3):245–55.
302. Zapka JG, Pbert L, Stoddard AM, Ockene JK, Goins KV, Bonollo D. Smoking cessation counseling with pregnant and postpartum women: a survey of community health center providers. American Journal of Public Health. 2000; 90:78–84.on PR, Heminger CL, et al. Quit4baby: results from a pilot test of a mobile smoking cessation program for pregnant women. JMIR Mhealth Uhealth. 2015;3(1):e10
303. Zhang X, Devasia R, Czarnecki G, et al. Effects of incentive-based smoking cessation program for pregnant women on birth outcomes. Matern Child Health J 2017;21:745–51.

**Included studies in diet and/or physical activity reviews**

| **Review** | **References of included diet/PA interventions delivered in pregnancy** |
| --- | --- |
| Agha *et al.* 2014 | 1. Claesson IM, Sydsjo¨ G, Brynhildsen J, Cedergren M, Jeppsson A, et al. (2008) Weight gain restriction for obese pregnant women: a case-control intervention study. BJOG. [Online]; 115(1): 44–50 2. Guelinckx I, Devlieger R, Mullie P, Vansant G (2010) Effect of lifestyle intervention on dietary habits, physical activity, and gestational weight gain in obese pregnant women: a randomized controlled trial. Am J Clin Nutr. [Online] 2010; 91(2): 373–380 3. Haakstad LA, Bø K (2011). Effect of regular exercise on prevention of excessive weight gain in pregnancy: A randomised controlled trial. The European Journal of Contraception and Reproductive Health Care;16: 116–125 4. Hui AL, Ludwig SM, Gardiner P, Sevenhuysen G, Murray R, et al (2006). Community-based exercise and dietary intervention during pregnancy: A pilot study. Canadian Journal of Diabetes;30(2): 169–175. 5. Ilmonen J, Isolauri E, Poussa T, Laitinen K (2011) Impact of dietary counselling and probiotic intervention on maternal anthropometric measurements during and after pregnancy: A randomized placebo-controlled trial. Clinical Nutrition. [Online] 2011; 30(2): 156–164. 6. Jackson RA, Stotland NE, Caughey AB, Gerbert B (2011) Improving diet and exercise in pregnancy with Video Doctor counseling: A randomized trial. Patient Education and Counselling. [Online]; 83(2): 203–209 7. Kinnunen TI, Pasanen M, Aittasalo M, Fogelholm M, Hilakivi-Clarke L, et al. (2007) Preventing excessive weight gain during pregnancy – a controlled trial in primary health care. Eur J Clin Nutr. [Online]; 61(7): 884–891 8. Phelan S, Phipps MG, Abrams B, Darroch F, Schaffner A, et al. (2011) Randomized trial of a behavioural intervention to prevent excessive gestational weight gain: The Fit for delivery study. American Journal of Clinical Nutrition. [Online] 2011; 93(4): 772–779 9. Polley BA, Wing RR, Sims CJ (2002). Randomized controlled trial to prevent excessive weight gain in pregnant women. Int J Obes Relat Metab Disord; 26(11): 1494–1502. 10. Weisman CS, Hillemeier MM, Downs DS, Feinberg ME, Chuang CH, et al. (2011) Improving Women’s Preconceptional Health: Long-Term Effects of the Strong Healthy Women Behaviour Change Intervention in the Central Pennsylvania Women’s Health Study. Women’s Health Issues. [Online]; 265– 271. A 11. Wolff S, Legarth J, Vangsgaard K, Toubro S, Astrup A (2008). A randomized trial of the effects of dietary counseling on gestational weight gain and glucose metabolism in obese pregnant women. Int J Obes (Lond); 32(3): 495–501 |
| Allen *et al.* 2014 | 1. Bogaerts A, Devlieger R, Nuyts E, Witters I, Gyselaers W,Van den Bergh BRH. Effects of lifestyle intervention inobese pregnant women on gestational weight gain andmental health: a randomised controlled trial. Int J Obesity.2012;36:1–8. 2. Crowther C, Hiller J, Moss J, McPhee A, Jeffries W,Robinson J. Effect of treatment of gestational diabetesmellitus on pregnancy outcomes. N Engl J Med.2005;352:2477–86 3. D’Almeida A, Carter JP, Anatol A, Prost C. Effects of acombination of evening primrose oil (gamma linolenicacid) and fish oil (eicosapentaenoic+docahexaenoic acid)versus magnesium, and versus placebo in preventingpreeclampsia. Women Health. 1992;19:117–31 4. Guelinckx I, Devlieger R, Mullie P, Vansant G. Effect oflifestyle intervention on dietary habits, physical activityand gestational weight gain in obese pregnant women: arandomised controlled trial. Am J Clin Nutr. 2010;91:373–80 5. Jefferies K, Shub A, Walker S, Hiscock R, Permezel M.Reducing excessive weight gain in pregnancy:a randomised controlled trial. Med J Aust. 2009;191:429–33 6. Khoury J, Henriksen T, Christophersen B, Tonstad S.Effect of cholesterol-lowering diet on maternal, cord andneonatal lipids, and pregnancy outcome: a randomisedclinical trial. Am J Obstet Gynecol. 2005;193:1292–301 7. Landon M, Spong C, Thom E, Carpenter MW, Ramins S,Casey B, et al. A multicenter, randomised trial oftreatment for mild gestational diabetes. N Engl J Med.2009;361:1339–48 8. Olsen SF, Secher NJ, Tabor A, Weber T, Walker JJ, GluudC. Randomised clinical trials of fish oil supplementation inhigh risk pregnancies. BJOG. 2000;107:382–95 9. Onwude JL, Lilford RJ, Hjartardottir H, Staines A, TuffnellD. A randomised double blind placebo controlled trial offish oil in high risk pregnancy. BJOG. 1995;102:95–100 10. Phelan S, Phipps M, Abrams B, Darroch F, Schaffner A,Wing R. Randomised trial of behavioural intervention toprevent excessive gestational weight gain: the fit fordelivery study. Am J Clin Nutr. 2011;93:772-9 11. Polley B, Wing R, Sims C. Randomised controlled trial toprevent excessive weight gain in pregnant women. Int JObesity. 2002;26:1494–502 12. Rae A, Bond D, Evans S, North F, Roberman B, Walters B.A randomised controlled trial of dietary energy restrictionin the management of obese women with gestationaldiabetes. Aust N Z J Obstet Gynaecol. 2000;40:416–22 13. Salvig J, Olsen S, Secher N. Effects of fish oilsupplementation in late pregnancy on blood pressure: arandomised controlled trial. BJOG. 1996;103:529–33 14. Smuts CM, Huang M, Mundy D, Plasse T, Major S,Carlson SE. A randomised trial of docosahexaenoic acidsupplementation during the third trimester of pregnancy.Obstet Gynecol. 2003;101:469–79 15. Thornton Y, Smarkola C, Kopacz SM, Ishoof S. Perinataloutcomes in nutritionally monitored obese pregnantwomen: a randomised clinical trial. J Natl Med Assoc.2009;101:569–77 16. Vinter C, Jensen DM, Oveson P, Henning B-N, JorgensenJS. The LiP (Lifestyle in Pregnancy) study. Diabetes Care.2011;34:2502–7 17. Wolff S, Legarth J, Vangsgaard K, Toubro S, Astrup A.A randomised trial of the effects of dieatry counselingon gestational weight gain and glucose metabolism inobese pregnant women. Int J Obesity. 2008;32:495–501 18. Zhou S, Yelland L, McPhee A, Quinlivan J, Gibson R,Makrides M. Fish oil supplementation in pregnancy doesnot reduce the risk of gestational diabetes or preeclampsia.Am J Clin Nutr. 2012;95:1378–84 |
| Baradwan *et al.* 2024 | 1. Aktan, B., F. Kayıkçıoğlu, and T. Akbayrak. 2021. “The Comparison of the Effects of Clinical Pilates Exercises with and without Childbirth Training on Pregnancy and Birth Results.” International Journal of Clinical Practice 75 (10): e14516. https://doi.org/10.1111/ijcp.14516 . 2. Ghandali, N. Y., M. Iravani, A. Habibi, and B. Cheraghian. 2021. “The Effectiveness of a Pilates Exercise Program During Pregnancy on Childbirth Outcomes: A Randomised Controlled Clinical Trial.” BMC Pregnancy and Childbirth 21 (1): 480. <https://doi.org/10.1186/s12884-021-03922-2> 3. Güder, D. S., M. Yalvaç, and G. Vural. 2018. “The Effect of Pregnancy Pilates-Assisted Childbirth Preparation Training on Childbirth Fear and Neonatal Outcomes: A Quasi-Experimental/quantitative Research.” Quality & Quantity 52 (6): 2667–79. https://doi.org/10.1007/s11135-018-0682-0 . 4. Kırıcı, P., M. C. Nacar, T. Karaçor, M. Bülbül, M. Baktıroğlu, N. Mavral, and N. Kaya. 2021. “Pilates Workouts Can Improve the Labor and Newborn Outcomes: A Case Control Study.” Journal of Surgery and Medicine 5 (6): 642–7. https://doi.org/10.28982/josam.951898 . 5. Mazzarino, M., D. Kerr, and M. E. Morris. 2022. “Feasibility of Pilates for Pregnant Women: A Randomised Trial.” Journal of Bodywork and Movement Therapies 32:207–12. <https://doi.org/10.1016/j.jbmt.2022.05.001> 6. Rodríguez-Díaz, L., C. Ruiz-Frutos, J. M. Vázquez-Lara, J. Ramírez-Rodrigo, C. Villaverde-Gutiérrez, and G. TorresLuque. 2017. “Effectiveness of a Physical Activity Programme Based on the Pilates Method in Pregnancy and Labour.” Enfermeria Clinica 27 (5): 271–7. <https://doi.org/10.1016/j.enfcli.2017.05.008> 7. Yaman, S., Ö. B. Tulmaç, B. Canarslan, and N. Hançerlioğullari. 2020. “Effect of Pilates During Pregnancy on Delivery Outcomes.” Journal of Health Sciences and Medicine 3 (4): 442–7. https://doi.org/10.32322/jhsm.790126 |
| Beetham *et al.* 2019 | 1. Bell RJ, Palma SM, Lumley JM. The effect of vigorous exercise during pregnancy on birth-weight. Aust N Z J Obstet Gynaecol. 1995;35(1):46–51. 2. Bisson M, Almeras N, Dufresne SS, Robitaille J, Rheaume C, Bujold E, et al. A 12-week exercise program for pregnant women with obesity to improve physical activity levels: An open randomised preliminary study. PLoS One. 2015;10(9):e0137742. 3. Cavalcante Sergio R, Cecatti Jose G, Pereira Rosa I, Baciuk Erica P, Bernardo Ana L, Silveira C. Water aerobics II: maternal body composition and perinatal outcomes after a program for low risk pregnant women. Reprod Health. 2009;6(1):1. 4. Collings C, Curet L, Mullin J. Maternal and fetal responses to a maternal aerobic exercise program. Am J Obstet Gynecol. 1983;145(6):702–7. 5. Hall DC, Kaufmann DA. Effects of aerobic and strength conditioning on pregnancy outcomes. Am J Obstet Gynecol. 1987;157(5):1199–203. 6. Hegaard HK, Petersson K, Hedegaard M, Ottesen B, Dykes AK, Henriksen TB, et al. Sports and leisure-time physical activity in pregnancy and birth weight: a population-based study. Scand J Med Sci Sports. 2010;20(1):e96– 102. 7. Hopkins SA, Baldi JC, Cutfield WS, McCowan L, Hofman PL. Exercise training in pregnancy reduces offspring size without changes in maternal insulin sensitivity. J Clin Endocrinol Metab. 2010;95(5):2080–8. 8. Kuhrt K, Harmon M, Hezelgrave NL, Seed PT, Shennan AH. Is recreational running associated with earlier delivery and lower birth weight in women who continue to run during pregnancy? An international retrospective cohort study of running habits of 1293 female runners during pregnancy. BMJ Open Sport Exerc Med. 2018;4:e000296. 9. Magann EF, Evans SF, Weitz B, Newnham J. Antepartum, intrapartum, and neonatal significance of exercise on healthy low-risk pregnant working women. Obstet Gynecol. 2002;99(3):466–72. 10. McCowan LME, Roberts CT, Dekker GA, Taylor RS, Chan EHY, Kenny LC, et al. Risk factors for small-for-gestational-age infants by customised birthweight centiles: data from an international prospective cohort study. BJOG. 2010; 117(13):1599–607. 11. Rose NC, Haddow JE, Palomaki GE, Knight GJ. Self-rated physical activity level during the second trimester and pregnancy outcome. Obstet Gynecol. 1991;78(6):1078–80. 12. Ruchat HS-M, Davenport MM, Giroux SI, Hillier FM, Batada FA, Sopper FM, et al. Nutrition and exercise reduce excessive weight gain in normal-weight pregnant women. Med Sci Sports Exerc. 2012a;44(8):1419–26. 13. Ruchat SM, Davenport MH, Hillier I, Batada A, Sopper J, Hammond M, et al. Walking program of low or vigorous intensity during pregnancy confers an aerobic benefit. Int J Sports Med. 2012b;33(8):661–6. 14. Sternfeld B, Quesenberry CP Jr, Eskenazi B, Newman LA. Exercise during pregnancy and pregnancy outcome. Med Sci Sports Exerc. 1995;27(5):634– 40. 15. Wang C, Wei Y, Zhang X, Zhang Y, Xu Q, Sun Y, et al. A randomized clinical trial of exercise during pregnancy to prevent gestational diabetes mellitus and improve pregnancy outcome in overweight and obese pregnant women. Am J Obstet Gynecol. 2017;216(4):340–51. 16. Zeanah M, Schlosser SP. Adherence to ACOG guidelines on exercise during pregnancy: effect on pregnancy outcome. J Obstet Gynecol Neonatal Nurs. 1993;22(4):329–35. |
| Bennett *et al.* 2023 | 1. Barakat R, Cordero Y, Coteron J, et al. Exercise during pregnancy improves maternal glucose screen at 24-28 weeks: a randomised controlled trial. Br J Sports Med. 2012;46(9):656–661. 2. Barakat R, Pelaez M, Lopez C, et al. Exercise during pregnancy and gestational diabetes-related adverse effects: a randomised controlled trial. Br J Sports Med. 2013;47(10):630–636 3. Barakat R, Perales M, Bacchi M, et al. A program of exercise throughout pregnancy. Is it safe to mother and newborn? Am J Health Promot. 2014;29(1):2–8. 4. Barakat R, Pelaez M, Cordero Y, et al. Exercise during pregnancy protects against hypertension and macrosomia: randomized clinical trial. Am J Obstet Gynecol. 2016;214(5):649.e1–649.e8 5. Barakat R, Refoyo I, Coteron J, et al. Exercise during pregnancy has a preventative effect on excessive maternal weight gain and gestational diabetes. A randomized controlled trial. Braz J Phys Ther. 2019;23(2):148–155. 6. Bisson M, Alméras N, Dufresne SS, et al. A 12-week exercise program for pregnant women with obesity to improve physical activity levels: an open randomised preliminary study. PLoS One. 2015;10(9):e0137742. 7. Cordero Y, Mottola MF, Vargas J, et al. Exercise is associated with a reduction in gestational diabetes mellitus. Med Sci Sports Exerc. 2015;47(7):1328–1333 8. Daly N, Farren M, McKeating A, et al. A medically supervised pregnancy exercise intervention in obese women: a randomized controlled trial. Obstet Gynecol. 2017;130(5):1001–1010. 9. da Silva SG, Hallal PC, Domingues MR, et al. A randomized controlled trial of exercise during pregnancy on maternal and neonatal outcomes: results from the PAMELA study. Int J Behav Nutr Phys Act. 2017;14(1):175. 10. Garnaes KK, Mørkved S, Salvesen Ø, et al. Exercise training and weight gain in obese pregnant women: a randomized controlled trial (ETIP trial). PLoS Med. 2016;13(7):e1002079 11. Guelfi KJ, Ming Jing O, Crisp NA, et al. Regular exercise to prevent the recurrence of gestational diabetes mellitus: a randomized controlled trial. Obstet Gynecol. 2016;128(4):819–827 12. Oostdam N, van Poppel MNM, Wouters MGAJ, et al. No effect of the FitFor2 exercise programme on blood glucose, insulin sensitivity, and birthweight in pregnant women who were overweight and at risk for gestational diabetes: results of a randomised controlled trial. BJOG. 2012;119(9):1098–1107. 13. Pelaez M, Gonzalez-Cerron S, Montejo R, et al. Protective effect of exercise in pregnant women including those who exceed weight gain recommendations: a randomized controlled trial. Mayo Clin Proc. 2019;94(10):1951–1959. 14. Price BB, Amini SB, Kappeler K. Exercise in pregnancy: effect on fitness and obstetric outcomes-a randomized trial. Med Sci Sports Exerc. 2012;44(12):2263–2269 15. Rakhshani A, Nagarathna R, Mhaskar R, et al. The effects of yoga in prevention of pregnancy complications in high-risk pregnancies: a randomized controlled trial. Prev Med. 2012;55(4):333–340. 16. Ruiz JR, Perales M, Pelaez M, et al. Supervised exercise-based intervention to prevent excessive gestational weight gain: a randomized controlled trial. Mayo Clin Proc. 2013;88(12):1388–1397. 17. Stafne SN, Salvesen K, Romundstad PR, et al. Regular exercise during pregnancy to prevent gestational diabetes: a randomized controlled trial. Obstet Gynecol. 2012;119(1):29–36 18. Tomić V, Sporiš G, Tomić J, et al. The effect of maternal exercise during pregnancy on abnormal fetal growth. Croat Med J. 2013;54(4):362–368. 19. Ussher M, Lewis S, Aveyard P, et al. The London Exercise and Pregnant smokers (LEAP) trial: a randomised controlled trial of physical activity for smoking cessation in pregnancy with an economic evaluation. Health Technol Assess. 2015;19(84):1–135 20. Wang C, Wei Y, Zhang X, et al. A randomized clinical trial of exercise during pregnancy to prevent gestational diabetes mellitus and improve pregnancy outcome in overweight and obese pregnant women. Am J Obstet Gynecol. 2017;216(4):340–351. |
| Bernado *et al.* 2023 | 1. Bisson M, Alméras N, Dufresne SS, et al. A 12-week exercise program for pregnant women with obesity to improve physical activity levels: an open randomised preliminary study. PLoS One. 2015;10(9):e0137742. 2. Daly N, Farren M, McKeating A, et al. A medically supervised pregnancy exercise intervention in obese women: a randomized controlled trial. Obstet Gynecol. 2017;130(5):1001–1010. 3. Garnaes KK, Mørkved S, Salvesen Ø, et al. Exercise training and weight gain in obese pregnant women: a randomized controlled trial (ETIP trial). PLoS Med. 2016;13(7):e1002079 4. Nascimento S, Surita F, Parpinelli M, Siani S, Pinto e Silva J. The effect of an antenatal physical exercise programme on maternal/perinatal outcomes and quality of life in overweight and obese pregnant women: a randomised clinical trial. BJOG Int J Obstet Gynaecol. 2011; 118: 1455-1463. doi:10.1111/j.1471-0528.2011.03084.x 5. Ruiz JR, Perales M, Pelaez M, et al. Supervised exercise-based intervention to prevent excessive gestational weight gain: a randomized controlled trial. Mayo Clin Proc. 2013;88(12):1388–1397. 6. Seneviratne SN, Jiang Y, Derraik JGB, et al. Effects of antenatal exercise in overweight and obese pregnant women on maternal and perinatal outcomes: a randomised controlled trial. BJOG Int J Obstet Gynaecol. 2016; 123: 588-597. doi:10.1111/1471-0528.13738 7. Wang C, Wei Y, Zhang X, et al. A randomized clinical trial of exercise during pregnancy to prevent gestational diabetes mellitus and improve pregnancy outcome in overweight and obese pregnant women. Am J Obstet Gynecol. 2017; 216: 340-351. doi:10.1016/j.ajog.2017.01.037 |
| Cancela-Carral *et al*. 2022 | 1. Aguilar-Cordero M.J., Sánchez-García J.C., Blanque R.R., López A.M.S., Mur-Villar N. Moderate Physical Activity in an Aquatic Environment During Pregnancy (SWEP Study) and Its Influence in Preventing Postpartum Depression. J. Am. Psychiatr. Nurses Assoc. 2019;25:112–121. doi: 10.1177/1078390317753675. 2. Bacchi M., Rodríguez Y.C., Puente M.P., Mas C.L., Carballo R. Efecto sobre la frecuencia cardiaca del ejercicio en tierra/agua en embarazadas en el tercer trimestre de gestación: Un estudio comparativo. Calid. Vida Salud. 2011;4:59–67. 3. Bacchi M., Mottola M.F., Perales M., Refoyo I., Barakat R. Aquatic Activities during Pregnancy Prevent Excessive Maternal Weight Gain and Preserve Birth Weight: A Randomized Clinical Trial. Am. J. Health Promot. 2018;32:729–735. doi: 10.1177/0890117117697520. - 4. Barakat R., Cordero Y., Coteron J., Luaces M., Montejo R. Exercise during pregnancy improves maternal glucose screen at 24–28 weeks: A randomised controlled trial. Br. J. Sports Med. 2012;46:656–661. doi: 10.1136/bjsports-2011-090009. 5. Barakat R., Perales M., Cordero Y., Bacchi M., Mottola M.F. Influence of Land or Water Exercise in Pregnancy on Outcomes. Med. Sci. Sports Exerc. 2017;49:1397–1403. doi: 10.1249/MSS.0000000000001234. - 6. Cordero Y., Pelaez M., De Miguel M., Perales M., Barakat R. ¿Puede el ejercicio físico moderado durante el embarazo actuar como un factor de prevención de la diabetes gestacional? Int. J. Sport Sci. 2012;8:3–19. 7. Cordero Y., Mottola M.F., Vargas J., Blanco M., Barakat R. Exercise Is Associated with a Reduction in Gestational Diabetes Mellitus. Med. Sci. Sports Exerc. 2015;47:1328–1333. doi: 10.1249/MSS.0000000000000547. 8. Granath A.B., Hellgren M.S., Gunnarsson R.K. Water Aerobics Reduces Sick Leave due to Low Back Pain during Pregnancy. J. Obstet. Gynecol. Neonatal Nurs. 2006;35:465–471. doi: 10.1111/j.1552-6909.2006.00066.x. - 9. Rodríguez-Blanque R., Sánchez-García J.C., Sánchez-López A.M., Mur-Villar N., Fernández-Castillo R., Cordero M.J.A. Influencia del ejercicio físico durante el embarazo sobre el peso del recién nacido: Un ensayo clínico aleatorizado. Nutrición Hospitalaria. 2017;34:834–840. doi: 10.20960/nh.1095. - 10. Rodriguez-Blanque R., Sánchez-García J., Sánchez-López A., Mur-Villar N., Aguilar-Cordero M. The influence of physical activity in water on sleep quality in pregnant women: A randomised trial. Women Birth. 2018;31:e51–e58. doi: 10.1016/j.wombi.2017.06.018. - 11. Rodríguez-Blanque R., Garcia J.C.S., López A.M.S., Expósito-Ruiz M., Aguilar-Cordero M.J. Randomized Clinical Trial of an Aquatic Physical Exercise Program during Pregnancy. J. Obstet. Gynecol. Neonatal Nurs. 2019;48:321–331. doi: 10.1016/j.jogn.2019.02.003. 12. Rodríguez-Blanque R., Garcia J.C.S., López A.M.S., Aguilar-Cordero M.J. Physical activity during pregnancy and its influence on delivery time: A randomized clinical trial. PeerJ. 2019;7:e6370. doi: 10.7717/peerj.6370. 13. Sánchez-García J.C., Aguilar-Cordero M.J., Menor-Rodríguez M.J., Paucar-Sánchez A., Rodríguez Blanque R. Influencia del ejercicio físico en la evolución del peso gestacional y posparto. Ensayo clínico aleatorizado. Nutr. Hosp. 2019;36:1–8. doi: 10.20960/nh.02456. 14. Sillero-Quintana M., Conde-Pascual E., Gomez-Carmona P.M., Fernandez-Cuevas I., García-Pastor T. Effect of yoga and swimming on body temperature of pregnant women. Thermol. Int. 2012;22:108. 15. Smith S.A., Michel Y. A Pilot Study on the Effects of Aquatic Exercises on Discomforts of Pregnancy. J. Obstet. Gynecol. Neonatal Nurs. 2006;35:315–323. doi: 10.1111/j.1552-6909.2006.00045.x. - 16. Vallim A.L., Osis M.J., Cecatti J.G., Baciuk E.P., Silveira C., Cavalcante S.R. Water exercises and quality of life during pregnancy. Reprod. Health. 2011;8:14–17. doi: 10.1186/1742-4755-8-14. - 17. Vázquez-Lara J.M., Ruiz-Frutos C., Rodríguez-Díaz L., Ramírez-Rodrigo J., Villaverde-Gutiérrez C., Torres-Luque G. Effect of a physical activity programme in the aquatic environment on haemodynamic constants in pregnant women. Enfermería Clínica. 2018;28:316–325. doi: 10.1016/j.enfcli.2017.07.009 |
| Cantor et al.. 2021 | 1. Aguilar-Cordero  MJ, Sánchez-García  JC, Rodriguez-Blanque  R, Sánchez-López  AM, Mur-Villar  N.  Moderate physical activity in an aquatic environment during pregnancy (SWEP study) and its influence in preventing postpartum depression.   J Am Psychiatr Nurses Assoc. 2019;25(2):112-121. doi:10.1177/1078390317753675 2. Al Wattar  BH, Dodds  J, Placzek  A,  et al; ESTEEM Study Group.  Mediterranean-style diet in pregnant women with metabolic risk factors (ESTEEM): a pragmatic multicentre randomised trial.   PLoS Med. 2019;16(7):e1002857. doi:10.1371/journal.pmed.1002857PubMedGoogle Scholar 3. Altazan  AD, Redman  LM, Burton  JH,  et al.  Mood and quality of life changes in pregnancy and postpartum and the effect of a behavioral intervention targeting excess gestational weight gain in women with overweight and obesity: a parallel-arm randomized controlled pilot trial.   BMC Pregnancy Childbirth. 2019;19(1):50. doi:10.1186/s12884-019-2196-8PubMedGoogle ScholarCrossref 4. Althuizen  E, van der Wijden  CL, van Mechelen  W, Seidell  JC, van Poppel  MN.  The effect of a counselling intervention on weight changes during and after pregnancy: a randomised trial.   BJOG. 2013;120(1):92-99. doi:10.1111/1471-0528.12014PubMedGoogle ScholarCrossref 5. Asbee  SM, Jenkins  TR, Butler  JR, White  J, Elliot  M, Rutledge  A.  Preventing excessive weight gain during pregnancy through dietary and lifestyle counseling: a randomized controlled trial.   Obstet Gynecol. 2009;113(2, pt 1):305-312. doi:10.1097/AOG.0b013e318195baefPubMedGoogle ScholarCrossref 6. Assaf-Balut  C, García de la Torre  N, Durán  A,  et al.  A Mediterranean diet with additional extra virgin olive oil and pistachios reduces the incidence of gestational diabetes mellitus (GDM): a randomized controlled trial: the St. Carlos GDM Prevention Study.   PLoS One. 2017;12(10):e0185873. doi:10.1371/journal.pone.0185873PubMedGoogle Scholar 7. Bacchi  M, Mottola  MF, Perales  M, Refoyo  I, Barakat  R.  Aquatic activities during pregnancy prevent excessive maternal weight gain and preserve birth weight: a randomized clinical trial.   Am J Health Promot. 2018;32(3):729-735. doi:10.1177/0890117117697520PubMedGoogle ScholarCrossref 8. Barakat  R, Cordero  Y, Coteron  J, Luaces  M, Montejo  R.  Exercise during pregnancy improves maternal glucose screen at 24-28 weeks: a randomised controlled trial.   Br J Sports Med. 2012;46(9):656-661. doi:10.1136/bjsports-2011-090009PubMedGoogle ScholarCrossref 9. Barakat  R, Franco  E, Perales  M, López  C, Mottola  MF.  Exercise during pregnancy is associated with a shorter duration of labor: a randomized clinical trial.   Eur J Obstet Gynecol Reprod Biol. 2018;224:33-40. doi:10.1016/j.ejogrb.2018.03.009PubMedGoogle ScholarCrossref 10. Barakat  R, Pelaez  M, Cordero  Y,  et al.  Exercise during pregnancy protects against hypertension and macrosomia: randomized clinical trial.   Am J Obstet Gynecol. 2016;214(5):649.e1-649.e8. doi:10.1016/j.ajog.2015.11.039PubMedGoogle ScholarCrossref 11. Barakat  R, Perales  M, Bacchi  M, Coteron  J, Refoyo  I.  A program of exercise throughout pregnancy: is it safe to mother and newborn?   Am J Health Promot. 2014;29(1):2-8. doi:10.4278/ajhp.130131-QUAN-56PubMedGoogle ScholarCrossref 12. Barakat  R, Refoyo  I, Coteron  J, Franco  E.  Exercise during pregnancy has a preventative effect on excessive maternal weight gain and gestational diabetes: a randomized controlled trial.   Braz J Phys Ther. 2019;23(2):148-155. doi:10.1016/j.bjpt.2018.11.005PubMedGoogle ScholarCrossref 13. Bogaerts  AF, Devlieger  R, Nuyts  E, Witters  I, Gyselaers  W, Van den Bergh  BR.  Effects of lifestyle intervention in obese pregnant women on gestational weight gain and mental health: a randomized controlled trial.   Int J Obes (Lond). 2013;37(6):814-821. doi:10.1038/ijo.2012.162PubMedGoogle ScholarCrossref 14. Brownfoot  FC, Davey  MA, Kornman  L.  Routine weighing to reduce excessive antenatal weight gain: a randomised controlled trial.   BJOG. 2016;123(2):254-261. doi:10.1111/1471-0528.13735PubMedGoogle ScholarCrossref 15. Bruno  R, Petrella  E, Bertarini  V, Pedrielli  G, Neri  I, Facchinetti  F.  Adherence to a lifestyle programme in overweight/obese pregnant women and effect on gestational diabetes mellitus: a randomized controlled trial.   Matern Child Nutr. 2017;13(3):e12333. doi:10.1111/mcn.12333PubMedGoogle Scholar 16. Cahill  AG, Haire-Joshu  D, Cade  WT,  et al.  Weight control program and gestational weight gain in disadvantaged women with overweight or obesity: a randomized clinical trial.   Obesity (Silver Spring). 2018;26(3):485-491. doi:10.1002/oby.22070PubMedGoogle ScholarCrossref 17. Daley  AJ, Jolly  K, Jebb  SA,  et al.  Feasibility and acceptability of regular weighing, setting weight gain limits and providing feedback by community midwives to prevent excess weight gain during pregnancy: randomised controlled trial and qualitative study.   BMC Obes. 2015;2(1):35. doi:10.1186/s40608-015-0061-5PubMedGoogle ScholarCrossref 18. Daley  A, Jolly  K, Jebb  SA,  et al.  Effectiveness of a behavioural intervention involving regular weighing and feedback by community midwives within routine antenatal care to prevent excessive gestational weight gain: POPS2 randomised controlled trial.   BMJ Open. 2019;9(9):e030174. doi:10.1136/bmjopen-2019-030174PubMedGoogle Scholar 19. Daly  N, Farren  M, McKeating  A, OʼKelly  R, Stapleton  M, Turner  MJ.  A medically supervised pregnancy exercise intervention in obese women: a randomized controlled trial.   Obstet Gynecol. 2017;130(5):1001-1010. doi:10.1097/AOG.0000000000002267PubMedGoogle ScholarCrossref 20. Dodd  JM, Deussen  AR, Louise  J.  A randomised trial to optimise gestational weight gain and improve maternal and infant health outcomes through antenatal dietary, lifestyle and exercise advice: the OPTIMISE randomised trial.   Nutrients. 2019;11(12):E2911. doi:10.3390/nu11122911PubMedGoogle Scholar 21. Dodd  JM, Turnbull  D, McPhee  AJ,  et al; LIMIT Randomised Trial Group.  Antenatal lifestyle advice for women who are overweight or obese: LIMIT randomised trial.   BMJ. 2014;348:g1285. doi:10.1136/bmj.g1285PubMedGoogle ScholarCrossref 22. Gallagher  D, Rosenn  B, Toro-Ramos  T,  et al.  Greater neonatal fat-free mass and similar fat mass following a randomized trial to control excess gestational weight gain.   Obesity (Silver Spring). 2018;26(3):578-587. doi:10.1002/oby.22079PubMedGoogle ScholarCrossref 23. Garnæs  KK, Mørkved  S, Salvesen  Ø, Moholdt  T.  Exercise training and weight gain in obese pregnant women: a randomized controlled trial (ETIP trial).   PLoS Med. 2016;13(7):e1002079. doi:10.1371/journal.pmed.1002079PubMedGoogle Scholar 24. Garnæs  KK, Mørkved  S, Salvesen  KA, Salvesen  Ø, Moholdt  T.  Exercise training during pregnancy reduces circulating insulin levels in overweight/obese women postpartum: secondary analysis of a randomised controlled trial (the ETIP trial).   BMC Pregnancy Childbirth. 2018;18(1):18. doi:10.1186/s12884-017-1653-5PubMedGoogle ScholarCrossref 25. Gesell  SB, Katula  JA, Strickland  C, Vitolins  MZ.  Feasibility and initial efficacy evaluation of a community-based cognitive-behavioral lifestyle intervention to prevent excessive weight gain during pregnancy in Latina women.   Matern Child Health J. 2015;19(8):1842-1852. doi:10.1007/s10995-015-1698-xPubMedGoogle ScholarCrossref 26. Guelfi  KJ, Ong  MJ, Crisp  NA,  et al.  Regular exercise to prevent the recurrence of gestational diabetes mellitus: a randomized controlled trial.   Obstet Gynecol. 2016;128(4):819-827. doi:10.1097/aog.0000000000001632PubMedGoogle ScholarCrossref 27. Haakstad  LA, Bø  K.  Effect of regular exercise on prevention of excessive weight gain in pregnancy: a randomised controlled trial.   Eur J Contracept Reprod Health Care. 2011;16(2):116-125. doi:10.3109/13625187.2011.560307PubMedGoogle ScholarCrossref 28. Harrison  CL, Lombard  CB, Strauss  BJ, Teede  HJ.  Optimizing healthy gestational weight gain in women at high risk of gestational diabetes: a randomized controlled trial.   Obesity (Silver Spring). 2013;21(5):904-909. doi:10.1002/oby.20163PubMedGoogle ScholarCrossref 29. Hawkins  M, Hosker  M, Marcus  BH,  et al.  A pregnancy lifestyle intervention to prevent gestational diabetes risk factors in overweight Hispanic women: a feasibility randomized controlled trial.   Diabet Med. 2015;32(1):108-115. doi:10.1111/dme.12601PubMedGoogle ScholarCrossref 30. Herring  SJ, Cruice  JF, Bennett  GG, Rose  MZ, Davey  A, Foster  GD.  Preventing excessive gestational weight gain among African American women: a randomized clinical trial.   Obesity (Silver Spring). 2016;24(1):30-36. doi:10.1002/oby.21240PubMedGoogle ScholarCrossref 31. Hui  A, Back  L, Ludwig  S,  et al.  Lifestyle intervention on diet and exercise reduced excessive gestational weight gain in pregnant women under a randomised controlled trial.   BJOG. 2012;119(1):70-77. doi:10.1111/j.1471-0528.2011.03184.xPubMedGoogle ScholarCrossref 32. Hui  AL, Back  L, Ludwig  S,  et al.  Effects of lifestyle intervention on dietary intake, physical activity level, and gestational weight gain in pregnant women with different pre-pregnancy body mass index in a randomized control trial.   BMC Pregnancy Childbirth. 2014;14:331. doi:10.1186/1471-2393-14-331PubMedGoogle ScholarCrossref 33. Koivusalo  SB, Rönö  K, Klemetti  MM,  et al.  Gestational diabetes mellitus can be prevented by lifestyle intervention: the Finnish gestational diabetes prevention study (RADIEL): a randomized controlled trial.   Diabetes Care. 2016;39(1):24-30. Published correction appears in Diabetes Care. 2017 Aug;40(8):1133. doi:10.2337/dc15-0511PubMedGoogle ScholarCrossref 34. Kunath  J, Gunther  J, Rauh  K,  et al.  Effects of a lifestyle intervention during pregnancy to prevent excessive gestational weight gain in routine care—the cluster-randomised GeliS trial.   BMC Med. 2019;17(1):5. doi:10.1186/s12916-018-1235-zPubMedGoogle ScholarCrossref 35. Luoto  R, Kinnunen  TI, Aittasalo  M,  et al.  Primary prevention of gestational diabetes mellitus and large-for-gestational-age newborns by lifestyle counseling: a cluster-randomized controlled trial.   PLoS Med. 2011;8(5):e1001036. doi:10.1371/journal.pmed.1001036PubMedGoogle Scholar 36. Magriples  U, Boynton  MH, Kershaw  TS,  et al.  The impact of group prenatal care on pregnancy and postpartum weight trajectories.   Am J Obstet Gynecol. 2015;213(5):688.e1-688.e9. doi:10.1016/j.ajog.2015.06.066PubMedGoogle ScholarCrossref 37. McCarthy  EA, Walker  SP, Ugoni  A, Lappas  M, Leong  O, Shub  A.  Self-weighing and simple dietary advice for overweight and obese pregnant women to reduce obstetric complications without impact on quality of life: a randomised controlled trial.   BJOG. 2016;123(6):965-973. doi:10.1111/1471-0528.13919PubMedGoogle ScholarCrossref 38. Okesene-Gafa  KAM, Li  M, McKinlay  CJD,  et al.  Effect of antenatal dietary interventions in maternal obesity on pregnancy weight-gain and birthweight: Healthy Mums and Babies (HUMBA) randomized trial.   Am J Obstet Gynecol. 2019;221(2):152e.1-152e.13. doi:10.1016/j.ajog.2019.03.003PubMedGoogle ScholarCrossref 39. Olson  CM, Groth  SW, Graham  ML, Reschke  JE, Strawderman  MS, Fernandez  ID.  The effectiveness of an online intervention in preventing excessive gestational weight gain: the e-Moms Roc randomized controlled trial.   BMC Pregnancy Childbirth. 2018;18(1):148. doi:10.1186/s12884-018-1767-4PubMedGoogle ScholarCrossref 40. Peccei  A, Blake-Lamb  T, Rahilly  D, Hatoum  I, Bryant  A.  Intensive prenatal nutrition counseling in a community health setting: a randomized controlled trial.   Obstet Gynecol. 2017;130(2):423-432. doi:10.1097/AOG.0000000000002134PubMedGoogle ScholarCrossref 41. Pelaez  M, Gonzalez-Cerron  S, Montejo  R, Barakat  R.  Protective effect of exercise in pregnant women including those who exceed weight gain recommendations: a randomized controlled trial.   Mayo Clin Proc. 2019;94(10):1951-1959. doi:10.1016/j.mayocp.2019.01.050PubMedGoogle ScholarCrossref 42. Petrella  E, Malavolti  M, Bertarini  V,  et al.  Gestational weight gain in overweight and obese women enrolled in a healthy lifestyle and eating habits program.   J Matern Fetal Neonatal Med. 2014;27(13):1348-1352. doi:10.3109/14767058.2013.858318PubMedGoogle ScholarCrossref 43. Perales  M, Rodríguez  YC, Terrones  MV, Mulas  AL, Carballo  RB.  Exercise and depression in overweight and obese pregnant women: a randomised controlled trial.   Archivos de Medicina del Deporte. 2015(167):156-163. http://archivosdemedicinadeldeporte.com/articulos/upload/or04_perales.pdfGoogle Scholar 44. Phelan  S, Phipps  MG, Abrams  B, Darroch  F, Schaffner  A, Wing  RR.  Randomized trial of a behavioral intervention to prevent excessive gestational weight gain: the Fit for Delivery Study.   Am J Clin Nutr. 2011;93(4):772-779. doi:10.3945/ajcn.110.005306PubMedGoogle ScholarCrossref 45. Phelan  S, Wing  RR, Brannen  A,  et al.  Randomized controlled clinical trial of behavioral lifestyle intervention with partial meal replacement to reduce excessive gestational weight gain.   Am J Clin Nutr. 2018;107(2):183-194. doi:10.1093/ajcn/nqx043PubMedGoogle ScholarCrossref 46. Polley  BA, Wing  RR, Sims  CJ.  Randomized controlled trial to prevent excessive weight gain in pregnant women.   Int J Obes Relat Metab Disord. 2002;26(11):1494-1502. doi:10.1038/sj.ijo.0802130PubMedGoogle ScholarCrossref 47. Rauh  K, Gabriel  E, Kerschbaum  E,  et al.  Safety and efficacy of a lifestyle intervention for pregnant women to prevent excessive maternal weight gain: a cluster-randomized controlled trial.   BMC Pregnancy Childbirth. 2013;13:151. doi:10.1186/1471-2393-13-151PubMedGoogle ScholarCrossref 48. Redman  LM, Gilmore  LA, Breaux  J,  et al.  Effectiveness of SmartMoms, a novel eHealth intervention for management of gestational weight gain: randomized controlled pilot trial.   JMIR Mhealth Uhealth. 2017;5(9):e133. doi:10.2196/mhealth.8228PubMedGoogle Scholar 49. Renault  KM, Norgaard  K, Nilas  L,  et al.  The Treatment of Obese Pregnant women (TOP) study: a randomized controlled trial of the effect of physical activity intervention assessed by pedometer with or without dietary intervention in obese pregnant women.   Am J Obstet Gynecol. 2013;210(2):134.e1-134.e9. doi:10.1016/j.ajog.2013.09.029PubMedGoogle ScholarCrossref 50. Ronnberg  AK, Ostlund  I, Fadl  H, Gottvall  T, Nilsson  K.  Intervention during pregnancy to reduce excessive gestational weight gain—a randomised controlled trial.   BJOG. 2015;122(4):537-544. doi:10.1111/1471-0528.13131PubMedGoogle ScholarCrossref 51. Rönö  K, Grotenfelt  NE, Klemetti  MM,  et al.  Effect of a lifestyle intervention during pregnancy—findings from the Finnish gestational diabetes prevention trial (RADIEL).   J Perinatol. 2018;38(9):1157-1164. doi:10.1038/s41372-018-0178-8PubMedGoogle ScholarCrossref 52. Rönö  K, Stach-Lempinen  B, Eriksson  JG,  et al.  Prevention of gestational diabetes with a prepregnancy lifestyle intervention—findings from a randomized controlled trial.   Int J Womens Health. 2018;10:493-501. doi:10.2147/IJWH.S162061PubMedGoogle ScholarCrossref 53. Ruiz  JR, Perales  M, Pelaez  M, Lopez  C, Lucia  A, Barakat  R.  Supervised exercise-based intervention to prevent excessive gestational weight gain: a randomized controlled trial.   Mayo Clin Proc. 2013;88(12):1388-1397. doi:10.1016/j.mayocp.2013.07.020PubMedGoogle ScholarCrossref 54. Sagedal  LR, Øverby  NC, Bere  E,  et al.  Lifestyle intervention to limit gestational weight gain: the Norwegian Fit for Delivery randomised controlled trial.   BJOG. 2017;124(1):97-109. doi:10.1111/1471-0528.13862PubMedGoogle ScholarCrossref 55. Seneviratne  SN, Jiang  Y, Derraik  J,  et al.  Effects of antenatal exercise in overweight and obese pregnant women on maternal and perinatal outcomes: a randomised controlled trial.   BJOG. 2016;123(4):588-597. doi:10.1111/1471-0528.13738PubMedGoogle ScholarCrossref 56. Simmons  D, Devlieger  R, van Assche  A,  et al.  Effect of physical activity and/or healthy eating on GDM risk: the DALI lifestyle study.   J Clin Endocrinol Metab. 2017;102(3):903-913. doi:10.1210/jc.2016-3455PubMedGoogle Scholar 57. Skouteris  H, McPhie  S, Hill  B,  et al.  Health coaching to prevent excessive gestational weight gain: a randomized-controlled trial.   Br J Health Psychol. 2016;21(1):31-51. doi:10.1111/bjhp.12154PubMedGoogle ScholarCrossref 58. Smith  K, Lanningham-Foster  L, Welch  A, Campbell  C.  Web-based behavioral intervention increases maternal exercise but does not prevent excessive gestational weight gain in previously sedentary women.   J Phys Act Health. 2016;13(6):587-593. doi:10.1123/jpah.2015-0219PubMedGoogle ScholarCrossref 59. Thomson  JL, Tussing-Humphreys  LM, Goodman  MH, Olender  SE.  Gestational weight gain: results from the Delta Healthy Sprouts comparative impact trial.   J Pregnancy. 2016;2016:5703607. doi:10.1155/2016/5703607PubMedGoogle Scholar 60. Van Horn  L, Peaceman  A, Kwasny  M,  et al.  Dietary approaches to stop hypertension diet and activity to limit gestational weight: maternal offspring metabolics family intervention trial, a technology enhanced randomized trial.   Am J Prev Med. 2018;55(5):603-614. doi:10.1016/j.amepre.2018.06.015PubMedGoogle ScholarCrossref 61. Vesco  KK, Karanja  N, King  JC,  et al.  Efficacy of a group-based dietary intervention for limiting gestational weight gain among obese women: a randomized trial.   Obesity (Silver Spring). 2014;22(9):1989-1996. doi:10.1002/oby.20831PubMedGoogle ScholarCrossref 62. Vinter  CA, Jensen  DM, Ovesen  P, Beck-Nielsen  H, Jørgensen  JS.  The LiP (Lifestyle in Pregnancy) study: a randomized controlled trial of lifestyle intervention in 360 obese pregnant women.   Diabetes Care. 2011;34(12):2502-2507. doi:10.2337/dc11-1150PubMedGoogle ScholarCrossref 63. Willcox  JC, Wilkinson  SA, Lappas  M,  et al.  A mobile health intervention promoting healthy gestational weight gain for women entering pregnancy at a high body mass index: the txt4two pilot randomised controlled trial.   BJOG. 2017;124(11):1718-1728. doi:10.1111/1471-0528.14552PubMedGoogle ScholarCrossref 64. Wolff  S, Legarth  J, Vangsgaard  K, Toubro  S, Astrup  A.  A randomized trial of the effects of dietary counseling on gestational weight gain and glucose metabolism in obese pregnant women.   Int J Obes (Lond). 2008;32(3):495-501. doi:10.1038/sj.ijo.0803710PubMedGoogle ScholarCrossref |
| Chan et al. 2019 | 1. A¸scı, O.; Rathfisch, G. Effect of lifestyle interventions of pregnant women on their dietary habits, lifestyle behaviors, and weight gain: A randomized controlled trial. J. Health Popul. Nutr. 2016, 35, 7 2. Arizabaleta, A.V.M.; Buitrago, L.O.; de Plata, A.C.A.; Escudero, M.M.; Ramirez-Velez, R. Aerobic exercise during pregnancy improves health-related quality of life: A randomised trial. J. Physiother. 2010, 56, 253–258. 3. Da Silva, S.G.; Hallal, P.C.; Domingues, M.R.; Bertoldi, A.D.; Silveira, M.F.D.; Bassani, D.; da Silva, I.C.M.; da Silva, B.G.C.; Coll, C.V.N.; Evenson, K. A randomized controlled trial of exercise during pregnancy on maternal and neonatal outcomes: Results from the PAMELA study. Int. J. Behav. Nutr. Phys. Act. 2017, 14, 175. 4. Eggen, M.H.; Stuge, B.; Mowinckel, P.; Jensen, K.S.; Hagen, K.B. Can supervised group exercises including ergonomic advice reduce the prevalence and severity of low back pain and pelvic girdle pain in pregnancy? A randomized controlled trial. Phys. Ther. 2012, 92, 781–790 5. Garshasbi, A.; Faghih Zadeh, S. The effect of exercise on the intensity of low back pain in pregnant women. Int. J. Gynaecol. Obstet. 2005, 88, 271–275. 6. Gau, M.L.; Chang, C.Y.; Tian, S.H.; Lin, K.C. Effects of birth ball exercise on pain and self-efficacy during childbirth: A randomised controlled trial in Taiwan. Midwifery 2011, 27, e293–e300 7. Ghodsi, Z.; Asltoghiri, M. Effects of aerobic exercise training on maternal and neonatal outcome: A randomized controlled trial on pregnant women in Iran. J. Pak. Med. Assoc. 2014, 64, 1053–1056 8. Gustafsson, M.K.; Stafne, S.N.; Romundstad, P.R.; Mørkved, S.; Salvesen, K.; Helvik, A.S. The effects of an exercise program during pregnancy on health-related quality of life in pregnant women: A Norwegian randomised controlled trial. BJOG 2016, 123, 1152–1160. 9. Haakstad, L.A.; Bø, K. Effect of a regular exercise program on pelvic girdle and low back pain in previously inactive pregnant women: A randomized controlled trial. J. Rehabil. Med. 2015, 47, 229–234 10. Haakstad, L.A.; Bø, K. Effect of regular exercise on prevention of excessive weight gain in pregnancy: A randomised controlled trial. Eur. J. Contracept. Reprod. Health Care 2011, 16, 116–125 11. Haakstad, L.A.; Torset, B.; Bø, K. What is the effect of regular group exercise on maternal psychological outcomes and common pregnancy complaints? An assessor blinded RCT. Midwifery 2016, 32, 81–86 12. Haakstad, L.A.; Vistad, I.; Sagedal, L.R.; Lohne-Seiler, H.; Torstveit, M.K. How does a lifestyle intervention during pregnancy influence perceived barriers to leisure-time physical activity? The Norwegian fit for delivery study, a randomized controlled trial. BMC Pregnancy Childbirth 2018, 18, 127 13. Huang, T.T.; Yeh, C.Y.; Tsai, Y.C. A diet and physical activity intervention for preventing weight retention among Taiwanese childbearing women: A randomised controlled trial. Midwifery 2011, 27, 257–264 14. Hui, A.; Back, L.; Ludwig, S.; Gardiner, P.; Sevenhuysen, G.; Dean, H.; Sellers, E.; McGavock, J.; Morris, M.; Bruce, S.; et al. Lifestyle intervention on diet and exercise reduced excessive gestational weight gain in pregnant women under a randomised controlled trial. BJOG 2012, 119, 70–77 15. Hui, A.; Ludwig, S.; Gardiner, P.; Sevenhuysen, G.; Murray, R.; Morris, M.; Shen, G.X. Community-based exercise and dietary intervention during pregnancy: A pilot study. Can. J. Diabetes 2006, 30, 1–7 16. Kinnunen, T.I.; Pasanen, M.; Aittasalo, M.; Fogelholm, M.; Hilakivi-Clarke, L.; Weiderpass, E.; Luoto, R. Preventing excessive weight gain during pregnancy—A controlled trial in primary health care. Eur. J. Clin. Nutr. 2007, 61, 884–891. 17. Kluge, J.; Hall, D.; Louw, Q.; Theron, G.; Grové, D. Specific exercises to treat pregnancy-related low back pain in a South African population. Int. J. Gynaecol. Obstet. 2011, 113, 187–191 18. Kokic, I.S.; Ivanisevic, M.; Uremovic, M.; Kokic, T.; Pisot, R.; Simunic, B. Effect of therapeutic exercises on pregnancy-related low back pain and pelvic girdle pain: Secondary analysis of a randomized controlled trial. J. Rehabil. Med. 2017, 49, 251–257 19. Marquez-Sterling, S.; Perry, A.C.; Kaplan, T.A.; Halberstein, R.A.; Signorile, J.F. Physical and psychological changes with vigorous exercise in sedentary primigravidae. Med. Sci. Sports Exerc. 2000, 32, 58–62. 20. Miquelutti, M.A.; Cecatti, J.G.; Makuch, M.Y. Evaluation of a birth preparation program on lumbopelvic pain, urinary incontinence, anxiety and exercise: A randomized controlled trial. BMC Pregnancy Childbirth 2013, 13, 154. 21. Ozdemir, S.; Bebis, H.; Ortabag, T.; Acikel, C. Evaluation of the efficacy of an exercise program for pregnant women with low back and pelvic pain: A prospective randomized controlled trial. J. Adv. Nurs. 2015, 71, 1926–1939 22. Perales, M.; Refoyo, I.; Coteron, J.; Bacchi, M.; Barakat, R. Exercise during pregnancy attenuates prenatal depression: A randomized controlled trial. Eval. Health Prof. 2015, 38, 59–72 23. Robledo-Colonia, A.F.; Sandoval-Restrepo, N.; Mosquera-Valderrama, Y.F.; Escobar-Hurtado, C.; Ramírez-Vélez, R. Aerobic exercise training during pregnancy reduces depressive symptoms in nulliparous women: A randomised trial. J. Physiother. 2012, 58, 9–15. 24. Ronnberg, A.K.; Ostlund, I.; Fadl, H.; Gottvall, T.; Nilsson, K. Intervention during pregnancy to reduce excessive gestational weight gain—A randomised controlled trial. BJOG 2015, 122, 537–544. 25. Ruiz, J.R.; Perales, M.; Pelaez, M.; Lopez, C.; Lucia, A.; Barakat, R. Supervised exercise-based intervention to prevent excessive gestational weight gain: A randomized controlled trial. Mayo Clin. Proc. 2013, 88, 1388–1397 26. Sagedal, L.R.; Øverby, N.C.; Bere, E.; Torstveit, M.K.; Lohne-Seiler, H.; Småstuen, M.; Hillesund, E.R.; Henriksen, T.; Vistad, I. Lifestyle intervention to limit gestational weight gain: The Norwegian Fit for Delivery randomised controlled trial. BJOG 2017, 124, 97–109. 27. Songoygard, K.M.; Stafne, S.N.; Evensen, K.A.; Salvesen, K.A.; Vik, T.; Morkved, S. Does exercise during pregnancy prevent postnatal depression? A randomized controlled trial. Acta Obstet. Gynecol. Scand. 2012, 91, 62–67 28. Stafne, S.N.; Salvesen, K.A.; Romundstad, P.R.; Stuge, B.; Mørkved, S. Does regular exercise during pregnancy influence lumbopelvic pain? A randomized controlled trial. Acta Obstet. Gynecol. Scand. 2012, 91, 552–559 29. Suputtitada, A.; Wacharapreechanont, T.; Chaisayan, P. Effect of the “sitting pelvic tilt exercise” during the third trimester in primigravidas on back pain. J. Med. Assoc. Thail. 2002, 85 (Suppl. 1), S170–S179. |
| Craemer et al.. 2019 | 1. Althuizen E, van der Wijden C L, van Mechelen W, Seidell J C, van Poppel M NM. The effect of a counselling intervention on weight changes during and after pregnancy: a randomised trial. BJOG. 2013;120(01):92– 99. [PubMed] [Google Scholar] 2. Asbee S M, Jenkins T R, Butler J R, White J, Elliot M, Rutledge A.Preventing excessive weight gain during pregnancy through dietary and lifestyle counseling: a randomized controlled trial Obstet Gynecol 2009113(2 Pt 1):305–312. [PubMed] [Google Scholar] 3. Aşcı Ö, Rathfisch G. Effect of lifestyle interventions of pregnant women on their dietary habits, lifestyle behaviors, and weight gain: a randomized controlled trial. J Health Popul Nutr. 2016;35:7. [PMC free article] [PubMed] [Google Scholar] 4. Bogaerts A F, Devlieger R, Nuyts E, Witters I, Gyselaers W, Van den Bergh B RH. Effects of lifestyle intervention in obese pregnant women on gestational weight gain and mental health: a randomized controlled trial. Int J Obes. 2013;37(06):814–821. [PubMed] [Google Scholar] 5. Byrne N M, Groves A M, McIntyre H D, Callaway L K; BAMBINO group.Changes in resting and walking energy expenditure and walking speed during pregnancy in obese women Am J Clin Nutr 20119403819–830. [PubMed] [Google Scholar] 6. Di Carlo C, Iannotti G, Sparice S et al..The role of a personalized dietary intervention in managing gestational weight gain: a prospective, controlled study in a low-risk antenatal population. Arch Gynecol Obstet. 2014;289(04):765–770. [PubMed] [Google Scholar] 7. Garnæs K K, Mørkved S, Salvesen Ø, Moholdt T. Exercise training and weight gain in obese pregnant women: a randomized controlled trial (ETIP trial) PLoS Med. 2016;13(07):e1002079. [PMC free article] [PubMed] [Google Scholar] 8. Haakstad L A, Bø K. Effect of regular exercise on prevention of excessive weight gain in pregnancy: a randomised controlled trial. Eur J Contracept Reprod Health Care. 2011;16(02):116–125. [PubMed] [Google Scholar] 9. Horan M K, McGowan C A, Gibney E R, Byrne J, Donnelly J M, McAuliffe F M. Maternal nutrition and glycaemic index during pregnancy impacts on offspring adiposity at 6 months of age analysis from the ROLO randomised controlled trial. Nutrients. 2016;8(01):13–15. [PMC free article] [PubMed] [Google Scholar] 10. Huang T T, Yeh C Y, Tsai Y C. A diet and physical activity intervention for preventing weight retention among Taiwanese childbearing women: a randomised controlled trial. Midwifery. 2011;27(02):257– 264. [PubMed] [Google Scholar] 11. Hui A L, Back L, Ludwig S et al..Effects of lifestyle intervention on dietary intake, physical activity level, and gestational weight gain in pregnant women with different pre-pregnancy body mass index in a randomized control trial. BMC Pregnancy Childbirth. 2014;14:331. [PMC free article] [PubMed] [Google Scholar] 12. Hui A, Back L, Ludwig S et al..Lifestyle intervention on diet and exercise reduced excessive gestational weight gain in pregnant women under a randomised controlled trial. BJOG. 2012;119(01):70–77. [PubMed] [Google Scholar] 13. Kong K L, Campbell C G, Foster R C, Peterson A D, Lanningham-Foster L. A pilot walking program promotes moderate-intensity physical activity during pregnancy. Med Sci Sports Exerc. 2014;46(03):462– 471. [PubMed] [Google Scholar] 14. Korpi-Hyövälti E A, Laaksonen D E, Schwab U S et al..Feasibility of a lifestyle intervention in early pregnancy to prevent deterioration of glucose tolerance. BMC Public Health. 2011;11:179. [PMC free article] [PubMed] [Google Scholar] 15. Luo X-D, Dong X, Zhou J. Effects of nutritional management intervention on gestational weight gain and perinatal outcome. Saudi Med J. 2014;35(10):1267–1270. [PMC free article] [PubMed] [Google Scholar] 16. Luoto R, Kinnunen T I, Aittasalo M et al..Primary prevention of gestational diabetes mellitus and large-for-gestational-age newborns by lifestyle counseling: a cluster-randomized controlled trial. PLoS Med. 2011;8(05):e1001036. [PMC free article] [PubMed] [Google Scholar] 17. McGowan C A, Walsh J M, Byrne J, Curran S, McAuliffe F M. The influence of a low glycemic index dietary intervention on maternal dietary intake, glycemic index and gestational weight gain during pregnancy: a randomized controlled trial. Nutr J. 2013;12(01):140. [PMC free article] [PubMed] [Google Scholar] 18. Mustila T, Raitanen J, Keskinen P, Saari A, Luoto R. Lifestyle counseling during pregnancy and offspring weight development until four years of age: follow-up study of a controlled trial. J Negat Results Biomed. 2012;11:11. [PMC free article] [PubMed] [Google Scholar] 19. Nascimento S L, Surita F G, Parpinelli MÂ, Siani S, Pinto e Silva J L. The effect of an antenatal physical exercise programme on maternal/perinatal outcomes and quality of life in overweight and obese pregnant women: a randomised clinical trial. BJOG. 2011;118(12):1455–1463. [PubMed] [Google Scholar] 20. Petrella E, Malavolti M, Bertarini V et al..Gestational weight gain in overweight and obese women enrolled in a healthy lifestyle and eating habits program. J Matern Fetal Neonatal Med. 2014;27(13):1348– 1352. [PubMed] [Google Scholar] 21. Phelan S, Phipps M G, Abrams B, Darroch F, Schaffner A, Wing R R. Randomized trial of a behavioral intervention to prevent excessive gestational weight gain: the Fit for Delivery Study. Am J Clin Nutr. 2011;93(04):772– 779. [PMC free article] [PubMed] [Google Scholar] 22. Poston L, Bell R, Croker H et al..Effect of a behavioural intervention in obese pregnant women (the UPBEAT study): a multicentre, randomised controlled trial. Lancet Diabetes Endocrinol. 2015;3(10):767– 777 23. Quinlivan J A, Lam L T, Fisher J. A randomised trial of a four-step multidisciplinary approach to the antenatal care of obese pregnant women. Aust N Z J Obstet Gynaecol. 2011;51(02):141–146. [PubMed] [Google Scholar] 24. Rauh K, Gabriel E, Kerschbaum E et al..Safety and efficacy of a lifestyle intervention for pregnant women to prevent excessive maternal weight gain: a cluster-randomized controlled trial. BMC Pregnancy Childbirth. 2013;13:151. [PMC free article] [PubMed] [Google Scholar] 25. Renault K M, Nørgaard K, Nilas L et al..The Treatment of Obese Pregnant Women (TOP) study: a randomized controlled trial of the effect of physical activity intervention assessed by pedometer with or without dietary intervention in obese pregnant women. Am J Obstet Gyecol. 2014;210(02):1340–1.34E11. [PubMed] [Google Scholar] 26. Ruchat S M, Davenport M H, Giroux I et al..Nutrition and exercise reduce excessive weight gain in normal-weight pregnant women. Med Sci Sports Exerc. 2012;44(08):1419–1426. [PubMed] [Google Scholar] 27. Ruiz J R, Perales M, Pelaez M, Lopez C, Lucia A, Barakat R. Supervised exercise-based intervention to prevent excessive gestational weight gain: a randomized controlled trial. Mayo Clin Proc. 2013;88(12):1388– 1397. [PubMed] [Google Scholar] 28. Szmeja M A, Cramp C, Grivell R M, Deussen A R, Yelland L N, Dodd J M. Use of a DVD to provide dietary and lifestyle information to pregnant women who are overweight or obese: a nested randomised trial. BMC Pregnancy Childbirth. 2014;14:409. [PMC free article] [PubMed] [Google Scholar] 29. Tanvig M, Vinter C A, Jørgensen J S et al..Effects of lifestyle intervention in pregnancy and anthropometrics at birth on offspring metabolic profile at 2.8 years: results from the Lifestyle in Pregnancy and Offspring (LiPO) study. J Clin Endocrinol Metab. 2015;100(01):175–183. [PubMed] [Google Scholar] 30. Vesco K K, Karanja N, King J C et al..Efficacy of a group-based dietary intervention for limiting gestational weight gain among obese women: a randomized trial. Obesity (Silver Spring) 2014;22(09):1989–1996. [PMC free article] [PubMed] [Google Scholar] 31. Walsh J M, McAuliffe F M. Impact of maternal nutrition on pregnancy outcome--does it matter what pregnant women eat? Best Pract Res Clin Obstet Gynaecol. 2015;29(01):63–78. [PubMed] [Google Scholar] |
| Currie et al.. 2013 | 1. Callaway LK, Colditz PB, Byrne NM, Lingwood BE, Rowlands IJ, et al. (2010) Prevention of gestational diabetes feasibility issues for an exercise intervention in obese pregnant women. Diabetes Care 33: 1457–1459. 2. Chasan-Taber L, Silveira M, Marcus BH, Braun B, Stanek E, et al. (2011) Feasibility and efficacy of a physical activity intervention among pregnant women: The behaviors affecting baby and you (BABY) study. Journal of physical activity & health 8: S228. 3. Gaston A, Prapavessis H (2009) Maternal-fetal disease information as a source of exercise motivation during pregnancy. Health Psychology 28: 726. 4. Guelinckx I, Devlieger R, Mullie P, Vansant G (2010) Effect of lifestyle intervention on dietary habits, physical activity, and gestational weight gain in obese pregnant women: A randomized controlled trial. Am J Clin Nutr 91: 373–380. 5. Haakstad LA, Bø K (2011) Effect of regular exercise on prevention of excessive weight gain in pregnancy: A randomised controlled trial. The European Journal of Contraception and Reproductive Health Care 16: 116–125. 6. Huang T, Yeh C, Tsai Y (2011) A diet and physical activity intervention for preventing weight retention among Taiwanese childbearing women: A randomised controlled trial. Midwifery 27: 257–264. 7. Jackson RA, Stotland NE, Caughey AB, Gerbert B (2011) Improving diet and exercise in pregnancy with video doctor counseling: A randomized trial. Patient Educ Couns 83: 203–209. 8. Luoto R, Kinnunen TI, Aittasalo M, Kolu P, Raitanen J, et al. (2011) Primary prevention of gestational diabetes mellitus and large-for-gestational-age newborns by lifestyle counseling: A cluster-randomized controlled trial. PLoS medicine 8: e1001036. 9. Ong M, Guelfi K, Hunter T, Wallman K, Fournier P, et al. (2009) Supervised home-based exercise may attenuate the decline of glucose tolerance in obese pregnant women. Diabetes Metab 35: 418–421. 10. Oostdam N, van Poppel M, Wouters M, Eekhoff E, Bekedam D, et al. (2012) No effect of the FitFor2 exercise programme on blood glucose, insulin sensitivity,and birthweight in pregnant women who were overweight and at risk for gestational diabetes: Results of a randomised controlled trial. BJOG: An International Journal of Obstetrics & Gynaecology: 1098–1107. 11. Polley B, Wing R, Sims C (2002) Randomized controlled trial to prevent excessive weight gain in pregnant women. Int J Obes 26: 1494–1502. 12. Rankin J (2002) Effects of antenatal exercise on psychological well-being.pregnancy and birth outcome. 13. Shen GX (2006) Community-based exercise and dietary intervention during pregnancy: A pilot study. Canadian Journal of diabetes 30: 169–175. 14. Yeo S (2009) Adherence to walking or stretching, and risk of preeclampsia in sedentary pregnant women. Res Nurs Health 32: 379–390. |
| Davenport et al. 2018a | 1. Battle CL, Uebelacker LA, Magee SR, et al. Potential for prenatal yoga to serve as an intervention to treat depression during pregnancy. Womens Health Issues 2015;25:134–41. 2. Beddoe AE, Paul Yang CP, Kennedy HP, et al. The effects of mindfulness-based yoga during pregnancy on maternal psychological and physical distress. J Obstet Gynecol Neonatal Nurs 2009;38:310–9. 3. Bershadsky S, Trumpfheller L, Kimble HB, et al. The effect of prenatal Hatha yoga on affect, cortisol and depressive symptoms. Complement Ther Clin Pract 2014;20:106–13. 4. Bowen A, Stewart N, Baetz M, et al. Antenatal depression in socially high-risk women in Canada. J Epidemiol Community Health 2009;63:414–6 5. Claesson IM, Klein S, Sydsjö G, et al. Physical activity and psychological well-being in obese pregnant and postpartum women attending a weight-gain restriction programme. Midwifery 2014;30:11–16. 6. Da Costa D, Rippen N, Dritsa M, et al. Self-reported leisure-time physical activity during pregnancy and relationship to psychological well-being. J Psychosom Obstet Gynaecol 2003;24:111–9. 7. de Wit L, Jelsma JG, van Poppel MN, et al. Physical activity, depressed mood and pregnancy worries in European obese pregnant women: results from the DALI study. BMC Pregnancy Childbirth 2015;15:158. 8. Dodd JM, Newman A, Moran LJ, et al. The effect of antenatal dietary and lifestyle advice for women who are overweight or obese on emotional well-being: the LIMIT randomized trial. Acta Obstet Gynecol Scand 2016;95:309–18 9. Downs DS, DiNallo JM, Kirner TL. Determinants of pregnancy and postpartum depression: prospective influences of depressive symptoms, body image satisfaction, and exercise behavior. Ann Behav Med 2008;36:54–63. 10. El-Rafie MM, Khafagy GM, Gamal MG. Effect of aerobic exercise during pregnancy on antenatal depression. Int J Womens Health 2016;8:53–7. 11. Ersek JL, Brunner Huber LR. Physical activity prior to and during pregnancy and risk of postpartum depressive symptoms. J Obstet Gynecol Neonatal Nurs 2009;38:556–66. 12. Field T, Diego M, Delgado J, et al. Yoga and social support reduce prenatal depression, anxiety and cortisol. J Bodyw Mov Ther 2013;17:397–403. 13. Field T. Postpartum depression effects on early interactions, parenting, and safety practices: a review. Infant Behav Dev 2010;33:1–6. 14. Gaston A, Prapavessis H, Tired PH. Tired, moody and pregnant? Exercise may be the answer. Psychol Health 2013;28:1353–69 15. Goodwin A, Astbury J, McMeeken J. Body image and psychological well-being in pregnancy. A comparison of exercisers and non-exercisers. Aust N Z J Obstet Gynaecol 2000;40:442–7 16. Guszkowska M, Langwald M, Sempolska K. Does physical exercise help maintain mental health during pregnancy? A comparison of changes in mental health in participants of physical exercise classes and childbirth classes. J Phys Act Health 2015;12:30–6. 17. Kalayeh S, Nikbakht R, Seddigi S. The effect of exercise during pregnancy on mother’s depression in zahedan. International Journal of Pharmacy and Technology 2016;8:12248–62. 18. Loprinzi PD, Cardinal BJ, Loprinzi KL, et al. Benefits and environmental determinants of physical activity in children and adolescents. Obes Facts 2012;5:597–610. 19. Miquelutti MA, Cecatti JG, Makuch MY. Evaluation of a birth preparation program on lumbopelvic pain, urinary incontinence, anxiety and exercise: a randomized controlled trial. BMC Pregnancy Childbirth 2013;13:154 20. Newham JJ, Wittkowski A, Hurley J, et al. Effects of antenatal yoga on maternal anxiety and depression: a randomized controlled trial. Depress Anxiety 2014;31:631–40. 21. Petrovic D, Perovic M, Lazovic B, et al. Association between walking, dysphoric mood and anxiety in late pregnancy: A cross-sectional study. Psychiatry Res 2016;246:360–3. 22. Strøm M, Mortensen EL, Halldorson TI, et al. Leisure-time physical activity in pregnancy and risk of postpartum depression: a prospective study in a large national birth cohort. J Clin Psychiatry 2009;70:1707–14 23. Ussher M, Lewis S, Aveyard P, et al. Physical activity for smoking cessation in pregnancy: randomised controlled trial. BMJ 2015;350:h2145 |
| Davenport et al. 2018b | 1. Asbee SM, Jenkins TR, Butler JR, et al. Preventing excessive weight gain during pregnancy through dietary and lifestyle Counseling. Obstetrics & Gynecology 2009;113(2, Part 1):305–12. 2. Avery MD, Leon AS, Kopher RA. Effects of a partially home-based exercise program for women with gestational diabetes. Obstet Gynecol 1997;89:10–15. 3. Badon SE, Wander PL, Qiu C, et al. Maternal leisure time physical activity and infant birth size. Epidemiology 2016;27:74–81. 4. Badon SE, Wartko PD, Qiu C, et al. Leisure time physical activity and gestational diabetes mellitus in the omega study. Medicine & Science in Sports & Exercise 2016;48:1044–52. 5. Barakat R, Cordero Y, Coteron J, et al. Exercise during pregnancy improves maternal glucose screen at 24–28 weeks: a randomised controlled trial. Br J Sports Med 2012;46:656–61. 6. Barakat R, Pelaez M, Cordero Y, et al. Exercise during pregnancy protects against hypertension and macrosomia: randomized clinical trial. Am J Obstet Gynecol 2016;214:649.e1–8. 7. Barakat R, Pelaez M, Lopez C, et al. Exercise during pregnancy and gestational diabetes-related adverse effects: a randomised controlled trial. Br J Sports Med 2013;47:630–6. 8. Barakat R, Pelaez M, Lopez C, et al. Exercise during pregnancy and gestational diabetes-related adverse effects: a randomised controlled trial. Br J Sports Med 2013;47:630–6. 9. Barakat R, Pelaez M, Montejo R, et al. Exercise during pregnancy improves maternal health perception: a randomized controlled trial. Am J Obstet Gynecol 2011;204:402.e1–7. 10. Barakat R, Perales M, Bacchi M, et al. A program of exercise throughout pregnancy. is it safe to mother and newborn? American Journal of Health Promotion 2014;29:2–8. 11. Barakat R, Ruiz JR, Stirling JR, et al. Type of delivery is not affected by light resistance and toning exercise training during pregnancy: a randomized controlled trial. Am J Obstet Gynecol 2009;201:590.e1–6. 12. Bell RJ, Palma SM, Lumley JM. The effect of vigorous exercise during pregnancy on birth-weight. The Australian and New Zealand Journal of Obstetrics and Gynaecology 1995;35:46–51. 13. Bisson M, Alméras N, Dufresne SS, et al. A 12-Week Exercise Program for Pregnant Women with Obesity to Improve Physical Activity Levels: An Open Randomised Preliminary Study. PLoS One 2015;10:e0137742. 14. Callaway LK, Colditz PB, Byrne NM, et al. Prevention of gestational diabetes: feasibility issues for an exercise intervention in obese pregnant women. Diabetes Care 2010;33:1457–9. 15. Chasan-Taber L, Schmidt MD, Pekow P, et al. Physical Activity and Gestational Diabetes Mellitus among Hispanic Women. J Womens Health 2008;17:999–1008. 16. Chasan-Taber L, Silveira M, Pekow P, et al. Physical activity, sedentary behavior and risk of hypertensive disorders of pregnancy in Hispanic women. Hypertens Pregnancy 2015;34:1–16. 17. Cordero Y, Mottola MF, Vargas J, et al. Exercise is associated with a reduction in gestational diabetes mellitus. Medicine & Science in Sports & Exercise 2015;47:1328–33. 18. Cordero Y, Peláez M, De Miguel M, et al. Can moderate physical exercise during pregnancy act as a factor in preventing Gestational Diabetes? Revista Internacional de Ciencias del Deporte 2012;27:3–19. 19. Currie LM, Woolcott CG, Fell DB, et al. The association between physical activity and maternal and neonatal outcomes: a prospective cohort. Matern Child Health J 2014;18:1823–30. 20. Cw K, Napolitano PG, Lee SP, et al. Physical activity, maternal metabolic measures, and the incidence of gallbladder sludge or stones during pregnancy: a randomized trial. Am J Perinatol 2014;31:39–48. 21. Dale E, Mullinax KM, Bryan DH. Exercise during pregnancy: effects on the fetus. Can J Appl Sport Sci 1982;7:98–103. 22. de Oliveria Melo AS, Silva JL, Tavares JS, et al. Effect of a physical exercise program during pregnancy on uteroplacental and fetal blood flow and fetal growth: a randomized controlled trial. Obstet Gynecol 2012;120(2 Pt 1):302–10. 23. Dempsey JC, Butler CL, Sorensen TK, et al. A case-control study of maternal recreational physical activity and risk of gestational diabetes mellitus. Diabetes Res Clin Pract 2004;66:203–15. 24. Dodd JM, Newman A, Moran LJ, et al. The effect of antenatal dietary and lifestyle advice for women who are overweight or obese on emotional well-being: the LIMIT randomized trial. Acta Obstet Gynecol Scand 2016;95:309–18. 25. Dyck RF, Sheppard SM, Klomp H, et al. Using exercise to prevent gestational diabetes among aboriginal women - hypothesis and results of a pilot/feasibility project in Saskatchewan. Canadian Journal of Diabetes Care 1999;23:32–8. 26. Elden H, Ostgaard H-C, Fagevik-Olsen M, et al. Treatments of pelvic girdle pain in pregnant women: adverse effects of standard treatment, acupuncture and stabilising exercises on the pregnancy, mother, delivery and the fetus/neonate. BMC Complement Altern Med 2008;8:34. 27. Ferland S, Bujold E, Giguère Y, et al. Association between physical activity in early pregnancy and markers of placental growth and function. J Obstet Gynaecol Can 2013;35:787–92. 28. Garnæs KK, Mørkved S, Salvesen Ø, et al. Exercise training and weight gain in obese pregnant women: a randomized controlled trial (ETIP Trial). PLoS Med 2016;13:e1002079 29. Guelfi KJ, Ong MJ, Crisp NA, et al. Regular exercise to prevent the recurrence of gestational diabetes mellitus: a randomized controlled trial. Obstet Gynecol 2016;128:819–27. 30. Guelinckx I, Devlieger R, Mullie P, et al. Effect of lifestyle intervention on dietary habits, physical activity, and gestational weight gain in obese pregnant women: a randomized controlled trial. Am J Clin Nutr 2010;91:373–80. 31. Haakstad LA, Bo K. Effect of regular exercise on prevention of excessive weight gain in pregnancy: a randomised controlled trial. Eur J Contracept Reprod Health Care 2011;16:116–25. 32. Harizopoulou VC, Kritikos A, Papanikolaou Z, et al. Maternal physical activity before and during early pregnancy as a risk factor for gestational diabetes mellitus. Acta Diabetol 2010;47(S1):83–9. 33. Harris ST, Liu J, Wilcox S, et al. Exercise during pregnancy and its association with gestational weight gain. Matern Child Health J 2015;19:528–37. 34. Harrison CL, Lombard CB, Strauss BJ, et al. Optimizing healthy gestational weight gain in women at high risk of gestational diabetes: A randomized controlled trial. Obesity 2013;21:904–9. 35. Hatch MC, Shu X-O, McLean DE, et al. Maternal exercise during pregnancy, physical fitness, and fetal growth. Am J Epidemiol 1993;137:1105–14. 36. Hollingsworth DR, Moore TR. Postprandial walking exercise in pregnant insulindependent (type I) diabetic women: reduction of plasma lipid levels but absence of a significant effect on glycemic control. Am J Obstet Gynecol 1987;157:1359–63. 37. Hopkins SA, Baldi JC, Cutfield WS, et al. Exercise training in pregnancy reduces offspring size without changes in maternal insulin sensitivity. J Clin Endocrinol Metab 2010;95:2080–8. 38. Hui A, Back L, Ludwig S, et al. Lifestyle intervention on diet and exercise reduced excessive gestational weight gain in pregnant women under a randomised controlled trial. BJOG 2012;119:70–7. 39. Hui AL, Back L, Ludwig S, et al. Effects of lifestyle intervention on dietary intake, physical activity level, and gestational weight gain in pregnant women with different pre-pregnancy Body Mass Index in a randomized control trial. BMC Pregnancy Childbirth 2014;14:331. 40. Hui AL, Ludwig S, Gardiner P, et al. Community-based exercise and dietary intervention during pregnancy:a pilot study. Can J Diabetes 2006;30:1–7. 41. Iqbal R, Rafique G, Badruddin S, et al. Increased body fat percentage and physical inactivity are independent predictors of gestational diabetes mellitus in South Asian women. Eur J Clin Nutr 2007;61:736–42. 42. Jing W, Huang Y, Liu X, et al. The effect of a personalized intervention on weight gain and physical activity among pregnant women in China. International Journal of Gynecology & Obstetrics 2015;129:138–41. 43. Juhl M, Kogevinas M, Andersen PK, et al. Is swimming during pregnancy a safe exercise? Epidemiology 2010;21:253–8. 44. Kasawara KT, Burgos CS, do Nascimento SL, et al. Maternal and Perinatal Outcomes of Exercise in Pregnant Women with Chronic Hypertension and/ or Previous Preeclampsia: A Randomized Controlled Trial. ISRN Obstet Gynecol 2013;2013:1–8. 45. Koivusalo SB, Rönö K, Klemetti MM, et al. Gestational diabetes mellitus can be prevented by lifestyle intervention: the finnish gestational diabetes prevention study (RADIEL). Diabetes Care 2016;39:24–30. 46. KONG KAIL, Campbell CG, Foster RC, et al. A pilot walking program promotes moderate-intensity physical activity during pregnancy. Medicine & Science in Sports & Exercise 2014;46:462–71. 47. Kordi R, Abolhasani M, Rostami M, et al. Comparison between the effect of lumbopelvic belt and home based pelvic stabilizing exercise on pregnant women with pelvic girdle pain; a randomized controlled trial. J Back Musculoskelet Rehabil 2013;26:133–9. 48. Korpi-Hyövälti E, Heinonen S, Schwab U, et al. Effect of intensive counselling on physical activity in pregnant women at high risk for gestational diabetes mellitus. A clinical study in primary care. Prim Care Diabetes 2012;6:261–8 49. Leng J, Liu G, Zhang C, et al. Physical activity, sedentary behaviors and risk of gestational diabetes mellitus: a population-based cross-sectional study in Tianjin, China. Eur J Endocrinol 2016;174:763–73. 50. Li Q, Xiong R, Wang L, et al. Associations of dietary habits, physical activity and cognitive views with gestational diabetes mellitus among Chinese women. Public Health Nutr 2014;17:1850–7. 51. Lindqvist M, Lindkvist M, Eurenius E, et al. Leisure time physical activity among pregnant women and its associations with maternal characteristics and pregnancy outcomes. Sexual & Reproductive Healthcare 2016;9:14–20. 52. Liu J, Wilcox S, Whitaker K, et al. Preventing excessive weight gain during pregnancy and promoting postpartum weight loss: a pilot lifestyle intervention for overweight and obese African American Women. Matern Child Health J 2015;19:840–9. 53. Lombardi W, Wilson S, Peniston PB. Wellness intervention with pregnant soldiers. Mil Med 1999;164:22–9. 54. Luoto R, Kinnunen TI, Aittasalo M, et al. Primary prevention of gestational diabetes mellitus and large-for-gestational-age newborns by lifestyle counseling: a clusterrandomized controlled trial. PLoS Med 2011;8:e1001036. 55. Magann EF, Evans SF, Weitz B, et al. intrapartum, and neonatal significance of exercise on healthy low-risk pregnant working women. Obstet Gynecol 2002;99:466–72. 56. Martin CL, Brunner Huber LR. Physical activity and hypertensive complications during pregnancy: findings from 2004 to 2006 North Carolina Pregnancy Risk Assessment Monitoring System. Birth 2010;37:202–10 57. McAuley SE, Jensen D, McGrath MJ, et al. Effects of human pregnancy and aerobic conditioning on alveolar gas exchange during exercise. Can J Physiol Pharmacol 2005;83:625–33. 58. McGiveron A, Foster S, Pearce J, et al. Limiting antenatal weight gain improves maternal health outcomes in severely obese pregnant women: findings of a pragmatic evaluation of a midwife-led intervention. Journal of Human Nutrition and Dietetics 2015;28(Suppl 1):29–37. 59. Momeni Javid F, Simbar M, Dolatian M, et al. Comparison of lifestyles of women with gestational diabetes and healthy pregnant women. Glob J Health Sci 2015;7:162–9. 60. Mørkrid K, Jenum AK, Berntsen S, et al. Objectively recorded physical activity and the association with gestational diabetes. Scand J Med Sci Sports 2014;24:e389–97. 61. Murtezani A, Paçarada M, Ibraimi Z, et al. The impact of exercise during pregnancy on neonatal outcomes: a randomized controlled trial. J Sports Med Phys Fitness 2014;54:802–8. 62. Mustila T, Raitanen J, Keskinen P, et al. Pragmatic controlled trial to prevent childhood obesity in maternity and child health care clinics: pregnancy and infant weight outcomes (The VACOPP Study). BMC Pediatr 2013;13:80. 63. Narendran S, Nagarathna R, Narendran V, et al. Efficacy of yoga on pregnancy outcome. J Altern Complement Med 2005;11:237–44. 64. Nasiri-Amiri F, Bakhtiari A, Faramarzi M, et al. The Association Between Physical Activity During Pregnancy and Gestational Diabetes Mellitus: A Case-Control Study. Int J Endocrinol Metab 2016;14:e37123. 65. Nobles C, Marcus BH, Stanek EJ, et al. Effect of an exercise intervention on gestational diabetes mellitus: a randomized controlled trial. Obstet Gynecol 2015;125:1195–204. 66. O’Connor PJ, Poudevigne MS, Cress ME, et al. Safety and efficacy of supervised strength training adopted in pregnancy. J Phys Act Health 2011;8:309–20. 67. Oken E, Ning Y, Rifas-Shiman SL, et al. Associations of physical activity and inactivity before and during pregnancy with glucose tolerance. Obstetrics & Gynecology 2006;108:1200–7. 68. Okido MM, Valeri FL, Martins WP, et al. Assessment of foetal wellbeing in pregnant women subjected to pelvic floor muscle training: a controlled randomised study. Int Urogynecol J 2015;26:1475–81 69. Oostdam N, van Poppel MNM, Wouters M, et al. No effect of the FitFor2 exercise programme on blood glucose, insulin sensitivity, and birthweight in pregnant women who were overweight and at risk for gestational diabetes: results of a randomised controlled trial. BJOG 2012;119:1098–107. 70. Pelaez M, Gonzalez-Cerron S, Montejo R, et al. Pelvic floor muscle training included in a pregnancy exercise program is effective in primary prevention of urinary incontinence: a randomized controlled trial. Neurourol Urodyn 2014;33:67–71. 71. Perales M, Mateos S, Vargas M, et al. Fetal and maternal heart rate responses to exercise in pregnant women. A randomized Controlled Trial. Archivos de medicina del deporte 2015;32:361–7. 72. Petrella E, Malavolti M, Bertarini V, et al. Gestational weight gain in overweight and obese women enrolled in a healthy lifestyle and eating habits program. The Journal of Maternal-Fetal & Neonatal Medicine 2014;27:1348–52. 73. Phelan S, Phipps MG, Abrams B, et al. Randomized trial of a behavioral intervention to prevent excessive gestational weight gain: the Fit for Delivery Study. Am J Clin Nutr 2011;93:772–9. 74. Piravej K, Saksirinukul R. Survey of patterns, attitudes, and the general effects of exercise during pregnancy in 203 Thai pregnant women at King Chulalongkorn Memorial Hospital. J Med Assoc Thai 2001;84(Suppl 1):S276–82. 75. Polley BA, Wing RR, Sims CJ. Randomized controlled trial to prevent excessive weight gain in pregnant women. Int J Obes 2002;26:1494–502. 76. Poston L, Bell R, Croker H, et al. Effect of a behavioural intervention in obese pregnant women (the UPBEAT study): a multicentre, randomised controlled trial. Lancet Diabetes Endocrinol 2015;3:767–77. 77. Price BB, Amini SB, Kappeler K. Exercise in pregnancy: effect on fitness and obstetric outcomes-a randomized trial. Med Sci Sports Exerc 2012;44:2263–9. 78. Putnam KF, Mueller LA, Magann EF, et al. Evaluating effects of self-reported domestic physical activity on pregnancy and neonatal outcomes in “stay at home” military wives. Mil Med 2013;178:893–8. 79. Rauh K, Gabriel E, Kerschbaum E, et al. Safety and efficacy of a lifestyle intervention for pregnant women to prevent excessive maternal weight gain: a clusterrandomized controlled trial. BMC Pregnancy Childbirth 2013;13:151. 80. Renault KM, Nørgaard K, Nilas L, et al. The Treatment of Obese Pregnant Women (TOP) study: a randomized controlled trial of the effect of physical activity intervention assessed by pedometer with or without dietary intervention in obese pregnant women. Am J Obstet Gynecol 2014;210:134.e1–9. 81. Ruiz JR, Perales M, Pelaez M, et al. Supervised exercise–based intervention to prevent excessive gestational weight gain: a randomized controlled trial. Mayo Clin Proc 2013;88:1388–97. 82. Saftlas AF, Logsden-Sackett N, Wang W, et al. Work, leisure-time physical activity, and risk of preeclampsia and gestational hypertension. Am J Epidemiol 2004;160:758–65. 83. Sagedal LR, Øverby NC, Bere E, et al. Lifestyle intervention to limit gestational weight gain: the Norwegian Fit for Delivery randomised controlled trial. BJOG 2017;124:97–109. 84. Seneviratne SN, Jiang Y, Derraik JGB, et al. Effects of antenatal exercise in overweight and obese pregnant women on maternal and perinatal outcomes: a randomised controlled trial. BJOG 2016;123:588–97. 85. Shirazian T, Monteith S, Friedman F, et al. Lifestyle Modification Program Decreases Pregnancy Weight Gain in Obese Women. Am J Perinatol 2010;27:411–4. 86. Simmons D, Devlieger R, van Assche A, et al. Effect of physical activity and/or healthy eating on GDM risk: the DALI Lifestyle Study. J Clin Endocrinol Metab 2017;102:903–13. 87. Simmons D, Jelsma JGM, Galjaard S, et al. Results from a European multicenter randomized trial of physical activity and/or healthy eating to reduce the risk of gestational diabetes mellitus: the DALI lifestyle pilot. Diabetes Care 2015;38:1650–6. 88. Smith K, Lanningham-Foster L, Welch A, et al. Web-Based Behavioral Intervention Increases Maternal Exercise but Does Not Prevent Excessive Gestational Weight Gain in Previously Sedentary Women. Journal of Physical Activity and Health 2016;13:587–93. 89. Stafne SN, Salvesen KA, Romundstad PR, et al. Regular exercise during pregnancy to prevent gestational diabetes: a randomized controlled trial. Obstet Gynecol 2012;119:29–36. 90. Sun Y, Zhao H. The effectiveness of lifestyle intervention in early pregnancy to prevent gestational diabetes mellitus in Chinese overweight and obese women: A quasi-experimental study. Applied Nursing Research 2016;30:125–30. 91. Taniguchi C, Sato C. Home-based walking during pregnancy affects mood and birth outcomes among sedentary women: A randomized controlled trial. Int J Nurs Pract 2016;22:420–6. 92. Tomić V, Sporiš G, Tomić J, et al. The effect of maternal exercise during pregnancy on abnormal fetal growth. Croat Med J 2013;54:362–8. 93. Ussher M, Lewis S, Aveyard P, et al. The London Exercise And Pregnant smokers (LEAP) trial: a randomised controlled trial of physical activity for smoking cessation in pregnancy with an economic evaluation. Health Technol Assess 2015;19:1–135. 94. Vesco KK, Karanja N, King JC, et al. Efficacy of a group-based dietary intervention for limiting gestational weight gain among obese women: A randomized trial. Obesity 2014;22:1989–96. 95. Vinter CA, Jensen DM, Ovesen P, et al. The LiP (Lifestyle in Pregnancy) study: a randomized controlled trial of lifestyle intervention in 360 obese pregnant women. Diabetes Care 2011;34:2502–7. 96. Vollebregt KC, Wolf H, Boer K, et al. Does physical activity in leisure time early in pregnancy reduce the incidence of preeclampsia or gestational hypertension? Acta Obstet Gynecol Scand 2010;89:261–7. 97. Wang C, Wei Y, Zhang X, et al. Effect of regular exercise commenced in early pregnancy on the incidence of gestational diabetes mellitus in overweight and obese pregnant women: a randomized controlled trial. Diabetes Care 2016;39:e163–4. 98. Weissgerber TL, Davies GA, Roberts JM. Modification of angiogenic factors by regular and acute exercise during pregnancy. J Appl Physiol 2010;108:1217–23. 99. White E, Pivarnik J, Pfeiffer K. Resistance training during pregnancy and perinatal outcomes. Journal of Physical Activity and Health 2014;11:1141–8. 100. Ww T, Wong MW. Bone mineral density changes during pregnancy in actively exercising women as measured by quantitative ultrasound. Arch Gynecol Obstet 2012;286:357–63. 101. Yeo S, Davidge S, Ronis DL, et al. A comparison of walking versus stretching exercises to reduce the incidence of preeclampsia: a randomized clinical trial. |
| Davenport et al. 2018c | 1. Abirami P, Judie A. Reduction of risk on newly detected gestational diabetes mellitus by multi model intervention-A hospital based study. International Journal of Pharmaceutical and Clinical Research 2014;6:370–4. 2. Artal R, Catanzaro RB, Gavard JA, et al. A lifestyle intervention of weight-gain restriction: diet and exercise in obese women with gestational diabetes mellitus. Appl Physiol Nutr Metab 2007;32:596–601. 3. Artal R, Platt LD, Sperling M, et al. I. Maternal cardiovascular and metabolic responses in normal pregnancy. Am J Obstet Gynecol 1981;140:123–7. 4. Artal R, Rutherford S, Romem Y, et al. Fetal heart rate responses to maternal exercise. Am J Obstet Gynecol 1986;155:729–33. 5. Artal R, Wiswell R, Romem Y. Hormonal responses to exercise in diabetic and nondiabetic pregnant patients. Diabetes 1985;34 Suppl 2(Supplement_2):78–80. 6. Avery MD, Leon AS, Kopher RA. Effects of a partially home-based exercise program for women with gestational diabetes. Obstet Gynecol 1997;89:10–15. 7. Avery MD, Walker AJ. Acute effect of exercise on blood glucose and insulin levels in women with gestational diabetes. J Matern Fetal Med 2001;10:52–8. 8. Bessinger RC, McMurray RG, Hackney AC. Substrate utilization and hormonal responses to moderate intensity exercise during pregnancy and after delivery. Am J Obstet Gynecol 2002;186:757–64. 9. Bo S, Rosato R, Ciccone G, et al. Simple lifestyle recommendations and the outcomes of gestational diabetes. A 2×2 factorial randomized trial. Diabetes, Obesity and Metabolism 2014;16:1032–5. 10. Bonen A, Campagna P, Gilchrist L, et al. Substrate and endocrine responses during exercise at selected stages of pregnancy. J Appl Physiol 1992;73:134–42. 11. Brankston GN, Mitchell BF, Ryan EA, et al. Resistance exercise decreases the need for insulin in overweight women with gestational diabetes mellitus. Am J Obstet Gynecol 2004;190:188–93. 12. Bung P, Artal R, Khodiguian N, et al. Exercise in gestational diabetes. An optional therapeutic approach? Diabetes 1991;40 Suppl 2(Supplement_2):182–5. 13. Callaway LK, Colditz PB, Byrne NM, et al. Prevention of gestational diabetes: feasibility issues for an exercise intervention in obese pregnant women. Diabetes Care 2010;33:1457–9. 14. Clapp JF, Capeless EL. The changing glycemic response to exercise during pregnancy. Am J Obstet Gynecol 1991;165:1678–83. 15. Clapp JF, Wesley M, Sleamaker RH. Thermoregulatory and metabolic responses to jogging prior to and during pregnancy. Med Sci Sports Exerc 1987;19:124???130–30. 16. Clapp JF. Effect of dietary carbohydrate on the glucose and insulin response to mixed caloric intake and exercise in both nonpregnant and pregnant women. Diabetes Care 1998;21 Suppl 2(Suppl 2):107. 17. Cordero Y, Mottola MF, Vargas J, et al. Exercise Is Associated with a Reduction in Gestational Diabetes Mellitus. Med Sci Sports Exerc 2015;47:1328–33. 18. Cowett RM, Carpenter MW, Carr S, et al. Glucose and lactate kinetics during a short exercise bout in pregnancy. Metabolism 1996;45:753–8. 19. Daniel J, Dikki C. Aerobic dance exercise improves blood glucose level in pregnant women with gestational diabetes mellitus: sport science. African Journal for Physical Health Education, Recreation and Dance. 2014:273–81. 20. Davenport MH, Mottola MF, McManus R, et al. A walking intervention improves capillary glucose control in women with gestational diabetes mellitus: a pilot study. Appl Physiol Nutr Metab 2008;33:511–7. 21. de Barros MC, Lopes MA, Francisco RP, et al. Resistance exercise and glycemic control in women with gestational diabetes mellitus. Am J Obstet Gynecol 2010;203:556. e1–556.e6. 22. Ferrara A, Hedderson MM, Albright CL, et al. A pregnancy and postpartum lifestyle intervention in women with gestational diabetes mellitus reduces diabetes risk factors: a feasibility randomized control trial. Diabetes Care 2011;34:1519–25. 23. Garnæs KK, Mørkved S, Salvesen Ø, et al. Exercise Training and Weight Gain in Obese Pregnant Women: A Randomized Controlled Trial (ETIP Trial). PLoS Med 2016;13:e1002079. 24. Giroux I, Inglis SD, Lander S, et al. Dietary intake, weight gain, and birth outcomes of physically active pregnant women: a pilot study. Appl Physiol Nutr Metab 2006;31:483–9. 25. Guelfi KJ, Ong MJ, Crisp NA, et al. Regular Exercise to Prevent the Recurrence of Gestational Diabetes Mellitus: A Randomized Controlled Trial. Obstet Gynecol 2016;128:819–27. 26. Halse RE, Wallman KE, Newnham JP, et al. Home-based exercise training improves capillary glucose profile in women with gestational diabetes. Med Sci Sports Exerc 2014;46:1702–9. 27. Halse RE, Wallman KE, Newnham JP, et al. Pregnant women exercise at a higher intensity during 30 min of self-paced cycling compared with walking during late gestation: implications for 2 h postprandial glucose levels. Metabolism 2013;62:801–7. 28. Hanan Sayed EM, Adly Ali S, Mohamed Mostafa R AP, et al. Effect of Antenatal Exercises on Umbilical Blood Flow and Neonate Wellbeing in Diabetic Pregnant Women. Indian Journal of Physiotherapy & Occupational Therapy 2012;6:121–5. 29. Hauth JC, Gilstrap LC, Widmer K. Fetal heart rate reactivity before and after maternal jogging during the third trimester. Am J Obstet Gynecol 1982;142:545–7. 30. Hawkins M, Hosker M, Marcus BH, et al. A pregnancy lifestyle intervention to prevent gestational diabetes risk factors in overweight Hispanic women: a feasibility randomized controlled trial. Diabet Med 2015;32:108–15. 31. Jovanovic L, Kessler A, Peterson CM. Human maternal and fetal response to graded exercise. J Appl Physiol 1985;58:1719–22. 32. Jovanovic-Peterson L, Durak EP, Peterson CM. Randomized trial of diet versus diet plus cardiovascular conditioning on glucose levels in gestational diabetes. Am J Obstet Gynecol 1989;161:415–9. 33. Ko CW, Napolitano PG, Lee SP, et al. Physical activity, maternal metabolic measures, and the incidence of gallbladder sludge or stones during pregnancy: a randomized trial. Am J Perinatol 2014;31:39–48. 34. Koivusalo SB, Rönö K, Klemetti MM, et al. Gestational Diabetes Mellitus Can Be Prevented by Lifestyle Intervention: The Finnish Gestational Diabetes Prevention Study (RADIEL): A Randomized Controlled Trial. Diabetes Care 2016;39:24–30. 35. Lesser KB, Gruppuso PA, Terry RB, et al. Exercise fails to improve postprandial glycemic excursion in women with gestational diabetes. J Matern Fetal Med 1996;5:211–7. 36. Lotgering FK, Spinnewijn WE, Struijk PC, et al. Respiratory and metabolic responses to endurance cycle exercise in pregnant and postpartum women. Int J Sports Med 1998;19:193–8. 37. Luoto R, Kinnunen TI, Aittasalo M, et al. Primary prevention of gestational diabetes mellitus and large-for-gestational-age newborns by lifestyle counseling: a clusterrandomized controlled trial. PLoS Med 2011;8:e1001036. 38. McMURRAY RG, Hackney AC, Guion WK, et al. Metabolic and hormonal responses to low-impact aerobic dance during pregnancy. Medicine & Science in Sports & Exercise 1996;28:41–6. 39. Mottola MF, Inglis S, Brun CR, et al. Physiological and metabolic responses of late pregnant women to 40 min of steady-state exercise followed by an oral glucose tolerance perturbation. J Appl Physiol 2013;115:597–604. 40. Ong MJ, Guelfi KJ, Hunter T, et al. Supervised home-based exercise may attenuate the decline of glucose tolerance in obese pregnant women. Diabetes Metab 2009;35:418–21. 41. Oostdam N, van Poppel MN, Wouters MG, et al. No effect of the FitFor2 exercise programme on blood glucose, insulin sensitivity, and birthweight in pregnant women who were overweight and at risk for gestational diabetes: results of a randomised controlled trial. BJOG 2012;119:1098–107. 42. Poston L, Bell R, Croker H, et al. Effect of a behavioural intervention in obese pregnant women (the UPBEAT study): a multicentre, randomised controlled trial. Lancet Diabetes Endocrinol 2015;3:767–77. 43. Ruchat SM, Davenport MH, Giroux I, et al. Effect of exercise intensity and duration on capillary glucose responses in pregnant women at low and high risk for gestational diabetes. Diabetes Metab Res Rev 2012;28:669–78. 44. Sagedal LR, Øverby NC, Bere E, et al. Lifestyle intervention to limit gestational weight gain: the Norwegian Fit for Delivery randomised controlled trial. BJOG 2017;124:97–109. 45. Simmons D, Devlieger R, van Assche A, et al. Effect of physical activity and/or healthy eating on GDM risk: The DALI Lifestyle Study. The Journal of Clinical Endocrinology & Metabolism 2016;102:jc.2016-3455–13. 46. Simmons D, Jelsma JG, Galjaard S, et al. Results from a european multicenter randomized trial of physical activity and/or healthy eating to reduce the risk of gestational diabetes mellitus: The dali lifestyle pilot. Diabetes Care 2015;38:1650–6. 47. Soultanakis HN, Artal R, Wiswell RA. Prolonged exercise in pregnancy: glucose homeostasis, ventilatory and cardiovascular responses. Semin Perinatol 1996;20:315–27. 48. Stafne SN, Salvesen KA, Romundstad PR, et al. Regular exercise during pregnancy to prevent gestational diabetes: a randomized controlled trial. Obstetrics and gynecology 2012;119:29–36. 49. Vinter CA, Jørgensen JS, Ovesen P, et al. Metabolic effects of lifestyle intervention in obese pregnant women. Results from the randomized controlled trial ’Lifestyle in Pregnancy’ (LiP). Diabet Med 2014;31:1323–30. 50. Wang C, Wei Y, Zhang X, et al. Effect of regular exercise commenced in early pregnancy on the incidence of gestational diabetes mellitus in overweight and obese pregnant women: A randomized controlled trial. Diabetes Care 2016;39:163:e16 3–e164. 51. Wang C, Wei Y, Zhang X, et al. Effect of Regular Exercise Commenced in Early Pregnancy on the Incidence of Gestational Diabetes Mellitus in Overweight and Obese Pregnant Women: A Randomized Controlled Trial. Diabetes Care 2016;39:e163–e164. 52. Wolfe LA, Heenan AP, Bonen A. Aerobic conditioning effects on substrate responses during graded cycling in pregnancy. Can J Physiol Pharmacol 2003;81:696–703. 53. Young JC, Treadway JL. The effect of prior exercise on oral glucose tolerance in late gestational women. Eur J Appl Physiol Occup Physiol 1992;64:430–3. 54. Youngwanichsetha S, Phumdoung S, Ingkathawornwong T. The effects of mindfulness eating and yoga exercise on blood sugar levels of pregnant women with gestational diabetes mellitus. Appl Nurs Res 2014;27:227–30. |
| Davenport et al. 2019a | 1. Abirami P, Judie A. Integrated approach of yoga therapy on maternal and fetal outcome in gestational diabetes mellitus. International Journal of Pharmaceutical and Clinical Research 2015;7:377–82. 2. Bisson M, Alméras N, Dufresne SS, et al. A 12-week exercise program for pregnant women with obesity to improve physical activity levels: an open randomised preliminary study. PLoS One 2015;10:e0137742. 3. Collings CA, Curet LB, Mullin JP. Maternal and fetal responses to a maternal aerobic exercise program. Am J Obstet Gynecol 1983;145:702–7. 4. Denda OS, Dalkiranis AX, Denda MS, et al. The effect of some dietary components on body weight and fat deposition in pregnant women in combination with exercise during pregnancy and postpartum period. Studies in Physical Culture and Tourism 2005;12. 5. Dodd JM, Cramp C, Sui Z, et al. The effects of antenatal dietary and lifestyle advice for women who are overweight or obese on maternal diet and physical activity: the LIMIT randomised trial. BMC Med 2014;12:161. 6. Dumas GA, Reid JG, Wolfe LA, et al. Exercise, posture, and back pain during pregnancy. Clin Biomech 1995;10:98–103. 7. Dumith SC, Domingues MR, Mendoza-Sassi RA, et al. Physical activity during pregnancy and its association with maternal and child health indicators. Rev Saude Publica 2012;46:327–33. 8. Elden H, Ostgaard HC, Fagevik-Olsen M, et al. Treatments of pelvic girdle pain in pregnant women: adverse effects of standard treatment, acupuncture and stabilising exercises on the pregnancy, mother, delivery and the fetus/neonate. BMC Complement Altern Med 2008;8:34. 9. Feodor Nilsson S, Andersen PK, Strandberg-Larsen K, et al. Risk factors for miscarriage from a prevention perspective: a nationwide follow-up study. BJOG 2014;121:1375–85. 10. Guelfi KJ, Ong MJ, Crisp NA, et al. Regular exercise to prevent the recurrence of gestational diabetes mellitus: a randomized controlled trial. Obstet Gynecol 2016;128:819–27. 11. Guelinckx I, Devlieger R, Mullie P, et al. Effect of lifestyle intervention on dietary habits, physical activity, and gestational weight gain in obese pregnant women: a randomized controlled trial. Am J Clin Nutr 2010;91:373–80. 12. Haby K, Glantz A, Hanas R, et al. Mighty mums - an antenatal health care intervention can reduce gestational weight gain in women with obesity. Midwifery 2015;31:685–92. 13. Hawkins M, Hosker M, Marcus BH, et al. A pregnancy lifestyle intervention to prevent gestational diabetes risk factors in overweight Hispanic women: a feasibility randomized controlled trial. Diabet Med 2015;32:108–15. 14. Hollingsworth DR, Moore TR. Postprandial walking exercise in pregnant insulindependent (type I) diabetic women: reduction of plasma lipid levels but absence of a significant effect on glycemic control. Am J Obstet Gynecol 1987;157:1359–63. 15. Jackson RA, Stotland NE, Caughey AB, et al. Improving diet and exercise in pregnancy with video doctor counseling: a randomized trial. Patient Educ Couns 2011;83:203–9. 16. Jing W, Huang Y, Liu X, et al. The effect of a personalized intervention on weight gain and physical activity among pregnant women in China. Int J Gynaecol Obstet 2015;129:138–41. 17. Jovanovic-Peterson L, Durak EP, Peterson CM. Randomized trial of diet versus diet plus cardiovascular conditioning on glucose levels in gestational diabetes. Am J Obstet Gynecol 1989;161:415–9. 18. Kihlstrand M, Stenman B, Nilsson S, et al. Water-gymnastics reduced the intensity of back/low back pain in pregnant women. Acta Obstet Gynecol Scand 1999;78:180–5. 19. Kluge J, Hall D, Louw Q, et al. Specific exercises to treat pregnancy-related low back pain in a South African population. Int J Gynaecol Obstet 2011;113:187–91. 20. Koivusalo SB, Rönö K, Klemetti MM, et al. Gestational diabetes mellitus can be prevented by lifestyle intervention: the finnish gestational diabetes prevention study (RADIEL): a randomized controlled trial. Diabetes Care 2016;39:24–30. 21. Korpi-Hyövälti E, Heinonen S, Schwab U, et al. Effect of intensive counselling on physical activity in pregnant women at high risk for gestational diabetes mellitus. A clinical study in primary care. Prim Care Diabetes 2012;6:261–8. 22. Lawani MM, Alihonou E, Akplogan B, et al. [Effect of antenatal gymnastics on childbirth: a study on 50 sedentary women in the Republic of Benin during the second and third quarters of pregnancy]. Sante 2003;13:235–41. 23. Lombardi W, Wilson S, Peniston PB. Wellness intervention with pregnant soldiers. Mil Med 1999;164:22–9. 24. Luoto R, Kinnunen TI, Aittasalo M, et al. Primary prevention of gestational diabetes mellitus and large-for-gestational-age newborns by lifestyle counseling: a clusterrandomized controlled trial. PLoS Med 2011;8:e1001036. 25. Maconochie N, Doyle P, Prior S, et al. Risk factors for first trimester miscarriage-results from a UK-population-based case-control study. BJOG 2007;114:170–86. 26. Madsen M, Jørgensen T, Jensen ML, et al. Leisure time physical exercise during pregnancy and the risk of miscarriage: a study within the Danish National Birth Cohort. BJOG 2007;114:1419–26. 27. Magann EF, Evans SF, Weitz B, et al. Antepartum, intrapartum, and neonatal significance of exercise on healthy low-risk pregnant working women. Obstet Gynecol 2002;99:466–72. 28. Magnus P, Trogstad L, Owe KM, et al. Recreational physical activity and the risk of preeclampsia: a prospective cohort of Norwegian women. Am J Epidemiol 2008;168:952–7. 29. Petrov Fieril K, Glantz A, Fagevik Olsen M. The efficacy of moderate-to-vigorous resistance exercise during pregnancy: a randomized controlled trial. Acta Obstet Gynecol Scand 2015;94:35–42. 30. Phelan S, Phipps MG, Abrams B, et al. Randomized trial of a behavioral intervention to prevent excessive gestational weight gain: the Fit for Delivery Study. Am J Clin Nutr 2011;93:772–9. 31. Polley BA, Wing RR, Sims CJ. Randomized controlled trial to prevent excessive weight gain in pregnant women. Int J Obes Relat Metab Disord 2002;26:1494–502. 32. Poston L, Bell R, Croker H, et al. Effect of a behavioural intervention in obese pregnant women (the UPBEAT study): a multicentre, randomised controlled trial. Lancet Diabetes Endocrinol 2015;3:767–77. 33. Prabhu N. Effect of a exercise by pregnant women and birth weight: a randomized controlled trial. Journal of Evolution of Medical and Dental Sciences 2015;04:1509–16. 34. Rauh K, Gabriel E, Kerschbaum E, et al. Safety and efficacy of a lifestyle intervention for pregnant women to prevent excessive maternal weight gain: a cluster-randomized controlled trial. BMC Pregnancy Childbirth 2013;13:151. 35. Renault KM, Nørgaard K, Nilas L, et al. The Treatment of Obese Pregnant Women (TOP) study: a randomized controlled trial of the effect of physical activity intervention assessed by pedometer with or without dietary intervention in obese pregnant women. Am J Obstet Gynecol 2014;210:134.e1–e9. 36. Ronnberg AK, Ostlund I, Fadl H, et al. Intervention during pregnancy to reduce excessive gestational weight gain—a randomised controlled trial. BJOG 2015;122:537–44. 37. Sagedal LR, Vistad I, Øverby NC, et al. The effect of a prenatal lifestyle intervention on glucose metabolism: results of the Norwegian fit for delivery randomized controlled trial. BMC Pregnancy Childbirth 2017;17:167. 38. Seneviratne SN, Jiang Y, Derraik J, et al. Effects of antenatal exercise in overweight and obese pregnant women on maternal and perinatal outcomes: a randomised controlled trial. BJOG 2016;123:588–97. 39. Sun Y, Zhao H. The effectiveness of lifestyle intervention in early pregnancy to prevent gestational diabetes mellitus in Chinese overweight and obese women: a quasiexperimental study. Appl Nurs Res 2016;30:125–30. 40. Taniguchi C, Sato C. Home-based walking during pregnancy affects mood and birth outcomes among sedentary women: A randomized controlled trial. Int J Nurs Pract 2016;22:420–6. 41. Ussher M, Lewis S, Aveyard P, et al. Physical activity for smoking cessation in pregnancy: randomised controlled trial. BMJ 2015;350:h2145. 42. Vinter CA, Jensen DM, Ovesen P, et al. The LiP (Lifestyle in Pregnancy) study: a randomized controlled trial of lifestyle intervention in 360 obese pregnant women. Diabetes Care 2011;34:2502–7. 43. Xu G, Wu Y, Yang L, et al. Risk factors for early miscarriage among Chinese: a hospitalbased case-control study. Fertil Steril 2014;101:1663–70. 44. Zhang X, Li J, Gu Y, et al. A pilot study on environmental and behavioral factors related to missed abortion. Environ Health Prev Med 2011;16:273–8. |
| Davenport et al. 2019b | 1. Abirami P, Judie A. Integrated approach of yoga therapy on maternal and fetal outcome in gestational diabetes mellitus. International Journal of Pharmaceutical and Clinical Research. 2015;7(6):377-82. 2. Althuizen E vdWC, van Mechelen W, Seidell JC, van Poppel MNM. The effect of a counselling intervention on weight changes during and after pregnancy: a randomised trial. British Journal of Obstetrics and Gynecology. 2013;120:92-9. 3. Asbee, S M, Jenkins, T R, Butler, J R, et al. Preventing excessive weight gain during pregnancy through dietary and lifestyle counseling: a randomized controlled trial. Obstetrics and gynecology. 2009;113(2 Pt 1):305. 4. Avery MD, Leon AS, Kopher RA. Effects of a partially home-based exercise program for women with gestational diabetes. Obstet Gynecol. 1997;89(1):10-5. 5. Baciuk EP, Pereira RI, Cecatti JG, Braga AF, Cavalcante SR. Water aerobics in pregnancy: Cardiovascular response, labor and neonatal outcomes. Reprod Health. 2008;5:10. 6. Banerjee A, V. Mahalakshmi V, R. Baranitharan R. Effect of Antenatal Exercise Program with and without Abdominal Strengthening Exercises on Diastasis Rectus Abdominis - A Post Partum Follow Up. Indian Journal of Physiotherapy & Occupational Therapy. 2013;7(4):123-6. 7. Barakat R, Alonso G, Rojo JJ. Physical exercise during pregnancy and its relationship with the duration of stages of labor. Prog Obstet Ginecol. 2005;48(2):61-8. 8. Barakat R, Cordero Y, Coteron J, Luaces M, Montejo R. Exercise during pregnancy improves maternal glucose screen at 24-28 weeks: a randomised controlled trial. British Journal of Sports Medicine. 2012;46(9):656-61. 9. Barakat R, Pelaez M, Cordero Y, Perales M, Lopez C, Coteron J, et al. Exercise during pregnancy protects against hypertension and macrosomia: randomized clinical trial. Am J Obstet Gynecol. 2016;214(5):649 e1-8. 10. Barakat R, Pelaez M, Lopez C, Lucia A, Ruiz JR. Exercise during pregnancy and gestational diabetes-related adverse effects: a randomised controlled trial. Br J Sports Med. 2013;47(10):630-6. 11. Barakat R, Pelaez M, Montejo R, Luaces M, Zakynthinaki M. Exercise during pregnancy improves maternal health perception: a randomized controlled trial. Am J Obstet Gynecol. 2011;204(5):402 e17. 12. Barakat R, Perales M, Bacchi M, Coteron J, Refoyo I. A program of exercise throughout pregnancy. Is it safe to mother and newborn? American Journal of Health Promotion. 2014;29(1):2-8. 13. Barakat R, Ruiz JR, Stirling JR, Zakynthinaki M, Lucia A. Type of delivery is not affected by light resistance and toning exercise training during pregnancy: a randomized controlled trial. Am J Obstet Gynecol. 2009;201(6):590 e1-6. 14. Battle CL, Uebelacker LA, Magee SR, Sutton KA, Miller IW. Potential for prenatal yoga to serve as an intervention to treat depression during pregnancy. Womens Health Issues. 2015;25(2):134-41. 15. Beckmann CR, Beckmann CA. Effect of a structured antepartum exercise program on pregnancy and labor outcome in primiparas. J Reprod Med. 1990;35(7):704-9. 16. Beyaz EA, Ozcan E, Ketenci A, Beyaz MM. The effectiveness of pregnancy rehabilitation: Effects on low back pain and calf cramps during pregnancy and pregnancy outcome. Nobel Medicus. 2011;7(2):67-74. 17. Bisson M, Almeras N, Dufresne SS, Robitaille J, Rheaume C, Bujold E, et al. A 12-Week Exercise Program for Pregnant Women with Obesity to Improve Physical Activity Levels: An Open Randomised Preliminary Study. PLoS One. 2015;10(9):e0137742. 18. Bo K, Fleten C, Nystad W. Effect of antenatal pelvic floor muscle training on labor and birth. Obstet Gynecol. 2009;113(6):1279-84. 19. Bo K, Hilde G, Staer-Jensen J, Siafarikas F, Tennfjord MK, Engh ME. Does general exercise training before and during pregnancy influence the pelvic floor "opening" and delivery outcome? A 3D/4D ultrasound study following nulliparous pregnant women from mid-pregnancy to childbirth. Br J Sports Med. 2015;49:196-9. 20. Bo S, Rosato R, Ciccone G, Canil S, Gambino R, Poala CB, et al. Simple lifestyle recommendations and the outcomes of gestational diabetes. A 2 x 2 factorial randomized trial. Diabetes Obes Metab. 2014;16(10):1032-5. 21. Botkin C, Driscoll CE. Maternal aerobic exercise: newborn effects. Fam Pract Res J. 1991;11(4):387-93. 22. Brenner IK, Wolfe LA, Monga M, McGrath MJ. Physical conditioning effects on fetal heart rate responses to graded maternal exercise. Medicine & Science in Sports & Exercise. 1999;31(6):792-9. 23. Callaway LK, Colditz PB, Byrne NM, Lingwood BE, Rowlands IJ, Foxcroft K, et al. Prevention of gestational diabetes: feasibility issues for an exercise intervention in obese pregnant women. Diabetes Care. 2010;33(7):1457- 9. 24. Carpenter RE, Emery SJ, Uzun O, Rassi D, Lewis MJ. Influence of antenatal physical exercise on heart rate variability and QT variability. Journal of Maternal-Fetal & Neonatal Medicine. 2017;30(1):79. 25. Carpenter RE, Emery SJ, Uzun O, Silva LAD, Lewis MJ. Influence of antenatal physical exercise on haemodynamics in pregnant women: A flexible randomisation approach. BMC Pregnancy and Childbirth. 2015;15:156. 26. Chávez VG, Sánchez, M. D. P V, Rasch JRK. Effect of pelvic floor exercise during pregnancy and puerperium on prevention of urinary stress incontinence. Ginecol Obstet Mex. 2004;72(628-636). 27. Chiarello CM, Falzone LA, McCaslin KE, Patel MN, Ulery KR. The effects of an exercise program on diastasis recti abdominis in pregnant women [corrected] [published erratum appears in J WOMENS HEALTH PHYS THER 2005;29(3):76]. 2005. 28. Choi J, Lee JH, Vittinghoff E, Fukuoka Y. mHealth Physical Activity Intervention: A Randomized Pilot Study in Physically Inactive Pregnant Women. Maternal & Child Health Journal. 2016;20(5):1091101. 29. Chuntharapat S, Petpichetchian W, Hatthakit U. Yoga during pregnancy: effects on maternal comfort, labor pain and birth outcomes. Complement Ther Clin Pract. 2008;14(2):105-15. 30. Clapp JF, 3rd. The course of labor after endurance exercise during pregnancy. Am J Obstet Gynecol. 1990;163(6 Pt 1):1799-805. 31. Collings CA, Curet LB, Mullin JP. Maternal and fetal responses to a maternal aerobic exercise program. Am J Obstet Gynecol. 1983;145(6):702-7. 32. controlled trial. BMC Pregnancy Childbirth. 2013;13:151. 33. Cordero Rodriguez Y, Peláez Puente M, Miguel Abad Md, Perales Santaella M, Barakat Carballo R. Puede el ejercicio físico moderado durante el embarazo actuar como un factor de prevención de la Diabetes Gestacional? /Can moderate physical exercise during pregnancy act as a factor in preventing Gestational Diabetes? RICYDE Rev int cienc deporte. 2012;27(8):3-19. 34. Cordero Y, Mottola MF, Vargas J, Blanco M, Barakat R. Exercise Is Associated with a Reduction in Gestational Diabetes Mellitus. Medicine and Science in Sports and Exercise. 2015;47(7):1328-33. 35. Dale E, Mullinax KM, Bryan DH. Exercise during pregnancy: effects on the fetus. Canadian Journal of Applied Sport Sciences. 1982;7(2):98-103 36. de Barros MC, Lopes MA, Francisco RP, Sapienza AD, Zugaib M. Resistance exercise and glycemic control in women with gestational diabetes mellitus. Am J Obstet Gynecol. 2010;203(6):556 e1-6. 37. Dias LA, Driusso P, Aita DL, Quintana SM, Bo K, Ferreira CH. Effect of pelvic floor muscle training on labour and newborn outcomes: a randomized controlled trial. Rev Bras Fisioter. 2011;15(6):487-93. 38. Dodd J, Grivell R, Owens J. Antenatal Dietary and Lifestyle Interventions for Women Who are Overweight or Obese: Outcomes from the LIMIT Randomized Trial. Current Nutrition Reports. 2014;3(4):392-9. 39. Downs DS, Hausenblas HA. Pregnant women's third trimester exercise behaviors, body mass index, and pregnancy outcomes. PsycholHealth. 2007;22(5):545-59. 40. Eggen MH, Stuge B, Mowinckel P, Jensen KS, Hagen KB. Can supervised group exercises including ergonomic advice reduce the prevalence and severity of low back pain and pelvic girdle pain in pregnancy? A randomized controlled trial. Phys Ther. 2012;92(6):781-90. 41. Elden H, Ostgaard HC, Fagevik-Olsen M, Ladfors L, Hagberg H. Treatments of pelvic girdle pain in pregnant women: adverse effects of standard treatment, acupuncture and stabilising exercises on the pregnancy, mother, delivery and the fetus/neonate. BMC Complement Altern Med. 2008;8:34. 42. Ferrara A, Hedderson MM, Brown SD, Albright CL, Ehrlich SF, Tsai AL, et al. The Comparative Effectiveness of Diabetes Prevention Strategies to Reduce Postpartum Weight Retention in Women With Gestational Diabetes Mellitus: The Gestational Diabetes' Effects on Moms (GEM) Cluster Randomized Controlled Trial. Diabetes Care. 2016;39(1):65-74. 43. Fritel X, de Tayrac R, Bader G, Savary D, Gueye A, Deffieux X, et al. Preventing Urinary Incontinence With Supervised Prenatal Pelvic Floor Exercises: A Randomized Controlled Trial. Obstet Gynecol. 2015;126(2):370-7. 44. Gaston A, Prapavessis H. Tired, moody and pregnant? Exercise may be the answer. Psychol Health. 2013;28(12):1353-69. 45. Ghodsi Z, Asltoghiri M. Effects of aerobic exercise training on maternal and neonatal outcome: a randomized controlled trial on pregnant women in Iran. J Pak Med Assoc. 2014;64(9):1053-6. 46. Guelfi KJ, Ong MJ, Crisp NA, Fournier PA, Wallman KE, Grove JR, et al. Regular Exercise to Prevent the Recurrence of Gestational Diabetes Mellitus: A Randomized Controlled Trial. Obstetrics & Gynecology. 2016;128(4):819-27. 47. Guelinckx I, Devlieger R, Mullie P, Vansant G. Effect of lifestyle intervention on dietary habits, physical activity, and gestational weight gain in obese pregnant women: a randomized controlled trial. Am J Clin Nutr. 2010;91(2):373-80. 48. Guszkowska M, Sempolska K, Zaremba A, Langwald M. Exercise or relaxation? Which is more effective in improving the emotional state of pregnant women? 2013. 49. Guszkowska M. PHYSICAL ACTIVITY IN RELATION TO AFFECTIVE STATES AND LABOR ANXIETY IN PREGNANT WOMEN. Med Sport. 2011;15(3):114-8. 50. Haakstad LA, Bo K. Effect of regular exercise on prevention of excessive weight gain in pregnancy: a randomised controlled trial. Eur J Contracept Reprod Health Care. 2011;16(2):116-25. 51. Halse RE, Wallman KE, Dimmock JA, Newnham JP, Guelfi KJ. Home-Based Exercise Improves Fitness and Exercise Attitude and Intention in Women with GDM. Med Sci Sports Exerc. 2015;47(8):1698-704. 52. Hollingsworth DR, Moore TR. Postprandial walking exercise in pregnant insulin-dependent (type I) diabetic women: reduction of plasma lipid levels but absence of a significant effect on glycemic control. Am J Obstet Gynecol. 1987;157(6):1359-63. 53. Horns PN, Ratcliffe LP, Leggett JC, Swanson MS. Pregnancy outcomes among active and sedentary primiparous women. Journal of Obstetric, Gynecologic, & Neonatal Nursing. 1996;25(1):4954. 54. Hui A, Back L, Ludwig S, Gardiner P, Sevenhuysen G, Dean H, et al. Lifestyle intervention on diet and exercise reduced excessive gestational weight gain in pregnant women under a randomised controlled trial. BJOG. 2012;119(1):70-7. 55. Hui AL, Back L, Ludwig S, Gardiner P, Sevenhuysen G, Dean HJ, et al. Effects of lifestyle intervention on dietary intake, physical activity level, and gestational weight gain in pregnant women with different pre-pregnancy Body Mass Index in a randomized control trial. BMC Pregnancy Childbirth. 2014;14:331. 56. Kariminia A, Chamberlain ME, Keogh J, Shea A. Randomised controlled trial of effect of hands and knees posturing on incidence of occiput posterior position at birth. BMJ. 2004;328(7438):490. 57. Kasawara KT, Burgos CS, do Nascimento SL, Ferreira NO, Surita FG, Pinto ESJL. Maternal and Perinatal Outcomes of Exercise in Pregnant Women with Chronic Hypertension and/or Previous Preeclampsia: A Randomized Controlled Trial. ISRN Obstet Gynecol. 2013;2013:857047. 58. Kihlstrand M, Stenman B, Nilsson S, Axelsson O. Water-gymnastics reduced the intensity of back/low back pain in pregnant women. Acta Obstet Gynecol Scand. 1999;78(3):180-5. 59. Ko PC, Liang CC, Chang SD, Lee JT, Chao AS, Cheng PJ. A randomized controlled trial of antenatal pelvic floor exercises to prevent and treat urinary incontinence. Int Urogynecol J. 2011;22(1):17-22. 60. Kobe H, Nakai A, Koshino T, Araki T. Effect of regular maternal exercise on lipid peroxidation levels and antioxidant enzymatic activities before and after delivery. J Nippon Med Sch. 2002;69(6):5428. 61. Koivusalo SB, Rono K, Klemetti MM, et al. Gestational Diabetes Mellitus Can Be Prevented by Lifestyle Intervention: The Finnish Gestational Diabetes Prevention Study (RADIEL): A Randomized Controlled Trial. Diabetes Care. 2016;39(1):24-30. 62. Kong KAI, Campbell C, Foster R, Peterson A, Lanningham-Foster L. A Pilot Walking Program Promotes Moderate-Intensity Physical Activity during Pregnancy. Medicine & Science in Sports & Exercise. 2014;46(3):462-71. 63. Kulpa PJ, White BM, Visscher R. Aerobic exercise in pregnancy. Am J Obstet Gynecol. 1987;156(6):1395-403. 64. Lawani MM, Alihonou E, Akplogan B, Poumarat G, Okou L, Adjadi N. [Effect of antenatal gymnastics on childbirth: a study on 50 sedentary women in the Republic of Benin during the second and third quarters of pregnancy]. Sante. 2003;13(4):235-41. 65. Lindqvist M, Lindkvist M, Eurenius E, Persson M, Ivarsson A, Mogren I. Leisure time physical activity among pregnant women and its associations with maternal characteristics and pregnancy outcomes. Sexual and Reproductive Healthcare. 2016;9:14-20. 66. Lombardi W, Wilson S, Peniston PB. Wellness intervention with pregnant soldiers. Mil Med. 1999;164(1):22-9. 67. Lynch AM, Goodman C, Choy PL, Dawson B, Newnham JP, McDonald S, et al. Maternal physiological responses to swimming training during the second trimester of pregnancy. Res Sports Med. 2007;15(1):33-45. 68. Magann EF, Evans SF, Weitz B, Newnham J. Antepartum, intrapartum, and neonatal significance of exercise on healthy low-risk pregnant working women. Obstet Gynecol. 2002;99(3):466-72. 69. Marquez-Sterling S, Perry AC, Kaplan TA, Halberstein RA, Signorile JF. Physical and psychological changes with vigorous exercise in sedentary primigravidae. Med Sci Sports Exerc. 2000;32(1):58-62. 70. Mason L, Roe B, Wong H, Davies J, Bamber J. The role of antenatal pelvic floor muscle exercises in prevention of postpartum stress incontinence: a randomised controlled trial. J Clin Nurs. 2010;19(1920):2777-86. 71. Melzer K, Schutz Y, Soehnchen N, et al. Effects of recommended levels of physical activity on pregnancy outcomes. American Journal of Obstetrics & Gynecology. 2010;202(3):266.e1-6. 72. Miquelutti MA, Cecatti JG, Makuch MY. Evaluation of a birth preparation program on lumbopelvic pain, urinary incontinence, anxiety and exercise: a randomized controlled trial. BMC Pregnancy Childbirth. 2013;13:154. 73. Morkved S, Bo K, Schei B, Salvesen KA. Pelvic floor muscle training during pregnancy to prevent urinary incontinence: a single-blind randomized controlled trial. Obstet Gynecol. 2003;101(2):313-9. 74. Nascimento SL, Surita FG, Parpinelli MA, Siani S, Pinto e Silva JL. The effect of an antenatal physical exercise programme on maternal/perinatal outcomes and quality of life in overweight and obese pregnant women: a randomised clinical trial. BJOG. 2011;118(12):1455-63. 75. Nielsen CA, Sigsgaard I, Olsen M, Tolstrup M, Danneskiold-Samsoee B, Bock JE. Trainability of the pelvic floor. A prospective study during pregnancy and after delivery. Acta Obstet Gynecol Scand. 1988;67(5):437-40. 76. Nobles C, Marcus BH, Stanek rEJ, Braun B, Whitcomb BW, Solomon CG, et al. Effect of an exercise intervention on gestational diabetes mellitus: a randomized controlled trial. Obstetrics and gynecology. 2015;125(5):1195. 77. O'Connor PJ, Poudevigne MS, Cress ME, Motl RW, Clapp JF, 3rd. Safety and efficacy of supervised strength training adopted in pregnancy. J Phys Act Health. 2011;8(3):309-20. 78. Okido M, Valeri F, Martins W, Ferreira C, Duarte G, Cavalli R. Assessment of foetal wellbeing in pregnant women subjected to pelvic floor muscle training: a controlled randomised study. International Urogynecology Journal. 2015;26(10):1475-81. 79. Ong MJ, Guelfi KJ, Hunter T, Wallman KE, Fournier PA, Newnham JP. Supervised home-based exercise may attenuate the decline of glucose tolerance in obese pregnant women. Diabetes Metab. 2009;35(5):418-21. 80. Oostdam N, van Poppel MNM, Wouters MGAJ, Eekhoff EMW, Bekedam DJ, Kuchenbecker WKH, et al. No effect of the FitFor2 exercise programme on blood glucose, insulin sensitivity, and birthweight in pregnant women who were overweight and at risk for gestational diabetes: results of a randomised controlled trial. Bjog-an International Journal of Obstetrics and Gynaecology. 2012;119(9):1098-107. 81. Orawan L, Preecha W. Effect of pelvic floor muscle training (PFMT) during pregnancy on bladder neck descend and delivery. Journal of the Medical Association of Thailand. 2014;97(Suppl 8):S156-63. 82. Perales M, Cordero Y, Vargas M, Lucia A, Barakat R. Exercise and depression in overweight and obese pregnant women: A randomised controlled trial. Archivos de Medicina del Deporte. 2015;32:707. 83. Perales M, Mateos S, Vargas M, Sanz I, Lucia A, Barakat R. Fetal and maternal heart rate responses to exercise in pregnant women. A randomized controlled trial. Archivos de Medicina del Deporte. 2015;32(6):361-7. 84. Perales M, Santos-Lozano A, Sanchis-Gomar F, Luaces M, Pareja-Galeano H, Garatachea N, et al. Maternal Cardiac Adaptations to a Physical Exercise Program during Pregnancy. Med Sci Sports Exerc. 2016;48(5):896-906. 85. Peterson CD, Haas M, Gregory WT. A pilot randomized controlled trial comparing the efficacy of exercise, spinal manipulation, and neuro emotional technique for the treatment of pregnancy-related low back pain. Chiropr Man Therap. 2012;20(1):18. 86. Petrella E, Malavolti M, Bertarini V, Pignatti L, Neri I, Battistini NC, et al. Gestational weight gain in overweight and obese women enrolled in a healthy lifestyle and eating habits program. J Matern Fetal Neonatal Med. 2014;27(13):1348-52. 87. Petrov Fieril K, Glantz A, Fagevik Olsen M. The efficacy of moderate-to-vigorous resistance exercise during pregnancy: a randomized controlled trial. Acta Obstetricia et Gynecologica Scandinavica. 2015;94(1):35-42. 88. Phelan S, Phipps MG, Abrams B, Darroch F, Schaffner A, Wing RR. Randomized trial of a behavioral intervention to prevent excessive gestational weight gain: the Fit for Delivery Study. Am J Clin Nutr. 2011;93(4):772-9. 7 89. Pinzon DC, Zamora K, Martinez JH, et al. Type of delivery and gestational age is not affected by pregnant Latin-American women engaging in vigorous exercise: a secondary analysis of data from a controlled randomized trial. Revista de Salud Publica. 2012;14(5):731-43. 90. Piravej K, Saksirinukul R. Survey of patterns, attitudes, and the general effects of exercise during pregnancy in 203 Thai pregnant women at King Chulalongkorn Memorial Hospital. Journal of the Medical Association of Thailand. 2001;84(Suppl 1):S276-82. 91. Polley BA, Wing RR, Sims CJ. Randomized controlled trial to prevent excessive weight gain in pregnant women. Int J Obes Relat Metab Disord. 2002;26(11):1494-502. 92. Pomerance JJ, Gluck L, Lynch VA. Physical fitness in pregnancy: its effect on pregnancy outcome. American Journal of Obstetrics & Gynecology. 1974;44(3):383-7. 93. Poston L, Bell R, Croker H, Flynn AC, Godfrey KM, Goff L, et al. Effect of a behavioural intervention in obese pregnant women (the UPBEAT study): a multicentre, randomised controlled trial. Lancet Diabetes Endocrinol. 2015;3(10):767-77. 94. Price BB, Amini SB, Kappeler K. Exercise in pregnancy: effect on fitness and obstetric outcomes a randomized trial. Med Sci Sports Exerc. 2012;44(12):2263-9. 95. Putnam KF, Mueller LA, Magann EF, et al. Evaluating effects of self-reported domestic physical activity on pregnancy and neonatal outcomes in "stay at home" military wives. Mil Med. 2013;178(8):893-8. 96. Rauh K, Gabriel E, Kerschbaum E, Schuster T, von Kries R, Amann-Gassner U, et al. Safety and efficacy of a lifestyle intervention for pregnant women to prevent excessive maternal weight gain: a cluster-randomized 97. Reilly ET, Freeman RM, Waterfield MR, Waterfield AE, Steggles P, Pedlar F. Prevention of postpartum stress incontinence in primigravidae with increased bladder neck mobility: a randomised controlled trial of antenatal pelvic floor exercises. Bjog. 2002;109(1):68-76. 98. Renault KM, Norgaard K, Nilas L, Carlsen EM, Cortes D, Pryds O, et al. The Treatment of Obese Pregnant Women (TOP) study: a randomized controlled trial of the effect of physical activity intervention assessed by pedometer with or without dietary intervention in obese pregnant women. Am J Obstet Gynecol. 2014;210(2):134 e1-9. 99. Rice PL, Fort IL. The relationship of maternal exercise on labor, delivery and health of the newborn. J Sports Med Phys Fitness. 1991;31(1):95-9. 100. Ruiz JR, Perales M, Pelaez M, Lopez C, Lucia A, Barakat R. Supervised exercise-based intervention to prevent excessive gestational weight gain: a randomized controlled trial. Mayo Clin Proc. 2013;88(12):1388-97. 101. Sagedal LR, Overby NC, Bere E, Torstveit MK, Lohne-Seiler H, Smastuen M, et al. Lifestyle intervention to limit gestational weight gain: the Norwegian Fit for Delivery randomised controlled trial. BJOG. 2017;124(1):97-109. 102. Salvesen KA, Stafne SN, Eggebo TM, Morkved S. Does regular exercise in pregnancy influence duration of labor? A secondary analysis of a randomized controlled trial. Acta Obstet Gynecol Scand. 2014;93(1):73-9. 103. Seneviratne SN, Jiang Y, Derraik J, McCowan L, Parry GK, Biggs JB, et al. Effects of antenatal exercise in overweight and obese pregnant women on maternal and perinatal outcomes: a randomised controlled trial. BJOG. 2016;123(4):588-97. 104. Stafne SN, Salvesen KA, Romundstad PR, Eggebo TM, Carlsen SM, Morkved S. Regular exercise during pregnancy to prevent gestational diabetes: a randomized controlled trial. Obstet Gynecol. 2012;119(1):29-36. 105. Taniguchi C, Sato C. Home-based walking during pregnancy affects mood and birth outcomes among sedentary women: A randomized controlled trial. International Journal of Nursing Practice. 2016;22(5):420-6. 106. To WWK, Wong MWN. Bone mineral density changes during pregnancy in actively exercising women as measured by quantitative ultrasound. Archives of Gynecology and Obstetrics. 2012;286(2):357-63. 107. Tomic V, Sporis G, Tomic J, Milanovic Z, Zigmundovac-Klaic D, Pantelic S. The effect of maternal exercise during pregnancy on abnormal fetal growth. Croat Med J. 2013;54(4):362-8. 108. Uebelacker LA, Battle CL, Sutton KA, Magee SR, Miller IW. A pilot randomized controlled trial comparing prenatal yoga to perinatal health education for antenatal depression. Arch Womens Ment Health. 2016;19(3):543-7. 109. Ussher M, Lewis S, Aveyard P, et al. Physical activity for smoking cessation in pregnancy: randomised controlled trial. British Medical Journal. 2015;350:h2145. 110. Vesco KK, Karanja N, King JC, Gillman MW, Leo MC, Perrin N, et al. Efficacy of a group-based dietary intervention for limiting gestational weight gain among obese women: a randomized trial. Obesity (Silver Spring, Md). 2014;22(9):1989-96. 111. Vinter CA, Jensen DM, Ovesen P, Beck-Nielsen H, Jorgensen JS. The LiP (Lifestyle in Pregnancy) study: a randomized controlled trial of lifestyle intervention in 360 obese pregnant women. Diabetes Care. 2011;34(12):2502- 7. 112. Vollebregt KC, Wolf H, Boer K, van der Wal MF, Vrijkotte TGM, Bonsel GJ. Does physical activity in leisure time early in pregnancy reduce the incidence of preeclampsia or gestational hypertension? Acta Obstetricia Et Gynecologica Scandinavica. 2010;89(2):261-7. 113. Wallace AM, Boyer DB, Dan A, Holm K. Aerobic exercise, maternal self-esteem, and physical discomforts during pregnancy. J Nurse Midwifery. 1986;31(6):255-62. 114. Wang X, Li GY, Deng ML. Pelvic floor muscle training as a persistent nursing intervention: Effect on delivery outcome and pelvic floor myodynamia. International Journal of Nursing Sciences. 2014;1(1):48-52. 115. Woldringh C, Wijngaart Mvd, Albers-Heitner P, Lycklama a N, A A, Lagro-Janssen T. Pelvic floor muscle training is not effective in women with UI in pregnancy: a randomised controlled trial. Int Urogynecol J Pelvic Floor Dysfunct. 2007;18(4):383-90. 116. Youngwanichsetha S, Phumdoung S, Ingkathawornwong T. The effects of mindfulness eating and yoga exercise on blood sugar levels of pregnant women with gestational diabetes mellitus. Appl Nurs Res. 2014;27(4):227- 30. |
| Di Mascio *et al*. 2016 | 1. Barakat, R. J.R. Stirling, A. Lucia Does exercise training during pregnancy affect gestational age? A randomized controlled trial Br J Sports Med, 42 (2008), pp. 674-678 2. Barakat, R.,  M. Perales, M. Bacchi, J. Coteron, I. Refoyo A program of exercise throughout pregnancy. Is it safe to mother and newborn? Am J Health Promot, 29 (2014), pp. 2-8 3. Barakat, R., M. Pelaez, R. Montejo, I. Refoyo, J. Coteron Exercise throughout pregnancy does not cause preterm delivery: a randomized, controlled trial J Phys Act Health, 11 (2014), pp. 1012-1017 4. Barakat, R., M. Pelaez, Y. Cordero, et al. Exercise during pregnancy protects against hypertension and macrosomia: randomized clinical trial Am J Obstet Gynecol, 214 (2016), pp. 649.e1-649.e8 5. Calvalcante, S.R., J.G. Cecatti, R.I. Pereira, E.P. Baciuk, A.L. Bernardo, C. Silveira Water aerobics II: maternal body composition and perinatal outcomes after a program for low risk pregnant women Reprod Health, 6 (2009), p. 1 6. Carpenter MW, Sady SP, Sady MA, Haydon BB, Coustah DR, Thompson PD. Effects of exercise training in midpregnancy: a randomized controlled trial. Presented at the 37th annual meeting of the Society for Gynecologic Investigation, St Louis. Missouri, March 21-24,1990;Abstr.497, p. 345 7. Haakstad, L.A.H., K. Bo Exercise in pregnant women and birth weight: a randomized controlled trial BMC Pregnancy Childbirth, 11 (2011;30), p. 66 8. Prevedel, T I. Calderon, M. DeConti, E. Consonni, M. Rudge Maternal and perinatal effects of hydrotherapy in pregnancy Revista Brasil Ginecol Obstet, 25 (2003), pp. 53-59 9. Ruiz, J.R., M. Perales, M. Pelaez, C. Lopez, A. Lucia, R. Barakat Supervised exercise-based intervention to prevent gestational weight gain: a randomized controlled trial Mayo Clin Proc, 88 (2013), pp. 1388-1397 |
| Gresham et al. 2014 | 1. Aaltonen J, Ojala T, Laitinen K, Piirainen TJ, Poussa TA, Isolauri E. Evidence of infant blood pressure programming by maternal nutrition during pregnancy: a prospective randomized controlled intervention study. J Pediatr 2008;152:79–84. 2. Bech BH, Obel C, Henriksen TB, Olsen J. Effect of reducing caffeine intake on birth weight and length of gestation: randomised controlled trial. BMJ 2007;334:409–12. 3. Briley C, Flanagan NL, Lewis N. In-home prenatal nutrition intervention increased dietary iron intakes and reduced low birthweight in low-income African-American women. J Am Diet Assoc 2002;102:984–7. 4. Ceesay SM, Prentice AM, Cole TJ, Foord F, Weaver LT, Poskitt EM, Whitehead RG. Effects on birth weight and perinatal mortality of maternal dietary supplements in rural Gambia: 5 year randomised controlled trial. BMJ 1997;315:786–90. 5. Chan GM, McElligott K, McNaught T, Gill G. Effects of dietary calcium intervention on adolescent mothers and newborns: a randomized controlled trial. Obstet Gynecol 2006;108:565–71. 6. Courville AB, Harel O, Lammi-Keefe C. Consumption of a DHAcontaining functional food during pregnancy is associated with lower infant ponderal index and cord plasma insulin concentration. Br J Nutr 2011;106:208–12. 8. Luoto R, Kinnunen T, Aittasalo M, Kolu P, Raitanen J, Ojala K, Mansikkama ̈ki K, Lamberg S, Vasankari T, Komulainen T, et al. Primary prevention of gestational diabetes mellitus and large-for-gestational age newborns by lifestyle counseling: a cluster-randomized controlled trial. PLoS Med 2011;8:e1001036. 7. de Groot RHM, Hornstra G, van Houwelingen AC, Roumen F. Effect of alpha-linolenic acid supplementation during pregnancy on maternal and neonatal polyunsaturated fatty acid status and pregnancy outcome. Am J Clin Nutr 2004;79:251–60. 8. Fard NM, Mehrabian F, Sarraf-Zadegan N, Sajadi F. Fat-modified diets during pregnancy and lactation and serum lipids after birth. Indian J Pediatr 2004;71:683–7. 9. Kafatos AG, Vlachonikolis I, Codrington C. Nutrition during pregnancy: the effects of an educational intervention program in Greece. Am J Clin Nutr 1989;50:970–9. 10. Khoury J, Henriksen T, Christophersen B, Tonstad S. Effect of a cholesterol-lowering diet on maternal, cord, and neonatal lipids, and pregnancy outcome: a randomized clinical trial. Am J Obstet Gynecol 2005;193:1292– 301. 11. Knuist M, Bonsel GJ, Zondervan HA, Treffers PE. Low sodium diet and pregnancy-induced hypertension: a multi-centre randomised controlled trial. Br J Obstet Gynaecol 1998;105:430–4. 12. Kusin JA, Kardjati S, Houtkooper JM, Renqvist UH. Energy supplementation during pregnancy and postnatal growth. Lancet 1992;340:623–6. 13. Mardones-Santander F, Rosso P, Stekel A, Ahumada E, Llaguno S, Pizarro F, Salinas J, Vial I, Walter T. Effect of a milk-based food supplement on maternal nutritional status and fetal growth in underweight Chilean women. Am J Clin Nutr 1988;47:413–9. 14. McDonald EC, Pollitt E, Mueller W. The bacon chow study: maternal nutritional supplementation and birth weight of offspring. Am J Clin Nutr 1981;34:2133–44. 15. Metcoff J, Costiloe P, Crosby WM, Dutta S, Sandstead HH, Milne D, Bodwell CE, Majors SH. Effect of food supplementation (WIC) during pregnancy on birth weight. Am J Clin Nutr 1985;41:933–47. 16. Mora JO, Clement J, Christiansen N, Suescun J, Wagner M, Herrera MG. Nutritonal supplementation and the outcome of pregnancy. III. Perinatal and neonatal mortality. Nutr Rep Int 1978;18:167–75. 17. O’Connor MJ, Whaley SE. Brief intervention for alcohol use by pregnant women. Am J Public Health 2007;97:252–8. 18. Quinlivan JA, Lam LT, Fisher J. A randomised trial of a four-step multidisciplinary approach to the antenatal care of obese pregnant women. Aust N Z J Obstet Gynaecol 2011;51:141–6. 19. Ross SM, Nel E, Naeye RL. Differing effects of low and high bulk maternal dietary supplements during pregnancy. Early Hum Dev 1985;10:295–302. 20. Rush D, Stein Z, Susser M. A randomized controlled trial of prenatal nutritional supplementation in New York City. Pediatrics 1980;65:683–97. 21. Smuts CM, Borod E, Peeples JM, Carlson SE. High-DHA eggs: feasibility as a means to enhance circulating DHA in mother and infant. Lipids 2003;38:407–14. 22. Smuts CM, Huang M, Mundy D, Plasse T, Major S, Carlson SE. A randomized trial of docosahexaenoic acid supplementation during the third trimester of pregnancy. Obstet Gynecol 2003;101:469–79. 23. Thornton YS, Smarkola C, Kopacz SM, Ishoof SB. Perinatal outcomes in nutritionally monitored obese pregnant women: a randomized clinical trial. J Natl Med Assoc 2009;101:569–77. 24. Van Buul BJA, Steegers EAP, Van Der Maten GD, Delemarre FMC, Jongsma HW, Oosterbaan HP, De Jong PA. Dietary sodium restriction does not prevent gestational hypertension: a Dutch two-center randomized trial. Hypertens Pregnancy 1997;16:335–46 25. Vinter CA, Jensen DM, Ovesen P, Beck-Nielsen H, Jørgensen JS. The LiP (Lifestyle in Pregnancy) study: a randomized controlled trial of lifestyle intervention in 360 obese pregnant women. Diabetes Care 2011;34:2502–7. 11. Asbee SM, Jenkins TR, Butler JR, White J, Elliot M, Rutledge A. Preventing excessive weight gain during pregnancy through dietary and lifestyle counseling: a randomized controlled trial. Obstet Gynecol 2009;113:305–12. 12. Bonomo M, Corica D, Mion E, Goncalves D, Motta G, Merati R, Ragusa A, Morabito A. Evaluating the therapeutic approach in pregnancies complicated by borderline glucose intolerance: a randomized clinical trial. Diabet Med 2005;22:1536–41. 26. Wolff S, Legarth J, Vangsgaard K, Toubro S, Astrup A. A randomized trial of the effects of dietary counseling on gestational weight gain and glucose metabolism in obese pregnant women. Int J Obes (Lond) 2008;32:495– 501. |
| Gresham et al. 2016 | 1. Asbee S.M., Jenkins T.R., Butler J.R.,White J., Elliot M. & Rutledge A. (2009) Preventing excessive weight gain during pregnancy through dietary and lifestyle counseling: a randomized controlled trial. Obstetrics and Gynecology 113, 305–312. 2. Bonomo M., Corica D., Mion E., Goncalves D., Motta G., Merati R. et al. (2005) Evaluating the therapeutic approach in pregnancies complicated by borderline glucose intolerance: a randomized clinical trial. Diabetic Medicine 22, 1536–1541. 3. Briley C., Flanagan N.L. & Lewis N. (2002) In-home prenatal nutrition intervention increased dietary iron intakes and reduced low birthweight in low-income African-American women. Journal of the American Dietetic Association 102 (7), 984–987. 4. Chan G.M., McElligott K., McNaught T. & Gill G. (2006) Effects of dietary calcium intervention on adolescent mothers and newborns: a randomized controlled trial. Obstetrics and Gynecology 108, 565–571. 5. Kafatos A., Vlachonikolis I. & Codrington C. (1989) Nutrition during pregnancy: the effects of an educational intervention program in Greece. The American Journal of Clinical Nutrition 50, 970–979. 6. Khoury J., Henriksen T., Christophersen B. & Tonstad S. (2005) Effect of a cholesterol-lowering diet on maternal, cord, and neonatal lipids, and pregnancy outcome: a randomized clinical trial. American Journal of Obstetrics and Gynecology 193, 1292–1301. 7. Knuist M., Bonsel G.J., Zondervan H.A. & Treffers P.E. (1998) Low sodium diet and pregnancy-induced hypertension: a multi-centre randomised controlled trial. British Journal of Obstetrics and Gynaecology 105, 430–434. 8. Luoto R., Kinnunen T.I., Aittasalo M., Kolu P., Raitanen J., Ojala K. et al. (2011) Primary prevention of gestational diabetes mellitus and large-for-gestational-age newborns by lifestyle counseling: a cluster-randomized controlled trial. PLoS (Public Library of Science) Medicine 8, e1001036′. 9. O’Connor M.J. & Whaley S.E. (2007) Brief intervention for alcohol use by pregnant women. American Journal of Public Health 97, 252–258. 10. Quinlivan J.A., Lam L.T. & Fisher J. (2011) A randomised trial of a four-step multidisciplinary approach to the antenatal care of obese pregnant women. Australian & New Zealand Journal of Obstetrics & Gynaecology 51, 141–146. 11. Thornton Y.S., Smarkola C., Kopacz S.M. & Ishoof S.B. (2009) Perinatal outcomes in nutritionally monitored obese pregnant women: a randomized clinical trial. Journal of the National Medical Association 101, 569– 577. 12. Van Buul B.J.A., Steegers E.A.P., Van Der Maten G.D., Delemarre F.M.C., Jongsma H.W., Oosterbaan H.P. et al. (1997) Dietary sodium restriction does not prevent gestational hypertension: a Dutch two-center randomized trial. Hypertension in Pregnancy 16, 335–346. 13. Vinter C.A., Jensen D.M., Ovesen P., Beck-Nielsen H. & Jørgensen J.S. (2011) The LiP (Lifestyle in Pregnancy) study: a randomized controlled trial of lifestyle intervention in 360 obese pregnant women. Diabetes Care 34, 2502–2507. 14. Wolff S., Legarth J., Vangsgaard K., Toubro S. & Astrup A. (2008) A randomized trial of the effects of dietary counselling on gestational weight gain and glucose metabolism in obese pregnant women. International Journal of Obesity 32, 495–501. |
| Guillemette et al. 2018 | 1. Avery MD, Leon AS, Kopher RA. Effects of a partially home-based exercise program for women with gestational diabetes. Obstet Gynecol. 1997;89(1):10-5. 2. Baciu k EP, Pereira RI, Cecatti JG, Braga AF, Cavalcante SR. Water aerobics in pregnancy: cardiovascular response, labor and neonatal outcomes. Reprod Health. 2008; https://doi.org/10.1186/1742-4755-5-10. 3. Barakat R, Lucia A, Ruiz JR. Resistance exercise training during pregnancy and newborn's birth size: a randomised controlled trial. International journal of obesity. 2005) 2009; https://doi.org/10.1038/ijo.2009.150. 4. Barakat R, Pelaez M, Cordero Y, Perales M, Lopez C, Coteron J, et al. Exercise during pregnancy protects against hypertension and macrosomia: randomized clinical trial. Am J Obstet Gynecol. 2016; https://doi.org/10. 1016/j.ajog.2015.11.039. 5. Barakat R, Pelaez M, Lopez C, Montejo R, Coteron J. Exercise during pregnancy reduces the rate of cesarean and instrumental deliveries: results of a randomized controlled trial. Journal of maternal-fetal & neonatal medicine. 2013; https://doi.org/10.3109/14767058.2012.696165. 6. Barakat R, Perales M, Bacchi M, Coteron J, Refoyo I. A program of exercise throughout pregnancy. Is it safe to mother and newborn? American journal of health promotion: AJHP. 2014;29(1):2–8. 7. Clapp JF, Kim H, Burciu B, Lopez B. Beginning regular exercise in early pregnancy: effect on fetoplacental growth. Am J Obstet Gynecol. 2000; https://doi.org/10.1067/mob.2000.107096. 7. Cordero FY, Mottola FM, Vargas FJ, Blanco FM, Barakat FR. Exercise is associated with a reduction in gestational diabetes mellitus. Med Sci Sports Exerc. 2015;47(7):1328–33. https://doi.org/10.1249/MSS.0000000000000547. 8. Daly N, Farren M, McKeating A, O'Higgins A, Mullaney L, Turner MJ. Effect of a randomized controlled trial of an intensive medically supervised exercise program designed to improve maternal glucose control on gestational weight gain. Am J Obstet Gynecol. 2017;216(1 Supplement 1):S24. 9. de Barros MC, Lopes MA, Francisco RP, Sapienza AD, Zugaib M. Resistance exercise and glycemic control in women with gestational diabetes mellitus. Am J Obstet Gynecol. 2010;203:556–6. e1–6 10. De Oliveria Melo AS, Silva JLP, Tavares JS, Barros VO, Leite DFB, Amorim MMR. Effect of a physical exercise program during pregnancy on uteroplacental and fetal blood flow and fetal growth: a randomized controlled trial. Obstet Gynecol. 2012;120(2 PART 1):302–10. 11. Erkkola R, Mäkelä M. Heart volume and physical fitness of parturients. Ann Clin Res. 1976;8(1):15–21. 12. Garnaes KK, Nyrnes SA, Salvesen KA, Salvesen O, Morkved S, Moholdt T. Effect of supervised exercise training during pregnancy on neonatal and maternal outcomes among overweight and obese women. Secondary analyses of the ETIP trial: a randomised controlled trial. PLoS One. 2017;12(3) (no pagination):e0173937. 13. Garshasbi A, Faghih Zadeh S. The effect of exercise on the intensity of low back pain in pregnant women. Int J Gynecol Obstet. 2005;88(3):271–5. 14. Ghodsi Z, Asltoghiri M. Effects of aerobic exercise training on maternal and neonatal outcome: a randomized controlled trial on pregnant women in Iran. JPMA The Journal of the Pakistan Medical Association. 2014;64(9):1053–6. 15. Guelfi KJ, Ong MJ, Crisp NA, Fournier PA, Wallman KE, Grove JR, et al. Regular exercise to prevent the recurrence of gestational diabetes mellitus: a randomized controlled trial. Obstet Gynecol. 2016;128(4):819–27. https:// doi.org/10.1097/aog.0000000000001632. 16. Haakstad LA, Bo K. Exercise in pregnant women and birth weight: a randomized controlled trial. BMC Pregnancy & Childbirth. 2011;11:66. 17. Hellenes OM, Vik T, Løhaugen GC, Salvesen K, Stafne SN, Mørkved S, et al. Regular moderate exercise during pregnancy does not have an adverse effect on the neurodevelopment of the child. Acta paediatrica (Oslo, Norway : 1992). 2015; https://doi.org/10.1111/apa.12890. 18. Hollingsworth DR, Moore TR. Postprandial walking exercise in pregnant insulin-dependent (type I) diabetic women: reduction of plasma lipid levels but absence of a significant effect on glycemic control. American Journal of Obstetrics & Gynecology. 1987;157(6):1359–63. 19. Hopkins SA, Baldi JC, Cutfield WS, McCowan L, Hofman PL. Exercise training in pregnancy reduces offspring size without changes in maternal insulin sensitivity. J Clin Endocrinol Metab. 2010; https://doi.org/10.1210/jc.2009-2255. 20. Kasawara KT, Burgos CS, do Nascimento SL, Ferreira NO, Surita FG, Pinto ESJL. Maternal and perinatal outcomes of exercise in pregnant women with chronic hypertension and/or previous preeclampsia: a randomized controlled trial. ISRN obstetrics and gynecology. 2013;2013:857047. https:// doi.org/10.1155/2013/857047. 21. Kihlstrand M, Stenman B, Nilsson S, Axelsson O. Water-gymnastics reduced the intensity of back/low back pain in pregnant women. Acta Obstet Gynecol Scand. 1999;78(3):180. https://doi.org/10.1080/j. 1600- 0412.1999.780302.x. 22. Kong KL, Campbell CG, Foster RC, Peterson AD, Lanningham-Foster L. A pilot walking program promotes moderate-intensity physical activity during pregnancy. Med Sci Sports Exerc. 2014;46(3):462–71. 23. Labonte-Lemoyne E, Curnier D, Ellemberg D. Exercise during pregnancy enhances cerebral maturation in the newborn: a randomized controlled trial. Journal of Clinical & Experimental Neuropsychology: official. J Int Neuropsychol Soc. 2017;39(4):347–54. 24. Marquez-Sterling S, Perry AC, Kaplan TA, Halberstein RA, Signorile JF. Physical and psychological changes with vigorous exercise in sedentary primigravidae. Med Sci Sports Exerc. 2000; 25. Murtezani A, Paçarada M, Ibraimi Z, Nevzati A, Abazi N. The impact of exercise during pregnancy on neonatal outcomes: a randomized controlled trial. J Sports Med Phys Fitness. 2014;54(6):802–8. 26. Nascimento SL, Surita FG, Parpinelli M, Siani S, Pinto e Silva JL. The effect of an antenatal physical exercise programme on maternal/perinatal outcomes and quality of life in overweight and obese pregnant women: a randomised clinical trial. BJOG: An International Journal of Obstetrics and Gynaecology. 2011;118(12):1455–63. 27. Oostdam N, Poppel MN, Wouters MG, Eekhoff EM, Bekedam DJ, Kuchenbecker WK, et al. No effect of the FitFor2 exercise programme on blood glucose, insulin sensitivity, and birthweight in pregnant women who were overweight and at risk for gestational diabetes: results of a randomised controlled trial. BJOG : an international journal of obstetrics and gynaecology. 2012; https://doi.org/10.1111/j.1471-0528.2012.03366.x. 28. Perales M, Calabria I, Lopez C, Franco E, Coteron J, Barakat R. Regular exercise throughout pregnancy is associated with a shorter first stage of labor. Am J Health Promot. 2017; https://doi.org/10.4278/ajhp.140221-QUAN-79. 29. Petrov Fieril K, Glantz A, Fagevik Olsen M. The efficacy of moderate-tovigorous resistance exercise during pregnancy: a randomized controlled trial. Acta Obstet Gynecol Scand. 2015;94(1):35–42. 30. Pinzon DC, Zamora K, Martinez JH, Florez-Lopez ME, de Plata AC, Mosquera M, et al. Type of delivery and gestational age is not affected by pregnant Latin-American women engaging in vigorous exercise: a secondary analysis of data from a controlled randomized trial. Revista de salud publica (Bogota, Colombia). 2012;14(5):731–43. 31. Price BB, Amini SB, Kappeler K. Exercise in pregnancy: effect on fitness and obstetric outcomes—a randomized trial. Medicine and science in sports and exercise. 2012; https://doi.org/10.1249/MSS.0b013e318267ad67. 32. Ramos JG, Bgeginski R, Opperman ML, Martins-Costa S, Delevatti R, Schuch R, et al. Effect of aerobic training in pregnant women diagnosed with gestational diabetes: a preliminary report. Pregnancy Hypertension.2015;5(1):105. 32. Ruiz JR, Perales M, Pelaez M, Lopez C, Lucia A, Barakat R. Supervised exercise-based intervention to prevent excessive gestational weight gain: a randomized controlled trial. Mayo Clin Proc. 2013;88(12):1388–97. https:// doi.org/10.1016/j.mayocp.2013.07.020. 33. Santos IA, Stein R, Fuchs SC, Duncan BB, Ribeiro JP, Kroeff LR, et al. Aerobic exercise and submaximal functional capacity in overweight pregnant women: a randomized trial. Obstet Gynecol. 2005;106(2):243–9. https://doi. org/10.1097/01.aog.0000171113.36624.86. 34. Seneviratne SN, Jiang Y, Derraik J, McCowan L, Parry GK, Biggs JB, et al. Effects of antenatal exercise in overweight and obese pregnant women on maternal and perinatal outcomes: a randomised controlled trial. BJOG : an international journal of obstetrics and gynaecology. 2016; https://doi.org/10. 1111/1471-0528.13738. 35. Sklempe Kokic I, Ivanisevic M, Biolo G, Simunic B, Kokic T, Pisot R. Combination of a structured aerobic and resistance exercise improves glycaemic control in pregnant women diagnosed with gestational diabetes mellitus. A randomised controlled trial. Women and birth. 2017; https://doi. org/10.1016/j.wombi.2017.10.004. 36. Ussher M, Lewis S, Aveyard P, Manyonda I, West R, Lewis B, et al. Physical activity for smoking cessation in pregnancy: randomised controlled trial. BMJ (Clinical research ed). 2015;350:h2145. 37. Wang C, Wei Y, Zhang X, Zhang Y, Xu Q, Sun Y, et al. A randomized clinical trial of exercise during pregnancy to prevent gestational diabetes mellitus and improve pregnancy outcome in overweight and obese pregnant women. Am J Obstet Gynecol. 2017;216(4):340–51. |
| Guo et al. 2019 | 1. Barakat R, Pelaez M, Cordero Y, Perales M, Lopez C, Coteron J, et al. Exercise during pregnancy protects against hypertension and macrosomia: randomized clinical trial. Am J Obstet Gynecol 2016;214:649.e1–8.. 2. Barakat R, Pelaez M, Lopez C, Lucia A, Ruiz JR. Exercise during pregnancy and gestational diabetes-related adverse effects: a randomised controlled trial. Br J Sport Med 2013;47:630–6. 29 Barakat R, Perales M, Bacchi M, Coteron J, Refoyo I. A program of exercise throughout pregnancy. is it safe to mother and newborn? Am J Health Promot 2014;29:2–8. 3. Bell RJ, Palma SM, Lumley JM. The effect of vigorous exercise during pregnancy on birth-weight. Aust N Z J Obstet Gynaecol. 1995;35(1):46–51. 4. Bisson M, Almeras N, Dufresne SS, Robitaille J, Rheaume C, Bujold E, et al. A 12-week exercise program for pregnant women with obesity to improve physical activity levels: an open randomised preliminary study. PLoS ONE 2015;10:e0137742. 5. Bisson M, Almeras N, Dufresne SS, Robitaille J, Rheaume C, Bujold E, et al. A 12-week exercise program for pregnant women with obesity to improve physical activity levels: An open randomised preliminary study. PLoS One. 2015;10(9):e0137742. 6. Bogaerts AF, Devlieger R, Nuyts E, Witters I, Gyselaers W, Van den Bergh BR. Effects of lifestyle intervention in obese pregnant women on gestational weight gain and mental health: a randomized controlled trial. Int J Obes (Lond) 2013;37:814–21. 7. Bruno R, Petrella E, Bertarini V, Pedrielli G, Neri I, Facchinetti F. Adherence to a lifestyle programme in overweight/obese pregnant women and effect on gestational diabetes mellitus: a randomized controlled trial. Matern Child Nutr 2016;13:e12333. 8. Callaway LK, Colditz PB, Byrne NM, Lingwood BE, Rowlands IJ, Foxcroft K, et al. Prevention of gestational diabetes: feasibility issues for an exercise intervention in obese pregnant women. Diabetes Care 2010;33:1457–9. 9. Cavalcante Sergio R, Cecatti Jose G, Pereira Rosa I, Baciuk Erica P, Bernardo Ana L, Silveira C. Water aerobics II: maternal body composition and perinatal outcomes after a program for low risk pregnant women. Reprod Health. 2009;6(1):1. 10. Collings C, Curet L, Mullin J. Maternal and fetal responses to a maternal aerobic exercise program. Am J Obstet Gynecol. 1983;145(6):702–7. 11. Dodd JM, Turnbull D, McPhee AJ, Deussen AR, Grivell RM, Yelland LN, et al. Antenatal lifestyle advice for women who are overweight or obese: LIMIT randomised trial. BMJ (Online) 2014;348:g1285. 12. Garnaes KK, Morkved S, Salvesen O, Moholdt T. Exercise training and weight gain in obese pregnant women: a Randomized Controlled Trial (ETIP Trial). PLoS Med 2016;13:e1002079. 13. Guelfi KJ, Ong MJ, Crisp NA, Fournier PA, Wallman KE, Grove JR, et al. Regular exercise to prevent the recurrence of gestational diabetes mellitus: a randomized controlled trial. Obstet Gynecol 2016;128:819–27. 14. Hall DC, Kaufmann DA. Effects of aerobic and strength conditioning on pregnancy outcomes. Am J Obstet Gynecol. 1987;157(5):1199–203. 15. Harrison CL, Lombard CB, Strauss BJ, Teede HJ. Optimizing healthy gestational weight gain in women at high risk of gestational diabetes: a randomized controlled trial. Obesity 2013;21:904–9. 16. Hegaard HK, Petersson K, Hedegaard M, Ottesen B, Dykes AK, Henriksen TB, et al. Sports and leisure-time physical activity in pregnancy and birth weight: a population-based study. Scand J Med Sci Sports. 2010;20(1):e96– 102. 17. Herring SJ, Cruice JF, Bennett GG, Rose MZ, Davey A, Foster GD. Preventing excessive gestational weight gain among African American women: a randomized clinical trial. Obesity (Silver Spring) 2016;24:30–6. 18. Hopkins SA, Baldi JC, Cutfield WS, McCowan L, Hofman PL. Exercise training in pregnancy reduces offspring size without changes in maternal insulin sensitivity. J Clin Endocrinol Metab. 2010;95(5):2080–8. 19. Hui A, Back L, Ludwig S, Gardiner P, Sevenhuysen G, Dean H, et al. Lifestyle intervention on diet and exercise reduced excessive gestational weight gain in pregnant women under a randomised controlled trial. BJOG 2011;119:70–7. 20. Hui AL, Back L, Ludwig S, Gardiner P, Sevenhuysen G, Dean HJ, et al. Effects of lifestyle intervention on dietary intake, physical activity level, and gestational weight gain in pregnant women with different pre-pregnancy Body Mass Index in a randomized control trial. BMC Pregnancy Childbirth 2014;14:331. 21. Jing W, Huang Y, Liu X, Luo B, Yang Y, Liao S. The effect of a personalized intervention on weight gain and physical activity among pregnant women in China. Int J Gynaecol Obstet 2015;129:138–41. 22. Ko CW, Napolitano PG, Lee SP, Schulte SD, Ciol MA, Beresford SA. Physical activity, maternal metabolic measures, and the incidence of gallbladder sludge or stones during pregnancy: a randomized trial. Am J Perinatol 2014;31:39–48. 23. Koivusalo SB, Ron€ o K, Klemetti MM, Roine RP, Lindstr € om J, Erkkola M, € et al. Gestational diabetes mellitus can be prevented by lifestyle intervention: The Finnish Gestational Diabetes Prevention Study (RADIEL): a randomized controlled trial. Diabetes Care 2016;39:24–30. 24. Kong KL, Campbell CG, Foster RC, Peterson AD, Lanningham-Foster L. A pilot walking program promotes moderate-intensity physical activity during pregnancy. Med Sci Sports Exerc 2014;46:462–71. 25. Kopacz SM, Ishoof SB. Perinatal outcomes in nutritionally monitored obese pregnant women: a randomized clinical trial. J Natl Med Assoc 2009;101:569–77. Vesco KK, Karanja N, King JC, Gillman MW, Leo MC, Perrin N, et al. Efficacy of a group-based dietary intervention for limiting gestational weight gain among obese women: a randomized trial. Obesity (Silver Spring) 2014;22:1989–96. 26. Korpi-Hyovalti E, Schwab U, Laaksonen DE, Linjama H, Heinonen S, Niskanen L. Effect of intensive counselling on the quality of dietary fats in pregnant women at high risk of gestational diabetes mellitus. Br J Nutr 2012;108:910–7. 27. Kuhrt K, Harmon M, Hezelgrave NL, Seed PT, Shennan AH. Is recreational running associated with earlier delivery and lower birth weight in women who continue to run during pregnancy? An international retrospective cohort study of running habits of 1293 female runners during pregnancy. BMJ Open Sport Exerc Med. 2018;4:e000296. 28. Luoto R, Kinnunen TI, Aittasalo M, Kolu P, Raitanen J, Ojala K, et al. Primary prevention of gestational diabetes mellitus and large-forgestational-age newborns by lifestyle counseling: a clusterrandomized controlled trial. PLoS Med 2011;8:e1001036. 29. Luoto R, Laitinen K, Nermes M, Isolauri E. Impact of maternal probiotic-supplemented dietary counselling on pregnancy outcome and prenatal and postnatal growth: a double-blind, placebocontrolled study. Br J Nutr 2010;103:1792–9. 30. Magann EF, Evans SF, Weitz B, Newnham J. Antepartum, intrapartum, and neonatal significance of exercise on healthy low-risk pregnant working women. Obstet Gynecol. 2002;99(3):466–72. 31. Markovic TP, Muirhead R, Overs S, Ross GP, Louie JC, Kizirian N, et al. Randomized controlled trial investigating the effects of a low-glycemic index diet on pregnancy outcomes in women at high risk of gestational diabetesmellitus:theGIBaby3Study.DiabCare2016;39:31–8. 32. McCarthy EA, Walker SP, Ugoni A, Lappas M, Leong O, Shub A. Selfweighing and simple dietary advice for overweight and obese pregnant women to reduce obstetric complications without impact on quality of life: a randomised controlled trial. BJOG 2016;123:965–73. 33. McCowan LME, Roberts CT, Dekker GA, Taylor RS, Chan EHY, Kenny LC, et al. Risk factors for small-for-gestational-age infants by customised birthweight centiles: data from an international prospective cohort study. BJOG. 2010; 117(13):1599–607. 34. Moses RG, Casey SA, Quinn EG, Cleary JM, Tapsell LC, Milosavljevic M, et al. Pregnancy and Glycemic Index Outcomes study: effects of low glycemic index compared with conventional dietary advice on selected pregnancy outcomes. Am J Clin Nutr 2014;99:517–23. 35. Nobles C, Marcus BH, Stanek EJ 3rd, Braun B, Whitcomb BW, Solomon CG, et al. Effect of an exercise intervention on gestational diabetes mellitus: a randomized controlled trial. Obstet Gynecol 2015;125:1195–204. 36. Oostdam N, Van Poppel MNM, Wouters MGAJ, Eekhoff EMW, Bekedam DJ, Kuchenbecker WKH, et al. No effect of the FitFor2 exercise programme on blood glucose, insulin sensitivity, and birthweight in pregnant women who were overweight and at risk for gestational diabetes: results of a randomised controlled trial. BJOG 2012;119:1098–107. 37. Petrella E, Malavolti M, Bertarin V, Pignatti L, Neri I, Battistini NC, et al. Gestational weight gain in overweight and obese women enrolled in a healthy lifestyle and eating habits program. J Matern Fetal Neonata Med 2013;27:1348–52. 38. Phelan S, Phipps MG, Abrams B, Darroch F, Schaffner A, Wing RR. Randomized trial of a behavioral intervention to prevent excessivegestational weight gain: the Fit for Delivery Study. Am J Clin Nutr 2011;93:772–9. 39. Polley BA, Wing RR, Sims CJ. Randomized controlled trial to prevent excessive weight gain in pregnant women. Int J Obesity 2002;26:1494–502. 40. Poston L, Bell R, Croker H, Flynn AC, Godfrey KM, Goff L, et al. Effect of a behavioural intervention in obese pregnant women (the UPBEAT study): a multicentre, randomised controlled trial. Lancet Diabetes Endo 2015;3:767–77. 41. Price BB, Amini SB, Kappeler K. Exercise in pregnancy: effect on fitness and obstetric outcomes – A randomized trial. Med Sci Sport Exer 2012;44:2263–9. 38 Rakhshani A, Nagarathna R, Mhaskar R, Mhaskar A, Thomas A, Gunasheela S. The effects of yoga in prevention of pregnancy complications in high-risk pregnancies: a randomized controlled trial. Prev Med 2012;55:333–40. 42. Quinlivan JA, Lam LT, Fisher J. A randomised trial of a four-step multidisciplinary approach to the antenatal care of obese pregnant women. Aust NZ J Obstet Gyn 2011;51:141–6. 23 Thornton YS, Smarkola C, 43. Rauh K, Gabriel E, Kerschbaum E, Schuster T, von Kries R, AmannGassner U, et al. Safety and efficacy of a lifestyle intervention for pregnant women to prevent excessive maternal weight gain: a clusterrandomized controlled trial. BMC Pregnancy Childb 2013;16:151. 44. Renault KM, Nørgaard K, Nilas L, Carlsen EM, Cortes D, Pryds O, et al. The Treatment of Obese Pregnant Women (TOP) study: a randomized controlled trial of the effect of physical activity intervention assessed by pedometer with or without dietary intervention in obese pregnant women. Am J Obstet Gynecol 2014;210:134.e1–9 45. Rose NC, Haddow JE, Palomaki GE, Knight GJ. Self-rated physical activity level during the second trimester and pregnancy outcome. Obstet Gynecol. 1991;78(6):1078–80. 46. Ruchat HS-M, Davenport MM, Giroux SI, Hillier FM, Batada FA, Sopper FM, et al. Nutrition and exercise reduce excessive weight gain in normal-weight pregnant women. Med Sci Sports Exerc. 2012a;44(8):1419–26. 47. Ruchat SM, Davenport MH, Hillier I, Batada A, Sopper J, Hammond M, et al. Walking program of low or vigorous intensity during pregnancy confers an aerobic benefit. Int J Sports Med. 2012b;33(8):661–6. 48. Ruiz JR, Perales M, Pelaez M, Lopez C, Lucia A, Barakat R. Supervised exercise-based intervention to prevent excessive gestational weight gain: a randomized controlled trial. Mayo Clin Proc 2013;88:1388–97. 49. Sagedal LR, Overby NC, Bere E, Torstveit MK, Lohne-Seiler H, Smastuen M, et al. Lifestyle intervention to limit gestational weight gain: the Norwegian Fit for Delivery randomised controlled trial. BJOG 2017;124:97–109. 50. Seneviratne SN, Jiang Y, Derraik J, McCowan L, Parry GK, Biggs JB, et al. Effects of antenatal exercise in overweight and obese pregnant women on maternal and perinatal outcomes: a randomised controlled trial. BJOG 2016;123:588–97. 51. Stafne SN, Salvesen KA, Romundstad PR, Eggebø TM, Carlsen SM, Mørkved S. Regular exercise during pregnancy to prevent gestational diabetes: a randomized controlled trial. Obstet Gynecol 2012;119:29–36. 43 Tomic V, Sporis G, Tomic J, Milanovic Z, Zigmundovac-Klaic D, Pantelic S. The effect of maternal exercise during pregnancy on abnormal fetal growth. Croat Med J 2013;54:362–8. 52. Sternfeld B, Quesenberry CP Jr, Eskenazi B, Newman LA. Exercise during pregnancy and pregnancy outcome. Med Sci Sports Exerc. 1995;27(5):634– 40. 53. Sun Y, Zhao H. The effectiveness of lifestyle intervention in early pregnancy to prevent gestational diabetes mellitus in Chinese overweight and obese women: a quasi-experimental study. Appl Nurs Res 2016;30:125–30. 54. Vinter CA, Jensen DM, Ovesen P, Beck-Nielsen H, Jørgensen JS. The LiP (Lifestyle in Pregnancy) study: a randomized controlled trial of lifestyle intervention in 360 obese pregnant women. Diab Care 2011;34:2502–7. 55. Walsh JM, McGowan CA, Mahony R, Foley ME, McAuliffe FM. Low glycaemic index diet in pregnancy to prevent macrosomia (ROLO study): randomised control trial. BMJ (Online) 2012;345: e5605. 56. Wang C, Wei Y, Zhang X, Zhang Y, Xu Q, Sun Y, et al. A randomized clinical trial of exercise during pregnancy to prevent gestational diabetes mellitus and improve pregnancy outcome in overweight and obese pregnant women. Am J Obstet Gynecol. 2017;216(4):340–51. 57. Wang C. Effect of regular exercise commenced in early pregnancy on the incidence of gestational diabetes mellitus in overweight and obese pregnant women – a randomized controlled trial. Diab Care 2016;39:e163–4. 58. Zeanah M, Schlosser SP. Adherence to ACOG guidelines on exercise during pregnancy: effect on pregnancy outcome. J Obstet Gynecol Neonatal Nurs. 1993;22(4):329–35. 59. Zhang YH. Comprehensive effect assessment of medical nutrition guidance during pregnancy towards the health of mothers and children. Clin Exp Obstet Gyn 2015;42:644–8. |
| Hanifi et al., 2023 | 1. Artal R, Catanzaro RB, Gavard JA, Mostello DJ, Friganza JC. A lifestyle intervention of weight-gain restriction: diet and exercise in obese women with gestational diabetes mellitus. Appl Physiol Nutr Metab. 2007 Jun;32(3):596–601. PMID: 17510701. doi: 10.1139/H07-024. 2. Dubois S, Dougherty C, Duquette MP, Hanley JA, Moutquin JM. Twin pregnancy: the impact of the Higgins Nutrition Intervention Program on maternal and neonatal outcomes. Am J Clin Nutr. 1991 Jun;53(6): 1397–1403. PMID: 2035467. doi: 10.1093/ajcn/53.6.1397. 3. Fassett MJ, Dhillon SHK, Williams TR. Effects on perinatal outcome of treating women with 1 elevated glucose tolerance test value. Am J Obstet Gynecol. 2007;196(6):597.e1–597.e4; discussion.e4. PMID: 17547912. doi: 10.1016/j.ajog.2007.03.017. 4. Gandhi M, Hosseinzadeh P, Salmanian B, Moaddab A, Golabbakhsh H, Clark SL, et al. Dietary consultation impact on weight gain in twin pregnancies. J Reprod Med. 2018;63(7–8):347–51. 5. Luke B, Brown MB, Misiunas R, Anderson E, Nugent C, van de Ven C, et al. Specialized prenatal care and maternal and infant outcomes in twin pregnancy. Am J Obstet Gynecol. 2003 Oct;189(4):934–938. PMID: 14586329. doi: 10.1067/S0002-9378(03)01054-8.. 6. Morris DL, Berenson AB, Lawson J, Wiemann CM. Comparison of adolescent pregnancy outcomes by prenatal care source. J Reprod Med. 1993;38(5):375–80. PMID: 8320675. 7. Peccei A, Blake-Lamb T, Rahilly D, Hatoum I, Bryant A. Intensive prenatal nutrition counseling in a community health setting a randomized controlled trial. Obstet Gynecol. 2017;130(2):423–432. PMID: 28697099. doi: 10.1097/AOG.0000000000002134. 29. 8. Phelan S, Phipps MG, Abrams B, Darroch F, Schaffner A, Wing RR. Randomized trial of a behavioral intervention to prevent excessive gestational weight gain: the Fit for Delivery Study. Am J Clin Nutr. 2011 Apr;93(4):772–779. PMID: 21310836. doi: 10.3945/ajcn.110.005306. 9. Reader D, Splett P, Gunderson EP. Impact of gestational diabetes mellitus nutrition practice guidelines implemented by registered dietitians on pregnancy outcomes. J Am Diet Assoc. 2006;106(9):1426–1433. PMID: 16963348. doi: 10.1016/j.jada.2006.06.009. 10. Ricketts SA, Murray EK, Schwalberg R. Reducing low birthweight by resolving risks: results from Colorado’s prenatal plus program. Am J Public Health. 2005;95(11):1952–1957. PMID: 16195530. doi: 10.2105/ AJPH.2004.047068.. 11. Seabrook JA, Smith A, Clark AF, Gilliland JA. Geospatial analyses of adverse birth outcomes in Southwestern Ontario: examining the impact of environmental factors. Environ Res. 2019 May;172:18–26. PMID: 30769185. doi: 10.1016/j.envres.2018.12.068. 12. Thornton YS, Smarkola C, Kopacz SM, Ishoof SB. Perinatal outcomes in nutritionally monitored obese pregnant women: a randomized clinical trial. J Natl Med Assoc. 2009 Jun;101(6):569–77. PMID: 19585925. 28. 13. Vesco KK, Karanja N, King JC, Gillman MW, Leo MC, Perrin N, et al. Efficacy of a group-based dietary intervention for limiting gestational weight gain among obese women: a randomized trial. Obesity (Silver Spring). 2014 Sep;22(9):1989–1996. PMID: 25164259. doi: 10.1002/oby. 20831. 14. Weiderman WC, Marcuz L, Platt LD. Gestational diabetes: a triage model of care for rural perinatal providers. Am J Obstet Gynecol. 1996;174(6):1719–1724. PMID: 8678132. doi: 10.1016/S0002-9378(96) 70202-8. 30. |
| Harrison*et al.* 2023 | 1. Jing W, Huang Y, Liu X, Luo B, Yang Y, Liao S. The effect of a personalized intervention on weight gain and physical activity among pregnant women in China. Int J Gynaecol Obstet. 2015;129(2):138-141. doi:10.1016/j.ijgo. 2014.11.014 2. Okesene-Gafa KAM, Li M, McKinlay CJD, et al. Effect of antenatal dietary interventions in maternal obesity on pregnancy weight-gain and birthweight: Healthy Mums and Babies (HUMBA) randomized trial. Am J Obstet Gynecol. 2019;221(2):152.e1-152.e13. doi:10.1016/j.ajog.2019.03.003 3. Kihlstrand M, Stenman B, Nilsson S, Axelsson O. Water-gymnastics reduced the intensity of back/low back pain in pregnant women. Acta Obstet Gynecol Scand. 1999;78(3):180-185. 4. Clapp JF III, Kim H, Burciu B, Lopez B. Beginning regular exercise in early pregnancy: effect on fetoplacental growth. Am J Obstet Gynecol. 2000;183(6):1484-1488. doi:10.1067/mob.2000.107096 5. Marquez-Sterling S, Perry AC, Kaplan TA, Halberstein RA, Signorile JF. Physical and psychological changes with vigorous exercise in sedentary primigravidae. Med Sci Sports Exerc. 2000;32(1):58-62. doi:10.1097/00005768- 200001000-00010 6. Bechtel-Blackwell DA. Computer-assisted self-interview and nutrition education in pregnant teens. Clin Nurs Res. 2002;11(4):450-462. doi:10.1177/105477302237456 7. Prevedel TTS, Calderon IdMP, De Conti MH, Consonni EB, Rudge MVC. Maternal and perinatal effects of hydrotherapy in pregnancy. Repercussões maternas e perinatais da hidroterapia na gravidez. Rev Bras Ginecol Obstet. 2003;25:53-59. doi:10.1590/S0100-7203200300010000 8. Garshasbi A, Faghih Zadeh S. The effect of exercise on the intensity of low back pain in pregnant women. Int J Gynaecol Obstet. 2005;88(3):271-275. doi:10.1016/j.ijgo.2004.12.001 9. Santos IA, Stein R, Fuchs SC, et al. Aerobic exercise and submaximal functional capacity in overweight pregnant women: a randomized trial. Obstet Gynecol. 2005;106(2):243-249. doi:10.1097/01.AOG.0000171113. 36624.86 10. Sedaghati P, Ziaee V, Ardjmand A. The effect of an ergometric training program on pregnants’ weight gain and low back pain. Gazz Med Ital. 2007;166(6):209-213. 11. Baciuk EP, Pereira RI, Cecatti JG, Braga AF, Cavalcante SR. Water aerobics in pregnancy: cardiovascular response, labor and neonatal outcomes. Reprod Health. 2008;5:10. doi:10.1186/1742-4755-5-10 12. Barakat R, Stirling JR, Lucia A. Does exercise training during pregnancy affect gestational age: a randomised controlled trial. Br J Sports Med. 2008;42(8):674-678. doi:10.1136/bjsm.2008.047837 13. Guelinckx I, Devlieger R, Mullie P, Vansant G. Effect of lifestyle intervention on dietary habits, physical activity, and gestational weight gain in obese pregnant women: a randomized controlled trial. Am J Clin Nutr. 2010;91(2): 373-380. doi:10.3945/ajcn.2009.28166 14. Khaledan A, Mirdar S, Motahari-Tabari N, Ahmad SM. Effect of an Aerobic Exercise Program on Fetal Growth in Pregnant Women. Hayat. 2010;16(1):55-64. 15. Barakat R, Pelaez M, Montejo R, Luaces M, Zakynthinaki M. Exercise during pregnancy improves maternal health perception: a randomized controlled trial. Am J Obstet Gynecol. 2011;204(5):402.e1-402.e7. doi:10.1016/j. Ajog.2011.01.043 16. Haakstad LA, Bø K. Effect of regular exercise on prevention of excessive weight gain in pregnancy: a randomised controlled trial. Eur J Contracept Reprod Health Care. 2011;16(2):116-125. doi:10.3109/13625187.2011. 560307 17. Nascimento SL, Surita FG, Parpinelli MÂ, Siani S, Pinto e Silva JL. The effect of an antenatal physical exercise programme on maternal/perinatal outcomes and quality of life in overweight and obese pregnant women: a randomised clinical trial. BJOG. 2011;118(12):1455-1463. doi:10.1111/j.1471-0528.2011.03084.x 18. Barakat R, Cordero Y, Coteron J, Luaces M, Montejo R. Exercise during pregnancy improves maternal glucose screen at 24-28 weeks: a randomised controlled trial. Br J Sports Med. 2012;46(9):656-661. doi:10.1136/bjsports2011-090009 19. Barakat R, Pelaez M, Lopez C, Montejo R, Coteron J. Exercise during pregnancy reduces the rate of cesarean and instrumental deliveries: results of a randomized controlled trial. J Matern Fetal Neonatal Med. 2012;25(11): 2372-2376. doi:10.3109/14767058.2012.696165 20. Hui A, Back L, Ludwig S, et al. Lifestyle intervention on diet and exercise reduced excessive gestational weight gain in pregnant women under a randomised controlled trial. BJOG. 2012;119(1):70-77. doi:10.1111/j.1471-0528. 2011.03184.x 21. Oostdam N, van Poppel MN, Wouters MG, et al. No effect of the FitFor2 exercise programme on blood glucose, insulin sensitivity, and birthweight in pregnant women who were overweight and at risk for gestational diabetes: results of a randomised controlled trial. BJOG. 2012;119(9):1098-1107. doi:10.1111/j.1471-0528.2012.03366.x 22. Price BB, Amini SB, Kappeler K. Exercise in pregnancy: effect on fitness and obstetric outcomes-a randomized trial. Med Sci Sports Exerc. 2012;44(12):2263-2269. doi:10.1249/MSS.0b013e318267ad67 23. Walsh JM, McGowan CA, Mahony R, Foley ME, McAuliffe FM. Low glycaemic index diet in pregnancy to prevent macrosomia (ROLO study): randomised control trial. BMJ. 2012;345:e5605. doi:10.1136/bmj.e5605 24. Barakat R, Pelaez M, Lopez C, Lucia A, Ruiz JR. Exercise during pregnancy and gestational diabetes-related adverse effects: a randomised controlled trial. Br J Sports Med. 2013;47(10):630-636. doi:10.1136/bjsports-2012- 091788 25. Bogaerts AF, Devlieger R, Nuyts E, Witters I, Gyselaers W, Van den Bergh BR. Effects of lifestyle intervention in obese pregnant women on gestational weight gain and mental health: a randomized controlled trial. Int J Obes (Lond). 2013;37(6):814-821. doi:10.1038/ijo.2012.162 26. Ruiz JR, Perales M, Pelaez M, Lopez C, Lucia A, Barakat R. Supervised exercise-based intervention to prevent excessive gestational weight gain: a randomized controlled trial. Mayo Clin Proc. 2013;88(12):1388-1397. doi:10. 1016/j.mayocp.2013.07.02 27. Barakat R, Perales M, Bacchi M, Coteron J, Refoyo I. A program of exercise throughout pregnancy: is it safe to mother and newborn? Am J Health Promot. 2014;29(1):2-8. doi:10.4278/ajhp.130131-QUAN-56 28. Hui AL, Back L, Ludwig S, et al. Effects of lifestyle intervention on dietary intake, physical activity level, and gestational weight gain in pregnant women with different pre-pregnancy body mass index in a randomized control trial. BMC Pregnancy Childbirth. 2014;14:331. doi:10.1186/1471-2393-14-331 29. Ko CW, Napolitano PG, Lee SP, Schulte SD, Ciol MA, Beresford SA. Physical activity, maternal metabolic measures, and the incidence of gallbladder sludge or stones during pregnancy: a randomized trial. Am J Perinatol. 2014;31(1):39-48. doi:10.1055/s-0033-1334455 30. Kong KL, Campbell CG, Foster RC, Peterson AD, Lanningham-Foster L. A pilot walking program promotes moderate-intensity physical activity during pregnancy. Med Sci Sports Exerc. 2014;46(3):462-471. doi:10.1249/ MSS.0000000000000141 31. Vesco KK, Karanja N, King JC, et al. Efficacy of a group-based dietary intervention for limiting gestational weight gain among obese women: a randomized trial. Obesity (Silver Spring). 2014;22(9):1989-1996. doi:10.1002/ oby.20831 32. Gesell SB, Katula JA, Strickland C, Vitolins MZ. Feasibility and initial efficacy evaluation of a community-based cognitive-behavioral lifestyle intervention to prevent excessive weight gain during pregnancy in Latina women. Matern Child Health J. 2015;19(8):1842-1852. doi:10.1007/s10995-015-1698-x 33. Perales M, Refoyo I, Coteron J, Bacchi M, Barakat R. Exercise during pregnancy attenuates prenatal depression: a randomized controlled trial. Eval Health Prof. 2015;38(1):59-72. doi:10.1177/0163278714533566 34. Poston L, Bell R, Croker H, et al; UPBEAT Trial Consortium. Effect of a behavioural intervention in obese pregnant women (the UPBEAT study): a multicentre, randomised controlled trial. Lancet Diabetes Endocrinol. 2015;3(10):767-777. doi:10.1016/S2213-8587(15)00227-2 35. Barakat R, Pelaez M, Cordero Y, et al. Exercise during pregnancy protects against hypertension and macrosomia: randomized clinical trial. Am J Obstet Gynecol. 2016;214(5):649.e1-649.e8. doi:10.1016/j.ajog.2015. 11.039 36. Garnæs KK, Mørkved S, Salvesen Ø, Moholdt T. Exercise training and weight gain in obese pregnant women: a randomized controlled trial (ETIP trial). PLoS Med. 2016;13(7):e1002079. doi:10.1371/journal.pmed.100207 37. Koivusalo SB, Rönö K, Klemetti MM, et al. Gestational diabetes mellitus can be prevented by lifestyle intervention: the Finnish gestational diabetes prevention study (RADIEL): a randomized controlled trial. Diabetes Care. 2016;39(1):24-30. doi:10.2337/dc15-0511 38. Perales M, Calabria I, Lopez C, Franco E, Coteron J, Barakat R. Regular exercise throughout pregnancy is associated with a shorter first stage of labor. Am J Health Promot. 2016;30(3):149-154. doi:10.4278/ajhp.140221- QUAN-79 39. Wang C, Wei Y, Zhang X, et al. Effect of regular exercise commenced in early pregnancy on the incidence of gestational diabetes mellitus in overweight and obese pregnant women: a randomized controlled trial. Diabetes Care. 2016;39(10):e163-e164. doi:10.2337/dc16-1320 40. Assaf-Balut C, García de la Torre N, Durán A, et al. A Mediterranean diet with additional extra virgin olive oil and pistachios reduces the incidence of gestational diabetes mellitus (GDM): a randomized controlled trial: the St. Carlos GDM prevention study. PLoS One. 2017;12(10):e0185873. doi:10.1371/journal.pone.0185873 41. da Silva SG, Hallal PC, Domingues MR, et al. A randomized controlled trial of exercise during pregnancy on maternal and neonatal outcomes: results from the PAMELA study. Int J Behav Nutr Phys Act. 2017;14(1):175. doi:10. 1186/s12966-017-0632-6 42. Daly N, Farren M, McKeating A, O’Higgins A, Mullaney L, Turner MJ. 34: Effect of a randomized controlled trial of an intensive medically supervised exercise program designed to improve maternal glucose control on gestational weight gain. Am J Obstet Gynecol. 2017;216(1)(suppl):S24. doi:10.1016/j.ajog.2016.11.926 43. Peaceman AM, Kwasny MJ, Gernhofer N, Vincent E, Josefson JL, Van Horn L. 2: MOMFIT: a randomized clinical trial of an intervention to prevent excess gestational weight gain in overweight and obese women. Am J Obstet Gynecol. 2017;216(1)(suppl):S2-S3. doi:10.1016/j.ajog.2016.11.003 44. Sagedal LR, Øverby NC, Bere E, et al. Lifestyle intervention to limit gestational weight gain: the Norwegian Fit for Delivery randomised controlled trial. BJOG. 2017;124(1):97-109. doi:10.1111/1471-0528.13862 45. Bacchi M, Mottola MF, Perales M, Refoyo I, Barakat R. Aquatic activities during pregnancy prevent excessive maternal weight gain and preserve birth weight: a randomized clinical trial. Am J Health Promot. 2018;32(3): 729-735. doi:10.1177/0890117117697520 46. Barakat R, Franco E, Perales M, López C, Mottola MF. Exercise during pregnancy is associated with a shorter duration of labor. a randomized clinical trial. Eur J Obstet Gynecol Reprod Biol. 2018;224:33-40. doi:10.1016/j. Ejogrb.2018.03.009 47. Kennelly MA, Ainscough K, Lindsay KL, et al. Pregnancy exercise and nutrition with smartphone application support: a randomized controlled trial. Obstet Gynecol. 2018;131(5):818-826. doi:10.1097/AOG. 0000000000002582 48. Kiani Asiabar A, Amin Shokravi F, Hajifaraji M, Zayeri F. The effect of an educational intervention in early pregnancy with spouse’s participation on optimal gestational weight gain in pregnancy: a randomized controlled trial. Health Educ Res. 2018;33(6):535-547. doi:10.1093/her/cyy040 49. Anleu E, Reyes M, Araya B M, Flores M, Uauy R, Garmendia ML. Effectiveness of an intervention of dietary counseling for overweight and obese pregnant women in the consumption of sugars and energy. Nutrients. 2019; 11(2):385. doi:10.3390/nu11020385 50. Barakat R, Refoyo I, Coteron J, Franco E. Exercise during pregnancy has a preventative effect on excessive maternal weight gain and gestational diabetes. a randomized controlled trial. Braz J Phys Ther. 2019;23(2): 148-155. doi:10.1016/j.bjpt.2018.11.005 51. Brik M, Fernández-Buhigas I, Martin-Arias A, Vargas-Terrones M, Barakat R, Santacruz B. Does exercise during pregnancy impact on maternal weight gain and fetal cardiac function: a randomized controlled trial. Ultrasound Obstet Gynecol. 2019;53(5):583-589. doi:10.1002/uog.20147 52. Pelaez M, Gonzalez-Cerron S, Montejo R, Barakat R. Protective effect of exercise in pregnant women including those who exceed weight gain recommendations: a randomized controlled trial. Mayo Clin Proc. 2019;94(10): 1951-1959. doi:10.1016/j.mayocp.2019.01.050 53. Rodríguez-Blanque R, Aguilar-Cordero MJ, Marín-Jiménez AE, Núñez-Negrillo AM, Sánchez-López AM, Sánchez-García JC. Influence of a water-based exercise program in the rate of spontaneous birth: a randomized clinical trial. Int J Environ Res Public Health. 2020;17(3):795. doi:10.3390/ijerph17030795 54. Briley C, Flanagan NL, Lewis N. In-home prenatal nutrition intervention increased dietary iron intakes and reduced low birthweight in low-income African-American women.J Am Diet Assoc. 2002;102(7):984-987. doi:10. 1016/S0002-8223(02)90225-7 55. Khoury J, Henriksen T, Christophersen B, Tonstad S. Effect of a cholesterol-lowering diet on maternal, cord, and neonatal lipids, and pregnancy outcome: a randomized clinical trial. Am J Obstet Gynecol. 2005;193(4): 1292-1301. doi:10.1016/j.ajog.2005.05.016 56. Wolff S, Legarth J, Vangsgaard K, Toubro S, Astrup A. A randomized trial of the effects of dietary counseling on gestational weight gain and glucose metabolism in obese pregnant women. Int J Obes (Lond). 2008;32(3): 495-501. doi:10.1038/sj.ijo.0803710 57. Asbee SM, Jenkins TR, Butler JR, White J, Elliot M, Rutledge A. Preventing excessive weight gain during pregnancy through dietary and lifestyle counseling: a randomized controlled trial. Obstet Gynecol. 2009;113(2 Pt 1):305-312. doi:10.1097/AOG.0b013e318195baef 58. Jeffries K, Shub A, Walker SP, Hiscock R, Permezel M. Reducing excessive weight gain in pregnancy: a randomised controlled trial. Med J Aust. 2009;191(8):429-433. doi:10.5694/j.1326-5377.2009.tb02877.x 59. Ong MJ, Guelfi KJ, Hunter T, Wallman KE, Fournier PA, Newnham JP. Supervised home-based exercise may attenuate the decline of glucose tolerance in obese pregnant women. Diabetes Metab. 2009;35(5):418-421. doi: 10.1016/j.diabet.2009.04.008 60. Thornton YS, Smarkola C, Kopacz SM, Ishoof SB. Perinatal outcomes in nutritionally monitored obese pregnant women: a randomized clinical trial.J Natl Med Assoc. 2009;101(6):569-577. doi:10.1016/S0027-9684 (15)30942-1 61. Hopkins SA, Baldi JC, Cutfield WS, McCowan L, Hofman PL. Exercise training in pregnancy reduces offspring size without changes in maternal insulin sensitivity.J Clin Endocrinol Metab. 2010;95(5):2080-2088. doi:10.1210/ jc.2009-2255 62. Huang TT, Yeh CY, Tsai YC. A diet and physical activity intervention for preventing weight retention among Taiwanese childbearing women: a randomised controlled trial. Midwifery. 2011;27(2):257-264. doi:10.1016/j.midw. 2009.06.009 63. Phelan S, Phipps MG, Abrams B, Darroch F, Schaffner A, Wing RR. Randomized trial of a behavioral intervention to prevent excessive gestational weight gain: the Fit for Delivery Study. Am J Clin Nutr. 2011;93(4): 772-779. doi:10.3945/ajcn.110.005306 64. Quinlivan JA, Lam LT, Fisher J. A randomised trial of a four-step multidisciplinary approach to the antenatal care of obese pregnant women. Aust N Z J Obstet Gynaecol. 2011;51(2):141-146. doi:10.1111/j.1479-828X.2010. 01268.x 65. Korpi-Hyövälti E, Schwab U, Laaksonen DE, Linjama H, Heinonen S, Niskanen L. Effect of intensive counselling on the quality of dietary fats in pregnant women at high risk of gestational diabetes mellitus. Br J Nutr. 2012;108 (5):910-917. doi:10.1017/S0007114511006118 66. Althuizen E, van der Wijden CL, van Mechelen W, Seidell JC, van Poppel MN. The effect of a counselling intervention on weight changes during and after pregnancy: a randomised trial. BJOG. 2013;120(1):92-99. doi:10. 1111/1471-0528.12014 67. Deveer R, Deveer M, Akbaba E, et al. The effect of diet on pregnancy outcomes among pregnant with abnormal glucose challenge test. Eur Rev Med Pharmacol Sci. 2013;17(9):1258-1261. 68. Harrison CL, Lombard CB, Strauss BJ, Teede HJ. Optimizing healthy gestational weight gain in women at high risk of gestational diabetes: a randomized controlled trial. Obesity (Silver Spring). 2013;21(5):904-909. doi:10. 1002/oby.20163 69. Di Carlo C, Iannotti G, Sparice S, et al. The role of a personalized dietary intervention in managing gestational weight gain: a prospective, controlled study in a low-risk antenatal population. Arch Gynecol Obstet. 2014;289(4): 765-770. doi:10.1007/s00404-013-3054-y 70. Dodd JM, Turnbull D, McPhee AJ, et al; LIMIT Randomised Trial Group. Antenatal lifestyle advice for women who are overweight or obese: LIMIT randomised trial. BMJ. 2014;348:g1285. doi:10.1136/bmj.g1285 71. Petrella E, Malavolti M, Bertarini V, et al. Gestational weight gain in overweight and obese women enrolled in a healthy lifestyle and eating habits program.J Matern Fetal Neonatal Med. 2014;27(13):1348-1352. doi:10.3109/ 14767058.2013.858318 72. Bisson M, Alméras N, Dufresne SS, et al. A 12-week exercise program for pregnant women with obesity to improve physical activity levels: an open randomised preliminary study. PLoS One. 2015;10(9):e0137742. doi:10. 1371/journal.pone.0137742 73. Dekker Nitert M, Barrett HL, Denny KJ, McIntyre HD, Callaway LK; BAMBINO group. Exercise in pregnancy does not alter gestational weight gain, MCP-1 or leptin in obese women. Aust N Z J Obstet Gynaecol. 2015;55 (1):27-33. doi:10.1111/ajo.12300 74. Ronnberg AK, Ostlund I, Fadl H, Gottvall T, Nilsson K. Intervention during pregnancy to reduce excessive gestational weight gain—a randomised controlled trial. BJOG. 2015;122(4):537-544. doi:10.1111/1471-0528.13131 75. Aşcı Ö, Rathfisch G. Effect of lifestyle interventions of pregnant women on their dietary habits, lifestyle behaviors, and weight gain: a randomized controlled trial.J Health Popul Nutr. 2016;35:7. doi:10.1186/s41043-016-0044-2 76. McCarthy EA, Walker SP, Ugoni A, Lappas M, Leong O, Shub A. Self-weighing and simple dietary advice for overweight and obese pregnant women to reduce obstetric complications without impact on quality of life: a randomised controlled trial. BJOG. 2016;123(6):965-973. doi:10.1111/1471-0528.13919 77. Seneviratne SN, Jiang Y, Derraik J, et al. Effects of antenatal exercise in overweight and obese pregnant women on maternal and perinatal outcomes: a randomised controlled trial. BJOG. 2016;123(4):588-597. doi:10. 1111/1471-0528.13738 78. Bruno R, Petrella E, Bertarini V, Pedrielli G, Neri I, Facchinetti F. Adherence to a lifestyle programme in overweight/obese pregnant women and effect on gestational diabetes mellitus: a randomized controlled trial. Matern Child Nutr. 2017;13(3):e12333. doi:10.1111/mcn.12333 79. Sewell DA, Hammersley VS, Robertson A, et al. A pilot randomised controlled trial investigating a Mediterranean diet intervention in pregnant women for the primary prevention of allergic diseases in infants. J Hum Nutr Diet. 2017;30(5):604-614. doi:10.1111/jhn.12469 80. Cahill AG, Haire-Joshu D, Cade WT, et al. Weight control program and gestational weight gain in disadvantaged women with overweight or obesity: a randomized clinical trial. Obesity (Silver Spring). 2018;26(3): 485-491. doi:10.1002/oby.22070 81. Phelan S, Wing RR, Brannen A, et al. Randomized controlled clinical trial of behavioral lifestyle intervention with partial meal replacement to reduce excessive gestational weight gain. Am J Clin Nutr. 2018;107(2):183-194. doi:10.1093/ajcn/nqx043 82. H Al Wattar B, Dodds J, Placzek A, et al; ESTEEM study group. Mediterranean-style diet in pregnant women with metabolic risk factors (ESTEEM): a pragmatic multicentre randomised trial. PLoS Med. 2019;16(7):e1002857. doi:10.1371/journal.pmed.1002857 83. Buckingham-Schutt LM, Ellingson LD, Vazou S, Campbell CG. The Behavioral Wellness in Pregnancy study: a randomized controlled trial of a multi-component intervention to promote appropriate weight gain. Am J Clin Nutr. 2019;109(4):1071-1079. doi:10.1093/ajcn/nqy35 84. Clark E, Isler C, Strickland D, et al. Influence of aerobic exercise on maternal lipid levels and offspring morphometrics. Int J Obes (Lond). 2019;43(3):594-602. doi:10.1038/s41366-018-0258-z 85. Daley A, Jolly K, Jebb SA, et al. Effectiveness of a behavioural intervention involving regular weighing and feedback by community midwives within routine antenatal care to prevent excessive gestational weight gain: POPS2 randomised controlled trial. BMJ Open. 2019;9(9):e030174. doi:10.1136/bmjopen-2019-030174 86. Kunath J, Günther J, Rauh K, et al. Effects of a lifestyle intervention during pregnancy to prevent excessive gestational weight gain in routine care—the cluster-randomised GeliS trial. BMC Med. 2019;17(1):5. doi:10.1186/ s12916-018-1235-z 87. Arthur C, Di Corleto E, Ballard E, Kothari A. A randomized controlled trial of daily weighing in pregnancy to control gestational weight gain. BMC Pregnancy Childbirth. 2020;20(1):223. doi:10.1186/s12884-020-02884-1 88. Polley BA, Wing RR, Sims CJ. Randomized controlled trial to prevent excessive weight gain in pregnant women. Int J Obes Relat Metab Disord. 2002;26(11):1494-1502. doi:10.1038/sj.ijo.0802130 89. Jackson RA, Stotland NE, Caughey AB, Gerbert B. Improving diet and exercise in pregnancy with Video Doctor counseling: a randomized trial. Patient Educ Couns. 2011;83(2):203-209. doi:10.1016/j.pec.2010.05.019 90. Herring SJ, Cruice JF, Bennett GG, Rose MZ, Davey A, Foster GD. Preventing excessive gestational weight gain among African American women: a randomized clinical trial. Obesity (Silver Spring). 2016;24(1):30-36. doi:10. 1002/oby.21240 91. Smith K, Lanningham-Foster L, Welch A, Campbell C. Web-based behavioral intervention increases maternal exercise but does not prevent excessive gestational weight gain in previously sedentary women.J Phys Act Health. 2016;13(6):587-593. doi:10.1123/jpah.2015-0219 92. Sun Y, Zhao H. The effectiveness of lifestyle intervention in early pregnancy to prevent gestational diabetes mellitus in Chinese overweight and obese women: a quasi-experimental study. Appl Nurs Res. 2016;30:125-130. doi:10.1016/j.apnr.2015.10.006 93. Willcox JC, Wilkinson SA, Lappas M, et al. A mobile health intervention promoting healthy gestational weight gain for women entering pregnancy at a high body mass index: the txt4two pilot randomised controlled trial. BJOG. 2017;124(11):1718-1728. doi:10.1111/1471-0528.14552 94. Chan RS-M, Tam W-H, Ho IC-H, et al. Randomized trial examining effectiveness of lifestyle intervention in reducing gestational diabetes in high risk Chinese pregnant women in Hong Kong. Sci Rep. 2018;8(1):13849. doi: 10.1038/s41598-018-32285-6 95. Olson CM, Groth SW, Graham ML, Reschke JE, Strawderman MS, Fernandez ID. The effectiveness of an online intervention in preventing excessive gestational weight gain: the e-moms roc randomized controlled trial. BMC Pregnancy Childbirth. 2018;18(1):148. doi:10.1186/s12884-018-1767-4 96. Ferrara A, Hedderson MM, Brown SD, et al. A telehealth lifestyle intervention to reduce excess gestational weight gain in pregnant women with overweight or obesity (GLOW): a randomised, parallel-group, controlled trial. Lancet Diabetes Endocrinol. 2020;8(6):490-500. doi:10.1016/S2213-8587(20)30107-8 97. Chao AM, Srinivas SK, Studt SK, Diewald LK, Sarwer DB, Allison KC. A pilot randomized controlled trial of a technology-based approach for preventing excess weight gain during pregnancy among women with overweight. Front Nutr. 2017;4:57. doi:10.3389/fnut.2017.00057 98. Hawkins M, Hosker M, Marcus BH, et al. A pregnancy lifestyle intervention to prevent gestational diabetes risk factors in overweight Hispanic women: a feasibility randomized controlled trial. Diabet Med. 2015;32(1):108-115. doi:10.1111/dme.12601 99. Simmons D, Devlieger R, van Assche A, et al. Effect of physical activity and/or healthy eating on GDM Risk: the DALI lifestyle study.J Clin Endocrinol Metab. 2017;102(3):903-913. doi:10.1210/jc.2016-3455 |
| He et al, 2023 | 1. Aguilar-Cordero, M.J., Sanchez-García, ´ J.C., Rodriguez-Blanque, R., Sanchez-L ´ opez, ´ A. M., Mur-Villar, N., 2019. Moderate physical activity in an aquatic environment during pregnancy (SWEP study) and its influence in preventing postpartum depression. J. Am. Psychiatr. Nurses Assoc. 25, 112–121. 2. Armstrong, K., Edwards, H., 2004. The effectiveness of a pram-walking exercise programme in reducing depressive symptomatology for postnatal women. Int. J. Nurs. Pract. 10, 177–194. 3. Coll, C.V.N., Domingues, M.R., Stein, A., da Silva, B.G.C., Bassani, D.G., Hartwig, F.P., da Silva, I.C.M., da Silveira, M.F., da Silva, S.G., Bertoldi, A.D., 2019. Efficacy of regular exercise during pregnancy on the prevention of postpartum depression: the PAMELA randomized clinical trial. JAMA Netw. Open 2, e186861. Services, U.D.o.H.a.H., 2018. Physical Activity Guidelines for Americans, 2nd edition. US Department of Health and Human Services, Washington 4. Daley, A.J., Blamey, R.V., Jolly, K., Roalfe, A.K., Turner, K.M., Coleman, S., McGuinness, M., Jones, I., Sharp, D.J., MacArthur, C., 2015a. A pragmatic randomized controlled trial to evaluate the effectiveness of a facilitated exercise intervention as a treatment for postnatal depression: the PAM-PeRS trial. Psychol. Med. 45, 2413–2425. 5. Daley, A.J., Foster, L., Long, G., Palmer, C., Robinson, O., Walmsley, H., Ward, R., 2015b. The effectiveness of exercise for the prevention and treatment of antenatal depression: systematic review with meta-analysis. BJOG 122, 57–62. 6. Daley, A., Riaz, M., Lewis, S., Aveyard, P., Coleman, T., Manyonda, I., West, R., Lewis, B., Marcus, B., Taylor, A., Ibison, J., Kent, A., Ussher, M., 2018. Physical activity for antenatal and postnatal depression in women attempting to quit smoking: randomised controlled trial. BMC Pregnancy Childbirth 18, 156. 7. Davis, K., Goodman, S.H., Leiferman, J., Taylor, M., Dimidjian, S., 2015. A randomized controlled trial of yoga for pregnant women with symptoms of depression and anxiety. Complement. Ther. Clin. Pract. 21, 166–172. 8. El-Rafie, M.M., Khafagy, G.M., Gamal, M.G., 2016. Effect of aerobic exercise during pregnancy on antenatal depression. Int. J. Women's Health 8, 53–57. 9. Field, T., Diego, M., Delgado, J., Medina, L., 2013a. Tai chi/yoga reduces prenatal depression, anxiety and sleep disturbances. Complement. Ther. Clin. Pract. 19, 6–10. Field, T., Diego, M., Delgado, J., Medina, L., 2013b. Yoga and social support reduce prenatal depression, anxiety and cortisol. J. Bodyw. Mov. Ther. 17, 397–403. 10. Forsyth, J., Boath, E., Henshaw, C., Brown, H., 2017. Exercise as an adjunct treatment for postpartum depression for women living in an inner city-a pilot study. Health Care Women Int. 38, 635–639. 11. Gallagher, A., Kring, D., Whitley, T., 2020. Effects of yoga on anxiety and depression for high risk mothers on hospital bedrest. Complement. Ther. Clin. Pract. 38, 101079. 12. Haruna, M., Watanabe, E., Matsuzaki, M., Ota, E., Shiraishi, M., Murayama, R., Yoshida, M., Yeo, S., 2013. The effects of an exercise program on health-related quality of life in postpartum mothers: a randomized controlled trial. Health 05 (03), 8. 13. Heh, S.S., Huang, L.H., Ho, S.M., Fu, Y.Y., Wang, L.L., 2008. Effectiveness of an exercise support program in reducing the severity of postnatal depression in Taiwanese women. Birth (Berkeley, Calif.) 35, 60–65. Jarbou, N.S., Newell, K.A., 2022. Exercise and yoga during pregnancy and their impact on depression: a systematic literature review. Arch. Womens Ment. Health 25, 539–559. 14. Keller, C., Ainsworth, B., Records, K., Todd, M., Belyea, M., Vega-Lopez, ´ S., Permana, P., Coonrod, D., Nagle-Williams, A., 2014. A comparison of a social support physical activity intervention in weight management among post-partum latinas. BMC Public Health 14, 971. 15. Ko, Y.L., Yang, C.L., Chiang, L.C., 2008. Effects of postpartum exercise program on fatigue and depression during "doing-the-month" period. J. Nurs. Res. 16, 177–186. Kołomanska, ´ D., Zarawski, M., Mazur-Bialy, A., 2019. Physical activity and depressive disorders in pregnant women-asystematic review. Medicina (Kaunas) 55. 16. Lewis, B.A., Gjerdingen, D.K., Avery, M.D., Sirard, J.R., Guo, H., Schuver, K., Marcus, B. H., 2014. A randomized trial examining a physical activity intervention for the prevention of postpartum depression: the healthy mom trial. Ment. Health Phys. Act. 7, 42–49. 17. Liu, Y.H., Chang, C.F., Hung, H.M., Chen, C.H., 2021. Outcomes of a walking exercise intervention in postpartum women with disordered sleep. J. Obstet. Gynaecol. Res. 47, 1380–1387. 18. Low, D.M., Bentley, K.H., Ghosh, S.S., 2020. Automated assessment of psychiatric disorders using speech: a systematic review. Laryngoscope Investig. Otolaryngol. 5, 96–116. LS, R., 1977. The CES-D Scale: a self-report depression scale for research in the general population. Appl. Psychol. Meas. 1, 385–401. 19. Mitchell, J., Field, T., Diego, M., Bendell, D., Newton, R., Pelaez, M., 2012. Yoga reduces prenatal depression symptoms. Psychology 0 (09), 5. 20. Mohammadi, F., Malakooti, J., Babapoor, J., Mohammad-Alizadeh-Charandabi, S., 2015. The effect of a home-based exercise intervention on postnatal depression and fatigue: a randomized controlled trial. Int. J. Nurs. Pract. 21, 478–485. 21. Navas, A., Carrascosa, M.D.C., Artigues, C., Ortas, S., Portells, E., Soler, A., Yanez, ˜ A.M., Bennasar-Veny, M., Leiva, A., 2021. Effectiveness of moderate-intensity aerobic water exercise during pregnancy on quality of life and postpartum depression: a multi-center, randomized controlled trial. J. Clin. Med. 10. 22. Norman, E., Sherburn, M., Osborne, R.H., Galea, M.P., 2010. An exercise and education program improves well-being of new mothers: a randomized controlled trial. Phys. Ther. 90, 348–355. 23. Ozkan, ¨ S.A., Kücükkelepce, D.S., Korkmaz, B., Yılmaz, G., Bozkurt, M.A., 2020. The effectiveness of an exercise intervention in reducing the severity of postpartum depression: a randomized controlled trial. Perspect.Psychiatr.Care 56, 844–850. 24. Perales, M., Santos-Lozano, A., Sanchis-Gomar, F., Luaces, M., Pareja-Galeano, H., Garatachea, N., Barakat, R., Lucia, A., 2016. Maternal cardiac adaptations to a physical exercise program during pregnancy. Med. Sci. Sports Exerc. 48, 896–906. 25. Perales, I. Refoyo, J. Coteron, M. Bacchi, R. Barakat. Exercise during pregnancy attenuates prenatal depression: a randomized controlled trial Eval. Health Prof., 38 (2015), pp. 59-72 26. Robledo-Colonia, A.F., Sandoval-Restrepo, N., Mosquera-Valderrama, Y.F., EscobarHurtado, C., Ramírez-V´elez, R., 2012. Aerobic exercise training during pregnancy reduces depressive symptoms in nulliparous women: a randomised trial. J. Physiother. 58, 9–15. 27. Rong, L., Wang, R., Ouyang, Y.Q., Redding, S.R., 2021. Efficacy of yoga on physiological and psychological discomforts and delivery outcomes in Chinese primiparas. Complement. Ther. Clin. Pract. 44, 101434. 28. Satyapriya, M., Nagarathna, R., Padmalatha, V., Nagendra, H.R., 2013. Effect of integrated yoga on anxiety, depression & well being in normal pregnancy. Complement. Ther. Clin. Pract. 19, 230–236. Silva, D.A.S., 2021. Longer leisure walking time is associated with positive self-rated health among adults and older adults: a Brazilian nationwide study. PeerJ 9, e11471. 29. Songøygard, K.M., Stafne, S.N., Evensen, K.A., Salvesen, K., Vik, T., Mørkved, S., 2012. Does exercise during pregnancy prevent postnatal depression? A randomized controlled trial. Acta Obstet. Gynecol. Scand. 91, 62–67. 30. Teychenne, M., Abbott, G., Stephens, L.D., Opie, R.S., Olander, E.K., Brennan, L., van der Pligt, P., Apostolopoulos, M., Ball, K., 2021. Mums on the move: a pilot randomised controlled trial of a home-based physical activity intervention for mothers at risk of postnatal depression. Midwifery 93, 102898. 31. Uebelacker, L.A., Battle, C.L., Sutton, K.A., Magee, S.R., Miller, I.W., 2016. A pilot randomized controlled trial comparing prenatal yoga to perinatal health education for antenatal depression. Arch. Womens Ment. Health 19, 543–547. 32. Vargas-Terrones, M., Barakat, R., Santacruz, B., Fernandez-Buhigas, I., Mottola, M.F., 2019. Physical exercise programme during pregnancy decreases perinatal depression risk: a randomised controlled trial. Br. J. Sports Med. 53, 348–353. 33. Vargas-Terrones, M., Nagpal, T.S., Perales, M., Prapavessis, H., Mottola, M.F., Barakat, R., 2021. Physical activity and prenatal depression: going beyond statistical significance by assessing the impact of reliable and clinical significant change. Psychol. Med. 51, 688–693. |
| Hill et al. 2013 | 1. Asbee SM, Jenkins TR, Butler JR, White J, Elliot M, Rutledge A. Preventing excessive weight gain during pregnancy through dietary and lifestyle counseling. Obstet Gynecol 2009; 113: 305-12. 2. Bogaerts AFL, Devlieger R, Nuyts E, Witters I, Gyselaers W, Van den Bergh BRH. Effects of lifestyle intervention in obese pregnant women on gestational weight gain and mental health: a randomized controlled trial. Int J Obes 2012; 1-8. Advance Online Publication. doi: 10.1038/ijo.2012.162 3. Claesson IM, Sydsjo G, Brynhildsen J et al. Weight gain restriction for obese pregnant women: a case-control intervention study. Br J Obstet Gynaecol 2008; 115: 44-50. 4. Guelinckx I, Devlieger R, Mullie P, Vansant G. Effect of lifestyle intervention on dietary habits, physical activity, and gestational weight gain in obese pregnant women: a randomized controlled trial. Am J Clin Nutr 2010; 91: 373-80. 5. Haakstad LAH, Bø K. Effect of regular exercise on prevention of excessive weight gain in pregnancy: a randomised controlled trial. European Journal of Contraception & Reproductive Health Care 2011; 16: 116-25. 6. Huang T-T, Yeh C-Y, Tsai Y-C. A diet and physical activity intervention for preventing weight retention among Taiwanese childbearing women: a randomised controlled trial. Midwifery 2011; 27: 257-64. 7. Hui A, Back L, Ludwig S et al. Lifestyle intervention on diet and exercise reduced excessive gestational weight gain in pregnant women under a randomised controlled trial. Br J Obstet Gynaecol 2012; 119: 70-77. 8. Kinnunen TI, Pasanen M, Aittasalo M et al. Preventing excessive weight gain during pregnancy - a controlled trial in primary health care. Eur J Clin Nutr 2007; 61: 884-91. 9. Lindholm ES, Norman M, Kilander CP, Altman D. Weight control program for obese pregnant women. Acta Obstet Gynecol Scand 2010; 89: 840-43. 10. Mottola MF, Giroux I, Gratton R et al. Nutrition and exercise prevent excess weight gain in overweight pregnant women. Med Sci Sports Exerc 2010; 42: 265-72. 11. Nascimento SL, Surita FG, Parpinelli MÂ, Siani S, Pinto e Silva JL. The effect of an antenatal physical exercise programme on maternal/perinatal outcomes and quality of life in overweight and obese pregnant women: a randomised clinical trial. Br J Obstet Gynaecol 2011; 118: 1455-63. 12. Olson CM, Strawderman MS, Reed RG. Efficacy of an intervention to prevent excessive gestational weight gain. Am J Obstet Gynecol 2004; 191: 530-36. 13. Phelan S, Phipps MG, Abrams B, Darroch F, Schaffner A, Wing RR. Randomized trial of a behavioral intervention to prevent excessive gestational weight gain: the Fit for Delivery Study. Am J Clin Nutr 2011; 93: 772-79. 14. Polley BA, Wing RR, Sims CJ. Randomized controlled trial to prevent excessive weight gain in pregnant women. Int J Obes Relat Metab Disord 2002; 26: 1494. 15. Ruchat S-M, Davenport MH, Giroux I et al. Nutrition and exercise reduce excessive weight gain in normal-weight pregnant women. Med Sci Sports Exerc 2012; 44(8): 1419-26. 16. Shirazian T. Lifestyle modification program decreases pregnancy weight gain in obese women. Am J Perinatol 2010; 27: 411-14. 17. Vinter CA, Jensen DM, Ovesen PER, Beck-Nielsen H, Jørgensen JS. The LiP (Lifestyle in Pregnancy) Study. Diabetes Care 2011; 34: 2502-07. 18. Wolff S, Legarth J, Vangsgaard K, Toubro S, Astrup A. A randomized trial of the effects of dietary counseling on gestational weight gain and glucose metabolism in obese pregnant women. Int J Obes (Lond) 2008; 32: 495- 501. |
| Hussain et al. 2020 | 1. Choi J, Lee JH, Vittinghoff E, Fukuoka Y. mHealth physical activity intervention: a randomized pilot study in physically inactive pregnant women. Matern Child Health J 2016 May;20(5):1091-1101 [FREE Full text] [doi: 10.1007/s10995-015-1895-7] [Medline: 26649879] 2. Dodd JM, Louise J, Cramp C, Grivell RM, Moran LJ, Deussen AR. Evaluation of a smartphone nutrition and physical activity application to provide lifestyle advice to pregnant women: The SNAPP randomised trial. Matern Child Nutr 2018 Jan;14(1). [doi: 10.1111/mcn.12502] [Medline: 28836373] 3. Herring SJ, Cruice JF, Bennett GG, Rose MZ, Davey A, Foster GD. Preventing excessive gestational weight gain among African American women: a randomized clinical trial. Obesity (Silver Spring) 2016 Jan;24(1):30-36 [FREE Full text] [doi: 10.1002/oby.21240] [Medline: 26592857] 4. Huberty JL, Buman MP, Leiferman JA, Bushar J, Hekler EB, Adams MA. Dose and timing of text messages for increasing physical activity among pregnant women: a randomized controlled trial. Transl Behav Med 2017 Jun;7(2):212-223 [FREE Full text] [doi: 10.1007/s13142-016-0445-1] [Medline: 27800565] 5. Kennelly MA, Ainscough K, Lindsay KL, O Sullivan E, Gibney ER, McCarthy M, et al. Pregnancy exercise and nutrition with smartphone application support: a randomized controlled trial. Obstet Gynecol 2018 May;131(5):818-826. [doi: 10.1097/AOG.0000000000002582] [Medline: 29630009] 6. Pollak KI, Alexander SC, Bennett G, Lyna P, Coffman CJ, Bilheimer A, et al. Weight-related SMS texts promoting appropriate pregnancy weight gain: a pilot study. Patient Educ Couns 2014 Nov;97(2):256-260 [FREE Full text] [doi: 10.1016/j.pec.2014.07.030] [Medline: 25153313] 7. Redman LM, Gilmore LA, Breaux J, Thomas DM, Elkind-Hirsch K, Stewart T, et al. Effectiveness of SmartMoms, a novel eHealth intervention for management of gestational weight gain: randomized controlled pilot trial. JMIR Mhealth Uhealth 2017 Sep 13;5(9):e133 [FREE Full text] [doi: 10.2196/mhealth.8228] [Medline: 28903892] 8. Soltani H, Duxbury A, Arden M, Dearden A, Furness P, Garland C. Maternal obesity management using mobile technology: a feasibility study to evaluate a text messaging based complex intervention during pregnancy. J Obes 2015;2015:814830 [FREE Full text] [doi: 10.1155/2015/814830] [Medline: 25960889] 9. Willcox JC, Wilkinson SA, Lappas M, Ball K, Crawford D, McCarthy E, et al. A mobile health intervention promoting healthy gestational weight gain for women entering pregnancy at a high body mass index: the txt4two pilot randomised controlled trial. Br J Obstet Gynaecol 2017 Oct;124(11):1718-1728 [FREE Full text] [doi: 10.1111/1471-0528.14552] [Medline: 28220604] |
| James et al. 2020 | 1. Aittasalo M, Pasanen M, Fogelholm M, Kinnunen TI, Ojala K, Luoto R. Physical activity counseling in maternity and child health care – a controlled trial. BMC Womens Health 2008;14(August 8):14. 2. Aittasalo M, Raitanen J, Kinnunen TI, Ojala K, Kolu P, Luoto R. Is intensive counseling in maternity care feasible and effective in promoting physical activity among women at risk for gestational diabetes? Secondary analysis of a cluster randomized NELLI study in Finland. Int J Behav Nutr Phys Act 2012;5 (September 9):104. 3. Chan RS-M, Tam W-H, Ho IC-H, Kwan MW-C, Li LS, Sea MM-M, et al. Randomized trial examining effectiveness of lifestyle intervention in reducing gestational diabetes in high risk Chinese pregnant women in Hong Kong. Sci Rep 2018;8(December 1):13849. 4. Chasan-Taber L, Silveira M, Marcus BH, Braun B, Stanek E, Markenson G. Feasibility and efficacy of a physical activity intervention among pregnant women: the behaviors affecting baby and you (B.A.B.Y.) study. J Phys Act Health 2011;8(September 0 2):S228–38. 5. Choi J, Lee Jhyeon, Vittinghoff E, Fukuoka Y. mHealth physical activity intervention: a randomized pilot study in physically inactive pregnant women. Matern Child Health J 2016;20(May 5):1091–101 6. Currie S, Sinclair M, Liddle DS, Nevill A, Murphy MH. Application of objective physical activity measurement in an antenatal physical activity consultation intervention: a randomised controlled trial. BMC Public Health [Internet] 2015. . Dec 18 [cited 2019 Jun 18];15. Available from: https://www.ncbi.nlm. nih.gov/pmc/articles/PMC4684930/. 7. Dodd JM, Cramp C, Sui Z, Yelland LN, Deussen AR, Grivell RM, et al. The effects of antenatal dietary and lifestyle advice for women who are overweight or obese on maternal diet and physical activity: the LIMIT randomised trial. BMC Med [Internet] 2014. . Oct 13 [cited 2019 Jun 18];12. Available from: https:// www.ncbi.nlm.nih.gov/pmc/articles/PMC4194375/. 8. Dodd JM, Louise J, Cramp C, Grivell RM, Moran LJ, Deussen AR. Evaluation of a smartphone nutrition and physical activity application to provide lifestyle advice to pregnant women: the SNAPP randomised trial. Matern Child Nutr 2018;14(1)e12502. 9. Gaston A, Prapavessis H. Maternal-fetal disease information as a source of exercise motivation during pregnancy. Health Psychol Off J Div Health Psychol Am Psychol Assoc. 2009;28(November 6):726–33. 10. Guelinckx I, Devlieger R, Mullie P, Vansant G. Effect of lifestyle intervention on dietary habits, physical activity, and gestational weight gain in obese pregnant women: a randomized controlled trial. Am J Clin Nutr 2010;91(February 2):373–80. 11. Jackson Ra, Stotland Ne, Caughey Ab, Gerbert B. Improving diet and exercise in pregnancy with Video Doctor counseling: a randomized trial. Patient Educ Couns 2011;83(May 2):203–9. 12. Kong KL, Campbell CG, Foster RC, Peterson AD, Lanningham-Foster L. A pilot walking program promotes moderate-intensity physical activity during pregnancy. Med Sci Sports Exerc 2014;46(March 3):462. 13. Luoto R, Kinnunen TI, Aittasalo M, Kolu P, Raitanen J, Ojala K, et al. Primary prevention of gestational diabetes mellitus and large-for-Gestational-Age newborns by lifestyle counseling: a cluster-randomized controlled trial. PLoS Med [Internet] 2011. . May 17 [cited 2019 Jun 18];8(5). Available from: https:// www.ncbi.nlm.nih.gov/pmc/articles/PMC3096610/. 14. Szmeja MA, Cramp C, Grivell RM, Deussen AR, Yelland LN, Dodd JM. Use of a DVD to provide dietary and lifestyle information to pregnant women who are overweight or obese: a nested randomised trial. BMC Pregnancy Childbirth [Internet] 2014. . Dec 12 [cited 2019 Jun 18];14. Available from: https://www. ncbi.nlm.nih.gov/pmc/articles/PMC4280000/.   Wilkinson SA, McIntyre HD. Evaluation of the “healthy start to pregnancy” early antenatal health promotion workshop: a randomized controlled trial. BMC Pregnancy Childbirth 2012;19(November 12):131. |
| Jorge et al. 2015 | 1. Barakat R, Cordero Y, Coteron J, Luaces M, Montejo R. Exercise during pregnancy improves maternal glucose screen at 24-28 weeks: a randomised controlled trial. Br J Sports Med 2012; 46: 656-61. 2. Barakat R, Pelaez M, Lopez C, Montejo R, Coteron J. Exercise during pregnancy reduces the rate of cesarean and instrumental deliveries: results of a randomized controlled trial. J Matern Fetal Neonatal Med 2012; 25(11): 2372-6. 3. Barakat R, Pelaez M, Montejo R, et al. Exercise during pregnancy improves maternal health perception: a randomized controlled trial. Am J Obstet Gynecol 2011; 204: 402e1-e7. 4. Barakat R, Pelaez M, Montejo R, Refoyo I, Coteron J. Exercise throughout pregnancy does not cause preterm delivery. A randomized, controlled trial. J Phys Act Health 2014; 11(5): 1012-7. 5. Barakat R, Perales M, Bacchi M, Coteron J, Refoyo I. A program of exercise throughout pregnancy. Is it safe to mother and newborn? Am J Health Promot 2013; 29(1): 2-8. 6. Barakat R, Ruiz J, Stirling J, Zakynthinaki M, Lucia A. Type of delivery is not affected by light resistance and toning exercise training during pregnancy: a randomized controlled trial. Am J Obstet Gynecol 2009; 201(6): 590.e591-596. 7. Barakat R, Ruiz JR, Lucia A. Exercise during pregnancy and risk of maternal anaemia: a randomised controlled trial. Br J Sports Med 2009; 43(12): 954-6. 8. Bø K, Haakstad LA. Is pelvic floor muscle training effective when taught in a general fitness class in pregnancy? A randomised controlled trial. Physiotherapy 2011; 97(3): 190-5. 9. de Oliveria Melo AS, Silva JL, Tavares JS, Barros VO, Leite DF, Amorim MM. Effect of a physical exercise program during pregnancy on uteroplacental and fetal blood flow and fetal growth: a randomized controlled trial. Obstet Gynecol 2012; 120: 302-10. 10. Eggen MH, Stuge B, Mowinckel P, Jensen KS, Hagen KB. Can supervised group exercises including ergonomic advice reduce the prevalence and severity of low back pain and pelvic girdle pain in pregnancy? A randomized controlled trial. Physical Therapy 2012; 92: 781-90. 11. Haakstad LA, Bø K. Exercise in pregnant women and birth weight: a randomized controlled trial. BMC Pregnancy Childbirth 2011; 11: 66. 12. Halvorsen S, Haakstad LA, Edvardsen E, Bø K. Effect of aerobic dance on cardiorespiratory fitness in pregnant women: a randomised controlled trial. Physiotherapy 2013; 99(1): 42-8. 13. Pelaez M, Gonzalez-Cerron S, Montejo R, Barakat R. Pelvic floor muscle training included in a pregnancy exercise program is effective in primary prevention of urinary incontinence: A randomized controlled trial. Neurourol Urodyn 2014; 33(1): 67-71. 14. Price BB, Amini SB, Kappeler K. Exercise in pregnancy: effect on fitness and obstetric outcomes-a randomized trial. Med Sci Sports Exerc 2012; 44(12): 2263-9. 15. Salvesen KÅ, Stafne SN, Eggebø TM, Mørkved S. Does regular exercise in pregnancy influence duration of labor? A secondary analysis of a randomized controlled trial. Acta Obstet Gynecol Scand 2014; 93(1): 73-9. 16. Stafne SN, Salvesen KA, Romundstad PR, Stuge B, Morkved S. Does regular exercise during pregnancy influence lumbopelvic pain? A randomized controlled trial. Acta Obstet Gynecol Scand 2012; 91(5): 552-9. 17. Stafne SN, Salvesen KA, Romundstad PR, Torjusen IH, Morkved S. Does regular exercise including pelvic floor muscle training prevent urinary and anal incontinence during pregnancy? A randomised controlled trial. BJOG 2012; 119(10): 1270-80. |
| Leonard et al. 2021 | 1. Chao, A.M., Srinivas, S. K., Studt, S. K., Diewald, L. K., Sarwer, D. B., & Allison, K. C. (2017). A pilot randomized controlled trial of a technology-based approach for preventing excess weight gain during pregnancy among women with overweight. Frontiers in Nutrition, 4, 57. 2. Choi, J., Lee, J. H., Vittinghoff, E., & Fukuoka, Y. (2016). MHealth physical activity intervention: a randomized pilot study in physically inactive pregnant women. Maternal Child and Health Journal, 20, 1091–1101. 3. Dodd, J. M., Louise, J., Cramp, C., Grivell, R. M., Moran, L., & Deussen, A. R. (2018). Evaluation of a smartphone nutrition and physical activity application to provide lifestyle advice to pregnant women: the SNAPP randomised trial. Maternal and Child Nutrition, 14, e12502. 4. Herring, S. J., Cruice, J. F., Bennet, G. G., Rose, M. Z., Davey, A., & Foster, G. D. (2016). Preventing excessive gestational weight gain among African American women: a randomized clinical trial. Obesity (Silver Spring), 24, 30–36. 5. Hui, A. L., Ludwig, S., Gardiner, P., Sevenhuysen, G., Murray, R., Morris, M., & Shen, G. X. (2006). Community-based exercise and dietary intervention during pregnancy: a pilot study. Canadian Journal of Diabetes, 30, 1–7. 6. Hui, A., Back, L., Ludwig, S., Gardiner, P., Sevenhuysen, G., Dean, H., et al. (2011). Lifestyle intervention on diet and exercise reduced excessive gestational weight gain in pregnant women under a randomised controlled trial. BJOG: An International Journal of Obstetrics and Gynaecology, 119, 70–77. 7. Jackson, R. A., Stotland, N. E., Caughey, A. B., & Gerbert, B. (2011). Improving diet and exercise in pregnancy with video doctor counseling: a randomized trial. Patient Education and Counseling, 83, 203–209. 8. Kennelly, M. A., Ainscough, K., Lindsay, K. L., O’Sullivan, E., Gibney, E. R., McCarthy, M., et al. (2018). Pregnancy exercise and nutrition with smartphone application support: a randomized controlled trial. Obstetrics & Gynecology, 131, 818–826. 9. Li, L. J., Aris, I. M., Han, W. M., & Tan, K. H. (2019). A promising food-coaching intervention program to achieve optimal gestational weight gain in overweight and obese pregnant women: pilot randomized controlled trial of a smartphone app. JMIR Formative Research, 3, e13013. 10. Mauriello, L. M., Van Marter, D. F., Umanzor, C. D., Castle, P. H., & de Aguiar, E. L. (2016). Using mHealth to deliver behavior change interventions within prenatal care at community health centers. American Journal of Health Promotion, 30, 554–562. 11. Olson, C. M., Groth, S. W., Graham, M. L., Reschke, J. E., Strawderman, M. S., & Fernanadez, I. D. (2018). The effectiveness of an online intervention in preventing excessive gestational weight gain: the e-moms roc randomized controlled trial. BMC Pregnancy and Childbirth, 18, 148. 12. Phelan, S., Phipps, M. G., Abrams, B., Darroch, F., Schaffner, A., & Wing, R. R. (2011). Randomized trial of a behavioral intervention to prevent excessive gestational weight gain: the fit for delivery study. American Journal of Clinical Nutrition, 93, 772–779. 13. Poston, L., Briley, A. L., Barr, S., Bell, R., Croker, H., Coxon, K., Essex, H. N., Hunt, C., Hayes, L., Howard, L. M., Khazaezadeh, N., Kinnunen, T., Nelson, S. M., Oteng-Ntim, E., Robson, S. C., Sattar, N., Seed, P. T., Wardle, J., Sanders, T. A. B., & Sandall, J. (2013). Developing a complex intervention for diet and activity behaviour change in obese pregnant women (the UPBEAT trial): assessment of behavioural change and process evaluation in a pilot randomised controlled trial. BMC Pregnancy and Childbirth, 13, 148. 14. Poston, L., Bell, R., Croker, H., Flynn, A. C., Godfrey, K. M., Goff, L., et al. (2015). Effect of a behavioural intervention in obese pregnant women (the UPBEAT study): a multicentre, randomised controlled trial. The Lancet Diabetes & Endocrinology, 3, 767–777. 15. Redman, L. M., Gilmore, L. A., Breaux, J., Thomas, D. M., Elkind-Hirsch, K., Stewart, T., Hsia, D. S., Burton, J., Apolzan, J. W., Cain, L. E., Altazan, A. D., Ragusa, S., Brady, H., Davis, A., Tilford, J. M., Sutton, E. F., & Martin, C. K. (2017). Effectiveness of SmartMoms, a novel eHealth intervention for management of gestational weight gain: randomized controlled pilot trial. JMIR mHealth and uHealth, 5, e133. 16. Senek, M. (2018). An mHealth walking intervention for pregnant women with obesity. Doctoral, Sheffield Hallam University. 17. Smith, K., Lanningham-Foster, L., Welch, A., & Campbell, C. (2016). Web-based behavioral intervention increases maternal exercise but does not prevent excessive gestational weight gain in previously sedentary women. Journal of Physical Activity and Health, 13, 587–593. 18. Symons Downs, D., Rivera, D. E., Savage, J. S., Stetter, C., Kunselman, A., Pauley, A. M., et al. (2019). Optimizing an individually-tailored, adaptive intervention to manage weight in overweight and obese pregnant women. Annals of Behavioral Medicine, 53(S1), S149–S150. 19. Van Horn, L., Peaceman, A., Kwasny, M., Vincent, E., Fought, A., Josefson, J., et al. (2018). Dietary approaches to stop hypertension diet and activity to limit gestational weight: maternal offspring metabolics family intervention trial, a technology enhanced randomized trial. American Journal of Preventive Medicine, 55, 603–614. 20. Vesco, K. K., Karanja, N., King, J. C., Gillman, M. W., Leo, M. C., Perrin, N., McEvoy, C. T., Eckhardt, C. L., Smith, K. S., & Stevens, V. J. (2014). Efficacy of a group-based dietary intervention for limiting gestational weight gain among obese women: a randomized trial. Obesity (Silver Spring), 22, 1989–1996. 21. Willcox, J. C., Wilkinson, S. A., Lappas, M., Ball, K., Crawford, D., McCarthy, E., Fjeldsoe, B., Whittaker, R., Maddison, R., & Campbell, K. J. (2017). A mobile health intervention promoting healthy gestational weight gain for women entering pregnancy at a high body mass index: the txt4two pilot randomised controlled trial. BJOG: An International Journal of Obstetrics and Gynaecology, 124, 1718–1728 |
| Liu *et al.* 2023 | 1. Aihemaiti R. Prepregnancy overweight/obese pregnant women dietary intervention on dietary intake and pregnancy outcome. Xinjiang Medical University; 2017 [MA thesis]. 2. Facchinetti F, Vijai V, Petrella E, et al. Food glycemic index changes in overweight/obese pregnant women enrolled in a lifestyle program: a randomized controlled trial. Am J Obstet Gynecol MFM 2019;13:100030. https://doi.org/10.1016/j.ajogmf.2019.100030. 3. Geiker NRW, Magkos F, Zingenberg H, et al. A high-protein lowglycemic index diet attenuates gestational weight gain in pregnant women with obesity: the "An optimized programming of healthy children" (APPROACH) randomized controlled trial. Am J Clin Nutr 2022;1153:970e9. https://doi.org/10.1093/ajcn/nqab405. 4. Goletzke J, De Haene J, Stotland NE, et al. Effect of a low-glycemic load diet intervention on maternal and pregnancy outcomes in obese pregnant women. Nutrients 2021;133. https://doi.org/10. 3390/nu13030748. 5. Kizirian NV, Kong Y, Muirhead R, et al. Effects of a low-glycemic index diet during pregnancy on offspring growth, body composition, and vascular health: a pilot randomized controlled trial. Am J Clin Nutr 2016;1034:1073e82. https://doi.org/10.3945/ajcn.115. 123695. 6. Markovic TP, Muirhead R, Overs S, et al. Randomized controlled trial investigating the effects of a low-glycemic index diet on pregnancy outcomes in women at high risk of gestational diabetes mellitus: the GI baby 3 study. Diabetes Care 2016;391:31e8. https: //doi.org/10.2337/dc15-0572. 7. Menichini D, Petrella E, Dipace V, et al. The impact of an early lifestyle intervention on pregnancy outcomes in a cohort of insulin-resistant overweight and obese women. Nutrients 2020; 125. https://doi.org/10.3390/nu12051496. 8. Rhodes Et Pawlak DB, Takoudes TC, et al. Effects of a low-glycemic load diet in overweight and obese pregnant women: a pilot randomized controlled trial. Am J Clin Nutr 2010;926:1306e15. https: //doi.org/10.3945/ajcn.2010.30130. 9. Walsh JM, McGowan CA, Mahony R, et al. Low glycaemic index diet in pregnancy to prevent macrosomia (ROLO study): randomised control trial. Br Med J 2012;345:e5605. https: //doi.org/10.1136/bmj.e5605. 10. Zhang Y, Wang L, Yang W, et al. Effectiveness of low glycemic index diet consultations through a diet glycemic assessment app tool on maternal and neonatal insulin resistance: a randomized controlled trial. JMIR Mhealth Uhealth 2019;74:e12081. https: //doi.org/10.2196/12081 |
| Makaruk et al. 2019 | 1. Barakat R, Pelaez M, Cordero Y, et al. Exercise during pregnancy protects against hypertension andmacrosomia: randomized clinical trial. Am J Obstet Gynecol. 2016;214:649.e1–649.e8. 2. Barakat R, Perales M, Bacchi M, et al. A program of exercise throughout pregnancy. Is it safe to mother and newborn? Am J Health Promot. 2014;29:2–8. 3. Cordero Y, Mottola MF, Vargas J, et al. Exercise is associated with a reduction in gestational diabetes mellitus. Med Sci Sports Exerc. 2014;47:1328–1333. 4. da Silva SG, Hallal PC, Domingues MR, et al. A randomized controlled trial of exercise during pregnancy on maternal and neonatal outcomes: results from the PAMELA study. Int J Behav Nutr Phys Act. 2017;14:175. 5. Barakat R, Cordero Y,Coteron J, et al. Exercise during pregnancy improves maternal glucose screen at 24–28 weeks: a randomised controlled trial. Br J Sports Med. 2012;46:656–661. 6. Barakat R, Pelaez M, Lopez C, et al. Exercise during pregnancy and gestational diabetes-related adverse effects: a randomised controlled trial. Br J Sports Med. 2013;47:630–636. 7. Garnæs KK, Mørkved S, Salvesen Ø, et al. Exercise training and weight gain in obese pregnant women: a randomized controlled trial (ETIP trial). PLoS Med. 2016;13:e1002079. 8. Oostdam N, Van Poppel MN, Eekhoff EM, et al. Design of FitFor2 study: the effects of an exercise program on insulin sensitivity and plasma glucose levels in pregnant women at high risk for gestational diabetes. BMC Pregnancy Childbirth. 2009;9:1. 9. Oostdam N, Van Poppel MN, Wouters MG, et al. No effect of the FitFor2 exercise programme on blood glucose, insulin sensitivity, and birthweight in pregnant women who were overweight and at risk for gestational diabetes: results of a randomised controlled trial. BJOG. 2012;119:1098–1107. 10. Stafne SN, Salvesen KÅ, Romundstad PR, et al. Regular exercise during pregnancy to prevent gestational diabetes: a randomized controlled trial. Obstet Gynecol. 2012;119:29–36. 11. Wang C,Wei Y, Zhang X, et al. A randomized clinical trial of exercise during pregnancy to prevent gestational diabetes mellitus and improve pregnancy outcome in overweight and obese pregnant women. Am J Obstet Gynecol. 2017;216:340–351. |
| McDonald et al. 2016 | 1. Barakat R, Lucia A, Ruiz JR. Resistance exercise training during pregnancy and newborn’s birth size: a randomised controlled trial. Int J Obes 2009; 33(9):1048–1057. 2. Barakat R, Perales M, Bacchi M et al. A program of exercise throughout pregnancy. Is it safe to mother and newborn? Am J Health Promot 2014; 29(1): 2–8. 3. Cavalcante SR, Cecatti JG, Pereira RI et al. Water aerobics II: maternal body composition and perinatal outcomes after a program for low risk pregnant women. Reprod Health 2009; 6(1):19126239. 4. Clapp III JF, Kim H, Burciu B et al. Beginning regular exercise in early pregnancy: effect on fetoplacental growth. Am J Obstet Gynecol 2000; 183(6):1484–1488. 5. Clapp III JF, Kim H, Burciu B et al. Continuing regular exercise during pregnancy: effect of exercise volume on fetoplacental growth. Am J Obstet Gynecol 2002; 186(1):142–147. 6. Collings CA, Curet L, Mullin J. Maternal and fetal responses to a maternal aerobic exercise program. Am J Obstet Gynecol 1983; 145(6):702–707. 7. Davenport MH, Mottola MF, McManus R et al. A walking intervention improves capillary glucose control in women with gestational diabetes mellitus: a pilot study. Appl Phys Nutr Metab 2008; 33(3):511–517. 8. Garshasbi A, Faghih Zadeh S. The effect of exercise on the intensity of low back pain in pregnant women. Int J Gynaecol Obstet 2005; 88(3):271–275. 9. Haakstad LA, Bø K. Effect of regular exercise on prevention of excessive weight gain in pregnancy: a randomised controlled trial. Euro J Contracept Reprod Health Care 2011; 16(2):116–125. 10. Hopkins SA, Baldi JC, Cutfield WS et al. Exercise training in pregnancy reduces offspring size without changes in maternal insulin sensitivity. J Clin Endocrinol Metab 2010; 95(5):2080–2088. 11. Kardel KR, Kase T. Training in pregnant women: effects on fetal development and birth. Am J Obstet Gynecol 1998; 178(2):280–286. 12. Marquez-Sterling S, Perry AC, Kaplan T et al. Physical and psychological changes with vigorous exercise in sedentary primigravidae. Med Sci Sports Exerc 2000; 32(1):58–62. 13. Nascimento S, Surita F, Parpinelli M et al. The effect of an antenatal physical exercise programme on maternal/perinatal outcomes and quality of life in overweight and obese pregnant women: a randomised clinical trial. BJOG 2011; 118(12):1455–1463. 14. Ong M, Guelfi K, Hunter T et al. Supervised home-based exercise may attenuate the decline of glucose tolerance in obese pregnant women. Diabetes Metab 2009; 35(5):418–421. 15. Oostdam N, van Poppel M, Wouters M et al. No effect of the FitFor2 exercise programme on blood glucose, insulin sensitivity, and birthweight in pregnant women who were overweight and at risk for gestational diabetes: results of a randomised controlled trial. BJOG 2012; 119(9):1098–1107. 16. Price BB, Amini SB, Kappeler K. Exercise in pregnancy: effect on fitness and obstetric outcomes—a randomized trial. Med Sci Sports Exerc 2012; 44(12):2263–2269. 17. Ruchat S-M, Davenport MH, Giroux I et al. Nutrition and exercise reduce excessive weight gain in normal-weight pregnant women. Med Sci Sports Exerc 2012; 44(8):1419–1426. 18. Ruiz JR, Perales M, Pelaez M et al. Supervised exercise-based intervention to prevent excessive gestational weight gain: a randomized controlled trial. Mayo Clin Proc 2013; 88(12):1388–1397. 19. Santos IA, Stein R, Fuchs SC et al. Aerobic exercise and submaximal functional capacity in overweight pregnant women—a randomized trial. Obstet Gynecol 2005; 106(2):243–249. 20. Sedaghati P, Ziaee V, Ardjmand A. The effect of an ergometric training program on pregnants weight gain and low back pain. Gazz Med Ital—Arch Sci Med 2007; 166(6):209–213. 21. Yeo S. Adherence to walking or stretching, and risk of preeclampsia in sedentary pregnant women. Res Nurs Health 2009; 32(4):379–390. |
| Muktabhant et al. 2015 | 1. Althuizen E, van der Wijden C, van Mechelen W, SeidellJ, van Poppel M. The effect of a counselling intervention on weight changes during and after pregnancy: a randomised trial. BJOG : an International Journal of Obstetrics and Gynaecology2013;120(1):92–9. 2. Althuizen E, Van Poppel MN, Seidell JC, Van der Wijden C, Van Mechelen W. Design of the new life(style) study: a randomised controlled trial to optimise maternal weight development during pregnancy. BMC Public Health 2006;6:168. 3. Angel MD, De Haene J, Perez M, Hernandez G, Castaneda D, King JC. Dietary patterns associated with gestational weight gain and fat mass gain in overweight and obese pregnant women. FASEB Journal 2011;25:783.15. 4. Asbee SM, Jenkins TR, Butler JR, White J, Elliot M, Rutledge A. Dietary counseling prevents excessive weight gain during pregnancy, a randomized controlled trial. Obstetrics & Gynecology 2008;11(4 Suppl):6S. 5. Asbee SM, Jenkins TR, ButlerJR, White J, Elliot M, Rutledge A. Preventing excessive weight gain during pregnancy through dietary and lifestyle counseling: a randomized controlled trial. Obstetrics & Gynecology 2009;113 (2 Pt 1):305–12. 6. Barakat R, Pelaez M, Montejo R, Luaces M, Zakynthinaki M. Exercise during pregnancy improves maternal health perception: a randomized controlled trial. American Journal of Obstetrics and Gynecology 2011;204(5):402.e1–402.e7. 7. Barakat R. Effect of physical exercise program during pregnancy on excessive weight gain and its consequences. ClinicalTrials.gov(http://clinicaltrials.gov/) [accessed 21 May 2013] 2011. 8. Bisson M, Almeras N, Dufresne S, Rheaume C, Bujold E, Robitailee J, et al. Exercise improving fitness in obese women during pregnancy: a difference for the mother and child?. Pediatric Academic Societies and Asian Society for Pediatric Research Joint Meeting; 2014 May 3-6;Vancouver, Canada. 2014:Abstract no: 2946.618. 9. Bo K, Haakstad LAH. Is pelvic floor muscle training effective when taught in a general fitness class in pregnancy? A randomised controlled trial. Physiotherapy 2011;97(3):190–5. 10. Bogaerts A, Devlieger R, Nuyts E, Witters I, Gyselaers W, Guelinckx I. Psycho-education reduces gestational weight gain in obese pregnant women: Randomized controlled trial [abstract]. Obesity Facts 2012;5:53. 11. Bogaerts A. Effect of psycho-education on gestational weight gain and anxiety/depression in obese pregnant women. ClinicalTrials.gov(http://clinicaltrials.gov/) [accessed 21 May 2013] 2011. 12. Brand-Miller J. A pregnancy intervention to reduce postprandial glucose excursions in the primary prevention of paediatric obesity. Current Controlled Trials (www.controlled-trials.com/) [accessed 31 October 2010]. 13. Briley AL, Barr S, Badger S, Bell R, Croker H, Godfrey KM, et al. A complex intervention to improve pregnancy outcome in obese women; the UPBEAT randomised controlled trial. BMC Pregnancy and Childbirth 2014;14(1):74. 14. Broekhuizen K, Althuizen E, van Poppel MNM, Donker M, van Mechelen W. From theory to practice: intervention fidelity in a randomized controlled trial aiming to optimize weight development during pregnancy. Health Promotion Practice 2012;13(6):816–25. 15. Byrne NM, Groves AM, McIntyre HD, Callaway LK. Changes in resting and walking energy expenditure and walking speed during pregnancy in obese women. American Journal of Clinical Nutrition 2011;94(3):819–30. 16. Callaway L, McIntyre D, Colditz P, Byrne N, Foxcroft K, O’Connor B. Exercise in obese pregnant women: a randomized study to assess feasibility. Hypertension in Pregnancy 2008;27(4):549. 17. Callaway L. A randomized controlled trial using exercise to reduce gestational diabetes and other adverse maternal and neonatal outcomes in obese pregnant women - the pilot study. Australian Clinical Trials Registry (www.actr.org.au/)[accessed 31 October 2010]. 18. Callaway LK, Colditz PB, Byrne NM, Lingwood BE, Rowlands IJ, Foxcroft K, et al. Prevention of gestational diabetes. Feasibility issues for an exercise intervention in obese pregnant women. Diabetes Care 2010;33(7):1457–9. 19. Clapp III JF. Maternal carbohydrate intake and pregnancy outcome. Proceedings of the Nutrition Society 2002; 61(1):45–50. 20. Clapp IJF. Diet, exercise, and feto-placental growth. Archives of Gynecology and Obstetrics 1997;260:101–8. 21. Clapp JF 3rd, Kim H, Burciu B, Schmidt S, Petry K, Lopez B. Continuing regular exercise during pregnancy: effect of exercise volume on fetoplacental growth. American Journal of Obstetrics and Gynecology 2002;186(1):142–7. 22. Cordero Y, Mottola MF, Vargas J, Blanco M, Barakat R. Exercise is associated with a reduction in gestational diabetes mellitus. Medicine and Science in Sports and Exercise 2014;Oct 20 [Epub ahead of print]. 23. Cramp CS, Moran LJ, Deussen AR, Yelland LN, Dodd JM. Evaluation of printed nutrition education material in overweight and obese women during pregnancy-findings from the limit randomised trial. Journal of Paediatrics and Child Health 2013;49 Suppl 2:118. 24. De Oliveria Melo AS, Silva JLP, Tavares JS, Barros VO, Leite DFB, Amorim MMR. Effect of a physical exercise program during pregnancy on uteroplacental and fetal blood flow and fetal growth: A randomized controlled trial. Obstetrics & Gynecology 2012;120(2 Pt 1):302–10. 25. Di Carlo C, Iannotti G, Sparice S, Chiacchio MP, Greco E, Tommaselli GA, et al. The role of a personalized dietary intervention in managing gestational weight gain: a prospective, controlled study in a low-risk antenatal population. Archives of Gynecology and Obstetrics 2014;289:765–70. 26. Dodd J. Limiting weight gain in overweight and obese women during pregnancy to improve health outcomes: a randomised trial. Australian Clinical Trials Registry (www.actr.org.au/) (accessed 31 October 2010). 27. Dodd J. Obesity in pregnancy-the limit randomised trial. Journal of Paediatrics and Child Health 2013;49 Suppl 2:4. 28. Dodd JM, Cramp C, Sui Z, Yelland LN, Deussen AR, Grivell RM, et al. The effects of antenatal dietary and lifestyle advice for women who are overweight or obese onmaternal diet and physical activity: the LIMIT randomised trial. BMC Medicine 2014;12(1):161. 29. Dodd JM, McPhee AJ, Turnbull D, Yelland LN, Deussen  AR, Grivell RM, et al. The effects of antenatal dietary and lifestyle advice for women who are overweight or obese on neonatal health outcomes: the LIMIT randomised trial. BMC Medicine 2014;12(1):163. 30. Dodd JM, Turnbull D, McPhee AJ, Deussen AR, Grivell RM, Yelland LN, et al. Antenatal lifestyle advice for women who are overweight or obese: LIMIT randomised trial.BMJ (Clinical Research Ed.)2014;348:g1285. 31. Dodd JM, Turnbull DA, McPhee AJ, Wittert G, Crowther CA, Robinson JS. Limiting weight gain in overweight and obese women during pregnancy to improve health outcomes: the LIMIT randomised controlled trial. BMC Pregnancy and Childbirth 2011;11:79. 32. Dodd JM. Dietary and lifestyle advice for pregnant women who Are overweight or obese: the LIMIT randomized trial. Annals of Nutrition & Metabolism 2014;64(3-4):197–202. 33. Donnelly J, Horan M, Walsh J, McGowan C, Byrne J, Molloy EJ, et al. Impact of a low GI diet on neonatal body composition (ROLO Kids). Pediatric Academic Societies Annual Meeting; 2013 May 4-7; Washington DC, USA. 2013. 34. Donnelly JM, Walsh JM, Byrne J, Molloy E, McAuliffe FM. Altered neonatal anthropometric measurements following maternal low GI diet in pregnancy (ROLO study). Acta Obstetricia et Gynecologica Scandinavica 2013;92(s160):13. 35. Eames AJ, Grivell RM, Dodd JM, Deussen A. The effect of limited gestational weight gain in overweight and obese women on maternal and infant outcomes. Journal of Paediatrics and Child Health 2013;49 Suppl 2:19. 36. Ehrlich SF, Hedderson MM, Feng J, Crites Y, Quesenberry CP, Ferrara A. Lifestyle intervention improves postpartum fasting glucose levels in women with gestational diabetes. Diabetes 2014; 63 (Suppl 1):A95. 37. European Journal of Contraception and Reproductive Health Care 2011;16(2):116–25. 38. Facchinetti F. Pregnancy complications in women with BMI > 25kg/m2 enrolled in a healthy lifestule and eating habits program. ClinicalTrials.gov (accessed 21 May 2013). NCT01783210 2013. 39. Ferrara A, Hedderson MM, Albright CL, Ehrlich SF, Quesenberry CP, Peng T, et al. A pregnancy and postpartum lifestyle intervention in women with gestational diabetes mellitus reduces diabetes risk factors: a feasibility randomized control trial. Diabetes Care 2011;34(7):1519–25. 40. Ferrara A. Diet, exercise and breastfeeding intervention program for women with gestational diabetes (DEBI Trial). ClinicalTrials.gov(www.clinicaltrials.gov) [accessed 31 October 2010]. 41. Foxcroft KF, Rowlands IJ, Byrne NM, McIntyre HD, Callaway LK, for the BAMBINO group. Exercise in obese pregnant women: the role of social factors, lifestyle and pregnancy symptoms. BMC Pregnancy and Childbirth 2011;11:4. 42. Grivell R, Yelland L, Earl RA, Staehr CJ, Dodd J. The effect of antenatal dietary and lifestyle advice on fetal body composition in women who are overweight or obese: findings from the LIMIT randomised trial. Ultrasound in Obstetrics and Gynecology 2013;42(Suppl 1):10. 43. Grivell R, Yelland L, Staehr CJ, Earl RA, Dodd J. The effect of antenatal dietary and lifestyle advice on fetal growth in women who are overweight or obese: findings from the LIMIT randomised trial. Ultrasound in Obstetrics and Gynecology 2013;42(Suppl 1):83. 44. Guelinckx I, Devlieger R, Mullie P, Vansant G. Effect of lifestyle intervention on dietary habits, physical activity, and gestational weight gain in obese pregnant women: a randomized controlled trial. American Journal of Clinical Nutrition 2010;91(2):373–80. 45. Haakstad L, Bo K. Effect of supervised aerobic dance exercise in prevention of excessive weight gain in pregnancy: a single blind randomized controlled trial. International Journal of Gynecology & Obstetrics 2009;107(Suppl 2):S198. 46. Haakstad L, Bo K. Exercise during pregnancy-Does it impact offspring birth weight parameters?. Journal of Science and Medicine in Sport 2012;15(Suppl 1):S340. 47. Haakstad L. Effect of regular exercise in prevention of excessive weight gain in pregnancy. ClinicalTrials.gov (www.clinicaltrials.gov) [accessed 31 October 2010]. 48. Haakstad LA, Bo K. Exercise in pregnant women and birth weight: a randomized controlled trial. BMC Pregnancy and Childbirth  2011;11:66. 49. Haakstad LAH, Bo K. Effect of regular exercise on prevention of excessive weight gain in pregnancy: A randomised controlled trial. 50. Harrison CL, Lombard CB, Gibson-Helm M, Deeks A, Teede HJ. Limiting excess weight gain in high-risk pregnancies: A randomized controlled trial. Endocrine Reviews 2011; 32(3 Meeting Abstracts):P1–466. 51. Harrison CL, Lombard CB, Strauss BJ, Teede HJ. Optimizing healthy gestational weight gain in women at high risk of gestational diabetes: a randomized controlled trial. Obesity 2013;21(5):904–9. 52. Harrison CL, Teede HJ, Lombard CB. How effective is self-weighing in the setting of a lifestyle intervention to reduce gestational weight gain and postpartum weight retention?. Australian & New Zealand Journal of Obstetrics & Gynaecology 2014;54:382–5. 53. Hawkins M, Hosker M, Marcus BH, Rosal MC, Braun B, Stanek EJ, et al. A pregnancy lifestyle intervention to prevent gestational diabetes risk factors in overweight Hispanic women: a feasibility randomized controlled trial. Diabetic Medicine 2014;32:108–15. 54. Hayes L, Bell R, Robson S, Poston L. Association between physical activity in obese pregnant women and pregnancy outcomes: The UPBEAT Pilot Study. Annals of Nutrition & Metabolism 2014;64(3-4):239–46. 55. Horan M, McGowan C, Donnelly J, Gibney E, McAuliffe F. Maternal diet and weight at 3 months partum following a pregnancy intervention with a low glycaemic index diet:Results from the ROLO randomised control trial. Archives of Disease in Childhood. Fetal and Neonatal Edition 2014;99 (Suppl 1):A129-A130, Abstract no: PMM.20. 56. Horan MK, McGowan CA, Donnelly J, Gibney E, McAuliffe FM. The association of maternal characteristics and macronutrient intake in pregnancy with neonatal body composition. Archives of Disease in Childhood. Fetal and Neonatal Edition 2014;99(Suppl 1):A11. 57. Horan MK, McGowan CA, Doyle O, McAuliffe FM. Well-being in pregnancy: An examination of the effect of socioeconomic, dietary and lifestyle factors including impact of a low glycaemic index dietary intervention. European Journal of Clinical Nutrition 2014;68(1):19–24. 58. Horan MK, McGowan CA, Gibney ER, Donnelly JM, McAuliffe FM. Maternal diet and weight at 3 months postpartum following a pregnancy intervention with a low glycaemic index diet: results from the ROLO randomised control trial. Nutrients 2014;6(7):2946–55. 59. Horan MK, McGowan CA, Gibney ER, Donnelly JM, McAuliffe FM. Maternal low glycaemic index diet, fat intake and postprandial glucose influences neonatal adiposity - secondary analysis from the ROLO study. Nutrition Journal 2014;13(1):78. 60. Huang TT, Yeh CY, Tsai YC. A diet and physical activity intervention for preventing weight retention among Taiwanese childbearing women: a randomised controlled trial. Midwifery 2011;27(2):257–64. 61. Hui A, Back L, Ludwig S, Gardiner P, Sevenhuysen G, Dean H, et al. Lifestyle intervention on diet and exercise reduced excessive gestational weight gain in pregnant women undera randomised controlled trial. BJOG: an International Journal of Obstetrics & Gynaecology 2012;119(1):70–7. 62. Hui AL, Ludwig S, Gardiner P, Sevenhuysen G, Dean HJ, Sellers E, et al. Effects of lifestyle intervention on dietary intake, physical activity level, and gestational weight gain in pregnant women with different pre-pregnancy Body Mass Index in a randomized control trial. BMC Pregnancy and Childbirth 2014;14(1):331. 63. Hui AL, Ludwig SM, Gardiner P, Sevenhuysen G, Murray R, Morris M, et al. Community-based exercise and dietary intervention during pregnancy: a pilot study. Canadian Journal of Diabetes 2006; 30(2):169–75. 64. Jackson RA, Stotland NE, Caughey AB, Gerbert B. Improving diet and exercise in pregnancy with Video Doctor counseling: a randomized trial. Patient Education and Counseling 2011;83(2):203–9. 65. Jeffries K, Shub A, Walker SP, Hiscock R, Permezel M. Reducing excessive weight gain in pregnancy: a randomised controlled trial. 66. Kannieappan LM, Deussen AR, Moran LJ, Grivell RM, Yelland LN, Dodd JM. The effect of antenatal dietary advice on maternal body composition in women who are overweight or obese - findings from the limit randomised trial. Journal of Paediatrics and Child Health  2013;49 Suppl2:94. 67. Kieffer EC, Welmerink DB, Sinco BR, Welch KB, Rees Clayton EM, Schumann CY, et al. Dietary outcomes in a Spanish-language randomized controlled diabetes prevention trial with pregnant Latinas. American Journal of Public Health 2014;104(3):526–33. 68. Kong KL, Campbell G, Foster C, Peterson D, Lanningham-Foster L. A pilot walking program promotes moderate-intensity physical activity during pregnancy. Medicine & Science in Sports & Exercise 2014;46(3):462–72. 69. Korpi-Hyovalti E, Schwab U, Laaksonen DE, Linjama H, Heinonen S, Niskanen L. Effect of intensive counselling on and increasing physical activity during pregnancy: A feasibility study. Diabetes 2012;61 Suppl 1:A344. 70. Lombard C, Harrison C, Teede H. A randomized controlled trial investigating self-weighing and the prevention of excess weight gain in early pregnancy. Endocrine Reviews 2011;32(3 Meeting Abstracts):P2–768. 71. Mahony R, Byrne J, Curran S, O’Herlihy C, McAuliffe F. A pilot study of the feasibility of a randomised trial of low glycaemic diet versus normal diet from early pregnancy in euglycaemic women. Archives of Disease in Childhood. Fetal and Neonatal Edition 2008;93(Suppl 1):Fa38. 72. Maitland RA, Barr S, Briley A, Seed P, Poston L. Incidence of gestational diabetes in an obese population using the International Association of Diabetes and Pregnancy Study Groups (IADPSG) criteria in the UK Pregnancies Better Eating and Activity Trial (UPBEAT) pilot study. Diabetic Medicine 2012;29(Suppl 1):152. 73. McAuliffe F. A randomised controlled trial of low glycaemic index carbohydrate diet versus no dietary intervention in the prevention of recurrence of foetal macrosomia. CurrentControlled Trials (www.controlled-trials.com/) (accessed 12.05.2010). 74. McGowan CA, Walsh JM, Byrne J, Curran S, McAuliffe FM. The influence of a low glycemic index dietary intervention on maternal dietary intake, glycemic index and gestational weight gain during pregnancy: a randomized controlled trial. Nutrition Journal 2013;12(1):140. 75. Medical Journal of Australia 2009;191(8):429–33. 76. Melo A. Exercise and pregnancy: randomised clinical trial. Current Controlled Trials (www.controlled-trials.com/)[accessed 31 October 2010]. 77. Morkved S. Effects of regular exercise during pregnancy.ClinicalTrials.gov(www.clinicaltrials.gov) (accessed 31 October 2010). 78. Moses RG, Barker M, Winter M, Petocz P, Brand-Miller JC. Can a low-glycemic index diet reduce the need for insulin in gestational diabetes mellitus? A randomized trial. Diabetes Care 2009;32(6):996–1000. 79. Moses RG, Casey S, Cleary J, Milosavljevic M, Quinn E, Tapsell L, et al. Effect of low glycaemic index dietary advice in normal pregnancy: The PREGGIO study. Obesity Research and Clinical Practice 2013;7:e34–5. 80. Moses RG, Casey SA, Quinn EG, Cleary JM, Tapsell LC, Milosavljevic M, et al. Pregnancy and Glycemic Index Outcomes study: effects of low glycemic index compared with conventional dietary advice on selected pregnancy outcomes. American Journal of Clinical Nutrition 2014;99(3):517–23. 81. Mujsindi W, Habash D, Childs G. Impact of nutrition education on gestational weight gain in obese pregnant women. American Journal of Obstetrics and Gynecology 2014;210(1 Suppl):S188. 82. Murtezani A, Pacarada M, Ibraimi Z, Nevzati A, Abazi N. The impact of exercise during pregnancy on neonatal outcomes: a randomised controlled trial. Journal of Sports Medicine and Physical Fitness 2014;54(6):802–8. 83. Nascimento KLK, Surita SLN, Parpinelli FGS, Kasawara MAP. Type of delivery and neonatal outcome in overweight and obese pregnant women with excessive weight gain. Journal of Maternal-Fetal and Neonatal Medicine 2012;25(S2):73–4. 84. Nascimento SL, Surita FG, Parpinelli MA, Siani S, Pintoe Silva JL. The effect of an antenatal physical exercise programme on maternal/perinatal outcomes and quality of life in overweight and obese pregnant women: a randomised clinical trial. BJOG: an International Journal of Obstetrics & Gynaecology 2011;118(12):1455–63. 85. Newman AK, Deussen AR, Moran LJ, Grivell RM, Yelland LN, Turnbull D, et al. The effect of antenatal dietary and lifestyle advice on maternal psychological health in women who are overweight or obese-findings from the limit randomised trial. Journal of Paediatrics and Child Health 2013;49 Suppl 2:119. 86. Nilsson K. Weight gain during pregnancy - a randomized controlled trial of intervention to prevent excessive gestational weight gain. ClinicalTrials.gov (http://clinicaltrials.gov/) [accessed 5 September 2014] 2009. 87. Oostdam N, Bosmans J, Wouters MGAJ, Eekhoff EMW, van Mechelen W, van Poppel MNM. Cost-effectiveness of an exercise program during pregnancy to prevent gestational diabetes: Results of an economic evaluation alongside a randomised controlled trial. BMC Pregnancy and Childbirth 2012;12:64. 88. Oostdam N, Van Poppel MN, Eekhoff EM, Wouters MG, Van Mechelen W. Design of FitFor2 study: the effects of an exercise program on insulin sensitivity and plasma glucose levels in pregnant women at high risk for gestational diabetes. BMC Pregnancy and Childbirth 2009;9:1. 89. Oostdam N, Van Poppel MNM, Wouters MGAJ, Eekhoff EMW, Bekedam DJ, Kuchenbecker WKH, et al. No effect of the FitFor2 exercise programme on blood glucose, insulin sensitivity, and birthweight in pregnant women who were overweight and at risk for gestational diabetes: Results of a randomised controlled trial. BJOG: an International Journal of Obstetrics and Gynaecology 2012;119(9):1098–107. 90. Pawlak DB. Glycemic load and infant birth weight in pregnant overweight/obese women. ClinicalTrials.gov (http://clinicaltrials.gov/) (accessed 31 October 2010). 91. Petrella E, Facchinetti F, Bertarini V, Pignatti L, Neri I, Battistini NC. Occurrence of pregnancy complications in women with BMI > 25 submitted to a healthy lifestyle and eating habits program. American Journal of Obstetrics and Gynecology 2013;208(1 Suppl):S33–4. 92. Petrella E, Malavolti M, Bertarini V, Pignatti L, Neri I, Battistini NC, et al. Gestational weight gain in overweight and obese women enrolled in a healthy lifestyle and eating habits program. Journal of Maternal-Fetal and Neonatal Medicine 2014;27(13):1348–52. 93. Petrov Fieril K, Glantz A, Fagevik Olsen M. The efficacy of moderate-to-vigorous resistance exercise during pregnancy: A randomized controlled trial. Acta Obstetricia et Gynecologica Scandinavica 2014 Oct 7 [Epub ahead of print]. 94. Phelan S, Phipps MG, Abrams B, Darroch F, Grantham K, Schaffner A, et al. Does behavioral intervention in pregnancy reduce postpartum weight retention? Twelve-month outcomes of the Fit for Delivery randomized trial. American Journal of Clinical Nutrition 2014;99(2):302–11. 95. Phelan S, Phipps MG, Abrams B, Darroch F, Schaffner A, Wing RR. Factors associated with success in the “fit for delivery” intervention to reduce excessive gestational weight gain. Obesity 2011;19(Suppl 1):S95. 96. Phelan S, Phipps MG, Abrams B, Darroch F, Schaffner A, Wing RR. Randomized trial of a behavioral intervention to prevent excessive gestational weight gain: the Fit for Delivery Study. American Journal of Clinical Nutrition 2011;93(4):772–9. 97. Pinzon DC, Zamora K, Martinez JH, Florez-Lopez ME, de Plata AC, Mosquera M, et al. Type of delivery and 98. Pollak KI, Alexander SC, Bennett G, Lyna P, Coffman CJ, Bilheimer A, et al. Weight-related SMS texts promoting appropriate pregnancy weight gain: A pilot study. Patient Education and Counselling 2014;97:256–60. 99. Polley BA, Wing RR, Sims CJ. Randomized controlled trial to prevent excessive weight gain in pregnant women. International Journal of Obesity & Related Metabolic Disorders: Journal of the International Association for the Study of Obesity 2002;26(11):1494–502. 100. Poston L, Briley AL, Barr S, Bell R, Croker H, Coxon K, et al. Developing a complex intervention for diet andactivity behaviour change in obese pregnant women (the UPBEAT trial); assessment of behavioural change and process evaluation in a pilot randomised controlled trial. BMC Pregnancy and Childbirth 2013;13(1):148. 101. Poston L. Improving pregnancy outcome in obese women: a feasibility study. Current Controlled Trials (http://controlled-trials.com/) (accessed 31 October 2010). 102. Price B, Amini B, Kappeler K. Exercise in pregnancy: Effect on fitness and obstetric outcomes - a randomized trial. Medicine & Science in Sports & Exercise 2012;44(12):2263–9. 103. Quinlivan JA, Lam LT, Fisher J. A randomised trial of a four-step multidisciplinary approach to the antenatal care of obese pregnant women. Australian and New Zealand Journal of Obstetrics and Gynaecology 2011;51:141–6. 104. Rae A, Bond D, Evans S, North F, Roberman B, Walters B. A randomised controlled trial of dietary energy restriction in the management of obese women with gestational diabetes. Australian & New Zealand Journal of Obstetrics & Gynaecology 2000;40 (4):416–22. 105. Ramirez-Velez R, Aguilar de Plata AC, Escudero MM, Echeverry I, Ortega JG, Salazar B, et al. Influence of regular aerobic exercise on endothelium-dependent vasodilation and cardiorespiratory fitness in pregnant women. Journal of Obstetrics and Gynaecology Research 2011;37(11):1601–8. 106. Ramirez-Velez R. Aerobic exercise in pregnant Latina women: Effect on metabolic and body composition outcomes. A randomized clinical trial. FASEB Journal 2014;28(1 Suppl 1):886.6. 107. Ramirez-Velez R. Combined aerobic and resistance exercise on metabolic and body composition outcomes in primigravid Latina women. Obesity Reviews 2014;15(Suppl2):201–2. 108. Rauh K, Gabriel E, Kerschbaum E, Schuster T, von Kries R, Amann-Gassner U, et al. Safety and efficacy of a lifestyle intervention for pregnant women to prevent excessive maternal weight gain: a cluster-randomized controlled trial. BMC Pregnancy and Childbirth 2013;13(1):151. 109. Renault KM, Norgaard K, Nilas L, Carlsen EM, Cortes D, Pryds O, et al. The Treatment of Obese Pregnant Women (TOP) study: A randomized controlled trial of the effect of physical activity intervention assessed by pedometer with or without dietary intervention in obese pregnant women. American Journal of Obstetrics and Gynecology 2014;210(2):134.e1–9. 110. Rhodes ET, Pawlak DB, Takoudes TC, Ebbeling CB, Feldman HA, Lovesky MM, et al. Effects of a low-glycemic load diet in overweight and obese pregnant women: a pilot randomized controlled trial. American Journal of Clinical Nutrition 2010;92(6):1306–15. 111. Ronnberg A, Ostlund I, Fadl H, Gottvall T, Nilsson K. Intervention during pregnancy to reduce excessive gestational weight gain-a randomised controlled trial. BJOG:an International Journal of Obstetrics and Gynaecology 2014 Nov 4 [Epub ahead of print]. 112. Ruchat SM, Davenport MH, Giroux I, Hillier M, Batada A, Sopper MM, et al. Nutrition and exercise reduce excessive weight gain in normal-weight pregnant women. Medicine and Science in Sports and Exercise 2012;44(8):1419–26. 113. Ruiz R, Perales M, Pelaez M, Lopez C, Lucia A, Barakat R. Supervised exercise-based intervention to prevent excessive gestational weight gain: a randomized controlled trial. Mayo Clinic Proceedings 2013;88(12):1388–97. 114. Santaella MP. The role of a supervised physical exercise program as an alternative on the control of maternal estational weight gain. ClinicalTrials.gov (accessed 21 May20 13). NCT01790347 2013. 115. Santos IA, Stein R, Fuchs SC, Duncan BB, Ribeiro JP, Kroeff LR, et al. Aerobic exercise and submaximal functional capacity in overweight pregnant women: a randomized trial. Obstetrics & Gynecology 2005;106(2):243–9. 116. Schneeberger C, Flynn A, Barr S, Seed PT, Inskip HM, Poston L. Maternal diet patterns and glycaemic load in obese pregnant women taking part in a pilot trial of a lifestyle intervention (the upbeat trial). Diabetes 2014; Vol.63. 117. Shub A. Diet and exercise in pregnancy. Australian Clinical Trials Registry (www.actr.org.au/) [accessed 21 June 2007]. 118. Stafne SN, Salvesen KÅ, Romundstad PR, Eggebø TM, Carlsen SM, Mørkved S. Regular exercise during pregnancy to prevent gestational diabetes: a randomized controlled trial. Obstetrics & Gynecology 2012;119(1):29–36. 119. Sui Z, Yelland LN, Turnbull D, Dodd JM. Walking to limit gestational weight gain and keep fit during pregnancy - findings from the walk randomised trial. Journal of Paediatrics and Child Health 2013;49 Suppl 2:120. 120. Surita F. Physical exercise influence among overweightand obese pregnant women. ClinicalTrials.gov (http://clinicaltrials.gov/) [accessed 21 May 2013] 2010. 121. Szmeja MA, Grivell RM, Deussen AR, Dodd JM. Evaluation of information provision to women who are overweight or obese during pregnancy. Journal of Paediatrics and Child Health 2011;47(Suppl 1):78. 122. Tanvig M, Vinter CA, Jorgensen JS, Wehberg S, Ovesen PG, Beck-Nielsen H, et al. Effects of lifestyle intervention in pregnancy and anthropometrics at birth on offspring metabolic profile at 2.8 years - results from the Lifestyle in Pregnancy and Offspring (LiPO) study. Journal of Clinical Endocrinology and Metabolism 2015;100(1):175–83. 123. Tanvig M. Lifestyle in pregnancy and offspring - comparison between children born to obese women and children born to normal weight women (LiPO). ClinicalTrials.gov (http:// clinicaltrials.gov/) [accessed 6 February 2014] 2013. 124. Tanvig M. Lifestyle in pregnancy and offspring (LiPO). ClinicalTrials.gov(http://clinicaltrials.gov/) [accessed 6 February 2014] 2013. 125. Tanvig M. Offspring body size and metabolic profile - Effects of lifestyle intervention in obese pregnant women. Danish Medical Journal 2014;61(7):B4893. 126. Teede HJ, Harrison CL, Gibson-Helm M, Lombard CB. Improving physical activity in high-risk pregnancies: A randomized controlled trial. Endocrine Reviews 2011;32(3Meeting Abstracts):P1–467. 127. Thornton YS, Smarkola C, Kopacz SM, Ishoof SB. Perinatal outcomes in nutritionally monitored obese pregnant women: a randomized clinical trial. Journal of the National Medical Association 2009;101:569–77. 128. Umpierrez G. Lifestyle intervention to limit excessive weight gain during pregnancy in minority women. ClinicalTrials.gov (accessed 21 May 2013). NCT01084941 2010. 129. Van Poppel M, Oostdam N, Wouters M, Eekhoff M, Van Mechelen W. FitFor2: Effects of an exercise training program on the incidence of gestational diabetes. Journal of Science and Medicine in Sport 2012;15 Suppl 1:S342–S343. 130. Vesco K, Leo M, Gillman M, King J, McEvoy C, Karanjaa N, et al. Impact of a weight management intervention on pregnancy outcomes among obese women: The Healthy Moms Trial. American Journal of Obstetrics and Gynecology 2013;208(1 Suppl 1):S352. 131. Vesco KK, Karanja N, King JC, Gillman MW, Leo C, Perrin N, et al. Efficacy of a group-based dietary intervention for limiting gestational weight gain among obese women: A randomized trial. Obesity (Silver Spring, Md.)2014;2(9):1989–96. 132. Vesco KK, Karanja N, King JC, Gillman MW, Perrin N, McEvoy C, et al. Healthy Moms, a randomized trial to promote and evaluate weight maintenance among obese pregnant women: study design and rationale. Contemporary Clinical Trials 2012;33(4):777–85. 133. Vinter A. Lifestyle and pregnancy: the clinical effect of lifestyle intervention during pregnancy in obese women. ClinicalTrials.gov(www.clinicaltrials.gov) [accessed 31 October 2010]. 134. Vinter C, Jensen D, Ovesen P, Beck-Nielsen H, Lamont R, Jorgensen J. Postpartum weight retention and breastfeeding among obese women from the LiP (Lifestyle in Pregnancy) Study. Acta Obstetricia et Gynecologica Scandinavica 2012;91(Suppl 159):141–2. 135. Vinter CA, Jensen DM, Ovesen P, Beck-Nielsen H, Jorgensen JS. The LiP (Lifestyle in Pregnancy) study: a randomized controlled trial of lifestyle intervention in 360 obese pregnant women. Diabetes Care 2011;34(12):2502–7. 136. Vinter CA, Jensen DM, Ovesen P, Beck-Nielsen H, Tanvig M, Lamont RF, et al. Postpartum weight retention and breastfeeding among obese women from the randomized controlled Lifestyle in Pregnancy (LiP) trial. Acta Obstetricia et Gynecologica Scandinavica 2014;93:794–801. 137. Vitolo MR, Fraga Bueno MS, Mendes Gama C. Impact of a dietary counseling program on the gain weight speed of pregnant women attended in a primary care service. Revista Brasileira de Ginecologia e Obstetricia 2011;33(1):13–9. 138. Vitolo MR. Impact of a nutritional intervention program for weight control during pregnancy. ClinicalTrials.gov (www.clinicaltrials.gov) [accessed 31 October 2010]. 139. Walsh J, Mahony R, Foley M, Mc Auliffe F. A randomised control trial of low glycaemic index carbohydrate diet versus no dietary intervention in the prevention of recurrence of macrosomia. BMC Pregnancy and Childbirth2010;10:16. 140. Walsh J, Mahony R, Foley M, McAuliffe F. ROLO study: a randomized control trial of low glycemic index diet to prevent macrosomia in euglycemic women. American Journal of Obstetrics and Gynecology 2012;206,Suppl 1):S4. 141. Walsh J, McGowan C, Byrne J, Foley M, Mahony R, McAuliffe F. The influence of a low glycaemic index dietary intervention on maternal glycaemic index, dietary intake and gestational weight gain. American Journal of Obstetrics and Gynecology 2013;208(1 Suppl):S33. 142. Walsh JM, Mahony RM, Canty G, Foley ME, McAuliffe FM. Identification of those most likely to benefit from a low-glycaemic index dietary intervention in pregnancy. British Journal of Nutrition 2014;112:583–9. 143. Walsh JM, McGowan CA, Mahony R, Foley ME, McAuliffe FM. Low glycaemic index diet in pregnancy to prevent macrosomia (ROLO study): randomised control trial. BMJ 2012;345:e5605. 144. Wilkinson SA, McIntyre HD. Evaluation of the ’healthy start to pregnancy’ early antenatal health promotion workshop: a randomized controlled trial. BMC Pregnancy and Childbirth 2012;12:131. 145. Wolff S, Legarth J, Vangsgaard K, Toubro S, Astrup A. A randomized trial of the effects of dietary counseling on gestational weight gain and glucose metabolism in obese pregnant women. International Journal of Obesity 2008;32(3):495–501 |
| O’Brien  et al. 2014 | 1. Bot M, Milder IE, Bemelmans WJ. Nationwide implementation of Hello World: a Dutch email-based health promotion program for pregnant women. J Med Internet Res 2009; 11: e24. 2. ClinicalTrials.gov The MOTHER (Mobile Technologies to Help Enhancing Regular Physical Activity) trial for pregnant women. National Library of Medicine: Bethesda, MD, USA. 2011. Identifier NCT01461707. Available from http://www. clinicaltrials.gov/ct2/show/record/NCT01461707 3. ClinicalTrials.gov. Electronically-mediated weight interventions for pregnant and postpartum women. National Library of Medicine: Bethesda, MD, USA. 2013. Identifier NCT01331564. Available from http://clinicaltrials.gov/ct2/show/record/ NCT01331564. 4. ClinicalTrials.gov. Personalised management of body weight during pregnancy. National Library of Medicine: Bethesda, MD, USA. 2012. Identifier NCT01610752. Available from http://www.clinicaltrials.gov/ct2/show/NCT01610752. 5. Jackson RA, Stotland NE, Caughey AB, Gerbert B. Improving diet and exercise in pregnancy with Video Doctor counseling: a randomized trial. Patient Educ Couns 2011; 83: 203–209. 6. Nagle C, Skouteris H, Morris h, Nankervis A, Rasmussen B, Mayall P et al. Primary prevention of gestational diabetes for women who are overweight and obese: a randomised controlled trial. BMC Pregnancy Childbirth 2013; 13: 65. 7. van Zutphen M, Milder IE, Bemelmans WJ. Usage of an online healthy lifestyle program by pregnant women attending midwifery practices in Amsterdam. Prev Med 2008; 46: 552–557. |
| Perales et al. 2016 | 1. Asbee SM, Jenkins TR, Butler JR, White J, Elliot M, Rutledge A. Preventing excessive weight gain during pregnancy through dietary and lifestyle counseling: a random- ized controlled trial. Obstet Gynecol 2009;113(2 Pt 1):305–12 2. Baciuk EP,PereiraRI,CecattiJG,BragaAF,CavalcanteSR.Wateraerobicsinpregnan- cy: cardiovascular response, labor and neonatal outcomes. Reprod Health 2008;5:10. 3. Barakat R, Cordero Y, Coteron J, Luaces M, Montejo R. Exercise during pregnancy improves maternal glucose screen at 24–28 weeks: a randomised controlled trial. Br J Sports Med 2012;46(9):656–61. 4. Barakat R, Lucia A, Ruiz JR. Resistance exercise training during pregnancy and newborn's birth size: a randomised controlled trial. Int J Obes (Lond) 2009;33(9): 1048–57. 5. Barakat R, Pelaez M, Lopez C, Lucia A, Ruiz JR. Exercise during pregnancy and gesta- tional diabetes-related adverse effects: a randomised controlled trial. Br J Sports Med 2013;47(10):630–6. 6. Barakat R, Pelaez M, Lopez C, Montejo R, Coteron J. Exercise during pregnancy reduces the rate of cesarean and instrumental deliveries: results of a randomized controlled trial. J Matern Fetal Neonatal Med 2012;25(11):2372–6. 7. Barakat R, Pelaez M, Montejo R, Luaces M, Zakynthinaki M. Exercise during pregnan- cy improves maternal health perception: a randomized controlled trial. Am J Obstet Gynecol 2011;204(5):402 [e1-7]. 8. Barakat R, Perales M, Bacchi M, Coteron J, Refoyo I. A program of exercise throughout pregnancy. Is it safe to mother and newborn? Am J Health Promot 2014;29(1):2–8. 9. Barakat R, Ruiz JR, Lucia A. Exercise during pregnancy and risk of maternal anaemia: a randomised controlled trial. Br J Sports Med 2009;43(12):954–6. 10. Barakat R, Ruiz JR, Stirling JR, Zakynthinaki M, Lucia A. Type of delivery is not affect- ed by light resistance and toning exercise training during pregnancy: a randomized controlled trial. Am J Obstet Gynecol 2009;201(6):590 [e1-6]. 11. Barakat R, Stirling JR, Lucia A. Does exercise training during pregnancy affect gesta- tional age? A randomised controlled trial. Br J Sports Med 2008;42(8):674–8 12. Callaway LK, Colditz PB, Byrne NM, et al. Prevention of gestational diabetes: feasibil- ity issues for an exercise intervention in obese pregnant women. Diabetes Care 2010;33(7):1457–9. 13. Cavalcante SR, Cecatti JG, Pereira RI, Baciuk EP, Bernardo AL, Silveira C. Water aero- bics II: maternal body composition and perinatal outcomes after a program for low risk pregnant women. Reprod Health 2009;6:1. 14. Cordero Y, Mottola MF, Vargas J, Blanco M, Barakat R. Exercise is associated with a reduction in gestational diabetes mellitus. Med Sci Sports Exerc 2015;47(7):1328–33. 15. Dekker Nitert M, Barrett HL, Denny KJ, McIntyre HD, Callaway LK. group B. Exercise in pregnancy does not alter gestational weight gain, MCP-1 or leptin in obese women. Aust N Z J Obstet Gynaecol 2015;55(1):27–33. 16. Eggen MH, Stuge B, Mowinckel P, Jensen KS, Hagen KB. Can supervised group exer- cises including ergonomic advice reduce the prevalence and severity of low back pain and pelvic girdle pain in pregnancy? A randomized controlled trial. Phys Ther 2012;92(6):781–90. 17. Garshasbi A, Faghih Zadeh S. The effect of exercise on the intensity of low back pain in pregnant women. Int J Gynaecol Obstet 2005;88(3):271–5. 18. Ghodsi Z, Asltoghiri M. Effects of aerobic exercise training on maternal and neonatal outcome: a randomized controlled trial on pregnant women in Iran. J Pak Med Assoc 2014;64(9):1053–6. 19. Haakstad LA, Bo K. Effect of a regular exercise programme on pelvic girdle and low back pain in previously inactive pregnant women: a randomized controlled trial. J Rehabil Med 2015;47(3):229–34 20. Haakstad LA, Bo K. Effect of regular exercise on prevention of excessive weight gain in pregnancy: a randomised controlled trial. Eur J Contracept Reprod Health Care 2011;16(2):116–25. 21. Haakstad LA, Bo K. Exercise in pregnant women and birth weight: a randomized controlled trial. BMC Pregnancy Childbirth 2011;11:66 22. Halvorsen S, Haakstad LA, Edvardsen E, Bo K. Effect of aerobic dance on cardiorespi- ratory fitness in pregnant women: a randomised controlled trial. Physiotherapy 2013;99(1):42–8. 23. Hopkins SA, Baldi JC, Cutfield WS, McCowan L, Hofman PL. Effects of exercise training on maternal hormonal changes in pregnancy. Clin Endocrinol (Oxf) 2011;74(4):495–500. 24. Hopkins SA, Baldi JC, Cutfield WS, McCowan L, Hofman PL. Exercise training in preg- nancy reduces offspring size without changes in maternal insulin sensitivity. J Clin Endocrinol Metab 2010;95(5):2080–8. 25. Hui A, Back L, Ludwig S, et al. Lifestyle intervention on diet and exercise reduced ex- cessive gestational weight gain in pregnant women under a randomised controlled trial. BJOG 2012;119(1):70–7. 26. Hui AL, Back L, Ludwig S, et al. Effects of lifestyle intervention on dietary intake, physical activity level, and gestational weight gain in pregnant women with differ- ent pre-pregnancy body mass index in a randomized control trial. BMC Pregnancy Childbirth 2014;14:331. 27. Kolu P, Raitanen J, Luoto R. Physical activity and health-related quality of life during pregnancy: a secondary analysis of a cluster-randomised trial. Matern Child Health J 2014;18(9):2098–105 28. Kong KL, Campbell CG, Foster RC, Peterson AD, Lanningham-Foster L. A pilot walking program promotes moderate-intensity physical activity during pregnancy. Med Sci Sports Exerc 2014;46(3):462–71. 29. Kordi R, Abolhasani M, Rostami M, Hantoushzadeh S, Mansournia MA, Vasheghani-Farahani F. Comparison between the effect of lumbopelvic belt and home based pel- vic stabilizing exercise on pregnant women with pelvic girdle pain; a randomized controlled trial. J Back Musculoskelet Rehabil 2013;26(2):133–9. 30. Marquez-Sterling S, Perry AC, Kaplan TA, Halberstein RA, Signorile JF. Physical and psychological changes with vigorous exercise in sedentary primigravidae. Med Sci Sports Exerc 2000;32(1):58–62. 31. Montoya Arizabaleta AV, Orozco Buitrago L. Aguilar de Plata AC, Mosquera Escudero M, Ramirez-Velez R. Aerobic exercise during pregnancy improves health-related quality of life: a randomised trial. J Physiother 2010;56(4):253–8. 32. Morkved S, Salvesen KA, Schei B, Lydersen S, Bo K. Does group training during preg- nancy prevent lumbopelvic pain? A randomized clinical trial. Acta Obstet Gynecol Scand 2007;86(3):276–82. 33. Murtezani A, Pacarada M, Ibraimi Z, Nevzati A, Abazi N. The impact of exerciseduring pregnancy on neonatal outcomes: a randomized controlled trial. J Sports Med Phys Fitness 2014;54(6):802–8. 34. Nascimento SL, Surita FG, Parpinelli MA, Siani S. Pinto e Silva JL. The effect of an antenatal physical exercise programme on maternal/perinatal outcomes and quality of life in overweight and obese pregnant women: a randomised clinical trial. BJOG 2011;118(12):1455–63. 35. Nobles C, Marcus BH, Stanek 3rd EJ, et al. Effect of an exercise intervention on gesta- tional diabetes mellitus: a randomized controlled trial. Obstet Gynecol 2015;125(5): 1195–204. 36. Ong MJ, Guelfi KJ, Hunter T, Wallman KE, Fournier PA, Newnham JP. Supervised home-based exercise may attenuate the decline of glucose tolerance in obese preg- nant women. Diabetes Metab 2009;35(5):418–21. 37. Oostdam N, Bosmans J, Wouters MG, Eekhoff EM, van Mechelen W, van Poppel MN. Cost-effectiveness of an exercise program during pregnancy to prevent gestational diabetes: results of an economic evaluation alongside a randomised controlled trial. BMC Pregnancy Childbirth 2012;12:64. 38. Oostdam N, van Poppel MN, Wouters MG, et al. No effect of the FitFor2 exercise pro- gramme on blood glucose, insulin sensitivity, and birthweight in pregnant women who were overweight and at risk for gestational diabetes: results of a randomised controlled trial. BJOG 2012;119(9):1098–107. 39. Pelaez M, Gonzalez-Cerron S, Montejo R, Barakat R. Pelvic floor muscle training in- cluded in a pregnancy exercise program is effective in primary prevention of urinary incontinence: a randomized controlled trial. NeurourolUrodyn 2014;33(1):67–71. 40. Perales M, Calabria I, Lopez C, Franco E, Coteron J, Barakat R. Regular exercise throughout pregnancy is associated with a shorter first stage of labor. Am J Health Promot 2015. 41. Perales M, Refoyo I, Coteron J, Bacchi M, Barakat R. Exercise during pregnancy atten- uates prenatal depression: a randomized controlled trial. Eval Health Prof 2015; 38(1):59–72. 42. Petrov Fieril K, Glantz A, Fagevik Olsen M. The efficacy of moderate-to-vigorous re- sistance exercise during pregnancy: a randomized controlled trial. Acta Obstet Gynecol Scand 2015;94(1):35–42. 43. Phelan S, Phipps MG, Abrams B, Darroch F, Schaffner A, Wing RR. Randomized trial of a behavioral intervention to prevent excessive gestational weight gain: the Fit for Delivery study. Am J Clin Nutr 2011;93(4):772–9. 44. Pinzon DC, Zamora K, Martinez JH, et al. Type of delivery and gestational age is not affected by pregnant Latin-American women engaging in vigorous exercise: a sec- ondary analysis of data from a controlled randomized trial. Rev Salud Publica (Bogota) 2012;14(5):731–43. 45. Polley BA, Wing RR, Sims CJ. Randomized controlled trial to prevent excessive weight gain in pregnant women. Int J Obes Relat Metab Disord 2002;26(11): 1494–502 46. Price BB, Amini SB, Kappeler K. Exercise in pregnancy: effect on fitness and obstetric outcomes—a randomized trial. Med Sci Sports Exerc 2012;44(12):2263–9. 47. Ramirez-Velez R. Aerobic exercise training during pregnancy increases antioxidant status in nulliparous women: secondary analysis of a controlled clinical trial. Endocrinol Nutr 2013;60(5):279–81. 48. Ramirez-Velez R. Aguilar de Plata AC, Escudero MM, et al. Influence of regular aero- bic exercise on endothelium-dependent vasodilation and cardiorespiratory fitness in pregnant women. J Obstet Gynaecol Res 2011;37(11):1601–8. 49. Renault KM, Norgaard K, Nilas L, et al. The Treatment of Obese Pregnant Women (TOP) study: a randomized controlled trial of the effect of physical activity interven- tion assessed by pedometer with or without dietary intervention in obese pregnant women. Am J Obstet Gynecol 2014;210(2):134 [e1-9]. 50. Robledo-Colonia AF, Sandoval-Restrepo N, Mosquera-Valderrama YF, Escobar-Hurtado C, Ramirez-Velez R. Aerobic exercise training during pregnancy reduces de- pressive symptoms in nulliparous women: a randomised trial. J Physiother 2012; 58(1):9–15. 51. Ronnberg AK, Ostlund I, Fadl H, Gottvall T, Nilsson K. Intervention during pregnancy to reduce excessive gestational weight gain—a randomised controlled trial. BJOG 2015;122(4):537–44. 52. Ruiz JR, Perales M, Pelaez M, Lopez C, Lucia A, Barakat R. Supervised exercise-based intervention to prevent excessive gestational weight gain: a randomized controlled trial. Mayo Clin Proc 2013;88(12):1388–97. 53. Salvesen KA, Stafne SN, Eggebo TM, Morkved S. Does regular exercise in pregnancy influence duration of labor? A secondary analysis of a randomized controlled trial. Acta Obstet Gynecol Scand 2014;93(1):73–9. 54. Santos IA, Stein R, Fuchs SC, et al. Aerobic exercise and submaximal functional capacity in overweight pregnant women: a randomized trial. Obstet Gynecol 2005: 106(2):243–9. 55. Stafne SN, Salvesen KA, Romundstad PR, Eggebo TM, Carlsen SM, Morkved S. Regular exercise during pregnancy to prevent gestational diabetes: a randomized controlled trial. Obstet Gynecol 2012;119(1):29–36. 56. Stafne SN, Salvesen KA, Romundstad PR, Stuge B, Morkved S. Does regular exercise during pregnancy influence lumbopelvic pain? A randomized controlled trial. Acta Obstet Gynecol Scand 2012;91(5):552–9. 57. Stafne SN, Salvesen KA, Romundstad PR, Torjusen IH, Morkved S. Does regular exer- cise including pelvic floor muscle training prevent urinary and anal incontinence during pregnancy? A randomised controlled trial. BJOG 2012;119(10):1270–80. 58. Tomic V, Sporis G, Tomic J, Milanovic Z, Zigmundovac-Klaic D, Pantelic S. The effect of maternal exercise during pregnancy on abnormal fetal growth. Croat Med J 2013; 54(4):362–8. 59. Vinter CA, Jensen DM, Ovesen P, Beck-Nielsen H, Jorgensen JS. The LiP (lifestyle in pregnancy) study: a randomized controlled trial of lifestyle intervention in 360 obese pregnant women. Diabetes Care 2011;34(12):2502–7. 60. Yeo S, Davidge S, Ronis DL, Antonakos CL, Hayashi R, O'Leary S. A comparison of walking versus stretching exercises to reduce the incidence of preeclampsia: a ran-domized clinical trial. Hypertens Pregnancy 2008;27(2):113–30. 61. Yeo S. Adherence to walking or stretching, and risk of preeclampsia in sedentary pregnant women. Res Nurs Health 2009;32(4):379–90. |
| Poyatos-Leon et al. 2015 | 1. Barakat R, Cordero Y, Coteron J, Luaces M, Montejo R. Exercise during pregnancy improves maternal glucose screen at 24–28 weeks: a randomised controlled trial. Br J Sports Med. 2012;46:656–61. 2. Barakat R, Pelaez M, Lopez C, Lucıa A, Ruiz J. Exercise during pregnancy and gestational diabetes-related adverse effects: a randomised controlled trial. Br J Sports Med. 2013;47:630–6. 3. Barakat R, Pelaez M, Lopez C, Montejo R, Coteron J. Exercise during pregnancy reduces the rate of cesarean and instrumental deliveries: results of a randomized controlled trial. J Matern Fetal Neonatal Med. 2012;25:2372–6. 4. Barakat R, Pelaez M, Montejo R, Luaces M, Zakynthinaki M. Exercise during pregnancy improves maternal health perception: a randomized controlled trial. Am J Obstet Gynecol. 2011;204:402.e1–7. 5. Barakat R, Perales M, Bacchi M, Coteron J, Refoyo I. A program of exercise throughout pregnancy. Is it safe to mother and newborn? Am J Health Promot. 2013;29:2–8. 6. Barakat R, Ruiz JR, Stirling JR, Zakynthinaki M, Lucia A. Type of delivery is not affected by light resistance and toning exercise training during pregnancy: a randomized controlled trial. Am J Obstet Gynecol. 2009;201:590.e1–6. 7. Cavalcante SR, Cecatti JG, Pereira RI, Baciuk EP, Bernardo AL, Silveira C. Water aerobics II: maternal body composition and perinatal outcomes after a program for low risk pregnant women. Reprod Health. 2009;6:1. 8. Price BB, Amini SB, Kappeler K. Exercise in pregnancy: effect on ﬁtness and obstetric outcomes a randomized trial. Med Sci Sports Exerc. 2012;44:2263–9. 9. Ruiz JR, Perales M, Pelaez M, Lopez C, Lucıa A, Barakat R. Supervised exercise-based intervention to prevent excessive gestational weight gain: a randomized controlled trial. Mayo Clin Proc. 2013;88:1388–97. 10. Stafne SN, Salvesen KA, Romundstad PR, Eggebo TM, Carlsen SM, Morkved S. Regular exercise during pregnancy to prevent gestational diabetes: a randomized controlled trial. Obstet Gynecol. 2012;119:29–36. |
| Rhodes et al., 2020 | 1. Choi J, Lee JH, Vittinghoff E, Fukuoka Y. mHealth physical activity intervention: a randomized pilot study in physically inactive pregnant women. Matern Child Health J 2016 May;20(5):1091-1101 2. Dahl A. Healthy Motivations for Moms-To-Be (Healthy MoM2B) Study: A Mobile Health Intervention Targeting Gestational Weight Gain among US Women. Semantic Scholar. 2018. URL: https://www.semanticscholar.org/paper/Healthy-Motivations-for-Moms-To-Be-(Healthy-MoM2B)Dahl/828d334dde24173f90099c03457c0674e0050740 [accessed 3. Evans W, Nielsen P, Szekely D, Bihm J, Murray E, Snider J, et al. Dose-response effects of the text4baby mobile health program: randomized controlled trial. JMIR Mhealth Uhealth 2015 Jan 28;3(1):e12 4. Evans WD, Wallace JL, Snider J. Pilot evaluation of the text4baby mobile health program. BMC Public Health 2012 Nov 26;12:103132. 5. Hayman M, Reaburn P, Browne M, Vandelanotte C, Alley S, Short CM. Feasibility, acceptability and efficacy of a web-based computer-tailored physical activity intervention for pregnant women - the Fit4Two randomised controlled trial. BMC Pregnancy Childbirth 2017 Mar 23;17(1):96 6. Huberty J, Buman M, Leiferman J, Bushar J, Hekler E, Adams MJ. Dose and timing of text messages for increasing physical activity among pregnant women: a randomized controlled trial. Transl Behav Med 2017 Jun;7(2):212-223 7. Olson C, Groth S, Graham M, Reschke J, Strawderman M, Fernandez I. The effectiveness of an online intervention in preventing excessive gestational weight gain: the e-moms ROC randomized controlled trial. BMC Pregnancy Childbirth 2018 May 9;18(1):148 8. Pollak K, Alexander S, Bennett G, Lyna P, Coffman C, Bilheimer A, et al. Weight-related SMS texts promoting appropriate pregnancy weight gain: a pilot study. Patient Educ Couns 2014 Nov;97(2):256-260 9. Redman LM, Gilmore LA, Breaux J, Thomas DM, Elkind-Hirsch K, Stewart T, et al. Effectiveness of SmartMoms, a novel ehealth intervention for management of gestational weight gain: randomized controlled pilot trial. JMIR Mhealth Uhealth 2017 Sep 13;5(9):e133 10. Smith K, Lanningham-Foster L, Welch A, Campbell CK. Web-based behavioral intervention increases maternal exercise but does not prevent excessive gestational weight gain in previously sedentary women. J Phys Act Health 2016 Jun;13(6):587-593. 11. Willcox J, Wilkinson S, Lappas M, Ball K, Crawford D, McCarthy E, et al. A mobile health intervention promoting healthy gestational weight gain for women entering pregnancy at a high body mass index: the txt4two pilot randomised controlled trial. BJOG 2017 Oct;124(11):1718-1728 |
| Sanabria-Martin et al. 2015 | 1. Barakat R, Cordero Y, Coteron J, Luaces M, Montejo R. Exercise during pregnancy improves maternal glucose screen at 24-28 weeks: a randomized controlled trial. Br J Sports Med 2012;46:656–61. 2. Barakat R, Pelaez M, Lopez C, Lucia A, Ruiz JR. Exercise during pregnancy and gestational diabetes-related adverse effects: a randomized controlled trial. Br J Sports Med 2013;47:630–6. 3. Barakat R, Pelaez M, Montejo R, Luaces M, Zakynthinaki M. Exercise during pregnancy improves maternal health perception: a randomized controlled trial. Am J Obstet Gynecol 2011;204:402 e1–7 4. Barakat R, Pelaez M, Montejo R, Refoyo I, Coteron J. Exercise throughout pregnancy does not cause preterm delivery. A randomized controlled trial. J Phys Act Health 2014;11:1012–7. 5. Barakat R, Perales M, Bacchi M, Coteron J, Refoyo I. A program of exercise throughout pregnancy. Is it safe to mother and newborn?. Am J Health Promot 2014;2014:2014–8. 6. Barakat R, Ruiz JR, Stirling JR, Zakynthinaki M, Lucia A. Type of delivery is not affected by light resistance and toning exercise training during pregnancy: a randomized controlled trial. Am J Obstet Gynecol 2009;201:590.e1–6. 7. Cavalcante SR, Cecatti JG, Pereira RI, Baciuk EP, Bernardo AL, Silveira C. Water aerobics II: maternal body composition and perinatal outcomes after a program for low risk pregnant women. Reprod Health 2009;6:1. 8. Clapp JF, Kim H, Burciu B, Lopez B. Beginning regular exercise in early pregnancy: effect on fetoplacental growth. Am J Obstet Gynecol 2000;183:1484–8. 9. Haakstad LAH, Bø K. Exercise in pregnant women and birth weight: a randomized controlled trial. BMC Pregnancy Childbirth 2011;11:66. 10. Hopkins S, Baldi JC, Cutfield WS, McCowan L, Hofman PL. Effects of exercise training on maternal hormonal changes in pregnancy. Clin Endocrinol 2011;74:495–500. 11. Price BB, Saeid B, Kappeper K. Exercise in pregnancy: effect on fitness and obstetric outcomes-A randomized trial. Med Sci Sports Exerc 2012;44:2263–9. 12. Ruiz J, Perales M, Pelaez M, Lopez C, Lucia A, Barakat R. Supervised exercise-based intervention to prevent excessive gestational weight gain: a randomized controlled trial. May Clin Proc 2013;88:1388–97 13. Tomic V, Sporis G, Tomic J, Milanovic Z, Zigmundovac-Klaic D, Pantelic S. The effect of maternal exercise during pregnancy on abnormal fetal growth. Croat Med J 2013;54:362–8. |
| Sheffield *et al.* 2016 | 1. Beddoe AE, & Lee KA (2010). Effects of mindful yoga on sleep in pregnant women: A pilot study— Mind-body interventions during pregnancy. Biological Research for Nursing, 11, 363–370. doi: 10.1177/1099800409356320 [PubMed: 20338897] 2. Beddoe AE, Paul Yang CP, Kennedy HP, Weiss SJ, & Lee KA (2009). The effects of mindfulnessbased yoga during pregnancy on maternal psychological and physical distress. Journal of Obstetric, Gynecologic, & Neonatal Nursing, 38, 310–319. doi:10.1111/j.1552-6909.2009.01023.x 3. Doran F, & Hornibrook J (2013). Women’s experiences of participation in a pregnancy and postnatal group incorporating yoga and facilitated group discussion: A qualitative evaluation. Women and Birth, 26, 82–86. doi:10.1016/j.wombi.2012.06.001 [PubMed: 22818031] 4. Field T, Diego M, Delgado J, & Medina L (2013a). Yoga and social support reduce prenatal depression, anxiety, and cortisol. Journal of Bodywork and Movement Therapies, 17, 397–403. doi: 10.1016/j.jbmt.2013.03.010 [PubMed: 24138994] 5. Field T, Diego M, Delgado J, & Medina L (2013b). Tai chi/yoga reduces prenatal depression, anxiety, and sleep disturbances. Complementary Therapies in Clinical Practice, 19(1), 6–10. doi:10.1016/ j.ctcp.2012.10.001 [PubMed: 23337557] 6. Field T, Diego M, Hernandez-Reif M, Medina L, Delgado J, & Hernandez A (2012). Yoga and massage therapy reduce prenatal depression and prematurity. Journal of Bodywork and Movement Therapies, 16, 204–209. doi:10.1016/j.jbmt.2011.08.002 [PubMed: 22464118] 7. Ko YL, Yang CL, Fang CL, Lee MY, & Lin PC (2013). Community-based postpartum exercise program. Journal of Clinical Nursing, 22, 2122–2131. doi:10.1111/jocn.12117 [PubMed: 23398359] 8. Mitchell J, Field T, Diego M, Bendell D, Newton R, & Pelaez M (2012). Yoga reduces prenatal depression symptoms. Psychology, 3, 782–786. doi:10.4236/psych.2012.329118 9. Muzik M, Hamilton SE, Rosenblum KL, Waxler E, & Hadi Z (2012). Mindfulness yoga during pregnancy for psychiatrically at-risk women: Preliminary results from a pilot feasibility study. Complementary Therapies in Clinical Practice, 18, 235–240. doi:10.1016/j.ctcp.2012.06.006 [PubMed: 23059438] 10. Rakhshani A, Maharana S, Raghuram N, Nagendra HR, & Venkatram P (2010). Effects of integrated yoga on quality of life and interpersonal relationship of pregnant women. Quality of Life Research, 19, 1447–1455. doi:10.1007/s11136-010-9709-2 [PubMed: 20632115] 11. Reis PJ, & Alligood MR (2014). Prenatal yoga in late pregnancy and optimism, power, and well-being. Nursing Science Quarterly, 27(1), 30–36. doi:10.1177/0894318413509706 [PubMed: 24403034] 12. Satyapriya M, Nagarathna R, Padmalatha V, & Nagendra HR (2013). Effect of integrated yoga on anxiety, depression and well-being in normal pregnancy. Complementary Therapies in Clinical Practice, 19, 230–236. doi:10.1016/j.ctcp.2013.06.003 [PubMed: 24199979] 13. Satyapriya M, Nagendra HR, Nagarathna R, & Padmalatha V (2009). Effect of integrated yoga on stress and heart rate variability in pregnant women. International Journal of Gynecology & Obstetrics, 104, 218–222. [PubMed: 19110245] |
| Sherifali et al. 2017 | 1. Carral F, Ayala Mdel C, Fernádez JJ, González C, Piñero A, García G, et al. Web-based telemedicine system is useful for monitoring glucose control in pregnant women with diabetes. Diabetes Technol Ther 2015 May;17(5):349-354. [doi: 10.1089/dia.2014.0223] [Medline: 25629547] 2. Herring SJ, Cruice JF, Bennett GG, Rose MZ, Davey A, Foster GD. Preventing excessive gestational weight gain among African American women: a randomized clinical trial. Obesity (Silver Spring) 2016 Jan;24(1):30-36 [FREE Full text] [doi: 10.1002/oby.21240] [Medline: 26592857] 3. Pérez-Ferre N, Galindo M, Fernández MD, Velasco V, Runkle I, de la Cruz MJ, et al. The outcomes of gestational diabetes mellitus after a telecare approach are not inferior to traditional outpatient clinic visits. Int J Endocrinol 2010;2010:386941 [FREE Full text] [doi: 10.1155/2010/386941] [Medline: 20628517] 4. Pollak KI, Alexander SC, Bennett G, Lyna P, Coffman CJ, Bilheimer A, et al. Weight-related SMS texts promoting appropriate pregnancy weight gain: a pilot study. Patient Educ Couns 2014 Nov;97(2):256-260 [FREE Full text] [doi: 10.1016/j.pec.2014.07.030] [Medline: 25153313] 5. Smith K, Lanningham-Foster L, Welch A, Campbell C. Web-based behavioral intervention increases maternal exercise but does not prevent excessive gestational weight gain in previously sedentary women. J Phys Act Health 2016 Jun;13(6):587-593. [doi: 10.1123/jpah.2015-0219] [Medline: 26594820] 6. Soltani H, Duxbury AM, Arden MA, Dearden A, Furness PJ, Garland C. Maternal obesity management using mobile technology: a feasibility study to evaluate a text messaging based complex intervention during pregnancy. J Obes 2015;2015:814830 [FREE Full text] [doi: 10.1155/2015/814830] [Medline: 25960889] |
| Skouteris et al. 2014 | 1. Asbee SM, Jenkins TR, Butler JR, et al. Preventing excessive weight gain during pregnancy through dietary and lifestyle counseling: a randomized controlled trial. Obstet Gynecol. 2009;113:305– 12. 2. Hui A, Back L, Ludwig S, Sevenhuysen G, Dean H, et al. Lifestyle intervention on diet and exercise reduced excessive gestational weight gain in pregnant women under a randomised controlled trial. BJOG. 2012;119:70–7. 3. Jeffries K, Shub A, Walker SP, et al. Reducing excessive weight gain in pregnancy: a randomised controlled trial. Med J Aust. 2009;191:8. 4. Luoto R, Kinnunen TI, Aittasalo M, et al. Primary prevention of gestational diabetes mellitus and large-for-gestational-age newborns by lifestyle counseling: a cluster-randomized controlled trial. PLoS Med. 2011;8:1–11. 5. Polley BA, Wing RR, Sims CJ. Randomized controlled trial to prevent excessive weight gain in pregnant women. Int J Obes. 2002;26:1494–502. 6. Quinlivan J, Lam L, Fisher J. A randomised trial of a four-step multidisciplinary approach to the antenatal care of obese pregnant women. Aust N Z J Obstet Gynaecol. 2011;51:141–6. 7. Shirazian T, Monteith S, Friedman F, et al. Lifestyle modification program decreases pregnangy weight gain in obese women. Am J Perinatol. 2010;27:411–4. 8. Stafne S, Salvesen K, Romundstad P, et al. Regular exercise during pregnancy to prevent gestational diabetes a randomized controlled trial. Obstet Gynecol. 2012;119:29–36. 9. Thornton Y, Smarkola C, Kopacz S, et al. Perinatal outcomes in nutritionally monitored obese pregnant women: a randomized clinical trial. J Natl Med Assoc. 2009;101:568–77 |
| Soltani et al. 2016 | 1. Asbee, T. R. Jenkins, J. R. Butler, J. White, M. Elliot, and A. Rutledge, “Preventing excessive weight gain during pregnancy through dietary and lifestyle counseling: a randomized controlled trial,” Obstetrics & Gynecology, vol. 113, no. 2, pp. 305– 312, 2009. 2. Baciuk, R. I. Pereira, J. G. Cecatti, A. F. Braga, and S. R. Cavalcante, “Water aerobics in pregnancy: cardiovascular response, labor and neonatal outcomes,” Reproductive Health, vol. 5, article 10, 2008. 3. Badrawi, M. K. Hassanein, M. H. H. Badraoui, Y. A. Wafa, H. A. Shawky, and N. Badrawi, “Pregnancy outcome in obese pregnant mothers,” Journal of Perinatal Medicine, vol. 20, supplement 1, p. 203, 1992. 4. Barakat, A. Lucia, and J. R. Ruiz, “Resistance exercise training during pregnancy and newborn’s birth size: a randomised controlled trial,” International Journal of Obesity, vol. 33, no. 9, pp. 1048–1057, 2009. 5. Barakat, Y. Cordero, J. Coteron, M. Luaces, and R. Montejo, “Exercise during pregnancy improves maternal glucose screen at 24-28 weeks: a randomised controlled trial,” British Journal of Sports Medicine, vol. 46, no. 9, pp. 656–661, 2012. 6. Bechtel-Blackwell, “Computer-assisted self-interview and nutrition education in pregnant teens,” Clinical Nursing Research, vol. 11, no. 4, pp. 450–462, 2002. 7. Bell and S. M. Palma, “Antenatal exercise and birthweight,” The Australian and New Zealand Journal of Obstetrics and Gynaecology, vol. 40, no. 1, pp. 70–73, 2000. 8. Briley, N. L. Flanagan, and N. M. Lewis, “In-home prenatal nutrition intervention increased dietary iron intakes and reduced low birthweight in low-income African-American women,” Journal of the American Dietetic Association, vol. 102, no. 7, pp. 984–987, 2002. 9. Bung, R. Artal, N. Khodiguian, and S. Kjos, “Exercise in gestational diabetes: an optional therapeutic approach?” Diabetes, vol. 40, supplement 2, pp. 182–185, 1991. 10. Cavalcante, J. G. Cecatti, R. I. Pereira, E. P. Baciuk, A. L. Bernardo, and C. Silveira, “Water aerobics II: maternal body composition and perinatal outcomes after a program for low risk pregnant women,” Reproductive Health, vol. 6, article 1, 2009. 11. Clapp III, H. Kim, B. Burciu, and B. Lopez, “Beginning regular exercise in early pregnancy: effect on fetoplacental growth,” American Journal of Obstetrics & Gynecology, vol. 183, no. 6, pp. 1484–1488, 2000. 12. Clapp, “Diet, exercise, and feto-placental growth,” Archieves of Gynecology and Obstetrics, vol. 260, no. 1, pp. 101–108, 1997. 13. Crowther, J. E. Hiller, J. R. Moss, A. J. McPhee, W. S. Jeffries, and J. S. Robinson, “Effect of treatment of gestational diabetes mellitus on pregnancy outcomes,” The New England Journal of Medicine, vol. 352, no. 24, pp. 2477–2486, 2005. 14. Erkkola and M. Makela, “Heart volume and physical fitness of parturients,” Annals of Clinical Research, vol. 8, no. 1, pp. 15– 21, 1976. 15. Erkkola, “The influence of physical training during pregnancy on physical work capacity and circulatory parameters,” Scandinavian Journal of Clinical & Laboratory Investigation, vol. 36, no. 8, pp. 747–754, 1976. 16. Ferrara, M. M. Hedderson, C. L. Albright et al. “A pregnancy and postpartum lifestyle intervention in women with gestational diabetes mellitus reduces diabetes risk factors. A feasibility randomized control trial,” Diabetes Care, vol. 34, no. 7, pp. 1519–1525, 2011. 17. Garshasbi and Z. S. Faghih, “The effect of exercise on the intensity of low back pain in pregnant women,” International Journal of Gynecology & Obstetrics, vol. 88, no. 3, pp. 271–275, 2005. 18. Gomez, J. G. Delgado, A. A. Agudelo, and H. Hurtado, “Diet effects on the perinatal result of obese pregnant patient,” Revista Colombiana de Obstetricia y Ginecolog´ıa, vol. 45, pp. 313–316, 1994 (Spanish). 19. Guelinckx, R. Devlieger, P. Mullie, and G. Vansant, “Effect of lifestyle intervention on dietary habits, physical activity, and gestational weight gain in obese pregnant women: a randomized controlled trial,” The American Journal of Clinical Nutrition, vol. 91, no. 2, pp. 373–380, 2010. 20. Haakstad and K. Bo, “Exercise in pregnant women and birth weight: a randomized controlled trial,” BMC Pregnancy and Childbirth, vol. 11, article 66, 2011. 21. Hopkins, J. C. Baldi, W. S. Cutfield, L. McCowan, and P. L. Hofman, “Exercise training in pregnancy reduces offspring size without changes in maternal insulin sensitivity,” The Journal of Clinical Endocrinology & Metabolism, vol. 95, no. 5, pp. 2080– 2088, 2010. 22. Huang, C.-Y. Yeh, and Y.-C. Tsai, “A diet and physical activity intervention for preventing weight retention among Taiwanese childbearing women:a randomised controlled trial,” Midwifery, vol. 27, no. 2, pp. 257–264, 2011. 23. Hui, L. Back, S. Ludwig et al. “Lifestyle intervention on diet and exercise reduced excessive gestational weight gain in pregnant women under a randomised controlled trial,” BJOG, vol. 119, no. 1, pp. 70–77, 2011. 24. Hui, S. M. Ludwig, P. Gardiner et al. “Community-based exercise and dietary intervention during pregnancy: a pilot study,” Canadian Journal of Diabetes, vol. 30, no. 2, pp. 169–175, 2006. 25. Jackson, N. E. Stotland, A. B. Caughey, and B. Gerbert, “Improving diet and exercise in pregnancy with Video Doctor counseling: a randomized trial,” Patient Education and Counseling, vol. 83, no. 2, pp. 203–209, 2011. 26. Jeffries, A. Shub, S. P. Walker, R. Hiscock, and M. Permezel, “Reducing excessive weight gain in pregnancy: a randomised controlled trial,” The Medical Journal of Australia, vol. 191, no. 8, pp. 429–433, 2009. 27. Khaledan, S. Midar, N. S. Motahari Tabari, and M. Ahmad Shirvani, “Effect of an aerobic exercise program on fetal growth in pregnant women,” Journal of HAYAT, vol. 16, no. 1, pp. 55–64, 2010. 28. Khoury, T. Henriksen, B. Christophersen, and S. Tonstad, “Effect of a cholesterol-lowering diet on maternal, cord, and neonatal lipids, and pregnancy outcome: a randomized clinical trial,” American Journal of Obstetrics & Gynecology, vol. 193, no. 4, pp. 1292–1301, 2005. 29. Landon, C. Y. Spong, E. Thom et al. “A multicenter, randomized trial of treatment for mild gestational diabetes,”The New England Journal of Medicine, vol. 361, no. 14, pp. 1339–1348, 2009. 30. Lee, S. Challenger, M. McNabb, and M. Sheridan, “Exercise in pregnancy,” Modern Midwife, vol. 6, no. 8, pp. 28–33, 1996. 31. Marquez-Sterling, A. C. Perry, T. A. Kaplan, R. A. Halberstein, and J. F. Signorile, “Physical and psychological changes with vigorous exercise in sedentary primigravidae,” Medicine and Science in Sports and Exercise, vol. 32, no. 1, pp. 58–62, 2000. 32. Ney, D. R. Hollingsworth, and L. Cousins, “Decreased insulin requirement and improved control of diabetes in pregnant women given a high-carbohydrate, high-fiber, low-fat diet,” Diabetes Care, vol. 5, no. 5, pp. 529–533, 1982. 33. Ong, K. J. Guelfi, T. Hunter, K. E. Wallman, P. A. Fournier, and J. P. Newnham, “Supervised home-based exercise may attenuate the decline of glucose tolerance in obese pregnant women,” Diabetes & Metabolism, vol. 35, no. 5, pp. 418–421, 2009. 34. Phelan, M. G. Phipps, B. Abrams, F. Darroch, A. Schaffner, and R. R. Wing, “Randomized trial of a behavioral intervention to prevent excessive gestational weight gain: the Fit for delivery study,” American Journal of Clinical Nutrition, vol. 93, no. 4, pp. 772–779, 2011. 35. PKulpa, B. M. White, and R. Visscher, “Aerobic exercise in pregnancy,” American Journal of Obstetrics and Gynecology, vol. 156, no. 6, pp. 1395–1403, 1987 36. Polley, R. R. Wing, and C. J. Sims, “Randomized controlled trial to prevent excessive weight gain in pregnant women,” International Journal of Obesity and Related Metabolic Disorders, vol. 26, no. 11, pp. 1494–1502, 2002. 37. Prevedel, I. M. P. Calderon, M. H. De Conti, E. B. Consonni, and M. V. C. Rudge, “Maternal and perinatal effects of hydrotherapy in pregnancy,” Revista Brasileira de Ginecologia e Obstetr´ıcia, vol. 25, no. 1, pp. 53–59, 2003. 38. Quinlivan, L. T. Lam, and J. Fisher, “A randomised trial of a four-step multidisciplinary approach to the antenatal care of obese pregnant women,” The Australian and New Zealand Journal of Obstetrics and Gynaecology, vol. 51, no. 2, pp. 141–146, 2011. 39. Rae, D. Bond, S. Evans, F. North, B. Roberman, and B. Walters, “A randomised controlled trial of dietary energy restriction in the management of obese women with gestational diabetes,” The Australian and New Zealand Journal of Obstetrics and Gynaecology, vol. 40, no. 4, pp. 416–422, 2000. 40. Santos, R. Stein, S. C. Fuchs et al. “Aerobic exercise and submaximal functional capacity in overweight pregnant women: a randomized trial,” Obstetrics & Gynecology, vol. 106, no. 2, pp. 243–249, 2005. 41. Sedaghati, V. Ziaee, and A. Ardjmand, “The effect of an ergometric training program on pregnants weight gain and low back pain,” Gazzetta Medica Italiana Archivio per le Scienze Mediche, vol. 166, no. 6, pp. 209–213, 2007. 42. Thornton, C. Smarkola, S. M. Kopacz, and S. B. Ishoof, “Perinatal outcomes in nutritionally monitored obese pregnant women: a randomized clinical trial,” Journal of the National Medical Association, vol. 101, no. 6, pp. 569–577, 2009. 43. Vinter, D. M. Jensen, P. Ovesen, H. Beck-Nielsen, and J. S. Jørgensen, “The LiP (Lifestyle in Pregnancy) study: a randomized controlled trial of lifestyle intervention in 360 obese pregnant women,” Diabetes Care, vol. 34, no. 12, pp. 2502– 2507, 2011. 44. Wolff, J. Legarth, K. Vangsgaard, S. Toubro, and A. Astrup, “A randomized trial of the effects of dietary counseling on gestational weight gain and glucose metabolism in obese pregnant women,” International Journal of Obesity, vol. 32, no. 3, pp. 495– 501, 2008. 45. Yeo, N. M. Steele, M.-C. Chang, S. M. Leclaire, D. L. Ronis, and R. Hayashi, “Effect of exercise on blood pressure in pregnant women with a high risk of gestational hypertensive disorders,” Journal of Reproductive Medicine for the Obstetrician and Gynecologist, vol. 45, no. 4, pp. 293–298, 2000. |
| Tieu et al. 2017 | 1. Clapp JF. Influence of endurance exercise and diet on human placental development and fetal growth. Placenta 2006;27(6-7):527–34 2. Clapp JF III. Diet, exercise, and feto-placental growth. Archives of Gynecology and Obstetrics 1997;260:101–8 3. Clapp JF III. Effect of dietary carbohydrate on the glucose and insulin response to mixed caloric intake and exercise in both nonpregnant and pregnant women. Diabetes Care 1998;21(Suppl 2):B107–B112. Clapp JF III. Maternal carbohydrate intake and pregnancy outcome. Proceedings of the Nutrition Society 2002;61: 45–50 4. Donnelly J, Horan M, Walsh J, McGowan C, Byrne J, Molloy EJ, et al. Impact of a low GI diet on neonatal body composition (ROLO Kids). Pediatric Academic Societies Annual Meeting; 2013 May 4-7; Washington DC, USA. 2013 5. Donnelly JM, Lindsay KL, Walsh JM, Horan M, Molloy EJ, McAuliffe FM. Fetal metabolic influences of neonatal anthropometry and adiposity. BMC Pediatrics 2015;15(1): 175 6. Donnelly JM, Walsh JM, Byrne J, Molloy E, McAuliffe FM. Altered neonatal anthropometric measurements following maternal low GI diet in pregnancy (ROLO study). Acta Obstetricia et Gynecologica Scandinavica 2013;92(s160):13 7. Donnelly JM, Walsh JM, Byrne J, Molloy EJ, McAuliffe FM. Impact of maternal diet on neonatal anthropometry: A randomized controlled trial. Pediatric Obesity 2015;10(1): 52–6 8. Donnelly JMT, Lindsay KL, Walsh JM, Horan M, Molloy EJ, McAuliffe F. Impact of maternal and fetal inflammatory markers on neonatal and infant adiposity. Archives of Disease in Childhood 2015;100:A133–4 9. Fraser RB, Ford FA, Milner RDG. A controlled trial of a high dietary fibre intake in pregnancy - effects on plasma glucose and insulin levels. Diabetologia 1983;25:238–41 10. Fraser RB. High fibre diets in pregnancy. Nutrition in Pregnancy. Proceedings of 10th Study Group of the Royal College of Obstetricians and Gynaecologists; 1982 September. London: RCOG, 1983:269–80 11. Goletzke J, Buyken AE, Louie JC, Moses RG, BrandMiller JC. Dietary micronutrient intake during pregnancy is a function of carbohydrate quality. American Journal of Clinical Nutrition 2015;102(3):626–32 12. Hoppu U, Isolauri E, Koskinen P, Laitinen K. Diet and blood lipids in 1-4 year-old children. Nutrition Metabolism and Cardiovascular Diseases 2013;23(10):980–6 13. Hoppu U, Isolauri E, Laakso P, Matomaki J, Laitinen K. Probiotics and dietary counselling targeting maternal dietary fat intake modifies breast milk fatty acids and cytokines. European Journal of Nutrition 2012;51(2):211–9 8. Laitinen K, Ilmonen J, Isolauri E. Dietary counselling and probiotic intervention during pregnancy modify postpartum adiposity. Annals of Nutrition and Metabolism 2011;58(Suppl 3):87 14. Horan M, Donnelly J, Gibney E, McGowan C, McAuliffe F. The association of maternal characteristics and macronutrient intake in pregnancy with neonatal body composition. The Power of Programming 2014: International Conference on Developmental Origins of Adiposity and Long-Term Health; 2014 March 13-15; Munich, Germany. 2014 15. Horan M, McGowan C, Donnelly J, Gibney E, McAuliffe F. Maternal diet and weight at 3 months partum following a pregnancy intervention with a low glycaemic index diet: Results from the ROLO randomised control trial. Archives of Disease in Childhood. Fetal and Neonatal Edition 2014;99 (Suppl 1):A129-A130, Abstract no: PMM.20 16. Horan MK, McGowan CA, Donnelly J, Gibney E, McAuliffe FM. The association of maternal characteristics and macronutrient intake in pregnancy with neonatal body composition. Archives of Disease in Childhood. Fetal and Neonatal Edition 2014;99(Suppl 1):A11 17. Horan MK, McGowan CA, Doyle O, McAuliffe FM. Well-being in pregnancy: An examination of the effect of socioeconomic, dietary and lifestyle factors including impact of a low glycaemic index dietary intervention. European Journal of Clinical Nutrition 2014;68(1):19–24 18. Horan MK, McGowan CA, Gibney ER, Donnelly JM, McAuliffe FM. Maternal diet and weight at 3 months postpartum following a pregnancy intervention with a low glycaemic index diet: results from the ROLO randomised control trial. Nutrients 2014;6(7):2946–55 19. Horan MK, McGowan CA, Gibney ER, Donnelly JM, McAuliffe FM. Maternal low glycaemic index diet, fat intake and postprandial glucose influences neonatal adiposity - secondary analysis from the ROLO study. Nutrition Journal 2014;13(1):78. ISRCTN54392969. A randomised controlled trial of low glycaemic index carbohydrate diet versus no dietary intervention in the prevention of recurrence of foetal macrosomia. controlled- trials.com/ISRCTN54392969 Date first received: August 2009 20. Kizirian N, Garnett S, Markovic T, Ross G, Louie J, Muirhead R, et al. Effects of a low-glycaemic index diet during pregnancy on offspring body composition: a pilot study. Obesity Research and Clinical Practice 2013;7(Suppl 2):e103 21. Kizirian N, Garnett S, Markovic T, Ross G, Muirhead R, Brodie S, et al. Maternal diet and infant body composition in women at risk of gestational diabetes mellitus. Obesity Research and Clinical Practice 2014;8(Suppl 1):55 20. Markovic TP, Muirhead R, Overs S, Kizirian N, Louie J, Sweeting A, et al. Predictors of birthweight in women at high risk of gestational diabetes mellitus. Obesity Research and Clinical Practice 2013;7(2):e3–4 22. Laitinen K, Poussa T, Isolauri E, Nutrition, Allergy, Mucosal Immunology and Intestinal Microbiota Group. Probiotics and dietary counselling contribute to glucose regulation during and after pregnancy: a randomised controlled trial. British Journal of Nutrition 2009;101(11): 1679–87 23. Luoto R, Laitinen K, Nermes M, Isolauri E. Impact of maternal probiotic-supplemented dietary counseling during pregnancy on colostrum adiponectin concentration: A prospective, randomized, placebo-controlled study. Early Human Development 2012;88(6):339–44 24. Luoto R, Laitinen K, Nermes M, Isolauri E. Impact of maternal probiotic-supplemented dietary counselling on pregnancy outcome and prenatal and postnatal growth: a double-blind, placebo-controlled study. British Journal of Nutrition 2010;103(12):1792–9 25. Luoto R, Nermes M, Laitinen K, Isolauri E. Impact of maternal probiotic-supplemented dietary counselling on pregnancy outcome and prenatal and postnatal growth: a double-blind, placebo-controlled study. Pediatric Academic Societies Annual Meeting; 2009 May 2-5; Baltimore, USA. 2009 26. Mahony R, Byrne J, Curran S, O’Herlihy C, McAuliffe F. A pilot study of the feasibility of a randomised trial of low glycaemic diet versus normal diet from early pregnancy in euglycaemic women. Archives of Disease in Childhood. Fetal and Neonatal Edition 2008;93(Suppl 1):Fa38 27. Markovic 2016 ACTRN12610000681055. A randomized, two-arm parallel dietary intervention study to compare the effects of consuming a low glycemic diet or wholegrain high fibre diet on infant birth weight and body composition, complications related to Gestational Diabetes Mellitus (GDM) and progression to GDM diagnosis in women at high-risk of GDM. anzctr.org.au/Trial/Registration/ TrialReview.aspx?id=335632 Date first received: 18 August 2010 28. Markovic TP, Muirhead R, Overs S, Ross GP, Louie JC, Kizirian N, et al. Randomized controlled trial investigating the effects of a low-glycemic index diet on pregnancy outcomes in women at high risk of gestational diabetes mellitus: the GI Baby 3 Study. Diabetes Care 2016;39(1): 31–8 29. McAuliffe F. Maternal nutrition and fetal health. The Power of Programming 2014: International Conference on Developmental Origins of Adiposity and Long-Term Health; 2014 March 13-15; Munich, Germany. 2014 30. McGowan CA, Walsh JM, Byrne J, Curran S, McAuliffe FM. The influence of a low glycemic index dietary intervention on maternal dietary intake, glycemic index and gestational weight gain during pregnancy: a randomized controlled trial. Nutrition Journal 2013;12(1):140 31. Moses RG, Casey S, Cleary J, Milosavljevic M, Quinn E, Tapsell L, et al. Effect of low glycaemic index dietary advice in normal pregnancy: The PREGGIO study. Obesity Research and Clinical Practice 2013;7:e34–5 32. Moses RG, Casey SA, Quinn EG, Cleary JM, Tapsell LC, Milosavljevic M, et al. Pregnancy and Glycemic Index Outcomes study: effects of low glycemic index compared with conventional dietary advice on selected pregnancy outcomes. American Journal of Clinical Nutrition 2014;99 (3):517–23 33. Moses RG, Luebcke M, Davis WS, Coleman KJ, Tapsell LC, Petocz P, et al. Effect of a low-glycemic-index diet during pregnancy on obstetric outcomes. American Journal of Clinical Nutrition 2006;84(4):807–12 34. Moses RG, Luebke M, Petocz P, Brand-Miller JC. Maternal diet and infant size 2 y after the completion of a study of a low-glycemic-index diet in pregnancy. American Journal of Clinical Nutrition 2007;86(6):1806 35. Niinivirta K, Isolauri E, Laakso P, Linderborg K, Laitinen K. Dietary counseling to improve fat quality during pregnancy alters maternal fat intake and infant essential fatty acid status. Journal of Nutrition 2011;141(7):1281–5 14. Niinivirta K, Laakso P, Linderborg K, Poussa T, Isolauri E, Laitinen K. Maternal dietary counseling during pregnancy and infant fatty acid profiles. International Journal of Food Sciences and Nutrition 2014;65(3):268–72 36. Piirainen T, Isolauri E, Lagstrom H, Laitinen K. Impact of dietary counselling on nutrient intake during pregnancy: a prospective cohort study. British Journal of Nutrition 2006; 96(6):1095–104 37. Quinlivan J. A randomised trial of a multidisciplinary teamcare approach involving obstetric, dietary and clinical psychological input in obese pregnant women to reduce the incidence of gestational diabetes. https:// www.anzctr.org.au/Trial/Registration/TrialReview.aspx?id= 821 (accessed 2 November 2015) 38. Quinlivan JA, Lam LT, Fisher J. A randomised trial of a four-step multidisciplinary approach to the antenatal care of obese pregnant women. Australian and New Zealand Journal of Obstetrics and Gynaecology 2011;51(2):141–6 39. Thornton YS, Smarkola C, Kopacz SM, Ishoof SB. Perinatal outcomes in nutritionally monitored obese pregnant women: a randomized clinical trial. Journal of the National Medical Association 2009;101(6):569–77 30. Vitolo MR, Fraga Bueno MS, Mendes Gama C. Impact of a dietary counseling program on the gain weight speed of pregnant women attended in a primary care service. Revista Brasileira de Ginecologia e Obstetricia 2011;33(1):13–9 40. Vahamiko S, Isolauri E, Laitinen K. Weight status and dietary intake determine serum leptin concentrations in pregnant and lactating women and their infants. British Journal of Nutrition 2013;110(6):1098–106 41. Walsh J, Mahony R, Foley M, Mc Auliffe F. A randomised control trial of low glycaemic index carbohydrate diet versus no dietary intervention in the prevention of recurrence of macrosomia. BMC Pregnancy and Childbirth 2010;10:16 42. Walsh J, Mahony R, Foley M, McAuliffe F. ROLO study: a randomized control trial of low glycemic index diet to prevent macrosomia in euglycemic women. American Journal of Obstetrics and Gynecology 2012;206(Suppl 1):S4 43. Walsh J, McGowan C, Byrne J, Foley M, Mahony R, McAuliffe F. The influence of a low glycaemic index dietary intervention on maternal glycaemic index, dietary intake and gestational weight gain. American Journal of Obstetrics and Gynecology 2013;208(1 Suppl):S33 44. Walsh JM, Mahony RM, Canty G, Foley ME, McAuliffe FM. Identification of those most likely to benefit from a low-glycaemic index dietary intervention in pregnancy. British Journal of Nutrition 2014;112:583–9 45. Walsh JM, Mahony RM, Culliton M, Foley ME, McAuliffe FM. Impact of a low glycemic index diet in pregnancy on markers of maternal and fetal metabolism and inflammation. Reproductive Sciences 2014;21(11):1378–81 46. Walsh JM, McAuliffe FM. Impact of maternal nutrition on pregnancy outcome - Does it matter what pregnant women eat? Best Practice and Research. Clinical Obstetrics and Gynaecology 2015;29(1):63–78 47. Walsh JM, McGowan CA, Mahony R, Foley ME, McAuliffe FM. Low glycaemic index diet in pregnancy to prevent macrosomia (ROLO study): randomised control trial. BMJ 2012;345:e5605. 48. Wolff S, Legarth J, Vangsgaard K, Toubro S, Astrup A. A randomized trial of the effects of dietary counseling on gestational weight gain and glucose metabolism in obese pregnant women. International Journal of Obesity 2008;32 (3):495–501 |
| Tombers *et al.* 2023 | 1. Akmes¸e ZB, Oran NT. Effects of progressive muscle relaxation exercises accompanied by music on low back pain and quality of life during pregnancy. J Midwifery Womens Health. 2014;59(5):503–509. doi:10.1111/jmwh.12176. 2. Backhausen MG, Tabor A, Albert H, Rosthøj S, Damm P, Hegaard HK. The effects of an unsupervised water exercise program on low back pain and sick leave among healthy pregnant women - A randomised controlled trial. PLoS One. 2017;12(9):e0182114. doi:10.1371/journal.pone.0182114 3. Mohseni-Bandpei MA, Ahmadshirvani M, Fakhri M, Rahmani N. The effect of an exercise program and ergonomic advices in the treatment of pregnancy related low back pain: a randomised controlled clinical trial. J Mazandaran Univ Med Sci. 2010;20(77):9–17. 4. Eggen MH, Stuge B, Mowinckel P, Jensen KS, Hagen KB. Can supervised group exercises including ergonomic advice reduce the prevalence and severity of low back pain and pelvic girdle pain in pregnancy? A randomized controlled trial. Phys Ther. 2012;92(6):781–790. doi:10.2522/ptj. 20110119. 5. Figueira H, Vale R, Rodrigues W, Figueira A, Figueira J, Dantas E. Pregnancyrelated low back pain relief after maximum static flexibility program. Health. 2014;6(21):2966–2972. doi:10.4236/health.2014.621335 6. Filipec M, Matijevic R. Expert advice about therapeutic exercise during pregnancy reduces the symptoms of sacroiliac dysfunction. J Perinat Med. 2020;48(6):559–565. doi:10.1515/jpm-2020-0143. 7. Gil V, Osis M, Faundes A. Lumbar pain during pregnancy: efficacy of Global Postural Reeducation (GPR) treatment. Fisioter Pesqui. 2011;18(2) doi:10.1590/S1809-29502011000200011. 8. Kamali M, Jafari E, Mazloom Zadeh S. The effect of “sitting pelvic tilt exercise” on low back pain and sleep disorder in primigravidas during the third trimester. Qom Univ Med Sci J. 2009;3(3):45–50. 9. Kihlstrand M, Stenman B, Nilsson S, Axelsson O. Water-gymnastics reduced the intensity of back/low back pain in pregnant women. Acta Obstet Gynecol Scand. 1999;78(3):180–185. 10. Kluge J, Hall D, Louw Q, Theron G, Grové D. Specific exercises to treat pregnancy-related low back pain in a South African population. Int J Gynaecol Obstet. 2011;113(3):187–191. doi:10.1016/j.ijgo.2010.10.030. 11. Nilsson-Wikmar L, Holm K, Oijerstedt R, Harms-Ringdahl K. Effect of three different physical therapy treatments on pain and activity in pregnant women with pelvic girdle pain: a randomized clinical trial with 3, 6, and 12 months follow-up postpartum. Spine (Phila Pa 1976). 2005;30(8):850–856. doi:10.1097/01. Brs.0000158870.68159.d9. 12. Ozdemir S, Bebis H, Ortabag T, Acikel C. Evaluation of the efficacy of an exercise program for pregnant women with low back and pelvic pain: a prospective randomized controlled trial. J Adv Nurs. 2015;71(8):1926–1939. doi:10.1111/ jan.12659. 13. Podeˇbradská R, Šarmírová M, Janura M, Elfmark M, Procházka M. The effect of physiotherapy intervention on the load of the foot and low back pain in pregnancy. Ceska Gynekol. 2019;84(6):450–457. 14. Sayed Mohsen M, Hajar S, Fatemeh M, Maryam Z. Effects of isometric exercise on the alleviation of lumbar and pelvic pain in pregnant women resident in Isfahan, Iran. J Rehabil Res Dev. 2012;7(5). doi:10.22122/jrrs.v7i5.365 15. Sedaghati P, Ziaee V, Ardjmand A. The effect of an ergometric training program on pregnants’ weight gain and low back pain. Gazetta Medica Italiana. 2007;166(6):209–213. 16. Shim MJ, Lee YS, Oh HE, Kim JS. Effects of a back-pain-reducing program during pregnancy for Korean women: a non-equivalent control-group pretest-posttest study. Int J Nurs Stud. 2007;44(1):19–28. doi:10.1016/j. Ijnurstu.2005.11.016. 17. Smith SA, Michel Y. A pilot study on the effects of aquatic exercises on discomforts of pregnancy. J Obstet Gynecol Neonatal Nurs. 2006;35(3):315–323. doi:10.1111/j.1552-6909.2006.00045.x. 18. Suputtitada A, Wacharapreechanont T, Chaisayan P. Effect of the “sitting pelvic tilt exercise” during the third trimester in primigravidas on back pain. J Med Assoc Thai. 2002;85(Suppl 1):S170–S179. 19. Yousefabadi SR, Sarani A, Arbabshastan ME, Adineh HA, Shahnavazi M. The effect of exercise on back pain and lordosis in the second trimester of pregnancy. Drug Invention Today. 2019;11(9):2169–2175. |
| Turan Alagoz *et al.* 2023 | 1. Ghandali NY, Iravani M, Habibi A, Cheraghian B. The effectiveness of a pilates exercise program during pregnancy on childbirth outcomes: a randomised controlled clinical trial. BMC Pregnancy Childbirth. 2021;21:1–11 2. Batool A, Kousar Z, Tariq M, Saleem M, Anwer N, Aslam J. Comparison of aerobic and pilates exercises on depression and sleep quality in primigravida females. Pakistan J Med Health Sci. 2023;17(11):27 3. Bulguroglu M, Bulguroglu HI. The effects of online pilates on the mood and fear of birth of pregnant women: a randomized controlled study. Sci Rep. 2024;14(1):16143 4. Güder DS. The effect of childbirth preparation training on primiparous women’s birth plans and the childbirth processes. 2019 5. Hyun A-H, Jeon Y-J. Effect of mat pilates on body fluid composition, pelvic stabilization, and muscle damage during pregnancy. Appl Sci. 2020;10(24):9111. 6. Manca A, Meloni M, Ventura L, Consoli G, Fenu S, Mura G et al. DoseResponse effects on LBP, sleep, and depression of online Pre-Partum and Post-Partum Pilates-Based programs. Res Q Exerc Sport. 2024:1–10. 7. Nascimento GRSd F, PdMVWd VSO, Ayach W, Christofoletti G, Pegorare ABGS. Effect of pilates exercises on blood glucose, urinary problems, sexual symptoms, pain and anxiety in women with gestational diabetes mellitus. Hum Mov. 2021. 8. Sonmezer E, Özköslü MA, Yosmaoğlu HB. The effects of clinical pilates exercises on functional disability, pain, quality of life and lumbopelvic stabilization in pregnant women with low back pain: A randomized controlled study. J Back Musculoskelet Rehabil. 2021;34(1):69–76. 9. Yıldırım P, Basol G, Karahan AY. Pilates-based therapeutic exercise for pregnancy-related low back and pelvic pain: A prospective, randomized, controlled trial. Turkish J Phys Med Rehabilitation. 2022;69(2):207. 10. Aktan B, Kayıkçıoğlu F, Akbayrak T. The comparison of the effects of clinical pilates exercises with and without childbirth training on pregnancy and birth results. Int J Clin Pract. 2021;75(10):e14516. 11. Buran G, Erim S. The effect of pregnancy pilates-assisted childbirth Preparation training on urinary incontinence and birth outcomes: a randomizedcontrolled study. Arch Gynecol Obstet. 2024;310(5):2725–35. 12. Dias NT, Ferreira LR, Fernandes MG, Resende APM, Pereira-Baldon VS. A pilates exercise program with pelvic floor muscle contraction: is it effective for pregnant women? A randomized controlled trial. Neurourol Urodyn. 2018;37(1):379–84. 13. Feria-Ramírez C, Gonzalez-Sanz JD, Molina-Luque R, Molina-Recio G. The effects of the pilates method on pelvic floor injuries during pregnancy and childbirth: A quasi-experimental study. Int J Environ Res Public Health. 2021;18(13):6995. 14. Mazzarino M, Kerr D, Morris ME. Feasibility of pilates for pregnant women: A randomised trial. J Bodyw Mov Ther. 2022;32:207–12.Pavithralochani V, Thangavignesh R, Saranya P, Ramanathan K. Efficacy of Kegel’s exercise vs pilates in subject with urinary incontinence during pregnancy. Res J Pharm Technol. 2019;12(12):5943–6. 15. Oktaviani I. Pilates workouts can reduce pain in pregnant women. Complement Ther Clin Pract. 2018;31:349–51. 16. Arksey H, O’Malley L. Scoping studies: towards a methodological framework. Int J Soc Res Methodol. 2005;8(1):19–32. 17. Tricco AC, Lillie E, Zarin W, O’Brien KK, Colquhoun H, Levac D, et al. PRISMA extension for scoping reviews (PRISMA-ScR): checklist and explanation. Ann Intern Med. 2018;169(7):467–73. 18. Hyun A-H, Cho J-Y, Koo J-H, editors. Effect of home-based tele-Pilates intervention on pregnant women: a pilot study. Healthcare: MDPI; 2022. 19. Kim H-B, Hyun A-H. Psychological and biochemical effects of an online pilates intervention in pregnant women during COVID-19: a randomized pilot study. Int J Environ Res Public Health. 2022;19(17):10931 20. Urer E, Ozen N, Terzioglu F. Effect of pilates performed during pregnancy on postpartum stress urinary incontinence in Primipara women. J Bodyw Mov Ther. 2023;35:228–32 |
| Vincze et al. 2019 | 1. Bertz F, Brekke HK, Ellega ̊rd L, Rasmussen KM, Wennergren M, Winkvist A. Diet and exercise weight-loss trial in lactating overweight and obese women. Am J Clin Nutr 2012;96(4): 698–705. 2. Bosaeus M, Hussain A, Karlsson T, Andersson L, Hulthen L, Svelander C, et al. A randomized longitudinal dietary intervention study during pregnancy: effects on fish intake, phospholipids, and body composition. Nutr J 2015;14:1. 3. Brekke HK, Bertz F, Rasmussen KM, Bosaeus I, Ellegard L, Winkvist A. Diet and exercise interventions among overweight and obese lactating women: Randomized trial of effects on cardiovascular risk factors. PloS One 2014;9(2):e88250. 4. Carlo C, Iannotti G, Sparice S, Chiacchio MP, Greco E, Tommaselli GA, et al. The role of a personalized dietary intervention in managing gestational weight gain: A prospective, controlled study in a low-risk antenatal population. Arch Gynecol Obstet 2014;289(4):765–70. 5. Colleran HL, Lovelady CA. Use of MyPyramid Menu Planner for Moms in a weight-loss intervention during lactation. J Acad Nutr Diet 2012;112(4):553–8. 66. Colleran HL, Wideman L, Lovelady CA. Effects of energy restriction and exercise on bone mineral density during lactation. Med Sci Sport Ex 2012;44(8):1570–9 6. Craigie AM, Macleod M, Barton KL, Treweek S, Anderson AS. Supporting postpartum weight loss in women living in deprived communities: design implications for a randomised control trial. Eur J Clin Nutr 2011;65(8):952– 8. 7. Davenport MH, Girouox I, Sopper MM, Mottola MF. Postpartum Exercise Regardless of Intensity Improves Chronic Disease Risk Factors. Med Sci Sports Exerc 2011;43(6):951–8. 8. Deveer R, Deveer M, Akbaba E, Engin-U ̈stu ̈ n Y, Aydo?an P, Celikkaya H, et al. The effect of diet on pregnancy outcomes among pregnant with abnormal glucose challenge test. Eur Rev Med Pharmacol Sci 2013;17(9):1258–61. 9. Dodd JM, Kannieappan LM, Grivell RM, Deussen AR, Moran LJ, Yelland LN, et al. Effects of an antenatal dietary intervention on maternal anthropometric measures in pregnant women with obesity. Obesity 2015;23(8):1555–62. 10. Dodd JM, Turnbull D, McPhee AJ, Deussen AR, Grivell RM, Yelland LN, et al. Antenatal lifestyle advice for women who are overweight or obese: LIMIT randomised trial. BMJ 2014;348:g1285. 11. Gray-Donald K, Robinson E, Collier A, David K, Renaud L, Rodrigues S. Intervening to reduce weight gain in pregnancy and gestational diabetes mellitus in Cree communities: an evaluation. Can Med Assoc J 2000;163(10):1247–51. 12. Guelinckx I, Devlieger R, Mullie P, Vansant G. Effect of lifestyle intervention on dietary habits, physical activity, and gestational weight gain in obese pregnant women: a randomized controlled trial. Am J Clin Nutr 2010;91(2):373–80. 13. Harrison CL, Lombard CB, Strauss BJ, Teede HJ. Optimizing healthy gestational weight gain in women at high risk of gestational diabetes: a randomized controlled trial. Obesity 2013;21(5):904–9. 14. Harrison CL, Lombard CB, Teede HJ. Limiting postpartum weight retention through early antenatal intervention: the HeLP-her randomised controlled trial. Int J Behav Nutr Phys Act 2014;11:134. 15. Herring SJ, Cruice JF, Bennett GG, Davey A, Foster GD. Using Technology to Promote Postpartum Weight Loss in Urban, Low-Income Mothers: A Pilot Randomized Controlled Trial. J Nutr Educ Behav 2014;46(6):610–5. 16. Herring SJ, Cruice JF, Bennett GG, Rose MZ, Davey A, Foster GD. Preventing excessive gestational weight gain among African American women: A randomized clinical trial. Obesity 2016;24(1):30–6. 17. Huang T-t, Yeh C-Y, Tsai Y-C. A diet and physical activity intervention for preventing weight retention among Taiwanese childbearing women: a randomised controlled trial. Midwifery 2011;27(2):257–64. Ilmonen J, Isolauri E, Poussa T, Laitinen K. Impact of dietary counselling and probiotic intervention on maternal anthropometric measurements during and after pregnancy: A randomized placebo-controlled trial. Clinical Nutrition 2011;30(2):156–64. 18. Hui A, Back L, Ludwig S, Gardiner P, Sevenhuysen G, Dean H, et al. Lifestyle intervention on diet and exercise reduced excessive gestational weight gain in pregnant women under a randomised controlled trial. BJOG 2012;119(1): 70–7. 19. Hui AL, Back L, Ludwig S, Gardiner P, Sevenhuysen G, Dean HJ, et al. Effects of lifestyle intervention on dietary intake, physical activity level, and gestational weight gain in pregnant women with different pre-pregnancy Body Mass Index in a randomized control trial. BMC Pregnancy Childbirth 2014;14:331. 20. Huseinovic E, Winkvist A, Bertz F, Berte ́us Forslund H, Brekke HK. Eating frequency, energy intake and body weight during a successful weight loss trial in overweight and obese postpartum women. Eur J Clin Nut 2014;68(1):71–6. 21. Jaakkola J, Isolauri E, Poussa T, Laitinen K. Benefits of repeated individual dietary counselling in long-term weight control in women after delivery. Maternal & Child Nutrition 2015;11:1041–8. 22. Kinnunen TI, Raitanen J, Aittasalo M, Luoto R. Preventing excessive gestational weight gain-a secondary analysis of a cluster-randomised controlled trial. Eur J Clin Nut 2012;66(12): 1344–50. 23. Leermakers EA, Anglin K, Wing RR. Reducing postpartum weight retention through a correspondence intervention. Int J Obes Relat Metab Disord 1998;22(11):1103–9 24. Lovelady CA, Garner KE, Moreno KL, Williams JP. The effect of weight loss in overweight, lactating women on the growth of their infants. New Engl J Med 2000;342(7):449–53. 25. Martin J, MacDonald-Wicks L, Hure A, Smith R, Collins CE. Reducing postpartum weight retention and improving breastfeeding outcomes in overweight women: A pilot randomised controlled trial. Nutrients 2015;7(3):1465–79 26. O’Toole ML, Sawicki MA, Artal R. Structured diet and physical activity prevent postpartum weight retention. J Womens Health 2003;12(10):991–8. 27. Ostbye T, Krause KM, Lovelady CA, Morey MC, Bastian LA, Peterson BL, et al. Active Mothers Postpartum. A Randomized Controlled Weight-Loss Intervention Trial. Am J Prev Med 2009;37(3):173–80. 28. Petrella E, Malavolti M, Bertarini V, Pignatti L, Neri I, Battistini NC, et al. Gestational weight gain in overweight and obese women enrolled in a healthy lifestyle and eating habits program. J Matern Fetal Neonatal Med 2014;27(13): 1348–52. 29. Phelan S, Phipps MG, Abrams B, Darroch F, Grantham K, Schaffner A, et al. Does behavioral intervention in pregnancy reduce postpartum weight retention? Twelve-month outcomes of the Fit for Delivery randomized trial. Am J Clin Nut 2014;99(2):302–11. 30. Phelan S, Phipps MG, Abrams B, Darroch F, Schaffner A, Wing RR. Randomized trial of a behavioral intervention to prevent excessive gestational weight gain: the Fit for Delivery Study. Am J Clin Nutr 2011;93(4):772–9. 31. Pollak KI, Alexander SC, Bennett G, Lyna P, Coffman CJ, Bilheimer A, et al. Weight-related SMS texts promoting appropriate pregnancy weight gain: A pilot study. Patient Educ Couns 2014;97(2):256–60. 32. Polley B, Wing R, Sims C. Randomized controlled trial to prevent excessive weight gain in pregnant women. Int J Obesity 2002;26(11):1494–502 33. Rauh K, Gabriel E, Kerschbaum E, Schuster T, Kries R, AmannGassner U, et al. Safety and efficacy of a lifestyle intervention for pregnant women to prevent excessive maternal weight gain: A cluster-randomized controlled trial. BMC Pregnancy Childbirth 2013;13:151. 34. Rauh K, Gunther J, Kunath J, Stecher L, Hauner H. Lifestyle intervention to prevent excessive maternal weight gain: mother and infant follow-up at 12 months postpartum. BMC Pregnancy Childbirth 2015;15:265. 35. Renault KM, Norgaard K, Nilas L, Carlsen EM, Cortes D, Pryds O, et al. The Treatment of Obese Pregnant Women (TOP) study: A randomized controlled trial of the effect of physical activity intervention assessed by pedometer with or without dietary intervention in obese pregnant women. Am Journal Obstet Gynecol 2014;210(2). 134.e1-.e9. 36. Shyam S, Arshad F, Abdul Ghani R, Wahab NA, Safii NS, Nisak MYB, et al. Low glycaemic index diets improve glucose tolerance and body weight in women with previous history of gestational diabetes: a six months randomized trial. Nutr J 2013;12:68-. Asbee SM, Jenkins TR, Butler JR, White J, Elliot M, Rutledge A. Preventing excessive weight gain during pregnancy through dietary and lifestyle counseling: a randomized controlled trial. Obstet Gynecol 2009;113(2 Pt 1):305–12. 37. Stendell-Hollis NR, Thompson PA, West JL, Wertheim BC, Thomson CA. A comparison of Mediterranean-style and MyPyramid diets on weight loss and inflammatory biomarkers in postpartum breastfeeding women. J Womens Health 2013;22(1):48–57. 38. Vesco KK, Karanja N, King JC, Gillman MW, Leo MC, Perrin N, et al. Efficacy of a group-based dietary intervention for limiting gestational weight gain among obese women: A randomized trial. Obesity 2014;22(9):1989–96. 39. Vinter CA, Jensen DM, Ovesen P, Beck-Nielsen H, Jørgensen JS. The LiP (Lifestyle in Pregnancy) study: a randomized controlled trial of lifestyle intervention in 360 obese pregnant women. Diabetes Care 2011;34(12):2502– 7. 40. Vinter CA, Jensen DM, Ovesen P, Beck-Nielsen H, Tanvig M, Lamont RF, et al. Postpartum weight retention and breastfeeding among obese women from the randomized controlled Lifestyle in Pregnancy (LiP) trial. Acta Obstet Gynecol Scand 2014;93(8):794–801. 41. Wilkinson SA, Pligt P, Gibbons KS, McIntyre HD. Trial for Reducing Weight Retention in New Mums: a randomised controlled trial evaluating a low intensity, postpartum weight management programme. J Hum Nutr Diet 2015;28(Suppl 1):15–28. 42. Wiltheiss GA, Lovelady CA, West DG, Brouwer RJ, Krause KM, Østbye T. Diet quality and weight change among overweight and obese postpartum women enrolled in a behavioral intervention program. J Acad Nutr Diet 2013;113(1): 54–62 43. Wolff S, Legarth J, Vangsgaard K, Toubro S, Astrup A. A randomized trial of the effects of dietary counseling on gestational weight gain and glucose metabolism in obese pregnant women. Int J Obesity 2008;32(3):495– 501. |
| Walker et al. 2018 | 1. Abdel-Aziz S., Hegazy I., Mohamed D., Abu El Kasem M., Hagag S. Effect of dietary counseling on preventing excessive weight gain during pregnancy. Public Health. 2018;154:172–181. doi: 10.1016/j.puhe.2017.10.014. 2. Althuizen E., van der Wijden C., van Mechelen W., Seidell J., van Poppel M. The effect of a counselling intervention on weight changes during and after pregnancy: A randomised trial. BJOG. 2013;120:92–99. doi: 10.1111/1471-0528.12014. 3. Asbee S., Jenkins T., Butler J., White J., Elliot M., Rutledge A. Preventing excessive weight gain during pregnancy through dietary and lifestyle counseling: A randomized controlled trial. Obstet. Gynecol. 2009;113:305–312. doi: 10.1097/AOG.0b013e318195baef. 4. Aşcı Ö., Rathfisch G. Effect of lifestyle interventions of pregnant women on their dietary habits, lifestyle behaviors, and weight gain: A randomized controlled trial. J. Health Popul. Nutr. 2016;35:1–9. doi: 10.1186/s41043- 016-0044-2. 5. Bacchi M., Mottola M., Perales M., Refoyo I., Barakat R. Aquatic activities during pregnancy prevent excessive maternal weight gain and preserve birth weight: A randomized clinical trial. Am. J. Health Promot. 2018;32:729–735. doi: 10.1177/0890117117697520. 6. Barakat R, Cordero Y, Coteron J, Luaces M, Montejo R. Exercise during pregnancy improves maternal glucose screen at 24–28 weeks: a randomised controlled trial. Br J Sports Med. 2012; 46: 656– 61 7. Barakat R., Cordero Y., Coteron J., Luaces M., Montejo R. Exercise during pregnancy improves maternal glucose screen at 24–28 weeks: A randomised controlled trial. Br. J. Sports Med. 2012;46:656–661. doi: 10.1136/bjsports-2011-090009. 8. Barakat R., Franco E., Perales M., Lopez C., Mottola M. Exercise during pregnancy is associated with shorter duration of labor: A randomized clinical trial. Eur. J. Obstet. Gynecol. Reprod. Biol. 2018;224:33–40. doi: 10.1016/j.ejogrb.2018.03.009 9. Barakat R., Lucia A., Ruiz J. Resistance exercise training during pregnancy and newborn’s birth size: A randomised controlled trial. Int. J. Obes. 2009;33:9. doi: 10.1038/ijo.2009.150. 10. Barakat R., Pelaez M., Cordero Y., Perales M., Lopez C., Coteron J., Mottola M.F. Exercise during pregnancy protects against hypertension and macrosomia: Randomized clinical trial. Am. J. Obstet. Gynecol. 2016;214:649- e1. doi: 10.1016/j.ajog.2015.11.039. 11. Barakat R., Pelaez M., Lopez C., Lucia A., Ruiz J.R. Exercise during pregnancy and gestational diabetes-related adverse effects: A randomised controlled trial. Br. J. Sports Med. 2013;47:630–636. doi: 10.1136/bjsports-2012- 091788. 12. Barakat R., Pelaez M., Montejo R., Luaces M., Zakynthinaki M. Exercise during pregnancy improves maternal health perception: A randomized controlled trial. Am. J. Obstet. Gynecol. 2011;204:402. doi: 10.1016/j.ajog.2011.01.043 13. Barakat R., Pelaez M., Montejo R., Refoyo I., Coteron J. Exercise throughout pregnancy does not cause preterm delivery: A randomized, controlled trial. J. Phys. Act. Health. 2014;11:1012–1017. doi: 10.1123/jpah.2012- 0344. 14. Bechtel-Blackwell D. Computer-assisted self-interview and nutrition education in pregnant teens. Clin. Nurs. Res. 2002;11:450–462. doi: 10.1177/105477302237456. Obes. 2013;37:814–821. doi: 10.1038/ijo.2012.162. 15. Bisson M., Almeras N., Dufresne S., Robitaille J., Rheaume C., Bujold E., Frenette J., Tremblay A., Marc I. A 12-week exercise program for pregnant women with obesity to improve physical activity levels: An open randomised preliminary study. PLoS ONE. 2015;10:e0137742. doi: 10.1371/journal.pone.0137742. 16. Bogaerts A., Devlieger R., Nuyts E., Witters I., Gyselaers W., Van den Bergh B. Effects of lifestyle intervention in obese pregnant women on gestational weight gain and mental health: A randomized controlled trial. Int. J. 17. Bonomo M., Corica D., Mion E., Goncalves D., Motta G., Merati R., Ragusa A., Morabito A. Evaluating the therapeutic approach in pregnancies complicated by borderline glucose intolerance: A randomized clinical trial. Diabet. Med. 2005;22:1536–1541. doi: 10.1111/j.1464-5491.2005.01690.x. 18. Bosaeus M., Hussain A., Karlsson T., Andersson L., Hulthen L., Svelander C., Sandberg A.S., Larsson I., Ellegard L., Holmang A. A randomized longitudinal dietary intervention study during pregnancy: Effects on fish intake, phospholipids, and body composition. Nutrition. 2015;14:1. doi: 10.1186/1475-2891-14-1. 19. Brownfoot F., Davey M., Kornman L., Brownfoot F. Routine weighing to reduce excessive antenatal weight gain: A randomised controlled trial. BJOG. 2016;123:254–261. doi: 10.1111/1471-0528.13735. 5. Clapp J., Kim H., Burciu B., Schmidt S., Petry K., Lopez B. Continuing regular exercise during pregnancy: Effect of exercise volume on fetoplacental growth. Am. J. Obstet. Gynecol. 2002;186:142–147. doi: 10.1067/mob.2002.119109. 20. Bruno R., Petrella E., Bertarini V., Pedrielli G., Neri I., Facchinetti F. Adherence to a lifestyle programme in overweight/obese pregnant women and effect on gestational diabetes mellitus: A randomized controlled trial. Matern. Child Nutr. 2017;13 doi: 10.1111/mcn.12333. 21. Cahill A., Haire-Joshu D., Cade W., Stein R., Woolfolk C., Moley K., Mathur A., Schechtman K., Klein S. Weight control program and gestational weight gain in disadvantaged women with overweight or obesity: A randomized clinical trial. Obesity. 2018;26:485–491. doi: 10.1002/oby.22070. 22. Cavalcante S., Cecatti J., Pereira R., Baciuk E., Bernardo A., Silveira C. Water aerobics II: Maternal body composition and perinatal outcomes after a program for low risk pregnant women. Reprod. Health. 2009;6:1. doi: 10.1186/1742-4755-6-1. 23. Clapp J., Kim H., Burciu B., Lopez B. Beginning regular exercise in early pregnancy: Effect on fetoplacental growth. Am. J. Obstet. Gynecol. 2000;183:1484–1488. doi: 10.1067/mob.2000.107096. 24. da Silva S., Hallal P., Domingues M., Bertoldi A., Silveira M., Bassani D., da Silva I.C., da Sliva B.C., Coll C.D., Evenson K. A randomized controlled trial of exercise during pregnancy on maternal and neonatal outcomes: Results from the PAMELA study. Int. J. Behav. Nutr. Phys. Act. 2017;14 doi: 10.1186/s12966-017-0632-6. 25. Daley A., Jolly K., Jebb S., Lewis A., Clifford S., Roalfe A., Kenyon S., Aveyard P. Feasibility and acceptability of regular weighing, setting weight gain limits and providing feedback by community midwives to prevent excess weight gain during pregnancy: Randomised controlled trial and qualitative study. BMC Obes. 2015;2:35. doi: 10.1186/s40608-015-0061-5. 26. Daly N., Farren M., McKeating A., O’Kelly R., Stapleton M., Turner M., et al. A medically supervised pregnancy exercise intervention in obese women: A randomized controlled trial. Obstet. Gynecol. 2017;130:1001–1010. doi: 10.1097/AOG.0000000000002267. 27. Dekker Nitert M., Barrett H., Denny K., McIntyre H., Callaway L. Exercise in pregnancy does not alter gestational weight gain, MCP-1 or leptin in obese women. Aust. N. Z. J. Obstet. Gynaecol. 2015;55:27–33. doi: 10.1111/ajo.12300. 28. Deveer R., Deveer M., Akbaba E., Engin-Üstün Y., Aydoǧan P., Çelikkaya H., Danisman N., Mollamahmutoglu L. The effect of diet on pregnancy outcomes among pregnants with abnormal glucose challenge test. Eur. Rev. Med. Pharmacol. Sci. 2013;17:1258–1261. 29. Di Carlo C., Iannotti G., Sparice S., Chiacchio M., Greco E., Tommaselli G., Nappi C. The role of a personalized dietary intervention in managing gestational weight gain: A prospective, controlled study in a low-risk antenatal population. Arch. Gynecol. Obstet. 2014;289:765–770. doi: 10.1007/s00404-013-3054-y. 30. Dodd J., Turnbull D., McPhee A., Deussen A., Grivell R., Yelland L., Crowther C.A., Wittert G., Owens J.A., Robinson J.S. Antenatal lifestyle advice for women who are overweight or obese: LIMIT randomised trial. BMJ. 2014;348:1285. doi: 10.1136/bmj.g1285. 31. Garnæs K., Mørkved S., Salvesen Ø., Moholdt T. Exercise training and weight gain in obese pregnant women: A randomized controlled trial (ETIP Trial) PLoS Med. 2016;13:1–18. doi: 10.1371/journal.pmed.1002079. 32. Garshasbi A., Faghih Zadeh S. The effect of exercise on the intensity of low back pain in pregnant women. Int. J. Gynecol. Obstet. 2005;88:271–275. doi: 10.1016/j.ijgo.2004.12.001. 33. Gesell S., Katula J., Strickland C., Vitolins M. Feasibility and initial efficacy evaluation of a community-based cognitive-behavioral lifestyle intervention to prevent excessive weight gain during pregnancy in Latina women. Matern. Child Health J. 2015;19:1842–1852. doi: 10.1007/s10995-015-1698-x. 34. Guelinckx I., Devlieger R., Mullie P., Vansant G. Effect of lifestyle intervention on dietary habits, physical activity, and gestational weight gain in obese pregnant women: A randomized controlled trial. Am. J. Clin. Nutr. 2010;91:373–380. doi: 10.3945/ajcn.2009.28166. 35. Haakstad L., Bø K. Effect of regular exercise on prevention of excessive weight gain in pregnancy: A randomised controlled trial. Eur. J. Contracept. Reprod. Health Care. 2011;16:116–125. doi: 10.3109/13625187.2011.560307. 36. Haby K., Berg M., Gyllensten H., Hanas R., Premberg A. Mighty Mums—A lifestyle intervention at primary care level reduces gestational weight gain in women with obesity. BMC Obes. 2018;5:16. doi: 10.1186/s40608-018-0194-4. 37. Harrison C., Lombard C., Strauss B., Teede H. Optimizing healthy gestational weight gain in women at high risk of gestational diabetes: A randomized controlled trial. Obesity. 2013;21:904–909. doi: 10.1002/oby.20163. 38. Hawkins M., Hosker M., Marcus B., Rosal M., Braun B., Stanek E., Markenson G., Chasan-Taber L. A pregnancy lifestyle intervention to prevent gestational diabetes risk factors in overweight Hispanic women: A feasibility randomized controlled trial. Diabet. Med. 2015;32:108–115. doi: 10.1111/dme.12601. 39. Herrera-Perdigon J., Hopkins E., Marcalle M., Brooten D., Youngblut J., Lizardo M. Weight gain in high-risk pregnant women: Comparison by primary diagnosis and type of care. Clin. Excell. Nurse Pract. 2005;9:195–201. 40. Herring Cruice J., Bennett G., Rose M., Davey A., Foster G. Preventing excessive gestational weight gain among African American women: A randomized clinical trial. Obesity. 2016;24:30–36. doi: 10.1002/oby.21240. 81. Willcox J., Wilkinson S., Lappas M., Ball K., Crawford D., McCarthy E., Fjeldsoe B.Whittaker R., Maddison R., Campbell K. A mobile health intervention promoting healthy gestational weight gain for women entering pregnancy at a high body mass index: The txt4two pilot randomised controlled trial. BJOG. 2017;124:1718–1728. doi: 10.1111/1471-0528.14552 41. Huang T., Yeh C., Tsai Y. A diet and physical activity intervention for preventing weight retention among Taiwanese childbearing women: A randomised controlled trial. Midwifery. 2011;27:257–264. doi: 10.1016/j.midw.2009.06.009. 42. Hui A., Back L., Ludwig S., Gardiner P., Sevenhuysen G., Dean H., Sellers E., McGavock J., Morris M., Bruce S., et al. Lifestyle intervention on diet and exercise reduced excessive gestational weight gain in pregnant women under a randomized controlled trial. Obstet. Gynecol. Surv. 2012;67:263–264. doi: 10.1097/OGX.0b013e3182561861 43. Hui A., Back L., Ludwig S., Gardiner P., Sevenhuysen G., Dean H., Sellers E., McGavock J., Morris M., Jiang D., et al. Effects of lifestyle intervention on dietary intake, physical activity level, and gestational weight gain in pregnant women with different pre-pregnancy Body Mass Index in a randomized control trial. BMC Pregnancy Childbirth. 2014;14:331. doi: 10.1186/1471-2393-14-331. 44. Ilmonen J., Isolauri E., Poussa T., Laitinen K. Impact of dietary counselling and probiotic intervention on maternal anthropometric measurements during and after pregnancy: A randomized placebo-controlled trial. Clin. Nutr. 2011;30:156–164. doi: 10.1016/j.clnu.2010.09.009. 45. Jackson R., Stotland N., Caughey A., Gerbert B. Improving diet and exercise in pregnancy with Video Doctor counseling: A randomized trial. Patient Educ. Couns. 2011;83:203–209. doi: 10.1016/j.pec.2010.05.019. 46. Jeffries K., Shub A., Walker S., Hiscock R., Permezel M. Reducing excessive weight gain in pregnancy: A randomised controlled trial. Med. J. Aust. 2009;191:429–433. 47. Jing W., Huang Y., Liu X., Luo B., Yang Y., Liao S. The effect of a personalized intervention on weight gain and physical activity among pregnant women in China. Int. J. Gynaecol. Obstet. 2015;129:138–141. doi: 10.1016/j.ijgo.2014.11.014. 48. Kong K., Campbell C., Foster R., Peterson A., Lanningham-Foster L. A pilot walking program promotes moderate-intensity physical activity during pregnancy. Med. Sci. Sports Exerc. 2014;46:462–471. doi: 10.1249/MSS.0000000000000141. 49. Korpi-Hyovalti E., Schwab U., Laaksonen D.E., Linjama H., Heinonen S., Niskanen L. Effect of intensive counselling on the quality of dietary fats in pregnant women at high risk of gestational diabetes mellitus. Br. J. Nutr. 2012;108:910–917. doi: 10.1017/S0007114511006118. 50. Luoto R., Kinnunen T., Aittasalo M., Ojala K., Mansikkamaki K., Toropainen E., Kolu P., Vasankari T. Prevention of gestational diabetes: Design of a cluster-randomized controlled trial and one-year follow-up. BMC Pregnancy Childbirth. 2010;10:39. doi: 10.1186/1471-2393-10-39. 51. Markovic T., Muirhead R., Overs S., Ross G., Louie J., Kizirian N., et al. Randomized Controlled Trial Investigating the Effects of a Low-Glycemic Index Diet on Pregnancy Outcomes in Women at High Risk of Gestational Diabetes Mellitus: The GI Baby 3 Study. Diabetes Care. 2016;39:31–38. doi: 10.2337/dc15-0572. 52. McCarthy E., Walker S., Ugoni A., Lappas M., Leong O., Shub A. Self-weighing and simple dietary advice for overweight and obese pregnant women to reduce obstetric complications without impact on quality of life: A randomised controlled trial. BJOG. 2016;123:965–973. doi: 10.1111/1471-0528.13919. 53. Moses R., Casey S., Quinn E., Cleary J., Tapsell L., Milosavljevic M., Petocz P., Brand-Miller J.C. Pregnancy and Glycemic Index Outcomes study: Effects of low glycemic index compared with conventional dietary advice on selected pregnancy outcomes. Am. J. Clin. Nutr. 2014;99:517–523. doi: 10.3945/ajcn.113.074138. 54. Nascimento S., Surita F., Parpinelli M., Siani S., Pinto e Silva J. The effect of an antenatal physical exercise programme on maternal/perinatal outcomes and quality of life in overweight and obese pregnant women: A randomised clinical trial. BJOG. 2011;118:1455–1463. doi: 10.1111/j.1471-0528.2011.03084.x. 55. Nobles C., Marcus B., Stanek E., Braun B., Whitcomb B., Manson J., et al. The Effect of an Exercise Intervention on Gestational Weight Gain: The Behaviors Affecting Baby and You (B.A.B.Y.) Study: A Randomized Controlled Trial. Am. J. Health Promot. 2018;32:736–744. doi: 10.1177/0890117117732409. 56. Olsen C., Groth S., Graham M., Reschke J., Strawderman M., Fernandez D. The effectiveness of an online intervention in preventing excessive gestational weight gain: The e-moms roc randomized controlled trial. BMC Pregnancy Childbirth. 2018;18:148. doi: 10.1186/s12884-018-1767-4. 57. Oostdam N., Van Poppel M., Wouters M., Eekhoff E., Bekedam D., Kuchenbecker W., Quartero H., Heres M., van Mechelen W. No effect of the FitFor2 exercise programme on blood glucose, insulin sensitivity, and birthweight in pregnant women who were overweight and at risk for gestational diabetes: Results of a randomised controlled trial. BJOG. 2012;119:1098–1107. doi: 10.1111/j.1471-0528.2012.03366.x. 58. Pawalia A., Kulandaivelan S., Savant S., Yadav V. Exercise in pregnancy: Effect on obesity parameters in indian women—A randomized controlled trial. Rom. J. Diabetes Nutr. Metab. Dis. 2017;24:315–323. doi:10.1515/rjdnmd-2017-0037. 59. Peccei A., Blake-Lamb T., Rahilly D., Hatoum I., Bryant A. Intensive prenatal nutrition counseling in a community health setting: A randomized controlled trial. Obstet. Gynecol. 2017;130:423–432. doi: 10.1097/AOG.0000000000002134. 60. Perales M., Cordero Y., Vargas M., Lucia A., Barakat R. Exercise and depression in overweight and obese pregnant women: A randomised controlled trial. Arch. Med. Deporte. 2015;32:156–163. 61. Petrella E., Malavolti M., Bertarini V., Pignatti L., Neri I., Battistini N.C., Facchinetti F. Gestational weight gain in overweight and obese women enrolled in a healthy lifestyle and eating habits program. J. Matern. Neonatal Med. 2014;27:1348–1352. doi: 10.3109/14767058.2013.858318. 62. Phelan S., Phipps M., Abrams B., Darroch F., Schaffner A., Wing R. Randomized trial of a behavioral intervention to prevent excessive gestational weight gain: The Fit for Delivery Study. Am. J. Clin. Nutr. 2011;93:772–779. doi: 10.3945/ajcn.110.005306. 63. Phelan S., Wing R., Brannen A., McHugh A., Hagobian T., Schaffner A., Jelalian E., Hart C.N., Scholl T.O., Munoz-Christian K., et al. Randomized controlled clinical trial of behavioral lifestyle intervention with partial meal replacement to reduce excessive gestational weight gain. Am. J. Clin. Nutr. 2018;107:183–194. doi: 10.1093/ajcn/nqx043. [PMC free article] 64. Polley B., Wing R., Sims C. Randomized controlled trial to prevent excessive weight gain in pregnant women. Int. J. Obes. 2002;26:1494–1502. doi: 10.1038/sj.ijo.0802130. 65. Poston L., Bell R., Croker H., Flynn A., Godfrey K., Goff L., Hayes L., Khazaezadeh N., Nelson S.M., Oteng-Ntim E., et al. Effect of a behavioural intervention in obese pregnant women (the UPBEAT study): A multicentre, randomised controlled trial. Lancet Diabetes Endocrinol. 2015;3:767–777. doi: 10.1016/S2213-8587(15)00227-2. 66. Quinlivan J., Lam L., Fisher J. A randomised trial of a four-step multidisciplinary approach to the antenatal care of obese pregnant women. Aust. N. Z. J. Obstet. Gynaecol. 2011;51:141–146. doi: 10.1111/j.1479- 828X.2010.01268.x. 67. Rauh K., Gabriel E., Kerschbaum E., Schuster T., von Kries R., Amann-Gassner U., Hauner H. Safety and efficacy of a lifestyle intervention for pregnant women to prevent excessive maternal weight gain: A cluster- randomized controlled trial. BMC Pregnancy Childbirth. 2013;13:151. doi: 10.1186/1471-2393-13-151. 68. Renault K., Nørgaard K., Nilas L., Carlsen E., Cortes D., Pryds O., Secher N.J. The Treatment of Obese Pregnant Women (TOP) study: A randomized controlled trial of the effect of physical activity intervention assessed by pedometer with or without dietary intervention in obese pregnant women. Am. J. Obstet. Gynecol. 2014;210:134. doi: 10.1016/j.ajog.2013.09.029. 69. Rhodes E., Pawlak D., Takoudes T., Ebbeling C., Feldman H., Lovesky M., Cooke E.A., Leidig M.M., Ludwig D.S. Effects of a low-glycemic load diet in overweight and obese pregnant women: A pilot randomized controlled trial. Am. J. Clin. Nutr. 2010;92:1306–1315. doi: 10.3945/ajcn.2010.30130 70. Rodríguez-Blanque R., Carlos Sánchez-García J., Manuel Sánchez-López A., Mur-Villar N., Fernández-Castillo R., José Aguilar-Cordero M. Influence of physical exercise during pregnancy on birthweight: A randomized clinical trial. Nutr. Hosp. 2017;34:834–840. doi: 10.20960/nh.1095. 71. Ronnberg A., Ostlund I., Fadl H., Gottvall T., Nilsson K. Intervention during pregnancy to reduce excessive gestational weight gain-a randomised controlled trial. BJOG. 2015;122:537–544. doi: 10.1111/1471-0528.13131. 72. Ruchat S., Davenport M., Giroux I., Hillier M., Batada A., Sopper M., Hammond J.M., Mottola M.F. Nutrition and exercise reduce excessive weight gain in normal-weight pregnant women. Med. Sci. Sports xerc. 2012;44:1419–1426. doi: 10.1249/MSS.0b013e31825365f1. 73. Ruiz J., Perales M., Pelaez M., Lopez C., Lucia A., Barakat R. Supervised exercise-based intervention to prevent excessive gestational weight gain: A randomized controlled trial. Mayo Clin. Proc. 2013;88:1388–1397. doi: 10.1016/j.mayocp.2013.07.020. 74. Sagedal L., Øverby N., Bere E., Torstveit M., Lohne-Seiler H., Småstuen M., Hillesund E.R., Henriksen T., Vistad I. Lifestyle intervention to limit gestational weight gain: The Norwegian Fit for Delivery randomised controlled trial. BJOG. 2016;124:97–109. doi: 10.1111/1471-0528.13862. 75. Santamaria A., Di Benedetto A., Petrella E., Pintaudi B., Corrado F., D’Anna R., Neri I., Facchinetti F. Myo-inositol may prevent gestational diabetes onset in overweight women: A randomized, controlled trial. J. Matern. Fetal Neonatal Med. 2016;29:3234–3237. doi: 10.3109/14767058.2015.1121478. 76. Simmons D., Jelsma J., Galjaard S., Devlieger R., van Assche A., Jans G., Corcoy R., Adelantado J.M., Dunne F., Desoye G., et al. Results from a European multicenter randomized trial of physical activity and/or healthy eating to reduce the risk of gestational diabetes mellitus: The DALI lifestyle pilot. Diabetes Care. 2015;38:1650–1656. doi: 10.2337/dc15-0360. 77. Skouteris H., McPhie S., Hill B., McCabe M., Milgrom J., Kent B., Bruce L., Herring S., Gale J., Mihalopoulos C., et al. Health coaching to prevent excessive gestational weight gain: A randomized-controlled trial. Br. J. Health Psychol. 2016;21:31–51. doi: 10.1111/bjhp.12154. 78. Smith K., Lanningham-Foster L., Welch A., Campbell C. Web-Based Behavioral Intervention Increases Maternal Exercise but Does Not Prevent Excessive Gestational Weight Gain in Previously Sedentary Women. J. Phys. Act. Health. 2016;13:587–593. doi: 10.1123/jpah.2015-0219. 79. Syngelaki A., Nicolaides K., Balani J., Hyer S., Akolekar R., Kotecha R., Pastides A., Shehata H. Metformin versus Placebo in Obese Pregnant Women without Diabetes Mellitus. N. Engl. J. Med. 2016;374:434–443. doi: 10.1056/NEJMoa1509819. 80. Thomson J., Tussing-Humphreys L., Goodman M., Olender S. Gestational weight gain: Results from the delta healthy sprouts comparative impact trial. J. Pregnancy. 2016;2016:12. doi: 10.1155/2016/5703607. 81. Thornton Y., Smarkola C., Kopacz S., Ishoof S. Perinatal outcomes in nutritionally monitored obese pregnant women: A randomized clinical trial. J. Natl. Med. Assoc. 2009;101:569–577. doi: 10.1016/S0027-9684(15)30942- 1. 82. Vesco K., Karanja N., King J., Gillman M., Leo M., Perrin N., McEvoy C.T., Eckhardt C.L., Smith K.S., Stevens C.L. Efficacy of a group-based dietary intervention for limiting gestational weight gain among obese women: A randomized trial. Obesity. 2014;22:1989–1996. doi: 10.1002/oby.20831 83. Vinter C., Jensen D., Ovesen P., Beck-Nielsen H.J.O. Lifestyle and pregnancy (LIP) study: The clinical effect of lifestyle intervention during pregnancy in obese women. Diabetes. 2011;60:348–349. doi: 10.2337/dc11-1150. 84. Vítolo M., Bueno M., Gama C. Impact of a dietary counseling program on the gain weight speed of pregnant women attended in a primary care service. Rev. Bras. Ginecol. Obstet. 2011;33:58–59. doi: 10.1590/S0100-72032011000100009. 85. Walsh J., McGowan C., Mahony R., Foley M., McAuliffe F. Low glycaemic index diet in pregnancy to prevent macrosomia (ROLO study): Randomised control trial. BMJ. 2012;345:e5605. doi: 10.1136/bmj.e5605. 86. Wang C., Wei Y., Zhang X., Zhang Y., Xu Q., Sun Y., Su S., Zhang L., Liu C., Feng Y., et al. A randomized clinical trial of exercise during pregnancy to prevent gestational diabetes mellitus and improve pregnancy outcome in overweight and obese pregnant women. Am. J. Obstet. Gynecol. 2017;216:340–351. doi: 10.1016/j.ajog.2017.01.037. 87. Wang S., Ma J., Yang H. Lifestyle intervention for gestational diabetes mellitus prevention: A cluster-randomized controlled study. Chronic Dis. Transl. Med. 2015;1:169–174. doi: 10.1016/j.cdtm.2015.09.001 88. Wolff S., Legarth J., Vangsgaard K., Toubro S., Astrup A. A randomized trial of the effects of dietary counseling on gestational weight gain and glucose metabolism in obese pregnant women. Int. J. Obes. 2008;32:495–501. doi: 10.1038/sj.ijo.0803710. |
| Wang et al. 2019 | 1. Barakat R, Cordero Y, Coteron J, et al. Exercise during pregnancy improves maternal glucose screen at 24–28 weeks: a randomised controlled trial. Br J Sports Med 2012;46:656–61. 2. Barakat R, Lucia A, Ruiz J. Resistance exercise training during pregnancy and newborn’s birth size: a randomised controlled trial. Int J Obes V 33 20052009;1048–57. 3. Barakat R, Pelaez M, Lopez C, et al. Exercise during pregnancy and gestational diabetes-related adverse effects: a randomised controlled trial. Br J Sports Med 2013;47:630–6. 4. Barakat R, Pelaez M, Montejo R, et al. Exercise during pregnancy improves maternal health perception: a randomized controlled trial. Am J Obstet Gynecol 2011;204:402e1-7. 5. Barakat R, Pelaez M, Montejo R, et al. Exercise throughout pregnancy does not cause preterm delivery: a randomized, controlled trial. J Phys Act Health 2014;11:1012–7 6. Barakat R, Perales M, Bacchi M, et al. A program of exercise throughout pregnancy. Is it safe to mother and newborn? Am J Health Promot 2014;29:2–8. 7. Cavalcante S, Cecatti J, Pereira R, et al. Water aerobics II: maternal body composition and perinatal outcomes after a program for low risk pregnant women. Reprod Health 2009;6:1. 8. Da Silva S, Hallal P, Domingues M, et al. A randomized controlled trial of exercise during pregnancy on maternal and neonatal outcomes: results from the PAMELA study. Int J Behav Nutr Phys Act 2017;14:175. 9. Garshasbi A, Faghih Zadeh S. The effect of exercise on the intensity of low back pain in pregnant women. Int J Gynaecol Obstet 2005;88:271–5. 10. Haakstad L, Bø K. Exercise in pregnant women and birth weight: a randomized controlled trial. BMC Pregnancy Childbirth 2011;11:66. 11. Hopkins S, Baldi J, Cutfield W, et al. Effects of exercise training on maternal hormonal changes in pregnancy. Clin Endocrinol (Oxf) 2011;74:495–500. 12. Kihlstrand M, Stenman B, Nilsson S, et al. Water-gymnastics reduced the intensity of back/low back pain in pregnant women. Acta Obstet GynecolScand 1999;78:180–5. 13. Perales M, Calabria I, Lopez C, et al. Regular exercise throughout pregnancy is associated with a shorter first stage of labor. Am J Health Promot 2016;30:149–54. 14. Polley BA, Wing RR, Sims CJ (2002). Randomized controlled trial to prevent excessive weight gain in pregnant women. Int J Obes Relat Metab Disord; 26(11): 1494–1502. 15. Price B, Amini S, Kappeler K. Exercise in pregnancy: effect on fitness and obstetric outcomes-a randomized trial. Med Sci Sports Exerc 2012;44:2263–9. 16. Ramírez-Vélez R, Aguilar de Plata AC, Mosquera-Escudero M, BS, et al. The effect of aerobic exercise on oxygen consumption in healthy first pregnancy females: a randomized clinical trial. Rev Colomb Obstet Ginecol 2011;62:15–23. 17. Rodríguez YC, Puente MP, Abad MDM, RBC, et al. Can moderate physical exercise during pregnancy act as a factor in preventing Gestational Diabetes? Rev Int Cienc Deporte 2012;8:3–19. 18. Ruiz J, Perales M, Pelaez M, et al. Supervised exercise-based intervention to prevent excessive gestational weight gain: a randomized controlled trial. Mayo Clin Proc 2013;88:1388–97. 19. Sedaghati P, Ziaee V, Ardjmand AA. The effect of an ergometric training program on pregnants’ weight gain and low back pain. Gazz Med Ital 2007;166:209–13. 20. Taniguchi C, Sato C. Home-based walking durig pregnancy affects mood and birth outcomes among sedentary women. In J Nurs Pract 2016; 22(5):420-426 21. Wang C, Wei Y, Zhang X, et al. A randomized clinical trial of exercise during pregnancy to prevent gestational diabetes mellitus and improve pregnancy outcome in overweight and obese pregnant women. Am J Obstet Gynecol 2017;216:340–51. |
| Yin et al. 2013 | 1. Barakat R, Cordero Y, Coteron J, et al. Exercise during pregnancy improves maternal glucose screen at 24–28 weeks: a randomised controlled trial.Br J Sports Med2011;46:656–61. 2. Barakat R, Ruiz JR, Stirling JR,et al. Type of delivery is not affected by light resistance and toning exercise training during pregnancy: a randomized controlled trial. Am J Obstet Gynecol 2009;201:509.e1–6. 3. Callaway LK, Colditz PB, Byrne NM,et al. Prevention of gestational diabetes: feasibility issues for an exercise intervention in obese pregnant women.DiabetesCare2010;33:1457–9. 4. Price BB, Amini SB, Kappeler K. Exercise in pregnancy: effect on fitness and obstetric outcomes—a randomized trial. Med Sci Sports Exerc 2012;44:2263–9. 5. Rakhshani A, Nagarathna R, Mhaskar R, et al. The effects of yoga in prevention of pregnancy complications in high-risk pregnancies: a randomized controlled trial. Prev Med 2012;55:333–40 6. Stafne SN, Salvesen KA, Romundstad PR, et al. Regular exercise during pregnancy to prevent gestational diabetes: a randomized controlled trial. Obstet Gynecol 2012;119:29–36. |
| Zenitasari *et al.*2023 | 1. Yaman, O.B. ¨ Tulmaç, B. Canarslan, N. Hançerliogullari, ˘ Effect of pilates during pregnancy on delivery outcomes, J. Health Sci. Med. 3 (4) (2020) 442–447. 2. Y.A. Reis, A. Akay, B. Aktan, S. Tetik, F.B. Flratllgil, F. Kaylkçloǧlu, The effect of clinical pilates exercises and prenatal education on maternal and fetal health, Z. Geburtshilfe Neonatol. 227 (5) (2023) 354–363. 3. M. Mazzarino, D. Kerr, M.E. Morris, Feasibility of pilates for pregnant women: a randomised trial, J. Bodyw. Mov. Ther. 32 (2022) 207–212. 4. N.Y. Ghandali, M. Iravani, A. Habibi, B. Cheraghian, The effectiveness of a Pilates exercise program during pregnancy on childbirth outcomes: a randomised controlled clinical trial, BMC Pregn. Childb. 21 (1) (2021) 480. 5. B. Aktan, F. Kayıkçıoglu, ˘ T. Akbayrak, The comparison of the effects of clinical Pilates exercises with and without childbirth training on pregnancy and birth results, Int. J. Clin. Pract. 75 (10) (2021) e14516 |
| Zhang *et al.* 2023 | 1. Lu CL, Yu L. Study on the correlation between aerobic exercise during pregnancy and pregnancy outcome. Zhonghua Linchuang Yishi Zazhi 2014;8:3990–3. 2. Bruno R, Petrella E, Bertarini V, Pedrielli G, Neri I, Facchinetti F. Adherence to a lifestyle programme in overweight/obese pregnant women and effect on gestational diabetes mellitus: a randomized controlled trial. Matern Child Nutr 2017;13:e12333. 3. Wang C, Wei Y, Zhang X, Zhang Y, Xu Q, Sun Y, et al. A randomized clinical trial of exercise during pregnancy to prevent gestational diabetes mellitus and improve pregnancy outcome in overweight and obese pregnant women. Am J Obstet Gynecol 2017;216:340–51. 4. Tomić V, Sporiš G, Tomić J, Milanović Z, Zigmundovac-Klaić D, Pantelić S. The effect of maternal exercise during pregnancy on abnormal fetal growth. Croat Med J 2013;54:362–8. 5. Rakhshani A, Nagarathna R, Mhaskar R, Mhaskar A, Thomas A, Gunasheela S. The effects of yoga in prevention of pregnancy complications in high-risk pregnancies: a randomized controlled trial. Prev Med 2012;55:333–40. 6. Barakat R, Cordero Y, Coteron J, Luaces M, Montejo R. Exercise during pregnancy improves maternal glucose screen at 24-28 weeks: a randomised controlled trial. Br J Sports Med 2012;46:656–61. 7. Deng Y, Hou Y, Wu L, Liu Y, Ma L, Yao A. Effects of Diet and Exercise Interventions to Prevent Gestational Diabetes Mellitus in Pregnant Women With High-Risk Factors in China: A Randomized Controlled Study. Clin Nurs Res 2022;31:836–47. 8. Bhartia N, Jain S, Shankar N, Rajaram S, Gupta M. Effects of Antenatal Yoga on Maternal Stress and Clinical Outcomes in North Indian Women: A Randomised Controlled Trial. JIACM 2019;20:10–4. 9. Barakat R, Pelaez M, Cordero Y, Perales M, Lopez C, Coteron J, et al. Exercise during pregnancy protects against hypertension and macrosomia: randomized clinical trial. Am J Obstet Gynecol 2016;214:649.e1–8. 10. da Silva SG, Hallal PC, Domingues MR, Bertoldi AD, Silveira MF, Bassani D, et al. A randomized controlled trial of exercise during pregnancy on maternal and neonatal outcomes: results from the PAMELA study. Int J Behav Nutr Phys Act 2017;14:175. 11. Xu MY, Guo YJ, Zhang LJ, Lu QB. Effect of individualized weight management intervention on excessive gestational weight gain and perinatal outcomes: a randomized controlled trial. PeerJ 2022;10:e13067 |

**Unique publications included in diet and/or physical activity reviews**

**Diet and/or physical activity unique publications**

1. Asci, O. Rathfisch, G. Effect of lifestyle interventions of pregnant women on their dietary habits, lifestyle behaviors, and weight gain: A randomized controlled trial. J. Health Popul. Nutr. 2016, 35, 7
2. Aaltonen J, Ojala T, Laitinen K, Piirainen TJ, Poussa TA, Isolauri E. Evidence of infant blood pressure programming by maternal nutrition during pregnancy: a prospective randomized controlled intervention study. J Pediatr 2008;152:79–84.
3. Abdel-Aziz S., Hegazy I., Mohamed D., Abu El Kasem M., Hagag S. Effect of dietary counseling on preventing excessive weight gain during pregnancy. Public Health. 2018;154:172–181. doi: 10.1016/j.puhe.2017.10.014.
4. Abirami P, Judie A. Integrated approach of yoga therapy on maternal and fetal outcome in gestational diabetes mellitus. International Journal of Pharmaceutical and Clinical Research 2015;7:377–82.
5. Abirami P, Judie A. Reduction of risk on newly detected gestational diabetes mellitus by multi model intervention-A hospital based study. International Journal of Pharmaceutical and Clinical Research 2014;6:370–4.
6. Aguilar-Cordero  MJ, Sánchez-García  JC, Rodriguez-Blanque  R, Sánchez-López  AM, Mur-Villar  N.  Moderate physical activity in an aquatic environment during pregnancy (SWEP study) and its influence in preventing postpartum depression.   J Am Psychiatr Nurses Assoc. 2019;25(2):112-121. doi:10.1177/1078390317753675
7. Aihemaiti R. Prepregnancy overweight/obese pregnant women dietary intervention on dietary intake and pregnancy outcome. Xinjiang Medical University; 2017 [MA thesis].
8. Aittasalo M, Pasanen M, Fogelholm M, Kinnunen TI, Ojala K, Luoto R. Physical activity counseling in maternity and child health care – a controlled trial. BMC Womens Health 2008;14(August 8):14.
9. Aittasalo M, Raitanen J, Kinnunen TI, Ojala K, Kolu P, Luoto R. Is intensive counseling in maternity care feasible and effective in promoting physical activity among women at risk for gestational diabetes? Secondary analysis of a cluster randomized NELLI study in Finland. Int J Behav Nutr Phys Act 2012;5 (September 9):104
10. Akmes¸e ZB, Oran NT. Effects of progressive muscle relaxation exercises accompanied by music on low back pain and quality of life during pregnancy. J Midwifery Womens Health. 2014;59(5):503–509. doi:10.1111/jmwh.12176
11. Aktan B, Kayıkçıoğlu F, Akbayrak T. The comparison of the effects of clinical pilates exercises with and without childbirth training on pregnancy and birth results. Int J Clin Pract. 2021;75(10):e14516.
12. Al Wattar  BH, Dodds  J, Placzek  A,  et al; ESTEEM Study Group.  Mediterranean-style diet in pregnant women with metabolic risk factors (ESTEEM): a pragmatic multicentre randomised trial.   PLoS Med. 2019;16(7):e1002857. doi:10.1371/journal.pmed.1002857PubMedGoogle Scholar
13. Albright CL, Steffen AD, Wilkens LR et al. Effectiveness of a 12-month randomized clinical trial to increase physical activity in multiethnic postpartum women: results from Hawaii’s Na Mikimiki Project. Prev Med 2014; 69: 214–223.
14. Altazan  AD, Redman  LM, Burton  JH,  et al.  Mood and quality of life changes in pregnancy and postpartum and the effect of a behavioral intervention targeting excess gestational weight gain in women with overweight and obesity: a parallel-arm randomized controlled pilot trial.   BMC Pregnancy Childbirth. 2019;19(1):50. doi:10.1186/s12884-019-2196-8PubMedGoogle ScholarCrossref
15. Althuizen E vdWC, van Mechelen W, Seidell JC, van Poppel MNM. The effect of a counselling intervention on weight changes during and after pregnancy: a randomised trial. British Journal of Obstetrics and Gynecology. 2013;120:92-9.
16. Althuizen E, Van Poppel MN, Seidell JC, Van der Wijden C, Van Mechelen W. Design of the new life(style) study: a randomised controlled trial to optimise maternal weight development during pregnancy. BMC Public Health 2006;6:168.
17. Angel MD, De Haene J, Perez M, Hernandez G, Castaneda D, King JC. Dietary patterns associated with gestational weight gain and fat mass gain in overweight and obese pregnant women. FASEB Journal 2011;25:783.15
18. Anleu E, Reyes M, Araya B M, Flores M, Uauy R, Garmendia ML. Effectiveness of an intervention of dietary counseling for overweight and obese pregnant women in the consumption of sugars and energy. Nutrients. 2019; 11(2):385. doi:10.3390/nu11020385
19. Arizabaleta, A.V.M.; Buitrago, L.O.; de Plata, A.C.A.; Escudero, M.M.; Ramirez-Velez, R. Aerobic exercise during pregnancy improves health-related quality of life: A randomised trial. J. Physiother. 2010, 56, 253–258
20. Arksey H, O’Malley L. Scoping studies: towards a methodological framework. Int J Soc Res Methodol. 2005;8(1):19–32.
21. Armstrong, K., Edwards, H., 2004. The effectiveness of a pram-walking exercise programme in reducing depressive symptomatology for postnatal women. Int. J. Nurs. Pract. 10, 177–194.
22. Artal R, Catanzaro R, Gavard J et al. (2007) A lifestyle intervention of weight-gain restriction: diet and exercise in obese women with gestational diabetes mellitus. Appl Physiol Nutr Metab 32:596–601
23. Artal R, Platt LD, Sperling M, et al. I. Maternal cardiovascular and metabolic responses in normal pregnancy. Am J Obstet Gynecol 1981;140:123–7.
24. Artal R, Rutherford S, Romem Y, et al. Fetal heart rate responses to maternal exercise. Am J Obstet Gynecol 1986;155:729–33.
25. Artal R, Wiswell R, Romem Y. Hormonal responses to exercise in diabetic and nondiabetic pregnant patients. Diabetes 1985;34 Suppl 2(Supplement_2):78–80
26. Arthur C, Di Corleto E, Ballard E, Kothari A. A randomized controlled trial of daily weighing in pregnancy to control gestational weight gain. BMC Pregnancy Childbirth. 2020;20(1):223. doi:10.1186/s12884-020-02884-1
27. Asbee  SM, Jenkins  TR, Butler  JR, White  J, Elliot  M, Rutledge  A.  Preventing excessive weight gain during pregnancy through dietary and lifestyle counseling: a randomized controlled trial.   Obstet Gynecol. 2009;113(2, pt 1):305-312. doi:10.1097/AOG.0b013e318195baefPubMedGoogle ScholarCrossref
28. Asbee SM, Jenkins TR, Butler JR, White J, Elliot M, Rutledge A. Dietary counseling prevents excessive weight gain during pregnancy, a randomized controlled trial. Obstetrics & Gynecology 2008;11(4 Suppl):6S.
29. Aşcı Ö., Rathfisch G. Effect of lifestyle interventions of pregnant women on their dietary habits, lifestyle behaviors, and weight gain: A randomized controlled trial. J. Health Popul. Nutr. 2016;35:1–9. doi: 10.1186/s41043- 016-0044-2.
30. Assaf-Balut  C, García de la Torre  N, Durán  A,  et al.  A Mediterranean diet with additional extra virgin olive oil and pistachios reduces the incidence of gestational diabetes mellitus (GDM): a randomized controlled trial: the St. Carlos GDM Prevention Study.   PLoS One. 2017;12(10):e0185873. doi:10.1371/journal.pone.0185873PubMedGoogle Scholar
31. Avery MD, Leon AS, Kopher RA. Effects of a partially home-based exercise program for women with gestational diabetes. Obstet Gynecol. 1997;89(1):10-5.
32. Avery MD, Walker AJ. Acute effect of exercise on blood glucose and insulin levels in women with gestational diabetes. J Matern Fetal Med 2001;10:52–8.
33. Bacchi M., Mottola M., Perales M., Refoyo I., Barakat R. Aquatic activities during pregnancy prevent excessive maternal weight gain and preserve birth weight: A randomized clinical trial. Am. J. Health Promot. 2018;32:729–735. doi: 10.1177/0890117117697520.
34. Bacchi M., Rodríguez Y.C., Puente M.P., Mas C.L., Carballo R. Efecto sobre la frecuencia cardiaca del ejercicio en tierra/agua en embarazadas en el tercer trimestre de gestación: Un estudio comparativo. Calid. Vida Salud. 2011;4:59–67.
35. Backhausen MG, Tabor A, Albert H, Rosthøj S, Damm P, Hegaard HK. The effects of an unsupervised water exercise program on low back pain and sick leave among healthy pregnant women - A randomised controlled trial. PLoS One. 2017;12(9):e0182114. doi:10.1371/journal.pone.0182114
36. Baciuk EP, Pereira RI, Cecatti JG, Braga AF, Cavalcante SR. Water aerobics in pregnancy: Cardiovascular response, labor and neonatal outcomes. Reprod Health. 2008;5:10.
37. Badon SE, Wander PL, Qiu C, et al. Maternal leisure time physical activity and infant birth size. Epidemiology 2016;27:74–81.
38. Badon SE, Wartko PD, Qiu C, et al. Leisure time physical activity and gestational diabetes mellitus in the omega study. Medicine & Science in Sports & Exercise 2016;48:1044–52.
39. Badrawi, M. K. Hassanein, M. H. H. Badraoui, Y. A. Wafa, H. A. Shawky, and N. Badrawi, “Pregnancy outcome in obese pregnant mothers,” Journal of Perinatal Medicine, vol. 20, supplement 1, p. 203, 1992.
40. Baker J. Developing a care pathway for obese women in pregnancy and beyond. Br J Midwifery. 2011;19(10):632–43.
41. Banerjee A, V. Mahalakshmi V, R. Baranitharan R. Effect of Antenatal Exercise Program with and without Abdominal Strengthening Exercises on Diastasis Rectus Abdominis - A Post Partum Follow Up. Indian Journal of Physiotherapy & Occupational Therapy. 2013;7(4):123-6.
42. Barakat  R, Cordero  Y, Coteron  J, Luaces  M, Montejo  R.  Exercise during pregnancy improves maternal glucose screen at 24-28 weeks: a randomised controlled trial.   Br J Sports Med. 2012;46(9):656-661. doi:10.1136/bjsports-2011-090009PubMedGoogle ScholarCrossref
43. Barakat  R, Franco  E, Perales  M, López  C, Mottola  MF.  Exercise during pregnancy is associated with a shorter duration of labor: a randomized clinical trial.   Eur J Obstet Gynecol Reprod Biol. 2018;224:33-40. doi:10.1016/j.ejogrb.2018.03.009PubMedGoogle ScholarCrossref
44. Barakat  R, Perales  M, Bacchi  M, Coteron  J, Refoyo  I.  A program of exercise throughout pregnancy: is it safe to mother and newborn?   Am J Health Promot. 2014;29(1):2-8. doi:10.4278/ajhp.130131-QUAN-56PubMedGoogle ScholarCrossref
45. Barakat  R, Refoyo  I, Coteron  J, Franco  E.  Exercise during pregnancy has a preventative effect on excessive maternal weight gain and gestational diabetes: a randomized controlled trial.   Braz J Phys Ther. 2019;23(2):148-155. doi:10.1016/j.bjpt.2018.11.005PubMedGoogle ScholarCrossref
46. Barakat R, Alonso G, Rojo JJ. Physical exercise during pregnancy and its relationship with the duration of stages of labor. Prog Obstet Ginecol. 2005;48(2):61-8.
47. Barakat R, Lucia A, Ruiz J. Resistance exercise training during pregnancy and newborn’s birth size: a randomised controlled trial. Int J Obes V 33 20052009;1048–57.
48. Barakat R, Pelaez M, Cordero Y et al. Exercise during pregnancy protects against hypertension and macrosomia. Randomized clinical trial. Am J Obstet Gynecol 2016; 214: 649.e1–649.e8. doi:10.1016/j.ajog.2015.11. 039
49. Barakat R, Pelaez M, Lopez C, et al. Exercise during pregnancy and gestational diabetes-related adverse effects: a randomised controlled trial. Br J Sports Med 2013;47:630–6.
50. Barakat R, Pelaez M, Lopez C, Montejo R, Coteron J. Exercise during pregnancy reduces the rate of cesarean and instrumental deliveries: results of a randomized controlled trial. J Matern Fetal Neonatal Med 2012; 25(11): 2372-6.
51. Barakat R, Pelaez M, Montejo R, et al. Exercise during pregnancy improves maternal health perception: a randomized controlled trial. Am J Obstet Gynecol 2011; 204: 402e1-e7.
52. Barakat R, Pelaez M, Montejo R, et al. Exercise throughout pregnancy does not cause preterm delivery: a randomized, controlled trial. J Phys Act Health 2014;11:1012–7
53. Barakat R, Perales M, Bacchi M et al. A program of exercise throughout pregnancy. Is it safe to mother and newborn? Am J Health Promot 2014; 29(1): 2–8.
54. Barakat R, Ruiz J, Stirling J, Zakynthinaki M, Lucia A. Type of delivery is not affected by light resistance and toning exercise training during pregnancy: a randomized controlled trial. Am J Obstet Gynecol 2009; 201(6): 590.e591-596.
55. Barakat R, Ruiz JR, Lucia A. Exercise during pregnancy and risk of maternal anaemia: a randomised controlled trial. Br J Sports Med 2009; 43(12): 954-6.
56. Barakat R, Stirling JR, Lucia A. Does exercise training during pregnancy affect gestational age? A randomised controlled trial. Br J Sports Med 2008;42(8):674–8
57. Barakat R., Perales M., Cordero Y., Bacchi M., Mottola M.F. Influence of Land or Water Exercise in Pregnancy on Outcomes. Med. Sci. Sports Exerc. 2017;49:1397–1403. doi: 10.1249/MSS.0000000000001234
58. Batool A, Kousar Z, Tariq M, Saleem M, Anwer N, Aslam J. Comparison of aerobic and pilates exercises on depression and sleep quality in primigravida females. Pakistan J Med Health Sci. 2023;17(11):27
59. Battle CL, Uebelacker LA, Magee SR, Sutton KA, Miller IW. Potential for prenatal yoga to serve as an intervention to treat depression during pregnancy. Womens Health Issues. 2015;25(2):134-41.
60. Bech BH, Obel C, Henriksen TB, Olsen J. Effect of reducing caffeine intake on birth weight and length of gestation: randomised controlled trial. BMJ 2007;334:409–12.
61. Bechtel-Blackwell D. Computer-assisted self-interview and nutrition education in pregnant teens. Clin. Nurs. Res. 2002;11:450–462. doi: 10.1177/105477302237456. Obes. 2013;37:814–821. doi: 10.1038/ijo.2012.162.
62. Beckmann CR, Beckmann CA. Effect of a structured antepartum exercise program on pregnancy and labor outcome in primiparas. J Reprod Med. 1990;35(7):704-9.
63. Beddoe AE, & Lee KA (2010). Effects of mindful yoga on sleep in pregnant women: A pilot study— Mind-body interventions during pregnancy. Biological Research for Nursing, 11, 363–370. doi: 10.1177/1099800409356320 [PubMed: 20338897]
64. Beddoe AE, Paul Yang CP, Kennedy HP, et al. The effects of mindfulness-based yoga during pregnancy on maternal psychological and physical distress. J Obstet Gynecol Neonatal Nurs 2009;38:310–9.
65. Bell and S. M. Palma, “Antenatal exercise and birthweight,” The Australian and New Zealand Journal of Obstetrics and Gynaecology, vol. 40, no. 1, pp. 70–73, 2000.
66. Bell RJ, Palma SM, Lumley JM. The effect of vigorous exercise during pregnancy on birth-weight. The Australian and New Zealand Journal of Obstetrics and Gynaecology 1995;35:46–51.
67. Bershadsky S, Trumpfheller L, Kimble HB, et al. The effect of prenatal Hatha yoga on affect, cortisol and depressive symptoms. Complement Ther Clin Pract 2014;20:106–13.
68. Bertz F, Brekke HK, Ellega ̊rd L, Rasmussen KM, Wennergren M, Winkvist A. Diet and exercise weight-loss trial in lactating overweight and obese women. Am J Clin Nutr 2012;96(4): 698–705.
69. Bertz F, Winkvist A, Brekke HK. Sustainable weight loss among overweight and obese lactating women is achieved with an energy-reduced diet in line with dietary recommendations: results from the LEVA randomized controlled trial. J Acad Nutr Diet 2015; 115: 78–8
70. Bessinger RC, McMurray RG, Hackney AC. Substrate utilization and hormonal responses to moderate intensity exercise during pregnancy and after delivery. Am J Obstet Gynecol 2002;186:757–64.
71. Beyaz EA, Ozcan E, Ketenci A, Beyaz MM. The effectiveness of pregnancy rehabilitation: Effects on low back pain and calf cramps during pregnancy and pregnancy outcome. Nobel Medicus. 2011;7(2):67-74
72. Bhartia N, Jain S, Shankar N, Rajaram S, Gupta M. Effects of Antenatal Yoga on Maternal Stress and Clinical Outcomes in North Indian Women: A Randomised Controlled Trial. JIACM 2019;20:10–4.
73. Bisson M, Almeras N, Dufresne S, Rheaume C, Bujold E, Robitailee J, et al. Exercise improving fitness in obese women during pregnancy: a difference for the mother and child?. Pediatric Academic Societies and Asian Society for Pediatric Research Joint Meeting; 2014 May 3-6;Vancouver, Canada. 2014:Abstract no: 2946.618.
74. Bisson M, Alméras N, Dufresne SS, et al. A 12-Week Exercise Program for Pregnant Women with Obesity to Improve Physical Activity Levels: An Open Randomised Preliminary Study. PLoS One 2015;10:e0137742.
75. Bo K, Fleten C, Nystad W. Effect of antenatal pelvic floor muscle training on labor and birth. Obstet Gynecol. 2009;113(6):1279-84.
76. Bø K, Haakstad LA. Is pelvic floor muscle training effective when taught in a general fitness class in pregnancy? A randomised controlled trial. Physiotherapy 2011; 97(3): 190-5.
77. Bo K, Hilde G, Staer-Jensen J, Siafarikas F, Tennfjord MK, Engh ME. Does general exercise training before and during pregnancy influence the pelvic floor "opening" and delivery outcome? A 3D/4D ultrasound study following nulliparous pregnant women from mid-pregnancy to childbirth. Br J Sports Med. 2015;49:196-9.
78. Bo S, Rosato R, Ciccone G, Canil S, Gambino R, Poala CB, et al. Simple lifestyle recommendations and the outcomes of gestational diabetes. A 2 x 2 factorial randomized trial. Diabetes Obes Metab. 2014;16(10):1032-5.
79. Bogaerts A F, Devlieger R, Nuyts E, Witters I, Gyselaers W, Van den Bergh B RH. Effects of lifestyle intervention in obese pregnant women on gestational weight gain and mental health: a randomized controlled trial. Int J Obes. 2013;37(06):814–821. [PubMed] [Google Scholar]
80. Bogaerts A, Devlieger R, Nuyts E, Witters I, Gyselaers W, Guelinckx I. Psycho-education reduces gestational weight gain in obese pregnant women: Randomized controlled trial [abstract]. Obesity Facts 2012;5:53.
81. Bonen A, Campagna P, Gilchrist L, et al. Substrate and endocrine responses during exercise at selected stages of pregnancy. J Appl Physiol 1992;73:134–42.
82. Bonomo M., Corica D., Mion E., Goncalves D., Motta G., Merati R., Ragusa A., Morabito A. Evaluating the therapeutic approach in pregnancies complicated by borderline glucose intolerance: A randomized clinical trial. Diabet. Med. 2005;22:1536–1541. doi: 10.1111/j.1464-5491.2005.01690.x.
83. Bosaeus M, Hussain A, Karlsson T, Andersson L, Hulthen L, Svelander C, et al. A randomized longitudinal dietary intervention study during pregnancy: effects on fish intake, phospholipids, and body composition. Nutr J 2015;14:1.
84. Bosaeus M., Hussain A., Karlsson T., Andersson L., Hulthen L., Svelander C., Sandberg A.S., Larsson I., Ellegard L., Holmang A. A randomized longitudinal dietary intervention study during pregnancy: Effects on fish intake, phospholipids, and body composition. Nutrition. 2015;14:1. doi: 10.1186/1475-2891-14-1.
85. Bot M, Milder IE, Bemelmans WJ. Nationwide implementation of Hello World: a Dutch email-based health promotion program for pregnant women. J Med Internet Res 2009; 11: e24.
86. Botkin C, Driscoll CE. Maternal aerobic exercise: newborn effects. Fam Pract Res J. 1991;11(4):387-93.
87. Bowen A, Stewart N, Baetz M, et al. Antenatal depression in socially high-risk women in Canada. J Epidemiol Community Health 2009;63:414–6
88. Brand-Miller J. A pregnancy intervention to reduce postprandial glucose excursions in the primary prevention of paediatric obesity. Current Controlled Trials (www.controlled-trials.com/) [accessed 31 October 2010].
89. Brankston GN, Mitchell BF, Ryan EA, et al. Resistance exercise decreases the need for insulin in overweight women with gestational diabetes mellitus. Am J Obstet Gynecol 2004;190:188–93.
90. Brekke HK, Bertz F, Rasmussen KM, Bosaeus I, Ellegard L, Winkvist A. Diet and exercise interventions among overweight and obese lactating women: Randomized trial of effects on cardiovascular risk factors. PloS One 2014;9(2):e88250.
91. Brenner IK, Wolfe LA, Monga M, McGrath MJ. Physical conditioning effects on fetal heart rate responses to graded maternal exercise. Medicine & Science in Sports & Exercise. 1999;31(6):792-9
92. Brik M, Fernández-Buhigas I, Martin-Arias A, Vargas-Terrones M, Barakat R, Santacruz B. Does exercise during pregnancy impact on maternal weight gain and fetal cardiac function: a randomized controlled trial. Ultrasound Obstet Gynecol. 2019;53(5):583-589. doi:10.1002/uog.20147
93. Briley AL, Barr S, Badger S, Bell R, Croker H, Godfrey KM, et al. A complex intervention to improve pregnancy outcome in obese women; the UPBEAT randomised controlled trial. BMC Pregnancy and Childbirth 2014;14(1):74.
94. Briley C, Flanagan NL, Lewis N. In-home prenatal nutrition intervention increased dietary iron intakes and reduced low birthweight in low-income African-American women. J Am Diet Assoc 2002;102:984–7.
95. Broekhuizen K, Althuizen E, van Poppel MNM, Donker M, van Mechelen W. From theory to practice: intervention fidelity in a randomized controlled trial aiming to optimize weight development during pregnancy. Health Promotion Practice 2012;13(6):816–25.
96. Brownfoot, M.A. Davey, L. Kornman, Routine weighing to reduce excessive antenatal weight gain: a randomised controlled trial, BJOG Int. J. Obst. Gynaecol. 123 (2) (2016) 254–261.
97. Bruno R, Petrella E, Bertarini V, Pedrielli G, Neri I, Facchinetti F. Adherence to a lifestyle programme in overweight/obese pregnant women and effect on gestational diabetes mellitus: a randomized controlled trial. Matern Child Nutr 2016;13:e12333
98. Buckingham-Schutt LM, Ellingson LD, Vazou S, Campbell CG. The Behavioral Wellness in Pregnancy study: a randomized controlled trial of a multi-component intervention to promote appropriate weight gain. Am J Clin Nutr. 2019;109(4):1071-1079. doi:10.1093/ajcn/nqy35
99. Bulguroglu M, Bulguroglu HI. The effects of online pilates on the mood and fear of birth of pregnant women: a randomized controlled study. Sci Rep. 2024;14(1):16143
100. Bung P, Artal R, Khodiguian N, et al. Exercise in gestational diabetes. An optional therapeutic approach? Diabetes 1991;40 Suppl 2(Supplement_2):182–5
101. Buran G, Erim S. The effect of pregnancy pilates-assisted childbirth Preparation training on urinary incontinence and birth outcomes: a randomizedcontrolled study. Arch Gynecol Obstet. 2024;310(5):2725–35.
102. Byrne NM, Groves AM, McIntyre HD, Callaway LK. Changes in resting and walking energy expenditure and walking speed during pregnancy in obese women. American Journal of Clinical Nutrition 2011;94(3):819–30.
103. Cahill A., Haire-Joshu D., Cade W., Stein R., Woolfolk C., Moley K., Mathur A., Schechtman K., Klein S. Weight control program and gestational weight gain in disadvantaged women with overweight or obesity: A randomized clinical trial. Obesity. 2018;26:485–491. doi: 10.1002/oby.22070.
104. Callaway L, McIntyre D, Colditz P, Byrne N, Foxcroft K, O’Connor B. Exercise in obese pregnant women: a randomized study to assess feasibility. Hypertension in Pregnancy 2008;27(4):549.
105. Callaway L. A randomized controlled trial using exercise to reduce gestational diabetes and other adverse maternal and neonatal outcomes in obese pregnant women - the pilot study. Australian Clinical Trials Registry (www.actr.org.au/)[accessed 31 October 2010].
106. Callaway LK, Colditz PB, Byrne NM, et al. Prevention of gestational diabetes: feasibility issues for an exercise intervention in obese pregnant women. Diabetes Care 2010;33(7):1457–9.
107. Carlo C, Iannotti G, Sparice S, Chiacchio MP, Greco E, Tommaselli GA, et al. The role of a personalized dietary intervention in managing gestational weight gain: A prospective, controlled study in a low-risk antenatal population. Arch Gynecol Obstet 2014;289(4):765–70.
108. Carpenter MW, Sady SP, Sady MA, Haydon BB, Coustah DR, Thompson PD. Effects of exercise training in midpregnancy: a randomized controlled trial. Presented at the 37th annual meeting of the Society for Gynecologic Investigation, St Louis. Missouri, March 21-24,1990;Abstr.497, p. 345
109. Carpenter RE, Emery SJ, Uzun O, Rassi D, Lewis MJ. Influence of antenatal physical exercise on heart rate variability and QT variability. Journal of Maternal-Fetal & Neonatal Medicine. 2017;30(1):79.
110. Carpenter RE, Emery SJ, Uzun O, Silva LAD, Lewis MJ. Influence of antenatal physical exercise on haemodynamics in pregnant women: A flexible randomisation approach. BMC Pregnancy and Childbirth. 2015;15:156.
111. Carral F, Ayala Mdel C, Fernández JJ, González C, Piñero A, García G, et al. Web-based telemedicine system is useful for monitoring glucose control in pregnant women with diabetes. Diabetes Technol Ther 2015 May;17(5):349-354. [doi: 10.1089/dia.2014.0223] [Medline: 25629547]
112. Cavalcante S., Cecatti J., Pereira R., Baciuk E., Bernardo A., Silveira C. Water aerobics II: Maternal body composition and perinatal outcomes after a program for low risk pregnant women. Reprod. Health. 2009;6:1. doi: 10.1186/1742-4755-6-1.
113. Ceesay SM, Prentice AM, Cole TJ, Foord F, Weaver LT, Poskitt EM, Whitehead RG. Effects on birth weight and perinatal mortality of maternal dietary supplements in rural Gambia: 5 year randomised controlled trial. BMJ 1997;315:786–90.
114. Chan G.M., McElligott K., McNaught T. & Gill G. (2006) Effects of dietary calcium intervention on adolescent mothers and newborns: a randomized controlled trial. Obstetrics and Gynecology 108, 565–571.
115. Chan RS-M, Tam W-H, Ho IC-H, Kwan MW-C, Li LS, Sea MM-M, et al. Randomized trial examining effectiveness of lifestyle intervention in reducing gestational diabetes in high risk Chinese pregnant women in Hong Kong. Sci Rep 2018;8(December 1):13849.
116. Chao, A.M., Srinivas, S. K., Studt, S. K., Diewald, L. K., Sarwer, D. B., & Allison, K. C. (2017). A pilot randomized controlled trial of a technology-based approach for preventing excess weight gain during pregnancy among women with overweight. Frontiers in Nutrition, 4, 57.
117. Chasan-Taber L, Schmidt MD, Pekow P, et al. Physical Activity and Gestational Diabetes Mellitus among Hispanic Women. J Womens Health 2008;17:999–1008.
118. Chasan-Taber L, Silveira M, Marcus BH, Braun B, Stanek E, et al. (2011) Feasibility and efficacy of a physical activity intervention among pregnant women: The behaviors affecting baby and you (BABY) study. Journal of physical activity & health 8: S228.
119. Chasan-Taber L, Silveira M, Pekow P, et al. Physical activity, sedentary behavior and risk of hypertensive disorders of pregnancy in Hispanic women. Hypertens Pregnancy 2015;34:1–16.
120. Chávez VG, Sánchez, M. D. P V, Rasch JRK. Effect of pelvic floor exercise during pregnancy and puerperium on prevention of urinary stress incontinence. Ginecol Obstet Mex. 2004;72(628-636).
121. Chiarello CM, Falzone LA, McCaslin KE, Patel MN, Ulery KR. The effects of an exercise program on diastasis recti abdominis in pregnant women [corrected] [published erratum appears in J WOMENS HEALTH PHYS THER 2005;29(3):76]. 2005.
122. Choi, J., Lee, J. H., Vittinghoff, E., & Fukuoka, Y. (2016). MHealth physical activity intervention: a randomized pilot study in physically inactive pregnant women. Maternal Child and Health Journal, 20, 1091–1101.
123. Chuntharapat S, Petpichetchian W, Hatthakit U. Yoga during pregnancy: effects on maternal comfort, labor pain and birth outcomes. Complement Ther Clin Pract. 2008;14(2):105-15.
124. Claesson IM, Klein S, Sydsjö G, et al. Physical activity and psychological well-being in obese pregnant and postpartum women attending a weight-gain restriction programme. Midwifery 2014;30:11–16.
125. Claesson IM, Sydsjö G, Brynhildsen J, Cedergren M, Jeppsson A, Nyström F, et al. Weight gain restriction for obese pregnant women: a case–control intervention study. BJOG. 2008;115(1):44–50.
126. Claesson, S. Klein, G. Sydsjo, A. Josefsson, Physical activity and psychological well-being in obese pregnant and postpartum women attending a weight-gain restriction programme, Midwifery 30 (1) (2014) 11–16.
127. Clapp III JF, Kim H, Burciu B et al. Beginning regular exercise in early pregnancy: effect on fetoplacental growth. Am J Obstet Gynecol 2000; 183(6):1484–1488.
128. Clapp III JF, Kim H, Burciu B et al. Continuing regular exercise during pregnancy: effect of exercise volume on fetoplacental growth. Am J Obstet Gynecol 2002; 186(1):142–147.
129. Clapp III JF. Maternal carbohydrate intake and pregnancy outcome. Proceedings of the Nutrition Society 2002; 61(1):45–50.
130. Clapp IJF. Diet, exercise, and feto-placental growth. Archives of Gynecology and Obstetrics 1997;260:101–8.
131. Clapp JF, 3rd. The course of labor after endurance exercise during pregnancy. Am J Obstet Gynecol. 1990;163(6 Pt 1):1799-805.
132. Clapp JF, Capeless EL. The changing glycemic response to exercise during pregnancy. Am J Obstet Gynecol 1991;165:1678–83.
133. Clapp JF, Wesley M, Sleamaker RH. Thermoregulatory and metabolic responses to jogging prior to and during pregnancy. Med Sci Sports Exerc 1987;19:124???130–30.
134. Clapp JF. Effect of dietary carbohydrate on the glucose and insulin response to mixed caloric intake and exercise in both nonpregnant and pregnant women. Diabetes Care 1998;21 Suppl 2(Suppl 2):107.
135. Clapp JF. Influence of endurance exercise and diet on human placental development and fetal growth. Placenta 2006;27(6-7):527–34
136. Clark E, Isler C, Strickland D, et al. Influence of aerobic exercise on maternal lipid levels and offspring morphometrics. Int J Obes (Lond). 2019;43(3):594-602. doi:10.1038/s41366-018-0258-z
137. ClinicalTrials.gov The MOTHER (Mobile Technologies to Help Enhancing Regular Physical Activity) trial for pregnant women. National Library of Medicine: Bethesda, MD, USA. 2011. Identifier NCT01461707. Available from http://www. clinicaltrials.gov/ct2/show/record/NCT01461707
138. ClinicalTrials.gov. Electronically-mediated weight interventions for pregnant and postpartum women. National Library of Medicine: Bethesda, MD, USA. 2013. Identifier NCT01331564. Available from http://clinicaltrials.gov/ct2/show/record/ NCT01331564.
139. ClinicalTrials.gov. Personalised management of body weight during pregnancy. National Library of Medicine: Bethesda, MD, USA. 2012. Identifier NCT01610752. Available from http://www.clinicaltrials.gov/ct2/show/NCT01610752.
140. Coll, C.V.N., Domingues, M.R., Stein, A., da Silva, B.G.C., Bassani, D.G., Hartwig, F.P., da Silva, I.C.M., da Silveira, M.F., da Silva, S.G., Bertoldi, A.D., 2019. Efficacy of regular exercise during pregnancy on the prevention of postpartum depression: the PAMELA randomized clinical trial. JAMA Netw. Open 2, e186861. Services, U.D.o.H.a.H., 2018. Physical Activity Guidelines for Americans, 2nd edition. US Department of Health and Human Services, Washington
141. Colleran HL, Lovelady CA. Use of mypyramid menu planner for moms in a weight-loss intervention during lactation. J Acad Nutr Diet 2012; 112: 553–558
142. Colleran HL, Wideman L, Lovelady CA. Effects of energy restriction and exercise on bone mineral density during lactation. Med Sci Sport Ex 2012;44(8):1570–9
143. Collings CA, Curet L, Mullin J. Maternal and fetal responses to a maternal aerobic exercise program. Am J Obstet Gynecol 1983; 145(6):702–707.
144. Collings CA, Curet LB, Mullin JP. Maternal and fetal responses to a maternal aerobic exercise program. Am J Obstet Gynecol 1983;145:702–7.
145. Cordero FY, Mottola FM, Vargas FJ, Blanco FM, Barakat FR. Exercise is associated with a reduction in gestational diabetes mellitus. Med Sci Sports Exerc. 2015;47(7):1328–33. https://doi.org/10.1249/MSS.0000000000000547.
146. Cordero Rodriguez Y, Peláez Puente M, Miguel Abad Md, Perales Santaella M, Barakat Carballo R. Puede el ejercicio físico moderado durante el embarazo actuar como un factor de prevención de la Diabetes Gestacional? /Can moderate physical exercise during pregnancy act as a factor in preventing Gestational Diabetes? RICYDE Rev int cienc deporte. 2012;27(8):3-19.
147. Cordero Y, Peláez M, De Miguel M, et al. Can moderate physical exercise during pregnancy act as a factor in preventing Gestational Diabetes? Revista Internacional de Ciencias del Deporte 2012;27:3–19.
148. Courville AB, Harel O, Lammi-Keefe C. Consumption of a DHA containing functional food during pregnancy is associated with lower infant ponderal index and cord plasma insulin concentration. Br J Nutr 2011;106:208–12. 8. Luoto R, Kinnunen T, Aittasalo M, Kolu P, Raitanen J, Ojala K, Mansikkama ̈ki K, Lamberg S, Vasankari T, Komulainen T, et al. Primary prevention of gestational diabetes mellitus and large-for-gestational age newborns by lifestyle counseling: a cluster-randomized controlled trial. PLoS Med 2011;8:e1001036.
149. Cowett RM, Carpenter MW, Carr S, et al. Glucose and lactate kinetics during a short exercise bout in pregnancy. Metabolism 1996;45:753–8.
150. Craigie AM, Macleod M, Barton KL, Treweek S, Anderson AS. Supporting postpartum weight loss in women living in deprived communities: design implications for a randomised control trial. Eur J Clin Nutr 2011;65(8):952– 8.
151. Cramp CS, Moran LJ, Deussen AR, Yelland LN, Dodd JM. Evaluation of printed nutrition education material in overweight and obese women during pregnancy-findings from the limit randomised trial. Journal of Paediatrics and Child Health 2013;49 Suppl 2:118.
152. Crowther C, Hiller J, Moss J, McPhee A, Jeffries W,Robinson J. Effect of treatment of gestational diabetesmellitus on pregnancy outcomes. N Engl J Med.2005;352:2477–86
153. Currie LM, Woolcott CG, Fell DB, et al. The association between physical activity and maternal and neonatal outcomes: a prospective cohort. Matern Child Health J 2014;18:1823–30.
154. Currie S, Sinclair M, Liddle DS, Nevill A, Murphy MH. Application of objective physical activity measurement in an antenatal physical activity consultation intervention: a randomised controlled trial. BMC Public Health [Internet] 2015. . Dec 18 [cited 2019 Jun 18];15. Available from: https://www.ncbi.nlm. nih.gov/pmc/articles/PMC4684930/.
155. Cw K, Napolitano PG, Lee SP, et al. Physical activity, maternal metabolic measures, and the incidence of gallbladder sludge or stones during pregnancy: a randomized trial. Am J Perinatol 2014;31:39–48.
156. D’Almeida A, Carter JP, Anatol A, Prost C. Effects of acombination of evening primrose oil (gamma linolenicacid) and fish oil (eicosapentaenoic+docahexaenoic acid)versus magnesium, and versus placebo in preventingpreeclampsia. Women Health. 1992;19:117–31
157. Da Costa D, Rippen N, Dritsa M, et al. Self-reported leisure-time physical activity during pregnancy and relationship to psychological well-being. J Psychosom Obstet Gynaecol 2003;24:111–9.
158. Da Silva, S.G.; Hallal, P.C.; Domingues, M.R.; Bertoldi, A.D.; Silveira, M.F.D.; Bassani, D.; da Silva, I.C.M.; da Silva, B.G.C.; Coll, C.V.N.; Evenson, K. A randomized controlled trial of exercise during pregnancy on maternal and neonatal outcomes: Results from the PAMELA study. Int. J. Behav. Nutr. Phys. Act. 2017, 14, 175.
159. Dahl A. Healthy Motivations for Moms-To-Be (Healthy MoM2B) Study: A Mobile Health Intervention Targeting Gestational Weight Gain among US Women. Semantic Scholar. 2018. URL: https://www.semanticscholar.org/paper/Healthy-Motivations-for-Moms-To-Be-(Healthy-MoM2B)Dahl/828d334dde24173f90099c03457c0674e0050740 [accessed
160. Dale E, Mullinax KM, Bryan DH. Exercise during pregnancy: effects on the fetus. Canadian Journal of Applied Sport Sciences. 1982;7(2):98-103
161. Daley  A, Jolly  K, Jebb  SA,  et al.  Effectiveness of a behavioural intervention involving regular weighing and feedback by community midwives within routine antenatal care to prevent excessive gestational weight gain: POPS2 randomised controlled trial.   BMJ Open. 2019;9(9):e030174. doi:10.1136/bmjopen-2019-030174PubMedGoogle Scholar
162. Daley  AJ, Jolly  K, Jebb  SA,  et al.  Feasibility and acceptability of regular weighing, setting weight gain limits and providing feedback by community midwives to prevent excess weight gain during pregnancy: randomised controlled trial and qualitative study.   BMC Obes. 2015;2(1):35. doi:10.1186/s40608-015-0061-5PubMedGoogle ScholarCrossref
163. Daley, A., Riaz, M., Lewis, S., Aveyard, P., Coleman, T., Manyonda, I., West, R., Lewis, B., Marcus, B., Taylor, A., Ibison, J., Kent, A., Ussher, M., 2018. Physical activity for antenatal and postnatal depression in women attempting to quit smoking: randomised controlled trial. BMC Pregnancy Childbirth 18, 156.
164. Daley, A.J., Blamey, R.V., Jolly, K., Roalfe, A.K., Turner, K.M., Coleman, S., McGuinness, M., Jones, I., Sharp, D.J., MacArthur, C., 2015a. A pragmatic randomized controlled trial to evaluate the effectiveness of a facilitated exercise intervention as a treatment for postnatal depression: the PAM-PeRS trial. Psychol. Med. 45, 2413–2425.
165. Daley, A.J., Foster, L., Long, G., Palmer, C., Robinson, O., Walmsley, H., Ward, R., 2015b. The effectiveness of exercise for the prevention and treatment of antenatal depression: systematic review with meta-analysis. BJOG 122, 57–62.
166. Daly  N, Farren  M, McKeating  A, OʼKelly  R, Stapleton  M, Turner  MJ.  A medically supervised pregnancy exercise intervention in obese women: a randomized controlled trial.   Obstet Gynecol. 2017;130(5):1001-1010. doi:10.1097/AOG.0000000000002267PubMedGoogle ScholarCrossref
167. Daly N, Farren M, McKeating A, O'Higgins A, Mullaney L, Turner MJ. Effect of a randomized controlled trial of an intensive medically supervised exercise program designed to improve maternal glucose control on gestational weight gain. Am J Obstet Gynecol. 2017;216(1 Supplement 1):S24.
168. Daniel J, Dikki C. Aerobic dance exercise improves blood glucose level in pregnant women with gestational diabetes mellitus: sport science. African Journal for Physical Health Education, Recreation and Dance. 2014:273–81.
169. D'Anna R, Scilipoti A, Giordano D, Caruso C, Cannata ML, et al. (2013) Myo-inositol supplementation and onset of gestational diabetes mellitus in pregnant women with a family history of type 2 diabetes: A prospective, randomized, placebo-controlled study. Diabetes Care 36(4):854–7. doi: 10.2337/dc12-1371 PMID: 23340885
170. Davenport MH, Girouox I, Sopper MM, Mottola MF. Postpartum Exercise Regardless of Intensity Improves Chronic Disease Risk Factors. Med Sci Sports Exerc 2011;43(6):951–8.
171. Davenport MH, Mottola MF, McManus R, et al. A walking intervention improves capillary glucose control in women with gestational diabetes mellitus: a pilot study. Appl Physiol Nutr Metab 2008;33:511–7.
172. Davis, K., Goodman, S.H., Leiferman, J., Taylor, M., Dimidjian, S., 2015. A randomized controlled trial of yoga for pregnant women with symptoms of depression and anxiety. Complement. Ther. Clin. Pract. 21, 166–172.
173. de Barros MC, Lopes MA, Francisco RP, et al. Resistance exercise and glycemic control in women with gestational diabetes mellitus. Am J Obstet Gynecol 2010;203:556. e1–556.e6.
174. de Groot RHM, Hornstra G, van Houwelingen AC, Roumen F. Effect of alpha-linolenic acid supplementation during pregnancy on maternal and neonatal polyunsaturated fatty acid status and pregnancy outcome. Am J Clin Nutr 2004;79:251–60.
175. de Oliveria Melo AS, Silva JL, Tavares JS, Barros VO, Leite DF, Amorim MM. Effect of a physical exercise program during pregnancy on uteroplacental and fetal blood flow and fetal growth: a randomized controlled trial. Obstet Gynecol 2012; 120: 302-10.
176. de Wit L, Jelsma JG, van Poppel MN, et al. Physical activity, depressed mood and pregnancy worries in European obese pregnant women: results from the DALI study. BMC Pregnancy Childbirth 2015;15:158.
177. Dekker Nitert D, Barrett HL, Denny KJ, McIntyre HD, Callaway LK. Exercise in pregnancy does not alter gestational weight gain, MCP-1 or leptin in obese women. Aust N Z JObstet Gynaecol 2015;55(1): 27– 33.https://doi.org/10.1111/ajo.12300.
178. Dempsey JC, Butler CL, Sorensen TK, et al. A case-control study of maternal recreational physical activity and risk of gestational diabetes mellitus. Diabetes Res Clin Pract 2004;66:203–15.
179. Denda OS, Dalkiranis AX, Denda MS, et al. The effect of some dietary components on body weight and fat deposition in pregnant women in combination with exercise during pregnancy and postpartum period. Studies in Physical Culture and Tourism 2005;12.
180. Deng Y, Hou Y, Wu L, Liu Y, Ma L, Yao A. Effects of Diet and Exercise Interventions to Prevent Gestational Diabetes Mellitus in Pregnant Women With High-Risk Factors in China: A Randomized Controlled Study. Clin Nurs Res 2022;31:836–47.
181. Deveer R., Deveer M., Akbaba E., Engin-Üstün Y., Aydoǧan P., Çelikkaya H., Danisman N., Mollamahmutoglu L. The effect of diet on pregnancy outcomes among pregnants with abnormal glucose challenge test. Eur. Rev. Med. Pharmacol. Sci. 2013;17:1258–1261.
182. Di Carlo C, Iannotti G, Sparice S et al..The role of a personalized dietary intervention in managing gestational weight gain: a prospective, controlled study in a low-risk antenatal population. Arch Gynecol Obstet. 2014;289(04):765–770. [PubMed] [Google Scholar]
183. Di Carlo C., Iannotti G., Sparice S., Chiacchio M., Greco E., Tommaselli G., Nappi C. The role of a personalized dietary intervention in managing gestational weight gain: A prospective, controlled study in a low-risk antenatal population. Arch. Gynecol. Obstet. 2014;289:765–770. doi: 10.1007/s00404-013-3054-y.
184. Dias LA, Driusso P, Aita DL, Quintana SM, Bo K, Ferreira CH. Effect of pelvic floor muscle training on labour and newborn outcomes: a randomized controlled trial. Rev Bras Fisioter. 2011;15(6):487-93
185. Dias NT, Ferreira LR, Fernandes MG, Resende APM, Pereira-Baldon VS. A pilates exercise program with pelvic floor muscle contraction: is it effective for pregnant women? A randomized controlled trial. Neurourol Urodyn. 2018;37(1):379–84.
186. Dodd  JM, Deussen  AR, Louise  J.  A randomised trial to optimise gestational weight gain and improve maternal and infant health outcomes through antenatal dietary, lifestyle and exercise advice: the OPTIMISE randomised trial.   Nutrients. 2019;11(12):E2911. doi:10.3390/nu11122911PubMedGoogle Scholar
187. Dodd  JM, Turnbull  D, McPhee  AJ,  et al; LIMIT Randomised Trial Group.  Antenatal lifestyle advice for women who are overweight or obese: LIMIT randomised trial.   BMJ. 2014;348:g1285. doi:10.1136/bmj.g1285PubMedGoogle ScholarCrossref
188. Dodd J. Limiting weight gain in overweight and obese women during pregnancy to improve health outcomes: a randomised trial. Australian Clinical Trials Registry (www.actr.org.au/) (accessed 31 October 2010).
189. Dodd J. Obesity in pregnancy-the limit randomised trial. Journal of Paediatrics and Child Health 2013;49 Suppl 2:4.
190. Dodd JM, Cramp C, Sui Z, Yelland LN, Deussen AR, Grivell RM, et al. The effects of antenatal dietary and lifestyle advice for women who are overweight or obese on maternal diet and physical activity: the LIMIT randomised trial. BMC Med [Internet] 2014. . Oct 13 [cited 2019 Jun 18];12. Available from: https:// www.ncbi.nlm.nih.gov/pmc/articles/PMC4194375/.
191. Dodd JM, Kannieappan LM, Grivell RM, Deussen AR, Moran LJ, Yelland LN, et al. Effects of an antenatal dietary intervention on maternal anthropometric measures in pregnant women with obesity. Obesity 2015;23(8):1555–62.
192. Dodd JM, Louise J, Cramp C, Grivell RM, Moran LJ, Deussen AR. Evaluation of a smartphone nutrition and physical activity application to provide lifestyle advice to pregnant women: The SNAPP randomised trial. Matern Child Nutr 2018 Jan;14(1). [doi: 10.1111/mcn.12502] [Medline: 28836373]
193. Dodd JM, McPhee AJ, Turnbull D, Yelland LN, Deussen  AR, Grivell RM, et al. The effects of antenatal dietary and lifestyle advice for women who are overweight or obese on neonatal health outcomes: the LIMIT randomised trial. BMC Medicine 2014;12(1):163.
194. Dodd JM, Newman A, Moran LJ, et al. The effect of antenatal dietary and lifestyle advice for women who are overweight or obese on emotional well-being: the LIMIT randomized trial. Acta Obstet Gynecol Scand 2016;95:309–18.
195. Dodd, J. Louise, A.R. Deussen, R.M. Grivell, G. Dekker, A.J. McPhee, W. Hague, Effect of metformin in addition to dietary and lifestyle advice for pregnant women who are overweight or obese: the GRoW randomised, double-blind, placebo-controlled trial, Lancet Diabetes Endocrinol. 7 (1)(2019) 15–24.
196. Donnelly J, Horan M, Walsh J, McGowan C, Byrne J, Molloy EJ, et al. Impact of a low GI diet on neonatal body composition (ROLO Kids). Pediatric Academic Societies Annual Meeting; 2013 May 4-7; Washington DC, USA. 2013
197. Donnelly JM, Lindsay KL, Walsh JM, Horan M, Molloy EJ, McAuliffe FM. Fetal metabolic influences of neonatal anthropometry and adiposity. BMC Pediatrics 2015;15(1): 175
198. Donnelly JM, Walsh JM, Byrne J, Molloy E, McAuliffe FM. Altered neonatal anthropometric measurements following maternal low GI diet in pregnancy (ROLO study). Acta Obstetricia et Gynecologica Scandinavica 2013;92(s160):13
199. Donnelly JM, Walsh JM, Byrne J, Molloy EJ, McAuliffe FM. Impact of maternal diet on neonatal anthropometry: A randomized controlled trial. Pediatric Obesity 2015;10(1): 52–6
200. Donnelly JMT, Lindsay KL, Walsh JM, Horan M, Molloy EJ, McAuliffe F. Impact of maternal and fetal inflammatory markers on neonatal and infant adiposity. Archives of Disease in Childhood 2015;100:A133–4
201. Doran F, & Hornibrook J (2013). Women’s experiences of participation in a pregnancy and postnatal group incorporating yoga and facilitated group discussion: A qualitative evaluation. Women and Birth, 26, 82–86. doi:10.1016/j.wombi.2012.06.001 [PubMed: 22818031]
202. Downs DS, DiNallo JM, Kirner TL. Determinants of pregnancy and postpartum depression: prospective influences of depressive symptoms, body image satisfaction, and exercise behavior. Ann Behav Med 2008;36:54–63.
203. Downs DS, Hausenblas HA. Pregnant women's third trimester exercise behaviors, body mass index, and pregnancy outcomes. PsycholHealth. 2007;22(5):545-59.
204. Dubois S, Dougherty C, Duquette MP, Hanley JA, Moutquin JM. Twin pregnancy: the impact of the Higgins Nutrition Intervention Program on maternal and neonatal outcomes. Am J Clin Nutr. 1991 Jun;53(6): 1397–1403. PMID: 2035467. doi: 10.1093/ajcn/53.6.1397.
205. Dumas GA, Reid JG, Wolfe LA, et al. Exercise, posture, and back pain during pregnancy. Clin Biomech 1995;10:98–103.
206. Dumith SC, Domingues MR, Mendoza-Sassi RA, et al. Physical activity during pregnancy and its association with maternal and child health indicators. Rev Saude Publica 2012;46:327–33.
207. Dyck RF, Sheppard SM, Klomp H, et al. Using exercise to prevent gestational diabetes among aboriginal women - hypothesis and results of a pilot/feasibility project in Saskatchewan. Canadian Journal of Diabetes Care 1999;23:32–8.
208. Eames AJ, Grivell RM, Dodd JM, Deussen A. The effect of limited gestational weight gain in overweight and obese women on maternal and infant outcomes. Journal of Paediatrics and Child Health 2013;49 Suppl 2:19.
209. Eggen MH, Stuge B, Mowinckel P, Jensen KS, Hagen KB. Can supervised group exercises including ergonomic advice reduce the prevalence and severity of low back pain and pelvic girdle pain in pregnancy? A randomized controlled trial. Phys Ther 2012;92(6):781–90.
210. Ehrlich SF, Hedderson MM, Feng J, Crites Y, Quesenberry CP, Ferrara A. Lifestyle intervention improves postpartum fasting glucose levels in women with gestational diabetes. Diabetes 2014; 63 (Suppl 1):A95.
211. Elden H, Ostgaard H-C, Fagevik-Olsen M, et al. Treatments of pelvic girdle pain in pregnant women: adverse effects of standard treatment, acupuncture and stabilising exercises on the pregnancy, mother, delivery and the fetus/neonate. BMC Complement Altern Med 2008;8:34.
212. El-Rafie, M.M., Khafagy, G.M., Gamal, M.G., 2016. Effect of aerobic exercise during pregnancy on antenatal depression. Int. J. Women's Health 8, 53–57.
213. Erkkola R, Mäkelä M. Heart volume and physical fitness of parturients. Ann Clin Res. 1976;8(1):15–21.
214. Erkkola, R The influence of physical training during pregnancy on physical work capacity and circulatory parameters, Scandinavian Journal of Clinical & Laboratory Investigation, vol. 36, no. 8, pp. 747–754, 1976.
215. Ersek JL, Brunner Huber LR. Physical activity prior to and during pregnancy and risk of postpartum depressive symptoms. J Obstet Gynecol Neonatal Nurs 2009;38:556–66.
216. Eslami E, Charandabi SMA, Khalili AF et al. The Effect of a Lifestyle-Based Training Package on Weight Gain and Frequency of Gestational Diabetes in Obese and Overweight Pregnant Females. Iran Red Crescent Med J 2018; 20 (Suppl 1): e62576. doi:10.5812/ircmj.62576
217. Evans W, Nielsen P, Szekely D, Bihm J, Murray E, Snider J, et al. Dose-response effects of the text4baby mobile health program: randomized controlled trial. JMIR Mhealth Uhealth 2015 Jan 28;3(1):e12
218. Evans WD, Wallace JL, Snider J. Pilot evaluation of the text4baby mobile health program. BMC Public Health 2012 Nov 26;12:103132.
219. Facchinetti F, Vijai V, Petrella E, et al. Food glycemic index changes in overweight/obese pregnant women enrolled in a lifestyle program: a randomized controlled trial. Am J Obstet Gynecol MFM 2019;13:100030. https://doi.org/10.1016/j.ajogmf.2019.100030.
220. Facchinetti F. Pregnancy complications in women with BMI > 25kg/m2 enrolled in a healthy lifestule and eating habits program. ClinicalTrials.gov (accessed 21 May 2013). NCT01783210 2013.
221. Fard NM, Mehrabian F, Sarraf-Zadegan N, Sajadi F. Fat-modified diets during pregnancy and lactation and serum lipids after birth. Indian J Pediatr 2004;71:683–7.
222. Fassett MJ, Dhillon SHK, Williams TR. Effects on perinatal outcome of treating women with 1 elevated glucose tolerance test value. Am J Obstet Gynecol. 2007;196(6):597.e1–597.e4; discussion.e4. PMID: 17547912. doi: 10.1016/j.ajog.2007.03.017.
223. Feodor Nilsson S, Andersen PK, Strandberg-Larsen K, et al. Risk factors for miscarriage from a prevention perspective: a nationwide follow-up study. BJOG 2014;121:1375–85
224. Feria-Ramírez C, Gonzalez-Sanz JD, Molina-Luque R, Molina-Recio G. The effects of the pilates method on pelvic floor injuries during pregnancy and childbirth: A quasi-experimental study. Int J Environ Res Public Health. 2021;18(13):6995.
225. Ferland S, Bujold E, Giguère Y, et al. Association between physical activity in early pregnancy and markers of placental growth and function. J Obstet Gynaecol Can 2013;35:787–92.
226. Ferrara A, Hedderson MM, Albright CL, Ehrlich SF, Quesenberry CP, Peng T, et al. A pregnancy and postpartum lifestyle intervention in women with gestational diabetes mellitus reduces diabetes risk factors: a feasibility randomized control trial. Diabetes Care 2011;34(7):1519–25.
227. Ferrara A, Hedderson MM, Brown SD, Albright CL, Ehrlich SF, Tsai AL, et al. The Comparative Effectiveness of Diabetes Prevention Strategies to Reduce Postpartum Weight Retention in Women With Gestational Diabetes Mellitus: The Gestational Diabetes' Effects on Moms (GEM) Cluster Randomized Controlled Trial. Diabetes Care. 2016;39(1):65-74.
228. Ferrara A. Diet, exercise and breastfeeding intervention program for women with gestational diabetes (DEBI Trial). ClinicalTrials.gov(www.clinicaltrials.gov) [accessed 31 October 2010].
229. Ferrara A, Hedderson MM, Brown SD, et al. A telehealth lifestyle intervention to reduce excess gestational weight gain in pregnant women with overweight or obesity (GLOW): a randomised, parallel-group, controlled trial. Lancet Diabetes Endocrinol. 2020;8(6):490-500. doi:10.1016/S2213-8587(20)30107-8
230. Field T, Diego M, Delgado J, & Medina L (2013a). Yoga and social support reduce prenatal depression, anxiety, and cortisol. Journal of Bodywork and Movement Therapies, 17, 397–403. doi: 10.1016/j.jbmt.2013.03.010 [PubMed: 24138994]
231. Field T, Diego M, Delgado J, & Medina L (2013b). Tai chi/yoga reduces prenatal depression, anxiety, and sleep disturbances. Complementary Therapies in Clinical Practice, 19(1), 6–10. doi:10.1016/ j.ctcp.2012.10.001 [PubMed: 23337557]
232. Field T, Diego M, Hernandez-Reif M, Medina L, Delgado J, & Hernandez A (2012). Yoga and massage therapy reduce prenatal depression and prematurity. Journal of Bodywork and Movement Therapies, 16, 204–209. doi:10.1016/j.jbmt.2011.08.002 [PubMed: 22464118]
233. Field T. Postpartum depression effects on early interactions, parenting, and safety practices: a review. Infant Behav Dev 2010;33:1–6
234. Figueira H, Vale R, Rodrigues W, Figueira A, Figueira J, Dantas E. Pregnancyrelated low back pain relief after maximum static flexibility program. Health. 2014;6(21):2966–2972. doi:10.4236/health.2014.621335
235. Filipec M, Matijevic R. Expert advice about therapeutic exercise during pregnancy reduces the symptoms of sacroiliac dysfunction. J Perinat Med. 2020;48(6):559–565. doi:10.1515/jpm-2020-0143.
236. Fjeldsoe BS, Miller YD, Marshall AL. MobileMums: a randomized controlled trial of an SMS-based physical activity intervention. Ann Behav Med 2010; 39: 101–111.
237. Forsyth, J., Boath, E., Henshaw, C., Brown, H., 2017. Exercise as an adjunct treatment for postpartum depression for women living in an inner city-a pilot study. Health Care Women Int. 38, 635–639.
238. Foxcroft KF, Rowlands IJ, Byrne NM, McIntyre HD, Callaway LK, for the BAMBINO group. Exercise in obese pregnant women: the role of social factors, lifestyle and pregnancy symptoms. BMC Pregnancy and Childbirth 2011;11:4.
239. Fraser RB, Ford FA, Milner RDG. A controlled trial of a high dietary fibre intake in pregnancy - effects on plasma glucose and insulin levels. Diabetologia 1983;25:238–41
240. Fraser RB. High fibre diets in pregnancy. Nutrition in Pregnancy. Proceedings of 10th Study Group of the Royal College of Obstetricians and Gynaecologists; 1982 September. London: RCOG, 1983:269–80
241. Fritel X, de Tayrac R, Bader G, Savary D, Gueye A, Deffieux X, et al. Preventing Urinary Incontinence With Supervised Prenatal Pelvic Floor Exercises: A Randomized Controlled Trial. Obstet Gynecol. 2015;126(2):370-7.
242. Gallagher  D, Rosenn  B, Toro-Ramos  T,  et al.  Greater neonatal fat-free mass and similar fat mass following a randomized trial to control excess gestational weight gain.   Obesity (Silver Spring). 2018;26(3):578-587. doi:10.1002/oby.22079PubMedGoogle ScholarCrossref
243. Gallagher, A., Kring, D., Whitley, T., 2020. Effects of yoga on anxiety and depression for high risk mothers on hospital bedrest. Complement. Ther. Clin. Pract. 38, 101079.
244. Gandhi M, Hosseinzadeh P, Salmanian B, Moaddab A, Golabbakhsh H, Clark SL, et al. Dietary consultation impact on weight gain in twin pregnancies. J Reprod Med. 2018;63(7–8):347–51.
245. Garnæs  KK, Mørkved  S, Salvesen  KA, Salvesen  Ø, Moholdt  T.  Exercise training during pregnancy reduces circulating insulin levels in overweight/obese women postpartum: secondary analysis of a randomised controlled trial (the ETIP trial).   BMC Pregnancy Childbirth. 2018;18(1):18. doi:10.1186/s12884-017-1653-5PubMedGoogle ScholarCrossref
246. Garnæs  KK, Mørkved  S, Salvesen  Ø, Moholdt  T.  Exercise training and weight gain in obese pregnant women: a randomized controlled trial (ETIP trial).   PLoS Med. 2016;13(7):e1002079. doi:10.1371/journal.pmed.1002079PubMedGoogle Scholar
247. Garshasbi, A.; Faghih Zadeh, S. The effect of exercise on the intensity of low back pain in pregnant women. Int. J. Gynaecol. Obstet. 2005, 88, 271–275.
248. Gaston A, Prapavessis H, Tired PH. Tired, moody and pregnant? Exercise may be the answer. Psychol Health 2013;28:1353–69
249. Gaston A, Prapavessis H. Maternal-fetal disease information as a source of exercise motivation during pregnancy. Health Psychol Off J Div Health Psychol Am Psychol Assoc. 2009;28(November 6):726–33.
250. Gau, M.L.; Chang, C.Y.; Tian, S.H.; Lin, K.C. Effects of birth ball exercise on pain and self-efficacy during childbirth: A randomised controlled trial in Taiwan. Midwifery 2011, 27, e293–e300
251. Geiker NRW, Magkos F, Zingenberg H, et al. A high-protein lowglycemic index diet attenuates gestational weight gain in pregnant women with obesity: the "An optimized programming of healthy children" (APPROACH) randomized controlled trial. Am J Clin Nutr 2022;1153:970e9. https://doi.org/10.1093/ajcn/nqab405.
252. Gesell S., Katula J., Strickland C., Vitolins M. Feasibility and initial efficacy evaluation of a community-based cognitive-behavioral lifestyle intervention to prevent excessive weight gain during pregnancy in Latina women. Matern. Child Health J. 2015;19:1842–1852. doi: 10.1007/s10995-015-1698-x.
253. Ghandali NY, Iravani M, Habibi A, Cheraghian B. The effectiveness of a pilates exercise program during pregnancy on childbirth outcomes: a randomised controlled clinical trial. BMC Pregnancy Childbirth. 2021;21:1–11
254. Ghodsi Z, Asltoghiri M. Effects of aerobic exercise training on maternal and neonatal outcome: a randomized controlled trial on pregnant women in Iran. J Pak Med Assoc 2014;64(9):1053–6.
255. Gil V, Osis M, Faundes A. Lumbar pain during pregnancy: efficacy of Global Postural Reeducation (GPR) treatment. Fisioter Pesqui. 2011;18(2) doi:10.1590/S1809-29502011000200011.
256. Giroux I, Inglis SD, Lander S, et al. Dietary intake, weight gain, and birth outcomes of physically active pregnant women: a pilot study. Appl Physiol Nutr Metab 2006;31:483–9.
257. Goletzke J, Buyken AE, Louie JC, Moses RG, BrandMiller JC. Dietary micronutrient intake during pregnancy is a function of carbohydrate quality. American Journal of Clinical Nutrition 2015;102(3):626–32
258. Goletzke J, De Haene J, Stotland NE, et al. Effect of a low-glycemic load diet intervention on maternal and pregnancy outcomes in obese pregnant women. Nutrients 2021;133. https://doi.org/10. 3390/nu13030748.
259. Gomez, J. G. Delgado, A. A. Agudelo, and H. Hurtado, “Diet effects on the perinatal result of obese pregnant patient,” Revista Colombiana de Obstetricia y Ginecolog´ıa, vol. 45, pp. 313–316, 1994 (Spanish).
260. Goodwin A, Astbury J, McMeeken J. Body image and psychological well-being in pregnancy. A comparison of exercisers and non-exercisers. Aust N Z J Obstet Gynaecol 2000;40:442–7
261. Granath A.B., Hellgren M.S., Gunnarsson R.K. Water Aerobics Reduces Sick Leave due to Low Back Pain during Pregnancy. J. Obstet. Gynecol. Neonatal Nurs. 2006;35:465–471. doi: 10.1111/j.1552-6909.2006.00066.x. -
262. Grant SM, Wolever TMS, O’Connor DL, Nisenbaum R, Josse RG (2011) Effect of a low glycaemic index diet on blood glucose in women with gestational hyperglycaemia. Diabetes Res Clin Pract 91:15–22
263. Gray-Donald K, Robinson E, Collier A, David K, Renaud L, Rodrigues S. Intervening to reduce weight gain in pregnancy and gestational diabetes mellitus in Cree communities: an evaluation. Can Med Assoc J 2000;163(10):1247–51.
264. Grivell R, Yelland L, Staehr CJ, Earl RA, Dodd J. The effect of antenatal dietary and lifestyle advice on fetal growth in women who are overweight or obese: findings from the LIMIT randomised trial. Ultrasound in Obstetrics and Gynecology 2013;42(Suppl 1):83.
265. Güder DS. The effect of childbirth preparation training on primiparous women’s birth plans and the childbirth processes. 2019
266. Güder, D. S., M. Yalvaç, and G. Vural. 2018. “The Effect of Pregnancy Pilates-Assisted Childbirth Preparation Training on Childbirth Fear and Neonatal Outcomes: A Quasi-Experimental/quantitative Research.” Quality & Quantity 52 (6): 2667–79. https://doi.org/10.1007/s11135-018-0682-0 .
267. Guelfi  KJ, Ong  MJ, Crisp  NA,  et al.  Regular exercise to prevent the recurrence of gestational diabetes mellitus: a randomized controlled trial.   Obstet Gynecol. 2016;128(4):819-827. doi:10.1097/aog.0000000000001632PubMedGoogle ScholarCrossref
268. Guelinckx I, Devlieger R, Mullie P, Vansant G. Effect of lifestyle intervention on dietary habits, physical activity, and gestational weight gain in obese pregnant women: a randomized controlled trial. Am J Clin Nutr 2010; 91: 373-80.
269. H Al Wattar B, Dodds J, Placzek A et al. Mediterranean-style diet in pregnant women with metabolic risk factors (ESTEEM). A pragmatic multicentre randomised trial. PLoS Med 2019; 16: e1002857. doi:10.1371/jou rnal.pmed.1002857
270. Haakstad  LA, Bø  K.  Effect of regular exercise on prevention of excessive weight gain in pregnancy: a randomised controlled trial.   Eur J Contracept Reprod Health Care. 2011;16(2):116-125. doi:10.3109/13625187.2011.560307PubMedGoogle ScholarCrossref
271. Haakstad L, Bo K. Effect of supervised aerobic dance exercise in prevention of excessive weight gain in pregnancy: a single blind randomized controlled trial. International Journal of Gynecology & Obstetrics 2009;107(Suppl 2):S198.
272. Haakstad L, Bo K. Exercise during pregnancy-Does it impact offspring birth weight parameters?. Journal of Science and Medicine in Sport 2012;15(Suppl 1):S340.
273. Haakstad L, Bø K. Exercise in pregnant women and birth weight: a randomized controlled trial. BMC Pregnancy Childbirth 2011;11:66.
274. Haakstad LA, Bo K. Effect of a regular exercise programme on pelvic girdle and low back pain in previously inactive pregnant women: a randomized controlled trial. J Rehabil Med 2015;47(3):229–34
275. Haakstad, L.A.; Torset, B.; Bø, K. What is the effect of regular group exercise on maternal psychological outcomes and common pregnancy complaints? An assessor blinded RCT. Midwifery 2016, 32, 81–86
276. Haakstad, L.A.; Vistad, I.; Sagedal, L.R.; Lohne-Seiler, H.; Torstveit, M.K. How does a lifestyle intervention during pregnancy influence perceived barriers to leisure-time physical activity? The Norwegian fit for delivery study, a randomized controlled trial. BMC Pregnancy Childbirth 2018, 18, 127
277. Haby K, Glantz A, Hanas R, et al. Mighty mums - an antenatal health care intervention can reduce gestational weight gain in women with obesity. Midwifery 2015;31:685–92.
278. Haby K., Berg M., Gyllensten H., Hanas R., Premberg A. Mighty Mums—A lifestyle intervention at primary care level reduces gestational weight gain in women with obesity. BMC Obes. 2018;5:16. doi: 10.1186/s40608-018-0194-4.
279. Hall DC, Kaufmann DA. Effects of aerobic and strength conditioning on pregnancy outcomes. Am J Obstet Gynecol. 1987;157(5):1199–203.
280. Halse RE, Wallman KE, Dimmock JA, Newnham JP, Guelfi KJ. Home-Based Exercise Improves Fitness and Exercise Attitude and Intention in Women with GDM. Med Sci Sports Exerc. 2015;47(8):1698-704.
281. Halse RE, Wallman KE, Newnham JP, et al. Home-based exercise training improves capillary glucose profile in women with gestational diabetes. Med Sci Sports Exerc 2014;46:1702–9.
282. Halse RE, Wallman KE, Newnham JP, et al. Pregnant women exercise at a higher intensity during 30 min of self-paced cycling compared with walking during late gestation: implications for 2 h postprandial glucose levels. Metabolism 2013;62:801–7.
283. Halvorsen S, Haakstad LA, Edvardsen E, Bo K. Effect of aerobic dance on cardiorespi- ratory fitness in pregnant women: a randomised controlled trial. Physiotherapy 2013;99(1):42–8.
284. Hanan Sayed EM, Adly Ali S, Mohamed Mostafa R AP, et al. Effect of Antenatal Exercises on Umbilical Blood Flow and Neonate Wellbeing in Diabetic Pregnant Women. Indian Journal of Physiotherapy & Occupational Therapy 2012;6:121–5.
285. Harden SM, Beauchamp MR, Pitts BH et al. Group-based life-style sessions for gestational weight gain management: a mixed method approach. Am J Health Behav 2014;38(4): 560–569.39.
286. Harizopoulou VC, Kritikos A, Papanikolaou Z, et al. Maternal physical activity before and during early pregnancy as a risk factor for gestational diabetes mellitus. Acta Diabetol 2010;47(S1):83–9.
287. Harris ST, Liu J, Wilcox S, et al. Exercise during pregnancy and its association with gestational weight gain. Matern Child Health J 2015;19:528–37.
288. Harrison  CL, Lombard  CB, Strauss  BJ, Teede  HJ.  Optimizing healthy gestational weight gain in women at high risk of gestational diabetes: a randomized controlled trial.   Obesity (Silver Spring). 2013;21(5):904-909. doi:10.1002/oby.20163PubMedGoogle
289. Harrison CL, Lombard CB, Gibson-Helm M, Deeks A, Teede HJ. Limiting excess weight gain in high-risk pregnancies: A randomized controlled trial. Endocrine Reviews 2011; 32(3 Meeting Abstracts):P1–466.
290. Harrison CL, Lombard CB, Teede HJ. Limiting postpartum weight retention through early antenatal intervention: the HeLP-her randomised controlled trial. Int J Behav Nutr Phys Act 2014;11:134.
291. Harrison CL, Teede HJ, Lombard CB. How effective is self-weighing in the setting of a lifestyle intervention to reduce gestational weight gain and postpartum weight retention?. Australian & New Zealand Journal of Obstetrics & Gynaecology 2014;54:382–5.
292. Haruna, M., Watanabe, E., Matsuzaki, M., Ota, E., Shiraishi, M., Murayama, R., Yoshida, M., Yeo, S., 2013. The effects of an exercise program on health-related quality of life in postpartum mothers: a randomized controlled trial. Health 05 (03), 8.
293. Hatch MC, Shu X-O, McLean DE, et al. Maternal exercise during pregnancy, physical fitness, and fetal growth. Am J Epidemiol 1993;137:1105–14.
294. Hauth JC, Gilstrap LC, Widmer K. Fetal heart rate reactivity before and after maternal jogging during the third trimester. Am J Obstet Gynecol 1982;142:545–7.
295. Hawkins  M, Hosker  M, Marcus  BH,  et al.  A pregnancy lifestyle intervention to prevent gestational diabetes risk factors in overweight Hispanic women: a feasibility randomized controlled trial.   Diabet Med. 2015;32(1):108-115. doi:10.1111/dme.12601PubMedGoogle
296. Hayes L, Bell R, Robson S, Poston L. Association between physical activity in obese pregnant women and pregnancy outcomes: The UPBEAT Pilot Study. Annals of Nutrition & Metabolism 2014;64(3-4):239–46.
297. Hayman M, Reaburn P, Browne M, Vandelanotte C, Alley S, Short CM. Feasibility, acceptability and efficacy of a web-based computer-tailored physical activity intervention for pregnant women - the Fit4Two randomised controlled trial. BMC Pregnancy Childbirth 2017 Mar 23;17(1):96
298. Hegaard HK, Petersson K, Hedegaard M, Ottesen B, Dykes AK, Henriksen TB, et al. Sports and leisure-time physical activity in pregnancy and birth weight: a population-based study. Scand J Med Sci Sports. 2010;20(1):e96– 102.
299. Heh, S.S., Huang, L.H., Ho, S.M., Fu, Y.Y., Wang, L.L., 2008. Effectiveness of an exercise support program in reducing the severity of postnatal depression in Taiwanese women. Birth (Berkeley, Calif.) 35, 60–65. Jarbou, N.S., Newell, K.A., 2022. Exercise and yoga during pregnancy and their impact on depression: a systematic literature review. Arch. Womens Ment. Health 25, 539–559.
300. Hellenes OM, Vik T, Løhaugen GC, Salvesen K, Stafne SN, Mørkved S, et al. Regular moderate exercise during pregnancy does not have an adverse effect on the neurodevelopment of the child. Acta paediatrica (Oslo, Norway : 1992). 2015; https://doi.org/10.1111/apa.12890.
301. Herrera-Perdigon J., Hopkins E., Marcalle M., Brooten D., Youngblut J., Lizardo M. Weight gain in high-risk pregnant women: Comparison by primary diagnosis and type of care. Clin. Excell. Nurse Pract. 2005;9:195–201.
302. Herring Cruice J., Bennett G., Rose M., Davey A., Foster G. Preventing excessive gestational weight gain among African American women: A randomized clinical trial. Obesity. 2016;24:30–36. doi: 10.1002/oby.21240. 81. Willcox J., Wilkinson S., Lappas M., Ball K., Crawford D., McCarthy E., Fjeldsoe B.Whittaker R., Maddison R., Campbell K. A mobile health intervention promoting healthy gestational weight gain for women entering pregnancy at a high body mass index: The txt4two pilot randomised controlled trial. BJOG. 2017;124:1718–1728. doi: 10.1111/1471-0528.14552
303. Herring SJ, Cruice JF, Bennett GG, Davey A, Foster GD. Using technology to promote postpartum weight loss in urban, low income mothers: a pilot randomized controlled trial. J Nutr Educ Behav 2014; 46: 610–615.
304. Herring SJ, Cruice JF, Bennett GG, Rose MZ, Davey A, Foster GD. Preventing excessive gestational weight gain among African American women: a randomized clinical trial. Obesity (Silver Spring). 2016;24(1):30-36. doi:10. 1002/oby.21240
305. Hollingsworth DR, Moore TR. Postprandial walking exercise in pregnant insulindependent (type I) diabetic women: reduction of plasma lipid levels but absence of a significant effect on glycemic control. Am J Obstet Gynecol 1987;157:1359–63.
306. Hopkins S, Baldi JC, Cutfield WS, McCowan L, Hofman PL. Effects of exercise training on maternal hormonal changes in pregnancy. Clin Endocrinol 2011;74:495–500.
307. Hopkins SA, Baldi JC, Cutfield WS et al. Exercise training in pregnancy reduces offspring size without changes in maternal insulin sensitivity. J Clin Endocrinol Metab 2010; 95(5):2080–2088.
308. Hoppu U, Isolauri E, Koskinen P, Laitinen K. Diet and blood lipids in 1-4 year-old children. Nutrition Metabolism and Cardiovascular Diseases 2013;23(10):980–6
309. Hoppu U, Isolauri E, Laakso P, Matomaki J, Laitinen K. Probiotics and dietary counselling targeting maternal dietary fat intake modifies breast milk fatty acids and cytokines. European Journal of Nutrition 2012;51(2):211–9 8. Laitinen K, Ilmonen J, Isolauri E. Dietary counselling and probiotic intervention during pregnancy modify postpartum adiposity. Annals of Nutrition and Metabolism 2011;58(Suppl 3):87
310. Horan M K, McGowan C A, Gibney E R, Byrne J, Donnelly J M, McAuliffe F M. Maternal nutrition and glycaemic index during pregnancy impacts on offspring adiposity at 6 months of age analysis from the ROLO randomised controlled trial. Nutrients. 2016;8(01):13–15. [PMC free article] [PubMed] [Google Scholar]
311. Horan M, Donnelly J, Gibney E, McGowan C, McAuliffe F. The association of maternal characteristics and macronutrient intake in pregnancy with neonatal body composition. The Power of Programming 2014: International Conference on Developmental Origins of Adiposity and Long-Term Health; 2014 March 13-15; Munich, Germany. 2014
312. Horan M, Donnelly J, McGowan C, Gibney E, McAuliffe F. The association between maternal nutrition and lifestyle during pregnancy and 2-year-old offspring adiposity: analysis from the ROLO study. J Public Health. 2016;24(5):427-436.
313. Horan M, McGowan C, Donnelly J, Gibney E, McAuliffe F. Maternal diet and weight at 3 months partum following a pregnancy intervention with a low glycaemic index diet: Results from the ROLO randomised control trial. Archives of Disease in Childhood. Fetal and Neonatal Edition 2014;99 (Suppl 1):A129-A130, Abstract no: PMM.20
314. Horan MK, McGowan CA, Doyle O, McAuliffe FM. Well-being in pregnancy: An examination of the effect of socioeconomic, dietary and lifestyle factors including impact of a low glycaemic index dietary intervention. European Journal of Clinical Nutrition 2014;68(1):19–24
315. Horan MK, McGowan CA, Gibney ER, Byrne J, Donnelly JM, McAuliffe FM. Maternal nutrition and glycaemic index during pregnancy impacts on offspring adiposity at 6 months of age-analysis from the ROLO randomised controlled trial. Nutrients. 2016;8(1):7.
316. Horan MK, McGowan CA, Gibney ER, Donnelly JM, McAuliffe FM. Maternal low glycaemic index diet, fat intake and postprandial glucose influences neonatal adiposity - secondary analysis from the ROLO study. Nutrition Journal 2014;13(1):78.
317. Horns PN, Ratcliffe LP, Leggett JC, Swanson MS. Pregnancy outcomes among active and sedentary primiparous women. Journal of Obstetric, Gynecologic, & Neonatal Nursing. 1996;25(1):4954.
318. Huang T T, Yeh C Y, Tsai Y C. A diet and physical activity intervention for preventing weight retention among Taiwanese childbearing women: a randomised controlled trial. Midwifery. 2011;27(02):257– 264. [PubMed] [Google Scholar]
319. Huberty J, Buman M, Leiferman J, Bushar J, Hekler E, Adams MJ. Dose and timing of text messages for increasing physical activity among pregnant women: a randomized controlled trial. Transl Behav Med 2017 Jun;7(2):212-223
320. Hui  AL, Back  L, Ludwig  S,  et al.  Effects of lifestyle intervention on dietary intake, physical activity level, and gestational weight gain in pregnant women with different pre-pregnancy body mass index in a randomized control trial.   BMC Pregnancy Childbirth. 2014;14:331. doi:10.1186/1471-2393-14-331PubMedGoogle ScholarCrossref
321. Hui A, Back L, Ludwig S, Gardiner P, Sevenhuysen G, Dean H, et al. Lifestyle intervention on diet and exercise reduced excessive gestational weight gain in pregnant women under a randomised controlled trial. BJOG 2012;119(1): 70–7.
322. Hui, A. L., Ludwig, S., Gardiner, P., Sevenhuysen, G., Murray, R., Morris, M., & Shen, G. X. (2006). Community-based exercise and dietary intervention during pregnancy: a pilot study. Canadian Journal of Diabetes, 30, 1–7.
323. Huseinovic E, Winkvist A, Bertz F, Berte ́us Forslund H, Brekke HK. Eating frequency, energy intake and body weight during a successful weight loss trial in overweight and obese postpartum women. Eur J Clin Nut 2014;68(1):71–6.
324. Hyun A-H, Cho J-Y, Koo J-H, editors. Effect of home-based tele-Pilates intervention on pregnant women: a pilot study. Healthcare: MDPI; 2022.
325. Hyun A-H, Jeon Y-J. Effect of mat pilates on body fluid composition, pelvic stabilization, and muscle damage during pregnancy. Appl Sci. 2020;10(24):9111.
326. Ickovics JR, Earnshaw V, Lewis JB, et al. Cluster randomized controlled trial of group prenatal care: perinatal outcomes among adolescents in New York City health centers. Am J Public Health. 2016;106(2):359-365.
327. Ilmonen J., Isolauri E., Poussa T., Laitinen K. Impact of dietary counselling and probiotic intervention on maternal anthropometric measurements during and after pregnancy: A randomized placebo-controlled trial. Clin. Nutr. 2011;30:156–164. doi: 10.1016/j.clnu.2010.09.009.
328. Iqbal R, Rafique G, Badruddin S, et al. Increased body fat percentage and physical inactivity are independent predictors of gestational diabetes mellitus in South Asian women. Eur J Clin Nutr 2007;61:736–42.
329. Jaakkola J, Isolauri E, Poussa T, Laitinen K. Benefits of repeated individual dietary counselling in long-term weight control in women after delivery. Maternal & Child Nutrition 2015;11:1041–8.
330. Jackson R., Stotland N., Caughey A., Gerbert B. Improving diet and exercise in pregnancy with Video Doctor counseling: A randomized trial. Patient Educ. Couns. 2011;83:203–209. doi: 10.1016/j.pec.2010.05.019.
331. Jefferies K, Shub A, Walker S, Hiscock R, Permezel M.Reducing excessive weight gain in pregnancy:a randomised controlled trial. Med J Aust. 2009;191:429–33
332. Jing W, Huang Y, Liu X, et al. The effect of a personalized intervention on weight gain and physical activity among pregnant women in China. Int J Gynaecol Obstet 2015;129:138–41.
333. Jovanovic L, Kessler A, Peterson CM. Human maternal and fetal response to graded exercise. J Appl Physiol 1985;58:1719–22.
334. Jovanovic-Peterson L, Durak EP, Peterson CM. Randomized trial of diet versus diet plus cardiovascular conditioning on glucose levels in gestational diabetes. Am J Obstet Gynecol 1989;161:415–9.
335. Juhl M, Kogevinas M, Andersen PK, et al. Is swimming during pregnancy a safe exercise? Epidemiology 2010;21:253–8.
336. Kafatos AG, Vlachonikolis I, Codrington C. Nutrition during pregnancy: the effects of an educational intervention program in Greece. Am J Clin Nutr 1989;50:970–9.
337. Kalayeh S, Nikbakht R, Seddigi S. The effect of exercise during pregnancy on mother’s depression in zahedan. International Journal of Pharmacy and Technology 2016;8:12248–62
338. Kamali M, Jafari E, Mazloom Zadeh S. The effect of “sitting pelvic tilt exercise” on low back pain and sleep disorder in primigravidas during the third trimester. Qom Univ Med Sci J. 2009;3(3):45–50.
339. Kannieappan LM, Deussen AR, Moran LJ, Grivell RM, Yelland LN, Dodd JM. The effect of antenatal dietary advice on maternal body composition in women who are overweight or obese - findings from the limit randomised trial. Journal of Paediatrics and Child Health  2013;49 Suppl2:94.
340. Kardel KR, Kase T. Training in pregnant women: effects on fetal development and birth. Am J Obstet Gynecol 1998; 178(2):280–286.
341. Kariminia A, Chamberlain ME, Keogh J, Shea A. Randomised controlled trial of effect of hands and knees posturing on incidence of occiput posterior position at birth. BMJ. 2004;328(7438):490.
342. Kasawara KT, Burgos CS, do Nascimento SL, Ferreira NO, Surita FG, Pinto ESJL. Maternal and Perinatal Outcomes of Exercise in Pregnant Women with Chronic Hypertension and/or Previous Preeclampsia: A Randomized Controlled Trial. ISRN Obstet Gynecol. 2013;2013:857047.
343. Keller, C., Ainsworth, B., Records, K., Todd, M., Belyea, M., Vega-Lopez, ´ S., Permana, P., Coonrod, D., Nagle-Williams, A., 2014. A comparison of a social support physical activity intervention in weight management among post-partum latinas. BMC Public Health 14, 971.
344. Kennelly MA, Ainscough K, Lindsay KL, O Sullivan E, Gibney ER, McCarthy M, et al. Pregnancy exercise and nutrition with smartphone application support: a randomized controlled trial. Obstet Gynecol 2018 May;131(5):818-826. [doi: 10.1097/AOG.0000000000002582] [Medline: 29630009]
345. Khaledan, S. Midar, N. S. Motahari Tabari, and M. Ahmad Shirvani, “Effect of an aerobic exercise program on fetal growth in pregnant women,” Journal of HAYAT, vol. 16, no. 1, pp. 55–64, 2010.
346. Khoury J, Henriksen T, Christophersen B, Tonstad S. Effect of a cholesterol-lowering diet on maternal, cord, and neonatal lipids, and pregnancy outcome: a randomized clinical trial. Am J Obstet Gynecol 2005;193:1292– 301.
347. Khoury J., Henriksen T., Christophersen B. & Tonstad S. (2005) Effect of a cholesterol-lowering diet on maternal, cord, and neonatal lipids, and pregnancy outcome: a randomized clinical trial. American Journal of Obstetrics and Gynecology 193, 1292–1301
348. Kiani Asiabar A, Amin Shokravi F, Hajifaraji M, Zayeri F. The effect of an educational intervention in early pregnancy with spouse’s participation on optimal gestational weight gain in pregnancy: a randomized controlled trial. Health Educ Res. 2018;33(6):535-547. doi:10.1093/her/cyy040
349. Kieffer EC, Welmerink DB, Sinco BR, Welch KB, Rees Clayton EM, Schumann CY, et al. Dietary outcomes in a Spanish-language randomized controlled diabetes prevention trial with pregnant Latinas. American Journal of Public Health 2014;104(3):526–33.
350. Kihlstrand M, Stenman B, Nilsson S, Axelsson O. Water-gymnastics reduced the intensity of back/low back pain in pregnant women. Acta Obstet Gynecol Scand. 1999;78(3):180. https://doi.org/10.1080/j. 1600- 0412.1999.780302.x.
351. Kim H-B, Hyun A-H. Psychological and biochemical effects of an online pilates intervention in pregnant women during COVID-19: a randomized pilot study. Int J Environ Res Public Health. 2022;19(17):10931
352. Kırıcı, P., M. C. Nacar, T. Karaçor, M. Bülbül, M. Baktıroğlu, N. Mavral, and N. Kaya. 2021. “Pilates Workouts Can Improve the Labor and Newborn Outcomes: A Case Control Study.” Journal of Surgery and Medicine 5 (6): 642–7. https://doi.org/10.28982/josam.951898 .
353. Kinnunen TI, Pasanen M, Aittasalo M et al. Preventing excessive weight gain during pregnancy - a controlled trial in primary health care. Eur J Clin Nutr 2007; 61: 884-91.
354. Kinnunen TI, Raitanen J, Aittasalo M, Luoto R. Preventing excessive gestational weight gain-a secondary analysis of a cluster-randomised controlled trial. Eur J Clin Nut 2012;66(12): 1344–50.
355. Kizirian N, Garnett S, Markovic T, Ross G, Louie J, Muirhead R, et al. Effects of a low-glycaemic index diet during pregnancy on offspring body composition: a pilot study. Obesity Research and Clinical Practice 2013;7(Suppl 2):e103
356. Kizirian N, Garnett S, Markovic T, Ross G, Muirhead R, Brodie S, et al. Maternal diet and infant body composition in women at risk of gestational diabetes mellitus. Obesity Research and Clinical Practice 2014;8(Suppl 1):55 20. Markovic TP, Muirhead R, Overs S, Kizirian N, Louie J, Sweeting A, et al. Predictors of birthweight in women at high risk of gestational diabetes mellitus. Obesity Research and Clinical Practice 2013;7(2):e3–4
357. Kizirian NV, Kong Y, Muirhead R, et al. Effects of a low-glycemic index diet during pregnancy on offspring growth, body composition, and vascular health: a pilot randomized controlled trial. Am J Clin Nutr 2016;1034:1073e82. https://doi.org/10.3945/ajcn.115. 123695.
358. Kluge J, Hall D, Louw Q, et al. Specific exercises to treat pregnancy-related low back pain in a South African population. Int J Gynaecol Obstet 2011;113:187–91.
359. Knuist M, Bonsel GJ, Zondervan HA, Treffers PE. Low sodium diet and pregnancy-induced hypertension: a multi-centre randomised controlled trial. Br J Obstet Gynaecol 1998;105:430–4.
360. Ko CW, Napolitano PG, Lee SP, et al. Physical activity, maternal metabolic measures, and the incidence of gallbladder sludge or stones during pregnancy: a randomized trial. Am J Perinatol 2014;31:39–48.
361. Ko PC, Liang CC, Chang SD, Lee JT, Chao AS, Cheng PJ. A randomized controlled trial of antenatal pelvic floor exercises to prevent and treat urinary incontinence. Int Urogynecol J. 2011;22(1):17-22.
362. Ko YL, Yang CL, Fang CL, Lee MY, & Lin PC (2013). Community-based postpartum exercise program. Journal of Clinical Nursing, 22, 2122–2131. doi:10.1111/jocn.12117 [PubMed: 23398359]
363. Ko, Y.L., Yang, C.L., Chiang, L.C., 2008. Effects of postpartum exercise program on fatigue and depression during "doing-the-month" period. J. Nurs. Res. 16, 177–186.
364. Kobe H, Nakai A, Koshino T, Araki T. Effect of regular maternal exercise on lipid peroxidation levels and antioxidant enzymatic activities before and after delivery. J Nippon Med Sch. 2002;69(6):5428.
365. Koivusalo  SB, Rönö  K, Klemetti  MM,  et al.  Gestational diabetes mellitus can be prevented by lifestyle intervention: the Finnish gestational diabetes prevention study (RADIEL): a randomized controlled trial.   Diabetes Care. 2016;39(1):24-30. Published correction appears in Diabetes Care. 2017 Aug;40(8):1133. doi:10.2337/dc15-0511PubMedGoogle ScholarCrossref
366. Kokic, I.S.; Ivanisevic, M.; Uremovic, M.; Kokic, T.; Pisot, R.; Simunic, B. Effect of therapeutic exercises on pregnancy-related low back pain and pelvic girdle pain: Secondary analysis of a randomized controlled trial. J. Rehabil. Med. 2017, 49, 251–257
367. Kolu P, Raitanen J, Luoto R. Physical activity and health-related quality of life during pregnancy: a secondary analysis of a cluster-randomised trial. Matern Child Health J 2014;18(9):2098–105
368. Kong K L, Campbell C G, Foster R C, Peterson A D, Lanningham-Foster L. A pilot walking program promotes moderate-intensity physical activity during pregnancy. Med Sci Sports Exerc. 2014;46(03):462– 471. [PubMed] [Google Scholar]
369. Kopacz SM, Ishoof SB. Perinatal outcomes in nutritionally monitored obese pregnant women: a randomized clinical trial. J Natl Med Assoc 2009;101:569–77. Vesco KK, Karanja N, King JC, Gillman MW, Leo MC, Perrin N, et al. Efficacy of a group-based dietary intervention for limiting gestational weight gain among obese women: a randomized trial. Obesity (Silver Spring) 2014;22:1989–96.
370. Kordi R, Abolhasani M, Rostami M, et al. Comparison between the effect of lumbopelvic belt and home based pelvic stabilizing exercise on pregnant women with pelvic girdle pain; a randomized controlled trial. J Back Musculoskelet Rehabil 2013;26:133–9.
371. Korpi-Hyövälti E A, Laaksonen D E, Schwab U S et al. Feasibility of a lifestyle intervention in early pregnancy to prevent deterioration of glucose tolerance. BMC Public Health. 2011;11:179. [PMC free article] [PubMed] [Google Scholar]
372. Korpi-Hyövälti E, Heinonen S, Schwab U, et al. Effect of intensive counselling on physical activity in pregnant women at high risk for gestational diabetes mellitus. A clinical study in primary care. Prim Care Diabetes 2012;6:261–8
373. Korpi-Hyövälti E, Schwab U, Laaksonen DE, Linjama H, Heinonen S, Niskanen L. Effect of intensive counselling on the quality of dietary fats in pregnant women at high risk of gestational diabetes mellitus. Br J Nutr. 2012;108 (5):910-917. doi:10.1017/S0007114511006118
374. Krukowski, D. West, M. DiCarlo, K. Shankar, M. Cleves, E. Tedford, A. Andres, A behavioral intervention to reduce excessive gestational weight gain, Maternal Child Health J. 21 (3) (2017) 485–491.
375. Kuhrt K, Harmon M, Hezelgrave NL, Seed PT, Shennan AH. Is recreational running associated with earlier delivery and lower birth weight in women who continue to run during pregnancy? An international retrospective cohort study of running habits of 1293 female runners during pregnancy. BMJ Open Sport Exerc Med. 2018;4:e000296.
376. Kulpa PJ, White BM, Visscher R. Aerobic exercise in pregnancy. Am J Obstet Gynecol. 1987;156(6):1395-403.
377. Kunath  J, Gunther  J, Rauh  K,  et al.  Effects of a lifestyle intervention during pregnancy to prevent excessive gestational weight gain in routine care—the cluster-randomised GeliS trial.   BMC Med. 2019;17(1):5. doi:10.1186/s12916-018-1235-zPubMedGoogle ScholarCrossref
378. Kusin JA, Kardjati S, Houtkooper JM, Renqvist UH. Energy supplementation during pregnancy and postnatal growth. Lancet 1992;340:623–6.
379. Labonte-Lemoyne E, Curnier D, Ellemberg D. Exercise during pregnancy enhances cerebral maturation in the newborn: a randomized controlled trial. Journal of Clinical & Experimental Neuropsychology: official. J Int Neuropsychol Soc. 2017;39(4):347–54.
380. Laitinen K, Poussa T, Isolauri E, Nutrition, Allergy, Mucosal Immunology and Intestinal Microbiota Group. Probiotics and dietary counselling contribute to glucose regulation during and after pregnancy: a randomised controlled trial. British Journal of Nutrition 2009;101(11): 1679–87
381. Landon M, Spong C, Thom E, Carpenter MW, Ramins S,Casey B, et al. A multicenter, randomised trial oftreatment for mild gestational diabetes. N Engl J Med.2009;361:1339–48
382. Lawani MM, Alihonou E, Akplogan B, et al. [Effect of antenatal gymnastics on childbirth: a study on 50 sedentary women in the Republic of Benin during the second and third quarters of pregnancy]. Sante 2003;13:235–41.
383. Lee, S. Challenger, M. McNabb, and M. Sheridan, “Exercise in pregnancy,” Modern Midwife, vol. 6, no. 8, pp. 28–33, 1996.
384. Leermakers EA, Anglin K, Wing RR. Reducing postpartum weight retention through a correspondence intervention. Int J Obes Relat Metab Disord 1998;22(11):1103–9
385. Leng J, Liu G, Zhang C, et al. Physical activity, sedentary behaviors and risk of gestational diabetes mellitus: a population-based cross-sectional study in Tianjin, China. Eur J Endocrinol 2016;174:763–73.
386. Lesser KB, Gruppuso PA, Terry RB, et al. Exercise fails to improve postprandial glycemic excursion in women with gestational diabetes. J Matern Fetal Med 1996;5:211–7.
387. Lewis, B.A., Gjerdingen, D.K., Avery, M.D., Sirard, J.R., Guo, H., Schuver, K., Marcus, B. H., 2014. A randomized trial examining a physical activity intervention for the prevention of postpartum depression: the healthy mom trial. Ment. Health Phys. Act. 7, 42–49.
388. Li Q, Xiong R, Wang L, et al. Associations of dietary habits, physical activity and cognitive views with gestational diabetes mellitus among Chinese women. Public Health Nutr 2014;17:1850–7.
389. Li, L. J., Aris, I. M., Han, W. M., & Tan, K. H. (2019). A promising food-coaching intervention program to achieve optimal gestational weight gain in overweight and obese pregnant women: pilot randomized controlled trial of a smartphone app. JMIR Formative Research, 3, e13013.
390. Lindholm ES, Norman M, Kilander CP, Altman D. Weight control program for obese pregnant women. Acta Obstet Gynecol Scand 2010; 89: 840-43.
391. Lindqvist M, Lindkvist M, Eurenius E, et al. Leisure time physical activity among pregnant women and its associations with maternal characteristics and pregnancy outcomes. Sexual & Reproductive Healthcare 2016;9:14–20.
392. Liu J, Wilcox S, Whitaker K, et al. Preventing excessive weight gain during pregnancy and promoting postpartum weight loss: a pilot lifestyle intervention for overweight and obese African American Women. Matern Child Health J 2015;19:840–9.
393. Liu, Y.H., Chang, C.F., Hung, H.M., Chen, C.H., 2021. Outcomes of a walking exercise intervention in postpartum women with disordered sleep. J. Obstet. Gynaecol. Res. 47, 1380–1387. Low, D.M., Bentley, K.H., Ghosh, S.S., 2020. Automated assessment of psychiatric disorders using speech: a systematic review. Laryngoscope Investig. Otolaryngol. 5, 96–116. LS, R., 1977. The CES-D Scale: a self-report depression scale for research in the general population. Appl. Psychol. Meas. 1, 385–401.
394. Lombard C, Harrison C, Teede H. A randomized controlled trial investigating self-weighing and the prevention of excess weight gain in early pregnancy. Endocrine Reviews 2011;32(3 Meeting Abstracts):P2–768.
395. Loprinzi PD, Cardinal BJ, Loprinzi KL, et al. Benefits and environmental determinants of physical activity in children and adolescents. Obes Facts 2012;5:597–610.
396. Lotgering FK, Spinnewijn WE, Struijk PC, et al. Respiratory and metabolic responses to endurance cycle exercise in pregnant and postpartum women. Int J Sports Med 1998;19:193–8.
397. Louie JCY, Markovic TP, Perera N, Foote D, Petocz P, Ross GP, Brand-Miller JC (2011) A randomized controlled trial investigating the effects of a low-glycemic index diet on pregnancy outcomes in gestational diabetes mellitus. Diabetes Care 34:2341–2346
398. Lovelady CA, Garner KE, Moreno KL, Williams JP. The effect of weight loss in overweight, lactating women on the growth of their infants. New Engl J Med 2000;342(7):449–53
399. Lu CL, Yu L. Study on the correlation between aerobic exercise during pregnancy and pregnancy outcome. Zhonghua Linchuang Yishi Zazhi 2014;8:3990–3.
400. Luke B, Brown MB, Misiunas R, Anderson E, Nugent C, van de Ven C, et al. Specialized prenatal care and maternal and infant outcomes in twin pregnancy. Am J Obstet Gynecol. 2003 Oct;189(4):934–938. PMID: 14586329. doi: 10.1067/S0002-9378(03)01054-8..
401. Luo X-D, Dong X, Zhou J. Effects of nutritional management intervention on gestational weight gain and perinatal outcome. Saudi Med J. 2014;35(10):1267–1270. [PMC free article] [PubMed] [Google Scholar]
402. Luoto R, Kinnunen TI, Aittasalo M, et al. Primary prevention of gestational diabetes mellitus and large-for-gestational-age newborns by lifestyle counseling: a clusterrandomized controlled trial. PLoS Med 2011;8:e1001036.
403. Luoto R, Nermes M, Laitinen K, Isolauri E. Impact of maternal probiotic-supplemented dietary counselling on pregnancy outcome and prenatal and postnatal growth: a double-blind, placebo-controlled study. Pediatric Academic Societies Annual Meeting; 2009 May 2-5; Baltimore, USA. 2009
404. Luoto R., Kinnunen T., Aittasalo M., Ojala K., Mansikkamaki K., Toropainen E., Kolu P., Vasankari T. Prevention of gestational diabetes: Design of a cluster-randomized controlled trial and one-year follow-up. BMC Pregnancy Childbirth. 2010;10:39. doi: 10.1186/1471-2393-10-39.
405. Lynch AM, Goodman C, Choy PL, Dawson B, Newnham JP, McDonald S, et al. Maternal physiological responses to swimming training during the second trimester of pregnancy. Res Sports Med. 2007;15(1):33-45.
406. Ma WJ, Huang ZH, Huang BX, Qi BH, Zhang YJ, Xiao BX, Li YH, Chen L, Zhu HL (2015) Intensive low-glycaemic-load dietary intervention for the management of glycaemia and serum lipids among women with gestational diabetes: a randomized control trial. Public Health Nutr 18:1506–1513
407. Maconochie N, Doyle P, Prior S, et al. Risk factors for first trimester miscarriage-results from a UK-population-based case-control study. BJOG 2007;114:170–86.
408. Madsen M, Jørgensen T, Jensen ML, et al. Leisure time physical exercise during pregnancy and the risk of miscarriage: a study within the Danish National Birth Cohort. BJOG 2007;114:1419–26.
409. Magann EF, Evans SF, Weitz B, et al. Antepartum, intrapartum, and neonatal significance of exercise on healthy low-risk pregnant working women. Obstet Gynecol 2002;99:466–72.
410. Magnus P, Trogstad L, Owe KM, et al. Recreational physical activity and the risk of preeclampsia: a prospective cohort of Norwegian women. Am J Epidemiol 2008;168:952–7.
411. Magriples  U, Boynton  MH, Kershaw  TS,  et al.  The impact of group prenatal care on pregnancy and postpartum weight trajectories.   Am J Obstet Gynecol. 2015;213(5):688.e1-688.e9. doi:10.1016/j.ajog.2015.06.066PubMedGoogle ScholarCrossref
412. Mahony R, Byrne J, Curran S, O’Herlihy C, McAuliffe F. A pilot study of the feasibility of a randomised trial of low glycaemic diet versus normal diet from early pregnancy in euglycaemic women. Archives of Disease in Childhood. Fetal and Neonatal Edition 2008;93(Suppl 1):Fa38.
413. Maitland RA, Barr S, Briley A, Seed P, Poston L. Incidence of gestational diabetes in an obese population using the International Association of Diabetes and Pregnancy Study Groups (IADPSG) criteria in the UK Pregnancies Better Eating and Activity Trial (UPBEAT) pilot study. Diabetic Medicine 2012;29(Suppl 1):152.
414. Manca A, Meloni M, Ventura L, Consoli G, Fenu S, Mura G et al. DoseResponse effects on LBP, sleep, and depression of online Pre-Partum and Post-Partum Pilates-Based programs. Res Q Exerc Sport. 2024:1–10.
415. Mardones-Santander F, Rosso P, Stekel A, Ahumada E, Llaguno S, Pizarro F, Salinas J, Vial I, Walter T. Effect of a milk-based food supplement on maternal nutritional status and fetal growth in underweight Chilean women. Am J Clin Nutr 1988;47:413–9.
416. Markovic 2016 ACTRN12610000681055. A randomized, two-arm parallel dietary intervention study to compare the effects of consuming a low glycemic diet or wholegrain high fibre diet on infant birth weight and body composition, complications related to Gestational Diabetes Mellitus (GDM) and progression to GDM diagnosis in women at high-risk of GDM. anzctr.org.au/Trial/Registration/ TrialReview.aspx?id=335632 Date first received: 18 August 2010
417. Markovic T., Muirhead R., Overs S., Ross G., Louie J., Kizirian N., et al. Randomized Controlled Trial Investigating the Effects of a Low-Glycemic Index Diet on Pregnancy Outcomes in Women at High Risk of Gestational Diabetes Mellitus: The GI Baby 3 Study. Diabetes Care. 2016;39:31–38. doi: 10.2337/dc15-0572.
418. Marquez-Sterling S, Perry AC, Kaplan TA, Halberstein RA, Signorile JF. Physical and psychological changes with vigorous exercise in sedentary primigravidae. Med Sci Sports Exerc 2000;32(1):58–62.
419. Mazzarino,M, D. Kerr, M.E. Morris, Feasibility of pilates for pregnant women: a randomised trial, J. Bodyw. Mov. Ther. 32 (2022) 207–212.
420. Martin CL, Brunner Huber LR. Physical activity and hypertensive complications during pregnancy: findings from 2004 to 2006 North Carolina Pregnancy Risk Assessment Monitoring System. Birth 2010;37:202–10
421. Martin J, MacDonald-Wicks L, Hure A, Smith R, Collins CE. Reducing postpartum weight retention and improving breastfeeding outcomes in overweight women: A pilot randomised controlled trial. Nutrients 2015;7(3):1465–79
422. Mason L, Roe B, Wong H, Davies J, Bamber J. The role of antenatal pelvic floor muscle exercises in prevention of postpartum stress incontinence: a randomised controlled trial. J Clin Nurs. 2010;19(1920):2777-86.
423. Mauriello, L. M., Van Marter, D. F., Umanzor, C. D., Castle, P. H., & de Aguiar, E. L. (2016). Using mHealth to deliver behavior change interventions within prenatal care at community health centers. American Journal of Health Promotion, 30, 554–562.
424. McAuley SE, Jensen D, McGrath MJ, et al. Effects of human pregnancy and aerobic conditioning on alveolar gas exchange during exercise. Can J Physiol Pharmacol 2005;83:625–33.
425. McAuliffe F. A randomised controlled trial of low glycaemic index carbohydrate diet versus no dietary intervention in the prevention of recurrence of foetal macrosomia. CurrentControlled Trials (www.controlled-trials.com/) (accessed 12.05.2010).
426. McAuliffe F. Maternal nutrition and fetal health. The Power of Programming 2014: International Conference on Developmental Origins of Adiposity and Long-Term Health; 2014 March 13-15; Munich, Germany. 2014
427. McCarthy  EA, Walker  SP, Ugoni  A, Lappas  M, Leong  O, Shub  A.  Self-weighing and simple dietary advice for overweight and obese pregnant women to reduce obstetric complications without impact on quality of life: a randomised controlled trial.   BJOG. 2016;123(6):965-973. doi:10.1111/1471-0528.13919PubMedGoogle ScholarCrossref
428. McCowan LME, Roberts CT, Dekker GA, Taylor RS, Chan EHY, Kenny LC, et al. Risk factors for small-for-gestational-age infants by customised birthweight centiles: data from an international prospective cohort study. BJOG. 2010; 117(13):1599–607.
429. McDonald EC, Pollitt E, Mueller W. The bacon chow study: maternal nutritional supplementation and birth weight of offspring. Am J Clin Nutr 1981;34:2133–44.
430. McEachan RRC, Santorelli G, Bryant M, et al. The HAPPY (Healthy and Active Parenting Programmme for early Years) feasibility randomised control trial: acceptability and feasibility of an intervention to reduce infant obesity. BMC Public Health. 2016;16(1):211-211.
431. McGiveron A, Foster S, Pearce J, et al. Limiting antenatal weight gain improves maternal health outcomes in severely obese pregnant women: findings of a pragmatic evaluation of a midwife-led intervention. Journal of Human Nutrition and Dietetics 2015;28(Suppl 1):29–37.
432. McGowan C A, Walsh J M, Byrne J, Curran S, McAuliffe F M. The influence of a low glycemic index dietary intervention on maternal dietary intake, glycemic index and gestational weight gain during pregnancy: a randomized controlled trial. Nutr J. 2013;12(01):140. [PMC free article] [PubMed] [Google Scholar]
433. McMurray RG, Hackney AC, Guion WK, et al. Metabolic and hormonal responses to low-impact aerobic dance during pregnancy. Medicine & Science in Sports & Exercise 1996;28:41–6.
434. Melo A. Exercise and pregnancy: randomised clinical trial. Current Controlled Trials (www.controlled-trials.com/)[accessed 31 October 2010].
435. Melzer K, Schutz Y, Soehnchen N, et al. Effects of recommended levels of physical activity on pregnancy outcomes. American Journal of Obstetrics & Gynecology. 2010;202(3):266.e1-6.
436. Menichini D, Petrella E, Dipace V, et al. The impact of an early lifestyle intervention on pregnancy outcomes in a cohort of insulin-resistant overweight and obese women. Nutrients 2020; 125. https://doi.org/10.3390/nu12051496.
437. Metcoff J, Costiloe P, Crosby WM, Dutta S, Sandstead HH, Milne D, Bodwell CE, Majors SH. Effect of food supplementation (WIC) during pregnancy on birth weight. Am J Clin Nutr 1985;41:933–47.
438. Miquelutti MA, Cecatti JG, Makuch MY. Evaluation of a birth preparation program on lumbopelvic pain, urinary incontinence, anxiety and exercise: a randomized controlled trial. BMC Pregnancy Childbirth. 2013;13:154.
439. Miquelutti, M.A.; Cecatti, J.G.; Makuch, M.Y. Evaluation of a birth preparation program on lumbopelvic pain, urinary incontinence, anxiety and exercise: A randomized controlled trial. BMC Pregnancy Childbirth 2013, 13, 154.
440. Mitchell, J., Field, T., Diego, M., Bendell, D., Newton, R., Pelaez, M., 2012. Yoga reduces prenatal depression symptoms. Psychology 0 (09), 5.
441. Mohammadi, F., Malakooti, J., Babapoor, J., Mohammad-Alizadeh-Charandabi, S., 2015. The effect of a home-based exercise intervention on postnatal depression and fatigue: a randomized controlled trial. Int. J. Nurs. Pract. 21, 478–485.
442. Mohseni-Bandpei MA, Ahmadshirvani M, Fakhri M, Rahmani N. The effect of an exercise program and ergonomic advices in the treatment of pregnancy related low back pain: a randomised controlled clinical trial. J Mazandaran Univ Med Sci. 2010;20(77):9–17.
443. Momeni Javid F, Simbar M, Dolatian M, et al. Comparison of lifestyles of women with gestational diabetes and healthy pregnant women. Glob J Health Sci 2015;7:162–9.
444. Montoya Arizabaleta AV, Orozco Buitrago L. Aguilar de Plata AC, Mosquera Escudero M, Ramirez-Velez R. Aerobic exercise during pregnancy improves health-related quality of life: a randomised trial. J Physiother 2010;56(4):253–8.
445. Mora JO, Clement J, Christiansen N, Suescun J, Wagner M, Herrera MG. Nutritonal supplementation and the outcome of pregnancy. III. Perinatal and neonatal mortality. Nutr Rep Int 1978;18:167–75.
446. Mørkrid K, Jenum AK, Berntsen S, et al. Objectively recorded physical activity and the association with gestational diabetes. Scand J Med Sci Sports 2014;24:e389–97.
447. Morkved S, Bo K, Schei B, Salvesen KA. Pelvic floor muscle training during pregnancy to prevent urinary incontinence: a single-blind randomized controlled trial. Obstet Gynecol. 2003;101(2):313-9.
448. Morkved S, Salvesen KA, Schei B, Lydersen S, Bo K. Does group training during preg- nancy prevent lumbopelvic pain? A randomized clinical trial. Acta Obstet Gynecol Scand 2007;86(3):276–82.
449. Morkved S. Effects of regular exercise during pregnancy.ClinicalTrials.gov(www.clinicaltrials.gov) (accessed 31 October 2010).
450. Morris DL, Berenson AB, Lawson J, Wiemann CM. Comparison of adolescent pregnancy outcomes by prenatal care source. J Reprod Med. 1993;38(5):375–80. PMID: 8320675.
451. Moses R., Casey S., Quinn E., Cleary J., Tapsell L., Milosavljevic M., Petocz P., Brand-Miller J.C. Pregnancy and Glycemic Index Outcomes study: Effects of low glycemic index compared with conventional dietary advice on selected pregnancy outcomes. Am. J. Clin. Nutr. 2014;99:517–523. doi: 10.3945/ajcn.113.074138.
452. Moses RG, Barker M, Winter M, Petocz P, Brand-Miller JC (2009) Can a low-glycemic index diet reduce the need for insulin in gestational diabetes mellitus? A randomized trial. Diabetes Care 32:996–1000
453. Moses RG, Casey S, Cleary J, Milosavljevic M, Quinn E, Tapsell L, et al. Effect of low glycaemic index dietary advice in normal pregnancy: The PREGGIO study. Obesity Research and Clinical Practice 2013;7:e34–5
454. Moses RG, Casey SA, Quinn EG, Cleary JM, Tapsell LC, Milosavljevic M, et al. Pregnancy and Glycemic Index Outcomes study: effects of low glycemic index compared with conventional dietary advice on selected pregnancy outcomes. American Journal of Clinical Nutrition 2014;99 (3):517–23
455. Moses RG, Luebke M, Petocz P, Brand-Miller JC. Maternal diet and infant size 2 y after the completion of a study of a low-glycemic-index diet in pregnancy. American Journal of Clinical Nutrition 2007;86(6):1806
456. Mottola MF, Giroux I, Gratton R et al. Nutrition and exercise prevent excess weight gain in overweight pregnant women. Med Sci Sports Exerc 2010; 42: 265-72.
457. Mottola MF, Inglis S, Brun CR, et al. Physiological and metabolic responses of late pregnant women to 40 min of steady-state exercise followed by an oral glucose tolerance perturbation. J Appl Physiol 2013;115:597–604.
458. Mujsindi W, Habash D, Childs G. Impact of nutrition education on gestational weight gain in obese pregnant women. American Journal of Obstetrics and Gynecology 2014;210(1 Suppl):S188.
459. Murtezani A, Pacarada M, Ibraimi Z, Nevzati A, Abazi N. The impact of exercise during pregnancy on neonatal outcomes: a randomised controlled trial. Journal of Sports Medicine and Physical Fitness 2014;54(6):802–8.
460. Mustila T, Raitanen J, Keskinen P, et al. Pragmatic controlled trial to prevent childhood obesity in maternity and child health care clinics: pregnancy and infant weight outcomes (The VACOPP Study). BMC Pediatr 2013;13:80.
461. Mustila T, Raitanen J, Keskinen P, Saari A, Luoto R. Lifestyle counseling during pregnancy and offspring weight development until four years of age: follow-up study of a controlled trial. J Negat Results Biomed. 2012;11:11. [PMC free article] [PubMed] [Google Scholar]
462. Muzik M, Hamilton SE, Rosenblum KL, Waxler E, & Hadi Z (2012). Mindfulness yoga during pregnancy for psychiatrically at-risk women: Preliminary results from a pilot feasibility study. Complementary Therapies in Clinical Practice, 18, 235–240. doi:10.1016/j.ctcp.2012.06.006 [PubMed: 23059438]
463. Nagle C, Skouteris H, Morris h, Nankervis A, Rasmussen B, Mayall P et al. Primary prevention of gestational diabetes for women who are overweight and obese: a randomised controlled trial. BMC Pregnancy Childbirth 2013; 13: 65.
464. Narendran S, Nagarathna R, Narendran V, et al. Efficacy of yoga on pregnancy outcome. J Altern Complement Med 2005;11:237–44.
465. Nascimento KLK, Surita SLN, Parpinelli FGS, Kasawara MAP. Type of delivery and neonatal outcome in overweight and obese pregnant women with excessive weight gain. Journal of Maternal-Fetal and Neonatal Medicine 2012;25(S2):73–4.
466. Nascimento S L, Surita F G, Parpinelli MÂ, Siani S, Pinto e Silva J L. The effect of an antenatal physical exercise programme on maternal/perinatal outcomes and quality of life in overweight and obese pregnant women: a randomised clinical trial. BJOG. 2011;118(12):1455–1463. [PubMed] [Google Scholar]
467. Nascimento GRSd F, PdMVWd VSO, Ayach W, Christofoletti G, Pegorare ABGS. Effect of pilates exercises on blood glucose, urinary problems, sexual symptoms, pain and anxiety in women with gestational diabetes mellitus. Hum Mov. 2021.
468. Nasiri-Amiri F, Bakhtiari A, Faramarzi M, et al. The Association Between Physical Activity During Pregnancy and Gestational Diabetes Mellitus: A Case-Control Study. Int J Endocrinol Metab 2016;14:e37123.
469. Navas, A., Carrascosa, M.D.C., Artigues, C., Ortas, S., Portells, E., Soler, A., Yanez, ˜ A.M., Bennasar-Veny, M., Leiva, A., 2021. Effectiveness of moderate-intensity aerobic water exercise during pregnancy on quality of life and postpartum depression: a multi-center, randomized controlled trial. J. Clin. Med. 10.
470. Newham JJ, Wittkowski A, Hurley J, et al. Effects of antenatal yoga on maternal anxiety and depression: a randomized controlled trial. Depress Anxiety 2014;31:631–40.
471. Newman AK, Deussen AR, Moran LJ, Grivell RM, Yelland LN, Turnbull D, et al. The effect of antenatal dietary and lifestyle advice on maternal psychological health in women who are overweight or obese-findings from the limit randomised trial. Journal of Paediatrics and Child Health 2013;49 Suppl 2:119.
472. Ney, D. R. Hollingsworth, and L. Cousins, “Decreased insulin requirement and improved control of diabetes in pregnant women given a high-carbohydrate, high-fiber, low-fat diet,” Diabetes Care, vol. 5, no. 5, pp. 529–533, 1982.
473. Nielsen CA, Sigsgaard I, Olsen M, Tolstrup M, Danneskiold-Samsoee B, Bock JE. Trainability of the pelvic floor. A prospective study during pregnancy and after delivery. Acta Obstet Gynecol Scand. 1988;67(5):437-40.
474. Niinivirta K, Isolauri E, Laakso P, Linderborg K, Laitinen K. Dietary counseling to improve fat quality during pregnancy alters maternal fat intake and infant essential fatty acid status. Journal of Nutrition 2011;141(7):1281–5 14. Niinivirta K, Laakso P, Linderborg K, Poussa T, Isolauri E, Laitinen K. Maternal dietary counseling during pregnancy and infant fatty acid profiles. International Journal of Food Sciences and Nutrition 2014;65(3):268–72
475. Nilsson K. Weight gain during pregnancy - a randomized controlled trial of intervention to prevent excessive gestational weight gain. ClinicalTrials.gov (http://clinicaltrials.gov/) [accessed 5 September 2014] 2009.
476. Nilsson-Wikmar L, Holm K, Oijerstedt R, Harms-Ringdahl K. Effect of three different physical therapy treatments on pain and activity in pregnant women with pelvic girdle pain: a randomized clinical trial with 3, 6, and 12 months follow-up postpartum. Spine (Phila Pa 1976). 2005;30(8):850–856. doi:10.1097/01. Brs.0000158870.68159.d9.
477. Nobles C, Marcus BH, Stanek 3rd EJ, et al. Effect of an exercise intervention on gesta- tional diabetes mellitus: a randomized controlled trial. Obstet Gynecol 2015;125(5): 1195–204.
478. Nobles C, Marcus BH, Stanek EJ3 et al. The Effect of an Exercise Intervention on Gestational Weight Gain. Am J Health Promot 2018; 32 736– 744. doi:10.1177/0890117117732409
479. Nobles C, Marcus BH, Stanek rEJ, Braun B, Whitcomb BW, Solomon CG, et al. Effect of an exercise intervention on gestational diabetes mellitus: a randomized controlled trial. Obstetrics and gynecology. 2015;125(5):1195.
480. Nobles C., Marcus B., Stanek E., Braun B., Whitcomb B., Manson J., et al. The Effect of an Exercise Intervention on Gestational Weight Gain: The Behaviors Affecting Baby and You (B.A.B.Y.) Study: A Randomized Controlled Trial. Am. J. Health Promot. 2018;32:736–744. doi: 10.1177/0890117117732409.
481. Norman, E., Sherburn, M., Osborne, R.H., Galea, M.P., 2010. An exercise and education program improves well-being of new mothers: a randomized controlled trial. Phys. Ther. 90, 348–355.
482. O’Connor M.J. & Whaley S.E. (2007) Brief intervention for alcohol use by pregnant women. American Journal of Public Health 97, 252–258.
483. O’Connor PJ, Poudevigne MS, Cress ME, et al. Safety and efficacy of supervised strength training adopted in pregnancy. J Phys Act Health 2011;8:309–20.
484. O’Toole ML, Sawicki MA, Artal R. Structured diet and physical activity prevent postpartum weight retention. J Womens Health 2003;12(10):991–8.
485. Oken E, Ning Y, Rifas-Shiman SL, et al. Associations of physical activity and inactivity before and during pregnancy with glucose tolerance. Obstetrics & Gynecology 2006;108:1200–7.
486. Okesene-Gafa  KAM, Li  M, McKinlay  CJD,  et al.  Effect of antenatal dietary interventions in maternal obesity on pregnancy weight-gain and birthweight: Healthy Mums and Babies (HUMBA) randomized trial.   Am J Obstet Gynecol. 2019;221(2):152e.1-152e.13. doi:10.1016/j.ajog.2019.03.003PubMedGoogle ScholarCrossref
487. Okido M, Valeri F, Martins W, Ferreira C, Duarte G, Cavalli R. Assessment of foetal wellbeing in pregnant women subjected to pelvic floor muscle training: a controlled randomised study. International Urogynecology Journal. 2015;26(10):1475-81.
488. Oktaviani I. Pilates workouts can reduce pain in pregnant women. Complement Ther Clin Pract. 2018;31:349–51.
489. Olsen C., Groth S., Graham M., Reschke J., Strawderman M., Fernandez D. The effectiveness of an online intervention in preventing excessive gestational weight gain: The e-moms roc randomized controlled trial. BMC Pregnancy Childbirth. 2018;18:148. doi: 10.1186/s12884-018-1767-4.
490. Olsen SF, Secher NJ, Tabor A, Weber T, Walker JJ, GluudC. Randomised clinical trials of fish oil supplementation inhigh risk pregnancies. BJOG. 2000;107:382–95
491. Olson CM, Strawderman MS, Reed RG. Efficacy of an intervention to prevent excessive gestational weight gain. Am J Obstet Gynecol 2004; 191: 530-36.
492. Olson CM, Groth SW, Graham ML, Reschke JE, Strawderman MS, Fernandez ID. The effectiveness of an online intervention in preventing excessive gestational weight gain: the e-moms roc randomized controlled trial. BMC Pregnancy Childbirth. 2018;18(1):148. doi:10.1186/s12884-018-1767-4
493. Ong M, Guelfi K, Hunter T et al. Supervised home-based exercise may attenuate the decline of glucose tolerance in obese pregnant women. Diabetes Metab 2009; 35(5):418–421.
494. Onwude JL, Lilford RJ, Hjartardottir H, Staines A, TuffnellD. A randomised double blind placebo controlled trial offish oil in high risk pregnancy. BJOG. 1995;102:95–100
495. Oostdam N, Bosmans J, Wouters MG, Eekhoff EM, van Mechelen W, van Poppel MN. Cost-effectiveness of an exercise program during pregnancy to prevent gestational diabetes: results of an economic evaluation alongside a randomised controlled trial. BMC Pregnancy Childbirth 2012;12:64.
496. Oostdam N, van Poppel M, Wouters M et al. No effect of the FitFor2 exercise programme on blood glucose, insulin sensitivity, and birthweight in pregnant women who were overweight and at risk for gestational diabetes: results of a randomised controlled trial. BJOG 2012; 119(9):1098–1107.
497. Oostdam N, Van Poppel MN, Eekhoff EM, Wouters MG, Van Mechelen W. Design of FitFor2 study: the effects of an exercise program on insulin sensitivity and plasma glucose levels in pregnant women at high risk for gestational diabetes. BMC Pregnancy and Childbirth 2009;9:1.
498. Opie RS, Neff M, Tierney AC. A behavioural nutrition intervention for obese pregnant women: effects on diet quality, weight gain and the incidence of gestational diabetes. Aust New Zealand J Obstetrics Gynaecol. 2016;56 (4):364–73. doi:10.1111/ajo.12474.
499. Orawan L, Preecha W. Effect of pelvic floor muscle training (PFMT) during pregnancy on bladder neck descend and delivery. Journal of the Medical Association of Thailand. 2014;97(Suppl 8):S156-63.
500. Osmundson SS, Norton ME, El-Sayed YY et al. Early Screening and Treatment of Women with Prediabetes. Am J Perinatol 2016; 33: 172–179. doi:10.1055/s-0035-1563715
501. Ostbye T, Krause KM, Lovelady CA, Morey MC, Bastian LA, Peterson BL, et al. Active Mothers Postpartum. A Randomized Controlled Weight-Loss Intervention Trial. Am J Prev Med 2009;37(3):173–80.
502. Ozdemir, S.; Bebis, H.; Ortabag, T.; Acikel, C. Evaluation of the efficacy of an exercise program for pregnant women with low back and pelvic pain: A prospective randomized controlled trial. J. Adv. Nurs. 2015, 71, 1926–1939
503. Ozkan, ¨ S.A., Kücükkelepce, D.S., Korkmaz, B., Yılmaz, G., Bozkurt, M.A., 2020. The effectiveness of an exercise intervention in reducing the severity of postpartum depression: a randomized controlled trial. Perspect.Psychiatr.Care 56, 844–850.
504. Patel N, Godfrey KM, Pasupathy D, et al. Infant adiposity following a randomised controlled trial of a behavioural intervention in obese pregnancy. Int J Obes (Lond). 2017;41(7):1018-1026.
505. Pawalia A., Kulandaivelan S., Savant S., Yadav V. Exercise in pregnancy: Effect on obesity parameters in indian women—A randomized controlled trial. Rom. J. Diabetes Nutr. Metab. Dis. 2017;24:315–323. doi:10.1515/rjdnmd-2017-0037.
506. Pawlak DB. Glycemic load and infant birth weight in pregnant overweight/obese women. ClinicalTrials.gov (http://clinicaltrials.gov/) (accessed 31 October 2010).
507. Peaceman AM, Kwasny MJ, Gernhofer N, Vincent E, Josefson JL, Van Horn L. 2: MOMFIT: a randomized clinical trial of an intervention to prevent excess gestational weight gain in overweight and obese women. Am J Obstet Gynecol. 2017;216(1)(suppl):S2-S3. doi:10.1016/j.ajog.2016.11.003
508. Peccei  A, Blake-Lamb  T, Rahilly  D, Hatoum  I, Bryant  A.  Intensive prenatal nutrition counseling in a community health setting: a randomized controlled trial.   Obstet Gynecol. 2017;130(2):423-432. doi:10.1097/AOG.0000000000002134PubMedGoogle ScholarCrossref
509. Pelaez  M, Gonzalez-Cerron  S, Montejo  R, Barakat  R.  Protective effect of exercise in pregnant women including those who exceed weight gain recommendations: a randomized controlled trial.   Mayo Clin Proc. 2019;94(10):1951-1959. doi:10.1016/j.mayocp.2019.01.050PubMedGoogle ScholarCrossref
510. Pelaez M, Gonzalez-Cerron S, Montejo R, Barakat R. Pelvic floor muscle training included in a pregnancy exercise program is effective in primary prevention of urinary incontinence: a randomized controlled trial. NeurourolUrodyn 2014;33(1):67–71.
511. Perales  M, Rodríguez  YC, Terrones  MV, Mulas  AL, Carballo  RB.  Exercise and depression in overweight and obese pregnant women: a randomised controlled trial.   Archivos de Medicina del Deporte. 2015(167):156-163. http://archivosdemedicinadeldeporte.com/articulos/upload/or04_perales.pdfGoogle Scholar
512. Perales M, Calabria I, Lopez C, et al. Regular exercise throughout pregnancy is associated with a shorter first stage of labor. Am J Health Promot 2016;30:149–54.
513. Perales M, Mateos S, Vargas M, Sanz I, Lucia A, Barakat R. Fetal and maternal heart rate responses to exercise in pregnant women. A randomized controlled trial. Archivos de Medicina del Deporte. 2015;32(6):361-7.
514. Perales M, Refoyo I, Coteron J, Bacchi M, Barakat R. Exercise during pregnancy attenuates prenatal depression: a randomized controlled trial. Eval Health Prof 2015; 38(1):59–72.
515. Perales M, Santos-Lozano A, Sanchis-Gomar F, Luaces M, Pareja-Galeano H, Garatachea N, et al. Maternal Cardiac Adaptations to a Physical Exercise Program during Pregnancy. Med Sci Sports Exerc. 2016;48(5):896-906.
516. Perales, M., Santos-Lozano, A., Sanchis-Gomar, F., Luaces, M., Pareja-Galeano, H., Garatachea, N., Barakat, R., Lucia, A., 2016. Maternal cardiac adaptations to a physical exercise program during pregnancy. Med. Sci. Sports Exerc. 48, 896–906.
517. Pérez-Ferre N, Galindo M, Fernández MD, Velasco V, Runkle I, de la Cruz MJ, et al. The outcomes of gestational diabetes mellitus after a telecare approach are not inferior to traditional outpatient clinic visits. Int J Endocrinol 2010;2010:386941 [FREE Full text] [doi: 10.1155/2010/386941] [Medline: 20628517]
518. Perichart-Perera O, Balas-Nakash M, Rodriguez-Cano A, Legorreta-Legorreta J, Parra-Covarrubias A, Vadillo-Ortega F (2012) Low glycemic index carbohydrates versus all types of carbohydrates for treating diabetes in pregnancy: a randomized clinical trial to evaluate the effect of glycemic control. Int J Endocrinol 2012:296017
519. Peterson CD, Haas M, Gregory WT. A pilot randomized controlled trial comparing the efficacy of exercise, spinal manipulation, and neuro emotional technique for the treatment of pregnancy-related low back pain. Chiropr Man Therap. 2012;20(1):18.
520. Petrella E, Facchinetti F, Bertarini V, Pignatti L, Neri I, Battistini NC. Occurrence of pregnancy complications in women with BMI > 25 submitted to a healthy lifestyle and eating habits program. American Journal of Obstetrics and Gynecology 2013;208(1 Suppl):S33–4.
521. Petrella E, Malavolti M, Bertarin V, Pignatti L, Neri I, Battistini NC, et al. Gestational weight gain in overweight and obese women enrolled in a healthy lifestyle and eating habits program. J Matern Fetal Neonata Med 2013;27:1348–52.
522. Petrella E, Malavolti M, Bertarini V, Pignatti L, Neri I, Battistini NC, et al. Gestational weight gain in overweight and obese women enrolled in a healthy lifestyle and eating habits program. Journal of Maternal-Fetal and Neonatal Medicine 2014;27(13):1348–52.
523. Petrov Fieril K, Glantz A, Fagevik Olsen M. The efficacy of moderate-to-vigorous re- sistance exercise during pregnancy: a randomized controlled trial. Acta Obstet Gynecol Scand 2015;94(1):35–42.
524. Petrovic D, Perovic M, Lazovic B, et al. Association between walking, dysphoric mood and anxiety in late pregnancy: A cross-sectional study. Psychiatry Res 2016;246:360–3.
525. Phelan  S, Phipps  MG, Abrams  B, Darroch  F, Schaffner  A, Wing  RR.  Randomized trial of a behavioral intervention to prevent excessive gestational weight gain: the Fit for Delivery Study.   Am J Clin Nutr. 2011;93(4):772-779. doi:10.3945/ajcn.110.005306PubMedGoogle ScholarCrossref
526. Phelan  S, Wing  RR, Brannen  A,  et al.  Randomized controlled clinical trial of behavioral lifestyle intervention with partial meal replacement to reduce excessive gestational weight gain.   Am J Clin Nutr. 2018;107(2):183-194. doi:10.1093/ajcn/nqx043PubMedGoogle ScholarCrossref
527. Phelan S, Phipps MG, Abrams B et al. Does behavioural intervention in pregnancy reduce postpartum weight retention? Twelve-month outcomes of the fit for delivery randomized trial. Am J Clin Nutr 2014; 99: 302–311.
528. Phelan S, Phipps MG, Abrams B, Darroch F, Schaffner A, Wing RR. Factors associated with success in the “fit for delivery” intervention to reduce excessive gestational weight gain. Obesity 2011;19(Suppl 1):S95.
529. Piirainen T, Isolauri E, Lagstrom H, Laitinen K. Impact of dietary counselling on nutrient intake during pregnancy: a prospective cohort study. British Journal of Nutrition 2006; 96(6):1095–104
530. Pinzon DC, Zamora K, Martinez JH, Florez-Lopez ME, de Plata AC, Mosquera M, et al. Type of delivery and gestational age is not affected by pregnant Latin-American women engaging in vigorous exercise: a secondary analysis of data from a controlled randomized trial. Revista de salud publica (Bogota, Colombia). 2012;14(5):731–43.
531. Podeˇbradská R, Šarmírová M, Janura M, Elfmark M, Procházka M. The effect of physiotherapy intervention on the load of the foot and low back pain in pregnancy. Ceska Gynekol. 2019;84(6):450–457.
532. Piravej K, Saksirinukul R. Survey of patterns, attitudes, and the general effects of exercise during pregnancy in 203 Thai pregnant women at King Chulalongkorn Memorial Hospital. J Med Assoc Thai 2001;84(Suppl 1):S276–82.
533. Pollak KI, Alexander SC, Bennett G, Lyna P, Coffman CJ, Bilheimer A, Farrell D, Bodner ME, Swamy GK, Østbye T. Weight-related SMS texts promoting appropriate pregnancy weight gain: a pilot study. Patient Educ Couns. 2014;97(2):256–60. doi:10.1016/j.pec.2014.07.030.
534. Polley  BA, Wing  RR, Sims  CJ.  Randomized controlled trial to prevent excessive weight gain in pregnant women.   Int J Obes Relat Metab Disord. 2002;26(11):1494-1502. doi:10.1038/sj.ijo.0802130PubMedGoogle ScholarCrossref
535. Polley BA, Wing RR, Sims CJ. Randomized controlled trial to prevent excessive weight gain in pregnant women. Int J Obes 2002;26:1494–502.
536. Pomerance JJ, Gluck L, Lynch VA. Physical fitness in pregnancy: its effect on pregnancy outcome. American Journal of Obstetrics & Gynecology. 1974;44(3):383-7.
537. Poston L. Improving pregnancy outcome in obese women: a feasibility study. Current Controlled Trials (http://controlled-trials.com/) (accessed 31 October 2010).
538. Poston L., Bell R., Croker H., Flynn A., Godfrey K., Goff L., Hayes L., Khazaezadeh N., Nelson S.M., Oteng-Ntim E., et al. Effect of a behavioural intervention in obese pregnant women (the UPBEAT study): A multicentre, randomised controlled trial. Lancet Diabetes Endocrinol. 2015;3:767–777. doi: 10.1016/S2213-8587(15)00227-2.
539. Poston, L., Briley, A. L., Barr, S., Bell, R., Croker, H., Coxon, K., Essex, H. N., Hunt, C., Hayes, L., Howard, L. M., Khazaezadeh, N., Kinnunen, T., Nelson, S. M., Oteng-Ntim, E., Robson, S. C., Sattar, N., Seed, P. T., Wardle, J., Sanders, T. A. B., & Sandall, J. (2013). Developing a complex intervention for diet and activity behaviour change in obese pregnant women (the UPBEAT trial): assessment of behavioural change and process evaluation in a pilot randomised controlled trial. BMC Pregnancy and Childbirth, 13, 148.
540. Prabhu N. Effect of a exercise by pregnant women and birth weight: a randomized controlled trial. Journal of Evolution of Medical and Dental Sciences 2015;04:1509–16.
541. Prevedel, T I. Calderon, M. DeConti, E. Consonni, M. Rudge Maternal and perinatal effects of hydrotherapy in pregnancy Revista Brasil Ginecol Obstet, 25 (2003), pp. 53-59
542. Price B, Amini B, Kappeler K. Exercise in pregnancy: Effect on fitness and obstetric outcomes - a randomized trial. Medicine & Science in Sports & Exercise 2012;44(12):2263–9.
543. Puhkala J, Raitanen J, Kolu P, Tuominen P, Husu P, Luoto R. Metabolic syndrome in Finnish women 7 years after a gestational diabetes prevention trial. BMJ Open. 2017;7(3):e014565.
544. Putnam KF, Mueller LA, Magann EF, et al. Evaluating effects of self-reported domestic physical activity on pregnancy and neonatal outcomes in "stay at home" military wives. Mil Med. 2013;178(8):893-8.
545. Quinlivan J, Lam L, Fisher J. A randomised trial of a four-step multidisciplinary approach to the antenatal care of obese pregnant women. Aust N Z J Obstet Gynaecol. 2011;51:141–6.
546. Quinlivan J. A randomised trial of a multidisciplinary teamcare approach involving obstetric, dietary and clinical psychological input in obese pregnant women to reduce the incidence of gestational diabetes. https:// www.anzctr.org.au/Trial/Registration/TrialReview.aspx?id= 821 (accessed 2 November 2015)
547. Rae A, Bond D, Evans S, North F, Roberman B, Walters B. A randomised controlled trial of dietary energy restriction in the management of obese women with gestational diabetes. Australian & New Zealand Journal of Obstetrics & Gynaecology 2000;40 (4):416–22.
548. Rakhshani A, Maharana S, Raghuram N, Nagendra HR, & Venkatram P (2010). Effects of integrated yoga on quality of life and interpersonal relationship of pregnant women. Quality of Life Research, 19, 1447–1455. doi:10.1007/s11136-010-9709-2 [PubMed: 20632115]
549. Rakhshani A, Nagarathna R, Mhaskar R, et al. The effects of yoga in prevention of pregnancy complications in high-risk pregnancies: a randomized controlled trial. Prev Med 2012;55:333–40
550. Ramirez-Velez R, Aguilar de Plata AC, Escudero MM, Echeverry I, Ortega JG, Salazar B, et al. Influence of regular aerobic exercise on endothelium-dependent vasodilation and cardiorespiratory fitness in pregnant women. Journal of Obstetrics and Gynaecology Research 2011;37(11):1601–8.
551. Ramírez-Vélez R, Aguilar de Plata AC, Mosquera-Escudero M, BS, et al. The effect of aerobic exercise on oxygen consumption in healthy first pregnancy females: a randomized clinical trial. Rev Colomb Obstet Ginecol 2011;62:15–23.
552. Ramirez-Velez R. Aerobic exercise in pregnant Latina women: Effect on metabolic and body composition outcomes. A randomized clinical trial. FASEB Journal 2014;28(1 Suppl 1):886.6.
553. Ramirez-Velez R. Aerobic exercise training during pregnancy increases antioxidant status in nulliparous women: secondary analysis of a controlled clinical trial. Endocrinol Nutr 2013;60(5):279–81.
554. Ramirez-Velez R. Combined aerobic and resistance exercise on metabolic and body composition outcomes in primigravid Latina women. Obesity Reviews 2014;15(Suppl2):201–2.
555. Rankin J (2002) Effects of antenatal exercise on psychological well-being.pregnancy and birth outcome.  Whurr Publishers Ltd.
556. Rauh K, Gabriel E, Kerschbaum E, Schuster T, Kries R, AmannGassner U, et al. Safety and efficacy of a lifestyle intervention for pregnant women to prevent excessive maternal weight gain: A cluster-randomized controlled trial. BMC Pregnancy Childbirth 2013;13:151.
557. Rauh K, Gunther J, Kunath J, Stecher L, Hauner H. Lifestyle intervention to prevent excessive maternal weight gain: mother and infant follow-up at 12 months postpartum. BMC Pregnancy Childbirth 2015;15:265.
558. Reader D, Splett P, Gunderson EP. Impact of gestational diabetes mellitus nutrition practice guidelines implemented by registered dietitians on pregnancy outcomes. J Am Diet Assoc. 2006;106(9):1426–1433. PMID: 16963348. doi: 10.1016/j.jada.2006.06.009.
559. Redman  LM, Gilmore  LA, Breaux  J,  et al.  Effectiveness of SmartMoms, a novel eHealth intervention for management of gestational weight gain: randomized controlled pilot trial.   JMIR Mhealth Uhealth. 2017;5(9):e133. doi:10.2196/mhealth.8228PubMedGoogle Scholar
560. Reilly ET, Freeman RM, Waterfield MR, Waterfield AE, Steggles P, Pedlar F. Prevention of postpartum stress incontinence in primigravidae with increased bladder neck mobility: a randomised controlled trial of antenatal pelvic floor exercises. Bjog. 2002;109(1):68-76.
561. Reis PJ, & Alligood MR (2014). Prenatal yoga in late pregnancy and optimism, power, and well-being. Nursing Science Quarterly, 27(1), 30–36. doi:10.1177/0894318413509706 [PubMed: 24403034]
562. Reis YA, A. Akay, B. Aktan, S. Tetik, F.B. Flratllgil, F. Kaylkçloǧlu, The effect of clinical pilates exercises and prenatal education on maternal and fetal health, Z. Geburtshilfe Neonatol. 227 (5) (2023) 354–363.
563. Renault  KM, Norgaard  K, Nilas  L,  et al.  The Treatment of Obese Pregnant women (TOP) study: a randomized controlled trial of the effect of physical activity intervention assessed by pedometer with or without dietary intervention in obese pregnant women.   Am J Obstet Gynecol. 2013;210(2):134.e1-134.e9. doi:10.1016/j.ajog.2013.09.029PubMedGoogle ScholarCrossref
564. Renault KM, Nørgaard K, Nilas L et al. The Treatment of Obese Pregnant Women (TOP) study. A randomized controlled trial of the effect of physical activity intervention assessed by pedometer with or without dietary intervention in obese pregnant women. Am J Obstet Gynecol 2014; 210: 134.e1–134.e9. doi:10.1016/j.ajog.2013.09.029
565. Rhodes E., Pawlak D., Takoudes T., Ebbeling C., Feldman H., Lovesky M., Cooke E.A., Leidig M.M., Ludwig D.S. Effects of a low-glycemic load diet in overweight and obese pregnant women: A pilot randomized controlled trial. Am. J. Clin. Nutr. 2010;92:1306–1315. doi: 10.3945/ajcn.2010.30130
566. Rice PL, Fort IL. The relationship of maternal exercise on labor, delivery and health of the newborn. J Sports Med Phys Fitness. 1991;31(1):95-9.
567. Ricketts SA, Murray EK, Schwalberg R. Reducing low birthweight by resolving risks: results from Colorado’s prenatal plus program. Am J Public Health. 2005;95(11):1952–1957. PMID: 16195530. doi: 10.2105/ AJPH.2004.047068..
568. Robledo-Colonia AF, Sandoval-Restrepo N, Mosquera-Valderrama YF, Escobar-Hurtado C, Ramirez-Velez R. Aerobic exercise training during pregnancy reduces de- pressive symptoms in nulliparous women: a randomised trial. J Physiother 2012; 58(1):9–15.
569. Rodríguez YC, Puente MP, Abad MDM, RBC, et al. Can moderate physical exercise during pregnancy act as a factor in preventing Gestational Diabetes? Rev Int Cienc Deporte 2012;8:3–19.
570. Rodríguez-Blanque R., Carlos Sánchez-García J., Manuel Sánchez-López A., Mur-Villar N., Fernández-Castillo R., José Aguilar-Cordero M. Influence of physical exercise during pregnancy on birthweight: A randomized clinical trial. Nutr. Hosp. 2017;34:834–840. doi: 10.20960/nh.1095.
571. Rodríguez-Blanque R., Garcia J.C.S., López A.M.S., Aguilar-Cordero M.J. Physical activity during pregnancy and its influence on delivery time: A randomized clinical trial. PeerJ. 2019;7:e6370. doi: 10.7717/peerj.6370.
572. Rodríguez-Blanque R., Garcia J.C.S., López A.M.S., Expósito-Ruiz M., Aguilar-Cordero M.J. Randomized Clinical Trial of an Aquatic Physical Exercise Program during Pregnancy. J. Obstet. Gynecol. Neonatal Nurs. 2019;48:321–331. doi: 10.1016/j.jogn.2019.02.003.
573. Rodríguez-Blanque R., Sánchez-García J.C., Sánchez-López A.M., Mur-Villar N., Fernández-Castillo R., Cordero M.J.A. Influencia del ejercicio físico durante el embarazo sobre el peso del recién nacido: Un ensayo clínico aleatorizado. Nutrición Hospitalaria. 2017;34:834–840. doi: 10.20960/nh.1095.
574. Rodríguez-Blanque R, Aguilar-Cordero MJ, Marín-Jiménez AE, Núñez-Negrillo AM, Sánchez-López AM, Sánchez-García JC. Influence of a water-based exercise program in the rate of spontaneous birth: a randomized clinical trial. Int J Environ Res Public Health. 2020;17(3):795. doi:10.3390/ijerph17030795
575. Rodríguez-Díaz, L., C. Ruiz-Frutos, J. M. Vázquez-Lara, J. Ramírez-Rodrigo, C. Villaverde-Gutiérrez, and G. TorresLuque. 2017. “Effectiveness of a Physical Activity Programme Based on the Pilates Method in Pregnancy and Labour.” Enfermeria Clinica 27 (5): 271–7. https://doi.org/10.1016/j.enfcli.2017.05.008
576. Rong, L., Wang, R., Ouyang, Y.Q., Redding, S.R., 2021. Efficacy of yoga on physiological and psychological discomforts and delivery outcomes in Chinese primiparas. Complement. Ther. Clin. Pract. 44, 101434.
577. Ronnberg  AK, Ostlund  I, Fadl  H, Gottvall  T, Nilsson  K.  Intervention during pregnancy to reduce excessive gestational weight gain—a randomised controlled trial.   BJOG. 2015;122(4):537-544. doi:10.1111/1471-0528.13131PubMedGoogle ScholarCrossref
578. Ronnberg A, Hanson U, Ostlund I, Nilsson K. Effects on postpartum weight retention after antenatal lifestyle intervention—a secondary analysis of a randomized controlled trial. Acta Obstet Gynecol Scand. 2016;95(9):999- 1007.
579. Rönö  K, Grotenfelt  NE, Klemetti  MM,  et al.  Effect of a lifestyle intervention during pregnancy—findings from the Finnish gestational diabetes prevention trial (RADIEL).   J Perinatol. 2018;38(9):1157-1164. doi:10.1038/s41372-018-0178-8PubMedGoogle ScholarCrossref
580. Rönö  K, Stach-Lempinen  B, Eriksson  JG,  et al.  Prevention of gestational diabetes with a prepregnancy lifestyle intervention—findings from a randomized controlled trial.   Int J Womens Health. 2018;10:493-501. doi:10.2147/IJWH.S162061PubMedGoogle ScholarCrossref
581. Rose NC, Haddow JE, Palomaki GE, Knight GJ. Self-rated physical activity level during the second trimester and pregnancy outcome. Obstet Gynecol. 1991;78(6):1078–80.
582. Ross SM, Nel E, Naeye RL. Differing effects of low and high bulk maternal dietary supplements during pregnancy. Early Hum Dev 1985;10:295–302.
583. Ruchat S M, Davenport M H, Giroux I et al. Nutrition and exercise reduce excessive weight gain in normal-weight pregnant women. Med Sci Sports Exerc. 2012;44(08):1419–1426. [PubMed] [Google Scholar]
584. Ruchat SM, Davenport MH, Giroux I, et al. Effect of exercise intensity and duration on capillary glucose responses in pregnant women at low and high risk for gestational diabetes. Diabetes Metab Res Rev 2012;28:669–78.
585. Ruchat SM, Davenport MH, Hillier I, Batada A, Sopper J, Hammond M, et al. Walking program of low or vigorous intensity during pregnancy confers an aerobic benefit. Int J Sports Med. 2012b;33(8):661–6.
586. Ruiz J R, Perales M, Pelaez M, Lopez C, Lucia A, Barakat R. Supervised exercise-based intervention to prevent excessive gestational weight gain: a randomized controlled trial. Mayo Clin Proc. 2013;88(12):1388– 1397. [PubMed] [Google Scholar]
587. Rush D, Stein Z, Susser M. A randomized controlled trial of prenatal nutritional supplementation in New York City. Pediatrics 1980;65:683–97.
588. Saftlas AF, Logsden-Sackett N, Wang W, et al. Work, leisure-time physical activity, and risk of preeclampsia and gestational hypertension. Am J Epidemiol 2004;160:758–65.
589. Sagedal  LR, Øverby  NC, Bere  E,  et al.  Lifestyle intervention to limit gestational weight gain: the Norwegian Fit for Delivery randomised controlled trial.   BJOG. 2017;124(1):97-109. doi:10.1111/1471-0528.13862PubMedGoogle ScholarCrossref
590. Sagedal LR, Vistad I, Øverby NC, et al. The effect of a prenatal lifestyle intervention on glucose metabolism: results of the Norwegian fit for delivery randomized controlled trial. BMC Pregnancy Childbirth 2017;17:167.
591. Salvesen KÅ, Stafne SN, Eggebø TM, Mørkved S. Does regular exercise in pregnancy influence duration of labor? A secondary analysis of a randomized controlled trial. Acta Obstet Gynecol Scand 2014; 93(1): 73-9.
592. Salvig J, Olsen S, Secher N. Effects of fish oilsupplementation in late pregnancy on blood pressure: arandomised controlled trial. BJOG. 1996;103:529–33
593. Sánchez-García J.C., Aguilar-Cordero M.J., Menor-Rodríguez M.J., Paucar-Sánchez A., Rodríguez Blanque R. Influencia del ejercicio físico en la evolución del peso gestacional y posparto. Ensayo clínico aleatorizado. Nutr. Hosp. 2019;36:1–8. doi: 10.20960/nh.02456.
594. Santaella MP. The role of a supervised physical exercise program as an alternative on the control of maternal estational weight gain. ClinicalTrials.gov (accessed 21 May20 13). NCT01790347 2013.
595. Santamaria A., Di Benedetto A., Petrella E., Pintaudi B., Corrado F., D’Anna R., Neri I., Facchinetti F. Myo-inositol may prevent gestational diabetes onset in overweight women: A randomized, controlled trial. J. Matern. Fetal Neonatal Med. 2016;29:3234–3237. doi: 10.3109/14767058.2015.1121478.
596. Santos IA, Stein R, Fuchs SC et al. Aerobic exercise and sub-maximal functional capacity in overweight pregnant women: a randomized trial. Obstet Gynecol 2005;106(2): 243–249.43.
597. Satyapriya M, Nagarathna R, Padmalatha V, & Nagendra HR (2013). Effect of integrated yoga on anxiety, depression and well-being in normal pregnancy. Complementary Therapies in Clinical Practice, 19, 230–236. doi:10.1016/j.ctcp.2013.06.003 [PubMed: 24199979]
598. Sayed Mohsen M, Hajar S, Fatemeh M, Maryam Z. Effects of isometric exercise on the alleviation of lumbar and pelvic pain in pregnant women resident in Isfahan, Iran. J Rehabil Res Dev. 2012;7(5). doi:10.22122/jrrs.v7i5.365
599. Schneeberger C, Flynn A, Barr S, Seed PT, Inskip HM, Poston L. Maternal diet patterns and glycaemic load in obese pregnant women taking part in a pilot trial of a lifestyle intervention (the upbeat trial). Diabetes 2014; Vol.63.
600. Seabrook JA, Smith A, Clark AF, Gilliland JA. Geospatial analyses of adverse birth outcomes in Southwestern Ontario: examining the impact of environmental factors. Environ Res. 2019 May;172:18–26. PMID: 30769185. doi: 10.1016/j.envres.2018.12.068.
601. Sedaghati P, Ziaee V, Ardjmand A. The effect of an ergometric training program on pregnants weight gain and low back pain. Gazz Med Ital—Arch Sci Med 2007; 166(6):209–213.
602. Senek, M. (2018). An mHealth walking intervention for pregnant women with obesity. Doctoral, Sheffield Hallam University.
603. Seneviratne S, Jiang Y, Derraik J, McCowan L, Parry G, Biggs J, et al. Effects of antenatal exercise in overweight and obese pregnant women on maternal and perinatal outcomes: a randomised controlled trial. BJOG Int J Obstet Gynaecol. 2016;123(4):588–97.
604. Sewell DA, Hammersley VS, Robertson A, et al. A pilot randomised controlled trial investigating a Mediterranean diet intervention in pregnant women for the primary prevention of allergic diseases in infants. J Hum Nutr Diet. 2017;30(5):604-614. doi:10.1111/jhn.12469
605. Shen GX (2006) Community-based exercise and dietary intervention during pregnancy: A pilot study. Canadian Journal of diabetes 30: 169–175.
606. Shim MJ, Lee YS, Oh HE, Kim JS. Effects of a back-pain-reducing program during pregnancy for Korean women: a non-equivalent control-group pretest-posttest study. Int J Nurs Stud. 2007;44(1):19–28. doi:10.1016/j. Ijnurstu.2005.11.016.
607. Shirazian T, Monteith S, Friedman F, et al. Lifestyle Modification Program Decreases Pregnancy Weight Gain in Obese Women. Am J Perinatol 2010;27:411–4.
608. Shub A. Diet and exercise in pregnancy. Australian Clinical Trials Registry (www.actr.org.au/) [accessed 21 June 2007].
609. Shyam S, Arshad F, Abdul Ghani R, Wahab NA, Safii NS, Nisak MYB, et al. Low glycaemic index diets improve glucose tolerance and body weight in women with previous history of gestational diabetes: a six months randomized trial. Nutr J 2013;12:68-. Asbee SM, Jenkins TR, Butler JR, White J, Elliot M, Rutledge A. Preventing excessive weight gain during pregnancy through dietary and lifestyle counseling: a randomized controlled trial. Obstet Gynecol 2009;113(2 Pt 1):305–12.
610. Sillero-Quintana M., Conde-Pascual E., Gomez-Carmona P.M., Fernandez-Cuevas I., García-Pastor T. Effect of yoga and swimming on body temperature of pregnant women. Thermol. Int. 2012;22:108.
611. Simmons  D, Devlieger  R, van Assche  A,  et al.  Effect of physical activity and/or healthy eating on GDM risk: the DALI lifestyle study.   J Clin Endocrinol Metab. 2017;102(3):903-913. doi:10.1210/jc.2016-3455PubMedGoogle Scholar
612. Simmons D, Jelsma JG, Galjaard S, Devlieger R, van Assche A, Jans G, Corcoy R, Adelantado JM, Dunne F, Desoye G, Harreiter J. Results from a European multicenter randomized trial of physical activity and/or healthy  eating to reduce the risk of gestational diabetes mellitus: the DALI lifestyle pilot. Diabetes Care. 2015;38 (9):1650–56. doi:10.2337/dc15-0360.
613. Sklempe Kokic I, Ivanisevic M, Biolo G, Simunic B, Kokic T, Pisot R. Combination of a structured aerobic and resistance exercise improves glycaemic control in pregnant women diagnosed with gestational diabetes mellitus. A randomised controlled trial. Women and birth. 2017; https://doi. org/10.1016/j.wombi.2017.10.004.
614. Skouteris H., McPhie S., Hill B., McCabe M., Milgrom J., Kent B., Bruce L., Herring S., Gale J., Mihalopoulos C., et al. Health coaching to prevent excessive gestational weight gain: A randomized-controlled trial. Br. J. Health Psychol. 2016;21:31–51. doi: 10.1111/bjhp.12154.
615. Smith  K, Lanningham-Foster  L, Welch  A, Campbell  C.  Web-based behavioral intervention increases maternal exercise but does not prevent excessive gestational weight gain in previously sedentary women.   J Phys Act Health. 2016;13(6):587-593. doi:10.1123/jpah.2015-0219PubMedGoogle ScholarCrossref
616. Smith KM. The Blossom Project Online: use of a behaviorally based website to promote physical activity and prevent excessive gestational weight gain in previously sedentary pregnant women. Graduate Theses and Dissertations 2014: 14051
617. Smith S.A., Michel Y. A Pilot Study on the Effects of Aquatic Exercises on Discomforts of Pregnancy. J. Obstet. Gynecol. Neonatal Nurs. 2006;35:315–323. doi: 10.1111/j.1552-6909.2006.00045.x. -
618. Smuts CM, Borod E, Peeples JM, Carlson SE. High-DHA eggs: feasibility as a means to enhance circulating DHA in mother and infant. Lipids 2003;38:407–14.
619. Smuts CM, Huang M, Mundy D, Plasse T, Major S,Carlson SE. A randomised trial of docosahexaenoic acidsupplementation during the third trimester of pregnancy.Obstet Gynecol. 2003;101:469–79
620. Soltani H, Duxbury A, Arden M, Dearden A, Furness P, Garland C. Maternal obesity management using mobile technology: a feasibility study to evaluate a text messaging based complex intervention during pregnancy. J Obes 2015;2015:814830 [FREE Full text] [doi: 10.1155/2015/814830] [Medline: 25960889]
621. Sonmezer E, Özköslü MA, Yosmaoğlu HB. The effects of clinical pilates exercises on functional disability, pain, quality of life and lumbopelvic stabilization in pregnant women with low back pain: A randomized controlled study. J Back Musculoskelet Rehabil. 2021;34(1):69–76.
622. Songøygard, K.M., Stafne, S.N., Evensen, K.A., Salvesen, K., Vik, T., Mørkved, S., 2012. Does exercise during pregnancy prevent postnatal depression? A randomized controlled trial. Acta Obstet. Gynecol. Scand. 91, 62–67.
623. Soultanakis HN, Artal R, Wiswell RA. Prolonged exercise in pregnancy: glucose homeostasis, ventilatory and cardiovascular responses. Semin Perinatol 1996;20:315–27.
624. Stafne SN, Salvesen KA, Romundstad PR, Eggebo TM, Carlsen SM, Morkved S. Regular exercise during pregnancy to prevent gestational diabetes: a randomized controlled trial. Obstet Gynecol. 2012;119(1):29-36.
625. Stafne SN, Salvesen KA, Romundstad PR, Stuge B, Morkved S. Does regular exercise during pregnancy influence lumbopelvic pain? A randomized controlled trial. Acta Obstet Gynecol Scand 2012;91(5):552–9.
626. Stafne SN, Salvesen KA, Romundstad PR, Torjusen IH, Morkved S. Does regular exercise including pelvic floor muscle training prevent urinary and anal incontinence during pregnancy? A randomised controlled trial. BJOG 2012; 119(10): 1270-80.
627. Stendell-Hollis NR, Thompson PA, West JL, Wertheim BC, Thomson CA. A comparison of Mediterranean-style and MyPyramid diets on weight loss and inflammatory biomarkers in postpartum breastfeeding women. J Womens Health 2013;22(1):48–57.
628. Sternfeld B, Quesenberry CP Jr, Eskenazi B, Newman LA. Exercise during pregnancy and pregnancy outcome. Med Sci Sports Exerc. 1995;27(5):634– 40.
629. Storck Lindholm E, Norman M, Kilander CP, Altman D. Weight control program for obese pregnant women. Acta Obstet Gynecol Scand. 2010;89:840–3.
630. Strøm M, Mortensen EL, Halldorson TI, et al. Leisure-time physical activity in pregnancy and risk of postpartum depression: a prospective study in a large national birth cohort. J Clin Psychiatry 2009;70:1707–14
631. Sui Z, Yelland LN, Turnbull D, Dodd JM. Walking to limit gestational weight gain and keep fit during pregnancy - findings from the walk randomised trial. Journal of Paediatrics and Child Health 2013;49 Suppl 2:120.
632. Sun Y, Zhao H. The effectiveness of lifestyle intervention in early pregnancy to prevent gestational diabetes mellitus in Chinese overweight and obese women: a quasiexperimental study. Appl Nurs Res 2016;30:125–30.
633. Suputtitada, A.; Wacharapreechanont, T.; Chaisayan, P. Effect of the “sitting pelvic tilt exercise” during the third trimester in primigravidas on back pain. J. Med. Assoc. Thail. 2002, 85 (Suppl. 1), S170–S179.
634. Surita F. Physical exercise influence among overweightand obese pregnant women. ClinicalTrials.gov (http://clinicaltrials.gov/) [accessed 21 May 2013] 2010.
635. Symons Downs, D., Rivera, D. E., Savage, J. S., Stetter, C., Kunselman, A., Pauley, A. M., et al. (2019). Optimizing an individually-tailored, adaptive intervention to manage weight in overweight and obese pregnant women. Annals of Behavioral Medicine, 53(S1), S149–S150.
636. Syngelaki A., Nicolaides K., Balani J., Hyer S., Akolekar R., Kotecha R., Pastides A., Shehata H. Metformin versus Placebo in Obese Pregnant Women without Diabetes Mellitus. N. Engl. J. Med. 2016;374:434–443. doi: 10.1056/NEJMoa1509819.
637. Szmeja M A, Cramp C, Grivell R M, Deussen A R, Yelland L N, Dodd J M. Use of a DVD to provide dietary and lifestyle information to pregnant women who are overweight or obese: a nested randomised trial. BMC Pregnancy Childbirth. 2014;14:409. [PMC free article] [PubMed] [Google Scholar]
638. Szmeja MA, Grivell RM, Deussen AR, Dodd JM. Evaluation of information provision to women who are overweight or obese during pregnancy. Journal of Paediatrics and Child Health 2011;47(Suppl 1):78.
639. Taniguchi C, Sato C. Home-based walking during pregnancy affects mood and birth outcomes among sedentary women: A randomized controlled trial. International Journal of Nursing Practice. 2016;22(5):420-6.
640. Tanvig M, Vinter C A, Jørgensen J S et al. Effects of lifestyle intervention in pregnancy and anthropometrics at birth on offspring metabolic profile at 2.8 years: results from the Lifestyle in Pregnancy and Offspring (LiPO) study. J Clin Endocrinol Metab. 2015;100(01):175–183. [PubMed] [Google Scholar]
641. Tanvig M. Lifestyle in pregnancy and offspring - comparison between children born to obese women and children born to normal weight women (LiPO). ClinicalTrials.gov (http:// clinicaltrials.gov/) [accessed 6 February 2014] 2013.
642. Tanvig M. Lifestyle in pregnancy and offspring (LiPO). ClinicalTrials.gov(http://clinicaltrials.gov/) [accessed 6 February 2014] 2013.
643. Tanvig M. Offspring body size and metabolic profile - Effects of lifestyle intervention in obese pregnant women. Danish Medical Journal 2014;61(7):B4893.
644. Teede HJ, Harrison CL, Gibson-Helm M, Lombard CB. Improving physical activity in high-risk pregnancies: A randomized controlled trial. Endocrine Reviews 2011;32(3Meeting Abstracts):P1–467.
645. Teychenne, M., Abbott, G., Stephens, L.D., Opie, R.S., Olander, E.K., Brennan, L., van der Pligt, P., Apostolopoulos, M., Ball, K., 2021. Mums on the move: a pilot randomised controlled trial of a home-based physical activity intervention for mothers at risk of postnatal depression. Midwifery 93, 102898.
646. Thomson  JL, Tussing-Humphreys  LM, Goodman  MH, Olender  SE.  Gestational weight gain: results from the Delta Healthy Sprouts comparative impact trial.   J Pregnancy. 2016;2016:5703607. doi:10.1155/2016/5703607PubMedGoogle Scholar
647. Thornton YS, Smarkola C, Kopacz SM, Ishoof SB. Perinatal outcomes in nutritionally monitored obese pregnant women: a randomized clinical trial. J Natl Med Assoc 2009;101(6): 569–577.
648. To WWK, Wong MWN. Bone mineral density changes during pregnancy in actively exercising women as measured by quantitative ultrasound. Archives of Gynecology and Obstetrics. 2012;286(2):357-63.
649. Tomić V, Sporiš G, Tomić J, et al. The effect of maternal exercise during pregnancy on abnormal fetal growth. Croat Med J 2013;54:362–8.
650. Tricco AC, Lillie E, Zarin W, O’Brien KK, Colquhoun H, Levac D, et al. PRISMA extension for scoping reviews (PRISMA-ScR): checklist and explanation. Ann Intern Med. 2018;169(7):467–73.
651. Uebelacker, L.A., Battle, C.L., Sutton, K.A., Magee, S.R., Miller, I.W., 2016. A pilot randomized controlled trial comparing prenatal yoga to perinatal health education for antenatal depression. Arch. Womens Ment. Health 19, 543–547.
652. Umpierrez G. Lifestyle intervention to limit excessive weight gain during pregnancy in minority women. ClinicalTrials.gov (accessed 21 May 2013). NCT01084941 2010.
653. Urer E, Ozen N, Terzioglu F. Effect of pilates performed during pregnancy on postpartum stress urinary incontinence in Primipara women. J Bodyw Mov Ther. 2023;35:228–32
654. Ussher M, Lewis S, Aveyard P, et al. Physical activity for smoking cessation in pregnancy: randomised controlled trial. BMJ 2015;350:h2145.
655. Ussher M, Lewis S, Aveyard P, et al. The London Exercise And Pregnant smokers (LEAP) trial: a randomised controlled trial of physical activity for smoking cessation in pregnancy with an economic evaluation. Health Technol Assess 2015;19:1–135.
656. Ussher M, Lewis S, Aveyard P, et al. Physical activity for smoking cessation in pregnancy: randomised controlled trial. BMJ 2015;350:h2145
657. Vahamiko S, Isolauri E, Laitinen K. Weight status and dietary intake determine serum leptin concentrations in pregnant and lactating women and their infants. British Journal of Nutrition 2013;110(6):1098–106
658. Vallim A.L., Osis M.J., Cecatti J.G., Baciuk E.P., Silveira C., Cavalcante S.R. Water exercises and quality of life during pregnancy. Reprod. Health. 2011;8:14–17. doi: 10.1186/1742-4755-8-14. -
659. Van Buul BJA, Steegers EAP, Van Der Maten GD, Delemarre FMC, Jongsma HW, Oosterbaan HP, De Jong PA. Dietary sodium restriction does not prevent gestational hypertension: a Dutch two-center randomized trial. Hypertens Pregnancy 1997;16:335–46
660. Van Horn  L, Peaceman  A, Kwasny  M,  et al.  Dietary approaches to stop hypertension diet and activity to limit gestational weight: maternal offspring metabolics family intervention trial, a technology enhanced randomized trial.   Am J Prev Med. 2018;55(5):603-614. doi:10.1016/j.amepre.2018.06.015PubMedGoogle ScholarCrossref
661. Van Poppel M, Oostdam N, Wouters M, Eekhoff M, Van Mechelen W. FitFor2: Effects of an exercise training program on the incidence of gestational diabetes. Journal of Science and Medicine in Sport 2012;15 Suppl 1:S342–S343.
662. van Zutphen M, Milder IE, Bemelmans WJ. Usage of an online healthy lifestyle program by pregnant women attending midwifery practices in Amsterdam. Prev Med 2008; 46: 552–557.
663. Vargas-Terrones, M., Barakat, R., Santacruz, B., Fernandez-Buhigas, I., Mottola, M.F., 2019. Physical exercise programme during pregnancy decreases perinatal depression risk: a randomised controlled trial. Br. J. Sports Med. 53, 348–353.
664. Vargas-Terrones, M., Nagpal, T.S., Perales, M., Prapavessis, H., Mottola, M.F., Barakat, R., 2021. Physical activity and prenatal depression: going beyond statistical significance by assessing the impact of reliable and clinical significant change. Psychol. Med. 51, 688–693.
665. Vázquez-Lara J.M., Ruiz-Frutos C., Rodríguez-Díaz L., Ramírez-Rodrigo J., Villaverde-Gutiérrez C., Torres-Luque G. Effect of a physical activity programme in the aquatic environment on haemodynamic constants in pregnant women. Enfermería Clínica. 2018;28:316–325. doi: 10.1016/j.enfcli.2017.07.009
666. Vesco K, Leo M, Gillman M, King J, McEvoy C, Karanjaa N, et al. Impact of a weight management intervention on pregnancy outcomes among obese women: The Healthy Moms Trial. American Journal of Obstetrics and Gynecology 2013;208(1 Suppl 1):S352.
667. Vesco K, Leo M, Karanja N, et al. One-year postpartum outcomes following a weight management intervention in pregnant women with obesity. Obesity. 2016;24(10):2042-2049.
668. Vesco K., Karanja N., King J., Gillman M., Leo M., Perrin N., McEvoy C.T., Eckhardt C.L., Smith K.S., Stevens C.L. Efficacy of a group-based dietary intervention for limiting gestational weight gain among obese women: A randomized trial. Obesity. 2014;22:1989–1996. doi: 10.1002/oby.20831
669. Vesco KK, Karanja N, King JC, Gillman MW, Perrin N, McEvoy C, et al. Healthy Moms, a randomized trial to promote and evaluate weight maintenance among obese pregnant women: study design and rationale. Contemporary Clinical Trials 2012;33(4):777–85.
670. Vinter A. Lifestyle and pregnancy: the clinical effect of lifestyle intervention during pregnancy in obese women. ClinicalTrials.gov(www.clinicaltrials.gov) [accessed 31 October 2010].
671. Vinter C, Jensen D, Ovesen P, Beck-Nielsen H, Lamont R, Jorgensen J. Postpartum weight retention and breastfeeding among obese women from the LiP (Lifestyle in Pregnancy) Study. Acta Obstetricia et Gynecologica Scandinavica 2012;91(Suppl 159):141–2.
672. Vinter C., Jensen D., Ovesen P., Beck-Nielsen H.J.O. Lifestyle and pregnancy (LIP) study: The clinical effect of lifestyle intervention during pregnancy in obese women. Diabetes. 2011;60:348–349. doi: 10.2337/dc11-1150.
673. Vinter CA, Jensen DM, Ovesen P, Beck-Nielsen H, Tanvig M, Lamont RF, et al. Postpartum weight retention and breastfeeding among obese women from the randomized controlled Lifestyle in Pregnancy (LiP) trial. Acta Obstetricia et Gynecologica Scandinavica 2014;93:794–801.
674. Vinter CA, Jorgensen JS, Ovesen P et al. Metabolic effects of lifestyle intervention in obese pregnant women. Results from the randomized controlled trial ‘Lifestyle in Pregnancy’ (LiP). Diabet Med 2014; 31: 1323– 1330. doi:10.1111/dme.12548
675. Vinter, C.A., Jensen, D.M., Ovesen, P., Beck-Nielsen, H., Jørgensen, J.S., 2011. The LiP (Lifestyle in Pregnancy) Study: a randomized controlled trial of lifestyle intervention in 360 obese pregnant women. Diabetes Care 34, 2502–2507.
676. Vitolo MR, Fraga Bueno MS, Mendes Gama C. Impact of a dietary counseling program on the gain weight speed of pregnant women attended in a primary care service. Revista Brasileira de Ginecologia e Obstetricia 2011;33(1):13–9.
677. Vitolo MR. Impact of a nutritional intervention program for weight control during pregnancy. ClinicalTrials.gov (www.clinicaltrials.gov) [accessed 31 October 2010].
678. Vollebregt KC, Wolf H, Boer K, van der Wal MF, Vrijkotte TGM, Bonsel GJ. Does physical activity in leisure time early in pregnancy reduce the incidence of preeclampsia or gestational hypertension? Acta Obstetricia Et Gynecologica Scandinavica. 2010;89(2):261-7.
679. Wallace AM, Boyer DB, Dan A, Holm K. Aerobic exercise, maternal self-esteem, and physical discomforts during pregnancy. J Nurse Midwifery. 1986;31(6):255-62.
680. Walsh J M, McAuliffe F M. Impact of maternal nutrition on pregnancy outcome--does it matter what pregnant women eat? Best Pract Res Clin Obstet Gynaecol. 2015;29(01):63–78. [PubMed] [Google Scholar]
681. Walsh J, Mahony R, Foley M, Mc Auliffe F. A randomised control trial of low glycaemic index carbohydrate diet versus no dietary intervention in the prevention of recurrence of macrosomia. BMC Pregnancy and Childbirth2010;10:16.
682. Walsh J, Mahony R, Foley M, McAuliffe F. ROLO study: a randomized control trial of low glycemic index diet to prevent macrosomia in euglycemic women. American Journal of Obstetrics and Gynecology 2012;206,Suppl 1):S4.
683. Walsh J, McGowan C, Byrne J, Foley M, Mahony R, McAuliffe F. The influence of a low glycaemic index dietary intervention on maternal glycaemic index, dietary intake and gestational weight gain. American Journal of Obstetrics and Gynecology 2013;208(1 Suppl):S33
684. Walsh JM, Mahony RM, Canty G, Foley ME, McAuliffe FM. Identification of those most likely to benefit from a low-glycaemic index dietary intervention in pregnancy. British Journal of Nutrition 2014;112:583–9.
685. Walsh JM, Mahony RM, Culliton M, Foley ME, McAuliffe FM. Impact of a low glycemic index diet in pregnancy on markers of maternal and fetal metabolism and inflammation. Reproductive Sciences 2014;21(11):1378–81
686. Walsh JM, McAuliffe FM. Impact of maternal nutrition on pregnancy outcome - Does it matter what pregnant women eat? Best Practice and Research. Clinical Obstetrics and Gynaecology 2015;29(1):63–78
687. Walsh JM, McGowan CA, Mahony R, Foley ME, McAuliffe FM. Low glycaemic index diet in pregnancy to prevent macrosomia (ROLO study): randomised control trial. BMJ 2012;345:e5605.
688. Wang C, Wei Y, Zhang X, Zhang Y, Xu Q, Su S, et al. Effect of regular exercise commenced in early pregnancy on the incidence of gestational diabetes mellitus in overweight and obese pregnant women: a randomized controlled trial. Diabetes Care. 2016;39(10):e163–4.
689. Wang C,Wei Y, Zhang X, et al. A randomized clinical trial of exercise during pregnancy to prevent gestational diabetes mellitus and improve pregnancy outcome in overweight and obese pregnant women. Am J Obstet Gynecol. 2017;216:340–351.
690. Wang S., Ma J., Yang H. Lifestyle intervention for gestational diabetes mellitus prevention: A cluster-randomized controlled study. Chronic Dis. Transl. Med. 2015;1:169–174. doi: 10.1016/j.cdtm.2015.09.001
691. Wang X, Li GY, Deng ML. Pelvic floor muscle training as a persistent nursing intervention: Effect on delivery outcome and pelvic floor myodynamia. International Journal of Nursing Sciences. 2014;1(1):48-52.
692. Weiderman WC, Marcuz L, Platt LD. Gestational diabetes: a triage model of care for rural perinatal providers. Am J Obstet Gynecol. 1996;174(6):1719–1724. PMID: 8678132. doi: 10.1016/S0002-9378(96) 70202-8. 30.
693. Weisman CS, Hillemeier MM, Downs DS, Feinberg ME, Chuang CH, et al. (2011) Improving Women’s Preconceptional Health: Long-Term Effects of the Strong Healthy Women Behaviour Change Intervention in the Central Pennsylvania Women’s Health Study. Women’s Health Issues. [Online]; 265– 271. A
694. Weissgerber TL, Davies GA, Roberts JM. Modification of angiogenic factors by regular and acute exercise during pregnancy. J Appl Physiol 2010;108:1217–23.
695. West C. Developing a support service for overweight women. Pract Midwife. 2010;13(10):19–21.
696. White E, Pivarnik J, Pfeiffer K. Resistance training during pregnancy and perinatal outcomes. Journal of Physical Activity and Health 2014;11:1141–8.
697. Wilkinson SA, McIntyre HD. Evaluation of the ’healthy start to pregnancy’ early antenatal health promotion workshop: a randomized controlled trial. BMC Pregnancy and Childbirth 2012;12:131.
698. Wilkinson SA, Pligt P, Gibbons KS, McIntyre HD. Trial for Reducing Weight Retention in New Mums: a randomised controlled trial evaluating a low intensity, postpartum weight management programme. J Hum Nutr Diet 2015;28(Suppl 1):15–28.
699. Willcox, J. C., Wilkinson, S. A., Lappas, M., Ball, K., Crawford, D., McCarthy, E., Fjeldsoe, B., Whittaker, R., Maddison, R., & Campbell, K. J. (2017). A mobile health intervention promoting healthy gestational weight gain for women entering pregnancy at a high body mass index: the txt4two pilot randomised controlled trial. BJOG: An International Journal of Obstetrics and Gynaecology, 124, 1718–1728
700. Wiltheiss GA, Lovelady CA, West DG, Brouwer RJ, Krause KM, Østbye T. Diet quality and weight change among overweight and obese postpartum women enrolled in a behavioral intervention program. J Acad Nutr Diet 2013;113(1): 54–62
701. Woldringh C, Wijngaart Mvd, Albers-Heitner P, Lycklama a N, A A, Lagro-Janssen T. Pelvic floor muscle training is not effective in women with UI in pregnancy: a randomised controlled trial. Int Urogynecol J Pelvic Floor Dysfunct. 2007;18(4):383-90.
702. Wolfe LA, Heenan AP, Bonen A. Aerobic conditioning effects on substrate responses during graded cycling in pregnancy. Can J Physiol Pharmacol 2003;81:696–703.
703. Wolff S, Legarth J, Vangsgaard K, Toubro S, Astrup A. A randomised trial of the effects of dieatry counseling on gestational weight gain and glucose metabolism inobese pregnant women. Int J Obesity. 2008;32:495–501
704. Ww T, Wong MW. Bone mineral density changes during pregnancy in actively exercising women as measured by quantitative ultrasound. Arch Gynecol Obstet 2012;286:357–63.
705. Xu G, Wu Y, Yang L, et al. Risk factors for early miscarriage among Chinese: a hospitalbased case-control study. Fertil Steril 2014;101:1663–70.
706. Xu MY, Guo YJ, Zhang LJ, Lu QB. Effect of individualized weight management intervention on excessive gestational weight gain and perinatal outcomes: a randomized controlled trial. PeerJ 2022;10:e13067
707. Yaman, O.B. ¨ Tulmaç, B. Canarslan, N. Hançerliogullari, ˘ Effect of pilates during pregnancy on delivery outcomes, J. Health Sci. Med. 3 (4) (2020) 442–447.
708. Yeo S, Davidge S, Ronis DL, Antonakos CL, Hayashi R, O'Leary S. A comparison of walking versus stretching exercises to reduce the incidence of preeclampsia: a ran-domized clinical trial. Hypertens Pregnancy 2008;27(2):113–30.
709. Yeo S. Adherence to walking or stretching, and risk of preeclampsia in sedentary pregnant women. Res Nurs Health 2009;32(4):379–90.
710. Yeo, N. M. Steele, M.-C. Chang, S. M. Leclaire, D. L. Ronis, and R. Hayashi, Effect of exercise on blood pressure in pregnant women with a high risk of gestational hypertensive disorders. Journal of Reproductive Medicine for the Obstetrician and Gynecologist, vol. 45, no. 4, pp. 293–298, 2000.
711. Yıldırım P, Basol G, Karahan AY. Pilates-based therapeutic exercise for pregnancy-related low back and pelvic pain: A prospective, randomized, controlled trial. Turkish J Phys Med Rehabilitation. 2022;69(2):207.
712. Young JC, Treadway JL. The effect of prior exercise on oral glucose tolerance in late gestational women. Eur J Appl Physiol Occup Physiol 1992;64:430–3.
713. Youngwanichsetha S, Phumdoung S, Ingkathawornwong T. The effects of mindfulness eating and yoga exercise on blood sugar levels of pregnant women with gestational diabetes mellitus. Appl Nurs Res 2014;27:227–30.
714. Yousefabadi SR, Sarani A, Arbabshastan ME, Adineh HA, Shahnavazi M. The effect of exercise on back pain and lordosis in the second trimester of pregnancy. Drug Invention Today. 2019;11(9):2169–2175.
715. Zeanah M, Schlosser SP. Adherence to ACOG guidelines on exercise during pregnancy: effect on pregnancy outcome. J Obstet Gynecol Neonatal Nurs. 1993;22(4):329–35.
716. Zhang X, Li J, Gu Y, et al. A pilot study on environmental and behavioral factors related to missed abortion. Environ Health Prev Med 2011;16:273–8.
717. Zhang Y, Wang L, Yang W, et al. Effectiveness of low glycemic index diet consultations through a diet glycemic assessment app tool on maternal and neonatal insulin resistance: a randomized controlled trial. JMIR Mhealth Uhealth 2019;74:e12081. https: //doi.org/10.2196/12081
718. Zhang YH. Comprehensive effect assessment of medical nutrition guidance during pregnancy towards the health of mothers and children. Clin Exp Obstet Gyn 2015;42:644–8.
719. Zhou S, Yelland L, McPhee A, Quinlivan J, Gibson R,Makrides M. Fish oil supplementation in pregnancy doesnot reduce the risk of gestational diabetes or preeclampsia.Am J Clin Nutr. 2012;95:1378–84
