# Supplementary material for: Features of smoking cessation, alcohol reduction, and diet and physical activity interventions associated with changing behaviours during pregnancy: a systematic review of reviews
Source: J Behav Med. 2026 Aug 6;49(6):985–1001. doi: 10.1007/s10865-026-00702-1 (PMC13748204; doi:10.1007/s10865-026-00702-1)
Supplement: Supplementary file 6 [file 10865_2026_702_MOESM6_ESM.docx]

**Supplement 6: Author reported conflicts of interest for systematic reviews reporting outcomes for each behavioural domain, and overall quality for all included systematic reviews**

**Supplement 6a: Systematic reviews reporting smoking cessation interventions**

| **Review, author** | **Author reported conflict(s) of interest** |
| --- | --- |
| Acar *et al.* 2022 | Authors declare: **“**No conflict of interest was declared by the authors.” |
| Brown *et al*., 2019 | Authors declare: “None declared.” |
| Chamberlain *et al.* 2013 | No statement of conflict of interest included in the published systematic review |
| Chamberlain *et al.* 2017 | Authors declare: “Catherine Chamberlain is receiving an Australian National Health and Medical Research Council Early Career Fellowship (1088813). She was also awarded an NIHR Cochrane Review Incentive Scheme award (15/81/18) to support the preparation of this updated review. Catherine Chamberlain is also an author on the Cochrane Review entitled ’Pharmacological Interventions to promote smoking cessation in pregnancy“ (Coleman 2015). Alison O’Mara-Eves: none known. Jessie Porter: none known. Tim Coleman has received awards from NIHR, HTA and NIHR paid to his institution. These awards have been used to run research projects. He has also received a single payment from Pierre Fabre Laboratories, France, for speaking at an educational meeting arranged by Pierre Fabre Laboratories (who are manufacturers of nicotine replacement therapy). PFL are a manufacturer of transdermal nicotine patches - the content of the presentation was not vetted and no attempt was made to influence the content of the presentation. Tim Coleman is also an author and contact person for the Cochrane Review entitled ’Pharmacological Interventions to promote smoking cessation in pregnancy” (Coleman 2015). Susan M Perlen: none known. James Thomas: none known. Joanne E McKenzie: none known.” |
| Griffiths *et al.* 2018 | Authors declare: “No potential conflict of interest was reported by the author” |
| Heminger *et al.* 2016 | Authors declare: “The George Washington University/Dr Lorien C Abroms has licensed the Quit4Baby program to Voxiva, Inc.; Dr Lorien C Abroms has stock options in Voxiva, Inc. The authors report no other conflicts of interest in this work.” |
| Hussain *et al.* 2020 | Authors declare: “None declared” |
| Su & Buttenheim 2014 | No statement of conflict of interest included in the published systematic review |
| Tahan *et al* 2023 | Authors declare: “None declared” |
| Wilson *et al.* 2018 | Authors declare: “The authors have no conflicts of interest to declare.” |

**Supplement 6b: Systematic reviews reporting alcohol interventions**

| **Review, author** | **Author reported conflict(s) of interest** |
| --- | --- |
| Froschl *et al.* 2013 | Authors declare: “The authors declare that they have no competing interests.” |
| Gomez *et al.* 2020 | Authors declare: “None” |
| Minozzi et al. 2024 | Authors declare: “Silvia Minozzi is the Joint‐Coordinating Editor of Cochrane Drugs and Alcohol Group. She was not involved in the editorial process of the current review. Ludovico Ambrosi declares no financial or other conflicts of interest. Rosella Saulle declares no financial or other conflicts of interest. Seilin S Uhm declares no financial or other conflicts of interest. Mishka Terplan declares no financial or other conflicts of interest. Julia MA Sinclair declares no financial or other conflicts of interest. Roberta Agabio is an Editor of Cochrane Drugs and Alcohol Group. She was not involved in the editorial process of the current review. |
| Oh *et al.* 2023 | Authors declare: “None declared” |
| Popova *et al.* 2023 | Authors declare: “All authors declare that they do not have any financial, personal, political, intellectual or religious competing interests relevant to this article to disclose.” |
| Samawi *et al.* 2021 | Authors declare: “The authors declare that they have no conflict of interest” |

**Supplement 6c: Systematic reviews reporting diet and/or physical activity interventions**

| **Review, author** | **Author reported conflict(s) of interest** |
| --- | --- |
| Agha *et al.* 2014 | Authors declare: “The authors have declared that no competing interests exist” |
| Allen *et al.* 2014 | Authors declare: “No support from any organization for the submitted work; no financial relationships with any organizations that might have an interest in the submitted work in the previous 3 years; no other relationships or activities that could appear to have influenced the submitted work” |
| Baradwan *et al*. 2024 | Authors declare: “No potential conflict of interest was reported by the author(s).” |
| Beetham *et al.* 2019 | Authors declare: “The authors declare that they have no competing interests.” |
| Bennett *et al.* 2023 | Authors declare: “No potential conflict of interest was reported by the author(s).” |
| Cancela-Carral *et al.* 2022 | Authors declare: “The authors declare there are no conflict of interest” |
| Cantor *et al.* 2021 | Authors declare: “None reported”. |
| Chan *et al.* 2019 | Authors declare: “The authors declare no conflicts of interest” |
| Craemer *et al.* 2019 | Authors declare: “The authors of this study declare no conflict of interest” |
| Currie *et al.* 2013 | Authors declare: “The authors have declared that no competing interests exist.” |
| Davenport *et al.* 2018a | Authors declare: “None declared” |
| Davenport *et al.*  2018b | Authors declare: “No competing interest” |
| Davenport *et al.*  2018c | Authors declare: “None declare” |
| Davenport *et al.* 2019a | Authors declare: “None declared” |
| Davenport *et al.* 2019b | Authors declare: “No competing interest” |
| Di Mascio *et al.* 2016 | Authors declare: “The authors report no conflict of interest.” |
| Gresham *et al.* 2014 | Authors declare: “None of the authors had a conflict of interest” |
| Gresham *et al.* 2016 | Authors declare: “The authors declare that they have no conflicts of interest.” |
| Guillemette *et al.* 2018 | Authors declare: “Laetitia Guillemette, Jacqueline L. Hay, D. Scott Kehler, Naomi C. Hamm, Christopher Oldfield, Jonathan M. McGavock, and Todd A. Duhamel declare that they have no competing interests” |
| Guo et al. 2019 | Authors declare: “None declared” |
| Hanifi *et al.* 2023 | Authors declare: “The authors declare they have no competing interest” |
| Harrison et al. 2023 | Authors declare: “None reported” |
| He *et al.* 2023 | Authors declare: “The authors declare that they have no known competing financial interests or personal relationships that could have appeared to influence the work reported in this paper.” |
| Hill *et al.* 2013 | Authors declare: “The authors declare no conflict of interest” |
| Hussain *et al.* 2020 | Authors declare: “None declared” |
| James *et al.* 2020 | Authors declare: “The authors declare no conflict of interest” |
| Jorge *et al.* 2015 | Authors declare: “The authors declare no commercial relationships or conflict of interests.” |
| Leonard *et al.* 2021 | Authors declare: “The authors declare that they have no conflict of interest” |
| Liu *et al.* 2023 | Authors declare: “The authors declare that they have no competing interests. “ |
| Makaruk *et al.* 2019 | No statement of conflict of interest included in the published systematic review |
| McDonald *et al.* 2016 | No statement of conflict of interest included in the published systematic review |
| Muktabhant *et al.* 2015 | Authors declare: “Declaration of Interest: None known” |
| O’Brien *et al.* 2014 | Authors declare: “no conflict of interest” |
| O’Connor *et al* 2025 | Authors declare: “no competing interests” |
| Perales *et al.* 2016 | Authors declare: “The authors declare no conflict of interest.” |
| Poyatos-Leon *et al.* 2015 | Authors declare: “The authors have stated explicitly that there are no conflicts of interest in connection with this article.” |
| Rhodes *et al.* 2020 | Authors declare: “CL reports grants from the Economic and Social Research Fund, grants from Best Beginnings (UK charity), during the conduct of the study; personal fees from Yellow Kite; personal fees from the experiment; and personal fees from Diamond Inc, outside the submitted work. The remaining authors have no conflicts of interest to declare.” |
| Sanabria-Martin *et al.* 2015 | Authors declare: “None declared” |
| Sheffield and Woods-Giscombe 2016 | No statement of conflict of interest included in the published systematic review |
| Sherifali *et al.* 2017 | Authors declare: “None declared” |
| Skouteris *et al.* 2014 | Authors declare: “Helen Skouteris declares that she has no conflict of interest. Heather Morris declares that she has no conflict of interest. Cate Nagle declares that she has no conflict of interest. Alison Nankervis declares that she has no conflict of interest.” |
| Soltani *et al.* 2016 | Authors declare: “The authors declare that there is no known conflict of interests.” |
| Tieu *et al.* 2017 | Authors declare: “Declarations of interest: Joanna Tieu: none known. Emily Shepherd: none known. Philippa Middleton: none known. Caroline A Crowther: none known.” |
| Tombers et al. 2023 | Authors declare: “The authors declare no conflicts of interest.” |
| Turan et al. 2025 | Authors declare: “The authors declare no competing interest.” |
| Vincze *et al.* 2019 | Authors declare: “There is no conflict of interest in this project” |
| Walker *et al.* 2018 | Authors declare: “The authors declared no conflicts of interest” |
| Wang *et al.* 2019 | Authors declare: “No conflicts of interest to disclose” |
| Yin *et al.* 2013 | Authors declare: “Competing interests – none” |
| Zenitasari et al. 2025 | Authors declare: “The authors declare that they have no known competing financial  interest or personal relationship that could have appeared to influence  the work reported in this paper.” |
| Zhang et al. 2023 | Authors declare: “The authors certify that there is no conflict of interest with any financial organization regarding the material discussed in the manuscript.” |
