# Supplementary material for: Features of smoking cessation, alcohol reduction, and diet and physical activity interventions associated with changing behaviours during pregnancy: a systematic review of reviews
Source: J Behav Med. 2026 Aug 6;49(6):985–1001. doi: 10.1007/s10865-026-00702-1 (PMC13748204; doi:10.1007/s10865-026-00702-1)
Supplement: Supplementary file 7 [file 10865_2026_702_MOESM7_ESM.docx]

**Supplementary files 7-11**

Contents

[Supplement 7 - Any diet/PA interventions (n=11)	2](#_Toc216769475)

[Supplement 8 - Physical activity-focused interventions (n=29)	6](#_Toc216769476)

[Supplement 9 - Diet-focused interventions (n=6)	12](#_Toc216769477)

[Supplement 10 - mHealth interventions (n=5)	14](#_Toc216769478)

[Supplement 11 – Smoking cessation Interventions (n=10)	16](#_Toc216769479)

[Supplement 12 – Alcohol Interventions (n=6)	19](#_Toc216769480)

Note:

GWG – Gestational Weight Gain

GDM – Gestational Diabetes Mellitus

LGA – Large-for-gestational age

SGA – Small-for-gestational age

PA – Physical activity

PE – Preclampsia

GH – Gestational hypertension

# Supplement 7 - Any diet/PA interventions (n=11)

| **Outcome** | **Author** | **Interventions** | **Findings relating to intervention features** |
| --- | --- | --- | --- |
| GWG | Agha 2014 | Behavioural interventions (no definition provided) | Interventions were more significant when delivered in the hospital setting (MD -1.85kg, 95% CI = -3.53 to -0.18) but were not significant when delivered in the community (MD=-0.67kg, 95%CI=-2.06 to 0.73). |
|  |  |  | Factors most used in successful interventions involved PA and diet counselling by a dietician, physician or a midwife supplemented by motivational talks on weight management, delivered in early pregnancy, and included feedback on the progress of participants and weight monitoring during pregnancy. |
|  |  |  | - Sub-analyses revealed both interventions with *or* without feedback and/or weight monitoring showed no difference from control groups (MD -2.13kg, 95% CI -4.38 to 0.13, and MD -0.98kg, 95% CI -2.28 to 0.32, respectively) - No significant difference between control groups in comparison to passive interventions (advice alone) (MD – 1.98kg, 95% CI -4.10 to 0.13) or pro-active interventions (PA/diet included) (MD -1.0kg, 95%CI -2.47 to 0.47) |
|  | Cantor 2021 | Diet, exercise, and/or behavioral counseling interventions that targeted GWG | - High-intensity interventions (>11 contacts) associated with greater effects on GWG (MD −1.47kg, 95% CI −1.78 to −1.22) than moderate-intensity (3-11 contacts) (MD −0.32 kg, 95% CI −0.71 to −0.04) or low-intensity (<2 contacts) interventions (MD −0.64kg, 94% CI −1.44 to 0.02]; *P* < .001 for interaction). - Significant interactions between effects of interventions on excess weight gain and intervention type (Active or counselling-only, *P* = .003) and intensity (*P*<.001 for interaction), with statistically significant effects in the active (RR 0.73, 95%CI 0.67 to 0.80) and high-intensity (RR 0.74, 95%CI 0.69 to 0.79) subgroups. |
|  | Craemer 2019 | Diet, PA or mixed interventions | Of PA interventions, neither supervised (p=.61) nor unsupervised (p=.49) programs produced significant results. |
|  | Harrison 2023 | Diet, PA, or mixed interventions in pregnant individuals that reported mean GWG per group | - Interventions delivered by an allied health professional associated with a greater decrease in GWG compared with those delivered by other facilitators (MD −1.36kg; 95%CI −1.71 to −1.02; *P* < .001) with no facilitator (i.e, remote delivery) being non-efficacious (MD −0.25 kg; 95%CI −0.98 to 0.48). - Of dietary interventions, individual delivery format (MD -3.91kg, 95%CI -5.82 to -2.01) was associated with a greater decrease in GWG compared with group format (MD -0.23 kg, 95%CI -1.28 to 0.82; P = .002) and moderate number (6-20) of sessions (MD −4.35kg, 95%CI −5.80 to −2.89; *P* < .001) were associated with the greatest decrease in GWG compared to those with low number of sessions (1-5). |
|  |  |  | - Across all interventions, the association of intervention session number, length of sessions, duration of intervention, provision of ongoing support or resources, and tailoring with reduction in GWG could not be delineated. - Of dietary interventions, intervention tailoring, provision of resources, and ongoing support were not associated with GWG. - Of PA and mixed interventions, no significant differences across subgroups of facilitator, intervention format, or mode found; and intervention theoretical underpinning, delivery setting, number and length of sessions, duration of intervention, number and length of sessions, provision of resources/ ongoing support, nor tailoring were associated with differences in GWG reduction. |
|  | Hill 2013 | GWG interventions derived from theories of behaviour change | - Significant differences favouring the intervention group were reported for four techniques (i.e. studies that used this technique were more effective than studies that did not); providing information on the consequences of behaviour to the individual (p=.010; *d* = -0.11), provide rewards contingent on successful behaviour (p=.042, *d* = -0.11), prompt self-monitoring of behaviour (p=.036, *d* =-0.07), and motivational interviewing (p=.016, *d* = -0.11). - The following techniques were not used in more effective interventions: outcome goal setting (p=.001, *d* = 0.11), action planning (p=.04, *d* = 0.07), prompt rewards contingent on effort of progress towards behaviour (p=.007, *d* = 0.21), prompt self-monitoring of behavioural outcome (p<.002, *d* = 0.10), use of follow-up prompts (p<0.001, *d*=0.15), and facilitate social comparison (p=.038, *d* = 0.09). |
|  |  |  | - In general, more BCTs were reported in effective interventions and there was a trend for interventions with a larger number of BCTs to report greater differences in GWG between the intervention and control groups (*r*= 0.30, *P* = 0.10) - Studies based on theory were as effective as studies that were supposedly non–theory-based at limiting GWG |
|  | Muktabhant 2015 | Diet, PA or mixed interventions | - Reductions in favour of the intervention arms consistent across different types of interventions, except for the heterogenous group comprising 'diet counselling or other interventions', with the largest reduction occurring with 'supervised exercise and diet' interventions. - Women in the intervention group were significantly more likely to experience low GWG compared with the control group but results according to types of interventions were not statistically significant. However, when supervised and unsupervised exercise groups were combined the effect was of borderline statistical significance. |
|  |  |  | Interventions involving low glycaemic load diets, supervised or unsupervised exercise only, or diet and exercise combined all led to similar reductions in the number of women gaining excessive weight in pregnancy. |
|  | Soltani 2016 | Dietary or lifestyle interventions with potential to influence maternal and fetal outcomes related to weight | - The BCT categories present in 50% or over of successful interventions were “feedback and monitoring,” “goals and planning,” and “shaping knowledge.” - Not possible to statistically analyse the individual effectiveness of BCTs or to assess the effectiveness of different combinations of BCTs. However, was noted that successful interventions always included BCTs from one or both of “goals and planning” or “feedback and monitoring”. |
|  | Skouteris 2014 | Behavioral interventions designed to prevent GDM | Use of self-monitoring and goal setting appear effective in the prevention of excessive GWG, especially when combined with a high frequency of intervention contact. |
|  | Walker 2018 | Interventions designed to prevent excessive GWG | - Interventions delivered to women individually were significantly more likely to be ineffective (ineffective 66.7%, effective 33.3%; *X*2 4.43; p=.04). In contrast, when combining interventions delivered to groups and those delivered to individuals with a group component involved, a significantly larger proportion were effective (effective 62.5%, ineffective 37.35%; x2 5.06; p=.02). - PA intervention effectiveness was significantly higher in studies that combined individual and group-delivery of interventions, compared with studies that were delivered to individuals only or groups only (B=-.99, 95% CI -3.45 to -0.52, p=0.01) |
|  |  |  | - Trimester initiated, frequency of sessions, intensity of intervention, nor duration influenced pooled effect size in each meta-analysis - Three eHealth interventions were included in a meta-analysis which favoured neither intervention nor control (WMD -1.06; 95% CI -4.13 to 2.00, p=.50) |
| GDM | Cantor 2021 | Diet, exercise, and/or behavioral counseling interventions that targeted GWG | Stratified analysis by intervention type (active vs. counselling-only) and intervention intensity (<2 contacts, 3-11 contacts, >11 contacts) showed no difference in association with GDM likelihood. |
|  | Guo 2019 | Lifestyle interventions with potential to prevent GD. | - Effect size was signiﬁcantly correlated with the initiation time of the intervention - Exercise of moderate intensity for 50–60 minutes twice a week could lead to an approximately 24% reduction, and to an approximately 35% reduction with exercising three times a week |
|  | Skouteris 2014 | Behavioral interventions designed to prevent GDM | - Studies with a high frequency of contact were effective for prevention of GDM - In all except 1 case, self-monitoring was used in studies that provided evidence of preventing GDM. However, efficacy of goal setting is yet to be established - The evidence is inconclusive in regards to the provision of education on an individual basis over a group format. |
| Preterm birth | Cantor 2021 | Diet, exercise, and/or behavioral counseling interventions that targeted GWG | Stratified analysis by intervention type (active vs. counselling-only) and intervention intensity (<2 contacts, 3-11 contacts, >11 contacts) showed no difference in association with likelihood of preterm birth. |
| PE | Allen 2014 | Diet/lifestyle interventions with potential to modify metabolic risks | Excluding studies where the intervention commenced after the ﬁrst trimester showed no signiﬁcant reduction in preeclampsia (RR 0.95, 95% CI 0.74–1.22, p=.69). |
| Hypertension | Cantor 2021 | Diet, exercise, and/or behavioral counseling interventions that targeted GWG | Stratified analysis showed statistically significant interactions between effects of interventions on risk of GH and intervention type and intensity (P<.001 for interactions). There were statistically significant effects in the active interventions (RR 0.60, 95% CI 0.41 to 0.82; p<.001) and high intensity (>11 contacts) (RR 0.69, 95% CI 0.50 to 0.91, p=.006) intervention subgroups. |
| Caesarean delivery/emergency caesarean delivery | Cantor 2021 | Diet, exercise, and/or behavioral counseling interventions that targeted GWG | Stratified analysis by intervention type (active vs. counselling-only) and intervention intensity (<2 contacts, 3-11 contacts, >11 contacts) showed no difference in association with likelihood of caesarean delivery. |
|  | Muktabhant 2015 | Diet, PA or mixed interventions | No statistically significant difference in the risk of caesarean section between intervention and control groups but estimates for supervised and unsupervised exercise interventions only, alone or combined, were robust to the overall findings. However, for the diet and exercise counselling interventions, the effect approached statistical significance with a 13% reduction in caesarean delivery in the intervention group (RR 0.87, 95% CI 0.75 to 1.01; p=0.06) |
| Macrosomia/LGA | Cantor 2021 | Diet, exercise, and/or behavioral counseling interventions that targeted GWG | Stratified analyses showed statistically significant interactions between effect of interventions on risk of macrosomia and intervention intensity (P =.03 for interaction). Significant effects were demonstrated in the high-intensity intervention subgroup (>11 contacts) (RR 0.65, 95% CI 0.49 to 0.84). |
|  |  |  | - Stratified analyses by intervention type (active vs. counselling-only) showed no difference in association with likelihood of macrosomia. - Stratified analysis by intervention type (active vs. counselling-only) and intervention intensity (<2 contacts, 3-11 contacts, >11 contacts) showed no difference in association with likelihood of LGA infants. |
|  | Muktabhant 2015 | Diet, PA or mixed interventions | For the 'supervised exercise' intervention group, the effect in favour of the intervention bordered on statistical significance for macrosomia (RR 0.81, 95%CI 0.64 to 1.02; p=.07) but not when supervised and unsupervised exercise interventions were combined (RR 0.87, 95% CI 0.71 to 1.07). |

# Supplement 8 - Physical activity-focused interventions (n=29)

| **Author** | **Outcome** | **Interventions** | **Findings relating to intervention features** |
| --- | --- | --- | --- |
| Baradwan 2024 | Obstetric, maternal, and neonatal outcomes | Pilates | In comparison to control group, the pilates group demonstrated significantly higher rate of vaginal birth (87% vs. 70%) (RR 1.26, 95% CI 1.18 to 1.34, p < .001), lower incidence of cesarean delivery (12% vs 25%) (RR 0.45, 95%CI 0.34 to 0.61, *p* < .001), greater reduction in the length of labor (MD −84.96, 95% −130.94 to −38.97, *p* = .003), lower incidence of episiotomy during delivery (34% vs 48%) (RR 0.69, 95%CI 0.58 to 0.82, *p* < .001), improved 1-minute Apgar score (MD 0.34, 95%CI 0.25 to 0.43, *p* < .001), improved 5-minute Apgar score (MD 0.16, 95%CI 0.09 to 0.23, *p* < .001) and decreased number of women requiring epidural analgesia (44% to 69%) (RR 0.65, 95%CI 0.47 to 0.89, *p*=.008). |
| Beetham 2019 | Birth weight, incidence of small for gestational age, low birth weight, risk of prematurity, gestational age at delivery and maternal weight gain | Intervention of pregnant women performing vigorous exercise | Women who engaged in vigorous PA in the third trimester had a small but significant increase in length of gestation (MD 0.21 weeks; 95% CI 0.15 to 0.27, g= 0.15, p<.001) and a small but significantly reduced risk of prematurity (RR=− 0.20; 95% CI − 0.36 to − 0.03, p=.03) |
|  |  |  | - No significant difference existed in maternal weight gain or birthweight outcomes in mothers who engaged in vigorous PA and those who did not. - Not possible to determine influence of intensity, frequency, duration, or volume of exercise |
| Bennett 2023 | GDM | Supervised exercise-exclusive interventions | Supervised PA reduces the incidence of GDM when initiated in first trimester (RR 0.57, 95% CI 0.41–0.79; p=.001) and by accumulating >600 MET-min of exercise (RR 0.77, 95% CI: 0.60 to 0.98; p=.03). |
|  |  |  | No significant risk reduction with interventions starting after first trimester |
| Cancela-Carral 2022 | GWG, birth weight, fitness, anthropomorphic and cognitive factors | Aquatic PA intervention | - Positive effects in the prevention of excessive maternal weight gain (MD −1.66kg, 95% CI −2.67 to −0.66) and reducing birth weight mean differences (MD −89.13g, 95% CI −143.18 to −35.08). - Programs with a greater volume of load produced greater beneﬁts than those programs which had a greater intensity of load |
|  |  |  | Appears to have positive effects on preventing excessive maternal weight gain, improving maternal body image, as well as promoting healthy behavior, decreasing medical leave due to lower back pain during pregnancy, preventing gestational depression by improving maternal glucose tolerance levels |
|  |  |  | Both aquatic and non-aquatic exercise programs help regulate newborn weight, with no signiﬁcant differences between them |
| Chan 2019 | GWG, pain and depression, self efficacy, PA level, quality of life | Land based PA interventions or lifestyle interventions that contain a PA component | - Interventions eﬀective in enhancing PA levels and potentially useful in alleviating pregnancy-related pain and psychological symptoms, reducing GWG, and increasing self-eﬃcacy in enhancing PA levels. - A generally consistent positive eﬀect of interventions including a compulsory exercise program was reported among studies investigating the eﬀects of interventions on PA level. |
|  |  |  | Heterogeneity of multiple confounding characteristics of the prevent ﬁrm conclusions regarding intervention factor effectiveness |
| Currie 2013 | Change in PA | Interventions designed to maintain or increase PA during pregnancy | - In 8/10 studies, participants in the intervention group were more physically active post intervention than controls. - Most effective BCTs appear to be goals and planning with feedback - Interventions with regular face-to-face meetings were more likely to produce positive changes in PA behaviour. |
| Davenport 2018a | Prenatal and postnatal anxiety and depression | Exercise interventions | - PA-only interventions were associated with a reduction in severity of antenatal depressive symptoms, with greatest reduction in women who were supervised. - Meta-regression analyses identified the following thresholds to a achieve a moderate effect in reducing prenatal depression: an exercise intensity of 4.0 METs/min (eg, light walking), exercise duration>49.9 min per session; exercise frequency >2.6 times per week; and volume of exercise per week >644 MET-min/week (eg, 150 min of moderate intensity exercise, such as brisk walking, water aerobics, stationary cycling, resistance training). |
| Davenport 2018b | GDM, PE, Gestational hypertensive | Exercise interventions | Meta-regression analyses identified that to achieve at least a 25% reduction in the odds of developing GDM, PE and GH, pregnant women need to accumulate at least 600 MET-min/week of moderate-intensity exercise (eg, 140 min of brisk walking, water aerobics, stationary cycling or resistance training). Results from meta-regression analyses suggested that the benefits would be attained when exercise is performed at a frequency of at least 3 days/week or at least 25 min per session. |
| Davenport 2018c | Glycaemic control: Maternal blood glucose before, during and after exercise, fasting blood glucose | Exercise interventions | Meta-regression analyses demonstrated an inverse dose–response relationship between volume of exercise (MET minutes per sessions) and glycaemic response post-acute exercise, indicating that larger volumes of exercise translated into greater reductions in blood glucose after an acute bout of PA |
| Davenport 2019a | Miscarriage, perinatal mortality | Exercise interventions | Meta-regressions identified no associations between exercise volume, intensity or frequency and the odds of fetal or newborn mortality |
| Davenport 2019b | Delivery and labour outcomes (See Supplement 3) | Exercise interventions | Meta-regression analyses did not identify a dose–response relationship between frequency, intensity, duration or volume of exercise and any outcome |
| DiMascio 2016 | Preterm birth  gestational age at delivery, spontaneous vaginal delivery, operative vaginal delivery, cesarean delivery, GDM, hypertensive disorders (defined as GH or PE), birthweight and low birthweight <2500g. | Aerobic exercise regimen beginning before 23 weeks that did not include diet, counselling, and/or weight monitoring | Compared to controls, women in the exercise group had a significantly higher incidence of vaginal delivery (RR 1.09, 95%CI 1.04-1.15), a significantly lower incidence of cesarean delivery (RR 0.82, 95% CI 0.69 to 0.97), GDM (RR 0.51, 95% CI 0.31 to 0.82), and hypertensive disorders (RR 0.21, 95% CI 0.09 to 0.45) |
|  |  |  | - No differences in operative vaginal delivery, low birthweight, nor mean birthweight between the exercise group and controls were found - Subgroup analyses by type or length of exercise session showed no significant difference |
| Guillemette 2018 | Offspring cardiometabolic risk factors including weight, adiposity, and blood pressure at birth and in childhood, the effects of maternal body mass index (BMI) and training variables. (Birth weight, large-for-gestational age status, fat and lean mass, dyslipidemia, dysglycemia, and blood pressure) | Aerobic and/or resistance (strength) training exercises ≥4weeks in duration | - Interventions did not significantly impact birth weight (MD -22.1g, 95% CI - 51.5 to 7.3) or LGA incidence (RR 0.85, 95% CI 0.51 to 1.44), but sub-group analyses showed that PA reduced birth weight according to timing (starting after 20 weeks of gestation (MD -84.3g, 95% CI - 142.2 to - 26.4), type of exercise (MD - 58.7g, 95% CI - 109.7 to - 7.8), and PA intensity (MD - 45.5g, 95% CI - 82.4 to -8.6). - Aerobic-only training reduced birth weight, but resistance training only, or combined regimens had non-significant effects - Interventions that were light to moderate intensity reduced birth weight but not those that were moderate to vigorous intensity |
|  |  |  | Frequency of exercise did not impact birth weight (less vs more than 3 weeks) |
| He 2023 | Depression | PA interventions targeting depression | - There was no difference in the severity of depression between the PA and control group in general population pregnant women (WMD -0.35, 95%CI −1.42 to 0.71, p=.52). However, with respect to the intervention stage, PA intervention during the 2nd trimester could reduce depression (WMD -0.90, 95% CI −1.60 to −0.20, p=.012). - When METs-min/week was ≥450, (WMD -0.90, 95%CI: −1.55 to −0.24, p=.007), the PA group had lower depression than the control group. - Risk of postpartum depression was lower in the PA group when duration of intervention was ≥12 weeks (RR 0.56, 95% CI 0.44 to 0.72, p<.001). - In the general population, moderate exercise intervention could decrease the risk of postpartum depression (RR 0.36, 95%CI 0.22 to 0.60, p<.001). |
| James 2020 | Behavioural outcomes only (change in PA levels) | Intervention designed to promote PA | All 5 significant trials (of 15) conducted at least one individual interview |
|  |  |  | Interventions seem to have a low impact on the promotion of PA during pregnancy. Does not appear to be one single technique that has an impact on PA increase |
| Jorge 2015 | Wide array of outcomes (See Supplement 3) | Group exercise programs | - Significant effects of the programs are related with improved maternal perception of health status, lower maternal weight gain, improved levels of maternal glucose tolerance, improved aerobic fitness and muscular strength, lower frequency of urinary incontinence, improved sick leave due to lumbopelvic pain, fewer cesarean and instrumental deliveries, higher newborn Apgar score and faster postpartum recovery. |
|  |  |  | Little consistency in the methods used in studies to assess the impact of intervention duration or intensity |
| Liu 2023 | Depression | Exercise intervention for depression | - PA positively improved antenatal depression (with medium effect, d=-0.56) - The single intervention duration of 10–75 min all had a good effect on antenatal depression, and 30–60 min had the best effect - The order of effect size of the exercise type on antenatal depression was Yoga (d=-0.59) and a combination of aerobic exercise(d=-0.58). - The intervention frequency of 3 to 5 times/week had the greatest amount of intervention effect on maternal depression - Exercise lasting 6–10 weeks had a good intervention effect on antepartum depression, and the amount of effect decreased gradually with the extension of time - In terms of exercise format, the amount of intervention effect on maternal depression was in the order of group exercise, individual + group exercise. |
|  |  |  | Group exercise 3–5 times a week for 30–60 min for 6–10 weeks is more likely to achieve the desired intervention effect of improving antenatal depression. |
| Makaruk 2019 | GDM | Exercise programme supervised and led by a pregnancy exercise specialist | Based on the analysis of 2 effective programs from 10 studies overall, the authors concluded that interventions should take place at early pregnancy stage (i.e. first trimester), have a long duration of sessions (72-90 sessions), high adherence, and three weekly sessions lasting up to 60 minutes each |
| McDonald 2016 | GWG | PA focused interventions | No discernible patterns of exercise dose (frequency, duration, intensity) and the reductions in GWG within the ‘successful’ vs ‘unsuccessful’ interventions |
| Perales 2016 | 1) GWG; 2) Cardiorespiratory fitness; 3) Muscle strength; 4) Urinary incontinence; 5) GDM; 6) Cesarean section; 7) Duration of labour; 8) Lumbopelvic pain; 9) Blood pressure. 10) Birth weight; 11) Macrosomia | Aerobic, resistance or combined (aerobic + resistance) exercise | Evidence for a beneﬁt of combined exercise [aerobic + resistance (muscle strength)] interventions on maternal cardiorespiratory ﬁtness and prevention of urinary incontinence was strong |
|  |  |  | Weak or insufﬁcient evidence found for other interventions and outcomes |
| Poyatos-Leon 2015 | Mode of delivery (i.e. normal, operative and caesarean) | Supervised exercise | Regular PA during pregnancy slightly increases the probability of normal deliveries. Subgroup analyses excluding studies carried out throughout pregnancy showed a higher rate of normal delivery and lower rate of caesarean delivery in the intervention group when exercise was performed during the second (RR 1.14; 95% CI 1.01 to 1.32; p=.048) and third trimesters (RR 0.66; 95% CI 0.46 to 0.96; p=.028) |
| Sanabria-Martin 2015 | Risk of GDM, Maternal weight | Low-to-moderate intensity exercise programmes | - Decreased risk of GDM (RR 0.69; 95% CI 0.52 to 0.91; p=.009) and a diminished GWG (WMD -1.14 kg; 95% CI -1.50 to -0.78; p<.001) in the exercise group. - PA interventions decreased the risk of GDM, particularly when the exercise programme was performed throughout pregnancy (RR 0.64; 95% CI 0.36 to 0.98, p=.038) and with combined aerobic, strength, and flexibility exercises (RR 0.69; 95% CI 0.48 to 0.99; p=.04). - Decreases in maternal weight observed when PA conducted throughout pregnancy (WMD -1.16kg, 95% CI -1.47 to -0.85, p<.001) and from the second trimester (WMD -1.03kg, 95% CI -1.48 to -0.59, p<.001) - Decreases in maternal weight were observed for both aerobic exercise (WMD -0.81kg; 95% CI -1.42 to -0.20; p<.001), and combined aerobic, strength, and flexibility (WMD -1.18kg; 95% CI -1.46 to -0.90; p<.001) |
|  |  |  | No difference between light intensity versus moderate intensity interventions |
| Sheffield 2016 | **Primary outcomes**  1)Depression;  2) Anxiety  **Secondary outcomes**  See Supplement Table 3 | Yoga | - Significant decreases in depression and anxiety observed regardless of yoga intervention style or outcome measure; however, only two of the studies reviewed reported between-group differences. - Decreases in anxiety and depression optimized in studies longer than 7 weeks. |
| Tombers 2023 | Pain intensity prior to delivery | PA including aerobic, aquatic, resistance training, mobility exercise, core stabilization, or relaxation exercises (supervised or unsupervised) | Meta-analysis found that the effect size of the benefit of exercise on pain decreased as the duration of the intervention increased (Slope -0.3381, p=.000) |
|  |  |  | Although sub­group meta-analyses performed for exercise type (aquatic, core stability, aerobic, mobility, relaxation) were all significantly different from one another, all exer­cise types indicated a reduction in pregnancy-related lumbopelvic pain. Because of large heterogeneity among studies, authors state this analysis should be interpreted with caution. |
| Turan 2025 | All outcomes eligible but subgroups of pain, sleep, depression, and quality of life | Pilates | - Pilates interventions demon­strated positive impact on various outcomes, including pain management, sleep quality, mental health, urinary incontinence, pelvic floor function, and birth outcomes. - Differences in intervention duration, session frequency, and Pilates methodology across studies may have influenced the reported outcomes. - Interventions lasting 8 to 12 weeks, conducted 2–3 times per week for 50–70 min per session, yielded most significant improvements across physical and mental health outcomes. Moreover, studies supervised by physiotherapists/cer­tified instructors demonstrated superior results, |
| Wang 2019 | GWG | PA interventions targeting GWG | - GWG was decreased significantly for women having PA from the 1st to the 3rd gestational trimester (WMD -1.42, 95% CI -1.85 to -0.98) and the 2nd to the 3rd gestational trimester (WMD -1.42, 95% CI -1.85 to -0.98). However, women who had PA from the 2nd trimester to delivery did not show decreased GWG (WMD 0.06, 95% CI -1.15 to 1.27). - GWG decreased with physical exercise 3 times per week (WMD -1.22; 95% CI -1.55 to -0.9); women having PA once to twice or 4 to 5 times per week did not show a significant decrease. - GWG was decreased significantly in trials with duration of exercise ranging from 30-45 minutes (WMD -1.32, 95% CI -1.79 to -0.85) and 50 to 60 minutes (WMD-0.99, 95% CI -1.42 to 0.57) |
|  |  |  | GWG decreased with light, moderate to light and moderate intensity of exercise; with no significant difference between groups (p=.357) |
| Yin 2013 | GDM | Interventions with PA as only intervention | Evidence was insufficient to suggest that PA during pregnancy might be effective to lower the risk of developing GDM |
|  |  |  | Concluded that better to perform physical exercise before pregnancy or in early pregnancy than during the second and third trimesters. |
| Zenitasari et al. 2025 | Labor pain and duration of labor | Pilates | - Pooled MD in pain scores during delivery across all studies was -1.13 (95% CI -1.64 to -0.61), indicating a statistically significant reduction in pain scores for the Pilates group compared to controls - Subgroup analysis revealed differences in session frequency and participant parity primarily drove heterogeneity in pain reduction. - Pilates significantly reduced duration of labor compared to control group (MD -97.84.min, 95%CI -136.7 to -58.98), with little heterogeneity between studies. |
| Zhang et al 2023 | GDM, GH, and pregnancy outcomes | Aerobic exercise with no restriction on type of exercise | - Compared to control group, intervention group had significantly lower incidence of GDM (OR 0.39, 95%CI 0.3 to 0.5, p<0.00001) and GH ()R 0.38, 95%CI 0.27 to 0.54, p<0.00001) - Both analyses highlighted high homogeneity in effect across studies |
|  |  |  | Different exercise intensities, durations, frequencies of interventions, and exercise modalities were not compared due to trial population specificity. |

# Supplement 9 - Diet-focused interventions (n=6)

| **Author** | **Outcome** | **Interventions** | **Findings relating to intervention features** |
| --- | --- | --- | --- |
| Gresham 2014 | Neonatal outcomes (e.g. birth weight, length) | Dietary interventions | Food and fortified food product interventions were effective in increasing birth weight (SMD 0.27; 95% CI 0.14 to 0.40; p=.01) and reducing the incidence of low birth weight (SMD 20.22, 95% CI 20.37 to 20.06, p<.01) but no effect of dietary counselling interventions. |
|  |  |  | No effect of either type of dietary intervention on length, or perinatal mortality placental weight, head circumference, macrosomoia, APGAR score, SGA, and LGA. |
| Gresham 2016 | Pregnancy outcomes (not GWG), GDM, pregnancy hypertensive disorders, Maternal blood pressure, caesarean section, length of gestation | Dietary interventions | - Dietary counselling reduces maternal blood pressure, but analysis not run for food and food product interventions - Dietary interventions focused on modifying macronutrient intakes during pregnancy reduces the incidence of preterm delivery (p=.01. |
|  |  |  | Meta-analysis demonstrated no effect of type or any dietary intervention on hypertensive disorders, GDM, caesarean delivery, or gestational length. |
| Hanifi 2023 | Low birth weight, SGA, LGA, macrosomia, infant mortality | Registered dietician (RD) involvement | Four of 5 studies that compared RD involvement to no RD involvement found a lower prevalence of LBW in groups with RD involvement. There was no significant difference in SGA prevalence between the RD involvement intervention group and the control group. |
| O’Connor 2025 | Between group change in the following dietary measures: fruits, vegetables, grains and cereals, meat and alternatives, dairy, discretionary foods and dietary quality measure scores. GWG (according to pre-pregnancy body mass index (BMI)), GDM diagnosis and maintenance of dietary change post pregnancy | Dietary interventions | - The BCT category Reward and threat was successful in improving dietary quality and vegetable intake (SMD 0.38, 95% CI 0.21 to 0.55, and SMD 1.43, 95% CI 0.44 to 2.42, respectively), whilst ’Action planning’ significantly improved dietary quality (SMD 0.65, 95% CI 0.25 to 1.04). - Interventions that did not include ‘Discrepancy between current behaviour and goal’ were associated with a higher dietary quality compared with interventions that did include it (SMD 0.30, 95% CI 0.30-1.01) - Interventions that did not include ‘Feedback on the behaviour’ were associated with significantly improved vegetable intake (SMD 0.32, 95% CI 0.17 to 0.47) compared to those that did include it. - Interventions delivered by a nutrition professional or health professional had significantly improved dietary quality compared that did not (SMD 0.57, 95% CI 0.25 to 0.88, p=.0005, and SMD .20, 95% CI 0.11 to 0.28, p<.00001, respectively) - Interventions that included group sessions improved dietary quality (SMD 0.97, 95% CI 0.41 to 1.53, p=.0007) and vegetable intake (SMD 0.43, 95% CI 0.31 to 0.56, p<.00001) more so than interventions that did not include group sessions or were mixed in delivery. |
|  |  |  | - No impact on effectiveness was seen from all other intervention characteristics including the mention of the underlying theory, the number of included BCTs, other forms of intervention delivery (individualised, individualised face to face, individual face-to-face only), frequency of intervention delivery, setting, or duration. - Due to high heterogeneity in dietary measures and timeframes the BCTs used to sustain dietary change postpartum were unable to be meta-analysed. |
| Tieu 2017 | GDM, LGA, GMD, caesarean birth  See Supplement Table 3 | Dietary advice | No clear differences were shown in the risks of GDM (RR 0.91, 95% CI 0.63 to 1.31), LGA (RR 0.60, 95% CI 0.19 to 1.86; p=.07), caesarean birth (RR 1.27, 95% CI 0.79 to 2.04), nor GWG (MD -1.23 kg, 95% CI -4.08 to 1.61) between the low-GI and moderate- to high-GI dietary advice groups. The trials did not report on: hypertensive disorders of pregnancy; perinatal mortality; neonatal mortality and morbidity. |
| Vincze 2019 | GWG | Weight management interventions which included a diet component aimed at limiting GWG | - Of the 11 studies reporting significantly less GWG for intervention versus comparator groups, nine provided contact at least monthly with either, individual, group, SMS, postal or a combination of these modalities. However, 7 of 9 unsuccessful interventions also provided some kind of monthly contact, with at least one being an in-person session - Eight of the 11 successful studies included individual in-person sessions with a dietitian (73%) compared with only 4 of 9 unsuccessful interventions (44%). |
|  |  |  | Lack of clarity within for antenatal interventions as to what were effective components in addition to diet. No clear pattern observed regarding the addition of PA advice or structured PA |

# Supplement 10 - mHealth interventions (n=5)

| **Author** | **Outcome** | **Interventions** | **Findings relating to intervention features** |
| --- | --- | --- | --- |
| Hussain 2020 | GWG, birth weight, small or large for gestational age, GDM | mHealth interventions used in pregnancy in high-income countries | Of 9 eligible studies, the 3 interventions associated with less overall GWG were all multimodal and incorporated at least one additional communicative technology (Facebook or emails) alongside its main intervention (text messages or an app). The 2 studies that evaluated GWG and utilized interactive text messages or an app alone exhibited no difference in GWG compared to controls |
|  |  |  | - Mixed findings regarding PA behaviour - No difference in incidence of GDM in any study |
| Leonard 2021 | GWG; PA and healthy eating. | Technology-supported interventions targeting GWG, PA and/or diet. | - Technology-supported RCTs had small effects on GWG (d= 0.23), PA (d= 0.38), energy intake (d= 0.38), and eating behaviors (d= 0.16). - For GWG studies, the technology and no face-to-face interventions (d = 0.34), interventions using a combination of technology tools (i.e., Wi-Fi weight scale plus smartphone application; d = 0.43), interventions using technology tools daily (d = 0.32), and interventions with technology-behavior correspondence (d = 0.38) resulted in larger effect sizes compared with the technology and face-to-face interventions (d = 0.27), interventions using a tracking tool only (d = 0.26) or website/smartphone application only (d = 0.18), interventions that did not use technology daily (d = 0.27), and interventions with no technology-behavior correspondence (d = 0.24), respectively. - For PA studies, the technology and face-to-face interventions (d =0.56), interventions using a tracking tool (d = 0.65), and interventions using technology tools daily (d = 0.57) resulted in larger effect sizes compared with the technology and no face-to-face interventions (d = 0.23), interventions using a website/smartphone application only (d = 0.30) or a combination of tools (i.e., pedometer plus smartphone application; d =0.35), and interventions that did not use technology daily (d =0.28), respectively. - For healthy studies, the technology and face-to-face interventions (d = 0.38), interventions using a tracking tool (d = 0.44), interventions using technology tools daily (d = 0.41), and interventions with no technology behavior correspondence (d = 0.50) resulted in larger effect sizes compared with the technology and no face-to-face interventions (d = 0.31), interventions using a website/smartphone application only (d = 0.27) or a combination of tools (i.e., food diary plus smartphone application; d = 0.29), interventions that did not use technology daily (d = 0.32), and interventions with technology-behavior correspondence (d = 0.26), respectively. |
| O’Brien 2014 | Primary: 1) fasting maternal glucose; 2) GWG; 3) GDM. Secondary: 1) Uptake/acceptance; 2) dietary/PA | Technology-supported lifestyle interventions | The quality and quantity of published evidence to support the use of such interventions was low and unable to differentiate impact between types of interventions |
| Rhodes 2020 | GWG | Digital interventions targeting dietary behaviours or PA in pregnancy | - 7 BCTs identified in all effective interventions. These were goal setting (behavior), problem solving, review of behavior goals, feedback on behavior, social support, information about health consequences, and information about emotional consequences. - ‘Review of the behavior goal’ was the only BCT used exclusively in the 3 effective interventions. - Information-only interventions were ineffective - Effective interventions averaged over twice as many BCTs from the goals and planning, and feedback and monitoring domains as ineffective interventions. - Effective interventions also used interactivity, in the form of messages of encouragement, personalized feedback, and prompts to remind participants to use key BCTs, such as goal setting and self-monitoring, to promote engagement. |
|  |  |  | Meta-analyses showed no significant benefit of interventions on GWG for either intention-to-treat data (−0.28 kg; 95% CI −1.43 to 0.87) or per-protocol data (−0.65 kg; 95% CI −1.98 to 0.67) and analysis not possible to determine influence of interventions on diet and PA |
| Sherifali 2017 | GWG, Glycaemic status, nutritional measures, PA | eHealth technology with nutrition or PA) | - The overall analysis for any eHealth technology intervention resulted in a non-significant reduction in GWG (−1.62 kg, 95% CI −3.57 to 0.33) - The eHealth interventions that were effective in minimizing excessive weight gain during pregnancy comprised multiple components, including individualized text messaging and the use of social media |

# Supplement 11 – Smoking cessation Interventions (n=10)

| **Author** | **Outcome** | **Interventions** | **Findings relating to intervention features** |
| --- | --- | --- | --- |
| Acar 2022 | Smoking abstinence | Theory-based interventions | Difficult to mention clear findings about the effectiveness of theory-based interventions on this topic, studies have pointed out that interventions based on stages of change can be supportive. |
| Brown 2019 | Smoking status | Interventions with ≥1 intervention component  aimed to maintain smoking abstinence | - Effective interventions used self-help, mainly in conjunction with counselling, and were largely delivered remotely. - Six BCTs as promising: ‘problem solving’, ‘information about health consequences’, ‘information about social and environmental consequences’, ‘social support’, ‘reduce negative emotions’ and ‘instruction on how to perform a behaviour’. - No clear pattern as to which modes of delivery and timings of intervention contact would be best used to deliver intervention components most effectively. |
| Chamberlain 2017 (includes previous update of Chamberlain 2013) | Birth weight, pre-term delivery, mortality outcomes (still birth, neonatal mortality, neonatal intensive care unit (NICU) admission | Smoking cessation interventions | - Interventions were categorised as counselling, health education, feedback, incentives, social support, exercise and dissemination. - Counselling increased smoking cessation in late pregnancy compared with usual care (RR 1.44, 95% CI 1.19 to 1.73) and less intensive interventions (RR 1.25, 95% CI 1.07 to 1.47). - Borderline effect of health education compared with usual care (RR 1.59, 95% CI 0.99 to 2.55), but effect was unclear when compared with less intensive interventions (RR 1.20, 95% CI 0.85 to 1.70), and alternative interventions (RR 1.88, 95% CI 0.19 to 18.60) - Feedback increased smoking cessation when compared with usual care and provided in conjunction with other strategies, such as counselling (RR 4.39, 95%CI 1.89 to 10.21), but the confidence in the quality of evidence was downgraded to moderate as this was based on only two studies and the effect was uncertain when feedback was compared to less intensive interventions (RR 1.29, 95% CI 0.75 to 2.20). - Incentive-based interventions are effective when compared with an alternative (non-contingent incentive) intervention (RR 2.36, 95% CI 1.36 to 4.09). Pooled effects not calculable for comparisons with usual care or less intensive interventions. |
|  |  |  | - The intensity of support women received in both the intervention and comparison groups has increased over time, with higher intensity interventions more likely to have higher-intensity comparisons |
|  |  |  | - Effect is unclear in social support interventions provided by peers - Effect was unclear in single interventions of exercise compared to usual care (RR 1.20, 95% CI 0.72 to 2.01) and dissemination of counselling (RR 1.63, 95% CI 0.62 to 4.32). - Unclear whether incorporating use of self-help manuals or telephone support increased the effectiveness of intervention |
| Griffiths 2018 | Smoking abstinence reported | Digital interventions | - Digital interventions more effective than comparison groups in smoking cessation (OR 1.44, 95% CI 1.04 to 2.00, p=.03). - Computer-based (OR 3.06, 95% CI 1.28 to 7.33) and text-message interventions (OR 1.59, 95% CI 1.07 to 2.38) were the most effective digital platforms. - Moderator analyses revealed seven BCTs associated with smoking cessation: information about antecedents; action planning; problem solving; goal setting (behaviour); review behaviour goals; social support (unspecified); and pros and cons. - A meta-regression suggested that interventions using larger numbers of BCTs produced the greatest effects. |
| Heminger 2016 | Smoking cessation during pregnancy or additional elements related to general pregnancy health | mHealth cessation programs | Little evidence was identified to support their use. |
| Hussain 2020^a^ | Smoking cessation | Stand-alone mobile phone interventions | Some evidence of positive effects on health behaviors and beliefs, but very limited correlation with improved health outcomes. Data and interventions were heterogeneous, precluding a meta-analysis. |
| Su and Buttenheim 2014 | Smoking cessation. Identifying differences in quit and relapse rates between spontaneous and nonspontaneous quitter. Postpartum smoking relapse. | Smoking interventions that continued 1 month postpartum | - Though intervention groups showed consistently higher quit rates during pregnancy than control groups, none of the intervention types were effective at preventing relapse in the longer-term postpartum. - None of the behavioral interventions such as CBT counseling, telephone counseling, self-help materials, and peer or partner counselling proved successful in maintaining smoking abstinence postpartum. - Incentives provided a significant difference in quit rate and short-term postpartum abstinence in comparison to control groups. Unfortunately, once incentives were removed, abstinence rates declined. |
| Tahan 2023 | At least one abstinence outcome | Counselling, advice, coaching, home visitation or other psychological interventions that were delivered by nurses or midwives | - Nursing or Midwife-led counselling (NOMC) interventions had moderate certainty evidence to increase pregnant smokers achieve point prevalence abstinence (PPA) by 54% (95% CI 16% to 206%, p<.01) in late pregnancy compared with usual care. - Low certainty evidence that NoMC interventions increase PPA among pregnant smokers by 79% (95% CI 14% to 283%, p=.01) during the postpartum period. - DH interventions had moderate certainty evidence to increase CA by 98% (95% CI 8% to 364%, p=0.03) in late pregnancy. - Digital health (DH) interventions had low certainty evidence to increase PPA by 46% (95% CI 5% to 202%, p=0.02) during the postpartum period. |
|  |  |  | - For DH type interventions, short text message, computer and mobile application programmes that include contingency management (ie, financial reward for abstinence), 5As-based counselling, TTM-based counselling or gamified type behavioural interventions appeared to be more effective for promoting abstinence compared with usual care or information-only comparators. - NoMC interventions, such as face-to-face, the 5As, TTM and CBT-based counselling, are more effective in achieving abstinence compared with nurse home visitation programmes or motivational interviewing. - Counselling is observed to increase with increased frequency (eg, those who attend more sessions of counselling are more likely to achieve abstinence) and are more effective when added to other components such as self-help or information material, peer counselling, further support or other types of intervention such as DH or BF. |
|  |  |  | - NoMC interventions did not assist pregnant smokers in achieving continuous abstinence (CA) in late pregnancy or during postpartum. - Digital health (DH) interventions did not increase PPA among pregnant smokers in late pregnancy (37% (95% CI −10% to 207%, p=0.14) - Biofeedback (BF) interventions did not increase PPA among pregnant smokers in late pregnancy (32% (95% CI −25% to 232%, p=0.34)) nor CA during postpartum (5% (95%CI −16% to 30%, p=0.66)). |
| Wilson 2018 | Smoking cessation, abstinence at three dedicated time points. | Contingency management or psychotherapy | While psychotherapy did not affect smoking abstinence, contingency management interventions had significant between-group treatment effects at late pregnancy (Q 31.3, p<.001), early postpartum (Q 3.99, p<.05), and late postpartum (Q 10.69, p<.01). |

# Supplement 12 – Alcohol Interventions (n=6)

| **Author** | **Outcome** | **Interventions** | **Findings relating to intervention features** |
| --- | --- | --- | --- |
| Froschl 2013 | FAS, alcohol intake/ abstinence, fetal mortality, birth weight, gestational age, preterm, NICU | Short-term alcohol interventions | - Short-term interventions, in particular screening interventions and systematic surveillance of alcohol intake reduce alcohol abuse and increase alcohol abstinence in pregnant women - Improvement in control groups suggest application of a screening instrument and the verbalisation of alcohol abuse already reduce the alcohol intake during pregnancy |
|  |  |  | No evidence for the key conditions and for the key factors for the success of interventions. Primary studies give little details regarding the types of interventions and varying in duration and follow-up. |
| Gomez 2020 | Abstinence of alcohol in pregnancy and reduction of drinking in motherhood | Alcohol interventions | - A signiﬁcant treatment effect abstinence (OR 2.31, 95% CI 1.61 to 3.32; p<.001) but unclear that BCTs are contributing to these effects (most commonly used general and alcohol-speciﬁc BCTs included information about consequences, social support, goal setting and action planning) - Brief interventions and motivational approaches show the most promise to change alcohol-related behaviour in pregnancy, but further investigation is warranted as multiple sessions may be more effective, especially for lower drinking populations because of message repetition. |
| Minozzi 2024 | Abstinence from alcohol, reduction in alcohol consumption, retention in treatment, and women with any adverse event | Psychosocial and medication interventions | All eligible studies were brief interventions, which may increase the rate of continuously abstinent participants (RR 1.34, 95% CI 1.14 to 1.57). |
|  |  |  | BIs may have little to no effect on the number of drinks per day, but the evidence is very uncertain (MD −0.42, 95% CI −1.13 to 0.28) |
|  |  |  | Could not perform subgroup analyses for intensity or frequency of psychosocial interventions as the number of studies was too few |
| Oh 2022 | Reduction in alcohol consumption | Digital intervention aiming to prevent alcohol consumption | - Meta-analysis favored digital interventions in decreasing risk of alcohol consumption (OR 0.62, 95% CI 0.42 to 0.91; p=.02) - Computer/internet-based interventions (OR 0.59, 95% CI 0.38-0.93) were an effective platform for preventing alcohol consumption |
|  |  |  | Most effective interventions in our review were those that incorporated both offline house counseling and internet or mobile-based feedback (ie, “blended” care) for individuals |
|  |  |  | - Too few studies of text messaging (OR 0.29, 95% CI 0.29-2.52) were available to draw a conclusion. - Intervention duration, quality, and intensity could not be controlled for |
| Popova 2023 | Change in alcohol use (quantity and frequency).  Preterm delivery, NICU admission, birthweight, body length, head circumference, APGAR score | Brief alcohol intervention | Modest effects in favor of BIs by increasing the odds of abstinence by 56% (OR 1.56, 95% CI 1.15 to 2.13; p=.04) and a modest and significant reduction in preterm birth (OR 0.67, 95% CI 0.46 to 0.98; p=.58). |
|  |  |  | - No significant differences observed for mean drinks/week, AUDIT scores, mean birthweight or lower likelihood of LBW. - Fewer than the required number of studies to conduct sub-group analyses of the components of Bis that might be effective. |
| Samawi 2021 | Alcohol reduction | Psychological intervention | - Most psychological interventions consisted of BIs, motivational interviews or elements of both. There was considerable variability in the content and strategies, including the number and duration of sessions. - Interventions that showed some efficacy were longer in duration and required more frequent engagement with intervention content compared to studies or BIs typically delivered in a single session |
